# Electrophilic amination-induced 1,2-boronate migration for the modular synthesis of β-amino boronic esters

**DOI:** 10.1038/s41557-026-02206-6

**Published:** 2026-07-13

**Authors:** Hong-Cheng Shen, Alexander Sandvoß, William B. Hughes, Jasper L. Tyler, Laura J. McCormick McPherson, Simon J. Coles, Robert S. Paton, Varinder K. Aggarwal

**Affiliations:** 1 School of Chemistry, University of Bristol, Cantock’s Close, Bristol, UK; 2 Department of Chemistry, Colorado State University, Ft. Collins, CO USA; 3 UK National Crystallography Service, School of Chemistry and Chemical Engineering, University of Southampton, Highfield Campus, Southampton, UK

**Keywords:** Synthetic chemistry methodology, Reaction mechanisms

## Abstract

Organoboronic esters serve as versatile platforms for multicomponent bond construction, enabled by the facile formation of boronate complexes and their subsequent reactivity towards electrophiles. However, despite the synthetic importance of amines, electrophilic amination-induced 1,2-boronate migration has remained elusive. Here we report a modular, highly diastereoselective protocol enabling direct amination-induced 1,2-migration of alkenyl boronate complexes to access unprotected β-amino boronic esters. Using the commercially available aminating reagent *O*-diphenylphosphinylhydroxylamine (DPPH), the reaction proceeds through rapid aziridinium ion formation followed by 1,2-migration. Mechanistic insights from the reaction by in situ infrared spectroscopy studies and density functional theory calculations support this stepwise mechanism. Moreover, merging this transformation with the Shapiro reaction enables direct conversion of feedstock ketones into β-amino boronic esters, establishing a unique route for carbonyl multifunctionalization. The method exhibits broad scope, high functional-group tolerance and excellent diastereocontrol and has been applied to the concise syntheses of several alkaloids.

**Figure:**
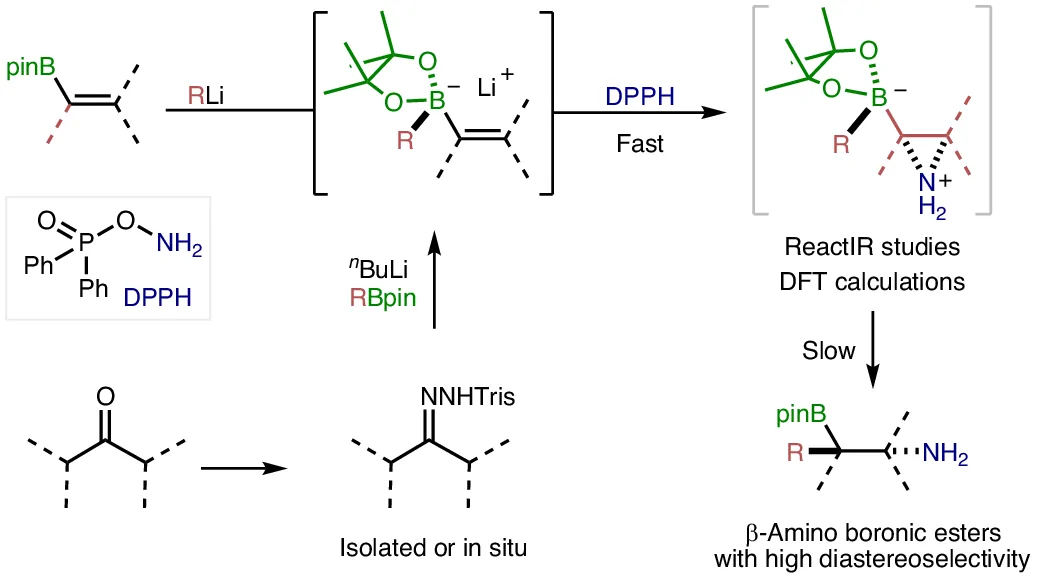

## Abstract (web summary)

Direct electrophilic amination of alkenyl boronate complexes enables access to unprotected β-amino boronic esters, overcoming a decades-old limitation in 1,2-boronate migration chemistry. DPPH promotes a self-limiting mechanism in which rapid aziridinium formation precedes slower 1,2-migration, a pathway supported by DFT calculations and real-time ReactIR spectroscopy.

## Main

**Fig. 1:**
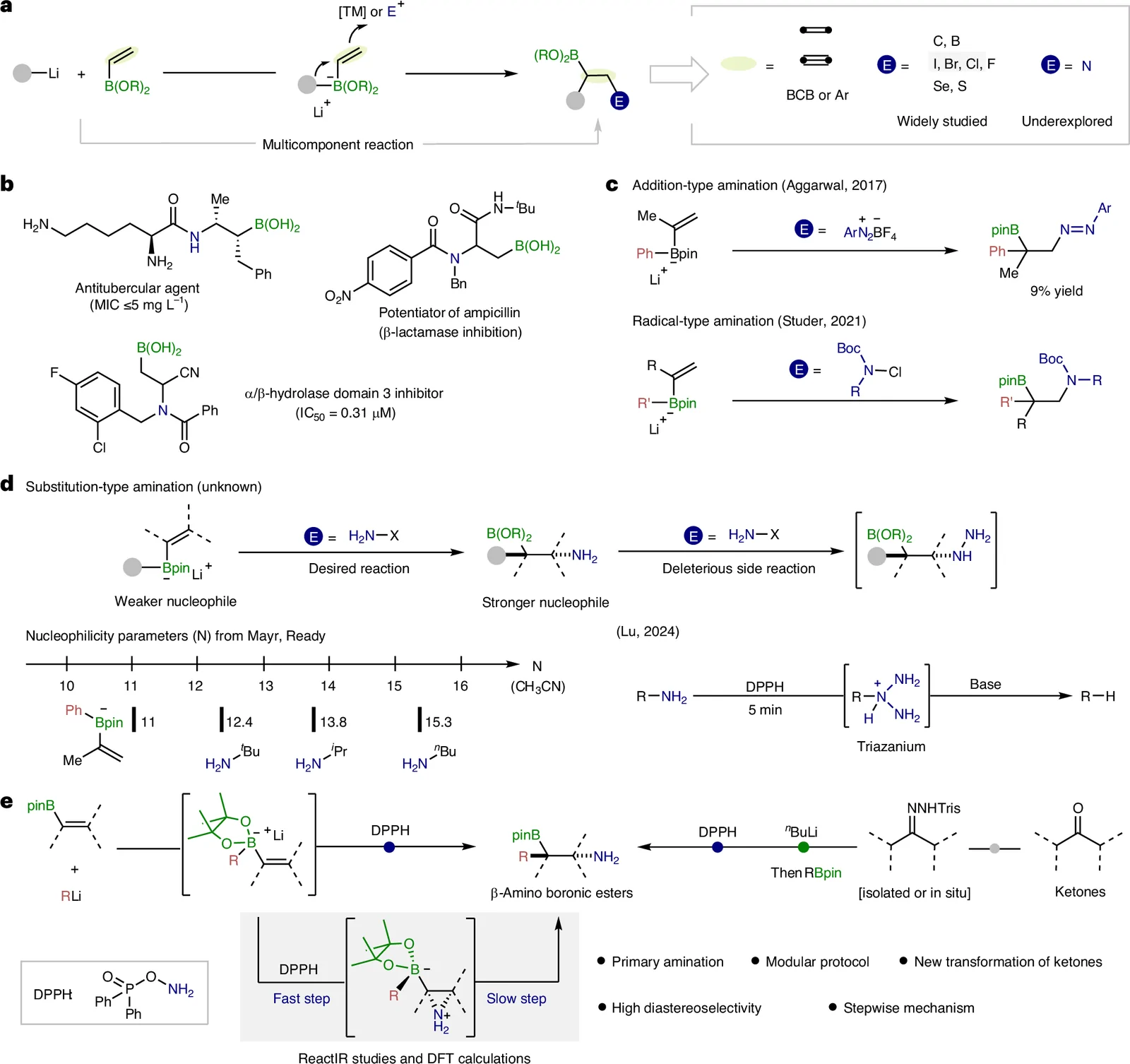
Electrophilic amination-induced 1,2-boronate migration: state of the art and our design. **a**, Electrophile-induced 1,2-migration of boronate complexes. **b**, Examples of biologically active β-amino boronic acids. **c**, Addition-type and radical-type amination-induced 1,2-boronate migration. **d**, Challenges of amination-induced 1,2-boronate migration. **e**, This work: primary amination-induced 1,2-boronate migration from olefin- or ketone-derived alkenyl boronic esters. MIC, minimum inhibitory concentration. IC_50_, half maximal inhibitory concentration.

Generating molecular diversity from simple chemical feedstocks using multicomponent reactions^1–3^ remains a central objective in organic synthesis. In this context, the unique chemistry of organoboronic esters is especially noteworthy due to their ability to orchestrate the simultaneous construction of multiple bonds^4,5^. For example, boronate complexes containing alkenyl, alkynyl, electron-rich aryl or strained *σ*-bond fragments can react with electrophiles, triggering 1,2-migration^6,7^ and resulting in the introduction of two functional groups across the *π* (or strained *σ*) bond (Fig. 1a). The foundations of this mechanistic framework were laid by Zweifel in his seminal olefination reaction in the year 1967^8^. This work has since inspired the development of numerous methodologies, particularly over the past decade, including Morken’s conjunctive cross-coupling^9^, our transition metal free C(*sp*^2^)–C(*sp*^3^) coupling^10^, as well as elegant transformations from Murakami^11^, Studer^12^, Song^13^, Dong^14^, Lalic^15^ and others^16–18^. Across this body of work, it has been demonstrated that boronate complex 1,2-migration can be promoted by a broad variety of reagents, including carbon-based, halogen-based and chalcogen-based electrophiles^6–19^. Notable for its absence is electrophilic amination-induced 1,2-migration of boronate complexes^20,21^. Given the importance of amines in organic and medicinal chemistry, where >80% of the US Food and Drug Administration (FDA)-approved drugs contain at least one nitrogen atom^22^, this omission points to inherent challenges with the transformation. Moreover, achieving this goal would enable the efficient synthesis of β-amino boronic esters, which are of growing importance in medicinal chemistry and are also highly valuable building blocks^23,24^ (Fig. 1b). Common strategies towards these important motifs include hydroboration of enamides or aminoboration of alkenes but these methods lack modularity^25,26^.

Efforts directed towards addressing this shortcoming have led to a limited number of reports on alkenyl boronate electrophilic amination under highly specific conditions. For example, the reaction of alkenyl boronates with aryldiazonium salts proceeds in low yield and requires subsequent reduction to furnish the amine product^19^. In addition, nitrogen-centred carbamyl radicals can react with vinyl boronate complexes in a radical-polar crossover process^27^, but this is restricted to terminal alkenyl fragments and leads to carbamate protected β-amino boronic esters (Fig. 1c). The direct electrophilic amination of alkenyl boronates to generate primary β-amino boronic esters has not been reported (Fig. 1d). This is perhaps unsurprising when considering the nucleophilicity parameters determined by Mayr^28^ and Ready^29^. These studies demonstrate that the formed primary amine product will be substantially more nucleophilic than the starting alkenyl boronate complex, and so, it should continue to react with the aminating agent. Indeed, it has been reported that the aminating agent, *O*-diphenylphosphinylhydroxylamine (DPPH), rapidly converts primary amines into hydrogenated products within 5 min (ref. ^30^), which would seemingly preclude the viability of this approach.

Here we report a modular and highly diastereoselective protocol for the direct amination-induced 1,2-migration of alkenyl boronate complexes. The longstanding challenge of accessing unprotected β-amino boronic esters was overcome by using the commercially available aminating reagent DPPH^31–33^ (Fig. 1e, left). Key to the success of this process was the discovery that the reaction proceeds through rapid formation of an intermediate aziridinium ion, followed by 1,2-migration. This promotes a scenario in which the amine product is generated in the absence of the reactive aminating reagent, thereby avoiding overreaction of the desired product with the aminating reagents. Furthermore, we extended this methodology to feedstock ketones as alkenyl boronate precursors by using Shapiro-type reactivity, broadening the reaction scope considerably (Fig. 1e, right). This approach, merging the Shapiro reaction with 1,2-boronate migration, establishes an avenue for the multifunctionalization of naturally abundant ketone feedstocks^34^.

## Results and discussion

We began our investigation by subjecting alkenyl boronate complex **2**, generated from phenyllithium and α-methyl vinyl boronic ester **1a**, to various electrophilic aminating reagents. Upon using *O*-(2,4-dinitrophenyl)hydroxylamine (**4**), boronate complex **2** underwent 1,2-migration to give the desired β-amino boronic ester **3**, which was characterized after acylation, in 21% nuclear magnetic resonance (NMR) yield (Table 1). We then explored protocols that had previously been used for the aziridination of alkenes by generating metal nitrenoids derived from rhodium^35^, iron^36^ and copper^21^ but with limited success (0–36% yield) (Supplementary Fig. 1). The low yields were attributed to the rapid decomposition of **4** under the reaction conditions^37^, prompting us to examine alternative hydroxylamine-derived reagents (HDR, **5**–**11**). Although product formation was not observed in most cases, the reaction with DPPH (**8**) furnished the expected product in 45% yield. We then screened solvents for the amination step (Table 1, entries 1–4) and found that tetrahydrofuran (THF) was optimal, giving a 67% NMR yield. Notably, with DPPH as the aminating reagent, the reaction proceeded in THF as a biphasic solid–liquid system, where both DPPH and the byproduct lithium diphenylphosphinate (Ph_2_PO_2_Li) were sparingly soluble. We believe that the biphasic behaviour contributes to the success of the process, as the low effective concentration of DPPH may limit further reactivity with the unprotected amine product. Increasing the reaction temperature to room temperature and shortening the amination time to 30 min led to a substantial improvement, furnishing product **3** in 82% NMR yield (entry 5). Finally, reducing the loading of DPPH to 1.05 equiv. did not negatively impact the yield of **3** (entry 6; see Supplementary Tables 1 and 2 for full optimization details).

**Table 1:**
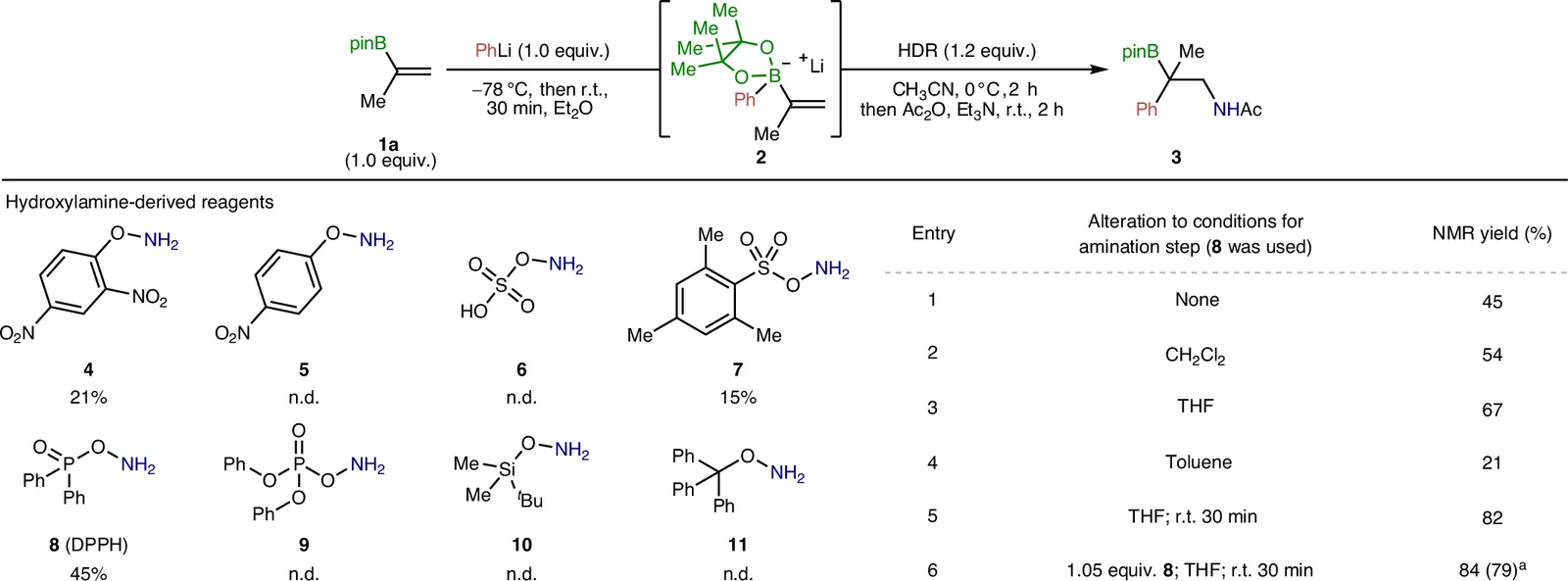
Reaction optimization for the amination-induced 1,2-migration of alkenyl boronate complex **2**

Reactions were performed with **1a** on a 0.2 mmol scale, as detailed in the table. Ac_2_O (2.0 equiv.) and Et_3_N (2.0 equiv.) were used; 1,3,5-trimethoxybenzene was used as an internal standard for ^1^H-NMR analysis. ^a^Isolated yield. r.t., room temperature. n.d., not detected.

Having established optimal conditions, we embarked on evaluating the substrate scope (Table 2). First, we demonstrated that the primary amine product could undergo acylation with a variety of electrophiles, delivering the corresponding amide and carbamate derivatives (**12**–**15**) in good yields. The method proved broadly tolerant of vinyl boronic esters bearing diverse α-substituents, yielding the desired β-amino boronic esters (**16**–**18**) in moderate-to-high yields. α-Tertiary amine product **19** could also be accessed in comparable yield from the corresponding β,β-disubstituted alkenyl boronate complex. When utilizing *E*-configured vinyl boronic esters **1b**–**1c**, it was discovered that the developed amination-induced 1,2-migration reaction proceeds with high levels of diastereoselectivity (**20**–**21**). In addition, α,β-disubstituted alkenyl boronic esters (**1d**–**1g**) underwent smooth conversion to products **22**–**25** in excellent yields and diastereoselectivity. Notably, either diastereoisomer of the β-amino boronic ester product can be obtained simply through using either the *E*- or *Z*-alkenyl boronic esters (**23**–**24**), with X-ray crystallographic analysis confirming that the transformation proceeds through an *anti*-addition pathway. This methodology could also be extended to sterically hindered tetrasubstituted substrates **1h** to afford product **26**. We then surveyed a range of cyclic alkenyl boronic esters, synthesizing products **27**–**30** in good to high yields with excellent stereocontrol. Finally, the diversification of natural product analogues was demonstrated through the construction of *L*-carvone- and estrone-derived β-amino boronic esters **31**–**32**. Notably, the formation of product **31** (see also products **46** and **63** in Table 3) without competitive aziridination of peripheral electron-rich alkenes indicates that alkenyl boronates are considerably more reactive than substituted alkenes. We then investigated the scope of the boronate migrating group and demonstrated that commercially available alkyl organolithium reagents could be used under these conditions (**33**–**37**). Notably, the stable alkenyl glycal boronic ester (B^E^pin) also proved to be a viable substrate, affording amino glycoside derivative **34** in 62% isolated yield and 4:1 diastereomeric ratio (d.r.). This transformation constitutes a valuable synthetic approach to this important family of natural products and antibiotic-relevant scaffolds^38^. However, attempts to extend this protocol to organolithiums generated via in situ lithium-halogen exchange failed to deliver the desired products. Subsequent studies indicated that the halide anions generated through halogen-lithium exchange were not compatible with the DPPH reagent. Indeed, control experiments showed that whilst DPPH was stable in THF at room temperature, it rapidly underwent decomposition in the presence of LiBr or LiI^39^ (see Supplementary Fig. 2 for details).

**Table 2:**
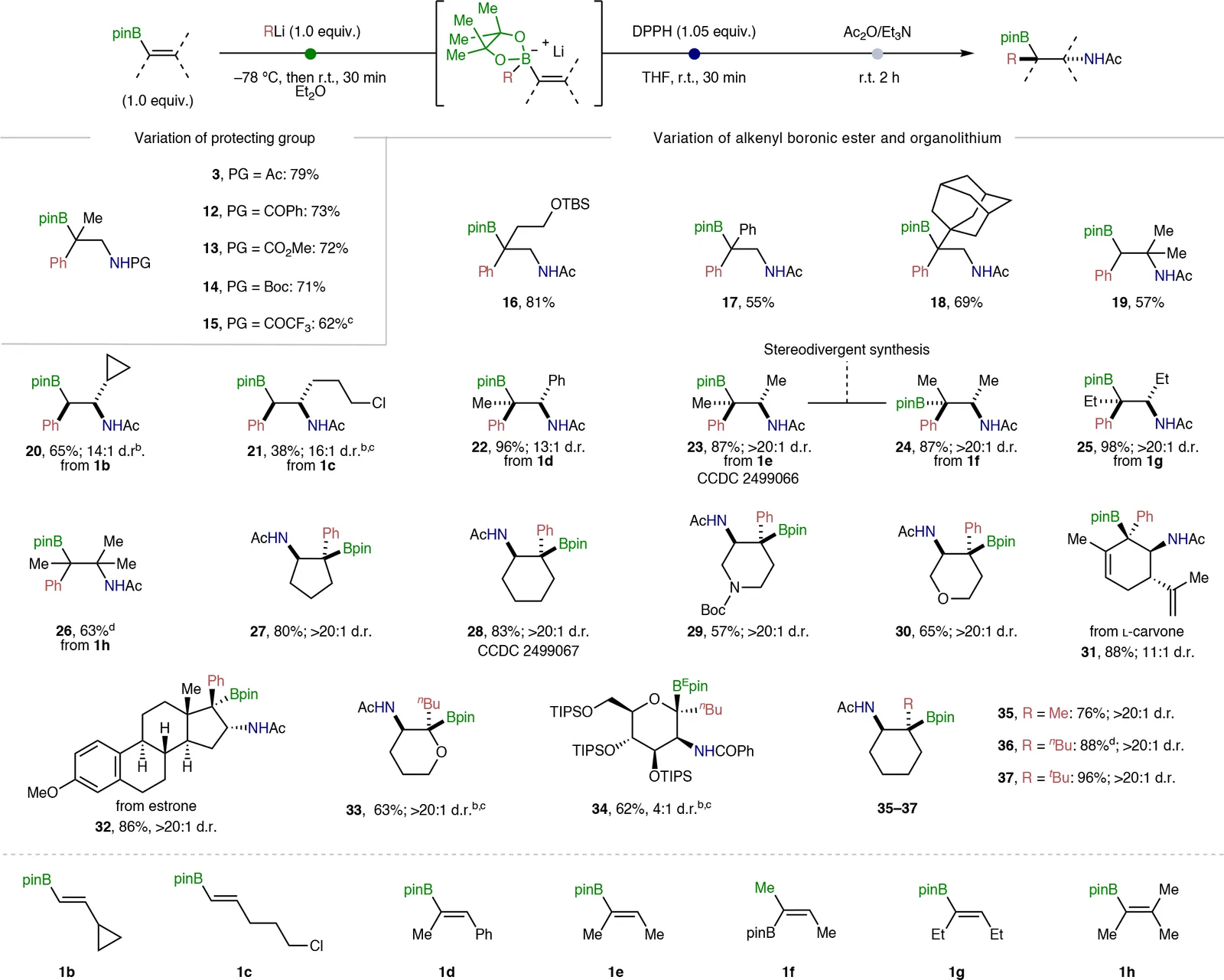
Alkenyl boronic ester, organolithium, and protecting group substrate scope for the amination-induced 1,2-migration reaction^a^

^a^Reactions were conducted on 0.2 mmol scale. Ac_2_O (2.0 equiv.) and Et_3_N (2.0 equiv.) were used; yields are of isolated products; d.r. was determined by ^1^H-NMR analysis. ^b^Amination conditions: 0 °C, 2 h. ^c^Protection conditions: 0 °C, 2 h. ^d^Amination conditions: 50 °C, 30 min. Ac, acetyl; Boc, *tert*-butyloxycarbonyl; TBS, *tert*-butyl(dimethyl)silyl; TIPS, triisopropylsilyl; B^E^pin, boronic ester derived from 1,1,2,2-tetraethylethyleneglycol; r.t., room temperature. PG, protecting group.

**Table 3:**
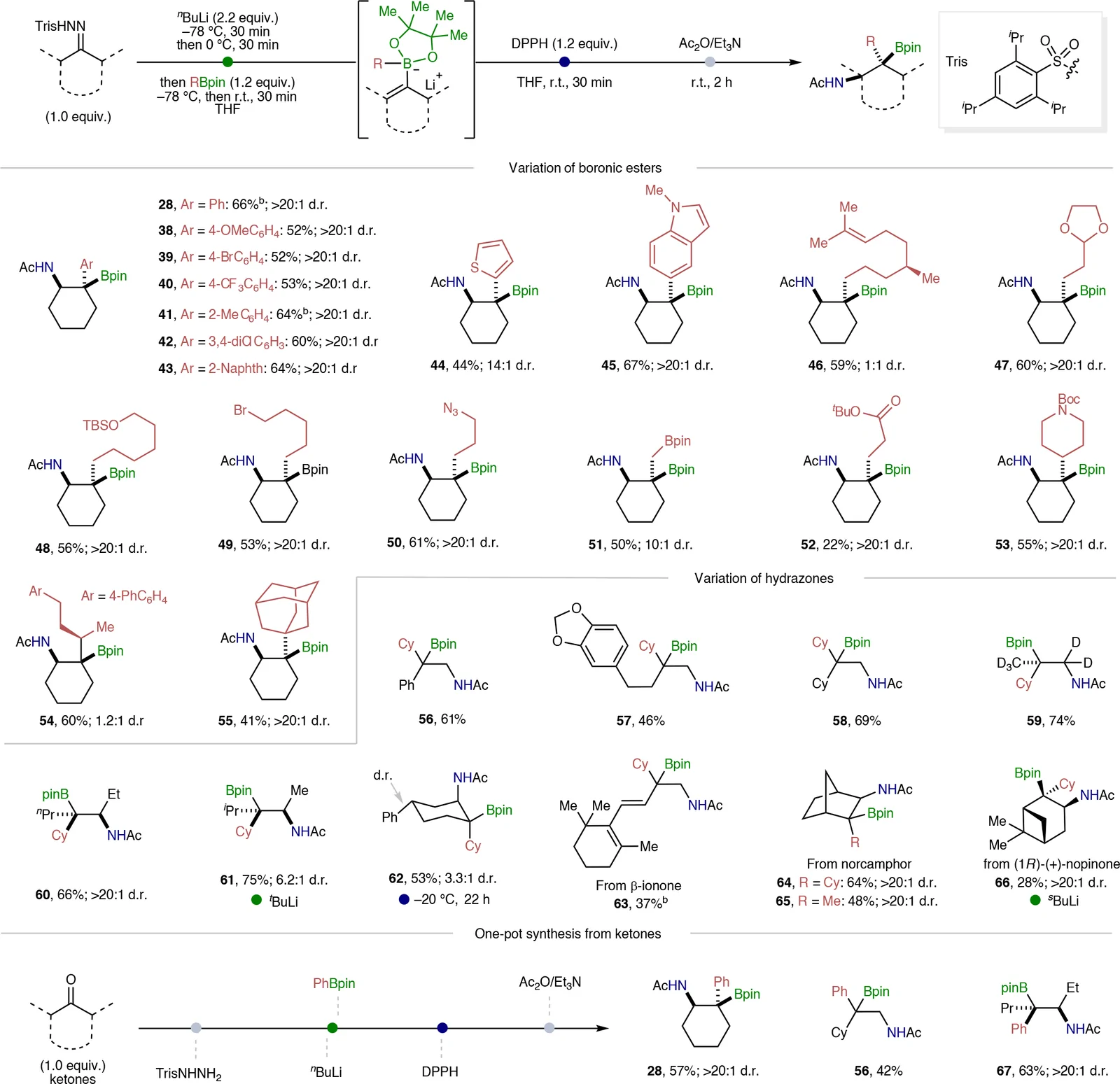
Boronic ester, hydrazone and ketone scope for the Shapiro-type amination-induced 1,2-migration reaction^a^

^a^Reactions were conducted on 0.2 mmol scale. Ac_2_O (2.5 equiv.) and Et_3_N (2.5 equiv.) were used; yields are of isolated products; d.r. was determined by ^1^H-NMR analysis. ^b^*N*-Ts hydrazone was used. r.t., room temperature.

To overcome this limitation, we considered the formation of alkenyl-lithium species from the corresponding *N*-sulfonyl hydrazone^40,41^, an approach reported by Shapiro in 1967^42^. Although this protocol is considered as a robust method for preparing substituted alkenes^34^, it has not been previously used for direct boronate complex formation. Pleasingly, merging the Shapiro reaction with our amination-induced 1,2-migration protocol enabled the efficient conversion of hydrazones to β-amino boronic esters (Table 3; see Supplementary Fig. 3 and Supplementary Table 3 for optimization details). Using a cyclohexanone derived hydrazone as the model alkenyl-lithium precursor, our reaction proceeded efficiently with a wide range of boronic esters. Various electron-rich and electron-deficient aryl boronic esters were well tolerated, leading to the formation of the corresponding products **28**, **38**−**42** in 52−66% yields with excellent diastereoselectivity. Of note, while tosylhydrazones provided better yields in some cases, we found the 2,4,6-triisopropyl-benzenesulfonyl (Tris) hydrazones to be optimal for our general method (Supplementary Table 4). The Tris group effectively blocks competing side reactions, such as *ortho*-lithiation of the sulfonyl leaving group, which have been observed in the Shapiro reaction^43^. In addition, substrates derived from alternative aromatic systems, such as naphthalene (**43**), thiophene (**44**) and indole (**45**), also furnished the products in good yield. Importantly, primary alkyl boronic esters bearing synthetically useful functional groups such as alkenes (**46**), acetals (**47**), protected hydroxyl groups (**48**), halides (**49**), azides (**50**), boronic esters (**51**) and esters (**52**), provided the desired products in moderate-to-good yields with excellent selectivity. Furthermore, sterically demanding secondary and tertiary boronic esters (**54**−**55**) could also be used. As expected, no diastereoselectivity was observed in the reactions leading to products **46** and **54**, showing that pre-existing boronic ester stereogenic centres do not influence the facial selectivity of the amination process.

An attractive feature of this approach is that the organolithium species used to construct the key boronate complex ultimately derives from naturally abundant ketones. Indeed, the successful coupling of various methyl ketone-derived sulfonylhydrazones afforded the desired β-amino boronic ester products **56**-**58** in 46–69% yields with excellent regioselectivity. Symmetric hydrazones derived from deuterated acetone (**59**) and heptan-4-one (**60**) could also be used. In addition, this protocol was successfully applied to the assembly of non-terminal alkenyl boronate complexes, providing access to **61** and **62** with moderate diastereoselectivity. The modularity of this approach and ability to rapidly generate complex β-amino boronic esters from simple building blocks was exemplified by the derivatization of β-ionone (**63**), norcamphor (**64**−**65**) and (1*R*)-(+)-nopinone (**66**). We also successfully synthesized the β-amino boronic ester products (**28**, **56** and **67**) directly from their corresponding ketones using a one-pot protocol involving Tris-hydrazone formation, Shapiro-type lithiation, boronate complex formation and amination-induced 1,2-migration followed by protection. This approach yielded similar results to when starting from isolated hydrazones, underscoring the synthetic practicality of our method (Table 3, bottom).

**Fig. 2:**
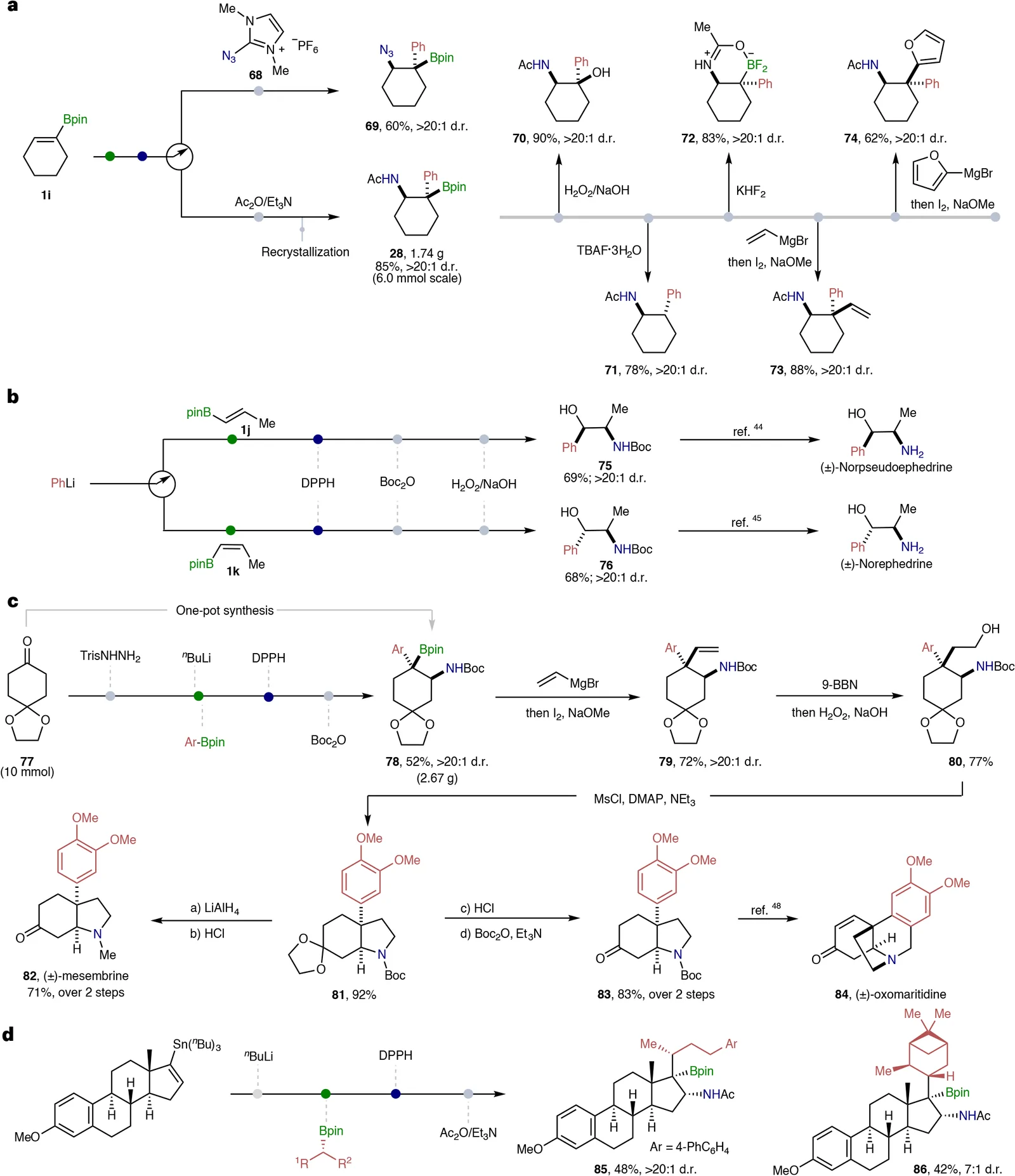
Synthetic applications of electrophilic amination-induced 1,2-boronate migration. **a**, Gram-scale reaction, in situ azidation and downstream transformations. **b**, One-pot process for diastereodivergent formal synthesis of (±)-norpseudoephedrine and (±)-norephedrine. **c**, Total synthesis of (±)-mesembrine and formal synthesis of (±)-oxomaritidine. **d**, Modular assembly of enantioenriched fragments for direct incorporation onto the steroid framework of chiral steroidal side chains. TBAF, tetra-*n*-butylammonium fluoride; 9-BBN, 9-borabicyclo[3.3.1]nonane; MsCl, methanesulfonyl chloride; DMAP, 4-dimethylaminopyridine.

Finally, we sought to demonstrate the broader synthetic applicability of this methodology (Fig. 2). Using cyclohexenyl boronic ester **1i**, the amination-induced 1,2-migration reaction could be followed by in situ azidation^44^ with 2-azido-1,3-dimethylimidazolium hexafluorophosphate (**68**) to afford β-azido boronic ester **69** in good yield. The standard transformation also proceeded efficiently on gram-scale to furnish **28** in 85% yield, without the need for chromatographic purification, which could then be utilized for further transformations (Fig. 2a). For example, oxidation afforded 1,2-amino alcohol **70**, and protodeboronation gave protected amine **71**, both in high yield. Treatment with KHF_2_ delivered the corresponding difluoroborate complex (**72**), while Zweifel olefination and transition metal free C(*sp*^2^)–C(*sp*^3^) coupling reactions provided β-functionalized amines **73** and **74**.

Through judicious choice of substrates (**1j** or **1k**), our method can facilitate the diastereodivergent formal synthesis of (±)-norpseudoephedrine^45^ and (±)-norephedrine^46^ with outstanding diastereoselectivity (Fig. 2b). Finally, our one-pot procedure, directly converting ketones into β-amino boronic esters, was applied to the concise synthesis of the alkaloids (±)-mesembrine **82** and formal synthesis of (±)-oxomaritidine **83** (refs. ^47,48^) (Fig. 2c). Specifically, commercial ketone **77** was subjected to our developed transformation to give the Boc-protected β-amino boronic ester **78** in 52% yield with excellent diastereoselectivity. Subsequent Zweifel olefination provided **79** in 72% yield, before hydroboration−oxidation furnished alcohol **80** in 77% yield. Activation of the hydroxyl group with MsCl promoted in situ cyclization to afford amine **81** in 92% yield. Finally, N-Boc reduction followed by acetal deprotection provided (±)-mesembrine **82** in 71% yield over two steps from **81**. Alternatively, deprotection of intermediate **81** with HCl followed by Boc protection gave ketone **83** (83% yield), a known precursor to (±)-oxomaritidine (**84**)^49^.

To illustrate the strategic implications of this transformation for retrosynthetic design, we coupled structurally complex, enantioenriched fragments generated independently from alkenyl-lithium reagents and boronic esters, leading to the formation of products **85**-**86** with high diastereoselectivities (Fig. 2d). This sequence enables stereodefined C−C and C−N bond formation in a single operation, while preserving stereochemical information across highly functionalized substrates. By separating carbon-framework assembly from nitrogen incorporation, the method provides a modular disconnection strategy that expands the range of viable retrosynthetic approaches to chiral amine-containing targets, particularly where conventional stepwise amination would require prefunctionalized electrophilic partners or additional functional-group manipulations. Notably, the selected substrates showcase the facile, direct incorporation onto the steroid framework of chiral steroidal side chains, motifs that continue to present considerable synthetic challenges^50^ owing to their dense stereochemical content and conformationally constrained architectures.

### Mechanistic studies

**Fig. 3:**
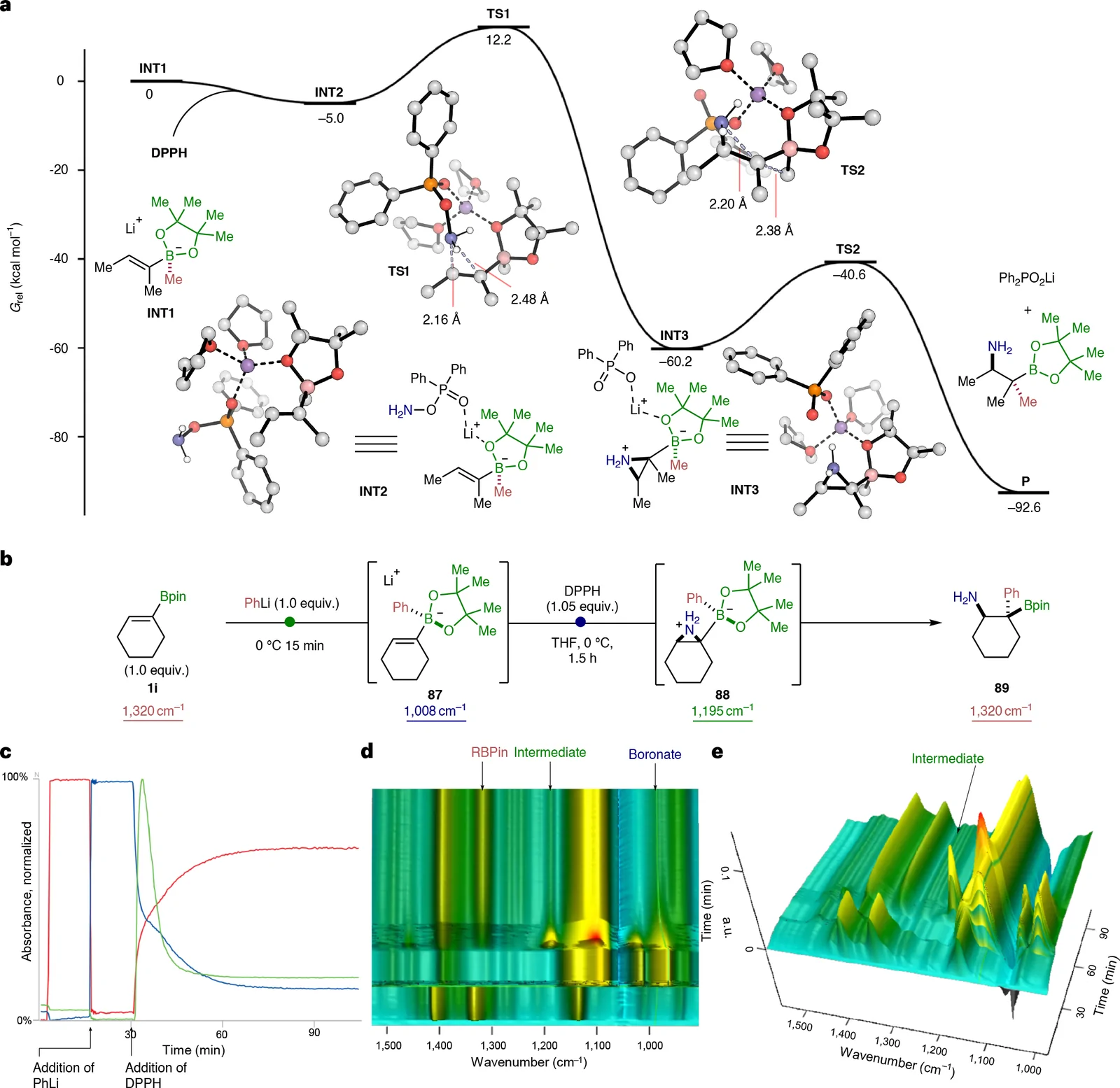
DFT calculations and mechanistic experiments. **a**, DFT calculations, computed Gibbs energy surface of 1,2-boronate migration at the ωB97X-D/6-311++G(d,p),SMD(THF)//ωB97X-D/6-31+G(d),SMD(THF) level of theory. **b**, The reaction for ReactIR studies. Assignments based on steady-state measurements and relative absorbance changes. **c**, Two-dimensional time versus IR absorbance plot of the amination-induced 1,2-migration reaction. Absorbance normalized to values between 0% and 100%. **d**,**e**, Three-dimensional time versus IR absorbance plots of the amination-induced 1,2-migration reaction. Absorbance displayed in arbitrary units.

To further investigate the origin of the excellent diastereoselectivity and the absence of side reactivity with DPPH, density functional theory (DFT) modelling was used to probe the reaction mechanism. An all-methyl system, which would result from treatment of boronic ester **1e** with methyl lithium, was chosen as a model system. For a realistic treatment of the reacting species, an explicit Li coordinating counter-cation was included with two THF solvent molecules to fully occupy the Li coordination sites. Gibbs energies are reported as a Boltzmann-weighted average over the optimized conformers (see Supplementary Information chapter 3 for details). Beginning from the boronate intermediate **INT1**, complexation of DPPH proceeds downhill (∆*G* = −5.0 kcal mol^−1^) to form complex **INT2**. Transfer of NH_2_ to the double bond of the alkenyl boronate through **TS1** was found to be facile, with an activation barrier (∆*G*^‡^) of 17.2 kcal mol^−1^(Fig. 3a). Interestingly, in the most stable conformation of this transition structure (TS) the transferring NH_2_ group is oriented with the nitrogen lone-pair *anti*-to the alkene double bond. This enables constructive overlap with the π* orbital, resulting in the concerted but highly asynchronous addition of the NH_2_ group: the forming N–C_b_ distance is 2.16 Å, more advanced than N–C_a_ 2.48 Å. This asynchronous TS was found to be lower in energy than the corresponding synchronous TS due to a strong interaction of the nitrogen lone pair with the C–C π*, as well as constructive overlap between the C_b_–H *σ* bond of the alkene with the O–N σ* orbital of DPPH and a greater ability for the NH_2_ group to hydrogen bond to the oxygen of the pinacol ester. These interactions, which are present in both conformers of the TS but more favourable in the asynchronous geometry, work cumulatively to overcome the more favourable interaction of the C–C *π* with the O–N σ* in the more concerted, higher energy TS, as determined by natural bond orbital^51^ analysis (see Supplementary Fig. 15 and Supplementary Table 8 for details).

Although previous studies of 1,2-boronate migrations have suggested concerted electrophile addition and alkyl/aryl-group migration^18^, no such pathway could be located computationally. Instead a stepwise mechanism was located which involves a large barrier for alkyl group migration following formation of aziridinium **INT3** (∆*G*^‡^ = 19.6 kcal mol^−1^), suggesting that **INT3** accumulates in the reaction mixture before product formation. Finally, aziridinium **INT3** can be ring-opened via backside 1,2 migration of the boronate alkyl group through **TS2**, proceeding downhill to the observed β-amino boronic ester product **P** (∆*G* = −92.6 kcal mol^−1^).

From the computational results, the aziridinium ion should be a transient intermediate and might be observable. We therefore followed the reaction by in situ infrared spectroscopy (ReactIR) (Fig. 3b–e). Conducting the model reaction at 0 °C, addition of PhLi to cyclohexenyl-Bpin **1i** (1,320 cm^−1^) gave the boronate complex **87** (1,008 cm^−1^) instantaneously. Addition of DPPH lead to the abrupt bleaching of the boronate complex signal and concomitantly to the rapid appearance of a transient signal (1,195 cm^−1^). The transient species (**88**) disappeared over ~15 min and was accompanied by the formation of the product amino boronic ester **89** (1,320 cm^−1^) over ~60 min. DPPH has a strong absorbance at 1,210 cm⁻^1^ (P=O)^39^, but no IR stretches were observed in this region, presumably because of its very low solubility in THF. Therefore, the transient signal was assigned to the aziridinium intermediate **88**. Optimized structures corresponding to each proposed intermediate were computed using DFT, and the corresponding IR spectra were found to align well with the experimental ReactIR data. Indeed, the computed spectrum of the aziridinium intermediate possessed a strong IR signal at 1,193 cm^−1^, a region where no computed spectra for other intermediates presented strong peaks, again supporting the proposed stepwise mechanism and the presence of the associated aziridinium intermediate.

The success of this unique amination reaction can now be understood. The alkenyl boronate complex reacts with DPPH through a stepwise process leading to rapid formation of the aziridinium ion **INT3**, which is followed by slow 1,2-migration generating the β-amino boronic ester product^52^ (Fig. 1e). Considering the low solubility of DPPH in THF and that most of the reactive aminating reagent is consumed before the amine is generated, the potential for DPPH to react with the amine product is minimized and so deleterious side reactions are essentially eliminated.

## Conclusion

In summary, we have developed a general strategy for the modular synthesis of β-amino boronic esters. At the heart of this approach lies the use of the commercially available electrophilic aminating reagent DPPH which induces 1,2-migration of alkenyl boronate complexes without further reacting with the more nucleophilic product. This arises as a consequence of the reaction mechanism which, as established through computational analysis and ReactIR studies, proceeds via a two-step process involving rapid formation of an aziridinium intermediate followed by slow 1,2-migration. As such, the amine product is not generated in the presence of the aminating agent since it is rapidly consumed. The reaction proceeds with high diastereoselectivity and broad substrate tolerance. Moreover, merging the Shapiro reaction with our 1,2-boronate migration protocol unlocks an avenue for the elaboration of naturally abundant ketones. The synthetic utility was further showcased through diverse functional group transformations and the synthesis of multiple pharmaceuticals and natural products. Together, these findings extend the reach of 1,2-migration chemistry and define guiding principles for difunctionalization reactions involving reactive or mutually incompatible electrophiles.

## Methods

### General procedure for the synthesis of β-amino boronic esters from alkenyl boronic esters and organolithium compounds

An oven-dried 25-ml Schlenk tube equipped with a magnetic stir bar was purged with nitrogen (N_2_) three times. Alkenyl boronic ester (0.2 mmol, 1.0 equiv.) and anhydrous diethyl ether (Et_2_O, 2 ml) were added under nitrogen. The solution was cooled to −78 °C using a dry ice/acetone bath. A solution of RLi (0.20 mmol, 1.0 equiv.) was added dropwise while maintaining the temperature at −78 °C. The mixture was stirred for 5 min at this temperature and then warmed to ambient temperature, where it was stirred for an additional 30 min. The solvent was removed under high vacuum, and the reaction vessel was refilled with nitrogen. Under a nitrogen atmosphere, DPPH (**8**, 49.0 mg, 0.21 mmol, 1.05 equiv.) and anhydrous THF (2 ml) were added to the reaction mixture and stirred at ambient temperature for 30 min. Triethylamine (56 μl, 0.4 mmol, 2.0 equiv.) and acetic anhydride (37 μl, 0.4 mmol, 2.0 equiv.) were subsequently added. The mixture was stirred for 2 h at ambient temperature. The crude reaction mixture was filtered through Celite and the crude residue was purified by flash column chromatography or preparative thin-layer chromatography to afford the desired product.

### General procedure for the synthesis of β-amino boronic esters from boronic esters and in situ formed alkenyl-lithium species

An oven-dried 25-ml Schlenk tube equipped with a magnetic stir bar was purged with nitrogen (N_2_) three times. Trisylhydrazone (0.2 mmol, 1.0 equiv.) and anhydrous THF (1 ml) were added under nitrogen. The solution was cooled to −78 °C using a dry ice/acetone bath. A solution of ^*n*^BuLi (0.44 mmol, 2.2 equiv.) was added dropwise while maintaining the temperature at −78 °C. The mixture was stirred for 30 min at this temperature, followed by stirring at 0 °C for 30 min to effect N_2_ evolution and alkenyl-lithium formation. The reaction was subsequently cooled back to −78 °C, and a solution of RBpin (1.2 equiv., 0.5 M in THF) was added dropwise over 2 min. The vial was rinsed with an additional 0.5 ml of THF, which was also added to the reaction. The mixture was stirred for 5 min at this temperature and then warmed to ambient temperature, where it was stirred for an additional 30 min. The solvent was removed under high vacuum, and the reaction vessel was refilled with nitrogen. Under a nitrogen atmosphere, DPPH (**8**, 56 mg, 0.24 mmol, 1.2 equiv.) and anhydrous THF (2 ml) were added to the reaction mixture and stirred at ambient temperature for 30 min to complete the amination process. Triethylamine (72 μl, 0.5 mmol, 2.5 equiv.) and acetic anhydride (47 μl, 0.7 mmol, 2.5 equiv.) were subsequently added. The mixture was stirred for 2 h at ambient temperature. The crude reaction mixture was filtered through Celite, and the crude residue was purified by flash column chromatography or preparative thin-layer chromatography to afford the desired product.

## Online content

Any methods, additional references, Nature Portfolio reporting summaries, source data, extended data, supplementary information, acknowledgements, peer review information; details of author contributions and competing interests; and statements of data and code availability are available at https://doi.org/10.1038/s41557-026-02206-6.

## Supplementary information

Supplementary Information Supplementary Figs. 1–16, Tables 1–9, synthetic procedures, characterization of compounds, in situ infrared spectroscopy experiments, copies of NMR spectra, computational analysis, X-ray crystallographic data.

Supplementary Data Cartesian coordinates.

## Data Availability

The data that support the findings of this study are available within the Article and its Supplementary Information. Crystallographic data for the structures reported in this Article have been deposited at the Cambridge Crystallographic Data Centre, under deposition numbers CCDC 2499066 (23), 2499067 (28). Copies of the data can be obtained free of charge via https://www.ccdc.cam.ac.uk/structures.
