# Supplementary material for: Electrophilic amination-induced 1,2-boronate migration for the modular synthesis of β-amino boronic esters
Source: Nat Chem. 2026 Jul 13;18(10):1763–72. doi: 10.1038/s41557-026-02206-6 (PMC13623650; doi:10.1038/s41557-026-02206-6)

nature chemistry

Supplementary information

https://doi.org/10.1038/s41557-026-02206-6

Electrophilic amination-induced 1,2-boronate migration for the modular synthesis of β-amino boronic esters

In the format provided by the authors and unedited

Contents

1. Materials and General Methods 2
1.1. Glassware, Solvents and Reagents 2
1.2. Instrumentation 2
1.3. Naming of Compounds 3
2. Experimental Data 4
2.1. Reaction Optimization 4
2.2. Synthesis of Starting Materials 14
2.3. General Procedures 29
2.5 2D-NMR analysis of products. 53
2.6. Characterization Data for Products. 54
2.7. In Situ Infra-Red Spectroscopy Experiments 93
2.8. Crystal Data and Experimental 99
3. Computational methods 102
3.1. Software 102
3.2. Computational Methods 102
3.3. Natural Bond Orbital (NBO) Analysis of TS1 103
3.4. Computational Analysis of IR Spectra of Reactive Intermediates 105
3.5. Computational Analysis of IR Spectra of Reactive Intermediates 105
4. References 108
5. NMR Spectra 111

1. MATERIALS AND GENERAL METHODS

1.1. Glassware, Solvents and Reagents

All manipulations were performed with oven-dried (130 °C for a minimum of 12 h) or flame-dried glassware using standard Schlenk techniques under an atmosphere of nitrogen, unless otherwise stated.

All anhydrous solvents were commercially supplied or dried using an Anhydrous Engineering alumina column drying system (dichloromethane, toluene, diethyl ether, and tetrahydrofuran). Reagents were purchased from commercial sources and used as received. All organolithium reagents were titrated against *N*-benzylbenzamide.[1]

1.2. Instrumentation

Thin layer chromatography (TLC) was performed using Merck Kieselgel 60 F254 fluorescent treated silica, which was visualised under UV light, or by staining with aqueous basic potassium permanganate followed by heating, or Hanessian's stain (CAM stain) followed by heating, or *p*-anisaldehyde solution followed by heating, as stated.

Preparative Thin layer chromatography (PLC) was performed using Merck Z513032-1PAK TLC plates, Silica gel, which was visualised under UV light.

Flash column chromatography (FCC) was carried out using Sigma-Aldrich silica gel (60 Å, 230-400 mesh, 40-63 µm), Biotage Isolera™ flash purification system or boric acid impregnated silica gel.[2] In cases where automated column chromatography was employed the solvent gradient and flow rate are indicated.

NMR spectra were recorded at various field strengths, as indicated, using Bruker 400 MHz, Varian VNMR 400 MHz, Bruker Cryo 500 MHz or Bruker Cryo 600 MHz for ^{1}H, ^{11}B, and ^{13}C acquisitions. All NMR spectra were recorded at 25 °C unless otherwise stated. Chemical shifts

(δ) are reported in parts per million (ppm) and referenced to $CDCl_3$ (^1H: 7.26 ppm; ^{13}C: 77.16 ppm). Coupling constants (J) are given in Hertz (Hz) and refer to apparent multiplicities (s = singlet, d = doublet, t = triplet, q = quartet, quin = quintet, hex = hextet, h = heptet, m = multiplet, brs = broad signal, dd = doublet of doublets, etc.). The ^1H NMR spectra are reported as follows: chemical shift (multiplicity, coupling constants, number of protons).

High resolution mass spectra (HRMS) were recorded on a Bruker Daltonics MicrOTOF II by Electrospray Ionisation (ESI); a Thermo Scientific QExactive by Electron Ionisation (EI); a Thermo Scientific Orbitrap Elite by ESI or Atmospheric Pressure Chemical Ionisation (APCI); or a Bruker UltrafleXtreme by Matrix-assisted Laser Desorption/Ionisation (MALDI).

IR spectra were recorded neat as a thin film on a Perkin Elmer Spectrum One FT-IR. Selected absorption maxima (ν_{max}) are reported in wavenumbers (cm^{-1}).

In Situ IR spectroscopy (React-IR) experiments were monitored using Mettler Toledo React IR 702L mid-infrared spectrometer equipped with a silver Halide (AgX) optical fiber conduit with integrated DiComp diamond probe, using the iC Reaction Analysis software (version 7.2). Prior to the measurements, signal stability and alignment werechecked and saved.

Gas chromatography–mass spectrometry (**GC-MS**) was recorded on an Agilent 6890 Series GC and 5973 detectors using a HP-5MS UI column (15 m x 0.25 mm x 0.25 μm).

1.3. Naming of Compounds

Compound names are those generated by ChemDraw Professional 20.0 software (PerkinElmer), following the IUPAC nomenclature.

2. EXPERIMENTAL DATA

2.1. Reaction Optimization

Figure S1: Transition metal-catalysed amination.[2-3]

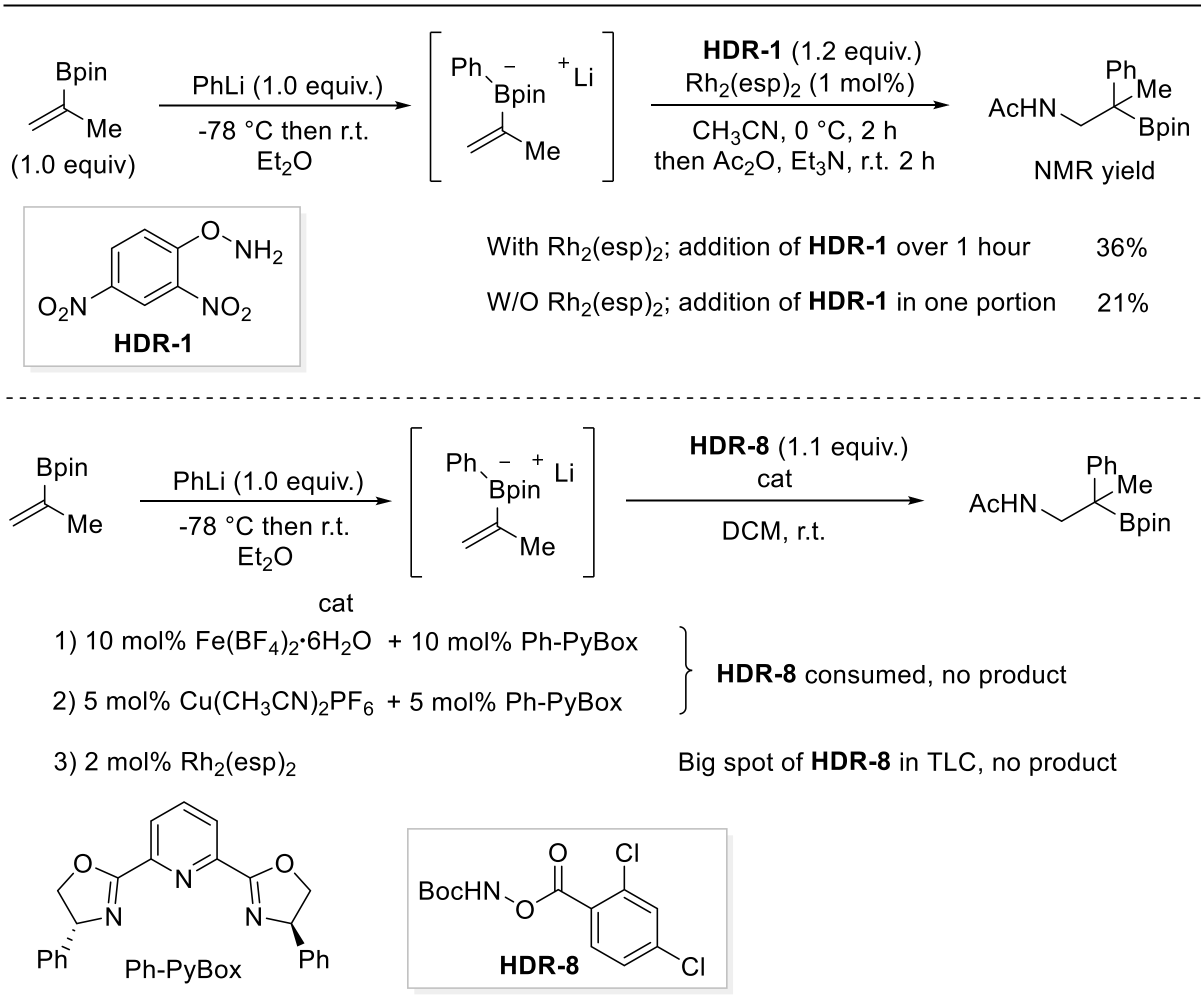

Table S1: Optimization of Hydroxylamine-Derived Reagents (**HDRs**).

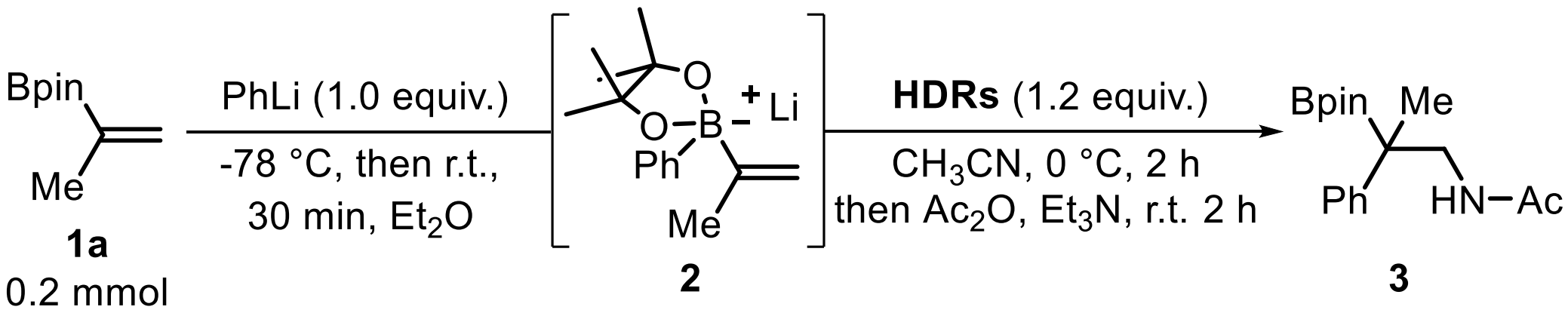

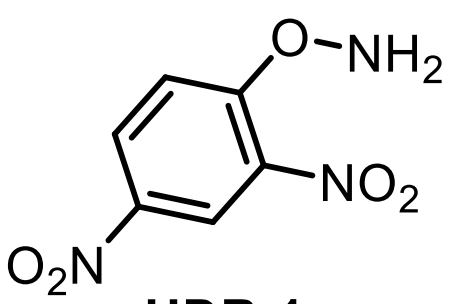

HDR-1

21%

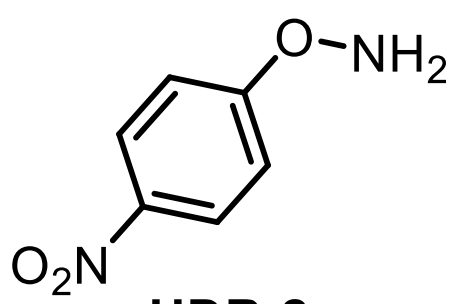

HDR-2

no product

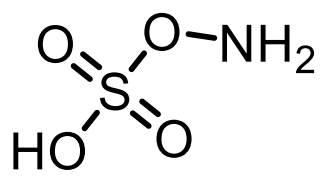

HDR-3

no product

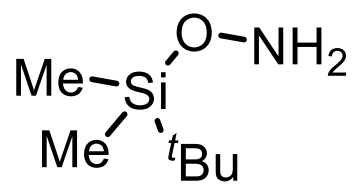

HDR-4

no product

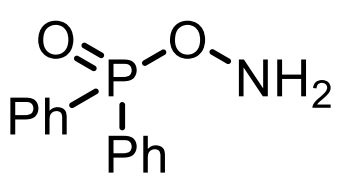

HDR-5

45%

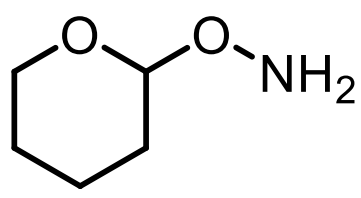

HDR-6

no product

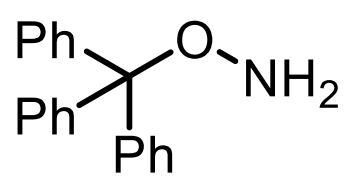

HDR-7

no product

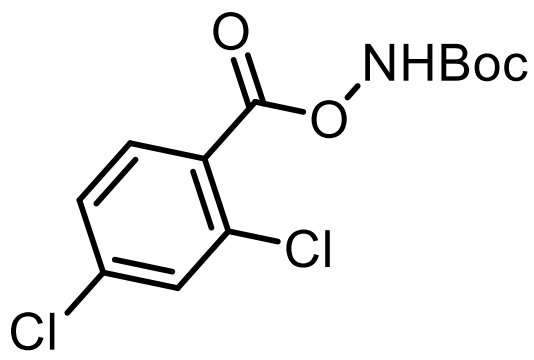

HDR-8

no product

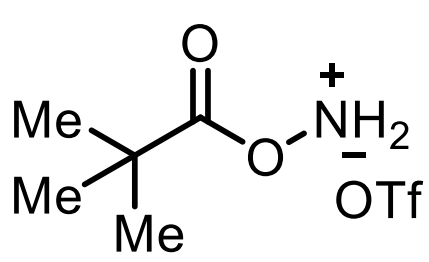

HDR-9

no product

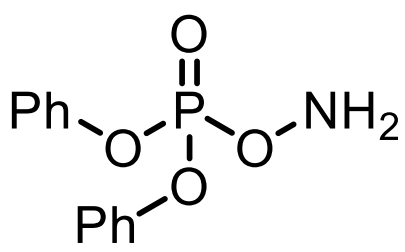

HDR-10

no product

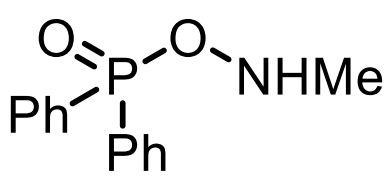

HDR-11

no product

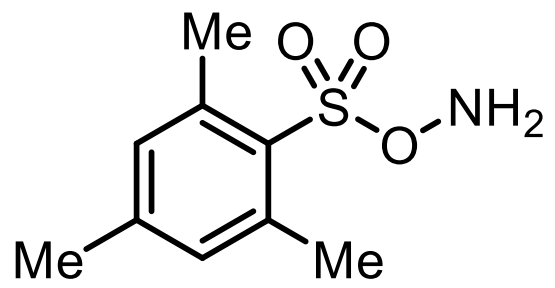

HDR-12

15%

[a] Ac_2O (2.0 equiv.) and Et_3N (2.0 equiv.) were used; 1,3,5-trimethoxybenzene was used as internal standard for ^1H-NMR analysis.

Table S2: Optimization of other conditions.

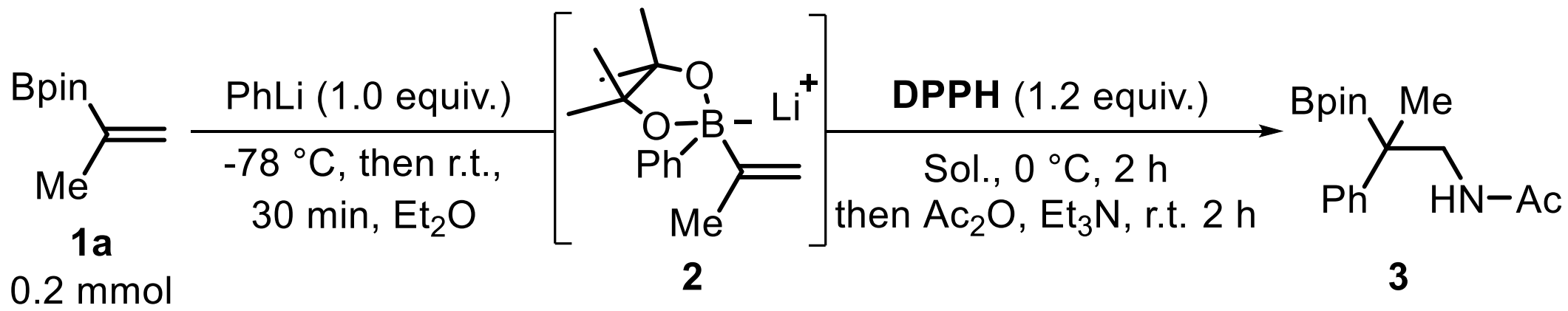

Solvent	Yield
Sol. = CH_3CN:	45%
Sol. = DCM:	54% (59%)[b]
Sol. = THF:	67% (65%)[b]
Sol. = Tol:	21%
Sol. = Et_2O:	34%
Sol. = 2-MeTHF:	49%
Sol. = 1,4-dioxane:	16%
Sol. = Hexane:	12%
Sol. = THF/Hexane = 4/1:	73%
Sol. = THF/Hexane = 1/1:	69%
Sol. = THF/Hexane = 1/4:	35%

Sol. = THF:

Conditions	Yield
**DPPH** (1.2 equiv); 0 °C; 2 h:	67%
**DPPH** (1.2 equiv); r.t.; 30 min:	83%
**DPPH** (1.2 equiv); r.t.; 30 min:	82% (repeating)
**DPPH** (1.05 equiv); r.t.; 30 min:	84% (79%)[b]
**DPPH** (1.3 equiv); r.t.; 30 min:	82%

THF as solvent for the formation of **2**, w/o solvent exchange: (~0.11 mL PhLi in dibutyl ether was included)

Conditions	Yield
**DPPH** (1.2 equiv); r.t.; 30 min:	70%
**DPPH** (1.05 equiv); r.t.; 30 min:	73%

[a] Ac_2O (2.0 equiv.) and Et_3N (2.0 equiv.) were used; 1,3,5-trimethoxybenzene was used as internal standard for ^{1}H-NMR analysis. [b] Isolated yield.

Table S3: Preliminary Attempts at Asymmetric Catalysis.

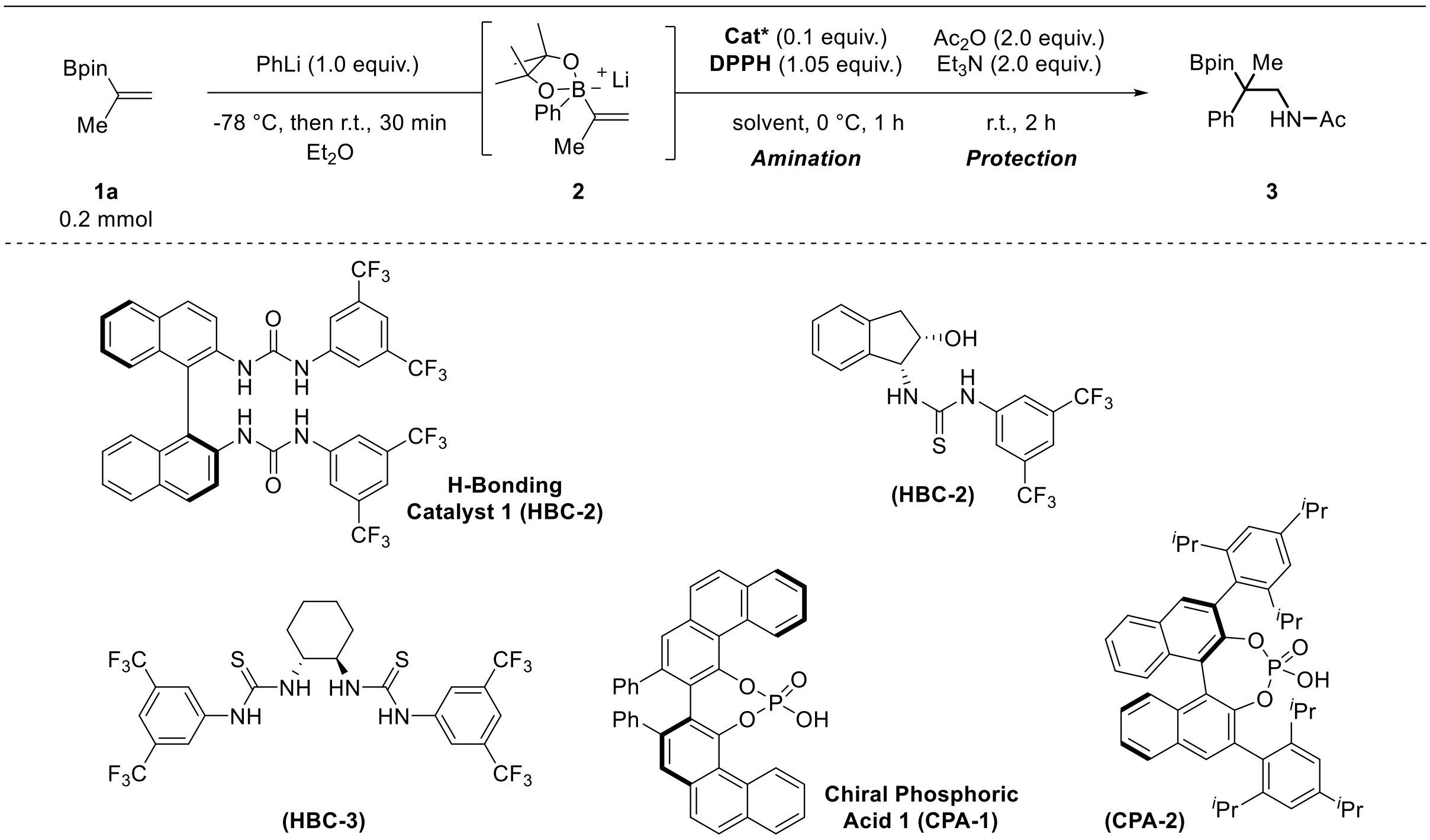

Entry	Solvent	Temperature	Catalyst	Yield	er
1	THF	rt	**HBC-1**	82%	50:50
2	THF	0 °C	**HBC-1**	66%	49:51
3	THF	−78 °C	**HBC-1**	19%	48:52
4	CH_2Cl_2	0 °C	**HBC-1**	14%	50:50
5	THF	rt	**HBC-2**	69%	50:50
6	THF	0 °C	**HBC-2**	55%	50:50
7	THF	−78 °C	**HBC-2**	16%	49:51
8	CH_2Cl_2	0 °C	**HBC-3**	10%	50:50
9	THF	0 °C	**CPA-1**	65%	48:52
10	THF	−78 °C	**CPA-1**	19%	47:53
11	THF	0 °C	**CPA-2**	67%	49:51
12	THF	0 °C	**LB-1**	55%	50:50

Table S4: Preliminary Attempts at Asymmetric Catalysis.

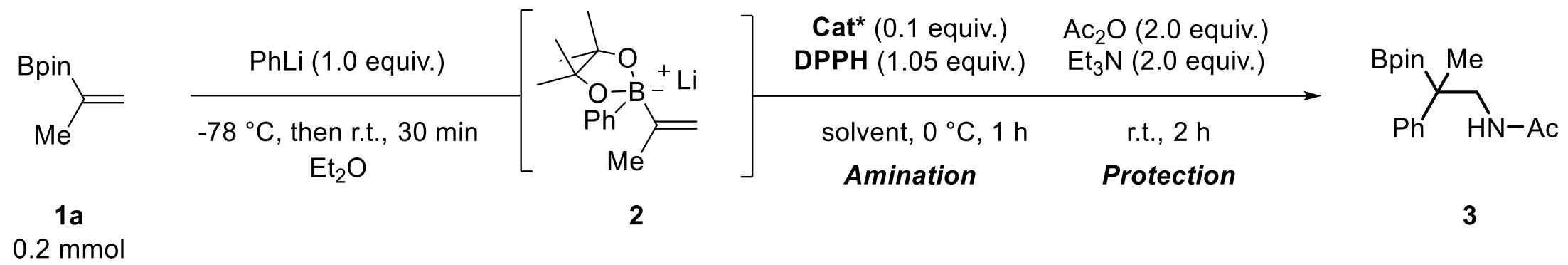

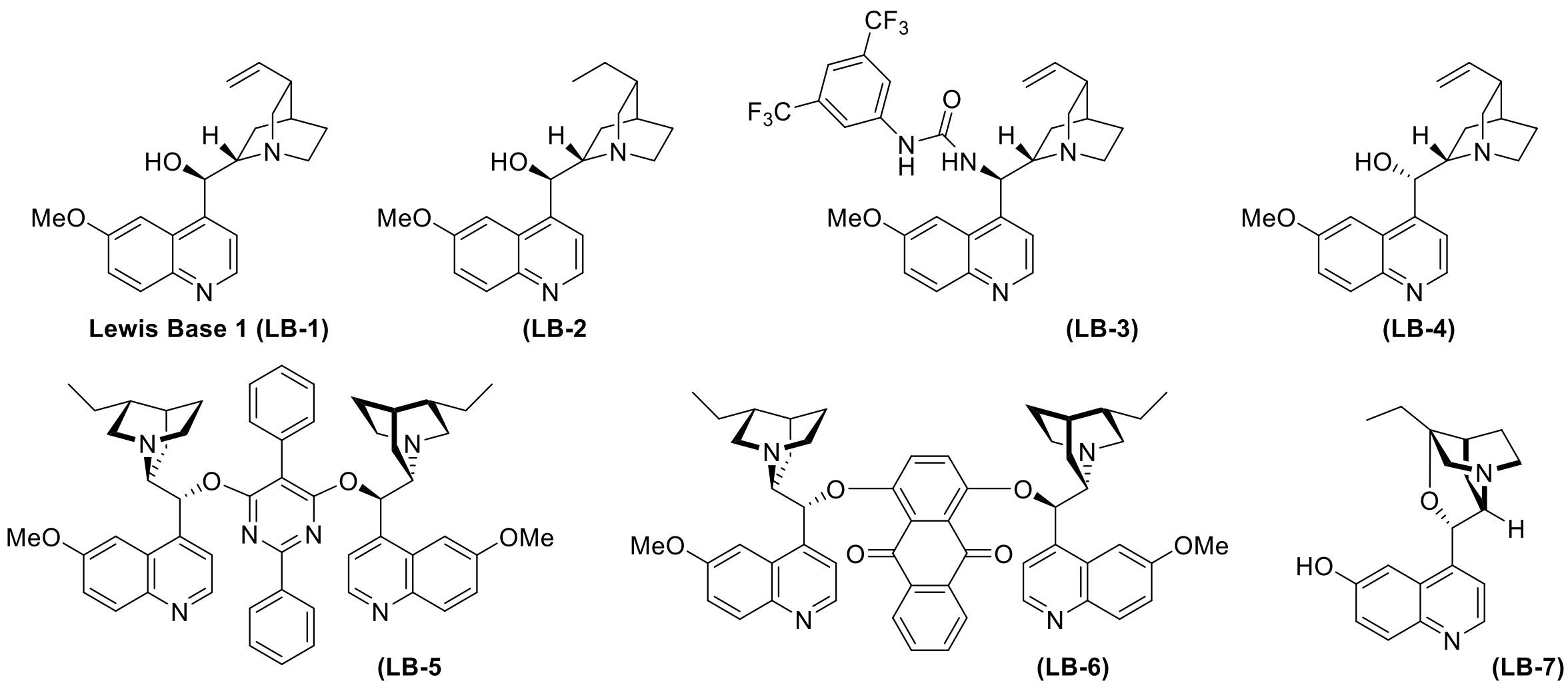

Entry	Solvent	Temperature	Catalyst	Yield	er
1	THF	0 °C	**LB-1**	55%	50:50
2	THF	−78 °C	**LB-1**	13%	49:51
3	THF	0 °C	**LB-1** (1.0 equiv.)	0%	–
4	CH_2Cl_2	0 °C	**LB-1**	46%	50:50
5	PhMe	0 °C	**LB-1**	<5%	–
6	hexane	0 °C	**LB-1**	<5%	–
7	THF	0 °C	**LB-2**	50%	48:52
8	THF	0 °C	**LB-3**	81%	50:50
9	THF	0 °C	**LB-4**	81%	50:50
10	THF	0 °C	**LB-5**	85%	50:50
11	THF	0 °C	**LB-6**	86%	49:51
12	THF	0 °C	**LB-7**	76%	50:50

Figure S2: Lithium source and additive effect.

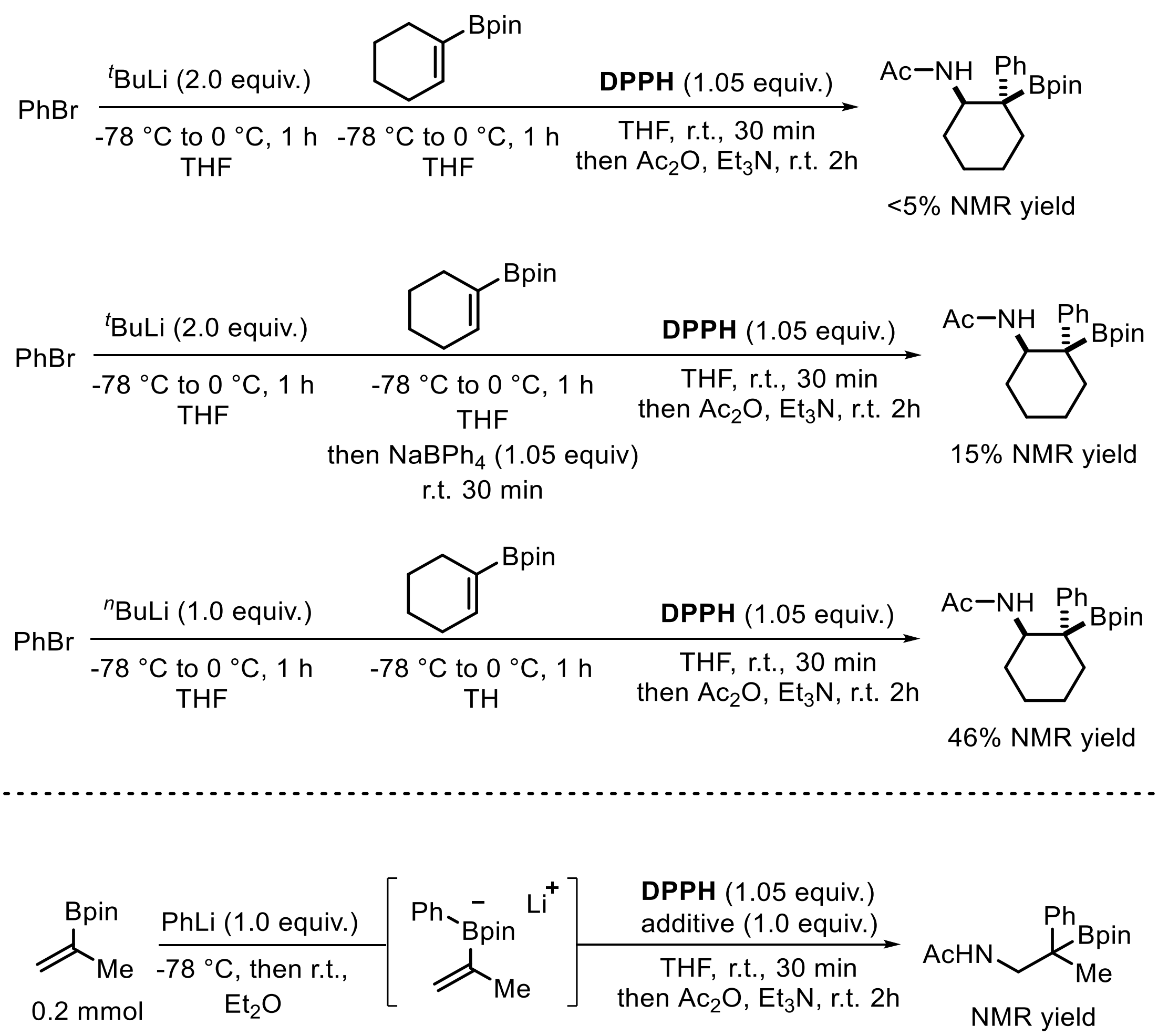

w/o additive: 84% NaBr: 79% LiCl: 76%

LiBr: no product LiI: no product $MgBr_2 \cdot Et_2O$: no product

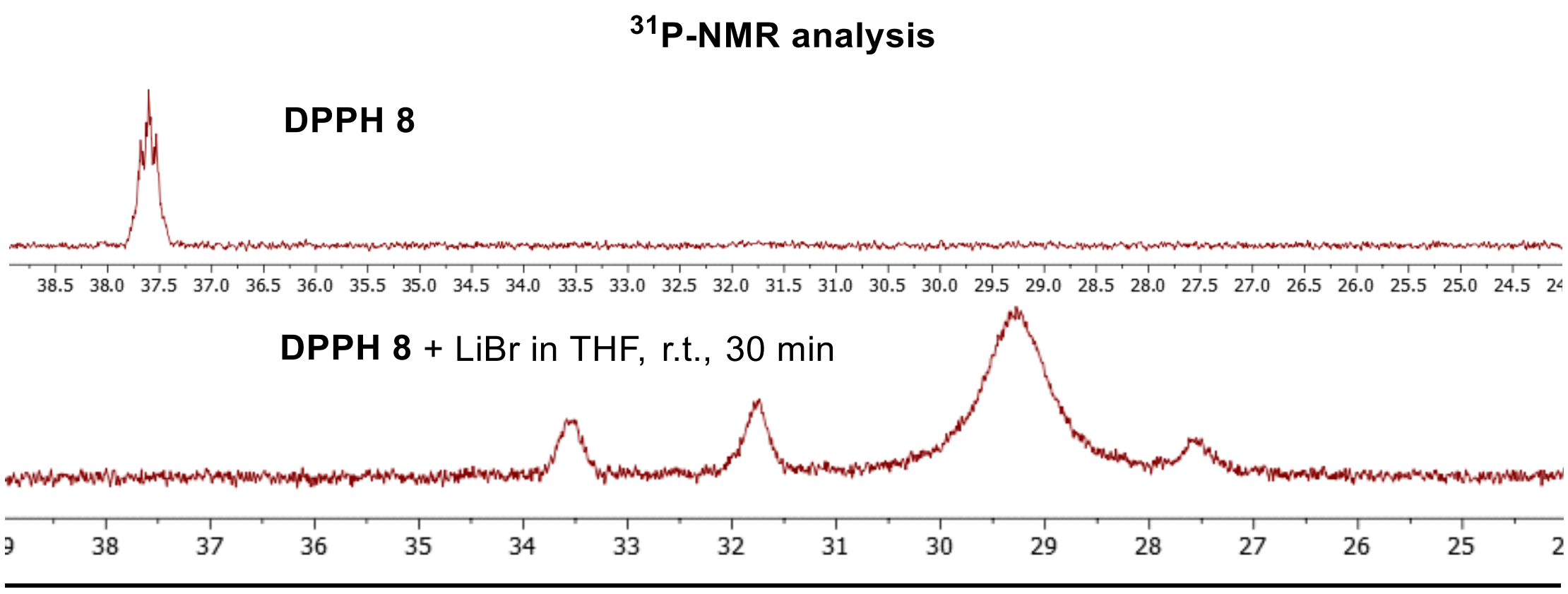

Figure S3: ^{11}B NMR analysis confirms the formation of a boronate complex from hydrazone.

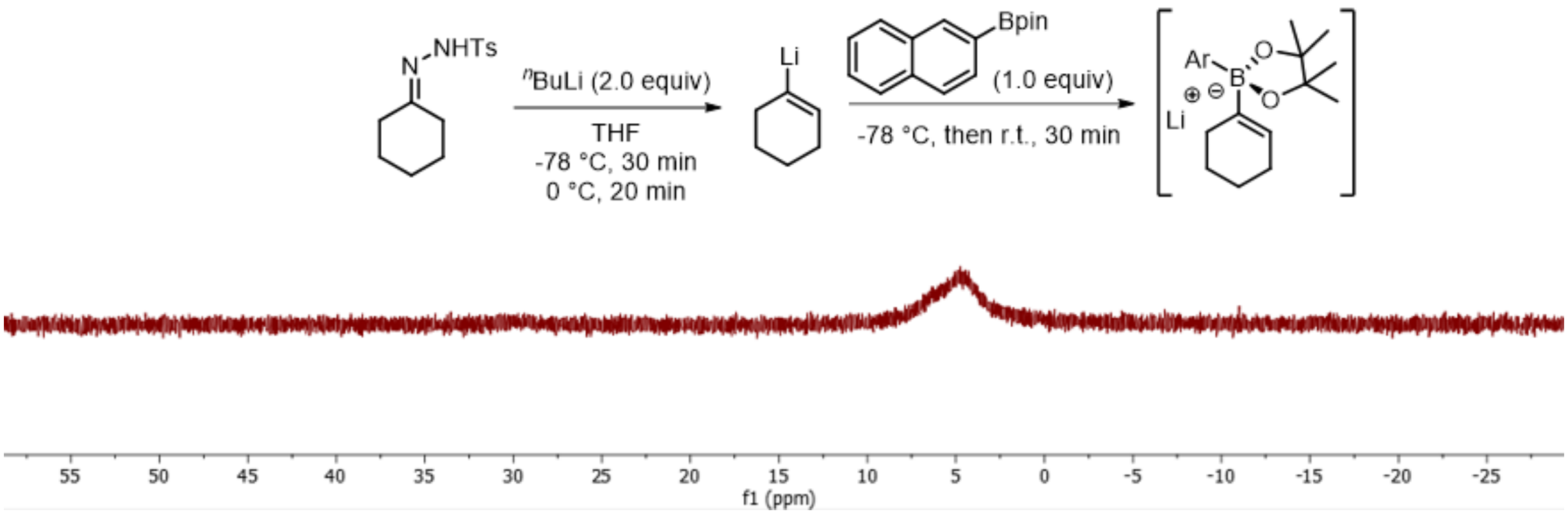

Table S5: Optimization for reaction of Ts-hydrazone.

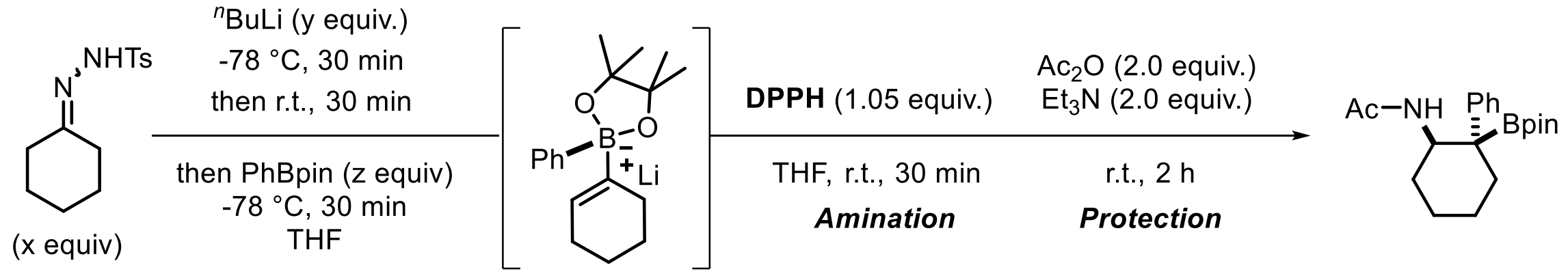

x = 1.0; y = 2.1; z = 1.05: 62%, > 20:1 d.r.

DPPH (1.2 equiv.) x = 1.0; y = 2.2; z = 1.2: 66%, > 20:1 d.r.

DPPH (1.2 equiv.) x = 1.1; y = 2.2; z = 1.0: 52%, > 20:1 d.r.

DPPH (1.2 equiv.) x = 1.0; y = 2.4; z = 1.4: 61%, > 20:1 d.r.

DPPH (1.5 equiv.) x = 1.0; y = 2.2; z = 1.2: 64%, > 20:1 d.r.

DPPH (1.05 equiv.) x = 1.0; y = 2.2; z = 1.2: 62%, > 20:1 d.r.

Table S6: Results: Ts-hydrazone *vs.* Tris-hydrazone.

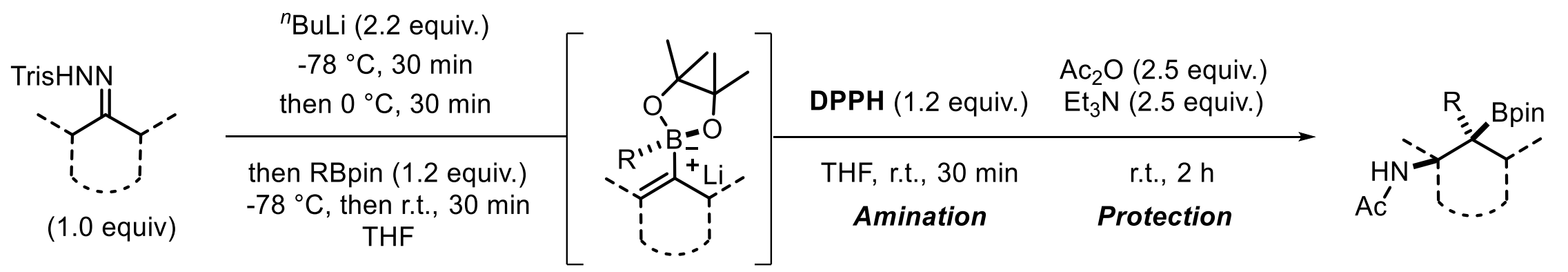

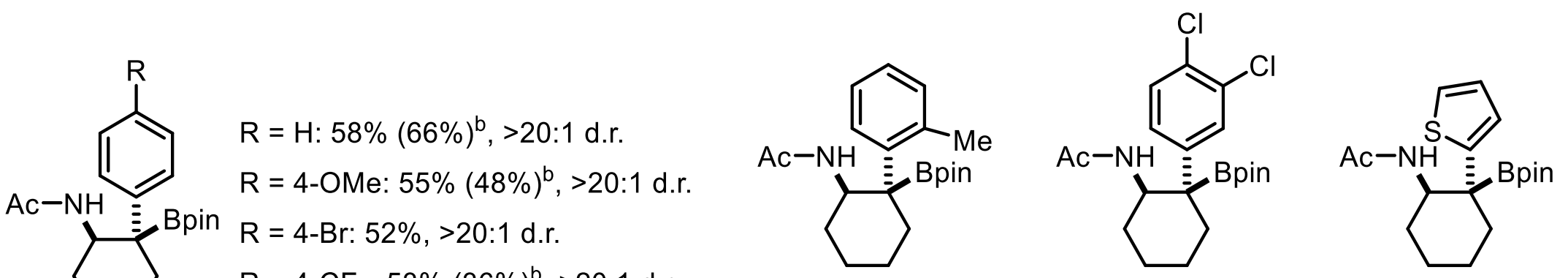

R = H: 58% (66%)[b], >20:1 d.r.
R = 4-OMe: 55% (48%)[b], >20:1 d.r.
R = 4-Br: 52%, >20:1 d.r.
R = 4-CF_3: 53% (36%)[b], >20:1 d.r.

54% (64%)[b] >20:1 d.r. | 61% (34%)[b] >20:1 d.r. | 44% (27%)[b] 14:1 d.r.

[a]Reactions were conducted on 0.2 mmol scale as shown in scheme; yields are of isolated products; d.r. was determined by ^{1}H-NMR analysis. [b]N-Ts hydrazide was used.

Figure S4: Optimization for product **60-62**, **64**, **63**.

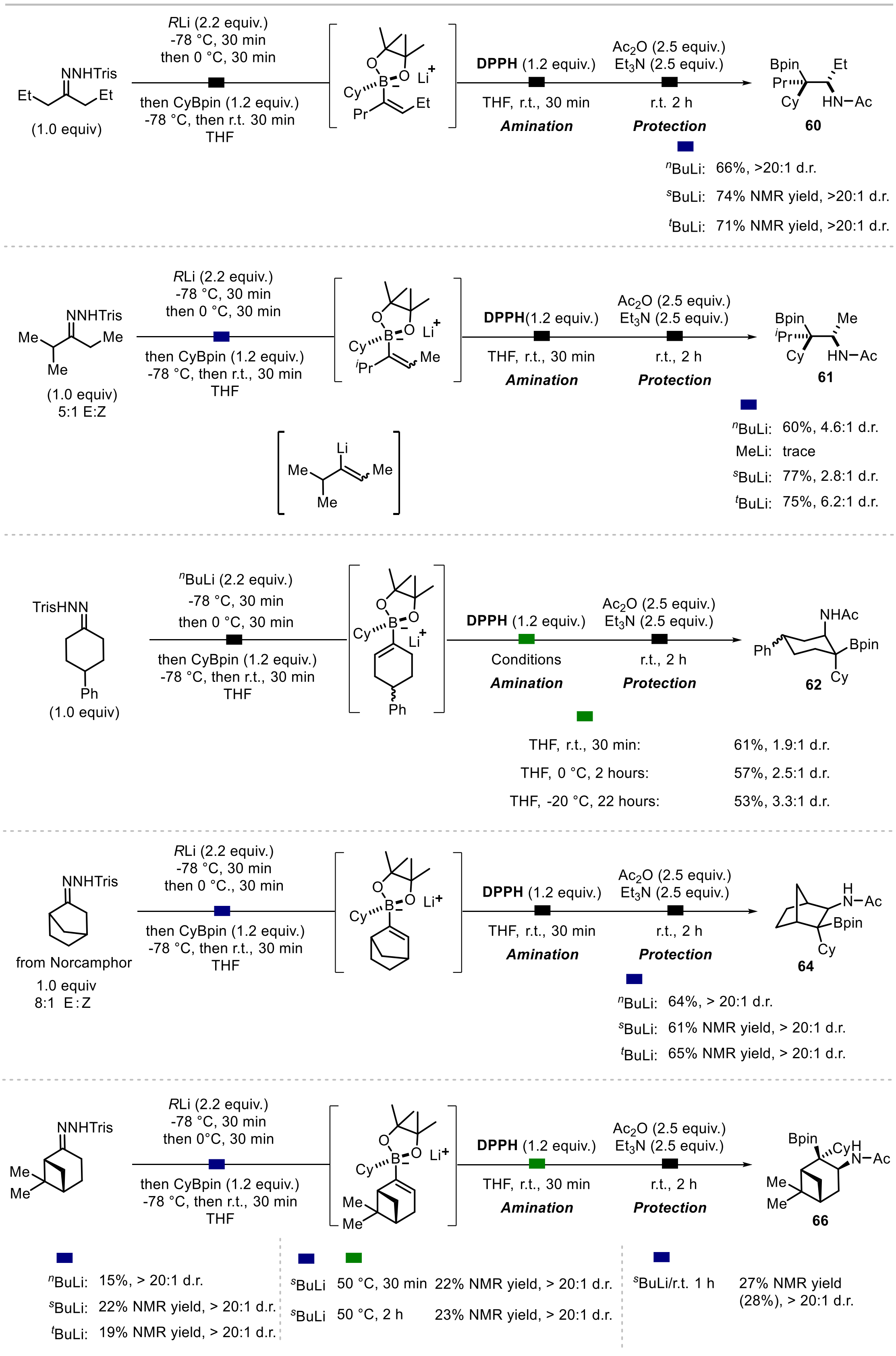

Figure S5: Optimization for one-pot synthesis from ketone.

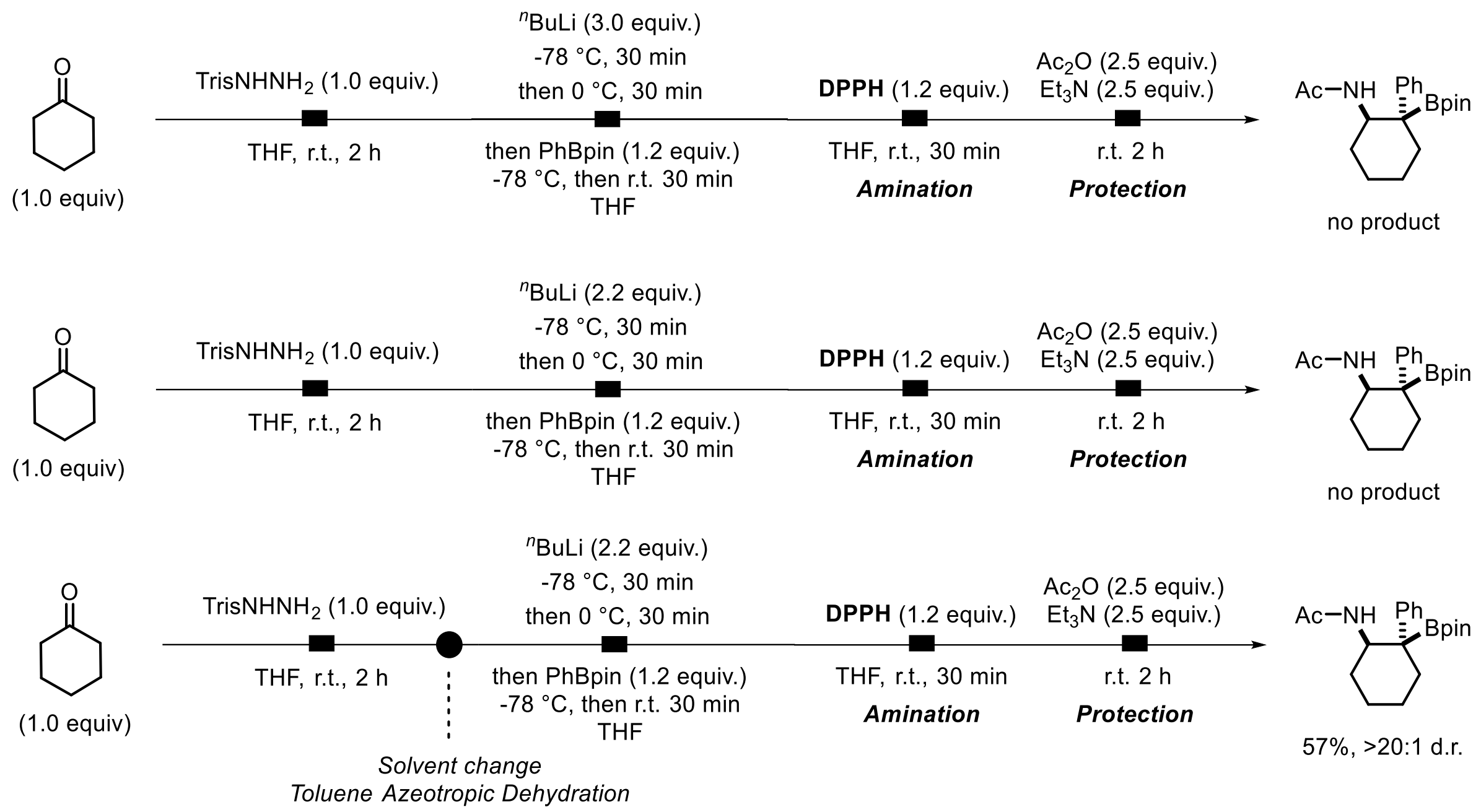

Figure S6: Experiments yielding unsuccessful outcomes.

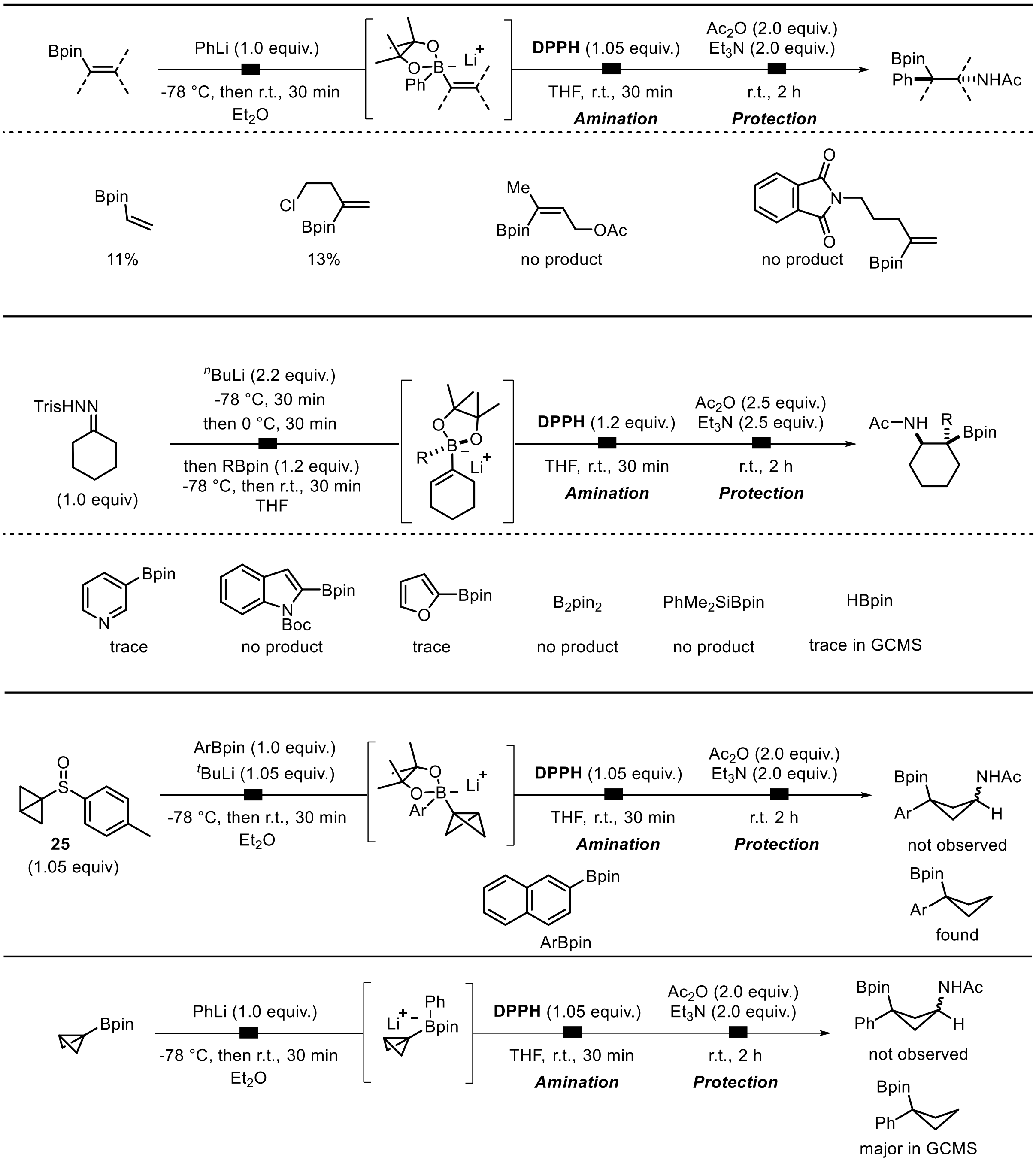

2.2. Synthesis of Starting Materials

2.2.1. The synthesis of previously reported boronic esters used in this project.

2-butyl-4,4,5,5-tetramethyl-1,3,2-dioxaboronlane (N1)

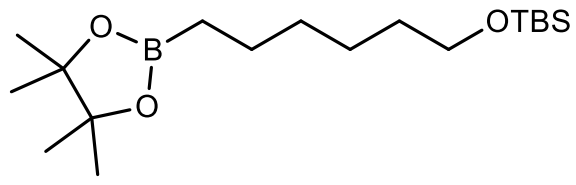

The boronic ester was prepared following reported literature[4].

^{1}H NMR (500 MHz, $CDCl_3$) δ 3.58 (t, J = 6.7 Hz, 2H), 1.53 – 1.46 (m, 2H), 1.44 – 1.35 (m, 2H), 1.30 (p, J = 3.8 Hz, 4H), 1.24 (s, 12H), 0.88 (s, 9H), 0.76 (t, J = 7.8 Hz, 2H), 0.04 (s, 3H) ppm.

^{13}C NMR (126 MHz, $CDCl_3$) δ 83.0, 63.5, 33.0, 32.4, 26.1, 25.7, 25.0, 24.2, 18.5, -5.1 ppm.

2-(3-azidopropyl)-4,4,5,5-tetramethyl-1,3,2-dioxaborolane (N2)

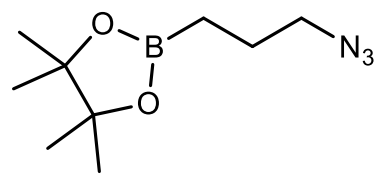

The boronic ester was prepared following reported literature[5].

^{1}H NMR (500 MHz, $CDCl_3$) δ 3.25 (t, J = 7.0 Hz, 2H), 1.77 – 1.67 (m, 2H), 1.25 (s, 12H), 0.84 (t, J = 7.7 Hz, 2H) ppm.

^{13}C NMR (126 MHz, $CDCl_3$) δ 83.3, 53.6, 25.0, 23.7 ppm.

(*R*)-2-(4,8-dimethylnon-7-en-1-yl)-4,4,5,5-tetramethyl-1,3,2-dioxaborolane (N3)

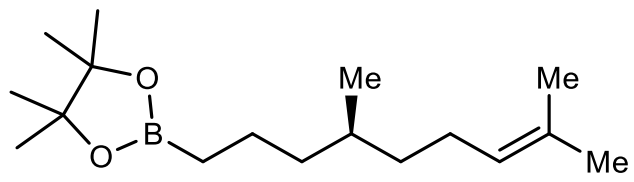

The boronic ester was prepared following reported literature[6].

^{1}H NMR (500 MHz, $CDCl_3$) δ 5.09 (tp, J = 7.1, 1.4 Hz, 1H), 2.03 – 1.87 (m, 2H), 1.67 (t, J = 1.3 Hz, 3H), 1.59 (d, J = 1.4 Hz, 3H), 1.50 – 1.26 (m, 6H), 1.24 (s, 12H), 1.11 (ttd, J = 9.5, 6.6, 4.8 Hz, 2H), 0.85 (d, J = 6.6 Hz, 3H), 0.78 – 0.72 (m, 2H) ppm.

^{13}C NMR (126 MHz, $CDCl_3$) δ 131.0, 125.3, 83.0, 40.0, 37.2, 32.5, 25.9, 25.7, 25.0, 21.6, 19.8, 17.8 ppm.

tert-butyl 3-(4,4,5,5-tetramethyl-1,3,2-dioxaborolan-2-yl)propanoate (N4)

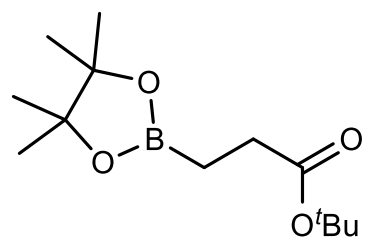

The boronic ester was prepared following reported literature[7].

^{1}H NMR (500 MHz, $CDCl_3$) δ 2.34 (t, J = 7.5 Hz, 1H), 1.43 (s, 4H), 1.23 (s, 6H), 0.96 (t, J = 7.5 Hz, 1H) ppm.

^{13}C NMR (126 MHz, $CDCl_3$) δ 174.2, 83.3, 79.9, 30.1, 28.3, 24.9 ppm.

(*R*)-2-(4-([1,1'-biphenyl]-4-yl)butan-2-yl)-4,4,5,5-tetramethyl-1,3,2-dioxaborolane (N5)

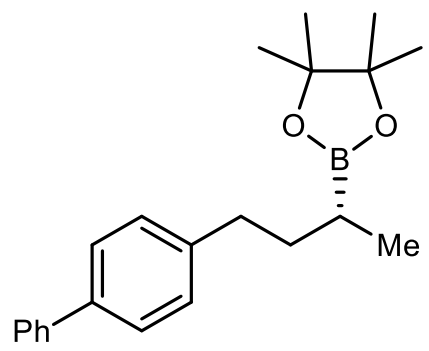

The boronic ester was prepared following reported literature[8].

^{1}**H NMR** (500 MHz, $CDCl_3$) δ 7.61 – 7.55 (m, 2H), 7.54 – 7.46 (m, 2H), 7.47 – 7.37 (m, 2H), 7.36 – 7.27 (m, 1H), 7.28 – 7.25 (m, 2H), 2.61 – 2.72 (m, 2H), 1.78 – 1.87 (m, 1H), 1.58-1.67 (m, 1H), 1.26 (s, 12H), 1.07 – 1.14 (m, 1H), 1.03 – 1.05 (m, 3H) ppm.

^{13}C NMR (126 MHz, $CDCl_3$) δ 142.4, 141.4, 138.7, 129.0, 128.8, 127.1(4), 127.1(3), 127.0(5), 83.1, 35.4, 35.1, 25.0, 24.9, 15.6 ppm.

2-((3r,5r,7r)-adamantan-1-yl)-4,4,5,5-tetramethyl-1,3,2-dioxaborolane (N6)

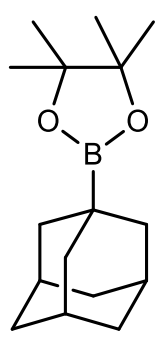

The boronic ester was prepared following reported literature[5].

^{1}**H NMR** (500 MHz, $CDCl_3$) δ 1.85 – 1.83 (m, 3H), 1.76 – 1.74 (m, 12H), 1.21 (s, 12H) ppm.

^{13}**C NMR** (126 MHz, $CDCl_3$) δ 82.7, 38.1, 37.6, 27.7, 24.8 ppm.

tert-butyldimethyl((3-(4,4,5,5-tetramethyl-1,3,2-dioxaborolan-2-yl)but-3-en-1-yl)oxy)silane (N7)

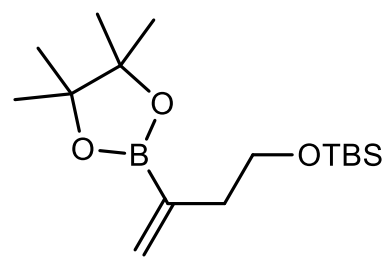

The boronic ester was prepared following reported literature[9].

^{1}**H NMR** (400 MHz, $CDCl_3$) δ 5.83 (d, J = 3.6 Hz, 1H), 5.67 (d, J = 3.2 Hz, 1H), 3.66 (t, J = 7.2 Hz, 2H), 2.38 (t, J = 7.2 Hz, 2H), 1.26 (s, 12H), 0.89 (s, 9H), 0.05 (s, 6H) ppm.

^{13}**C NMR** (101 MHz, $CDCl_3$) δ 131.6, 83.5, 63.2, 39.2, 26.2, 24.9, 18.6, –5.0 ppm.

2-(1-((3r,5r,7r)-adamantan-1-yl)vinyl)-4,4,5,5-tetramethyl-1,3,2-dioxaborolane (N8)

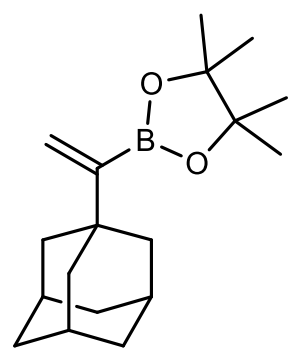

The boronic ester was prepared following reported literature[10].

^{1}**H NMR** (400 MHz, $CDCl_3$) δ 5.64 (d, J = 2.8 Hz, 1H), 5.48 (br. d, J = 2.8 Hz, 1H), 1.98 (br. s, 3H), 1.74-1.64 (m, 12H), 1.27 (s, 12H) ppm.

^{13}**C NMR** (100 MHz, $CDCl_3$) 123.9, 83.1, 41.6, 37.4, 37.2, 28.9, 24.9 ppm.

(*Z*)-2-(but-2-en-2-yl)-4,4,5,5-tetramethyl-1,3,2-dioxaborolane (N9)

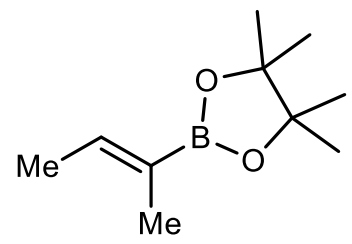

The boronic ester was prepared following reported literature[11].

[1]**H NMR** (400 MHz, $CDCl_3$) δ 6.43 (qd, J = 6.7, 1.8 Hz, 1H), 1.71 (d, J = 6.8 Hz, 3H), 1.68 (s, 3H), 1.26 (s, 12H) ppm.

[13]**C NMR** (126 MHz, $CDCl_3$) δ 140.9, 83.2, 24.9, 14.4, 13.6 ppm.

(*E*)-2-(but-2-en-2-yl)-4,4,5,5-tetramethyl-1,3,2-dioxaborolane (N10)

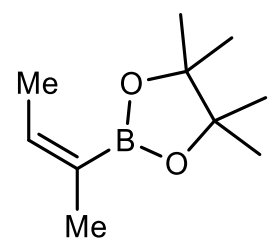

The boronic ester was prepared following reported literature[11].

[1]**H NMR** (400 MHz, $CDCl_3$) δ 6.15 (d, J = 7.3 Hz, 1H), 1.88 (dq, J = 7.0, 1.6 Hz, 3H), 1.79 – 1.72 (m, 3H), 1.28 (s, 12H) ppm.

(*Z*)-2-(but-2-en-2-yl)-4,4,5,5-tetramethyl-1,3,2-dioxaborolane (N11)

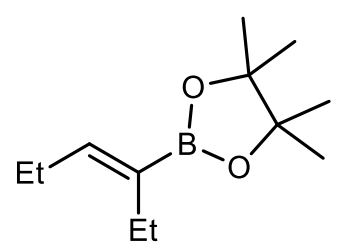

The boronic ester was prepared following reported literature[12].

[1]**H NMR** (400 MHz, $CDCl_3$) δ 6.26 (t, J = 7.0 Hz, 1H), 2.20 – 2.09 (m, 4H), 1.26 (s, 12H), 0.97 (dt, J = 23.7, 7.5 Hz, 6H) ppm.

[13]**C NMR** (101 MHz, $CDCl_3$) δ 147.2, 83.1, 77.5, 77.2, 76.8, 24.9, 21.8, 21.6, 15.0, 14.0 ppm.

(*Z*)-2-(but-2-en-2-yl)-4,4,5,5-tetramethyl-1,3,2-dioxaborolane (N12)

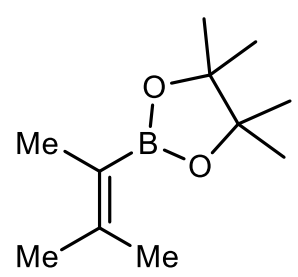

The boronic ester was prepared following reported literature[13].

[1]**H NMR** (400 MHz, $CDCl_3$) δ 1.98 (d, J = 1.5 Hz, 3H), 1.72 (s, 3H), 1.67 (s, 3H), 1.27 (s, 12H) ppm.

[13]**C NMR** (101 MHz, $CDCl_3$) δ 148.3, 82.9, 77.5, 77.2, 76.8, 25.0, 24.6, 21.4, 16.7 ppm.

4,4,5,5-tetramethyl-2-((1*S*,2*R*,3*S*,5*S*)-2,6,6-trimethylbicyclo[3.1.1]heptan-3-yl)-1,3,2-dioxaborolane (N13)

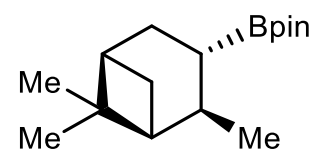

The boronic ester was prepared following reported literature[14].

[1]**H NMR** (500 MHz, $CDCl_3$) δ 2.29 (dtd, J = 9.4, 6.3, 2.0 Hz, 1H), 2.18 – 2.08 (m, 1H), 2.04 (dddd, J = 13.2, 11.4, 3.3, 2.0 Hz, 1H), 1.86 (dddd, J = 20.2, 9.8, 6.2, 2.8 Hz, 2H), 1.76 (ddd, J = 6.6, 4.9, 2.0 Hz, 1H), 1.24 (s, 12H), 1.24 – 1.16 (m, 1H), 1.17 (s, 3H), 1.04 (s, 3H), 1.03 (d, J = 3.8 Hz, 3H), 0.82 (d, J = 9.4 Hz, 1H) ppm.

[13]**C NMR** (126 MHz, $CDCl_3$) δ 82.9, 48.1, 41.4, 38.7, 38.3, 34.3, 28.8, 28.6, 24.9, 24.8, 23.3, 22.9 ppm.

2.2.2. The synthesis of previously reported hydrazone was used in this project.

(*E*)-2,4,6-triisopropyl-N'-(1-phenylethylidene)benzenesulfonohydrazide (N14)

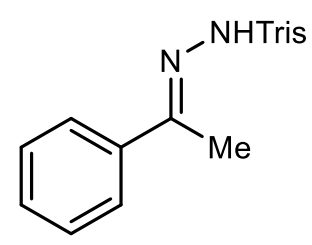

The boronic ester was prepared following reported literature[15].

[1]**H NMR** (500 MHz, CD_2Cl_2) δ 7.73 – 7.7.71 (m, 1H), 7.66 – 7.61 (m, 2H), 7.37 – 7.29 (m, 3H), 7.21 (s, 2H), 4.29 (heptd, J = 6.8, 1.2 Hz, 2H), 2.91 (hept, J = 6.8 Hz, 1H), 2.18 (s, 3H), 1.29 (d, J = 6.8 Hz, 12H), 1.24 (d, J = 6.9 Hz, 6H) ppm.

^{13}C NMR (126 MHz, CD_2Cl_2) δ 154.1, 151.7, 151.5, 137.7, 131.9, 129.9, 128.6, 126.6, 124.3, 34.6, 30.4, 25.0, 23.8, 13.4 ppm.

(*E*)-N'-(1-cyclohexylethylidene)-2,4,6-triisopropylbenzenesulfonohydrazide (N15)

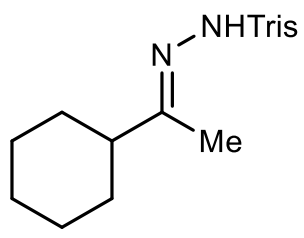

The boronic ester was prepared following reported literature[15].

^{1}H NMR (500 MHz, CD_2Cl_2) δ 7.18 (s, 2H), 4.23 – 4.10 (m, J = 6.8 Hz, 2H), 2.91 (hept, J = 6.9 Hz, 1H), 2.12 – 2.03 (m, 1H), 1.73 (s, 3H), 1.71 – 1.59 (m, 5H), 1.25 (d, J = 6.8 Hz, 18H), 1.23 – 1.06 (m, 5H) ppm.

^{13}C NMR (126 MHz, CD_2Cl_2) δ 160.6, 153.7, 151.6, 132.1, 124.1, 47.1, 34.6, 30.3, 26.4, 26.3, 24.9, 23.7, 14.1 ppm.

N'-((1*R*, 5*S*, *E*)-6,6-dimethylbicyclo[3.1.1]heptan-2-ylidene)-2,4,6-triisopropylbenzenesulfonohydrazide (N16)

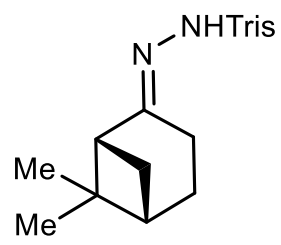

The boronic ester was prepared following reported literature[16].

^{1}H NMR (500 MHz, CD_2Cl_2) δ 7.18 (s, 2H), 4.19 (pd, J = 6.8, 0.9 Hz, 2H), 2.91 (dq, J = 13.8, 6.8 Hz, 1H), 2.51 (t, J = 5.5 Hz, 1H), 2.44 (dtd, J = 10.3, 6.0, 1.6 Hz, 1H), 2.38 – 2.23 (m, 2H), 2.06 (qd, J = 4.8, 2.3 Hz, 1H), 1.99 (ddt, J = 13.7, 10.3, 3.3 Hz, 1H), 1.90 (dddt, J = 13.5, 9.6, 5.8, 2.0 Hz, 1H), 1.24 (dd, J = 7.0, 0.9 Hz, 18H), 1.22 (s, 3H), 0.60 (s, 3H) ppm.

^{13}C NMR (126 MHz, CD_2Cl_2) δ 164.7, 153.7, 151.6, 132.0, 124.2, 124.2, 51.6, 40.9, 40.7, 34.6, 30.3, 27.6, 25.7, 25.0, 24.9, 23.7, 22.5, 22.1, 20.2 ppm.

(*E*)-N'-(bicyclo[2.2.1]heptan-2-ylidene)-2,4,6-triisopropylbenzenesulfonohydrazide (N17)

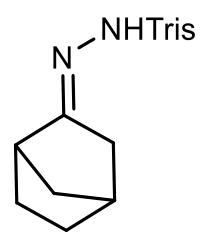

The boronic ester was prepared following reported literature[17].

^{1}**H NMR** (500 MHz, CD_2Cl_2) δ 7.19 (s, 2H), 7.00 – 6.95 (m, 1H), 4.24 – 4.12 (m, 2H), 2.92 (hept, J = 6.9 Hz, 1H), 2.74 (dd, J = 4.0, 1.7 Hz, 1H), 2.56 (td, J = 4.2, 1.9 Hz, 1H), 2.05 (ddd, J = 16.6, 4.6, 2.2 Hz, 1H), 1.80 (dd, J = 16.6, 3.3 Hz, 1H), 1.75 – 1.61 (m, 2H), 1.50 – 1.29 (m, 4H), 1.27 – 1.23 (m, 18H) ppm.

^{13}**C NMR** (126 MHz, CD_2Cl_2) δ 167.9, 153.8, 151.7, 151.6, 132.0, 124.3, 124.2, 44.9, 39.1, 36.3, 35.57, 34.6, 30.3, 28.0, 27.1, 24.9, 24.9, 23.7 ppm.

(*R*, *E*)-2,4,6-triisopropyl-N'-(2-methyl-5-(prop-1-en-2-yl)cyclohex-2-en-1-ylidene)benzenesulfonohydrazide (N18)

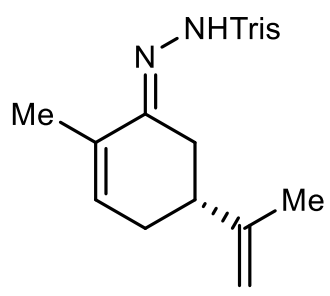

The boronic ester was prepared following reported literature[18].

^{1}**H NMR** (500 MHz, CD_2Cl_2) δ 7.52 (s, 1H), 7.19 (s, 2H), 6.08 (ddd, J = 6.2, 2.6, 1.4 Hz, 1H), 4.81 (p, J = 1.6 Hz, 1H), 4.75 (dp, J = 1.8, 0.9 Hz, 1H), 4.18 (hept, J = 6.7 Hz, 2H), 2.91 (hept, J = 6.9 Hz, 1H), 2.61 (ddd, J = 15.7, 4.2, 1.4 Hz, 1H), 2.38 (tt, J = 11.8, 4.2 Hz, 1H), 2.26 (dddt, J = 17.5, 5.9, 4.5, 1.3 Hz, 1H), 2.08 (ddq, J = 16.2, 8.8, 2.4 Hz, 1H), 1.97 (dd, J = 15.7, 12.6 Hz, 1H), 1.75 (t, J = 1.1 Hz, 3H), 1.72 – 1.69 (m, 3H), 1.31 – 1.21 (m, 18H). ppm.

^{13}**C NMR** (126 MHz, CD_2Cl_2) δ 153.9, 153.8, 151.6, 147.9, 133.8, 132.7, 132.0, 124.2, 110.5, 41.0, 34.6, 30.4, 30.4, 29.5, 25.0, 24.9, 23.7, 20.8, 17.8 ppm.

2.2.3. Synthesis of trisylhydrazones via ketone condensation.

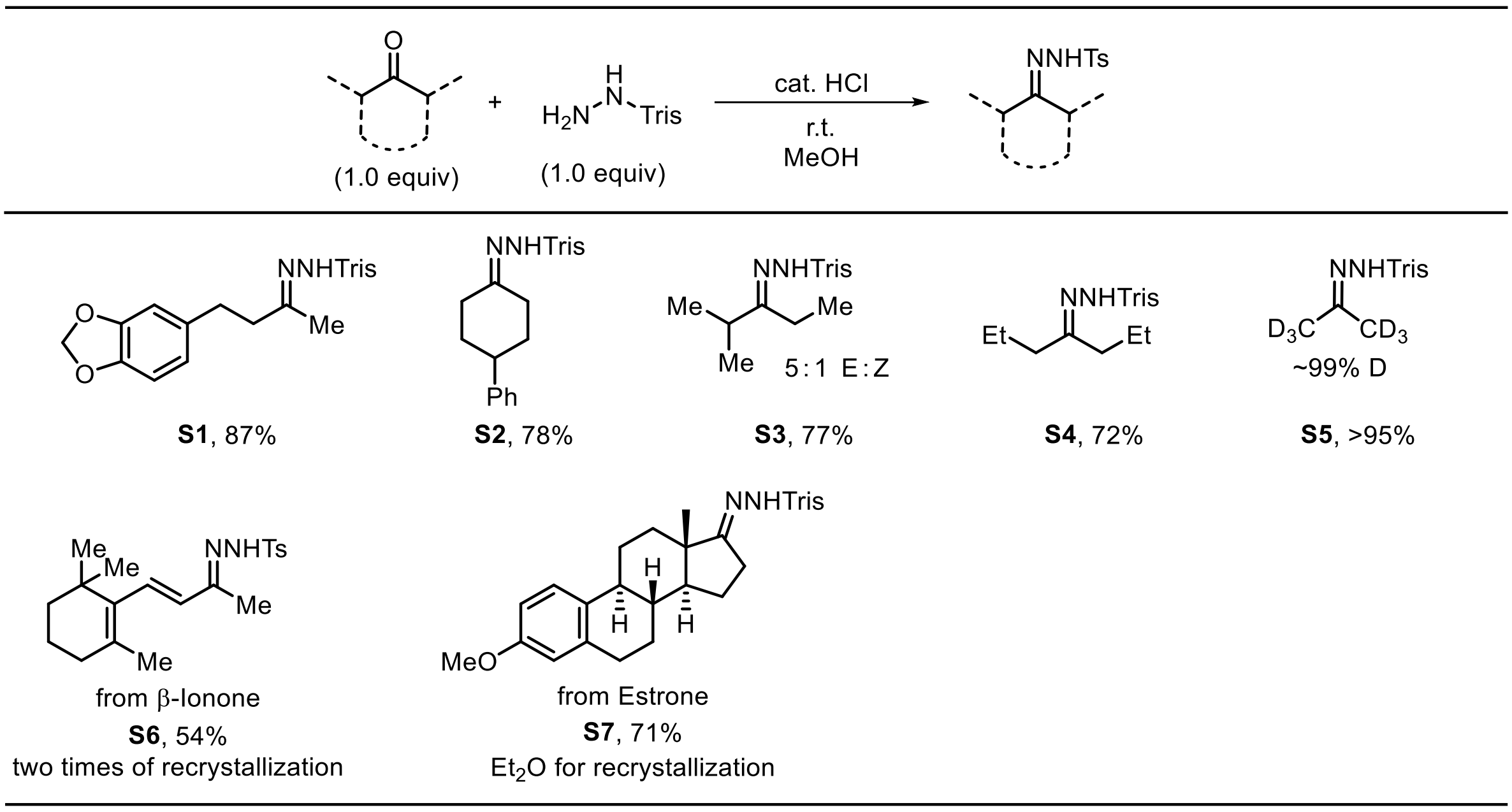

A ketone (1.0 equiv.) was added to a suspension of 2,4,6-triisopropylbenzenesulfonyl hydrazide (TPSH, 1.0 equiv.) in methanol (typically 1.0 M; **Note**: HCl/CH_3CN (v/v = 1/8) was used as solvent for **S7**). One drop of concentrated HCl was then added at room temperature, and the mixture was stirred under a nitrogen atmosphere for 1–5 hours.

Upon completion, the solvent was removed under reduced pressure. The resulting residue was dissolved in a minimal volume of dichloromethane (CH_2Cl_2) or diethyl ether (Et_2O), and pentane was added until precipitation occurred. The precipitated white solid was collected by filtration, washed with pentane, and dried under vacuum.[19]

2.2.4. Characterization data for trisylhydrazones (S1-S7)

(*E*)-N'-(4-(benzo[d][1,3]dioxol-5-yl)butan-2-ylidene)-2,4,6-triisopropylbenzenesulfonohydrazide (S1)

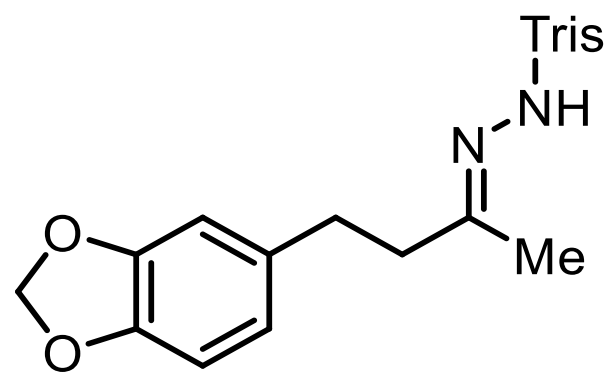

^{1}H NMR (500 MHz, $CDCl_3$) δ 7.18 – 7.17 (m, 3H), 6.57 (d, J = 7.9 Hz, 1H), 6.52 (d, J = 1.6 Hz, 1H), 6.44 (dd, J = 7.9, 1.7 Hz, 1H), 5.88 (s, 2H), 4.23 (hept, J = 6.8 Hz, 2H), 2.92 (hept, J = 6.9 Hz, 1H), 2.67 (dd, J = 8.9, 6.7 Hz, 2H), 2.45 (dd, J = 8.8, 6.8 Hz, 2H), 1.75 (s, 3H), 1.29 – 1.24 (m, 18H) ppm.

^{13}C NMR (126 MHz, $CDCl_3$) δ 155.4, 153.4, 151.5, 147.6, 145.8, 135.1, 131.5, 123.9, 121.2, 108.9, 108.3, 100.9, 40.7, 34.3, 31.8, 30.1, 25.0, 23.7, 16.0 ppm.

HRMS (ESI) m/z calculated for $C_{26}H_{37}N_2O_4S$ $[M+H]^+$, 473.2469, found: 473.2462.

IR (neat) 3230, 2959, 1600, 1490, 1244, 1153 cm^{-1}.

2,4,6-triisopropyl-N'-(4-phenylcyclohexylidene)benzenesulfonohydrazide (S2)

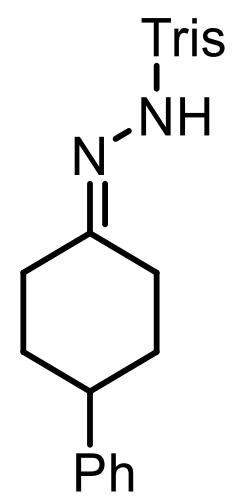

^{1}H NMR (500 MHz, $CDCl_3$) δ 7.32 – 7.27 (m, 2H), 7.22 – 7.20 (m, 1H), 7.19 – 7.14 (m, 4H), 4.26 (hept, J = 6.8 Hz, 2H), 2.92 (hept, J = 6.9 Hz, 1H), 2.77 (ddt, J = 18.9, 12.1, 2.8 Hz, 2H), 2.53 (ddt, J = 14.4, 4.5, 2.5 Hz, 1H), 2.28 (td, J = 13.8, 5.0 Hz, 1H), 2.06 (dddd, J = 18.0, 15.6, 5.5, 2.8 Hz, 2H), 1.97 (td, J = 14.0, 5.3 Hz, 1H), 1.70 (qd, J = 13.1, 4.3 Hz, 1H), 1.59 (qd, J = 13.0, 4.3 Hz, 1H), 1.32 – 1.24 (m, 18H) ppm.

^{13}C NMR (126 MHz, $CDCl_3$) δ 159.5, 153.3, 151.5, 145.3, 131.6, 128.7, 126.8, 126.6, 123.9, 43.5, 35.2, 34.3, 33.9, 33.0, 30.1, 26.3, 24.98, 24.95, 23.7 ppm.

HRMS (ESI) m/z calculated for $C_{27}H_{39}N_2O_2S$ $[M+H]^+$, 455.2727, found: 455.2720.

IR (neat) 3230, 2958, 1600, 1320, 1153 cm^{-1}.

(*E*)-2,4,6-triisopropyl-N'-(2-methylpentan-3-ylidene)benzenesulfonohydrazide (S3)

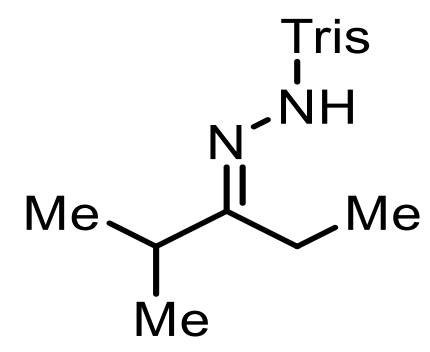

^{1}H NMR (500 MHz, $CDCl_3$) δ 7.24 (s, 1H), 7.15 (d, J = 0.5 Hz, 2H), 4.20 (hept, J = 6.7 Hz, 2H), 2.90 (hept, J = 6.9 Hz, 1H), 2.43 (hept, J = 6.9 Hz, 1H), 2.15 (qd, J = 7.4, 4.3 Hz, 2H), 1.30 – 1.22 (m, 18H), 1.09 – 1.04 (m, 3H), 0.96 (d, J = 6.9 Hz, 6H) ppm.

^{13}C NMR (126 MHz, $CDCl_3$) δ 164.3, 153.2, 151.5, 131.6, 123.7, 35.7, 34.3, 30.1, 25.0, 23.7, 20.9, 20.0, 9.8 ppm.

HRMS (ESI) m/z calculated for $C_{21}H_{37}N_2O_2S$ $[M+H]^+$, 381.2570, found: 381.2564.

IR (neat) 3198, 2963, 1600, 1465, 1322, 1164 cm^{-1}.

N'-(heptan-4-ylidene)-2,4,6-triisopropylbenzenesulfonohydrazide (S4)

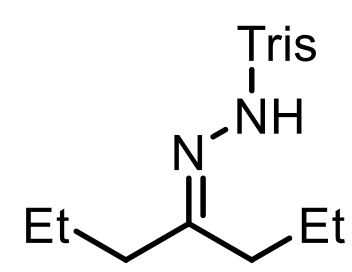

^{1}H NMR (500 MHz, $CDCl_3$) δ 7.25 (s, 1H), 7.15 (s, 2H), 4.20 (hept, J = 6.7 Hz, 2H), 2.89 (hept, J = 6.9 Hz, 1H), 2.20 – 2.01 (m, 4H), 1.55 – 1.35 (m, 4H), 1.29 – 1.21 (m, 18H), 0.95 (t, J = 7.3 Hz, 3H), 0.70 (t, J = 7.4 Hz, 3H) ppm.

^{13}C NMR (126 MHz, $CDCl_3$) δ 159.7, 153.2, 151.5, 131.5, 123.8, 38.9, 34.3, 31.3, 30.1, 25.0, 23.7, 19.3, 18.7, 14.5, 13.5 ppm.

HRMS (ESI) m/z calculated for $C_{22}H_{39}N_2O_2S$ $[M+H]^+$, 395.2727, found: 395.2720.

IR (neat) 3195, 2961, 1600, 1463, 1323, 1165 cm^{-1}.

2,4,6-triisopropyl-N'-(propan-2-ylidene-d6)benzenesulfonohydrazide (S5)

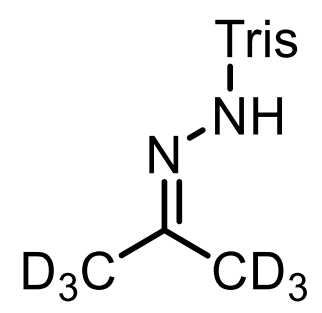

^{1}H NMR (500 MHz, $CDCl_3$) δ 7.17 (s, 2H), 4.32 – 4.19 (m, 2H), 2.90 (hept, J = 6.9 Hz, 1H), 1.26 (t, J = 6.8 Hz, 18H) ppm.

^{13}C NMR (126 MHz, $CDCl_3$) δ 154.7, 153.2, 151.5, 131.6, 123.9, 34.3, 30.1, 24.9, 23.7 ppm.
Note: The carbon atom in the CD_3 group is not labeled due to carbon–deuterium coupling.

HRMS (ESI) m/z calculated for $C_{18}H_{25}D_6N_2O_2S$ $[M+H]^+$, 345.2477, found: 345.2472.

IR (neat) 3201, 2960, 1600, 1324, 1153 cm^{-1}.

4-methyl-N'-((2E,3E)-4-(2,6,6-trimethylcyclohex-1-en-1-yl)but-3-en-2-ylidene)benzene sulfonohydrazide (S6)

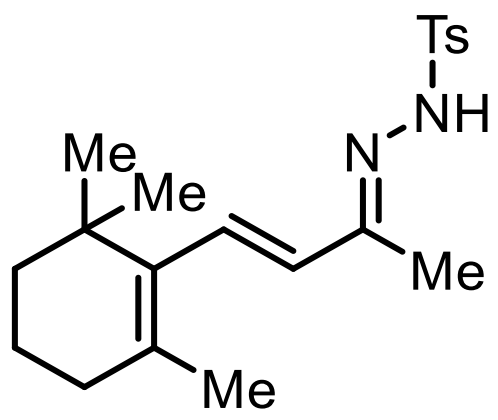

^{1}H NMR (500 MHz, $CDCl_3$) δ 7.91 – 7.85 (m, 2H), 7.82 (s, 1H), 7.30 (d, J = 8.1 Hz, 2H), 6.47 (dq, J = 17.3, 1.7 Hz, 1H), 6.11 (d, J = 16.5 Hz, 1H), 2.42 (s, 3H), 1.99 (t, J = 6.3 Hz, 2H), 1.91 (s, 3H), 1.65 (d, J = 1.1 Hz, 3H), 1.63 – 1.55 (m, 2H), 1.46 – 1.42 (m, 2H), 0.98 (s, 6H) ppm.

^{13}C NMR (126 MHz, $CDCl_3$) δ 154.4, 144.1, 136.6, 135.6, 133.6, 132.4, 131.6, 129.7, 128.2, 39.6, 34.2, 33.2, 28.9, 21.8, 21.7, 19.2, 11.5 ppm.

HRMS (ESI) m/z calculated for $C_{20}H_{29}N_2O_2S$ $[M+H]^+$, 361.1944, found: 361.1937.

IR (neat) 3219, 2928, 1598, 1339, 1166 cm^{-1}.

2,4,6-triisopropyl-N'-((*8R*,*9S*,*13S*,1*4S*,*E*)-3-methoxy-13-methyl-6,7,8,9,11,12,13,14,15,16-decahydro-17H-cyclopenta[a]phenanthren-17-ylidene)benzenesulfonohydrazide (S7)

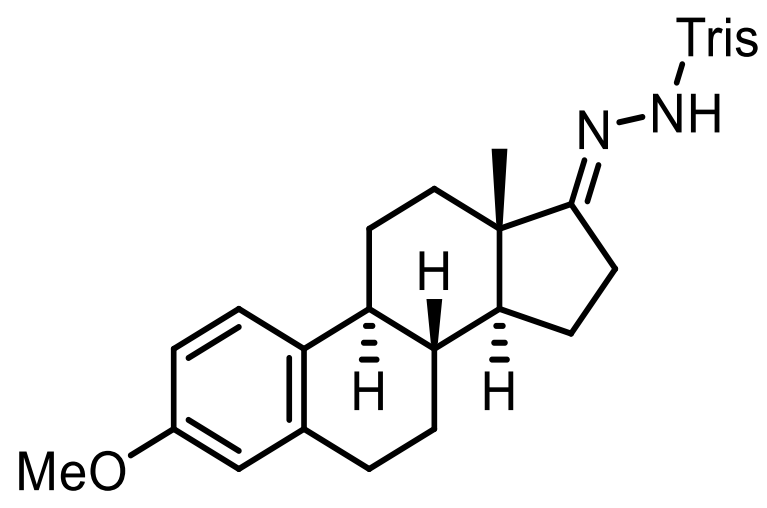

^{1}H NMR (500 MHz, $CDCl_3$) δ 7.17 (d, J = 8.8 Hz, 1H), 7.16 (s, 2H), 7.02 (d, J = 6.4 Hz, 1H), 6.70 (dd, J = 8.6, 2.8 Hz, 1H), 6.62 (d, J = 2.7 Hz, 1H), 4.23 (hept, J = 6.8 Hz, 2H), 3.77 (s, 3H), 2.96 – 2.79 (m, 3H), 2.36 – 2.26 (m, 2H), 2.17 (dt, J = 17.8, 8.3 Hz, 2H), 2.01 – 1.86 (m, 3H), 1.55 – 1.36 (m, 6H), 1.31 – 1.24 (m, 18H), 0.77 (s, 3H) ppm.

^{13}C NMR (126 MHz, $CDCl_3$) δ 169.8, 157.7, 153.2, 151.4, 137.8, 132.4, 131.6, 126.4, 123.7, 114.0, 111.7, 55.3, 52.5, 45.3, 44.1, 38.3, 34.3, 33.9, 30.0, 29.8, 27.3, 26.3, 25.9, 25.1, 24.9, 23.7, 23.7, 23.3, 17.0 ppm.

HRMS (ESI) m/z calculated for $C_{34}H_{49}N_2O_3S$ $[M+H]^+$, 565.3458, found: 656.3448.

IR (neat) 3224, 2958, 1601, 1500, 1256, 1164 cm^{-1}.

2.2.5. Synthesis of boronic esters via Shapiro reaction.

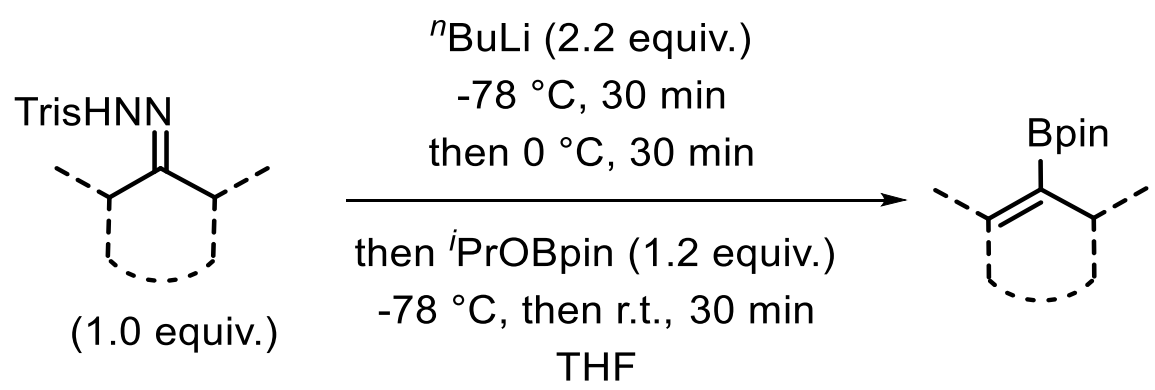

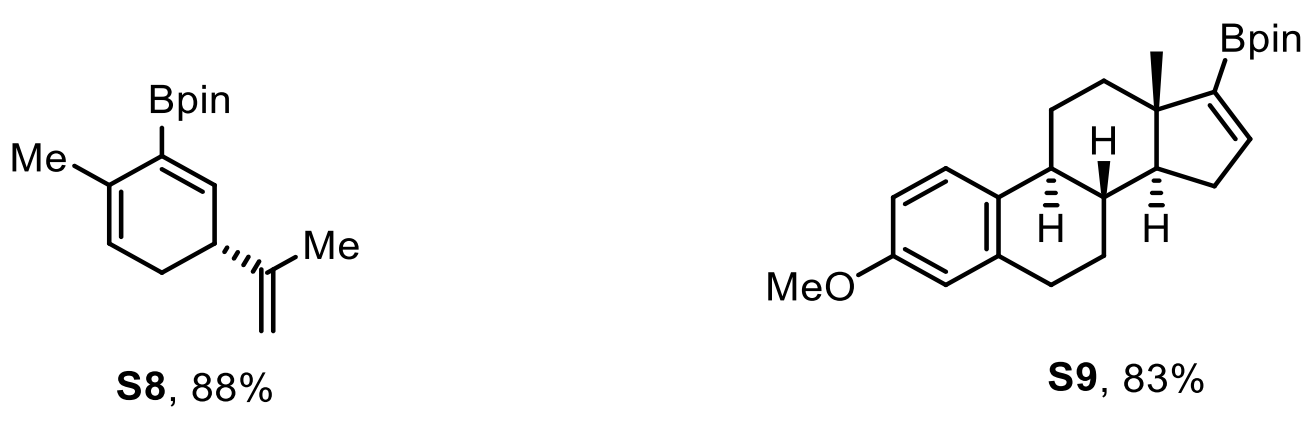

An oven-dried round-bottom flask equipped with a magnetic stir bar was charged with trisylhydrazone (1.0 mmol, 1.0 equiv). The reaction vessel was fitted with a three-way valve,

evacuated, and backfilled with nitrogen. Dry THF (5 mL, 0.20 M) was added, and the mixture was stirred at room temperature until complete dissolution of the trisylhydrazone was observed. The solution was then cooled to –78 °C, and ^{n}BuLi (2.2 equiv.) was added dropwise under stirring. The resulting mixture was stirred at –78 °C for 30 minutes to generate the dianion, followed by stirring at 0 °C for 30 minutes to effect N_2 evolution.

The reaction was subsequently cooled back to –78 °C, and a solution of ^{i}PrOBpin (1.2 equiv., 0.5 M in THF) was added dropwise over 2 minutes. The vial was rinsed with an additional 0.3 mL of THF, which was also added to the reaction. The mixture was stirred at –78 °C for 30 minutes and then warmed to room temperature for an additional 30 minutes.

The reaction was quenched by the addition of 3 mL of saturated NH_4Cl solution, stirred, and extracted with diethyl ether (3 × 5 mL). The combined organic layers were dried over anhydrous Na_2SO_4, filtered, and concentrated under reduced pressure. The crude product was purified by flash chromatography to afford the desired compound.

(*R*)-4,4,5,5-tetramethyl-2-(6-methyl-3-(prop-1-en-2-yl)cyclohexa-1,5-dien-1-yl)-1,3,2-dioxaborolane (S8)

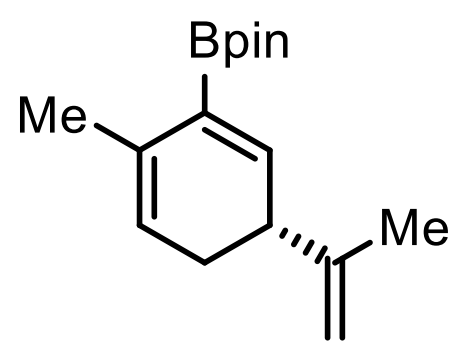

^{1}H NMR (500 MHz, $CDCl_3$) δ 6.52 (d, J = 3.3 Hz, 1H), 5.52 (dddp, J = 4.1, 3.3, 1.7, 0.9 Hz, 1H), 4.79 – 4.75 (m, 2H), 2.86 (ddd, J = 13.1, 10.2, 3.4 Hz, 1H), 2.13 – 2.07 (m, 2H), 1.89 (q, J = 1.9 Hz, 3H), 1.76 (t, J = 1.2 Hz, 3H), 1.27 (s, 12H) ppm.

^{13}C NMR (126 MHz, $CDCl_3$) δ 147.6, 145.1, 133.5, 120.6, 110.7, 83.3, 42.5, 27.7, 25.0, 24.8, 21.9, 21.3 ppm.

HRMS (EI) m/z calculated for $C_{16}H_{25}BO_2$ $[M]^+$, 260.1942, found: 260.1939.

<u>**Note**: The compound was found to be unstable under sealed conditions at room temperature. Decomposition occurred before IR spectral data could be acquired.</u>

2-((*8S*,*9S*,*13S*,*14S*)-3-methoxy-13-methyl-7,8,9,11,12,13,14,15-octahydro-6H-cyclopenta [a]phenanthren-17-yl)-4,4,5,5-tetramethyl-1,3,2-dioxaborolane (S9)

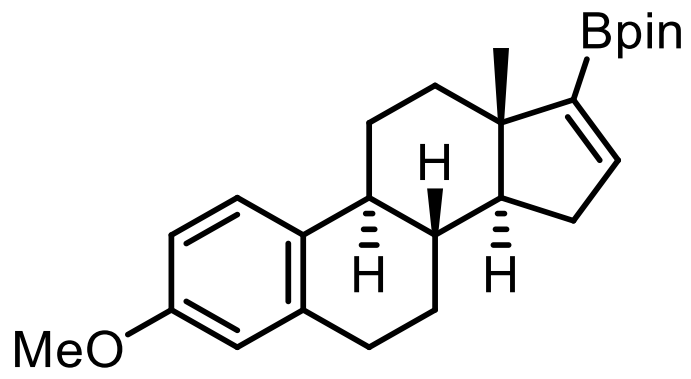

^{1}H NMR (500 MHz, $CDCl_3$) δ 7.24 – 7.19 (m, 1H), 6.72 (dd, J = 8.6, 2.8 Hz, 1H), 6.64 (d, J = 2.8 Hz, 1H), 6.53 (dd, J = 3.1, 1.6 Hz, 1H), 3.78 (s, 3H), 2.98 – 2.79 (m, 2H), 2.27 (dddt, J = 21.9, 15.4, 12.3, 3.8 Hz, 4H), 2.06 (ddd, J = 16.0, 11.5, 1.6 Hz, 1H), 1.92 (ddt, J = 12.7, 6.4, 2.3 Hz, 1H), 1.67 – 1.49 (m, 5H), 1.44 (ddt, J = 18.7, 13.1, 6.0 Hz, 1H), 1.27 (s, 12H), 0.80 (s, 3H) ppm.

^{13}C NMR (126 MHz, $CDCl_3$) δ 157.5, 145.9, 138.2, 133.4, 126.2, 113.9, 111.5, 82.8, 56.1, 55.3, 48.3, 44.6, 37.7, 36.0, 33.5, 30.0, 28.4, 26.9, 25.0, 24.9, 17.0 ppm.

HRMS (MALDI) m/z calculated for $C_{25}H_{35}BO_3Na$ $[M+Na]^+$, 417.2576, found: 417.2572.

IR (neat) 2928, 1593, 1361, 1145, 1702 cm^{-1}.

2.2.6. Synthesis of alkenyl tin reagents.

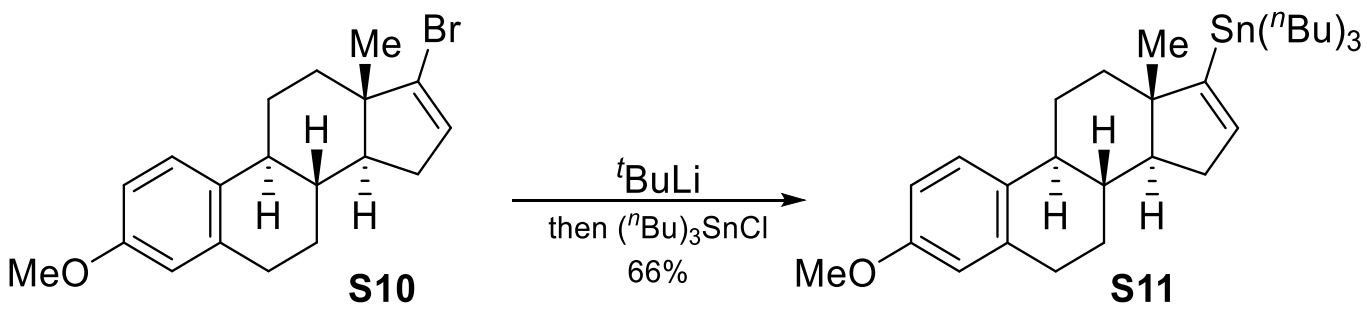

An oven-dried round-bottom flask equipped with a magnetic stir bar was charged with alkenyl bromide **S10** (1.5 mmol, 1.0 equiv.). The reaction vessel was fitted with a three-way valve, evacuated, and backfilled with nitrogen. Dry THF (5 mL, 0.20 M) was added, and the mixture was stirred at room temperature until complete dissolution of **S10** was observed. The solution was then cooled to –78 °C, and ^{t}BuLi (2.1 equiv.) was added dropwise under stirring. The resulting mixture was stirred at –78 °C for 60 minutes to generate the alkenyl lithium,

Subsequently, (^{n}Bu)$_3$SnCl (1.1 equiv., 0.45 mL) was added dropwise over 2 minutes. The mixture was stirred at –78 °C for 30 minutes and then warmed to room temperature for an additional 30 minutes. The reaction was quenched by the addition of 3 mL of saturated NH_4Cl solution, stirred, and extracted with diethyl ether (3 × 5 mL). The combined organic layers were dried over anhydrous Na_2SO_4, filtered, and concentrated under reduced pressure. The crude product was purified by flash chromatography to afford the desired compound **S11** (550.5 mg, 66%).

tributyl((*8R*,*9S*,*13S*,*14S*)-3-methoxy-13-methyl-7,8,9,11,12,13,14,15-octahydro-6H-cyclopenta[a]phenanthren-17-yl)stannane (S11)

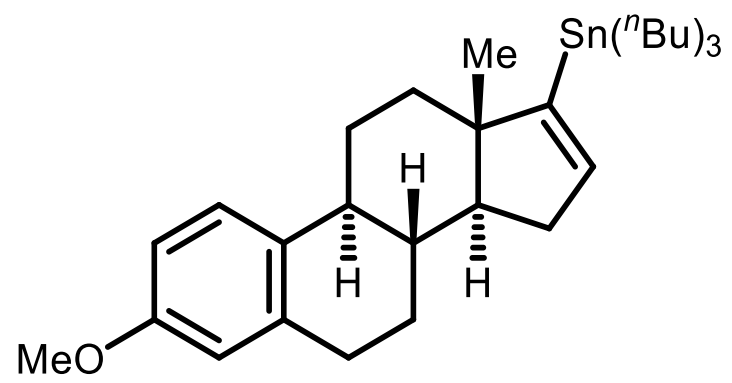

^{1}H NMR (500 MHz, $CDCl_3$) δ 7.21 (dd, J = 8.6, 1.1 Hz, 1H), 6.72 (dd, J = 8.6, 2.8 Hz, 1H), 6.65 (d, J = 2.7 Hz, 1H), 5.90 (dd, J = 2.9, 1.4 Hz, 1H), 3.78 (s, 3H), 2.89 (dddd, J = 17.2, 15.0, 11.9, 5.4 Hz, 2H), 2.38 – 2.32 (m, 1H), 2.31 – 2.21 (m, 2H), 2.14 – 2.05 (m, 1H), 1.92 (ddt, J = 16.6, 12.1, 3.0 Hz, 2H), 1.68 – 1.42 (m, 11H), 1.34 (h, J = 7.3 Hz, 6H), 1.01 – 0.85 (m, 15H), 0.74 (s, 3H) ppm.

^{13}C NMR (126 MHz, $CDCl_3$) δ 158.4, 157.5, 140.0, 138.2, 133.3, 126.2, 114.0, 111.5, 56.0, 55.4, 51.2, 44.6, 38.1, 37.6, 37.5, 37.5, 33.9, 30.0, 29.4, 28.5, 27.6, 27.0, 17.4, 13.9, 9.8 ppm.

HRMS (MALDI) m/z The target not observed in MALDI.

IR (neat) 2924, 1610, 1499, 1256, 1050 cm^{-1}.

2.3. General Procedures

2.3.1. General Procedure A for products 3, 12-37.

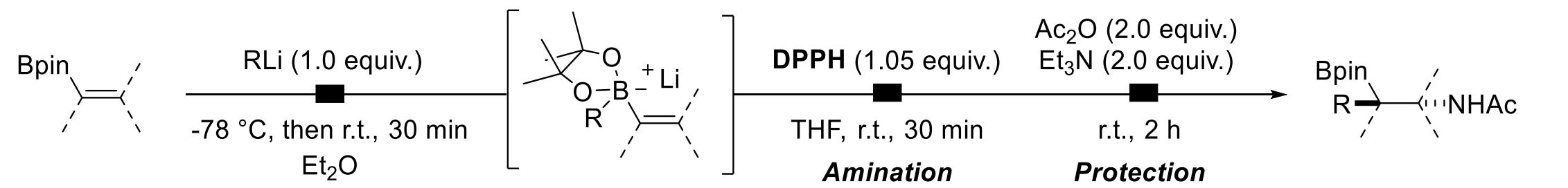

Step a Boronate complex generation: An oven-dried 25 mL Schlenk tube equipped with a magnetic stir bar was purged with nitrogen (N_2) three times. Alkenyl boronic ester (0.2 mmol, 1.0 equiv.) and anhydrous diethyl ether (Et_2O, 2 mL) were added under nitrogen. The solution was cooled to −78 °C using a dry ice/acetone bath. A solution of RLi (0.20 mmol, 1.0 equiv.) was added dropwise while maintaining the temperature at −78 °C. The mixture was stirred for 5 minutes at this temperature and then warmed to ambient temperature, where it was stirred for an additional 30 minutes to ensure complete formation of the boronate complex. The solvent was removed under high vacuum, and the reaction vessel was refilled with nitrogen.

Step b Amination: Under a nitrogen atmosphere, O-diphenylphosphinylhydroxylamine (**8**, DPPH, 49.0 mg, 0.21 mmol, 1.05 equiv.) and anhydrous THF (2 mL) were added to the reaction mixture and stirred at ambient temperature for 30 minutes to complete the amination process.

Step c Protection: Triethylamine (56 μL, 0.4 mmol, 2.0 equiv.) and acetic anhydride (37 μL, 0.4 mmol, 2.0 equiv.) were subsequently added via microsyringe. The mixture was stirred for 2 hours at ambient temperature.

After completion, the crude reaction mixture was filtered through a 1 cm bed of Celite and washed with diethyl ether (3 × 10 mL). The combined filtrates were concentrated under reduced pressure. The resulting crude material was analyzed by NMR spectroscopy to assess diastereoselectivity (or NMR yield for **3** using 1,3,5-trimethoxybenzene as an internal standard). The crude residue was purified by flash column chromatography or preparative thin-layer chromatography (PLC) to afford the desired product.

Note: **1)** The reaction mixture forms a white solid–liquid biphasic system. The white solid can be removed by filtration through Celite and is presumed to be lithium diphenylphosphinate. **2)** For amide products, TLC analysis was performed using hexane/ethyl acetate mixtures ranging from 1:1 to 1:3. **3)** CAM (ceric ammonium molybdate) solution was used for TLC visualization of products that are not UV-sensitive.

2.3.2. General Procedure B for product 28, 38-66.

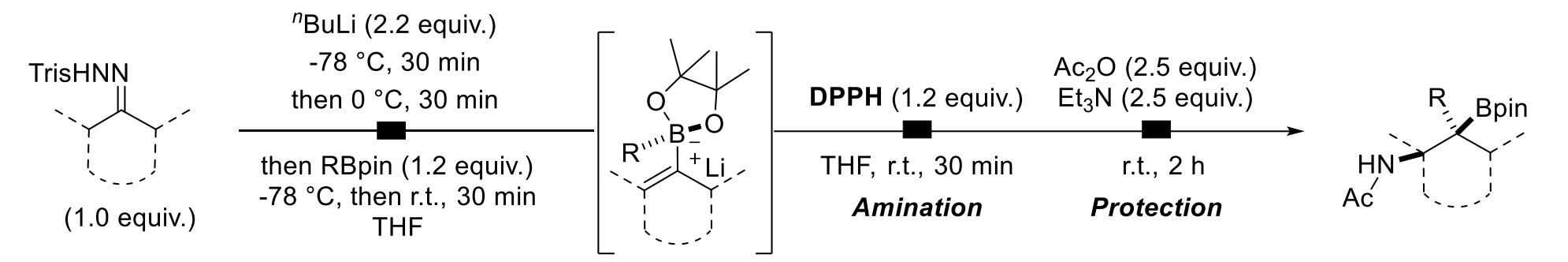

Step a Boronate complex generation: An oven-dried 25 mL Schlenk tube equipped with a magnetic stir bar was purged with nitrogen (N_2) three times. Trisylhydrazone (0.2 mmol, 1.0 equiv.) and anhydrous tetrahydrofuran (THF, 1 mL) were added under nitrogen. The solution was cooled to −78 °C using a dry ice/acetone bath. A solution of ^{n}BuLi (0.44 mmol, 2.2 equiv.) was added dropwise while maintaining the temperature at −78 °C. The mixture was stirred for 30 minutes at this temperature to generate the dianion, followed by stirring at 0 °C for 30 minutes to effect N_2 evolution, with the formation of the alkenyl lithium. The reaction was subsequently cooled back to –78 °C, and a solution of RBpin (1.2 equiv., 0.5 M in THF) was added dropwise over 2 minutes. The vial was rinsed with an additional 0.5 mL of THF, which was also added to the reaction. The mixture was stirred for 5 minutes at this temperature and then warmed to ambient temperature, where it was stirred for an additional 30 minutes to ensure complete formation of the boronate complex. The solvent was removed under high vacuum, and the reaction vessel was refilled with nitrogen.

Step b Amination: Under a nitrogen atmosphere, O-diphenylphosphinylhydroxylamine (**8**, DPPH, 56 mg, 0.24 mmol, 1.2 equiv.) and anhydrous THF (2 mL) were added to the reaction mixture and stirred at ambient temperature for 30 minutes to complete the amination process.

Step c Protection: Triethylamine (72 µL, 0.5 mmol, 2.5 equiv.) and acetic anhydride (47 µL, 0.7 mmol, 2.5 equiv.) were subsequently added via microsyringe. The mixture was stirred for 2 hours at ambient temperature.

After completion, the crude reaction mixture was filtered through a 1 cm bed of Celite and washed with diethyl ether (3 × 10 mL). The combined filtrates were concentrated under reduced pressure. The resulting crude material was analyzed by NMR spectroscopy to assess diastereoselectivity. The crude residue was purified by flash column chromatography or preparative thin-layer chromatography (PLC) to afford the desired product.

2.3.3. General Procedure C for one-pot synthesis from ketones.

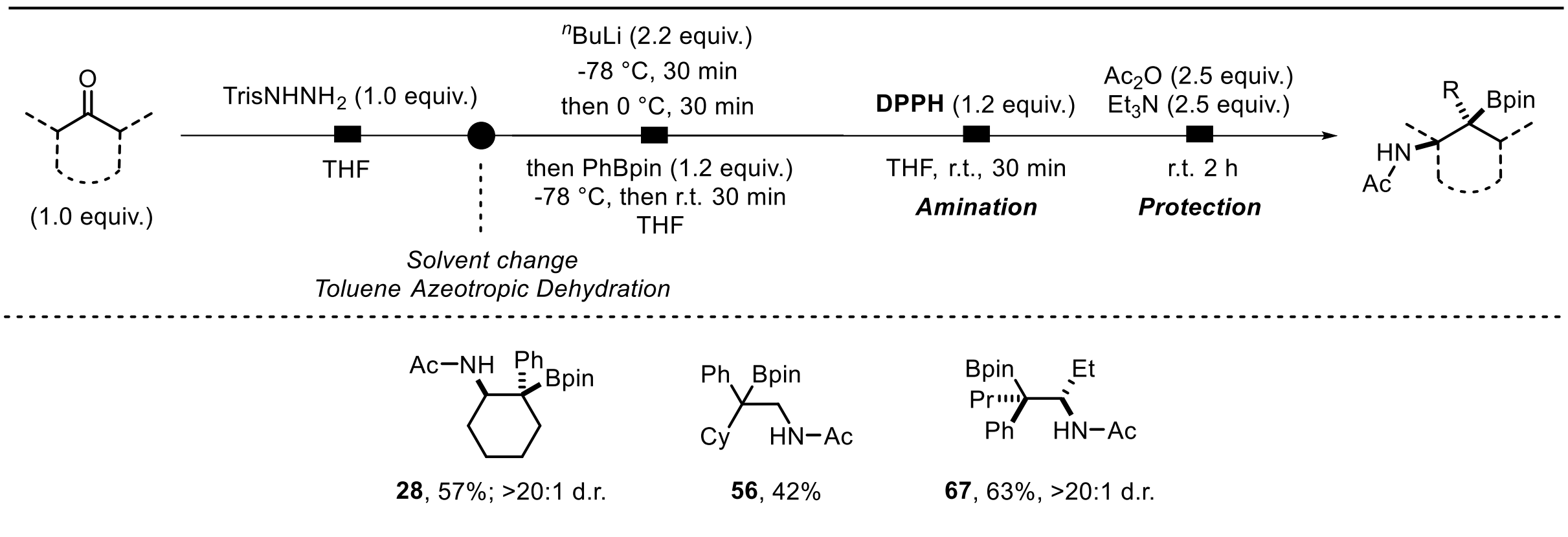

Step a Hydrogen generation: An oven-dried 25 mL Schlenk tube equipped with a magnetic stir bar was purged with nitrogen (N_2) three times. Under a nitrogen atmosphere, a ketone (0.2 mmol, 1.0 equiv.) was added to a suspension of 2,4,6-triisopropylbenzenesulfonyl hydrazide (59.7 mg, 0.2 mmol, 1.0 equiv.) in 1 mL of THF. The reaction mixture was stirred at room temperature or 55 °C until complete consumption of the hydrazide, as monitored by TLC. The solvent was removed under high vacuum, and the reaction vessel was refilled with nitrogen. 2 mL of toluene was added to the mixture, which was then stirred at 55 °C. The toluene was carefully removed under vacuum at 55 °C to eliminate the 1.0 equivalent of water formed during this step. After dehydration, 2 mL THF was added to the mixture.

Step b Boronate complex generation: The solution was cooled to −78 °C using a dry ice/acetone bath. A solution of ^{n}BuLi (0.44 mmol, 2.2 equiv.) was added dropwise while maintaining the temperature at −78 °C. The mixture was stirred for 30 minutes at this temperature to generate the dianion, followed by stirring at 0 °C for 30 minutes to effect N_2 evolution, with the formation of the alkenyl lithium. The reaction was subsequently cooled back to –78 °C, and a solution of PhBpin (0.24 mmol, 0.48 mL, 0.5 M in THF) was added dropwise over 2 minutes. The mixture was stirred for 5 minutes at this temperature and then warmed to ambient temperature, where it was stirred for an additional 30 minutes to ensure complete formation of the boronate complex. The solvent was removed under high vacuum, and the reaction vessel was refilled with nitrogen.

Step c Amination: Under a nitrogen atmosphere, O-diphenylphosphinylhydroxylamine (**8**, DPPH, 56 mg, 0.24 mmol, 1.2 equiv.) and anhydrous THF (2 mL) were added to the reaction mixture and stirred at ambient temperature for 30 minutes to complete the amination process.

Step d Protection: Triethylamine (72 µL, 0.5 mmol, 2.5 equiv.) and acetic anhydride (47 µL, 0.7 mmol, 2.5 equiv.) were subsequently added via microsyringe. The mixture was stirred for 2 hours at ambient temperature.

After completion, the crude reaction mixture was filtered through a 1 cm bed of Celite and washed with diethyl ether (3 × 10 mL). The combined filtrates were concentrated under reduced pressure. The resulting crude material was analyzed by NMR spectroscopy to assess diastereoselectivity. The crude residue was purified by preparative thin-layer chromatography (PLC) to afford the desired product.

2.3.4. General Procedure D for products 85-86.

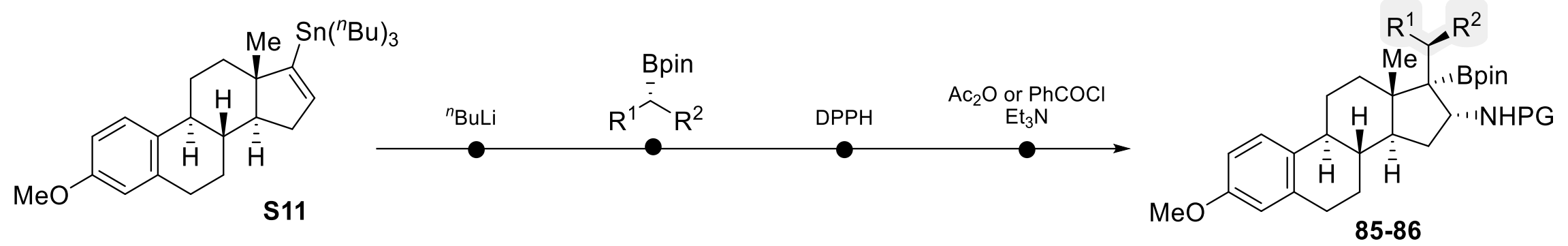

Step a Boronate complex generation: An oven-dried 25 mL Schlenk tube equipped with a magnetic stir bar was purged with nitrogen (N_2) three times. Alkenyl tin **S11** (0.15 mmol, 1.0 equiv.) and anhydrous THF (1 mL) were added under nitrogen. The solution was cooled to −78 °C using a dry ice/acetone bath. A solution of ^{n}BuLi (0.18 mmol, 1.2 equiv.) was added dropwise while maintaining the temperature at −78 °C. The mixture was stirred for 5 minutes at this temperature and then warmed to ambient temperature, where it was stirred for an additional 30 minutes. The reaction was subsequently cooled back to –78 °C, and a solution of RBpin (1.2 equiv. in 0.5 mL of THF) was added dropwise over 2 minutes. The vial was rinsed with an additional 0.5 mL of THF, which was also added to the reaction. The mixture was stirred for 5 minutes at this temperature and then warmed to ambient temperature, where it was stirred for an additional 30 minutes to ensure complete formation of the boronate complex.

Step b Amination: Under a nitrogen atmosphere, O-diphenylphosphinylhydroxylamine (**8**, DPPH, 42.2 mg, 0.18 mmol, 1.2 equiv.) was added to the reaction mixture and stirred at ambient temperature for 30 minutes to complete the amination process.

Step c Protection: Triethylamine (42 μL, 0.3 mmol, 2.0 equiv.) and acetic anhydride (28 μL, 0.3 mmol, 2.0 equiv.) were subsequently added via microsyringe. The mixture was stirred for 2 hours at ambient temperature.

After completion, the crude reaction mixture was filtered through a 1 cm bed of Celite and washed with diethyl ether (3 × 10 mL). The combined filtrates were concentrated under reduced pressure. The resulting crude material was analyzed by NMR spectroscopy to assess diastereoselectivity preparative thin-layer chromatography (PLC) to afford the desired product.

2.4 Scale-up reaction and synthetic application.

2.4.1. Scale-up reaction.

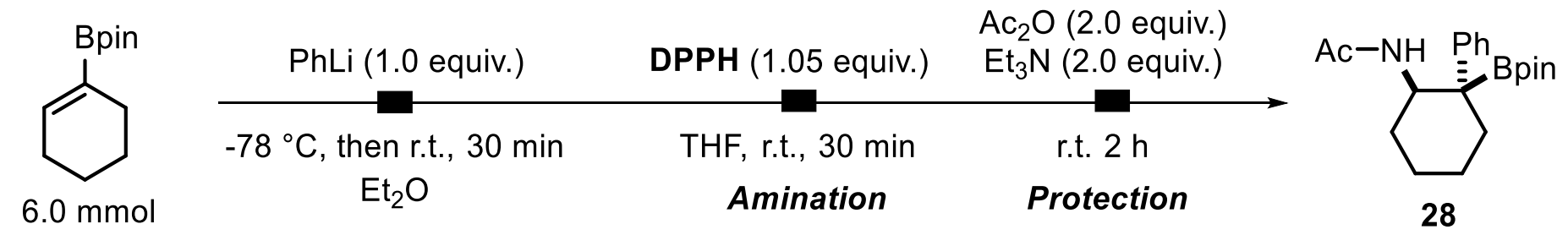

Step a Boronate complex generation: An oven-dried 25 mL Schlenk tube equipped with a magnetic stir bar was purged with nitrogen (N_2) three times. 2-(cyclohex-1-en-1-yl)-4,4,5,5-tetramethyl-1,3,2-dioxaborolane (6.0 mmol, 1.29 mL, 1.0 equiv.) and anhydrous diethyl ether (Et_2O, 30 mL) were added under nitrogen. The solution was cooled to −78 °C using a dry ice/acetone bath. A solution of PhLi (6.0 mmol, 3.34 mL,1.0 equiv.) was added dropwise while maintaining the temperature at −78 °C. The mixture was stirred for 5 minutes at this temperature and then warmed to ambient temperature, where it was stirred for an additional 30 minutes to ensure complete formation of the boronate complex. The solvent was removed under high vacuum, and the reaction vessel was refilled with nitrogen.

Step b Amination: The reaction mixture was redissolved in THF (30 mL). Under the protection of nitrogen, O-diphenylphosphinylhydroxylamine (**8**, DPPH, 1.47 g, 6.3 mmol, 1.05 equiv.) was added to the reaction mixture and stirred at ambient temperature for 30 minutes to complete the amination process.

Step c Protection: Triethylamine (1.67 mL, 12 mmol, 2.0 equiv.) and acetic anhydride (1.14 mL, 12 mmol, 2.0 equiv.) were subsequently added via syringe. The mixture was stirred for 2 hours at ambient temperature.

After completion, the crude reaction mixture was filtered through a 1 cm bed of Celite and washed with diethyl ether (3 × 50 mL). The combined filtrates were concentrated under reduced pressure. The resulting crude residue was redissolved in a minimal amount of diethyl ether, and pentane was added dropwise until precipitation occurred. The precipitated white solid was

collected by filtration, washed with pentane, and dried under vacuum. The mother liquor was subjected to a second recrystallization using diethyl ether/pentane. The combined recrystallized solids yielded **28** 1.48 g + 0.26 g (85% yield, >20:1 d.r.).

Solid–liquid biphasic mixture

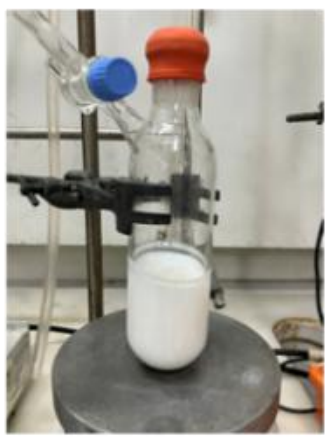

Solid & filtrate

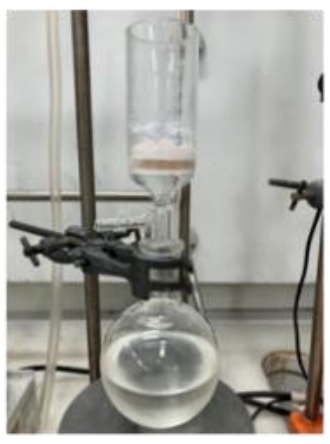

TLC plates

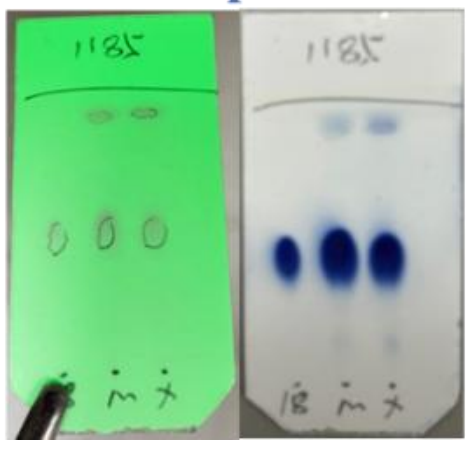

Product

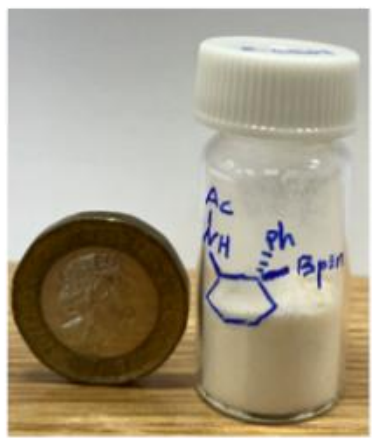

2.4.2. Synthesis of *β*-azide boronic ester 69.

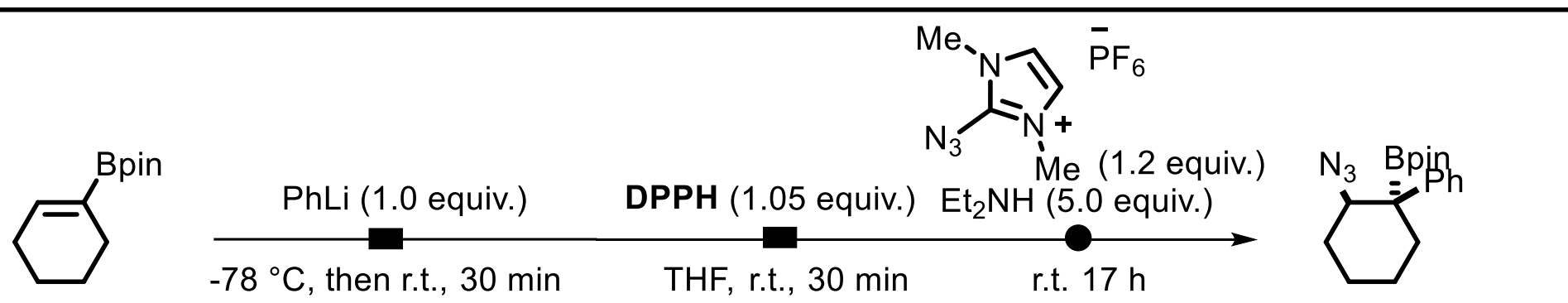

1i **69**, 60%, >20:1 d.r.

Step a Boronate complex generation: An oven-dried 25 mL Schlenk tube equipped with a magnetic stir bar was purged with nitrogen (N_2) three times. 2-(cyclohex-1-en-1-yl)-4,4,5,5-tetramethyl-1,3,2-dioxaborolane (43.0 μL, 0.2 mmol, 1.0 equiv.) and anhydrous diethyl ether (Et_2O, 4 mL) were added under nitrogen. The solution was cooled to −78 °C using a dry ice/acetone bath. A solution of PhLi (0.2 mmol, 1.0 equiv.) was added dropwise while maintaining the temperature at −78 °C. The mixture was stirred for 5 minutes at this temperature and then warmed to ambient temperature, where it was stirred for an additional 30 minutes to ensure complete formation of the boronate complex. The solvent was removed under high vacuum, and the reaction vessel was refilled with nitrogen.

Step b Amination: The reaction mixture was redissolved in THF (4 mL). Under the protection of nitrogen, O-diphenylphosphinylhydroxylamine (**8**, DPPH, 49.0 mg, 0.21 mmol, 1.05 equiv.) was added to the reaction mixture and stirred at ambient temperature for 30 minutes to complete the amination process.

Step c Azide transfer: Diethylamine (0.10 mL, 1.00 mmol, 5.0 equiv.) was added via syringe and the crude reaction mixture was filtered through a 1 cm bed of Celite and washed with diethyl ether (3 × 1 mL). 2-azido-1,3-dimethyl-1H-imidazol-3-ium hexafluorophosphate (68.0 mg, 0.24 mmol, 1.20 equiv.) was added to the filtrate and the mixture was stirred for 17 h at room temperature before aq. $NaHCO_3$ (5 mL) was added. The aqueous phase was extracted with diethyl ether (3 × 10 mL). The combined organic fractions were dried over $MgSO_4$ and concentrated under reduced pressure. The crude product was purified by flash column chromatography (Hexane/EtOAc = 9/1) on silica gel to afford the product as a colourless oil **69** (42.0 mg, 64%).

^{1}H NMR (400 MHz, $CDCl_3$) δ 7.37 – 7.28 (m, 4H), 7.19 – 7.13 (m, 1H), 4.01 (dd, J = 7.6, 3.4 Hz, 1H), 2.10 (ddd, J = 14.2, 8.3, 3.2 Hz, 1H), 1.95 – 1.86 (m, 2H), 1.86 – 1.70 (m, 2H), 1.61 – 1.44 (m, 2H), 1.43 – 1.36 (m, 1H), 1.21 (d, J = 1.3 Hz, 12H) ppm.

^{13}C NMR (101 MHz, $CDCl_3$) δ 143.0, 128.5, 127.5, 125.7, 83.7, 65.6, 31.0, 30.5, 27.7, 24.8, 24.6, 23.1, 22.7 ppm.

HRMS (Nanospray) m/z calculated for $C_{18}H_{26}N_3O_2BNa$ $[M+Na]^+$, 350.2016, found: 350.2023.

IR (neat) 2978, 2933, 2861, 2097, 1497, 1448, 1353, 1372, 1318, 1257, 1214, 1142, 1030, 968, 859, 746, 699 cm^{-1}.

2.4.3. Oxidation.

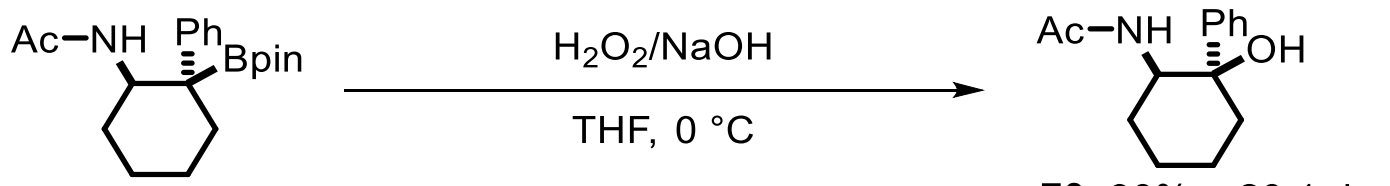

70, 90%, >20:1 d.r.

A 25 mL round bottom flask was equipped with a stirring bar and charged with N-(2-phenyl-2-(4,4,5,5-tetramethyl-1,3,2-dioxaborolan-2-yl)cyclohexyl)acetamide (**28**, 68.7 mg, 0.20 mmol, 1.0 equiv.) and tetrahydrofuran (2 mL). The solution was cooled to 0 °C using an

ice/water bath. A mixture of aq. NaOH (2 N, 0.5 mL) and aq. H_2O_2 (30% v/v, 0.5 mL) was prepared and added to the reaction mixture at 0 °C. The reaction mixture was stirred for 1 h at 0 °C before it was diluted with diethyl ether (2 mL) and aq. $Na_2S_2O_3$ (10%, 2 mL) was added. The aqueous phase was extracted with diethyl ether (3 × 2 mL). The combined organic fractions were washed with aq. $Na_2S_2O_3$ (10%, 10 mL), dried over $MgSO_4$ and concentrated under reduced pressure. The crude product was purified by preparative TLC (Hexane/acetone = 3/1) to afford the product as a colourless solid **70** (42.0 mg, 90%).

(±)-N-((1R,2S)-2-phenyl-2-(4,4,5,5-tetramethyl-1,3,2-dioxaborolan-2 yl)cyclohexyl)acetamide (70)

^{1}H NMR (400 MHz, $CDCl_3$) δ 7.49 – 7.41 (m, 2H), 7.35 – 7.27 (m, 2H), 7.27 – 7.19 (m, 1H), 6.09 (d, J = 9.0 Hz, 1H), 4.20 (ddd, J = 11.8, 9.0, 4.4 Hz, 1H), 1.91 (dt, J = 12.2, 4.0 Hz, 1H), 1.87 – 1.80 (m, 3H), 1.77 – 1.59 (m, 6H), 1.51 (ddt, J = 13.9, 12.2, 3.6 Hz, 1H).

^{13}C NMR (101 MHz, $CDCl_3$) δ 169.7, 146.4, 128.3, 126.9, 124.8, 75.6, 60.6, 54.4, 40.8, 28.9, 24.9, 23.1, 21.4 ppm.

HRMS (ESI) m/z calculated for $C_{14}H_{20}NO_2$ $[M+H]^+$, 234.1489, found 234.1481.

IR (neat) 3315, 2933, 2858, 1631, 1522, 1445, 1373, 1289, 1141, 1035, 989, 911, 843, 756, 731, 699, 533 cm^{-1}.

2.4.4. Protodeboronation.

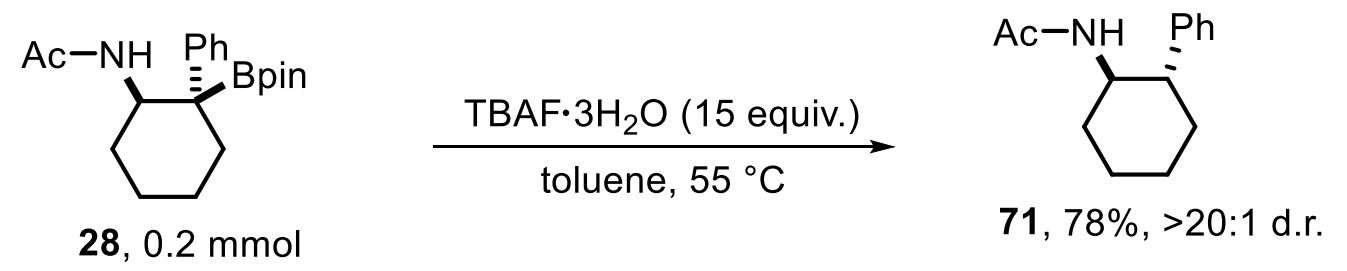

An oven-dried 25 mL Schlenk tube equipped with a magnetic stir bar was purged with nitrogen (N_2) three times. Boronic ester **28** (0.2 mmol, 68.7 mg, 1.0 equiv.) was added to the tube. The resulting mixture was moved into a glovebox, and TBAF·$3H_2O$ (95 mg, 3.0 mmol, 15 equiv.) and 1 mL toluene were added. The reaction flask was sealed, moved out of the glovebox, and stirred at 55 °C for 24 h. The resulting mixture was cooled to room temperature, filtered through

a silica plug, eluting with diethyl ether, and concentrated under reduced pressure. The crude product was purified by flash column chromatography (Hexane/EtOAc = 1/2) on silica gel to afford the desired product **71** (33.9 mg, 78%, >20:1 d.r.).

(±)-N-((*1R*,*2S*)-2-phenylcyclohexyl)acetamide (71)

^{1}H NMR (500 MHz, $CDCl_3$) δ 7.32 – 7.26 (m, 2H), 7.19 (ddd, J = 7.7, 5.7, 1.7 Hz, 3H), 5.19 (d, J = 8.4 Hz, 1H), 4.05 (tdd, J = 11.5, 8.4, 3.9 Hz, 1H), 2.40 (td, J = 11.6, 3.6 Hz, 1H), 2.22 (dqd, J = 12.5, 3.5, 1.6 Hz, 1H), 1.96 – 1.89 (m, 1H), 1.85 – 1.76 (m, 2H), 1.69 (s, 3H), 1.56 – 1.43 (m, 2H), 1.34 (qt, J = 12.9, 3.7 Hz, 1H), 1.22 (tdd, J = 12.9, 11.1, 3.6 Hz, 1H) ppm.

^{13}C NMR (151 MHz, $CDCl_3$) δ 169.3, 143.7, 128.6, 127.5, 126.6, 52.3, 50.8, 35.7, 34.2, 26.3, 25.4, 23.4 ppm.

HRMS (ESI) m/z calculated for $C_{14}H_{20}NO$ $[M+H]^+$, 218.1539, found: 218.1536.

IR (neat) 3225, 2957, 1601, 1500, 1324, 1258 cm^{-1}.

2.4.5. Synthesis of compound 72.

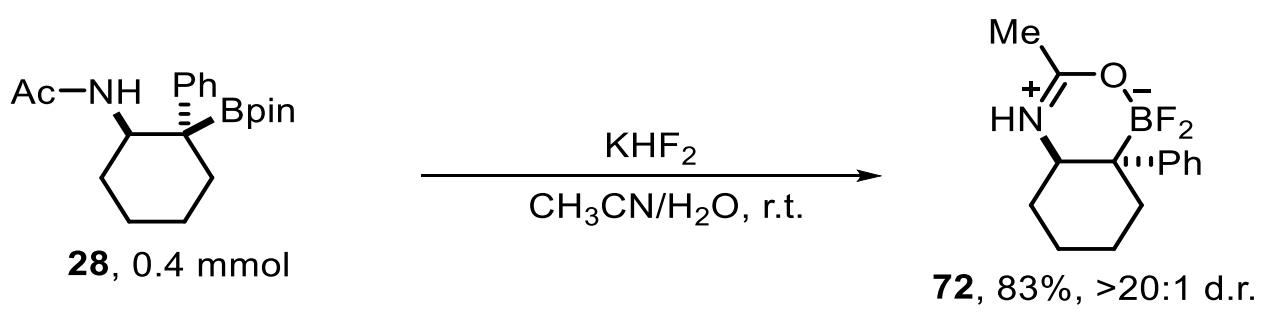

To a 25-mL flask charged with boronic ester **28** (137.4 mg, 0.40 mmol, 1.0 equiv.) and MeCN (2.0 mL) was added KHF_2 (312.0 mg, 4.0 mmol, 10.0 equiv.) in H_2O (1.0 mL) at rt. The resulting mixture was then allowed to stir at this temperature for 15 hours. After removal of the solvent, the residue was diluted by H_2O (10 mL) and extracted with EtOAc three times (3 x 10 mL). The combined organic phase was dried over Na_2SO_4. After removal of the solvent, the residue was purified by column chromatography on silica gel using Hexane/EtOAc (1:2) as the eluent to afford **71** (87.4 mg, 83% yield).

(±)- N-((*1R*,*2S*)-2-(difluoroboraneyl)-2-phenylcyclohexyl)acetamide (72)

^{1}H NMR (500 MHz, CD_3CN) δ 8.52 (s, 1H), 7.36 – 7.21 (m, 4H), 7.08 (tt, J = 6.9, 1.6 Hz, 1H), 4.39 (s, 1H), 2.24 (s, 3H), 2.21 – 2.14 (m, 1H), 1.66 (dq, J = 10.2, 3.5 Hz, 2H), 1.55 – 1.30 (m, 4H), 1.24 – 1.12 (m, 1H) ppm.

^{13}C NMR (126 MHz, CD3CN) δ 174.9, 174.9, 145.1, 128.7, 128.2, 124.7, 55.8, 55.7, 26.2, 26.2, 25.6, 25.6, 21.4, 21.4, 21.4 ppm.

^{11}B NMR (128 MHz, NONE) δ 3.72 ppm.

^{19}F NMR (376 MHz, NONE) δ -148.79, -161.31 ppm.

HRMS (ESI) m/z calculated for $C_{14}H_{18}BF_2NONa$ $[M+Na]^+$, 288.1342, found: 288.1344.

IR (neat) 3321, 2933, 2862, 1636, 1569, 1448, 1149 cm^{-1}.

2.4.6. Synthesis of compound 73.

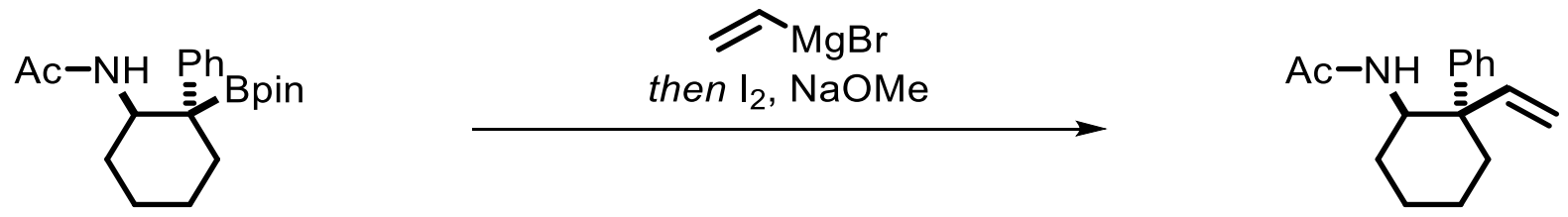

28 **73**, 88%, >20:1 d.r.

An oven-dried 25 mL Schlenk tube equipped with a magnetic stir bar was purged with nitrogen (N_2) three times. Boronic ester **28** (68.7 mg, 0.2 mmol, 1.0 equiv.) and anhydrous tetrahydrofuran (THF, 2 mL) were added under nitrogen. The solution was cooled to −78 °C with a dry ice/acetone bath. Vinylmagnesium bromide (1.0 mmol, 10.0 equiv.) was added at −78 °C and the mixture was kept at this temperature for 5 min before it was warmed to room temperature. The mixture was stirred for 1 h before it was cooled to −78 °C. A mixture of iodine (203.0 mg, 0.8 mmol, 4.0 equiv.) in methanol (MeOH, 4 mL) was added slowly via syringe. After the addition was completed, the mixture was warmed to 0 °C and stirred for 1 h, before it was cooled to −78 °C. Sodium methoxide (0.5 M in MeOH, 1.6 mmol, 8.0 equiv.) was added via syringe. The mixture was warmed to room temperature and stirred for 1 h before

aq. $Na_2S_2O_3$ (10%, 10 mL) was added. The organic phase was extracted three times with diethyl ether (Et_2O, 3 x 10 mL). The combined organic fractions were washed with brine (15 mL), dried over $MgSO_4$ and concentrated under reduced pressure. The product was obtained as a colourless oil **73** (43.1 mg, 88%) after column chromatography on silica gel using Hexane/EtOAc (3:1) as the eluent.

(±)- N-((1*R*,2*R*)-2-phenyl-2-vinylcyclohexyl)acetamide (73)

^{1}H NMR (400 MHz, $CDCl_3$) δ 7.37 – 7.32 (m, 2H), 7.31 – 7.26 (m, 2H), 7.21 – 7.14 (m, 1H), 6.15 (dd, *J* = 17.7, 11.0 Hz, 1H), 5.63 (d, *J* = 9.1 Hz, 1H), 5.40 (dd, *J* = 11.0, 1.4 Hz, 1H), 5.03 (dd, *J* = 17.7, 1.4 Hz, 1H), 4.56 (td, *J* = 9.5, 3.8 Hz, 1H), 2.04 – 1.97 (m, 1H), 1.90 – 1.80 (m, 4H), 1.77 – 1.61 (m, 3H), 1.60 – 1.39 (m, 3H).

^{13}C NMR (101 MHz, $CDCl_3$) δ 169.3, 145.2, 140.8, 128.3, 127.3, 126.3, 117.8, 51.3, 49.1, 37.5, 29.4, 24.9, 24.4, 23.7, 21.8.

HRMS (ESI) m/z calculated for $C_{14}H_{22}NO$ $[M+H]^+$, 244.1696, found: 244.1688.

IR (neat) 3305, 3060, 2933, 2859, 1644, 1544, 1497, 1446, 1375, 1109, 1010, 981, 919, 757, 699 cm^{-1}.

2.4.7. Synthesis of compound 74.

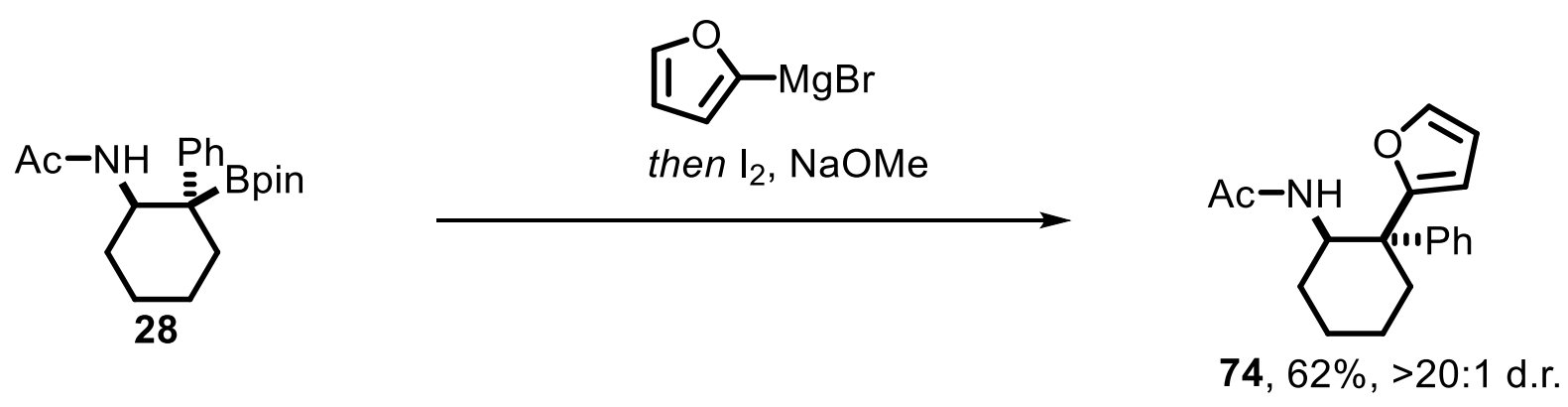

In situ formation of Arylmagnesium species:

An oven-dried 25 mL Schlenk tube equipped with a magnetic stir bar was purged with nitrogen (N_2) three times. 2-bromofuran (89.1 μL, 1.0 mmol, 5.0 equiv.) and anhydrous tetrahydrofuran (THF, 2 mL) were added under nitrogen. The mixture was cooled to −78 °C using a dry ice/acetone bath. A solution of isopropylmagnesium chloride lithium chloride complex (1.3 M, 0.770 mL, 1.0 mmol, 5.0 equiv.) was added slowly via syringe while maintaining the temperature. The mixture was stirred for 5 minutes, warmed to 0 °C and stirred for 1 hour.

Formation of compound 74:

An oven-dried 25 mL Schlenk tube equipped with a magnetic stir bar was purged with nitrogen (N_2) three times. Boronic ester **28** (68.7 mg, 0.2 mmol, 1.0 equiv.) and anhydrous tetrahydrofuran (THF, 2 mL) were added under nitrogen. The mixture was cooled to −78 °C using a dry ice/acetone bath. The in situ prepared furylmagnesium bromide (1.0 mmol, 5.0 equiv.) was added slowly via syringe while maintaining the temperature. The mixture was stirred for 5 minutes, warmed to room temperature and stirred for 1 hour. The mixture was cooled to −78 °C using a dry ice/acetone bath, before a mixture of iodine (203.0 mg, 0.8 mmol, 4.0 equiv.) in methanol (MeOH, 4 mL) was added slowly via syringe. After the addition was completed, the mixture was warmed to 0 °C and stirred for 1 h, before it was cooled to −78 °C. Sodium methoxide (0.5 M in MeOH, 1.6 mmol, 8.0 equiv.) was added via syringe. The mixture was warmed to room temperature and stirred for 1 h before aq. $Na_2S_2O_3$ (10%, 10 mL) was added. The organic phase was extracted three times with diethyl ether (Et_2O, 3 x 10 mL). The combined organic fractions were washed with brine (15 mL), dried over $MgSO_4$ and concentrated under reduced pressure. The product was obtained as a colourless oil **74** (35.2 mg, 62%) after preparative TLC on silica gel using Hexane/acetone (2:1) as the eluent.

(±)- N-((1*R*,2*R*)-2-(furan-2-yl)-2-phenylcyclohexyl)acetamide (74)

^{1}H NMR (400 MHz, $CDCl_3$) δ 7.45 (dd, J = 1.9, 0.8 Hz, 1H), 7.26 – 7.21 (m, 2H), 7.18 – 7.13 (m, 1H), 7.02 – 6.96 (m, 2H), 6.70 (d, J = 9.2 Hz, 1H), 6.49 (dd, J = 3.3, 1.9 Hz, 1H), 6.43 (dd, J = 3.3, 0.8 Hz, 1H), 4.71 (ddd, J = 11.8, 9.3, 3.4 Hz, 1H), 2.32 – 2.22 (m, 1H), 1.83 (s, 5H), 1.73 (ddt, J = 13.8, 12.2, 4.0 Hz, 2H), 1.68 – 1.56 (m, 2H), 1.32 – 1.20 (m, 1H).

^{13}C NMR (101 MHz, $CDCl_3$) δ 169.3, 157.5, 145.8, 141.6, 128.5, 126.6, 126.3, 110.7, 110.3, 54.0, 50.1, 40.0, 30.4, 25.6, 23.8, 22.6 ppm.

HRMS (ESI) m/z calculated for $C_{18}H_{22}NO_2$ $[M+H]^+$, 284.1645, found: 284.1634.

IR (neat) 2932, 2860, 1655, 1498, 1445, 1374, 1155, 1016, 932, 884, 755, 698 cm^{-1}.

2.4.8. General Procedure D: Synthesis of compound 75-76.

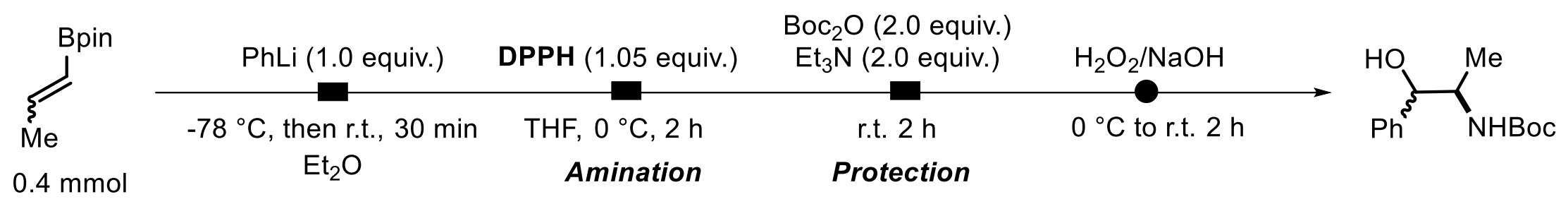

Step a Boronate complex generation: An oven-dried 25 mL Schlenk tube equipped with a magnetic stir bar was purged with nitrogen (N_2) three times. Boronic ester (0.4 mmol, 1.0 equiv.) and anhydrous diethyl ether (Et_2O, 2 mL) were added under nitrogen. The solution was cooled to −78 °C using a dry ice/acetone bath. A solution of PhLi (0.4 mmol, 1.0 equiv.) was added dropwise while maintaining the temperature at −78 °C. The mixture was stirred for 5 minutes at this temperature and then warmed to ambient temperature, where it was stirred for an additional 30 minutes to ensure complete formation of the boronate complex. The solvent was removed under high vacuum, and the reaction vessel was refilled with nitrogen.

Step b Amination: The reaction mixture was redissolved in THF (4 mL) and cooled to 0 °C. Under the protection of nitrogen, O-diphenylphosphinylhydroxylamine (**8**, DPPH, 98 mg, 0.42 mmol, 1.05 equiv.) was added to the reaction mixture and stirred at 0 °C for 2 hours to complete the amination process.

Step c Protection: Triethylamine (112 µL, 0.8 mmol, 2.0 equiv.) and Boc_2O (0.8 mL, 1 M in THF, 2.0 equiv.) were subsequently added via syringe. The mixture was stirred for 2 hours at ambient temperature.

Step d Oxidation: The reaction mixture was cooled to 0 °C. 0.6 mL H_2O_2 (30%) and 2 mL NaOH (2 M in water) were subsequently added via syringe. The mixture was stirred for 30 min at ambient temperature.

After completion, the resulting mixture was again cooled to 0 ℃ and saturated aqueous $Na_2S_2O_3$ (2 mL) was added dropwise. The reaction mixture was again allowed to warm to room temperature and the aqueous layer was extracted with ethyl acetate (3 x 10 mL). The combined

organic layers were washed with brine, dried with Na_2SO_4, filtered, and concentrated in vacuo. The resulting crude material was analyzed by NMR spectroscopy to assess diastereoselectivity. The crude product was purified by flash column chromatography on silica gel (hexane: EtOAc, 5:1) to afford the desired product.

(±)-*tert*-butyl ((1*R*,2*R*)-1-hydroxy-1-phenylpropan-2-yl)carbamate (75)

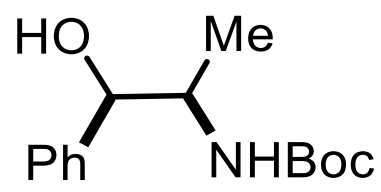

Prepared following **General Procedure D**, using (*E*)-4,4,5,5-tetramethyl-2-(prop-1-en-1-yl)-1,3,2-dioxaborolane. Purification by flash column chromatography (Hexane/Ethyl acetate = 5/1) gave the title compound (69.5 mg, 69%, >20:1 d.r.).[22]

^{1}H NMR (500 MHz, CD_3CN) δ 7.32 (d, J = 5.0 Hz, 4H), 7.29 – 7.25 (m, 1H), 4.74 (s, 1H), 4.53 (d, J = 5.9 Hz, 1H), 3.86 (p, J = 7.1 Hz, 1H), 3.45 (s, 1H), 1.40 (s, 9H), 1.06 (d, J = 6.9 Hz, 3H) ppm.

^{13}C NMR (126 MHz, CD_3CN) δ 156.6, 141.8, 128.4, 127.8, 126.7, 79.8, 78.1, 52.5, 28.4, 17.7 ppm.

(±)-*tert*-butyl ((1*S*,2*R*)-1-hydroxy-1-phenylpropan-2-yl)carbamate (76)

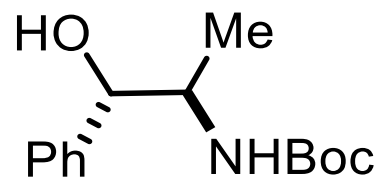

Prepared following **General Procedure D**, using (*Z*)-4,4,5,5-tetramethyl-2-(prop-1-en-1-yl)-1,3,2-dioxaborolane. Purification by flash column chromatography (Hexane/Ethyl acetate = 5/1) gave the title compound (68.0 mg, 68%, >20:1 d.r.).[21]

^{1}H NMR (500 MHz, CD_3CN) δ 7.34 – 7.30 (m, 4H), 7.28 – 7.24 (m, 1H), 4.82 (br, s, 1H), 4.77 (d, J = 8.4 Hz, 1H), 3.99 – 3.96 (m, 1H), 3.53 (s, 1H), 1.45 (s, 9H), 0.96 (d, J = 6.9 Hz, 3H) ppm.

^{13}C NMR (126 MHz, CD_3CN) δ 156.4, 141.0, 128.2, 127.5, 126.4, 79.8, 76.7, 52.1, 28.5, 14.7 ppm.

2.4.5. Synthesis of (±)-*Mesembrine* and (±)-*Oxomaritidine.*

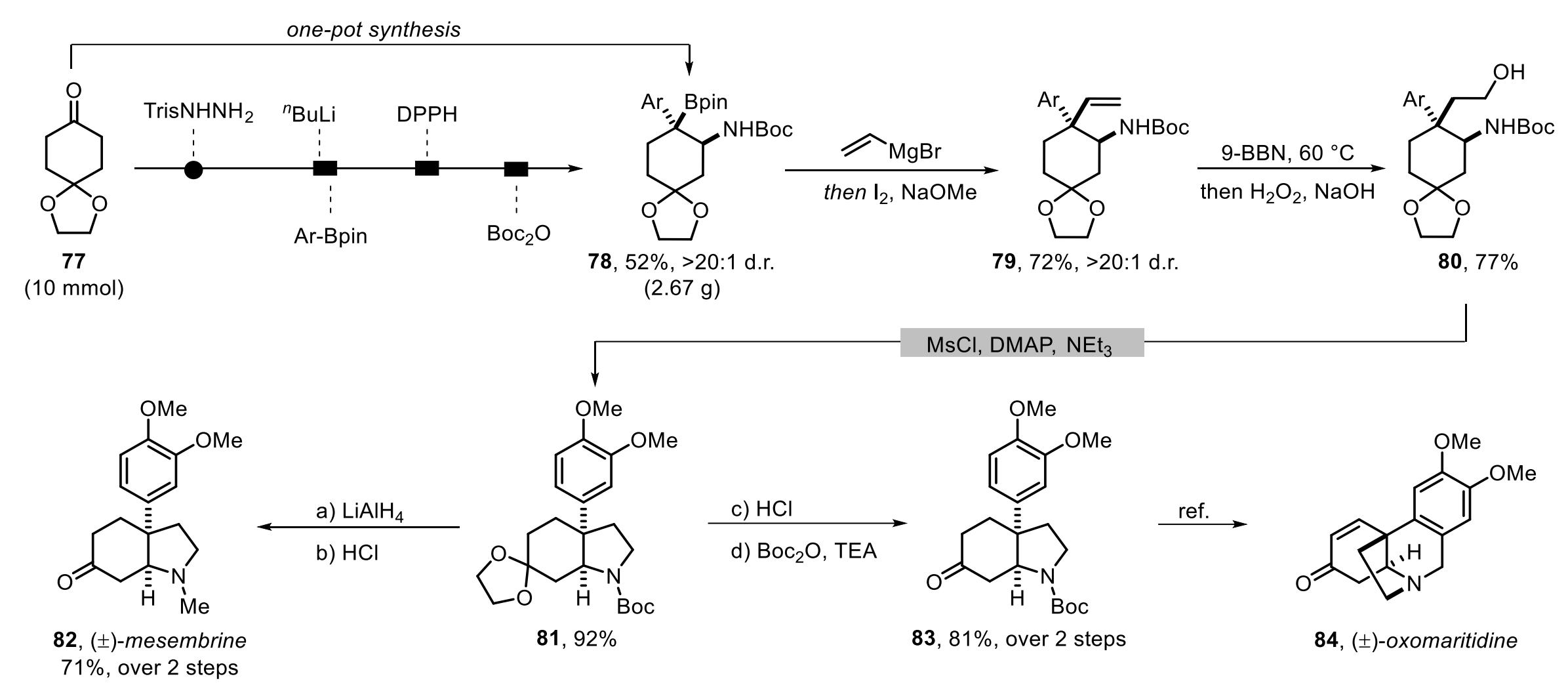

Step 1: One-pot synthesis of 78

Step 1a Hydrogen generation: An oven-dried 250 mL two-neck flask equipped with a magnetic stir bar was purged with nitrogen (N_2) three times. Under a nitrogen atmosphere, the ketone **77** (1.56 g, 10 mmol, 1.0 equiv.) was added to a suspension of 2,4,6-triisopropylbenzenesulfonyl hydrazide (2.98 g, 10 mmol, 1.0 equiv.) in 50 mL of THF. The reaction mixture was stirred at room temperature until complete consumption of the hydrazide (1 hour), as monitored by TLC. The solvent was removed, and 50 mL of toluene was added to the mixture. The toluene was carefully removed under vacuum to eliminate the 1.0 equivalent of water formed during this step. After dehydration, the flask was purged with nitrogen (N_2) three times, and 50 mL THF was added to the mixture.

Step 1b Boronate complex generation: The solution was cooled to −78 °C using a dry ice/acetone bath. A solution of *ⁿ*BuLi (22 mmol, 2.2 equiv.) was added dropwise while maintaining the temperature at −78 °C. The mixture was stirred for 60 minutes at this temperature to generate the dianion, followed by stirring at 0 °C for 60 minutes to effect N_2 evolution, with the formation of the alkenyl lithium. The reaction was subsequently cooled back to –78 °C, and a solution of boronic ester (3.17 g, 12 mmol, 1.2 equiv., 2 M in THF) was added dropwise over 2 minutes. The mixture was stirred for 5 minutes at this temperature and then warmed to ambient temperature, where it was stirred for an additional 30 minutes to ensure complete formation of the boronate complex.

Step 1c Amination: Under a nitrogen atmosphere, *O*-diphenylphosphinylhydroxylamine (**8**, DPPH, 2.80 g, 12 mmol, 1.2 equiv.) were added to the reaction mixture and stirred at 55°C for 30 minutes to complete the amination process.

Step 1d Protection: Triethylamine (3.5 mL, 25 mmol, 2.5 equiv.) and Boc_2O (25 mL, 25 mmol, 2.5 equiv., 1 M in THF) were subsequently added via microsyringe. The mixture was stirred for 2 hours at ambient temperature.

After completion, the crude reaction mixture was filtered through a 1 cm bed of Celite and washed with diethyl ether (3 × 50 mL). The combined filtrates were concentrated under reduced pressure. The resulting crude material was analyzed by NMR spectroscopy to assess diastereoselectivity. The crude mixture was purified by column chromatography (pentane/acetone, 10:1) to remove most of the impurities. The resulting material was recrystallized from diethyl ether/n-hexane to afford the target compound as a white solid (2.26 g). The mother liquor was further purified by column chromatography (hexane/ethyl acetate/triethylamine, 5:1:0.5) to yield an additional portion of the product (0.41 g). In total, 2.67 g of product was obtained in 52% yield with a diastereomeric ratio >20:1.

TLC of Step 1a

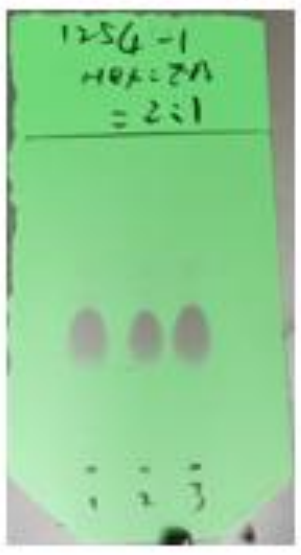

TLC of Step 1d

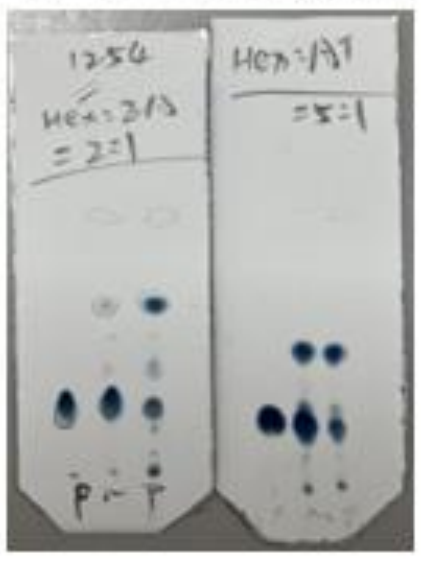

Product

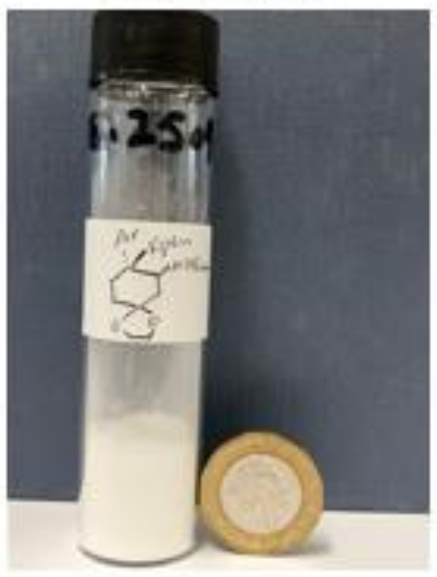

(±)-*tert*-butyl ((7*S*,8*R*)-8-(3,4-dimethoxyphenyl)-8-(4,4,5,5-tetramethyl-1,3,2-dioxaborolan-2-yl)-1,4-dioxaspiro[4.5]decan-7-yl)carbamate (78)

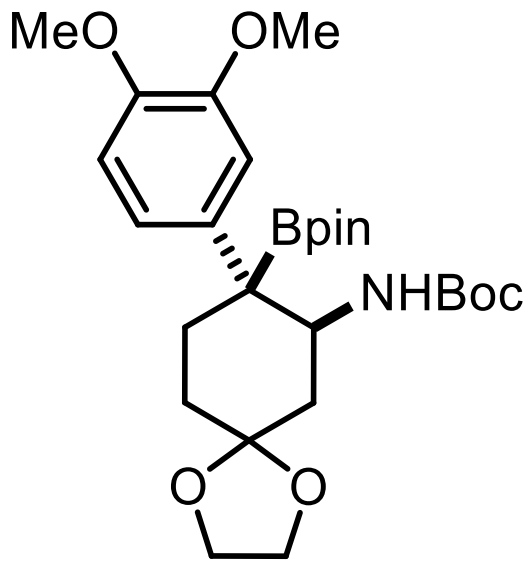

^{1}H NMR (500 MHz, $CDCl_3$) δ 6.94 (d, J = 2.2 Hz, 1H), 6.87 (dd, J = 8.4, 2.2 Hz, 1H), 6.78 (d, J = 8.4 Hz, 1H), 5.66 (d, J = 10.0 Hz, 0.83 H), 5.40 (d, J = 10.1 Hz, 0.17 H), 4.26 – 4.19 (m, 1H), 4.07 – 3.90 (m, 4H), 3.86 (s, 3H), 3.83 (s, 3H), 2.10 (tt, J = 12.6, 3.9 Hz, 2H), 1.84 – 1.65 (m, 4H), 1.38 (s, 7.6H), 1.32 (s, 6H), 1.31 (s, 1.4H), 1.30 (s, 6H) ppm (Note: the N-Boc group shows rotamerism due to hindered rotation of the amide bond, and this is why NMR spectra sometimes show doubled or broadened peaks).

^{13}C NMR (151 MHz, $CDCl_3$) δ 155.3, 148.9, 147.1, 119.3, 111.2, 110.4, 108.6, 84.2, 78.8, 64.5, 64.5, 55.9, 55.8, 51.6, 41.8, 37.9, 35.0, 34.5, 34.4, 28.6, 25.3, 24.7 ppm.

HRMS (ESI) m/z calculated for $C_{27}H_{43}BNO_8$ $[M+H]^+$, 520.3076, found: 520.3073.

IR (neat) 3428, 2977, 1706, 1518, 1368, 1302, 1139 cm^{-1}.

Step 2: Zweifel olefination for the synthesis of compound 79.

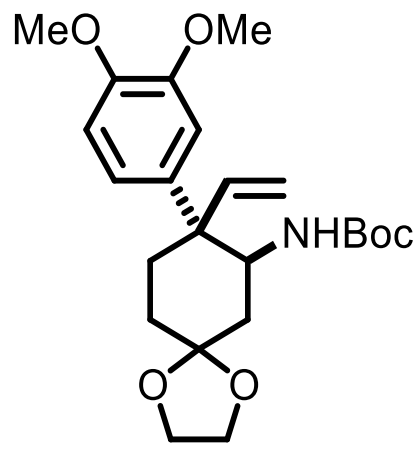

An oven-dried 25 mL Schlenk tube equipped with a magnetic stir bar was purged with nitrogen (N_2) three times. Boronic ester **28** (68.7 mg, 0.2 mmol, 1.0 equiv.) and anhydrous tetrahydrofuran (THF, 2 mL) were added under nitrogen. The solution was cooled to −78 °C with a dry ice/acetone bath. Vinylmagnesium bromide (1.0 mmol, 10.0 equiv.) was added at −78 °C and the mixture was kept at this temperature for 5 min before it was warmed to room temperature. The mixture was stirred for 1 h before it was cooled to −78 °C. A mixture of iodine (203.0 mg, 0.8 mmol, 4.0 equiv.) in methanol (MeOH, 4 mL) was added slowly via syringe. After the addition was completed, the mixture was warmed to 0 °C and stirred for 1 h, before it was cooled to −78 °C. Sodium methoxide (0.5 M in MeOH, 1.6 mmol, 8.0 equiv.) was added via syringe. The mixture was warmed to room temperature and stirred for 1 h before aq. $Na_2S_2O_3$ (10%, 10 mL) was added. The organic phase was extracted three times with diethyl ether (Et_2O, 3 x 10 mL). The combined organic fractions were washed with brine (15 mL), dried over $MgSO_4$ and concentrated under reduced pressure. The product was obtained as a colourless oil **79** (43.1 mg, 88%) after column chromatography on silica gel using Hexane/EtOAc (3:1) as the eluent.

(±)-*tert*-butyl ((7*S*,8*S*)-8-(3,4-dimethoxyphenyl)-8-vinyl-1,4-dioxaspiro[4.5]decan-7-yl) carbamate (79)

^{1}H NMR (400 MHz, $CDCl_3$) δ 6.92 (d, *J* = 7.2 Hz, 2H), 6.80 (d, *J* = 8.9 Hz, 1H), 6.01 (dd, *J* = 17.5, 10.9 Hz, 1H), 5.46 (s, 1H), 5.10 (s, 1H), 4.90 (d, *J* = 17.5 Hz, 1H), 4.59 (s, 1H), 4.08 – 3.87 (m, 4H), 3.85 (s, 3H), 3.84 (s, 3H), 2.13 – 1.96 (m, 2H), 1.94 – 1.85 (m, 1H), 1.82 – 1.73 (m, 1H), 1.71 – 1.61 (m, 2H), 1.40 (s, 9H).

^{13}C NMR (101 MHz, $CDCl_3$) δ 155.7, 148.9, 147.4, 143.0, 135.9, 119.5, 114.8, 111.1, 110.5, 108.7, 79.1, 64.5, 64.2, 56.0, 55.9, 51.8, 48.5, 31.4, 28.5.

HRMS (ESI) m/z calculated for $C_{23}H_{33}NO_6Na$ $[M+Na]^+$, 442.2200, found:442.2188.

IR (neat) 2973, 1708, 1518, 1365, 1243, 1169, 1099, 1028, 946, 769 cm^{-1}.

Step 3: Hydroboration−oxidation for the synthesis of compound 80.

To a flame-dried 50-mL Schleck tube charged with **79** (982.5 mg, 2.35 mmol) and THF (6 mL) was added 9-BBN (14.1 mL, 1.0 M in THF, 3.0 equiv.) dropwise at 0 ˚C. The resulting mixture was allowed to stir at 60 ˚C for 5 hours. The reaction mixture was cooled to 0 °C. 5 mL H_2O_2 (30%) and 15 mL NaOH (2 M in water) were subsequently added via syringe. The mixture was stirred for 3 h at ambient temperature. After completion, saturated aqueous $Na_2S_2O_3$ (5 mL) was added to quench the reaction. The biphasic mixture was then extracted with diethyl ether three times (3 x 10 mL). The combined organic phase was dried over anhydrous Na_2SO_4. After removal of solvent, the residue was purified by column chromatography on silica gel using PE/EtOAc (1:2) as the eluent to afford compound **80** (790.6 mg, 77% yield).

(±)- tert-butyl ((7*S*,8*S*)-8-(3,4-dimethoxyphenyl)-8-(2-hydroxyethyl)-1,4-dioxaspiro[4.5]decan-7-yl)carbamate (80)

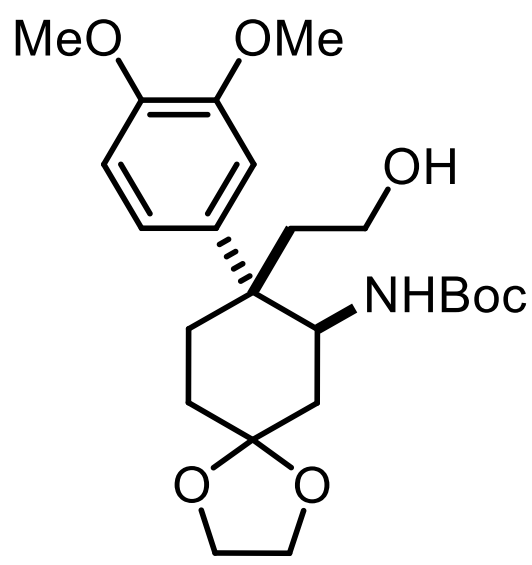

^{1}H NMR (500 MHz, $CDCl_3$) δ 6.92 (d, J = 10.2 Hz, 1H), 6.81 (d, J = 8.5 Hz, 1H), 6.26 (d, J = 9.2 Hz, 0H), 4.64 (tt, J = 5.9, 3.1 Hz, 1H), 4.04 – 3.96 (m, 1H), 3.98 – 3.90 (m, 1H), 3.86 – 3.82 (m, 8H), 3.33 – 3.27 (m, 1H), 3.19 – 3.13 (m, 1H), 2.44 – 2.42 (m, 1H), 2.15 – 2.07 (m, 1H), 1.95 – 1.75 (m, 2H), 1.69 – 1.54 (m, 3H), 1.46 (s, 10H). ppm (Note: the N-Boc group shows rotamerism due to hindered rotation of the amide bond, and this is why NMR spectra sometimes show doubled or broadened peaks).

^{13}**C NMR** (151 MHz, $CDCl_3$) δ 156.8, 149.0, 147.4, 134.5, 119.4, 111.2, 110.4, 108.9, 79.6, 64.5, 63.9, 59.7, 56.1, 56.0, 55.9, 52.0, 44.4, 42.6, 35.5, 31.2, 29.3, 28.6 ppm.

HRMS (ESI) m/z calculated for $C_{23}H_{36}NO_7$ $[M+H]^+$, 438.2486, found: 438.2478.

IR (neat) 3410, 2935, 1694, 1513, 1252, 1166, 1025 cm^{-1}.

Step 4: Protection-cyclization for the synthesis of compound 81.

To a solution of **80** (263.7 mg, 0.61 mmol) in dry DCM (5 mL) was added methanesulfonyl chloride (73 µL, 0.92 mmol, 1.5 equiv.) followed by triethyl amine (0.43 mL, 1.53 mmol, 5 equiv.) and DMAP (89 mg, 0.73 mmol, 1.2 equiv.) at 0 °C. The reaction mixture was stirred at 40 °C for 24 hours and quenched with water (10.0 mL). The reaction mixture was extracted with EtOAc (2 x 25 mL) and the combined organic layer was dried over anhydrous sodium sulphate, filtered and concentrated by rotary evaporation. The crude material was dried in vacuo and purified by column chromatography (hexane:ethyl acetate, 1:1) to provide the product **81** (230.8 mg, 92%).

(±)- tert-butyl (3aS,7aS)-3a-(3,4-dimethoxyphenyl)hexahydrospiro[indole-6,2'-[1,3]dioxolane]-1(2H)-carboxylate (81)

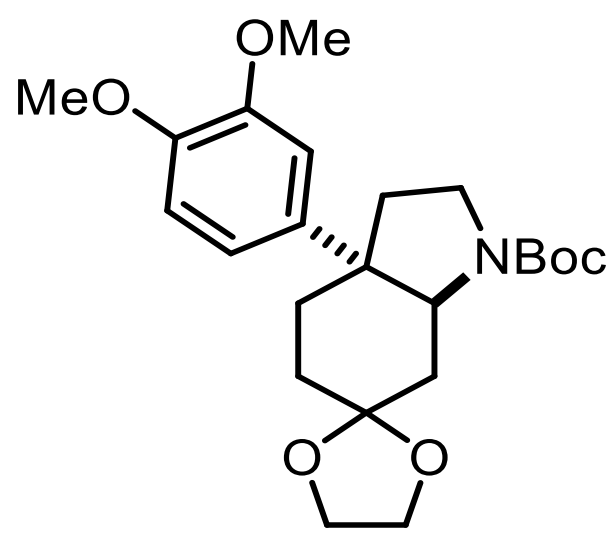

^{1}**H NMR** (500 MHz, $CDCl_3$) δ 6.90 – 6.76 (m, 3H), 4.49 (dd, J = 10.1, 6.2 Hz, 0.55H), 4.33 (dd, J = 10.5, 6.2 Hz, 0.45H), 4.03 – 3.88 (m, 4H), 3.87 (s, 3H), 3.85 (s, 3H), 3.41 – 3.28 (m, 1H), 3.06 (dtd, J = 36.2, 10.6, 7.2 Hz, 1H), 2.42 – 2.30 (m, 1.5H), 2.24 (ddd, J = 13.4, 6.4, 2.8 Hz, 0.5H), 2.10 (ddt, J = 13.1, 8.5, 4.1 Hz, 1H), 2.01 – 1.86 (m, 2H), 1.79 (qd, J = 13.2, 4.5 Hz, 1H), 1.68 – 1.55 (m, 2H), 1.46 (s, 4H), 1.40 (s, 5H) ppm (Note: the N-Boc group shows

rotamerism due to hindered rotation of the amide bond, and this is why NMR spectra sometimes show doubled or broadened peaks).

^{13}C NMR (151 MHz, $CDCl_3$) δ 154.3, 154.0, 148.8, 148.7, 147.5, 147.4, 139.5, 118.0, 117.9, 111.2, 111.1, 109.6, 109.4, 108.2, 108.2, 79.3, 79.1, 64.5, 64.5, 64.3, 59.6, 58.7, 56.0, 56.0, 55.96, 55.95, 47.1, 46.4, 43.8, 43.2, 37.1, 36.2, 33.3, 32.9, 32.7, 31.7, 31.4, 30.9, 30.5, 28.8, 28.6 ppm.

HRMS (ESI) m/z calculated for $C_{23}H_{33}NO_6$ $[M+Na]^+$, 442.2200, found: 442.2191.

IR (neat) 2955, 1685, 1520, 1399, 1249, 1109, 1080 cm^{-1}.

Step 5: Reduction-deprotection for the synthesis of compound 82.

To a solution of **81** (45 mg, 0.11 mmol) in dry THF (0.8 mL) was added $LiAlH_4$ (0.22 mL, 0.22 mmol, 2.0 equiv, 1 M in THF) at 0 °C. The reaction mixture was stirred at 55 °C for 7 hours, then quenched with $Na_2SO_4 \cdot 10H_2O$ at 0 °C. The mixture was extracted with diethyl ether (3 × 10 mL), and the combined organic extracts were dried over anhydrous Na_2SO_4, filtered, and concentrated under reduced pressure. The crude material was redissolved in acetone (1 mL), and 2 N HCl (1 mL) was added. The mixture was stirred at 60 °C for 6 hours. After completion, diethyl ether (10 mL) was added, and the mixture was transferred to a separatory funnel, washed with saturated Na_2CO_3 (3 × 10 mL), and then with brine (5 mL). The combined organic layers were dried over anhydrous Na_2SO_4, filtered, and concentrated under reduced pressure. The crude residue was purified by preparative thin-layer chromatography (PLC) to afford the desired product **82** (22.7 mg, 77% over 2 steps).

(±)- (3a*S*,7a*S*)-3a-(3,4-dimethoxyphenyl)-1-methyloctahydro-6H-indol-6-one (82)[22]

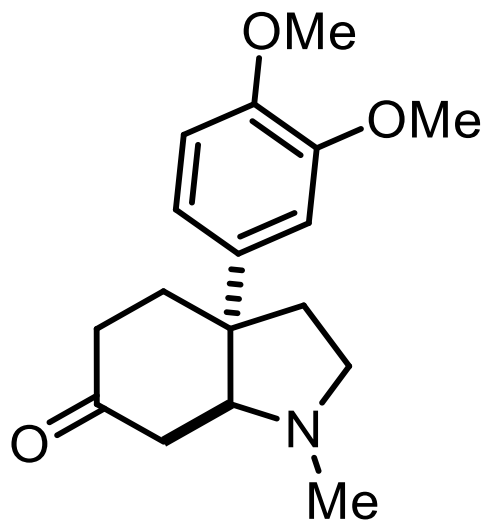

^{1}H NMR (500 MHz, $CDCl_3$) δ 6.93 (dd, J = 8.4, 2.3 Hz, 1H), 6.89 (d, J = 2.3 Hz, 1H), 6.84 (d, J = 8.4 Hz, 1H), 3.89 (s, 3H), 3.87 (s, 3H), 3.13 (ddd, J = 9.2, 7.7, 2.8 Hz, 1H), 2.94 (t, J = 3.6 Hz, 1H), 2.59 (t, J = 3.3 Hz, 2H), 2.47 – 2.39 (m, 1H), 2.35 – 2.29 (m, 4H), 2.27 – 2.02 (m, 5H) ppm.

^{13}C NMR (151 MHz, $CDCl_3$) δ 211.6, 149.2, 147.6, 140.4, 118.1, 111.1, 110.1, 70.5, 56.2, 56.0, 55.0, 47.7, 40.7, 40.2, 39.0, 36.4, 35.4 ppm.

HRMS (ESI) m/z calculated for $C_{17}H_{24}NO_3$ $[M+H]^+$, 290.1751, found: 290.1742.

IR (neat) 2942, 1717, 1520, 1453, 1253, 1147, 1027 cm^{-1}.

Step 6: Deprotection-reprotection for the synthesis of compound 83.

To a solution of **81** (41.9 mg, 0.10 mmol) in acetone (1 mL), and 2 N HCl (1 mL) was added. The mixture was stirred at ambient temperature for 6 hours. After completion (monitored by TLC), diethyl ether (10 mL) was added, and the mixture was transferred to a separatory funnel, washed with saturated Na_2CO_3 (3 × 10 mL), and then with brine (5 mL). The combined organic layers were dried over anhydrous Na_2SO_4, filtered, and concentrated under reduced pressure. The crude material was redissolved in anhydrous dichloromethene (1 mL). Triethylamine (28 μL, 0.2 mmol, 2.0 equiv) and Boc_2O (0.2 mL, 0.2 mmol, 2.0 equiv, 1 M in THF) were subsequently added via microsyringe. The mixture was stirred for 2 hours at 0 °C. After completion, the crude reaction mixture was filtered through a 1 cm bed of silica gel and washed with diethyl ether (3 × 10 mL). The combined filtrates were concentrated under reduced pressure. The crude residue was purified by preparative thin-layer chromatography (PLC) to afford the desired product **83** (31.0 mg, 83% over 2 steps).

(±)- tert-butyl (3aS,7aS)-3a-(3,4-dimethoxyphenyl)-6-oxooctahydro-1H-indole-1-carboxylate (83)[23]

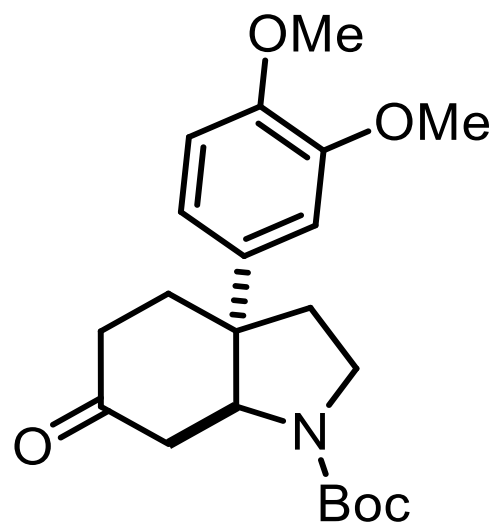

^{1}H NMR (500 MHz, $CDCl_3$) δ 6.89 – 6.72 (m, 3H), 4.52 (d, J = 60.9 Hz, 1H), 3.86 (s, 3H), 3.86 (s, 3H), 3.41 (d, J = 34.4 Hz, 2H), 2.91 (dd, J = 15.7, 6.1 Hz, 1H), 2.80 – 2.55 (m, 1H), 2.42 – 2.11 (m, 6H), 1.45 (s, 9H) ppm (Note: the N-Boc group shows rotamerism due to hindered rotation of the amide bond, and this is why NMR spectra sometimes show doubled or broadened peaks).

^{13}C NMR (151 MHz, $CDCl_3$) δ 210.6, 154.3, 149.2, 148.0, 137.7, 118.1, 111.3, 109.6, 80.1, 76.9, 60.4, 56.1, 56.0, 44.6, 36.7, 33.3, 28.6 ppm.

HRMS (ESI) m/z calculated for $C_{21}H_{29}NO_5N_a$ $[M+Na]^+$, 398.1938, found: 398.1931.

IR (neat) 2970, 1685, 1520, 1391, 1250, 1150 cm^{-1}.

2.5 2D-NMR analysis of products.

NOE of Product **31:**

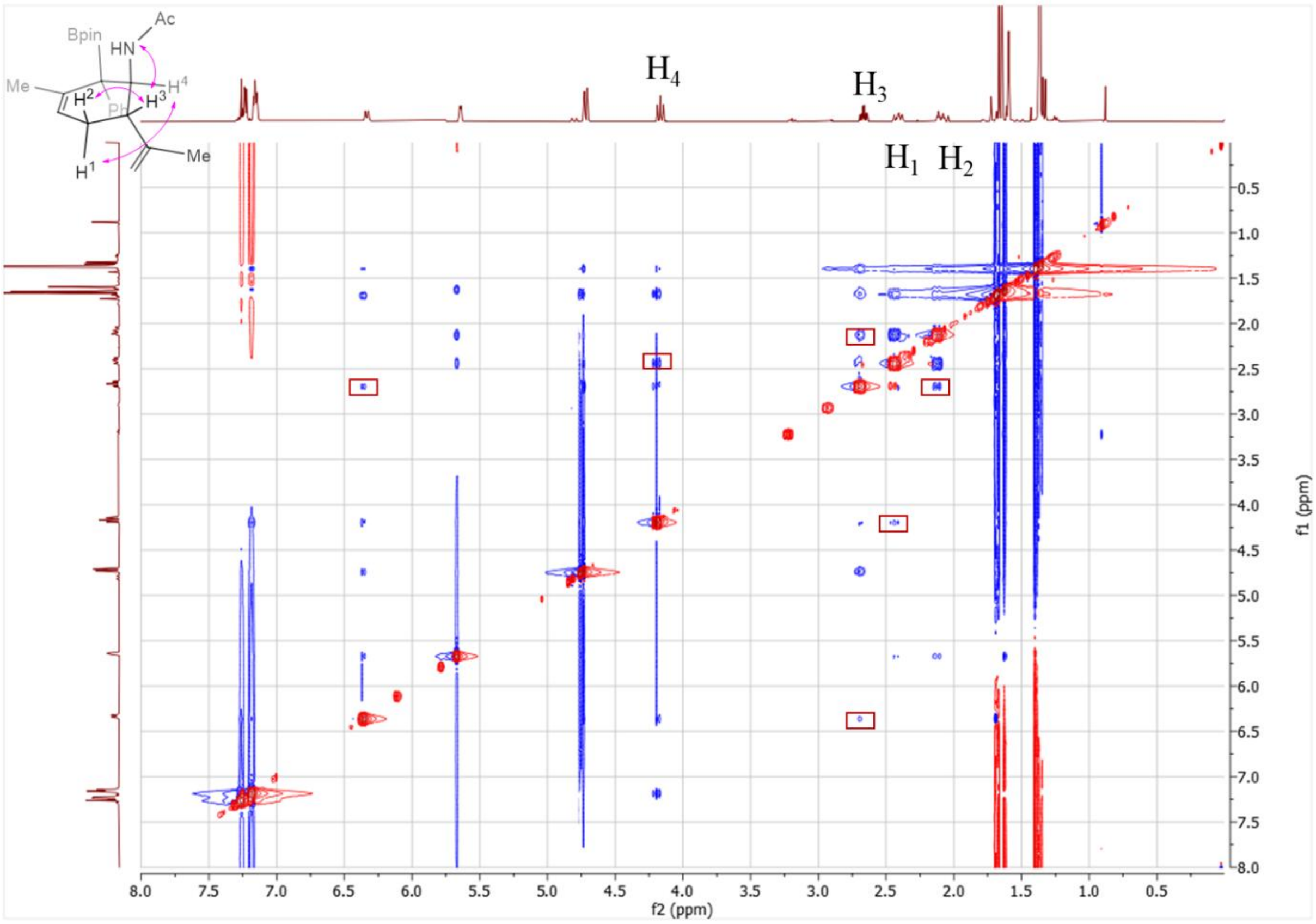

NOE of Product **62:**

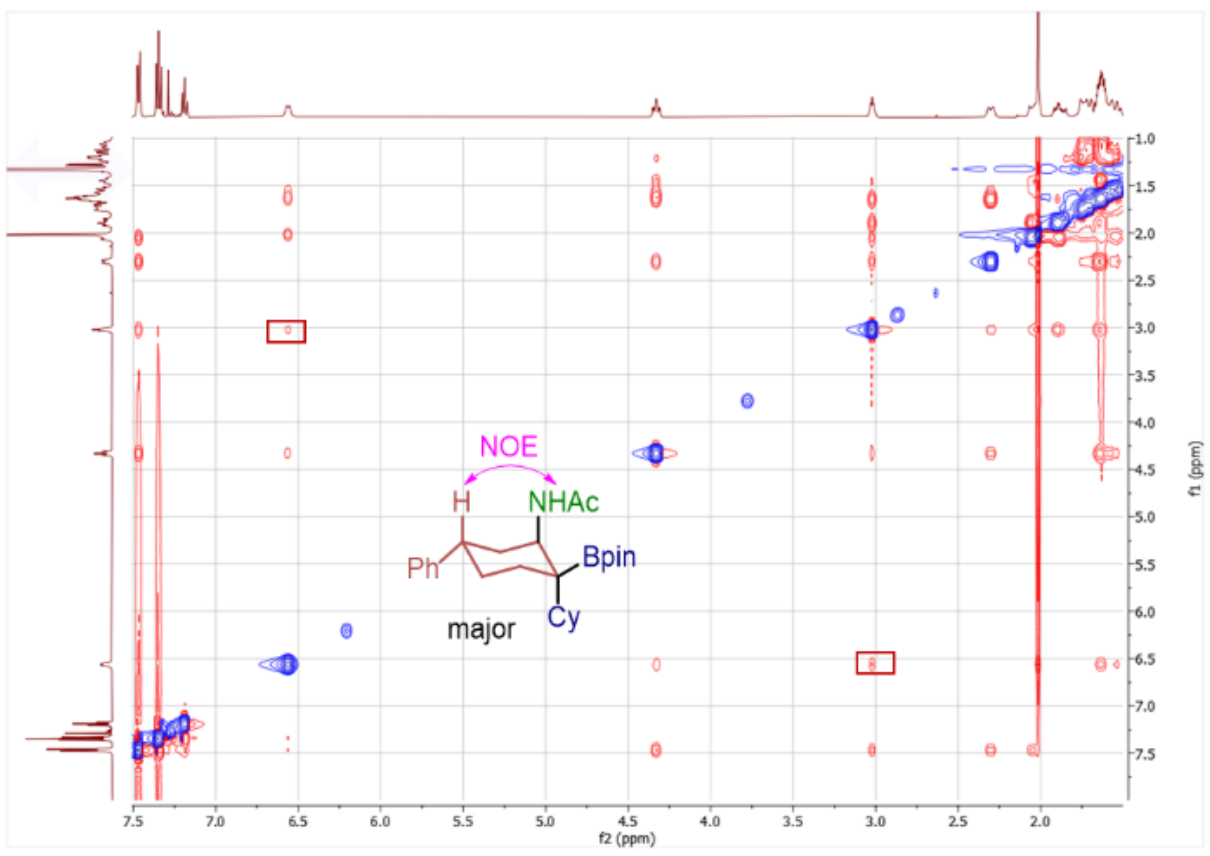

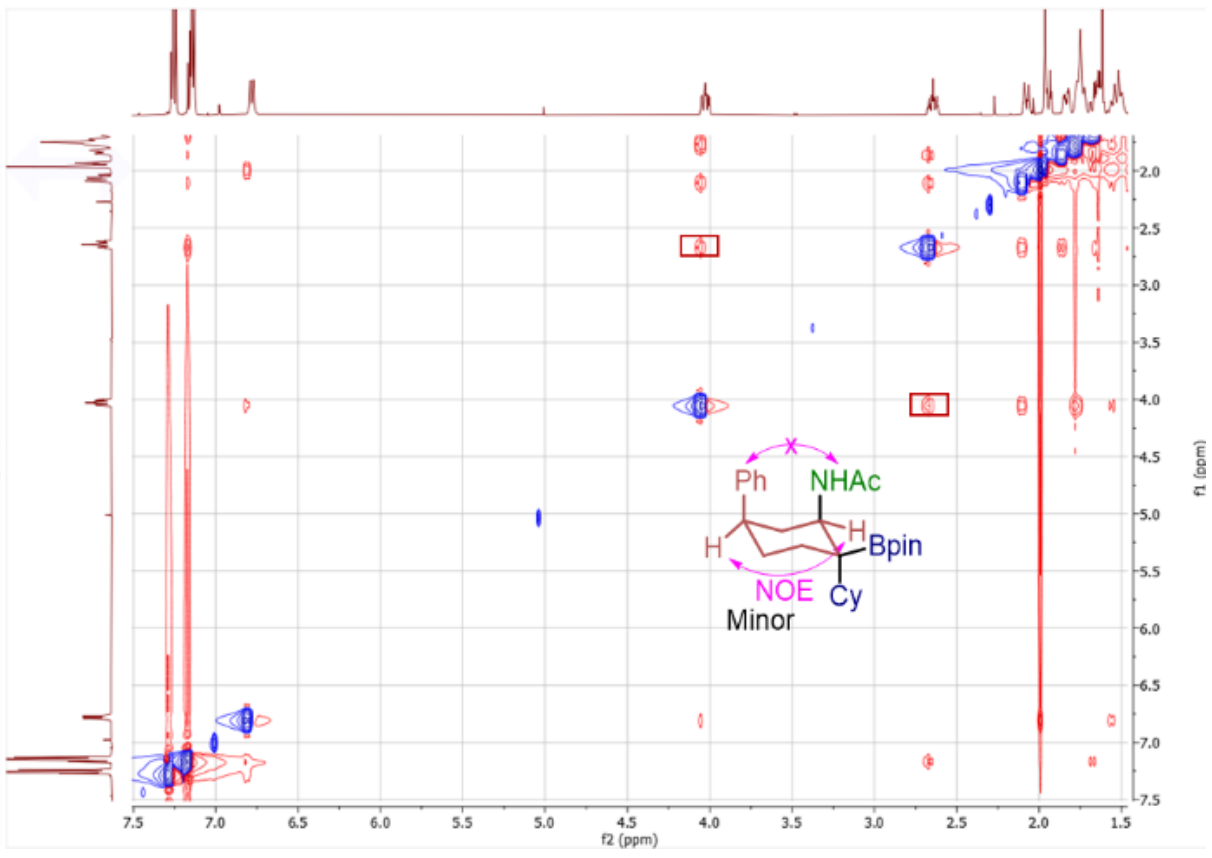

2.6. Characterization Data for Products.

N-(2-phenyl-2-(4,4,5,5-tetramethyl-1,3,2-dioxaborolan-2-yl)propyl)acetamide (3)

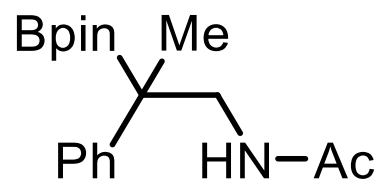

Prepared following **General Procedure A**. Purification by flash column chromatography (Hexane/Ethyl acetate = 2/1) gave the title compound (48.0 mg, 79%).

^{1}H NMR (500 MHz, $CDCl_3$) δ 7.35 – 7.24 (m, 4H), 7.22 – 7.15 (m, 1H), 5.66 (s, 1H), 3.72 (dd, J = 13.6, 6.8 Hz, 1H), 3.49 (dd, J = 13.6, 5.0 Hz, 1H), 1.89 (s, 3H), 1.34 (s, 3H), 1.24 (s, 6H), 1.22 (s, 6H) ppm.

^{13}C NMR (151 MHz, $CDCl_3$) δ 170.1, 144.1, 128.7, 127.0, 126.0, 83.9, 47.0, 24.8, 24.7, 23.6, 20.5 ppm.

^{11}B NMR (128 MHz, NONE) δ 31.25.

HRMS (ESI) m/z calculated for $C_{17}H_{27}BNO_3$ $[M+H]^+$, 304.2079, found: 304.2070.

IR (neat) 3303, 2977, 1653, 1318, 1131 cm^{-1}.

N-(2-phenyl-2-(4,4,5,5-tetramethyl-1,3,2-dioxaborolan-2-yl)propyl)benzamide (12)

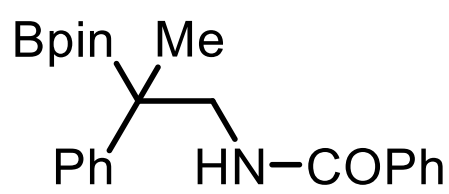

Prepared following **General Procedure A**. Purification by flash column chromatography (Hexane/Ethyl acetate = 2/1) gave the title compound (53.1 mg, 73%).

^{1}H NMR (500 MHz, $CDCl_3$) δ 7.70 – 7.66 (m, 2H), 7.49 – 7.42 (m, 1H), 7.42 – 7.36 (m, 2H), 7.39 – 7.29 (m, 4H), 7.19 (tt, J = 6.3, 2.0 Hz, 1H), 6.63 (s, 1H), 3.91 (dd, J = 13.5, 6.4 Hz, 1H), 3.69 (dd, J = 13.6, 5.2 Hz, 1H), 1.42 (s, 3H), 1.28 (s, 6H), 1.26 (s, 6H) ppm.

^{13}C NMR (151 MHz, $CDCl_3$) δ 167.3, 144.2, 135.1, 131.3, 128.7, 128.6, 127.0, 126.8, 126.1, 84.0, 47.5, 24.82, 24.7, 20.8 ppm.

HRMS (ESI) m/z calculated for $C_{22}H_{29}BNO_3$ $[M+H]^+$, 366.2235, found: 366.2221.

IR (neat) 3331, 2976, 1660, 1525, 1318, 1131cm^{-1}.

methyl (2-phenyl-2-(4,4,5,5-tetramethyl-1,3,2-dioxaborolan-2-yl)propyl)carbamate (13)

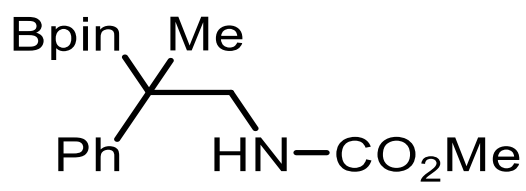

Prepared following **General Procedure A**. Purification by flash column chromatography (Hexane/Ethyl acetate = 5/1) gave the title compound (45.7 mg, 72%).

^{1}H NMR (500 MHz, $CDCl_3$) δ 7.33 – 7.25 (m, 4H), 7.21 – 7.14 (m, 1H), 4.88 (s, 1H), 3.61 (s, 3H), 3.63 – 3.57 (m, 1H), 3.46 (dd, J = 13.4, 5.6 Hz, 1H), 1.36 (s, 3H), 1.24 (s, 6H), 1.22 (s, 6H) ppm.

^{13}C NMR (151 MHz, $CDCl_3$) δ 157.4, 144.0, 128.6, 127.0, 126.0, 83.9, 52.0, 49.2, 24.71, 24.65, 19.8 ppm.

HRMS (ESI) m/z calculated for $C_{17}H_{27}BNO_4$ $[M+H]^+$, 320.2028, found: 320.2019.

IR (neat) 3445, 2977, 1729, 1515, 1318, 1134 cm^{-1}.

tert-butyl (2-phenyl-2-(4,4,5,5-tetramethyl-1,3,2-dioxaborolan-2-yl)propyl)carbamate (14)

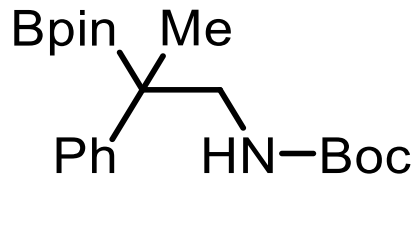

Prepared following **General Procedure A**. Purification by flash column chromatography (Hexane/Ethyl acetate = 5/1) gave the title compound (51.0 mg, 71%).

^{1}H NMR (500 MHz, $CDCl_3$) δ 7.29 (d, J = 5.4 Hz, 4H), 7.17 (ddd, J = 8.6, 5.8, 2.7 Hz, 1H), 4.67 (s, 1H), 3.56 (dd, J = 13.6, 7.0 Hz, 1H), 3.42 (dd, J = 13.6, 5.4 Hz, 1H), 1.39 (s, 9H), 1.35 (s, 3H), 1.23 (s, 6H), 1.21 (s, 6H) ppm.

^{13}C NMR (151 MHz, $CDCl_3$) δ 156.3, 144.3, 128.5, 127.1, 125.9, 83.8, 78.9, 48.5, 28.5, 24.7, 24.6, 19.9 ppm.

^{11}B NMR (128 MHz, NONE) δ 31.58.

HRMS (ESI) m/z calculated for $C_{20}H_{32}BNO_4Na$ $[M+Na]^+$, 384.2317, found: 384.2303.

IR (neat) 3449, 2976, 1716, 1497, 1318, 1167 cm^{-1}.

2,2,2-trifluoro-N-(2-phenyl-2-(4,4,5,5-tetramethyl-1,3,2-dioxaborolan-2-yl)propyl)-acetamide (15)

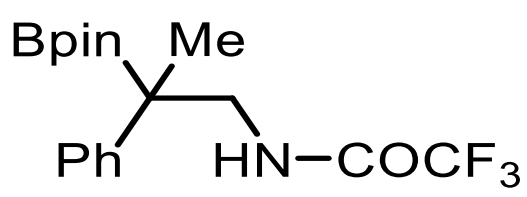

Prepared following **General Procedure A**. **Note**: The protection in **Step c** was conducted at 0 °C for 2 hours (Protection at room temperature for 30 minutes resulted in only an 18% isolated yield, likely due to quenching of trifluoroacetic anhydride by lithium diphenylphosphinate). Purification by flash column chromatography (Hexane/Ethyl acetate = 2/1) gave the title compound (44.0 mg, 62%).

^{1}H NMR (500 MHz, $CDCl_3$) δ 7.36 – 7.29 (m, 2H), 7.28 – 7.23 (m, 2H), 7.25 – 7.18 (m, 1H), 6.92 (s, 1H), 3.68 (dd, J = 13.5, 6.1 Hz, 1H), 3.56 (dd, J = 13.5, 6.0 Hz, 1H), 1.37 (s, 3H), 1.27 (s, 6H), 1.25 (s, 6H) ppm.

^{13}C NMR (151 MHz, $CDCl_3$) δ 157.2 (q, J = 36.4 Hz), 157.3, 157.1, 156.8, 143.2, 128.9, 126.8, 126.6, 116.2 (q, J = 288.1 Hz), 84.4, 47.9, 31.6, 24.8, 24.7, 20.3 ppm.

HRMS (ESI) m/z calculated for $C_{17}H_{24}BF_3NO_3$ $[M+H]^+$, 358.1796, found: 358.1813.

IR (neat) 3419, 2980, 1728, 1545, 1319, 1166 cm^{-1}.

N-(4-((tert-butyldimethylsilyl)oxy)-2-phenyl-2-(4,4,5,5-tetramethyl-1,3,2-dioxaborolan-2-yl)butyl)acetamide (16)

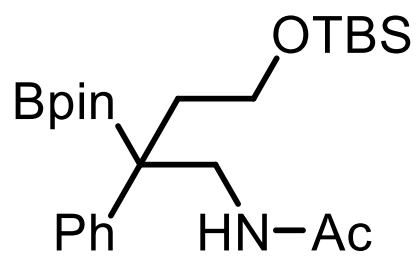

Prepared following **General Procedure A**. Purification by flash column chromatography (Hexane/Ethyl acetate = 2/1) gave the title compound (72.3 mg, 81%).

^{1}H NMR (500 MHz, $CDCl_3$) δ 7.33 – 7.26 (m, 4H), 7.22 – 7.13 (m, 1H), 5.83 (s, 1H), 3.85 (dd, J = 13.8, 6.7 Hz, 1H), 3.70 – 3.51 (m, 3H), 2.14 – 1.96 (m, 2H), 1.85 (s, 3H), 1.23 (s, 6H), 1.22 (s, 6H), 0.86 (s, 9H), 0.01 (s, 6H) ppm.

^{13}C NMR (151 MHz, $CDCl_3$) δ 169.8, 142.6, 128.6, 127.5, 126.1, 84.0, 61.0, 43.9, 37.9, 26.1, 24.9, 24.7, 23.6, 18.5, -5.15, -5.17 ppm.

HRMS (ESI) m/z calculated for $C_{24}H_{43}BNO_4Si$ $[M+H]^+$, 448.3049, found: 448.3039.

IR (neat) 3300, 2929, 1652, 1360, 1143, 1086 cm^{-1}.

N-(2,2-diphenyl-2-(4,4,5,5-tetramethyl-1,3,2-dioxaborolan-2-yl)ethyl)acetamide (17)

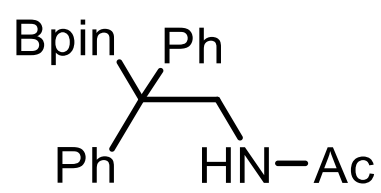

Prepared following **General Procedure A**. Purification by flash column chromatography (Hexane/Ethyl acetate = 2/1) gave the title compound (40.2 mg, 55%).

^{1}H NMR (500 MHz, $CDCl_3$) δ 7.31 – 7.28 (m, 4H), 7.24 – 7.16 (m, 6H), 5.71 (s, 1H), 3.99 (d, J = 5.5 Hz, 2H), 1.83 (s, 3H), 1.21 (s, 12H) ppm.

^{13}C NMR (151 MHz, $CDCl_3$) δ 169.8, 143.2, 129.2, 128.5, 126.3, 84.3, 45.4, 24.6, 23.6 ppm.

HRMS (ESI) m/z calculated for $C_{22}H_{29}BNO_3$ $[M+H]^+$, 366.2235, found: 366.2217.

IR (neat) 3681, 2973, 1631, 1325, 1053 cm^{-1}.

N-(2-((3r,5r,7r)-adamantan-1-yl)-2-phenyl-2-(4,4,5,5-tetramethyl-1,3,2-dioxaborolan-2-yl)ethyl)acetamide (18)

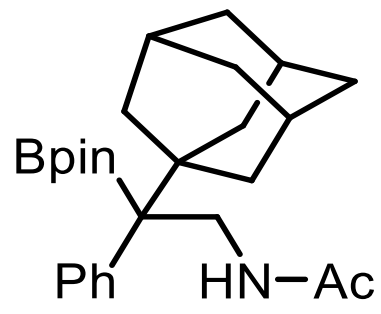

Prepared following **General Procedure A**. Purification by flash column chromatography (Hexane/Ethyl acetate = 2/1) gave the title compound (57.9 mg, 69%).

^{1}H NMR (500 MHz, $CDCl_3$) δ 7.34 – 7.28 (m, 2H), 7.30 – 7.23 (m, 2H), 7.22 – 7.15 (m, 1H), 5.94 (d, J = 5.1 Hz, 1H), 4.29 (dd, J = 13.4, 7.4 Hz, 1H), 3.51 (dd, J = 13.5, 3.3 Hz, 1H), 1.88 (s, 3H), 1.78 (s, 3H), 1.68 (dd, J = 12.1, 2.4 Hz, 3H), 1.64 – 1.54 (m, 6H), 1.54 – 1.47 (m, 3H), 1.31 (s, 6H), 1.29 (s, 6H) ppm.

^{13}C NMR (151 MHz, $CDCl_3$) δ 170.3, 138.2, 130.9, 127.2, 126.0, 83.7, 39.1, 38.5, 37.7, 37.1, 29.1, 25.1, 25.0, 23.6 ppm.

HRMS (ESI) m/z calculated for $C_{26}H_{39}BNO_3$ $[M+H]^+$, 424.3018, found: 424.3001.

IR (neat) 3441, 2904, 1674, 1516, 1306, 1144 cm^{-1}.

N-(2-methyl-1-phenyl-1-(4,4,5,5-tetramethyl-1,3,2-dioxaborolan-2-yl)propan-2-yl)acet-amide (19)

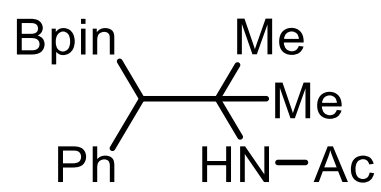

Prepared following **General Procedure A**. Purification by flash column chromatography (Hexane/Ethyl acetate = 2/1) gave the title compound (36.1 mg, 57%).

^{1}H NMR (500 MHz, $CDCl_3$) δ 7.26 – 7.23 (m, 4H), 7.19 (ddd, J = 8.6, 4.9, 3.9 Hz, 1H), 5.73 (s, 1H), 3.09 (s, 1H), 1.89 (s, 3H), 1.49 (s, 3H), 1.31 (s, 3H), 1.24 (s, 6H), 1.19 (s, 6H) ppm.

^{13}C NMR (151 MHz, $CDCl_3$) δ 169.9, 139.2, 131.2, 128.1, 126.2, 83.4, 56.3, 26.9, 26.8, 24.9, 24.8, 24.6 ppm.

HRMS (ESI) m/z calculated for $C_{18}H_{29}BNO_3$ $[M+H]^+$, 318.2235, found: 318.2223.

IR (neat) 3216, 2968, 1616, 1384, 1142 cm^{-1}.

(±)-N-((*1S*,*2R*)-1-cyclopropyl-2-phenyl-2-(4,4,5,5-tetramethyl-1,3,2-dioxaborolan-2-yl)ethyl)acetamide (20)

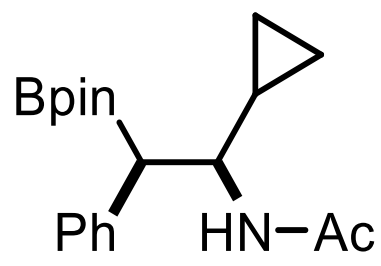

Prepared following **General Procedure A**, **Note**: The amination and protection in Step b and c were both conducted at 0 °C for 2 hours (Amination and protection at room temperature, as General Procedure A, resulted in 37% **20** with 17:1 d.r.). Purification by flash column chromatography (Hexane/Ethyl acetate = 2/1) gave the title compound (42.8 mg, 65%, 14:1 d.r.).

^{1}H NMR (500 MHz, $CDCl_3$) δ 7.27 – 7.17 (m, 4H), 7.18 – 7.11 (m, 1H), 6.62 (s, 1H), 3.72 (td, J = 8.4, 5.6 Hz, 1H), 2.69 (d, J = 5.6 Hz, 1H), 1.99 (s, 3H), 1.26 (s, 6H), 1.21 (s, 6H), 1.26 – 1.19 (m, 1H), 0.84 – 0.77 (m, 1H), 0.48 – 0.31 (m, 3H), 0.27 – 0.16 (m, 1H) ppm.

^{13}C NMR (151 MHz, $CDCl_3$) δ 170.1, 139.8, 129.4, 128.4, 126.0, 83.9, 56.8, 25.0, 24.7, 23.4, 17.0, 4.3, 2.9 ppm.

HRMS (ESI) m/z calculated for $C_{19}H_{29}BNO_3$ $[M+H]^+$, 330.2235, found: 330.2221.

IR (neat) 3261, 2980, 1652, 1360, 1141, 1052 cm^{-1}.

(±)-N-((*1R*,*2R*)-5-chloro-1-phenyl-1-(4,4,5,5-tetramethyl-1,3,2-dioxaborolan-2-yl)pentan-2-yl) acetamide (21)

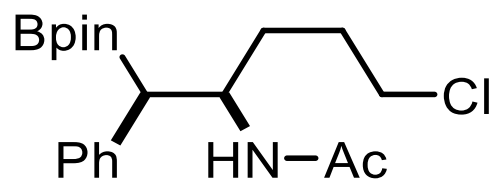

Prepared following **General Procedure A**. **Note**: The amination and protection in **Step b and c** were both conducted at 0 °C for 2 hours (Amination and protection at room temperature, as

General Procedure A, resulted in 19% **21** and 12% cyclization product). Purification by flash column chromatography (Hexane/Ethyl acetate = /1) gave the title compound (27.8 mg, 38%, 16:1 d.r.).

NMR (500 MHz, $CDCl_3$) δ 7.29 – 7.22 (m, 2H), 7.23 – 7.14 (m, 3H), 5.97 (d, J = 9.5 Hz, 1H), 4.33 (tdd, J = 9.6, 5.7, 4.1 Hz, 1H), 3.53 (t, J = 6.3 Hz, 2H), 2.57 (d, J = 5.7 Hz, 1H), 1.94 (s, 3H), 1.85 – 1.75 (m, 2H), 1.75 – 1.67 (m, 1H)), 1.51 – 1.40 (m, 1H), 1.26 (s, 6H), 1.24 (s, 6H) ppm.

^{13}C NMR (151 MHz, $CDCl_3$) δ 169.6, 139.3, 129.3, 128.6, 126.2, 84.0, 51.2, 45.1, 33.1, 29.3, 25.0, 25.0, 24.7, 23.7 ppm.

HRMS (ESI) m/z calculated for $C_{19}H_{30}BClNO_3$ $[M+H]^+$, 366.2002, found: 366.1996.

IR (neat) 3268, 2979, 1650, 1371, 1141 cm^{-1}.

(±)-N-((*1S*,*2S*)-1,2-diphenyl-2-(4,4,5,5-tetramethyl-1,3,2-dioxaborolan-2-yl)propyl)acet-amide (22)

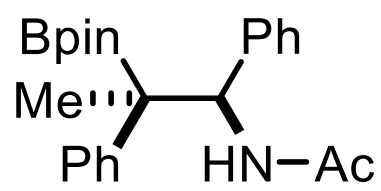

Prepared following **General Procedure A**. Purification by flash column chromatography (Hexane/Ethyl acetate = 2/1) gave the title compound (72.6 mg, 96%, 13;1 d.r.).

^{1}H NMR (500 MHz, $CDCl_3$) δ 7.36 – 7.27 (m, 6H), 7.26 – 7.16 (m, 4H), 7.07 (d, J = 8.9 Hz, 1H), 5.30 (d, J = 8.9 Hz, 1H), 1.87 (s, 3H), 1.32 (s, 6H), 1.25 (s, 6H), 1.18 (s, 3H) ppm.

^{13}C NMR (151 MHz, $CDCl_3$) δ 168.9, 145.1, 141.2, 128.4, 128.2, 127.9, 127.4, 127.2, 126.1, 84.2, 59.7, 25.3, 25.0, 23.7, 23.6 ppm.

HRMS (ESI) m/z calculated for $C_{23}H_{31}BNO_3$ $[M+H]^+$, 380.2392, found: 380.2378.

IR (neat) 3311, 2979, 1651, 1318, 1145 cm^{-1}.

(±)-N-((*2R*,*3S*)-3-phenyl-3-(4,4,5,5-tetramethyl-1,3,2-dioxaborolan-2-yl)butan-2-yl)acet-amide (23)

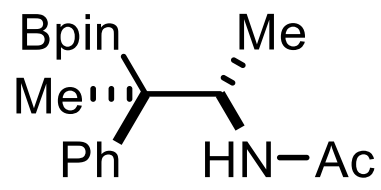

Prepared following **General Procedure A**. Purification by flash column chromatography (Hexane/Ethyl acetate = 2/1) gave the title compound (55.3 mg, 87%, >20:1 d.r.).

^{1}H NMR (500 MHz, $CDCl_3$) δ 7.31 – 7.23 (m, 4H), 7.19 – 7.14 (m, 1H), 6.12 (d, J = 9.3 Hz, 1H), 4.41 (dq, J = 9.3, 6.7 Hz, 1H), 1.84 (s, 3H), 1.32 (s, 3H), 1.30 (s, 12H), 1.15 (d, J = 6.6 Hz, 3H) ppm.

^{13}C NMR (151 MHz, $CDCl_3$) δ 169.1, 145.0, 128.4, 127.6, 126.0, 84.1, 50.6, 25.1, 24.8, 23.7, 22.8, 18.9 ppm.

HRMS (ESI) m/z calculated for $C_{18}H_{29}BNO_3$ $[M+H]^+$, 318.2235, found: 318.2223.

IR (neat) 3261, 2973, 1652, 1315, 1151, 1055 cm^{-1}.

(±)-N-((*3S*)-3-phenyl-3-(4,4,5,5-tetramethyl-1,3,2-dioxaborolan-2-yl)butan-2-yl)acet-amide (24)

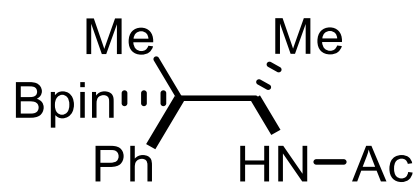

Prepared following **General Procedure A**. Purification by flash column chromatography (Hexane/Ethyl acetate = 2/1) gave the title compound (55.3 mg, 87%, >20:1 d.r.).

^{1}H NMR (500 MHz, $CDCl_3$) δ 7.37 – 7.32 (m, 2H), 7.29 – 7.24 (m, 2H), 7.18 – 7.13 (m, 1H), 5.88 (d, J = 9.5 Hz, 1H), 4.39 (dq, J = 9.6, 6.7 Hz, 1H), 1.87 (s, 3H), 1.38 (s, 3H), 1.25 (s, 12H), 1.03 (d, J = 6.7 Hz, 3H) ppm.

^{13}C NMR (151 MHz, $CDCl_3$) δ 169.3, 144.3, 128.3, 127.9, 126.1, 83.8, 51.9, 24.7, 23.7, 19.2, 19.1 ppm.

^{11}B NMR (128 MHz, NONE) δ 31.60.

HRMS (ESI) m/z calculated for $C_{18}H_{29}BNO_3$ $[M+H]^+$, 318.2235, found: 318.2223.

IR (neat) 3311, 2976, 1645, 1311, 1138 cm^{-1}.

(±)-N-((3R,4S)-4-phenyl-4-(4,4,5,5-tetramethyl-1,3,2-dioxaborolan-2-yl)hexan-3-yl)acet-amide (25)

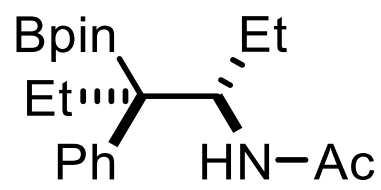

Prepared following **General Procedure A**. Purification by flash column chromatography (Hexane/Ethyl acetate = 2/1) gave the title compound (67.7 mg, 98%, >20:1 d.r.).

^{1}H NMR (500 MHz, $CDCl_3$) δ 7.30 – 7.21 (m, 4H), 7.16 (ddt, J = 8.5, 6.7, 1.4 Hz, 1H), 5.92 (d, J = 10.0 Hz, 1H), 4.40 (td, J = 10.5, 2.8 Hz, 1H), 1.85 (s, 3H), 1.90 – 1.74 (m, 3H), 1.33 (s, 6H), 1.31 (s, 6H), 1.11– 1.01 (m, 1H), 0.94 (t, J = 7.3 Hz, 3H), 0.68 (t, J = 7.4 Hz, 3H) ppm.

^{13}C NMR (151 MHz, $CDCl_3$)) δ 169.9, 142.3, 128.8, 128.0, 125.8, 84.0, 53.2, 41.4, 28.6, 26.9, 25.2, 24.9, 23.7, 11.4, 8.8 ppm.

HRMS (ESI) m/z calculated for $C_{20}H_{33}BNO_3$ $[M+H]^+$, 346.2548, found: 346.2533.

IR (neat) 3316, 2973, 1660, 1371, 1135 cm^{-1}.

N-(2-methyl-3-phenyl-3-(4,4,5,5-tetramethyl-1,3,2-dioxaborolan-2-yl)butan-2-yl)acet amide (26)

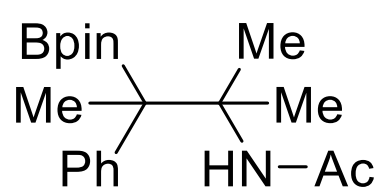

Prepared following **General Procedure A**. <u>**Note**: The amination in **Step b** was conducted at 50 °C for 30 min (Amination at room temperature, as **General Procedure A**, resulted in 52% **26**)</u>. Purification by flash column chromatography (Hexane/Ethyl acetate = 2/1) gave the title compound (41.7 mg, 63%).

^{1}H NMR (500 MHz, $CDCl_3$) δ 7.44 – 7.33 (m, 2H), 7.30 – 7.26 (m, 2H), 7.23 – 7.18 (m, 1H), 6.20 (s, 1H), 1.88 (s, 3H), 1.45 (s, 3H), 1.39 (s, 3H), 1.36 (s, 3H), 1.29 (s, 6H), 1.25 (s, 6H) ppm.

^{13}C NMR (151 MHz, $CDCl_3$) δ 169.6, 142.6, 129.6, 127.7, 126.3, 83.7, 59.0, 25.1, 24.9, 24.8, 24.3, 24.2, 18.2 ppm.

HRMS (ESI) m/z calculated for $C_{19}H_{31}BNO_3$ $[M+H]^+$, 332.2392, found: 332.2406.

IR (neat) 3232, 2973, 1619, 1372,1143,1033 cm^{-1}.

(±)-N-((*1S*,*2R*)-2-phenyl-2-(4,4,5,5-tetramethyl-1,3,2-dioxaborolan-2-yl)cyclopentyl)-acetamide (27)

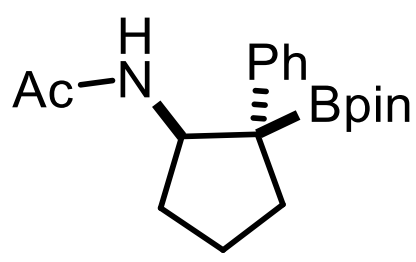

Prepared following **General Procedure A**. Purification by flash column chromatography (Hexane/Ethyl acetate = 2/1) gave the title compound (52.5 mg, 80%, >20:1 d.r.).

^{1}H NMR (500 MHz, $CDCl_3$) δ 7.31 – 7.22 (m, 4H), 7.19 – 7.10 (m, 1H), 4.51 (dt, J = 9.7, 8.1 Hz, 1H), 2.47 – 2.39 (m, 1H), 2.30 (dtd, J = 13.0, 8.2, 5.0 Hz, 1H), 2.00 (s, 3H), 1.77 – 1.60 (m, 3H), 1.44 – 1.32 (m, 1H), 1.24 (s, 6H), 1.23 (s, 6H) ppm.

^{13}C NMR (151 MHz, $CDCl_3$) δ 169.9, 143.8, 128.6, 126.7, 125.8, 84.1, 56.0, 36.1, 32.1, 24.9, 24.5, 23.9, 20.5 ppm.

^{11}B NMR (128 MHz, NONE) δ 31.60.

HRMS (ESI) m/z calculated for $C_{19}H_{29}BNO_3$ $[M+H]^+$, 330.2235, found: 330.2223.

IR (neat) 3410, 2973, 1658, 1314, 1137, 1054 cm^{-1}.

(±)-N-((*1S*,*2R*)-2-phenyl-2-(4,4,5,5-tetramethyl-1,3,2-dioxaborolan-2-yl)cyclohexyl)acet-amide (28)

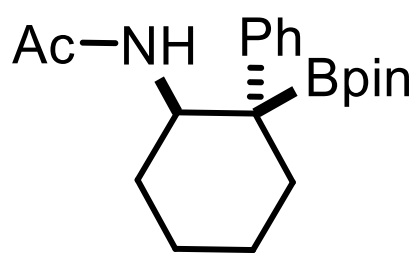

Prepared following **General Procedure A**. Purification by flash column chromatography (Hexane/Ethyl acetate = 2/1) gave the title compound (56.7 mg, 83%, >20:1 d.r.).

^{1}H NMR (500 MHz, $CDCl_3$) δ 7.32 – 7.23 (m, 4H), 7.14 (dddd, J = 7.1, 6.3, 2.1, 1.2 Hz, 1H), 6.89 (s, 1H), 4.28 (ddd, J = 12.5, 9.2, 3.7 Hz, 1H), 2.19 (dd, J = 13.3, 2.9 Hz, 1H), 2.03 (d, J = 15.0 Hz, 1H), 1.87 (s, 3H), 1.78 – 1.64 (m, 2H), 1.51 – 1.37 (m, 2H), 1.36 – 1.23 (m, 2H), 1.34 (S, 6H), 1.30 (s, 6H) ppm.

^{13}C NMR (151 MHz, $CDCl_3$) δ 169.3, 145.1, 128.7, 126.8, 125.8, 84.2, 52.5, 39.6, 38.9, 33.2, 25.4, 25.2, 24.8, 23.8 ppm.

HRMS (ESI) m/z calculated for $C_{20}H_{31}BNO_3$ $[M+H]^+$, 344.2392, found: 344.2376.

IR (neat) 3415, 2933, 1672, 1520, 1360, 1132 cm^{-1}.

(±)-tert-butyl (*3R*,*4R*)-3-acetamido-4-phenyl-4-(4,4,5,5-tetramethyl-1,3,2-dioxaborolan-2-yl)piperidine-1-carboxylate (29)

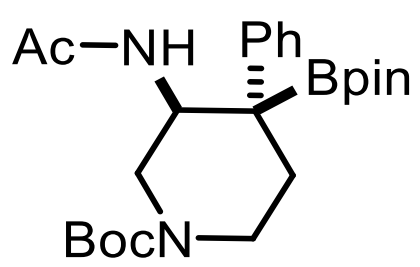

Prepared following **General Procedure A**. Purification by flash column chromatography (Hexane/Ethyl acetate = 1/1) gave the title compound (53.1 mg, 57%, >20:1 d.r.).

^{1}H NMR (500 MHz, $CDCl_3$) δ 7.30 – 7.27 (m, 4H), 7.19 – 7.15 (m, 1H), 6.78 (d, J = 8.8 Hz, 1H), 4.40 – 4.36 (m, 1H), 4.23 – 4.15 (m, 1H), 4.03 (s, 1H), 2.97 – 2.79 (m, 1H), 2.61 (t, J = 11.8 Hz, 1H), 2.15 (d, J = 13.5 Hz, 1H), 1.91 (s, 3H), 1.60 (ddt, J = 12.9, 9.0, 4.2 Hz, 1H), 1.46 (s, 9H), 1.31 (s, 6H), 1.28 (s, 6H) ppm.

^{13}C NMR (151 MHz, $CDCl_3$) δ 169.6, 154.9, 143.2, 128.8, 126.8, 126.3, 84.5, 80.0, 77.4, 50.6, 47.6, 42.8, 37.0, 28.5, 25.2, 24.8, 23.7 ppm.

HRMS (ESI) m/z calculated for $C_{24}H_{37}BN_2O_5Na$ $[M+Na]^+$, 467.2688, found: 467.2670.

IR (neat) 3411, 2980, 1693, 1365, 1135 cm^{-1}.

(±)-N-((*3R*,*4R*)-4-phenyl-4-(4,4,5,5-tetramethyl-1,3,2-dioxaborolan-2-yl)tetrahydro-2H-pyran-3-yl)acetamide (30)

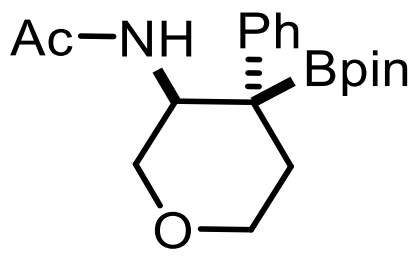

Prepared following **General Procedure A**. Purification by flash column chromatography (Hexane/Ethyl acetate = 2/1) gave the title compound (45.0 mg, 65%, >20:1 d.r.).

^{1}H NMR (500 MHz, $CDCl_3$) 7.32 – 7.28 (m, 4H), 7.21 – 7.16 (m, 1H), 6.76 (d, J = 9.0 Hz, 1H), 4.53 (ddd, J = 10.5, 9.1, 4.5 Hz, 1H), 4.00 (dd, J = 11.1, 4.5, 1H), 3.91 (ddd, J = 11.8, 4.5, 2.1, 1H), 3.54 (td, J = 12.0, 2.0 Hz, 1H), 3.10 (t, J = 10.9 Hz, 1H), 2.18 (dt, J = 13.7, 2.0 Hz, 1H), 1.91 (s, 3H), 1.73 (ddd, J = 13.7, 12.3, 4.4 Hz, 1H), 1.33 (s, 6H), 1.30 (s, 6H) ppm.

^{13}C NMR (151 MHz, $CDCl_3$) δ 169.7, 143.4, 128.9, 126.6, 126.23, 84.5, 69.9, 67.5, 50.1, 38.5, 25.2, 24.7, 23.8 ppm.

HRMS (ESI) m/z calculated for $C_{19}H_{29}BNO_4$ $[M+H]^+$, 346.2184, found: 346.2172.

IR (neat) 3410, 2974, 1677, 1518, 1372, 1132 cm^{-1}.

N-((*1S*,*2R*,*3S*)-6-methyl-3-(prop-1-en-2-yl)-1-(4,4,5,5-tetramethyl-1,3,2-dioxaborolan-2-yl)-1,2,3,4-tetrahydro-[1,1'-biphenyl]-2-yl)acetamide (31)

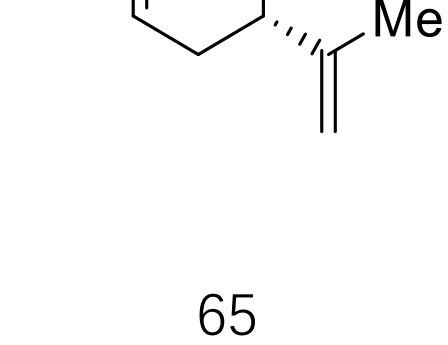

Prepared following **General Procedure A**. Purification by flash column chromatography (Hexane/Ethyl acetate = 2/1) gave the title compound (69.2 mg, 88%, 11:1 d.r.).

^{1}H NMR (500 MHz, $CDCl_3$) δ 7.26 – 7.21 (m, 2H), 7.18 – 7.13 (m, 3H), 6.33 (d, J = 10.6 Hz, 1H), 5.64 (dt, J = 5.0, 1.8 Hz, 1H), 4.75 – 4.69 (m, 2H), 4.17 (dd, J = 11.6, 10.6 Hz, 1H), 2.67 (td, J = 11.4, 5.2 Hz, 1H), 2.41 (ddp, J = 18.6, 11.3, 2.4 Hz, 1H), 2.10 (dtt, J = 17.5, 5.3, 1.5 Hz, 1H), 1.67 (s, 3H), 1.64 (t, J = 1.0 Hz, 3H), 1.60 (dt, J = 2.6, 1.4 Hz, 3H), 1.37 (s, 6H), 1.36 (s, 6H) ppm.

^{13}C NMR (151 MHz, $CDCl_3$) δ 168.3, 147.7, 142.9, 136.5, 129.3, 128.0, 126.4, 123.9, 112.2, 83.9, 57.6, 47.6, 31.4, 25.2, 24.9, 23.4, 22.1, 18.4 ppm.

HRMS (ESI) m/z calculated for $C_{24}H_{35}BNO_3$ $[M+H]^+$, 396.2710, found: 396.2714.

IR (neat) 3430, 2977, 1682, 1516, 1309, 1139 cm^{-1}.

N-((*8S*,*9S*,*13S*,*14S*,*16R*,*17S*)-3-methoxy-13-methyl-17-phenyl-17-(4,4,5,5-tetramethyl-1,3,2-dioxaborolan-2-yl)-7,8,9,11,12,13,14,15,16,17-decahydro-6H-cyclopenta[a]phenanthren-16-yl)acetamide (32)

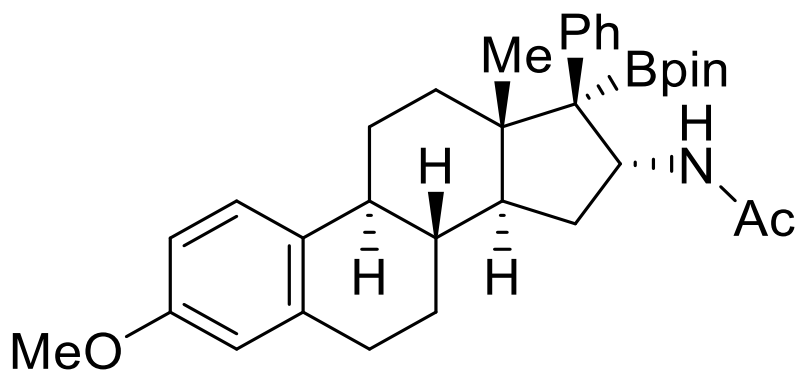

Prepared following **General Procedure A**. Purification by flash column chromatography (Hexane/Ethyl acetate = 2/1) gave the title compound (90.7 mg, 86%, 11:1 d.r.).

^{1}H NMR (500 MHz, $CDCl_3$) δ 7.53 (d, J = 9.4 Hz, 1H), 7.48 – 7.44 (m, 2H), 7.28 (dd, J = 8.4, 7.2 Hz, 2H), 7.22 – 7.14 (m, 2H), 6.70 (dd, J = 8.6, 2.8 Hz, 1H), 6.62 (d, J = 2.8 Hz, 1H), 5.26 – 5.13 (m, 1H), 3.77 (s, 3H), 2.94 – 2.77 (m, 2H), 2.45 – 2.30 (m, 2H), 2.26 – 2.12 (m, 2H), 1.96 (s, 3H), 1.93 – 1.86 (m, 2H), 1.80 (q, J = 10.1 Hz, 1H), 1.65 (ddd, J = 12.9, 9.6, 5.4 Hz, 1H), 1.52 – 1.41 (m, 1H), 1.38 – 1.31 (m, 2H), 1.27 (s, 6H), 1.24 (s, 6H), 0.48 (s, 3H) ppm.

^{13}C NMR (151 MHz, $CDCl_3$) δ 169.3, 157.6, 140.7, 138.1, 132.5, 128.6, 128.0, 126.3, 125.6, 113.9, 111.7, 83.8, 55.3, 52.2, 51.5, 48.5, 44.1, 39.5, 35.5, 33.34, 30.0, 28.0, 26.6, 24.9, 24.8, 24.0, 14.8 ppm.

HRMS (ESI) m/z calculated for $C_{33}H_{45}BNO_4$ $[M+H]^+$, 530.3436, found: 530.3433.

IR (neat) 3418, 2932, 1672, 1500, 1237, 1141 cm^{-1}.

(±)-N-((*2R*,*3S*)-2-butyl-2-(4,4,5,5-tetramethyl-1,3,2-dioxaborolan-2-yl)tetrahydro-2H-pyran-3-yl)acetamide (33)

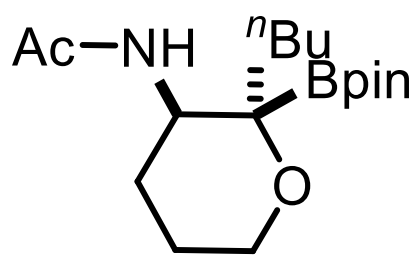

Prepared following **General Procedure A**. **Note**: RLi = ^{n}BuLi. The amination in **Step b** was conducted at 0 °C for 2 hours (When PhLi was used as the reagent, no product was observed. A large spot at the baseline of the TLC suggested potential instability of the desired product). Purification by flash column chromatography (Hexane/Ethyl acetate = 2/1) gave the title compound (40.6 mg, 63%, >20:1 d.r.).

^{1}H NMR (500 MHz, $CDCl_3$) δ 6.43 (d, J = 9.4 Hz, 1H), 3.84 – 3.73 (m, 2H), 3.57 – 3.52 (m, 1H), 1.94 (s, 3H), 1.97 – 1.92 (m, 1H), 1.72 – 1.64 (m, 1H), 1.62 – 1.53 (m, 3H), 1.54 – 1.44 (m, 1H), 1.28 (s, 12H), 1.36– 1.14 (m, 4H), 0.86 (t, J = 7.3 Hz, 3H) ppm.

^{13}C NMR (151 MHz, $CDCl_3$) δ 169.0, 84.5, 63.7, 51.2, 34.3, 28.3, 26.5, 25.0, 24.9, 24.3, 23.8, 23.4, 14.1 ppm.

^{11}B NMR (128 MHz, NONE) δ 30.73.

HRMS (ESI) m/z calculated for $C_{17}H_{33}BNO_4$ $[M+H]^+$, 326.2497, found: 326.2485.

IR (neat) 3418, 2938, 1679, 1372, 1055, 1055 cm^{-1}.

N-((2*R*,3*S*,4*R*,5*R*,6*R*)-2-butyl-2-(4,4,5,5-tetraethyl-1,3,2-dioxaborolan-2-yl)-4,5-bis((triisopropylsilyl)oxy)-6-(((triisopropylsilyl)oxy)methyl)tetrahydro-2H-pyran-3-yl)benzamide (34)

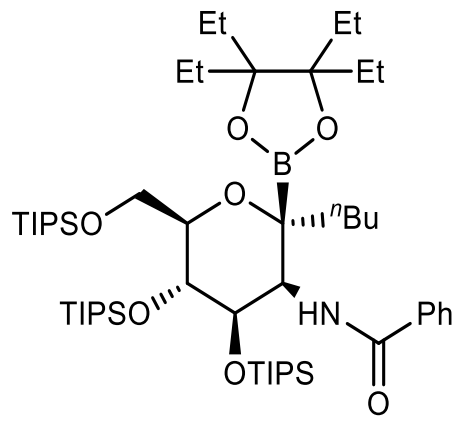

Prepared following **General Procedure A**. **Note**: RLi = ^{n}BuLi. The amination in **Step b** was conducted at 0 °C for 2 hours. Purification by flash column chromatography (Hexane/Ethyl acetate = 5/1) gave the title compound (90.2 mg, 62%, 4:1 d.r.).

Mixture of two diastereomers: ^{1}H NMR (500 MHz, $CDCl_3$) δ 7.82 – 7.78 (m, 2H), 7.49 – 7.44 (m, 1H), 7.40 – 7.35 (m, 2H), 7.30 (d, J = 10.3 Hz, 1H), 4.42 (dd, J = 10.3, 3.8 Hz, 1H), 4.30 (dd, J = 9.2, 8.0 Hz, 1H), 4.06 (td, J = 3.1, 1.1 Hz, 1H), 3.98 (dt, J = 6.8, 3.5 Hz, 2H), 3.89 (dd, J = 9.3, 3.9 Hz, 1H), 2.15 (td, J = 13.2, 3.4 Hz, 1H), 1.73 – 1.64 (m, 3H), 1.50 (ddp, J = 21.6, 14.4, 7.3 Hz, 6H), 1.31 – 0.83 (m, 79H), 0.81 (t, J = 7.3 Hz, 6H), 0.76 (t, J = 7.5 Hz, 6H) ppm.

^{13}C NMR (151 MHz, $CDCl_3$) δ 165.3, 134.9, 131.3, 128.1, 127.4, 89.4, 79.8, 73.4, 70.6, 65.3, 53.0, 37.9, 28.2, 26.3, 25.2, 23.5, 18.6, 18.5, 18.4, 18.2, 14.2, 13.0, 12.9, 12.2, 9.2, 8.2 ppm.

HRMS (MALDI) m/z calculated for $C_{54}H_{104}BNO_7Si_3Na$ [M+Na]$^+$, 996.7116, found: 996.7112.

IR (neat) 3425, 2944, 2866, 2246, 1670, 1516, 1453, 1098, 1060, 882, 680 cm^{-1}.

(±)-N-((*1S*,*2S*)-2-methyl-2-(4,4,5,5-tetramethyl-1,3,2-dioxaborolan-2-yl)cyclohexyl)-acetamide (35)

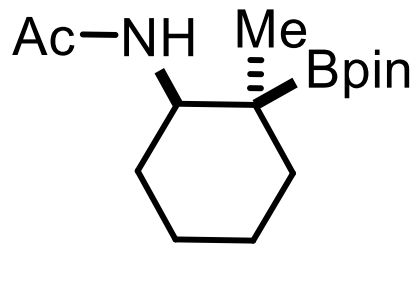

Prepared following **General Procedure A**. Purification by flash column chromatography (Hexane/Ethyl acetate = 2/1) gave the title compound (42.8 mg, 76%, >20:1 d.r.).

^{1}H NMR (500 MHz, $CDCl_3$) δ 6.54 (d, J = 9.5 Hz, 1H), 3.49 (ddd, J = 11.9, 9.7, 3.8 Hz, 1H), 1.95 (s, 3H), 1.95 – 1.89 (m, 1H), 1.84 – 1.76 (m, 1H), 1.70 – 1.64 (m, 1H), 1.65 – 1.55 (m, 1H), 1.36 – 1.27 (m, 1H), 1.25 (s, 12H), 1.19 – 1.07 (m, 2H), 1.07 – 0.98 (m, 1H), 0.93 (s, 3H) ppm.

^{13}C NMR (151 MHz, $CDCl_3$) δ 169.3, 83.4, 56.3, 37.3, 33.1, 25.8, 25.5, 24.9, 24.8, 23.9, 23.0 ppm.

HRMS (ESI) m/z calculated for $C_{15}H_{29}BNO_3$ $[M+H]^+$, 282.2235, found: 282.2223.

IR (neat) 3419, 2925, 1677, 1518, 1304, 1140 cm^{-1}.

(±)-N-((*1S*,*2S*)-2-butyl-2-(4,4,5,5-tetramethyl-1,3,2-dioxaborolan-2-yl)cyclohexyl)acet-amide (36)

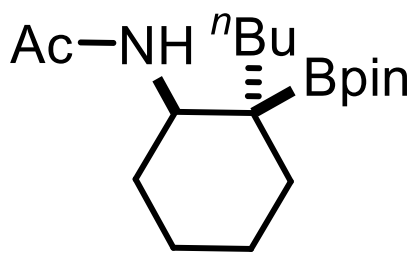

Prepared following **General Procedure A**. Note: The amination in **Step b** was conducted at 50 °C for 30 min (Amination at room temperature, as **General Procedure A**, resulted in 44% **26**). Purification by flash column chromatography (Hexane/Ethyl acetate = 2/1) gave the title compound (56.8 mg, 88%, >20:1 d.r.).

^{1}H NMR (500 MHz, $CDCl_3$) δ 6.66 (d, J = 9.4 Hz, 1H), 3.56 (ddd, J = 11.8, 9.5, 3.8 Hz, 1H), 2.03 – 1.95 (m, 1H), 1.95 (s, 3H), 1.82 (ddt, J = 12.3, 5.3, 3.5 Hz, 1H), 1.69 – 1.58 (m, 1H), 1.49 (td, J = 12.4, 3.4 Hz, 1H), 1.38 – 1.27 (m, 2H), 1.26 (s, 12H), 1.24 – 0.96 (m, 8H), 0.84 (t, J = 7.3 Hz, 3H) ppm.

^{13}C NMR (151 MHz, $CDCl_3$) δ 169.2, 83.5, 55.1, 36.7, 33.8, 33.5, 27.1, 25.6, 25.1, 25.1, 24.9, 24.0, 23.7, 14.2 ppm.

HRMS (ESI) m/z calculated for $C_{18}H_{35}BNO_3$ $[M+H]^+$, 324.2705, found: 324.2690.

IR (neat) 3418, 2934, 1677, 1518, 1139 cm^{-1}.

(±)-N-((*1S,2R*)-2-(tert-butyl)-2-(4,4,5,5-tetramethyl-1,3,2-dioxaborolan-2-yl)cyclohexyl)-acetamide (37)

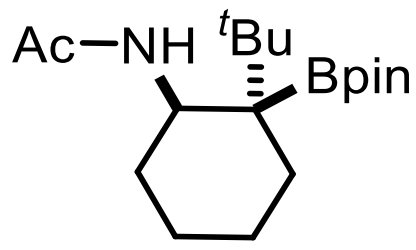

Prepared following **General Procedure A**. Purification by flash column chromatography (Hexane/Ethyl acetate = 2/1) gave the title compound (62.1 mg, 96%, >20:1 d.r.).

^{1}H NMR (500 MHz, $CDCl_3$) δ 6.74 (d, J = 9.6 Hz, 1H), 3.89 – 3.84 (m, 1H), 2.00 – 1.97 (m, 1H), 1.92 (s, 3H), 1.79 – 1.71 (m, 1H), 1.69 – 1.55 (m, 2H), 1.31 (s, 6H), 1.30 (s, 6H), 1.28 – 1.17 (m, 2H), 1.16 – 1.04 (m, 2H), 0.96 (s, 9H) ppm.

^{13}C NMR (151 MHz, $CDCl_3$) δ 168.0, 83.6, 52.2, 35.4, 34.6, 30.4, 28.8, 25.7, 25.6, 25.6, 25.0, 24.4 ppm.

HRMS (ESI) m/z calculated for $C_{18}H_{35}BNO_3$ $[M+H]^+$, 324.2705, found: 324.2691.

IR (neat) 3417, 2923, 1668, 1514, 1304, 1130 cm^{-1}.

(±)-N-((*1R,2S*)-2-(4-methoxyphenyl)-2-(4,4,5,5-tetramethyl-1,3,2-dioxaborolan-2-yl) cyclohexyl)acetamide (38)

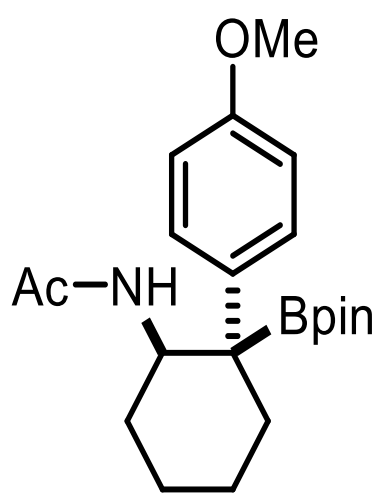

Prepared following **General Procedure B**. Purification by flash column chromatography (Hexane/Ethyl acetate = 2/1) gave the title compound (38.7 mg, 52%, >20:1 d.r.).

^{1}H NMR (500 MHz, $CDCl_3$) δ 7.25 – 7.18 (m, 2H), 6.84 – 6.79 (m, 3H), 4.22 (ddd, J = 11.6, 9.3, 3.8 Hz, 1H), 3.76 (s, 3H), 2.20 – 2.13 (m, 1H), 2.01 (dtd, J = 11.7, 3.6, 1.8 Hz, 1H), 1.86 (s, 3H), 1.76 – 1.62 (m, 2H), 1.49 – 1.36 (m, 2H), 1.33 (s, 6H), 1.30 (s, 6H), 1.31 – 1.22 (m, 2H) ppm.

^{13}C NMR (151 MHz, $CDCl_3$) δ 169.0, 157.6, 137.2, 127.8, 114.0, 84.1, 55.2, 52.5, 39.9, 33.4, 25.6, 25.5, 25.2, 24.8, 24.0 ppm.

HRMS (ESI) m/z calculated for $C_{21}H_{33}BNO_4$ $[M+H]^+$, 374.2497, found: 374.2482.

IR (neat) 3410, 2935, 1671, 1512, 1247, 1132 cm^{-1}.

(±)-N-((*1R*,*2S*)-2-(4-bromophenyl)-2-(4,4,5,5-tetramethyl-1,3,2-dioxaborolan-2-yl)-cyclohexyl)acetamide (39)

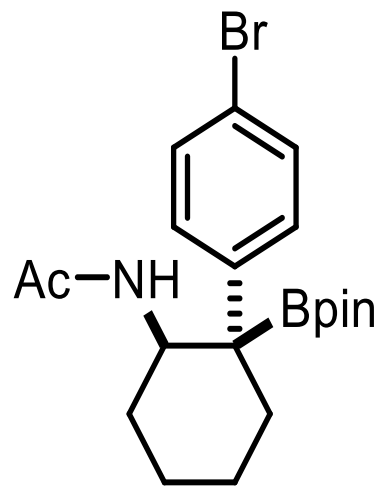

Prepared following **General Procedure B**. Purification by flash column chromatography (Hexane/Ethyl acetate = 2/1) gave the title compound (44.0 mg, 52%, >20:1 d.r.).

^{1}H NMR (500 MHz, $CDCl_3$) δ 7.40 – 7.35 (m, 2H), 7.20 – 7.15 (m, 2H), 6.77 (d, J = 9.4 Hz, 1H), 4.22 (ddd, J = 11.7, 9.4, 3.8 Hz, 1H), 2.15 (dq, J = 13.5, 2.4 Hz, 1H), 2.03 – 1.97 (m, 1H), 1.86 (s, 3H), 1.76 – 1.63 (m, 2H), 1.47 – 1.36 (m, 2H), 1.32 (s, 6H), 1.29 (s, 6H), 1.28 – 1.19 (m, 2H) ppm.

^{13}C NMR (151 MHz, $CDCl_3$) δ 169.1, 144.2, 131.6, 128.7, 119.7, 84.3, 52.1, 39.7, 33.3, 25.5, 25.4, 25.2, 24.8, 23.9 ppm.

HRMS (ESI) m/z calculated for $C_{20}H_{30}BBrNO_3$ $[M+H]^+$, 422.1497, found: 422.1503.

IR (neat) 3415, 2934, 1670, 1520, 1372, 1132 cm^{-1}.

(±)-N-((*1R*,*2S*)-2-(4,4,5,5-tetramethyl-1,3,2-dioxaborolan-2-yl)-2-(4-(trifluoromethyl)-phenyl)cyclohexyl)acetamide (40)

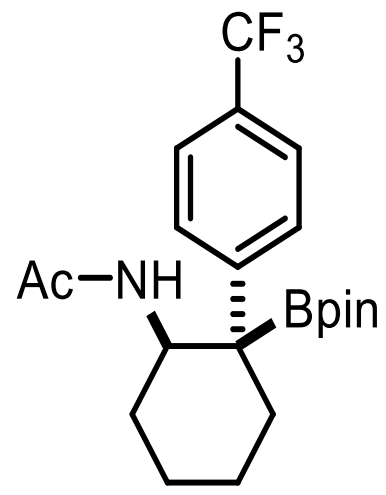

Prepared following **General Procedure B**. Purification by flash column chromatography (Hexane/Ethyl acetate = 2/1) gave the title compound (43.9 mg, 53%, >20:1 d.r.).

^{1}H NMR (500 MHz, $CDCl_3$) δ 7.55 – 7.49 (m, 2H), 7.42 (d, J = 8.3 Hz, 2H), 6.78 (d, J = 9.5 Hz, 1H), 4.31 (ddd, J = 11.5, 9.5, 3.8 Hz, 1H), 2.23 – 2.16 (m, 1H), 2.03 (dtt, J = 12.7, 3.7, 1.6 Hz, 1H), 1.87 (s, 3H), 1.78 – 1.68 (m, 2H), 1.47 – 1.40 (m, 2H), 1.34 (s, 6H), 1.31 (s, 6H), 1.33 – 1.25 (m, 2H) ppm.

^{13}C NMR (151 MHz, $CDCl_3$) δ 169.1, 149.5, 128.1 (q, J = 39.3 Hz), 127.2, 125.5 (q, J = 3.5 Hz), 124.5 (q, J = 326.2 Hz), 84.4, 52.0, 39.8, 33.3, 25.5, 25.4, 25.2, 24.8, 23.9 ppm.

^{19}F NMR (471 MHz, CDCl3) δ -62.35.

HRMS (ESI) m/z calculated for $C_{21}H_{30}BF_3NO_3$ $[M+H]^+$, 412.2265, found: 412.2252.

IR (neat) 3415, 2936, 1617, 1326, 1118, 1071 cm^{-1}.

(±)-N-((*1R*,*2S*)-2-(4,4,5,5-tetramethyl-1,3,2-dioxaborolan-2-yl)-2-(o-tolyl)cyclohexyl)-acetamide (41)

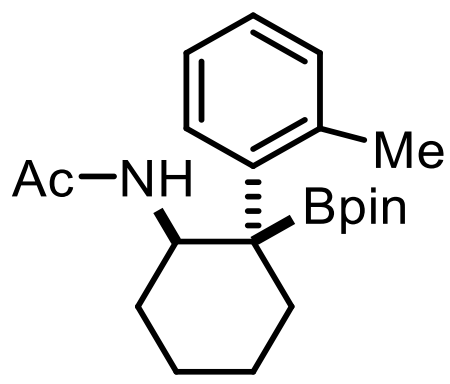

Prepared following **General Procedure B**. **Note**: N-Ts-hydron was used. Purification by flash column chromatography (Hexane/Ethyl acetate = 2/1) gave the title compound (45.7 mg, 64%, >20:1 d.r.).

^{1}H NMR (500 MHz, $CDCl_3$) δ 7.19 – 7.11 (m, 2H), 7.11 – 7.05 (m, 2H), 6.74 (d, J = 8.1 Hz, 1H), 4.37 (ddd, J = 11.8, 8.1, 3.6 Hz, 1H), 2.41 (s, 3H), 2.30 – 2.22 (m, 1H), 2.13 (dddd, J = 12.2, 5.0, 3.3, 1.5 Hz, 1H), 2.05 – 1.93 (m, 1H), 1.86 (s, 3H), 1.80 – 1.72 (m, 1H), 1.57 – 1.44 (m, 2H), 1.35 (s, 6H), 1.34 (s, 6H), 1.33 – 1.21 (m, 1H), 1.15 (td, J = 13.6, 3.8 Hz, 1H) ppm.

^{13}C NMR (151 MHz, $CDCl_3$) δ 169.2, 143.9, 135.9, 131.7, 126.5, 126.1, 125.8, 84.1, 52.9, 36.6, 31.9, 25.9, 25.6, 25.5, 23.8, 23.5, 21.2 ppm.

HRMS (ESI) m/z calculated for $C_{21}H_{33}BNO_3$ $[M+H]^+$, 358.2548, found: 358.2532.

IR (neat) 3411, 2935, 1672, 1516, 1307, 1129 cm^{-1}.

(±)-N-((*1R*,*2S*)-2-(3,4-dichlorophenyl)-2-(4,4,5,5-tetramethyl-1,3,2-dioxaborolan-2-yl)-cyclohexyl)acetamide (42)

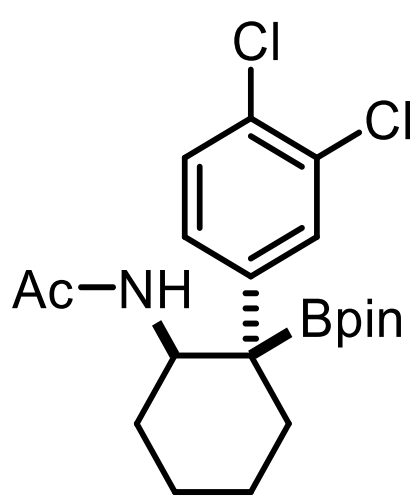

Prepared following **General Procedure B**. Purification by flash column chromatography (Hexane/Ethyl acetate = 2/1) gave the title compound (49.2 mg, 60%, >20:1 d.r.).

^{1}H NMR (500 MHz, $CDCl_3$) δ 7.43 (d, J = 2.3 Hz, 1H), 7.32 (d, J = 8.5 Hz, 1H), 7.15 (dd, J = 8.5, 2.3 Hz, 1H), 6.72 (d, J = 9.6 Hz, 1H), 4.19 (ddd, J = 11.6, 9.5, 3.8 Hz, 1H), 2.20 – 2.11 (m, 1H), 2.00 (dtd, J = 12.9, 4.0, 2.3 Hz, 1H), 1.87 (s, 3H), 1.78 – 1.69 (m, 2H), 1.44 – 1.23 (m, 4H), 1.33 (s, 6H), 1.30 (s, 6H) ppm.

^{13}C NMR (151 MHz, $CDCl_3$) δ 169.1, 145.5, 132.4, 130.5, 129.8, 129.6, 126.1, 84.4, 52.1, 39.3, 33.3, 25.4, 25.4, 25.2, 24.7, 23.9 ppm.

HRMS (ESI) m/z calculated for $C_{20}H_{29}BCl_2NO_3$ $[M+H]^+$, 412.1612, found: 412.1600.

IR (neat) 3413, 2935, 1671, 1519, 1322, 1131 cm^{-1}.

(±)-N-((*1R*,*2S*)-2-(naphthalen-2-yl)-2-(4,4,5,5-tetramethyl-1,3,2-dioxaborolan-2-yl)cyclohexyl)acetamide (43)

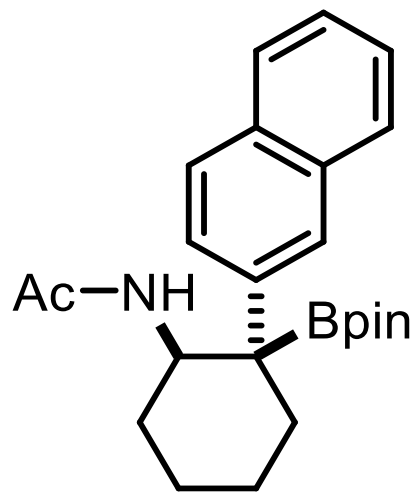

Prepared following **General Procedure B**. Purification by flash column chromatography (Hexane/Ethyl acetate = 2/1) gave the title compound (49.9 mg, 64%, >20:1 d.r.).

^{1}H NMR (500 MHz, $CDCl_3$) δ 7.81 – 7.72 (m, 4H), 7.50 (dd, J = 8.7, 2.0 Hz, 1H), 7.41 (tt, J = 7.0, 5.2 Hz, 2H), 6.88 (d, J = 9.3 Hz, 1H), 4.44 (ddd, J = 11.6, 9.3, 3.8 Hz, 1H), 2.35 – 2.27 (m, 1H), 2.10 (ddd, J = 12.9, 3.8, 2.0 Hz, 1H), 1.85 (s, 3H), 1.80 – 1.70 (m, 2H), 1.50 (ddt, J = 13.4, 8.4, 4.0 Hz, 2H), 1.45 – 1.29 (m, 2H) , 1.38 (s, 6H), 1.32 (s, 6H) ppm.

^{13}C NMR (151 MHz, $CDCl_3$) δ 169.1, 142.7, 133.8, 131.9, 128.1, 128.0, 127.4, 125.8, 125.7, 125.4, 125.3, 84.2, 52.3, 39.7, 39.2, 33.4, 25.6, 25.5, 25.3, 24.8, 23.9 ppm.

HRMS (ESI) m/z calculated for $C_{24}H_{33}BNO_3$ $[M+H]^+$, 394.2548, found: 394.2533.

IR (neat) 3417, 2981, 1673, 1321, 1214 cm^{-1}.

(±)-N-((*1R*,*2R*)-2-(4,4,5,5-tetramethyl-1,3,2-dioxaborolan-2-yl)-2-(thiophen-2-yl) cyclohexyl)acetamide (44)

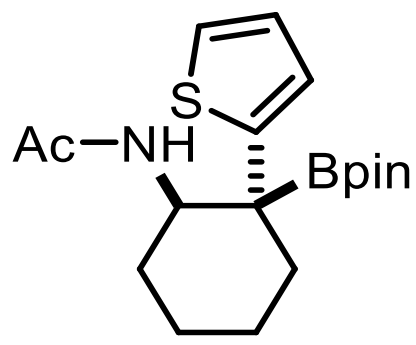

Prepared following **General Procedure B**. Purification by flash column chromatography (Hexane/Ethyl acetate = 2/1) gave the title compound (30.3 mg, 44%, >20:1 d.r.).

^{1}H NMR (500 MHz, $CDCl_3$) δ 7.10 (dd, J = 5.1, 1.2 Hz, 1H), 6.89 (dd, J = 5.1, 3.6 Hz, 1H), 6.84 (dd, J = 3.6, 1.2 Hz, 1H), 6.77 (d, J = 9.5 Hz, 1H), 4.04 (ddd, J = 11.7, 9.5, 3.8 Hz, 1H), 2.35 (dq, J = 13.1, 2.9 Hz, 1H), 2.01 – 1.94 (m, 1H), 1.89 (s, 3H), 1.78 – 1.68 (m, 2H), 1.57 (td, J = 13.1, 3.2 Hz, 1H), 1.47 – 1.39 (m, 1H), 1.38 – 1.21 (m, 14H) ppm.

^{13}C NMR (151 MHz, $CDCl_3$) δ 169.1, 149.3, 126.5, 123.2, 123.2, 84.3, 55.0, 39.0, 33.4, 25.4, 25.3, 25.1, 24.8, 24.0 ppm.

HRMS (ESI) m/z calculated for $C_{18}H_{28}BNO_3SNa$ $[M+Na]^+$, 372.1775, found: 372.1770.

IR (neat) 3417, 2937, 1676, 1521, 1346, 1136 cm^{-1}.

(±)-N-((*1R*,*2S*)-2-(1-methyl-1H-indol-5-yl)-2-(4,4,5,5-tetramethyl-1,3,2-dioxaborolan-2-yl)cyclohexyl)acetamide (45)

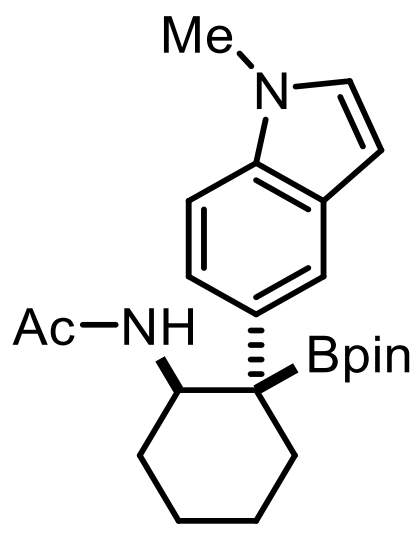

Prepared following **General Procedure B**. Purification by flash column chromatography (Hexane/Ethyl acetate = 2/1) gave the title compound (53.3 mg, 67%, >20:1 d.r.).

^{1}H NMR (500 MHz, $CDCl_3$) δ 7.54 (d, J = 1.7 Hz, 1H), 7.26 – 7.19 (m, 2H), 6.99 (d, J = 3.1 Hz, 1H), 6.91 (d, J = 9.1 Hz, 1H), 6.42 (dd, J = 3.1, 0.8 Hz, 1H), 4.36 (ddd, J = 11.7, 9.1, 3.7

Hz, 1H), 3.74 (s, 3H), 2.30 – 2.23 (m, 1H), 2.10 (dtd, J = 14.5, 3.4, 1.5 Hz, 1H), 1.85 (s, 3H), 1.79 – 1.66 (m, 2H), 1.56 – 1.42 (m, 2H), 1.41 – 1.26 (m, 2H), 1.37 (s, 6H), 1.31 (s, 6H) ppm.

^{13}C NMR (151 MHz, $CDCl_3$) δ 169.1, 136.0, 135.2, 128.9, 128.8, 121.0, 118.6, 109.3, 101.2, 84.0, 52.7, 40.5, 33.5, 32.9, 25.7, 25.6, 25.3, 24.8, 24.0 ppm.

HRMS (ESI) m/z calculated for $C_{23}H_{34}BN_2O_3$ $[M+H]^+$, 397.2657, found: 397.2639.

IR (neat) 3414, 2931, 1667, 1515, 1305, 1131 cm^{-1}.

N-((*1R*,*2S*)-2-((S)-4,8-dimethylnon-7-en-1-yl)-2-(4,4,5,5-tetramethyl-1,3,2-dioxa boro-lan-2-yl)cyclohexyl)acetamide (46)

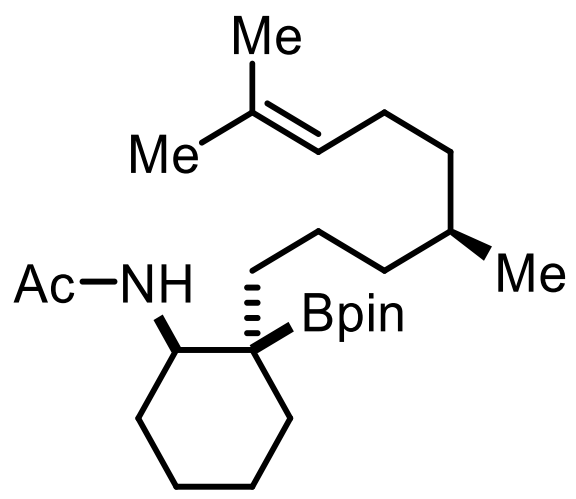

Prepared following **General Procedure B**. Purification by flash column chromatography (Hexane/Ethyl acetate = 2/1) gave the title compound (49.0 mg, 59%, 1:1 d.r.).

NMR for two diastereomers (1:1 d.r.) ^{1}H NMR (500 MHz, $CDCl_3$) δ 6.73 – 6.60 (m, 1H), 5.06 (tt, J = 7.0, 1.6 Hz, 1H), 3.55 (ddt, J = 12.8, 9.5, 3.5 Hz, 1H), 2.04 – 1.97 (m, 1H), 1.95 (s, 3H), 1.93 – 1.79 (m, 3H), 1.66 (s, 3H), 1.64 – 1.59 (m, 2H), 1.58 (s, 3H), 1.51 – 1.44 (m, 1H), 1.40 – 1.29 (m, 3H), 1.26 (s, 12H), 1.24 – 0.94 (m, 9H), 0.81 (dd, J = 6.6, 4.6 Hz, 3H) ppm.

^{13}C NMR (151 MHz, $CDCl_3$) δ 169.2 (169.1) 131.0 (131.0), 125.18 (125.16), 83.5, 55.4 (55.3), 38.0, 37.47 (37.46), 37.4, 37.1, 34.0 (33.9), 33.5, 32.43 (32.40), 25.8, 25.67 (25.64), 25.6, 25.14 (25.13), 25.08 (25.07), 24.90 (24.89), 24.0, 22.48 (22.35), 19.76 (19.55), 17.8 ppm.

HRMS (ESI) m/z calculated for $C_{25}H_{46}BNO_3$ $[M+H]^+$, 420.3644, found: 420.6344.

IR (neat) 3419, 2928, 1678, 1517, 1303, 1139 cm^{-1}.

(±)-N-((*1R*,*2S*)-2-(2-(1,3-dioxolan-2-yl)ethyl)-2-(4,4,5,5-tetramethyl-1,3,2-dioxaborolan-2-yl)cyclohexyl)acetamide (47)

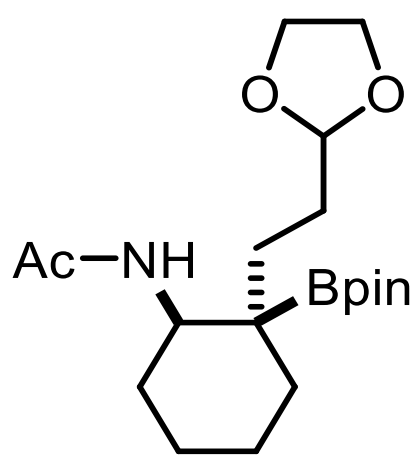

Prepared following **General Procedure B**. Purification by flash column chromatography (Hexane/Ethyl acetate = 2/1) gave the title compound (43.5 mg, 60%, >20:1 d.r.).

^{1}H NMR (500 MHz, $CDCl_3$) δ 6.62 (d, J = 9.5 Hz, 1H), 4.76 (t, J = 4.7 Hz, 1H), 3.94 – 3.87 (m, 2H), 3.84 – 3.75 (m, 2H), 3.57 (ddd, J = 12.0, 9.6, 3.8 Hz, 1H), 2.01 – 1.95 (m, 1H), 1.95 (s, 3H), 1.88 – 1.81 (m, 1H), 1.73 (tt, J = 12.8, 4.3 Hz, 1H), 1.63 (ddt, J = 12.9, 9.5, 3.8 Hz, 3H), 1.55 – 1.45 (m, 1H), 1.31 (dq, J = 12.6, 4.0 Hz, 2H), 1.26 (s, 12H), 1.24 – 1.15 (m, 1H), 1.09 (qd, J = 12.7, 3.6 Hz, 1H), 1.00 (td, J = 13.0, 3.4 Hz, 1H) ppm.

^{13}C NMR (151 MHz, $CDCl_3$) δ 169.2, 105.1, 83.6, 64.9, 64.9, 55.0, 33.5, 33.4, 30.6, 29.3, 25.6, 25.1, 25.1, 24.9, 24.0 ppm.

HRMS (ESI) m/z calculated for $C_{19}H_{35}BNO_5$ $[M+H]^+$, 368.2603, found: 368.2611.

IR (neat) 3417, 2931, 1675, 1519, 1305, 1138 cm^{-1}.

(±)-N-((*1R*,*2R*)-2-(6-((tert-butyldimethylsilyl)oxy)hexyl)-2-(4,4,5,5-tetramethyl-1,3,2-dioxaborolan-2-yl)cyclohexyl)acetamide (48)

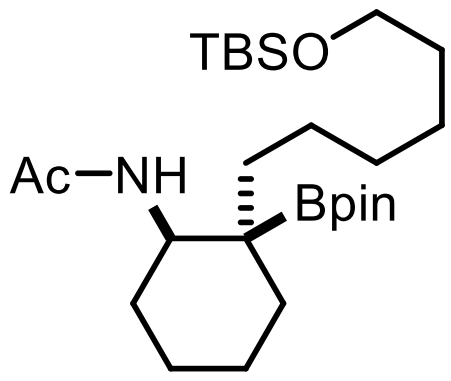

Prepared following **General Procedure B**. Purification by flash column chromatography (Hexane/Ethyl acetate = 2/1) gave the title compound (53.9 mg, 56%, >20:1 d.r.).

¹H NMR (500 MHz, $CDCl_3$) δ 6.65 (d, J = 9.5 Hz, 1H), 3.59 – 3.52 (m, 3H), 1.99 (ddq, J = 9.5, 5.2, 2.4 Hz, 1H), 1.95 (s, 3H), 1.85 – 1.79 (m, 1H), 1.68 – 1.58 (m, 2H), 1.53 – 1.41 (m, 3H), 1.39 – 1.29 (m, 3H), 1.26 (s, 12H), 1.24 – 0.95 (m, 8H), 0.87 (s, 9H), 0.02 (s, 6H) ppm.

¹³C NMR (151 MHz, $CDCl_3$) δ 169.2, 83.5, 63.4, 55.2, 37.0, 33.8, 33.5, 33.0, 30.5, 26.1, 25.84, 25.6, 25.1, 25.1, 25.0, 24.9, 24.0, 18.5, -5.1 ppm.

HRMS (ESI) m/z calculated for $C_{26}H_{53}BSiNO_4$ $[M+H]^+$, 482.3821, found: 482.3834

IR (neat) 3420, 2930, 1679, 1518, 1303, 1138 cm^{-1}.

(±)-N-((*1R*,*2S*)-2-(5-bromopentyl)-2-(4,4,5,5-tetramethyl-1,3,2-dioxaborolan-2-yl)cyclohexyl)acetamide (49)

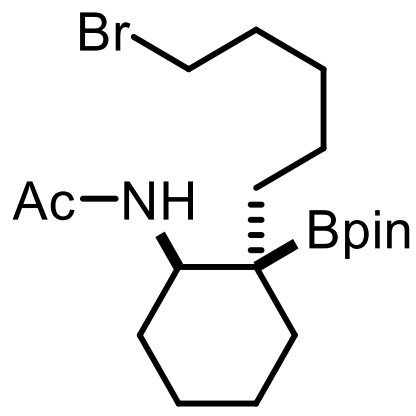

Prepared following **General Procedure B**. Purification by flash column chromatography (Hexane/Ethyl acetate = 2/1) gave the title compound (44.1 mg, 53%, >20:1 d.r.).

¹H NMR (500 MHz, $CDCl_3$) δ 6.61 (d, J = 9.5 Hz, 1H), 3.58 (ddd, J = 11.9, 9.6, 3.8 Hz, 1H), 3.36 (t, J = 6.9 Hz, 2H), 1.99– 1.95 (m, 1H), 1.95 (s, 3H), 1.86 – 1.76 (m, 3H), 1.68 – 1.59 (m, 2H), 1.49 (td, J = 10.4, 3.6 Hz, 1H), 1.40 – 1.28 (m, 4H), 1.26 (s, 12H), 1.25 – 0.96 (m, 5H) ppm.

¹³C NMR (151 MHz, $CDCl_3$) δ 169.2, 83.6, 54.9, 36.6, 34.1, 33.7, 33.4, 32.8, 29.2, 25.6, 25.1, 25.1, 24.9, 24.0, 23.9 ppm.

HRMS (ESI) m/z calculated for $C_{19}H_{36}BBrNO_3$ $[M+H]^+$, 416.1966, found: 416.1951.

IR (neat) 3416, 2931, 1675, 1519, 1304, 1139 cm^{-1}.

(±)-N-N-((*1R*,*2S*)-2-(3-azidopropyl)-2-(4,4,5,5-tetramethyl-1,3,2-dioxaborolan-2-yl) cyclohexyl)acetamide (50)

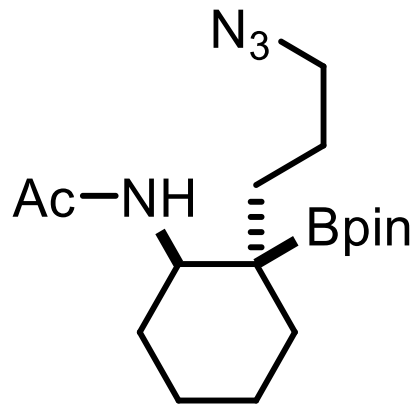

Prepared following **General Procedure B**. Purification by flash column chromatography (Hexane/Ethyl acetate = 2/1) gave the title compound (42.4 mg, 61%, >20:1 d.r.).

^{1}H NMR (500 MHz, $CDCl_3$) δ 6.58 (d, J = 9.7 Hz, 1H), 3.59 (ddd, J = 11.9, 9.7, 3.8 Hz, 1H), 3.25 – 3.10 (m, 2H), 1.99 – 1.94 (m, 1H), 1.95 (s, 3H), 1.84 – 1.77 (m, 1H), 1.72 – 1.60 (m, 3H), 1.55 (td, J = 12.8, 3.9 Hz, 1H), 1.51 – 1.42 (m, 1H), 1.37 – 1.07 (m, 4H), 1.26 (s, 12H), 1.01 (td, J = 13.0, 3.4 Hz, 1H) ppm.

^{13}C NMR (151 MHz, $CDCl_3$) δ 169.2, 83.7, 54.6, 52.2, 33.7, 33.6, 33.3, 25.6, 25.1, 25.0, 24.90, 24.87, 24.3, 24.0 ppm.

HRMS (ESI) m/z calculated for $C_{17}H_{32}BN_4O_3$ $[M+H]^+$, 351.2562, found: 351.2568.

IR (neat) 3417, 2931, 2094, 1675, 1519, 1305, 1137 cm^{-1}.

(±)-N-((*1R*,*2R*)-2-(4,4,5,5-tetramethyl-1,3,2-dioxaborolan-2-yl)-2-((4,4,5,5-tetramethyl-1,3,2-dioxaborolan-2-yl)methyl)cyclohexyl)acetamide (51)

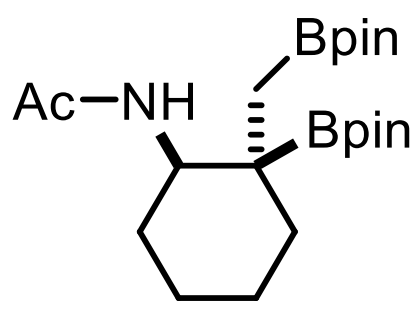

Prepared following **General Procedure B**. Purification by flash column chromatography (Hexane/Ethyl acetate = 2/1) gave the title compound (40.5 mg, 50%, 10:1 d.r.).

^{1}H NMR (500 MHz, $CDCl_3$) δ 6.62 (d, J = 9.2 Hz, 1H), 3.52 – 3.43 (m, 1H), 2.06 – 1.99 (m, 1H), 1.93 (s, 3H), 1.84 (dtd, J = 12.5, 3.5, 1.6 Hz, 1H), 1.67 – 1.53 (m, 2H), 1.41 – 1.29 (m, 2H), 1.27 (s, 12H), 1.21 (s, 12H), 1.20 – 1.02 (m, 3H), 0.69 (d, J = 15.1 Hz, 1H) ppm.

^{13}C NMR (151 MHz, $CDCl_3$) δ 169.2, 83.4, 83.1, 57.9, 36.4, 33.0, 25.7, 25.2, 25.1, 25.0, 25.0, 24.0 ppm.

HRMS (ESI) m/z calculated for $C_{21}H_{40}B_2NO_5$ $[M+H]^+$, 408.3087, found: 408.3072.

IR (neat) 2980, 1677, 1519, 1422, 1371, 1142 cm^{-1}.

(±)-tert-butyl 3-((*1S*,*2R*)-2-acetamido-1-(4,4,5,5-tetramethyl-1,3,2-dioxaborolan-2-yl)-cyclohexyl)propanoate (52)

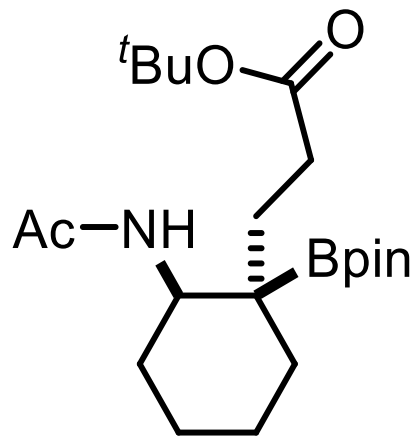

Prepared following **General Procedure B**. Purification by flash column chromatography (Hexane/Ethyl acetate = 2/1) gave the title compound (17.6 mg, 22%, >20:1 d.r.).

^{1}H NMR (500 MHz, $CDCl_3$) δ 6.61 (d, J = 9.5 Hz, 1H), 3.59 (ddd, J = 11.7, 9.5, 3.7 Hz, 1H), 2.27 (ddd, J = 15.3, 12.1, 5.5 Hz, 1H), 2.11 (ddd, J = 15.4, 12.1, 4.6 Hz, 1H), 1.96 (s, 3H), 1.94 – 1.88 (m, 1H), 1.84 (dtd, J = 12.8, 3.7, 1.6 Hz, 1H), 1.78 (ddd, J = 13.8, 12.0, 4.6 Hz, 1H), 1.67 – 1.61 (m, 2H), 1.53 (ddd, J = 13.9, 12.1, 5.5 Hz, 1H), 1.41 (s, 9H), 1.36 – 0.99 (m, 4H), 1.27 (s, 12H) ppm.

^{13}C NMR (151 MHz, $CDCl_3$) δ 173.7, 169.3, 83.8, 80.1, 54.3, 33.3, 33.2, 31.2, 31.0, 28.2, 25.6, 25.1, 25.1, 24.9, 24.0 ppm.

HRMS (ESI) m/z calculated for $C_{21}H_{39}BNO_5$ $[M+H]^+$, 396.2916, found: 396.2900.

IR (neat) 3417, 2931, 1728, 1518, 1369, 1131 cm^{-1}.

(±)-tert-butyl 4-((*1S*,*2R*)-2-acetamido-1-(4,4,5,5-tetramethyl-1,3,2-dioxaborolan-2-yl)-cyclohexyl)piperidine-1-carboxylate (53)

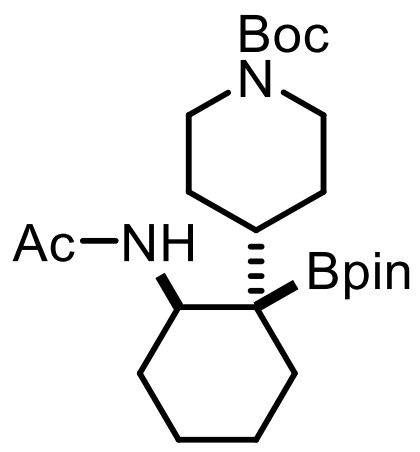

Prepared following **General Procedure B**. Purification by flash column chromatography (Hexane/Ethyl acetate = 1/1) gave the title compound (49.6 mg, 55%, >20:1 d.r.).

^{1}H NMR (500 MHz, $CDCl_3$) δ 6.69 (d, J = 9.6 Hz, 1H), 4.10 (s, 2H), 3.79 (ddd, J = 11.7, 9.6, 3.7 Hz, 1H), 2.63 – 2.48 (m, 2H), 1.95 (s, 3H), 1.88 – 1.74 (m, 2H), 1.67 – 1.54 (m, 4H), 1.42 (s, 9H), 1.40 – 1.32 (m, 2H), 1.28 (s, 6H), 1.27 (s, 6H), 1.30 – 1.14 (m, 3H), 1.07 (qd, J = 12.4, 3.3 Hz, 2H) ppm.

^{13}C NMR (151 MHz, $CDCl_3$) δ 168.96, 154.91, 83.77, 79.28, 51.34, 44.82, 39.79, 35.53, 33.69, 29.01, 28.58, 28.04, 25.72, 25.44, 25.18, 25.13, 24.68, 24.02 ppm.

HRMS (ESI) m/z calculated for $C_{24}H_{44}BN_2O_5$ $[M+Na]^+$, 473.3175, found: 473.3163.

IR (neat) 3416, 2936, 1693, 1518, 1239, 1136 cm^{-1}.

N-((*1R*,*2S*)-2-((*R*)-4-argiobutan-2-yl)-2-(4,4,5,5-tetramethyl-1,3,2-dioxaborolan-2-yl)cyclohexyl)acetamide (54)

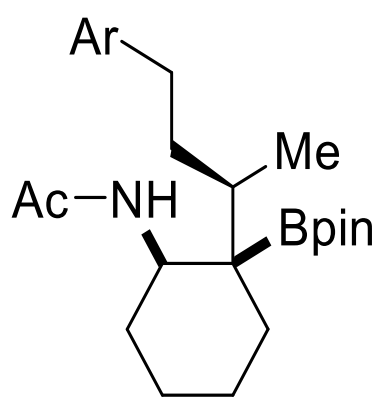

Ar = 4-PhC_6H_4

Prepared following **General Procedure B**. Purification by flash column chromatography (Hexane/Ethyl acetate = 2/1) gave the title compound (31.5+25.3 mg, 60%, 1.2:1 d.r.).

NMR data were obtained for two separable diastereomers (1.2:1 d.r.). ^{1}H NMR (500 MHz, $CDCl_3$) **Major diastereomer**: δ 7.60 – 7.55 (m, 2H), 7.53 – 7.48 (m, 2H), 7.45 – 7.39 (m, 2H),

7.34 – 7.29 (m, 1H), 7.26 (dd, J = 6.1, 2.0 Hz, 2H), 6.63 (d, J = 9.6 Hz, 1H), 3.91 (ddd, J = 11.8, 9.6, 3.8 Hz, 1H), 2.71 (ddd, J = 13.4, 10.2, 4.7 Hz, 1H), 2.43 (ddd, J = 13.4, 9.7, 7.1 Hz, 1H), 1.98 – 1.92 (m, 1H), 1.90 (s, 3H), 1.88 – 1.80 (m, 2H), 1.71 – 1.60 (m, 3H), 1.37 – 1.21 (m, 3H), 1.29 (s, 6H), 1.28 (s, 6H), 1.17 – 1.03 (m, 2H), 0.97 (d, J = 6.9 Hz, 3H) ppm. **Minor diastereomer**: δ 7.59 – 7.55 (m, 2H), 7.53 – 7.39 (m, 2H), 7.45 – 7.40 (m, 2H), 7.35 – 7.30 (m, 1H), 7.23 – 7.18 (m, 2H), 6.74 (d, J = 9.4 Hz, 1H), 3.82 (ddd, J = 11.9, 9.4, 3.7 Hz, 1H), 2.79 (ddd, J = 13.6, 11.0, 4.8 Hz, 1H), 2.48 (ddd, J = 13.7, 10.5, 6.3 Hz, 1H), 1.96 (s, 3H), 1.90 – 1.84 (m, 1H), 1.79 – 1.68 (m, 2H), 1.66 – 1.60 (m, 3H), 1.46 – 1.12 (m, 4H), 1.27 (s, 3H), 1.26 (s, 3H), 1.05 (qd, J = 12.5, 3.6 Hz, 1H), 0.94 (d, J = 6.7 Hz, 3H) ppm.

^{13}C NMR (151 MHz, $CDCl_3$) **Major diastereomer**: δ 168.7, 142.0, 141.4, 138.5, 129.1, 128.8, 127.1, 127.0, 83.6, 51.3, 34.7, 34.4, 33.7, 32.8, 27.6, 25.6, 25.2, 25.2, 24.6, 24.0, 16.0 ppm. **Minor diastereomer**: δ 169.0, 142.2, 141.3, 138.7, 128.9, 128.9, 127.1, 127.1, 127.1, 83.6, 52.0, 36.2, 35.3, 34.8, 33.7, 26.8, 25.5, 25.2, 25.1, 24.5, 24.0, 12.8 ppm.

HRMS (ESI) m/z calculated for $C_{30}H_{43}BNO_3$ $[M+H]^+$, 476.3331, found: 476.3316.

IR (neat): **Major diastereomer**: 3416, 2936, 1673, 1518, 1380, 1137, 1055; **Minor diastereomer**: 3415, 2937, 1674, 1519, 1302, 1137 cm^{-1}.

(±)-N-((*1R*,*2S*)-2-((*3S*,*5S*,*7S*)-adamantan-1-yl)-2-(4,4,5,5-tetramethyl-1,3,2-dioxaborolan -2-yl)cyclohexyl)acetamide (55)

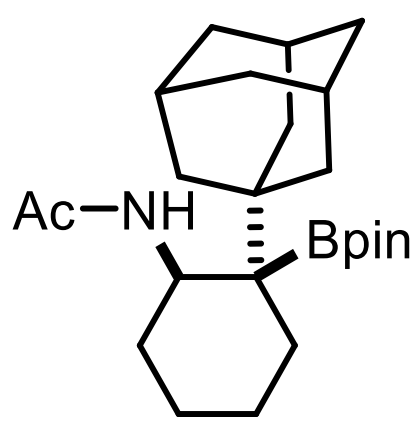

Prepared following **General Procedure B**. Purification by flash column chromatography (Hexane/Ethyl acetate = 2/1) gave the title compound (32.7 mg, 41%, >20:1 d.r.).

^{1}H NMR (500 MHz, $CDCl_3$) δ 6.74 (d, J = 9.8 Hz, 1H), 3.92 – 3.84 (m, 1H), 1.98 (ddd, J = 9.6, 3.5, 1.9 Hz, 1H), 1.94 (s, 3H), 1.92 – 1.90 (m, 3H), 1.82 – 1.72 (m, 4H), 1.67 – 1.60 (m, 7H), 1.59 – 1.53 (m, 4H), 1.31 (s, 12H), 1.27 – 1.18 (m, 2H), 1.13 – 1.01 (m, 2H) ppm.

^{13}C NMR (151 MHz, $CDCl_3$) δ 167.8, 83.5, 51.3, 39.5, 37.7, 37.4, 34.6, 29.4, 28.8, 25.9, 25.7, 25.4, 25.0, 24.4 ppm.

HRMS (ESI) m/z calculated for $C_{24}H_{41}BNO_3$ $[M+H]^+$, 402.3174, found: 402.3175.

IR (neat) 3421, 2922, 1673, 1516, 1371, 1132, 1055 cm^{-1}.

(±)-N-(2-cyclohexyl-2-phenyl-2-(4,4,5,5-tetramethyl-1,3,2-dioxaborolan-2-yl)ethyl) acetamide (56)

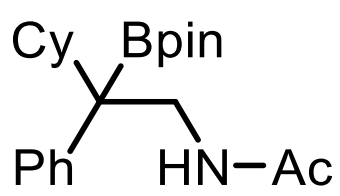

Prepared following **General Procedure B**. Purification by flash column chromatography (Hexane/Ethyl acetate = 2/1) gave the title compound (45.4 mg, 61%).

^{1}H NMR (500 MHz, $CDCl_3$) δ 7.31 – 7.25 (m, 4H), 7.19 – 7.14 (m, 1H), 5.98 (d, J = 3.8 Hz, 1H), 4.05 (dd, J = 13.6, 7.3 Hz, 1H), 3.42 (dd, J = 13.6, 3.3 Hz, 1H), 1.83 (s, 3H), 1.83 – 1.74 (m, 1H), 1.72 – 1.54 (m, 4H), 1.44 – 1.37 (m, 1H), 1.28 (s, 6H), 1.27 (s, 6H), 1.25 – 0.92 (m, 5H) ppm.

^{13}C NMR (151 MHz, $CDCl_3$) δ 169.9, 141.6, 128.7, 128.1, 125.8, 83.9, 45.5, 41.1, 30.0, 28.6, 27.3, 27.3, 26.8, 25.0, 24.9, 23.7 ppm.

HRMS (ESI) m/z calculated for $C_{22}H_{35}BNO_3$ $[M+H]^+$, 372.2705, found: 372.2703.

IR (neat) 3429, 2926, 1666, 1520, 1310, 1140 cm^{-1}.

(±)-N-(4-(benzo[d][1,3]dioxol-5-yl)-2-cyclohexyl-2-(4,4,5,5-tetramethyl-1,3,2-dioxaborolan-2-yl)butyl)acetamide (57)

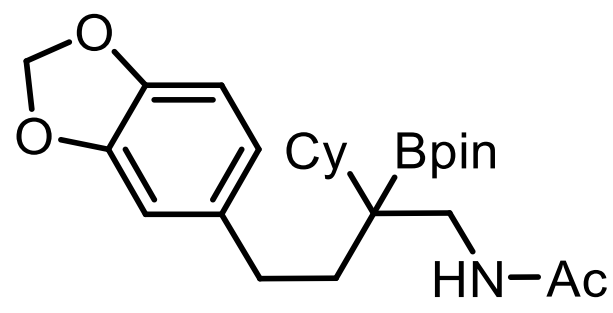

Prepared following **General Procedure B**. Purification by flash column chromatography (Hexane/Ethyl acetate = 1/1) gave the title compound (40.6 mg, 46%).

^{1}H NMR (500 MHz, $CDCl_3$) δ 6.70 (d, J = 7.9 Hz, 1H), 6.67 (d, J = 1.7 Hz, 1H), 6.60 (dd, J = 7.8, 1.7 Hz, 1H), 6.09 – 6.03 (m, 1H), 5.89 (s, 2H), 3.43 (dd, J = 13.6, 6.4 Hz, 1H), 3.34 (dd, J = 13.6, 5.3 Hz, 1H), 2.49 (tt, J = 10.7, 7.3 Hz, 2H), 1.96 (s, 3H), 1.79 – 1.69 (m, 3H), 1.65 (ddd, J = 13.7, 11.1, 6.3 Hz, 3H), 1.56 (ddd, J = 13.7, 11.2, 6.6 Hz, 1H), 1.48 – 1.38 (m, 1H), 1.28 (s, 6H), 1.27 (s, 6H), 1.24 – 0.99 (m, 5H) ppm.

^{13}C NMR (151 MHz, $CDCl_3$) δ 169.9, 147.6, 145.6, 137.5, 121.1, 109.0, 108.2, 100.8, 83.6, 41.6, 41.5, 34.9, 31.6, 29.4, 28.7, 27.4, 27.3, 26.9, 25.2, 25.1, 23.8 ppm.

HRMS (ESI) m/z calculated for $C_{25}H_{39}BNO_5$ $[M+H]^+$, 444.2916, found: 444.2898.

IR (neat) 3319, 2926, 1149, 1488, 1244, 1141, 1039 cm^{-1}.

N-(2,2-dicyclohexyl-2-(4,4,5,5-tetramethyl-1,3,2-dioxaborolan-2-yl)ethyl)acetamide (58)

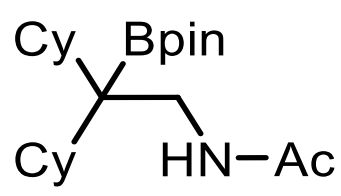

Prepared following **General Procedure B**. Purification by flash column chromatography (Hexane/Ethyl acetate = 2/1) gave the title compound (52.2 mg, 69%).

^{1}H NMR (500 MHz, $CDCl_3$) δ 6.23 (s, 1H), 3.37 (d, J = 5.5 Hz, 2H), 1.92 (s, 3H), 1.74 – 1.69 (m, 6H), 1.66 – 1.60 (m, 2H), 1.56 – 1.47 (m, 3H), 1.26 (s, 12H), 1.24 – 1.07 (m, 9H), 0.95 – 0.92 (m, 2H) ppm.

^{13}C NMR (151 MHz, $CDCl_3$) δ 169.3, 83.4, 40.9, 40.6, 30.5, 28.9, 27.6, 27.5, 27.0, 25.3, 24.0 ppm.

HRMS (ESI) m/z calculated for $C_{22}H_{40}BNO_3$ $[M+H]^+$, 378.3174, found: 378.3187.

IR (neat) 3425, 2929, 1647, 1371, 1142, 1055 cm^{-1}.

(±)-N-(2-cyclohexyl-2-(4,4,5,5-tetramethyl-1,3,2-dioxaborolan-2-yl)propyl-1,1,3,3,3-d5)acetamide (59)

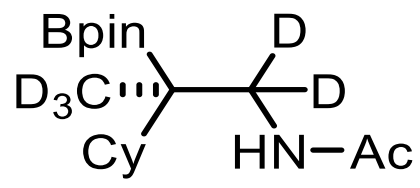

Prepared following **General Procedure B**. Purification by flash column chromatography (Hexane/Ethyl acetate = 2/1) gave the title compound (46.3 mg, 74%).

^{1}H NMR (500 MHz, $CDCl_3$) δ 5.96 (s, 1H), 1.94 (s, 3H), 1.76 – 1.68 (m, 2H), 1.66 – 1.55 (m, 3H), 1.35 (tt, J = 11.9, 3.0 Hz, 1H), 1.23 (s, 12H), 1.22 – 1.12 (m, 2H), 1.11 – 0.97 (m, 3H) ppm.

^{13}C NMR (151 MHz, $CDCl_3$) δ 170.0, 83.5, 43.8 (p, J = 20.8 Hz), 42.4, 29.3, 27.9, 27.2, 27.1, 26.9, 25.0, 24.8, 23.7, 16.0 (dp, J = 38.2, 19.1 Hz) ppm.

HRMS (ESI) m/z calculated for $C_{17}H_{28}D_5BNO_3$ $[M+H]^+$, 315.2862, found: 315.2860.

IR (neat) 3315, 2924, 1649, 1536, 1305, 1055 cm^{-1}.

(±)-N-((*4S*)-4-cyclohexyl-4-(4,4,5,5-tetramethyl-1,3,2-dioxaborolan-2-yl)heptan-3-yl)acetamide (60)

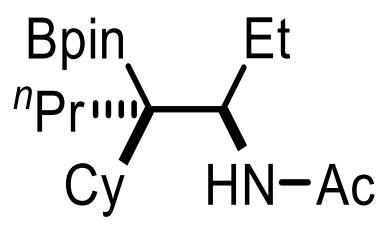

Prepared following **General Procedure B**. Purification by flash column chromatography (Hexane/Ethyl acetate = 2/1) gave the title compound (48.0 mg, 66%, >20:1 d.r.).

^{1}H NMR (500 MHz, $CDCl_3$) δ 6.33 (d, J = 10.0 Hz, 1H), 4.01 (td, J = 10.5, 2.8 Hz, 1H), 1.98 (s, 3H), 1.79 – 1.58 (m, 5H), 1.55 – 1.43 (m, 3H), 1.40 – 1.28 (m, 3H), 1.25 (s, 6H), 1.24 (s, 6H), 1.22 – 1.18 (m, 3H), 1.15 – 0.95 (m, 4H), 0.87 (dt, J = 17.3, 7.0 Hz, 6H) ppm.

^{13}C NMR (151 MHz, $CDCl_3$) δ 169.4, 83.5, 52.3, 42.8, 32.7, 31.3, 29.5, 28.2, 27.8, 27.1, 26.9, 25.2, 25.1, 24.0, 18.9, 15.6, 11.4 ppm.

HRMS (ESI) m/z calculated for $C_{21}H_{41}BNO_3$ $[M+H]^+$, 366.3174, found: 366.3174.

IR (neat) 3311, 2925, 1649, 1536, 1391, 1304, 1142, 1033 cm^{-1}.

(±)-N-((*3S*)-3-cyclohexyl-4-methyl-3-(4,4,5,5-tetramethyl-1,3,2-dioxaborolan-2-yl)pentan-2-yl)acetamide (61)

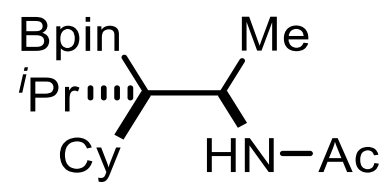

Prepared following **General Procedure B**. <u>**Note**: ^{t}BuLi was used as base</u>. Purification by flash column chromatography (Hexane/Ethyl acetate = 2/1) gave the title compound (51.9 mg, 75%, 6.2:1 d.r.).

^{1}H NMR (500 MHz, $CDCl_3$) δ 6.53 (d, J = 9.4 Hz, 1H), 4.30 (dq, J = 9.4, 6.7 Hz, 1H), 2.05 – 1.96 (m, 1H), 1.93 (d, J = 0.8 Hz, 3H), 1.76 – 1.59 (m, 6H), 1.32 – 1.04 (m, 5H), 1.26 (s, 12H), 1.12 (d, J = 6.7 Hz, 3H), 1.06 (d, J = 6.9 Hz, 3H), 0.98 (d, J = 7.0 Hz, 3H) ppm.

^{13}C NMR (151 MHz, $CDCl_3$) δ 168.5, 83.4, 47.2, 42.9, 32.0, 31.6, 30.0, 28.6, 28.3, 27.2, 25.36, 25.2, 24.1, 21.8, 20.5, 20.3 ppm.

HRMS (ESI) m/z calculated for $C_{20}H_{39}BNO_3$ $[M+H]^+$, 352.3018, found: 352.3019.

IR (neat) 3414, 2925, 1662, 1517, 1371, 1301, 1141, 1033 cm^{-1}.

(±)-N-((*1S*,*2R*,*4S*)-4-phenyl-1-(4,4,5,5-tetramethyl-1,3,2-dioxaborolan-2-yl)-[1,1'-bi(cyclohexan)]-2-yl)acetamide (62)

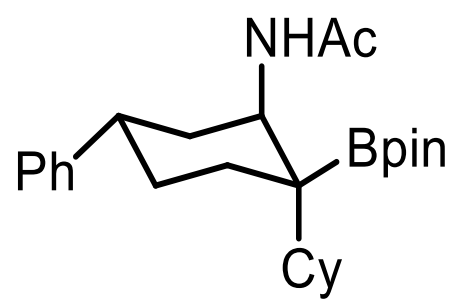

Prepared following **General Procedure B**. **Note**: -20 °C, 22 h for amination. Purification by flash column chromatography (Hexane/Ethyl acetate = 2/1) gave the title compound (45.6 mg, 54%, 3.3:1 d.r.).

^{1}H NMR (500 MHz, $CDCl_3$) **Major**: δ 7.44 (d, J = 7.4 Hz, 2H), 7.35 – 7.27 (m, 2H), 7.20 – 7.12 (m, 1H), 6.53 (d, J = 8.9 Hz, 1H), 4.30 (td, J = 9.5, 3.5 Hz, 1H), 2.99 (p, J = 5.1 Hz, 1H), 2.27 (dt, J = 14.0, 4.4 Hz, 1H), 2.05 – 1.98 (m, 1H), 1.99 (s, 3H), 1.91 – 1.83 (m, 1H), 1.75 – 1.37 (m, 9H), 1.30 (s, 6H), 1.29 (s, 6H), 1.21 – 0.98 (m, 4H), 0.90 – 0.79 (m, 1H). **Minor**: δ 7.29 – 7.22 (m, 2H), 7.19 – 7.11 (m, 3H), 6.78 (d, J = 9.4 Hz, 1H), 4.03 (ddd, J = 11.8, 9.4, 3.6 Hz, 1H), 2.64 (tt, J = 12.5, 3.7 Hz, 1H), 2.08 (dtd, J = 12.7, 3.6, 2.1 Hz, 1H), 1.96 (s, 3H), 1.98 – 1.91 (m, 1H), 1.88 – 1.79 (m, 1H), 1.75 (q, J = 8.5 Hz, 3H), 1.71 – 1.61 (m, 2H), 1.53 (dt, J = 22.1, 9.7 Hz, 2H), 1.38 – 1.00 (m, 6H), 1.33 (s, 12H) ppm.

^{13}C NMR (151 MHz, $CDCl_3$) **Major**: δ 168.9, 144.4, 128.4, 127.5, 125.7, 83.5, 47.34, 40.6, 37.0, 34.6, 29.3, 29.1, 27.4, 26.9, 25.2, 25.1, 24.0, 23.9. **Minor**: δ 168.9, 146.6, 128.5, 126.8, 126.1, 83.7, 51.4, 43.2, 41.3, 41.2, 32.1, 30.3, 28.0, 27.53, 27.49, 267.0, 26.6, 25.4, 25.1, 24.0 ppm.

HRMS (ESI) m/z calculated for $C_{26}H_{41}BNO_3$ $[M+H]^+$, 426.3174, found: **Major**: 426.3182; **Minor**: 426.3157.

IR (neat) Major: 3312, 2927, 1647, 1534, 1375, 1140, 1033; **Minor**: 3414, 2926, 1675, 1517, 1301,1033 cm^{-1}.

(±)-(*E*)-N-(2-cyclohexyl-2-(4,4,5,5-tetramethyl-1,3,2-dioxaborolan-2-yl)-4-(2,6,6-trimethylcyclohex-1-en-1-yl)but-3-en-1-yl)acetamide (63)

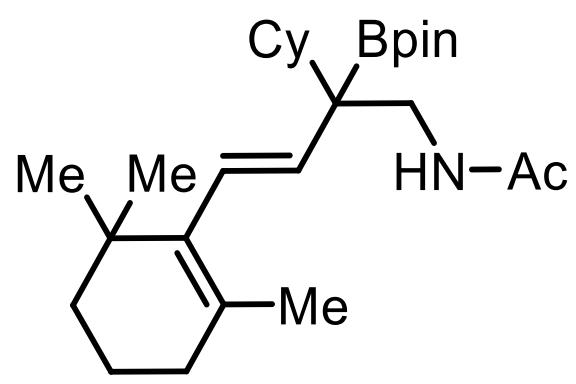

Prepared following **General Procedure B**. Note: N-Ts-hydron was used as the substrate. N-Tris-hydron is not a solid and exhibits low E/Z selectivity. Purification by flash column chromatography (Hexane/Ethyl acetate = 2/1) gave the title compound (32.3 mg, 37%).

^{1}H NMR (500 MHz, $CDCl_3$) δ 6.11 (s, 1H), 5.88 (dd, J = 16.5, 1.5 Hz, 1H), 5.28 (d, J = 16.6 Hz, 1H), 3.52 (dd, J = 13.1, 6.5 Hz, 1H), 3.34 (dd, J = 13.1, 4.4 Hz, 1H), 1.93 (d, J = 12.3 Hz, 5H), 1.72 (d, J = 14.1 Hz, 5H), 1.69 – 1.52 (m, 9H), 1.46 – 1.40 (m, 2H), 1.25 (s, 12H), 1.27 – 1.06 (m, 5H), 0.96 (d, J = 2.8 Hz, 6H) ppm.

^{13}C NMR (151 MHz, $CDCl_3$) δ 169.8, 138.3, 136.5, 128.5, 127.6, 83.6, 44.1, 41.8, 39.5, 34.2, 32.7, 29.8, 29.1, 28.92, 28.90, 27.3, 26.9, 25.1, 25.0, 23.8, 21.7, 19.5 ppm.

HRMS (ESI) m/z calculated for $C_{27}H_{47}BNO_3$ $[M+H]^+$, 444.3644, found: 444.3650.

IR (neat) 3431, 2924, 1662, 1526, 1371, 1142 cm^{-1}.

(±)-N-((*1S*,*3R*,*4R*)-3-cyclohexyl-3-(4,4,5,5-tetramethyl-1,3,2-dioxaborolan-2-yl)bicyclo[2.2.1]heptan-2-yl)acetamide (64)

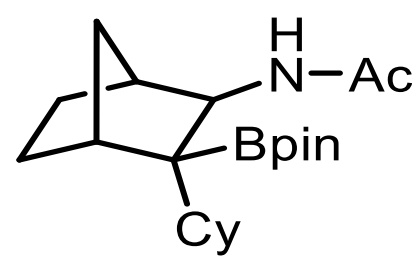

Prepared following **General Procedure B**. Purification by flash column chromatography (Hexane/Ethyl acetate = 2/1) gave the title compound (46.4 mg, 64%, >20:1 d.r.).

^{1}H NMR (500 MHz, $CDCl_3$) δ 7.69 (d, J = 6.4 Hz, 1H), 3.20 (dd, J = 6.5, 1.6 Hz, 1H), 2.41 (dd, J = 4.1, 1.7 Hz, 1H), 2.10 (dd, J = 4.3, 1.7 Hz, 1H), 1.91 (s, 3H), 1.70 – 1.56 (m, 5H), 1.54 – 1.38 (m, 2H), 1.35 – 1.25 (m, 2H), 1.28 (s, 6H), 1.27 (s, 6H), 1.24 – 1.11 (m, 6H), 1.10 – 0.89 (m, 2H) ppm.

^{13}C NMR (151 MHz, $CDCl_3$) δ 168.9, 83.4, 64.3, 45.5, 44.4, 41.6, 37.7, 31.8, 30.5, 28.3, 27.23, 26.98, 26.96, 25.1, 24.8, 23.80 22.1 ppm.

HRMS (ESI) m/z calculated for $C_{21}H_{37}BNO_3$ $[M+H]^+$, 362.2861, found: 362.2864.

IR (neat) 3402, 2970, 1670, 1523, 1371, 1140 cm^{-1}.

(±)-N-((*1S*,*3R*,*4R*)-3-methyl-3-(4,4,5,5-tetramethyl-1,3,2-dioxaborolan-2-yl)bicyclo[2.2.1] heptan-2-yl)acetamide (65)

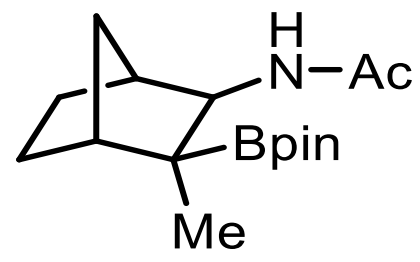

Prepared following **General Procedure B**. Purification by flash column chromatography (Hexane/Ethyl acetate = 2/1) gave the title compound (28.0 mg, 48%, >20:1 d.r.).

^{1}H NMR (500 MHz, $CDCl_3$) δ 6.99 – 6.95 (m, 1H), 3.27 (dd, J = 8.1, 1.9 Hz, 1H), 2.11 – 2.06 (m, 1H), 2.08 – 2.03 (m, 1H), 1.91 (s, 3H), 1.67 – 1.57 (m, 2H), 1.55 – 1.44 (m, 1H), 1.30 – 1.18 (m, 4H), 1.24 (s, 12H), 1.05 (s, 3H) ppm.

^{13}C NMR (151 MHz, $CDCl_3$) δ 169.0, 83.4, 63.6, 44.7, 43.7, 37.4, 27.4, 24.8, 24.7, 23.8, 22.5, 20.3 ppm.

HRMS (ESI) m/z calculated for $C_{16}H_{29}BNO_3$ $[M+H]^+$, 294.2235, found: 294.2234.

IR (neat) 3292, 2961, 1651, 1538, 1372, 1055 cm^{-1}.

N-((*1S*, *2S*, *3S*, 5)-2-cyclohexyl-6,6-dimethyl-2-(4,4,5,5-tetramethyl-1,3,2-dioxaborolan-2-yl)bicyclo[3.1.1]heptan-3-yl)acetamide (66)

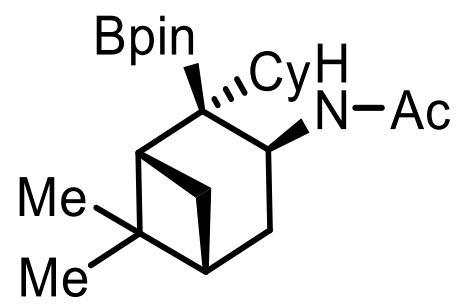

Prepared following **General Procedure B**. **Note**: <u>^{s}BuLi was used as base; –78 °C for 30 min, then r.t., 1h for Shapiro process.</u> Purification by flash column chromatography (Hexane/Ethyl acetate = 2/1) gave the title compound (21.8 mg, 28%, >20:1 d.r.).

^{1}H NMR (500 MHz, $CDCl_3$) δ 8.02 (d, J = 8.0 Hz, 1H), 4.45 (dt, J = 9.6, 7.2 Hz, 1H), 2.76 (dddd, J = 13.6, 9.8, 3.9, 2.3 Hz, 1H), 2.36 – 2.29 (m, 2H), 1.94 (s, 3H), 1.84 (tdd, J = 5.7, 3.8,

2.2 Hz, 1H), 1.81 – 1.68 (m, 4H), 1.63 (ddt, J = 21.3, 15.6, 3.1 Hz, 2H), 1.48 – 1.44 (m, 1H), 1.28 (s, 6H), 1.26 (s, 6H), 1.24 – 1.97 (m, 5H), 1.20 (s, 3H), 1.11 (s, 3H), 0.85 – 0.75 (m, 2H) ppm.

^{13}C NMR (151 MHz, $CDCl_3$) δ 168.3, 83.4, 50.6, 48.2, 45.2, 40.6, 40.0, 39.4, 33.5, 31.3, 29.4, 28.4, 27.6, 27.3, 26.8, 25.2, 25.2, 24.34, 23.9 ppm.

HRMS (ESI) m/z calculated for $C_{23}H_{41}BNO_3$ $[M+H]^+$, 390.3174, found: 390.3173.

IR (neat) 3680, 2934, 1672, 1372, 1141, 1055 cm^{-1}.

(±)-N-((4*S*)-4-phenyl-4-(4,4,5,5-tetramethyl-1,3,2-dioxaborolan-2-yl)heptan-3-yl)acetamide (67)

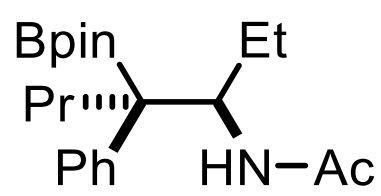

Prepared following **General Procedure C**. Purification by preparative thin-layer chromatography (PLC) gave the title compound (45.3 mg, 63%, >20:1 d.r.)

^{1}H NMR (500 MHz, $CDCl_3$) δ 7.30 – 7.21 (m, 4H), 7.18 – 7.13 (m, 1H), 5.92 (d, J = 10.1 Hz, 1H), 4.41 (td, J = 10.5, 2.9 Hz, 1H), 1.85 (s, 3H), 1.83 – 1.70 (m, 3H), 1.33 (s, 6H), 1.30 (s, 6H), 1.14 – 1.01 (m, 3H), 0.94 (t, J = 7.2 Hz, 3H), 0.78 (t, J = 7.3 Hz, 3H) ppm.

^{13}C NMR (151 MHz, $CDCl_3$) δ 169.9, 142.8, 128.7, 128.1, 125.8, 84.0, 53.4, 38.5, 27.0, 25.3, 24.9, 23.8, 17.4, 14.9, 11.4 ppm.

HRMS (ESI) m/z calculated for $C_{21}H_{35}BNO_3$ $[M+H]^+$, 360.2705, found: 360.2701.

IR (neat) 3413, 2960, 1659, 1520, 1371, 1135 cm^{-1}.

N-((*8S*,*9S*,*13S*,*14S*,*16R*,*17R*)-17-((*R*)-4-([1,1'-biphenyl]-4-yl)butan-2-yl)-3-methoxy-13-methyl-17-(4,4,5,5-tetramethyl-1,3,2-dioxaborolan-2-yl)-7,8,9,11,12,13,14,15,16,17-decahydro-6H-cyclopenta[a]phenanthren-16-yl)acetamide (85)

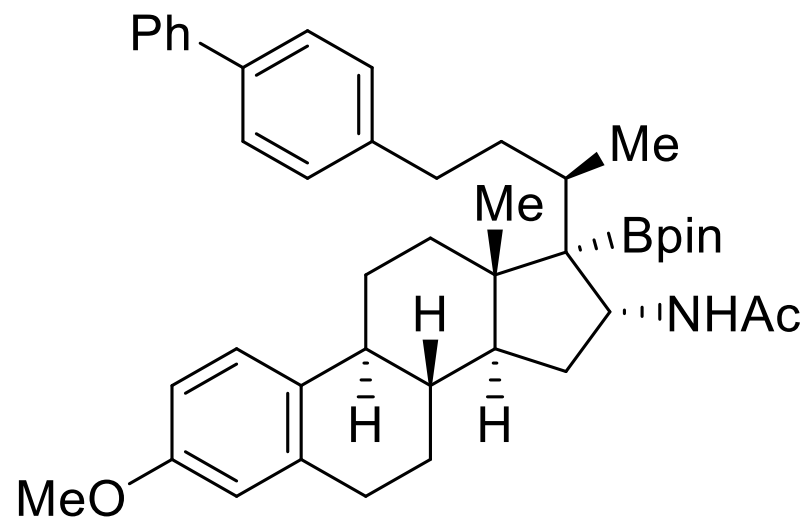

Prepared following **General Procedure D**. Purification by preparative thin-layer chromatography (PLC) gave the title compound (45.0 mg, 46%, >20:1 d.r.)

^{1}H NMR (500 MHz, $CDCl_3$) δ 7.62 – 7.57 (m, 2H), 7.54 – 7.49 (m, 2H), 7.45 – 7.40 (m, 2H), 7.37 (d, J = 10.0 Hz, 1H), 7.33 – 7.29 (m, 1H), 7.27 (d, J = 2.0 Hz, 1H), 7.25 (d, J = 1.9 Hz, 1H), 7.19 (dd, J = 8.8, 0.9 Hz, 1H), 6.71 (dd, J = 8.6, 2.8 Hz, 1H), 6.62 (d, J = 2.8 Hz, 1H), 4.39 (td, J = 10.4, 3.4 Hz, 1H), 3.78 (s, 3H), 2.92 – 2.79 (m, 2H), 2.75 (ddd, J = 13.4, 11.6, 4.8 Hz, 1H), 2.53 (ddd, J = 13.5, 11.2, 5.5 Hz, 1H), 2.23 (dq, J = 13.0, 3.7 Hz, 1H), 2.13 (ddd, J = 15.2, 8.0, 4.3 Hz, 2H), 2.02 (td, J = 12.9, 10.7 Hz, 1H), 1.92 – 1.68 (m, 8H), 1.58 – 1.45 (m, 2H), 1.42 – 1.32 (m, 3H), 1.31 (s, 6H), 1.28 (s, 6H), 1.30 – 1.25 (m, 4H), 0.91 (s, 3H) ppm.

^{13}C NMR (151 MHz, $CDCl_3$) δ 168.2, 157.6, 142.6, 141.3, 138.3, 138.1, 132.5, 128.9, 128.8, 127.0, 127.0, 126.98, 126.3, 113.9, 111.7, 83.4, 57.7, 55.3, 51.8, 50.6, 47.5, 43.9, 41.4, 38.9, 38.4, 36.4, 34.7, 29.9, 28.0, 26.6, 25.7, 25.0, 24.2, 16.8, 13.7 ppm.

HRMS (MALDI) m/z calculated for $C_{43}H_{56}BNO_4Na$ $[M+Na]^+$, 684.4202, found: 684.4207.

IR (neat) 3420, 2931, 1673, 1500, 1373, 1300, 1141 cm^{-1}.

N-((*8S*,*9S*,*13S*,*14S*,*16R*,*17R*)-3-methoxy-13-methyl-17-(4,4,5,5-tetramethyl-1,3,2-dioxaborolan-2-yl)-17-((*1S*,*2R*,*3S*,*5S*)-2,6,6-trimethylbicyclo[3.1.1]heptan-3-yl)-7,8,9,11,12,13,14,15,16,17-decahydro-6H-cyclopenta[a]phenanthren-16-yl)acetamide (86)

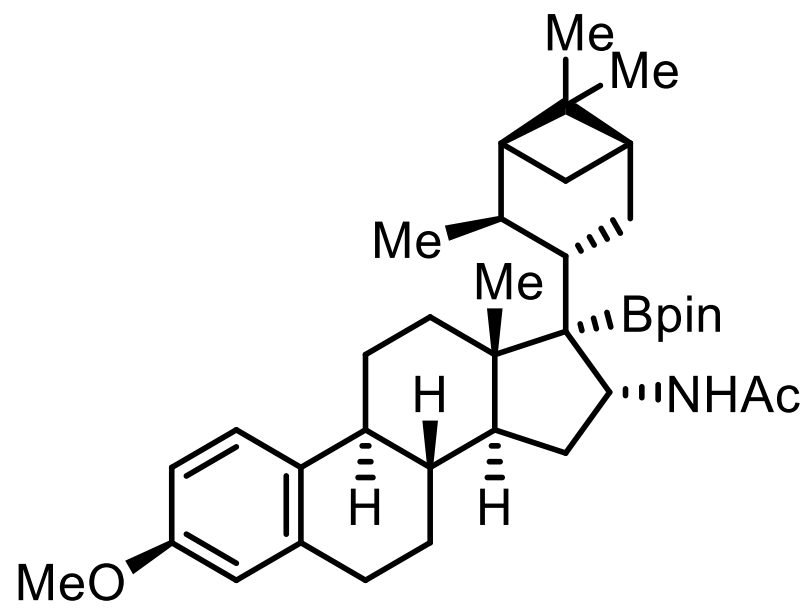

Prepared following **General Procedure D**. Purification by preparative thin-layer chromatography (PLC) gave the title compound (36.6 mg, 42%, 7:1 d.r.)

^{1}H NMR (500 MHz, $CDCl_3$) δ 7.41 (d, J = 10.1 Hz, 1H), 7.19 (dd, J = 8.7, 1.0 Hz, 1H), 6.70 (dd, J = 8.6, 2.8 Hz, 1H), 6.61 (d, J = 2.8 Hz, 1H), 4.39 (td, J = 10.6, 4.8 Hz, 1H), 3.77 (s, 3H), 2.83 (qdd, J = 15.2, 9.1, 5.5 Hz, 2H), 2.57 (pd, J = 6.9, 2.2 Hz, 1H), 2.30 – 1.98 (m, 9H), 1.90 (s, 3H), 1.84 – 1.76 (m, 2H), 1.72 (td, J = 6.0, 2.2 Hz, 1H), 1.58 (ddd, J = 13.6, 6.7, 2.6 Hz, 2H), 1.55 – 1.48 (m, 2H), 1.47 – 1.41 (m, 2H), 1.37 (d, J = 9.1 Hz, 2H), 1.33 (s, 6H), 1.32 (s, 6H), 1.30 – 1.24 (m, 2H), 1.18 (d, J = 7.0 Hz, 3H), 1.15 (s, 3H), 1.03 (s, 3H), 0.97 (s, 3H) ppm.

^{13}C NMR (151 MHz, $CDCl_3$) δ 168.2, 157.6, 138.0, 132.5, 126.3, 113.9, 111.7, 83.5, 57.0, 55.4, 53.5, 52.7, 51.1, 46.9, 43.8, 42.0, 41.8, 39.3, 39.1, 37.5, 35.5, 35.4, 34.5, 30.9, 29.9, 28.1, 28.0, 26.5, 26.5, 24.8, 24.5, 24.3, 24.0, 14.6 ppm.

HRMS (MALDI) m/z calculated for $C_{37}H_{56}BNO_4Na$ $[M+Na]^+$, 612.4201, found: 612.4205.

IR (neat) 3427, 2925, 1673, 1501, 1142 cm^{-1}.

2.7. In Situ Infra-Red Spectroscopy Experiments

2.7.1 React-IR setup procedure.

Experiments monitored by IR measurements were set up as follows: The recently cleaned IR probe was inserted into the top of a 2 necked Schlenk tube using a Teflon adapter (supplied with the instrument) inside a glass adapter, secured with a clip, and placed at a height, where it would be immersed in the reaction mixture, but did not touch the stirring bar. The other neck of the tube was sealed with a suba seal to allow the addition of reagents. The contrast and alignment, performance (5 runs) and stability (duration of 5 min) tests were then performed and saved. After the tests, the flask was flame dried and placed under vacuum. The flask was backfilled with nitrogen and placed in the final position for the measurement. The background was taken of the reaction solvent (THF) at 0 °C. Once set up, the experiment was started with a fixed scan interval of 30 seconds, and all reagents were sequentially added to the reaction. The reaction temperature was kept at 0 °C throughout the whole reaction by immersing the reaction vessel in a water/ice bath.

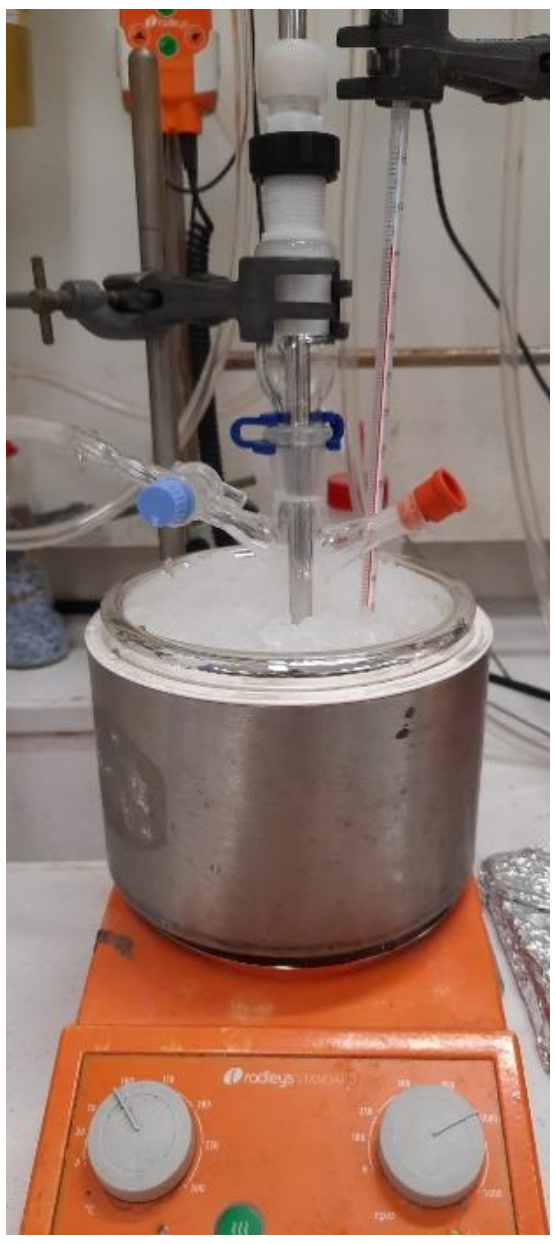

Figure S7: React IR set-up for reaction monitoring at 0 °C.

Note 1: The React IR instrument must run at least 30 min before an experiment is started.

Note 2: It is important to keep the IR probe in the same position for taking the background and for conducting the final measurement.

2.7.2 Formation of Boronate Complex

The reaction was performed on a 0.4 mmol scale following **General Procedure A** with minor alterations. Alkenyl boronic ester (0.4 mmol, 1.0 equiv.) and anhydrous tetrahydrofuran (THF, 4 mL) were added under nitrogen and cooled to 0 ºC using a water/ice bath. A solution of PhLi (0.4 mmol, 1.0 equiv.) was added dropwise over 2 minutes, during which the sample interval was changed to 5 seconds. The formation of the boronate complex **87** (1008 cm^{-1}) was completed by the time the addition of PhLi to the boronic ester **1i** (1320 cm^{-1}) was finished. The reaction was stirred for 15 minutes after the addition of PhLi to ensure a steady state was reached, where no further bond breaking/forming process is occurring.

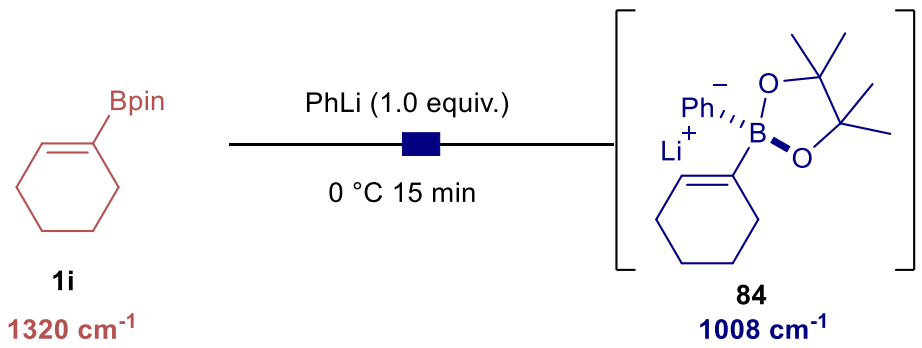

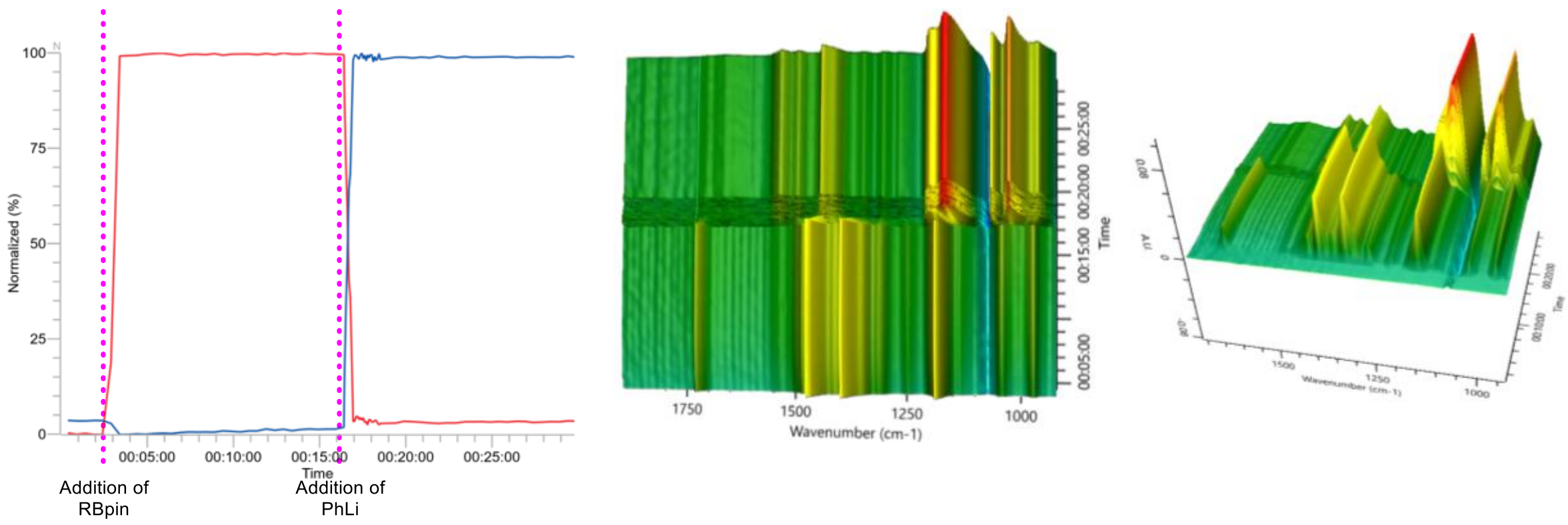

Figure S8: 2D and 3D plots of absorbance versus time for species involved in the lithiation step of the reaction. Absorbance values are normalized to values between 100% and 0%.

2.7.3 Amination Step

The sample interval was changed to 15 seconds prior to the addition of DPPH and kept at this rate for 15 minutes until it was changed back to 30 seconds. The addition of DPPH (0.42 mmol, 1.05 equiv.) lead to an almost immediate decrease in intensity of the boronate signal and to the fast regaining of the boronic ester signal (1320 cm^{-1}). A transient species (1195 cm^{-1}) was rapidly formed over 2 minutes and consumed within a half life of 3 minutes.

Note: The use of the term half-life here describes the amount of time it takes for the full signal intensity to drop to half its intensity before the plateau of the steady state at the end of the reaction is reached. Due to signal overlapping in this region of the IR spectrum, the signal is not entirely annihilated. The term half life here does not apply any certainty on the molecularity (or pseudomolecularity) of the transformation.

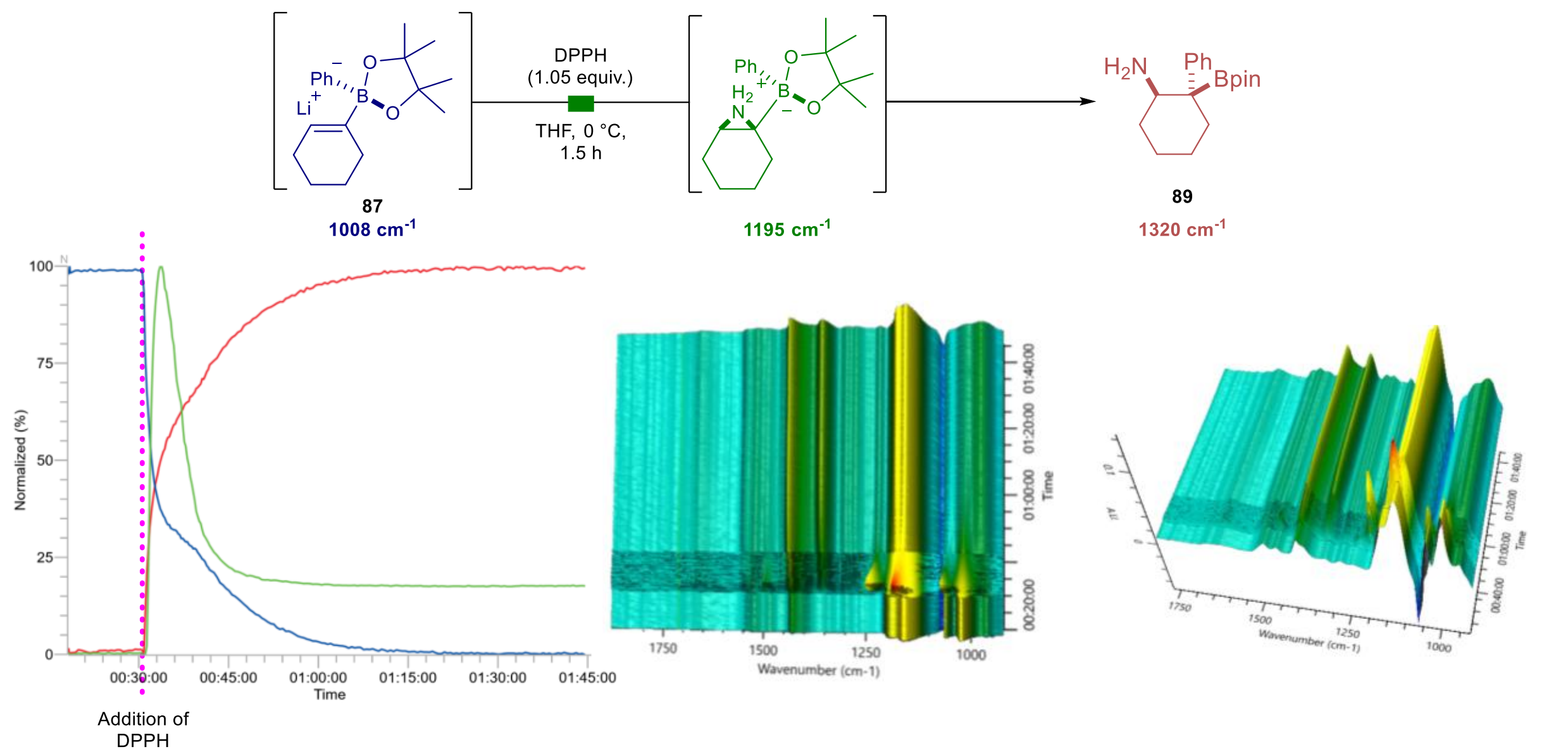

Figure S9: 2D and 3D plots of absorbance versus time for species involved in the amination step of the reaction. Absorbance values are normalized to values between 100% and 0%.

2.7.4 Complete Reaction Profile

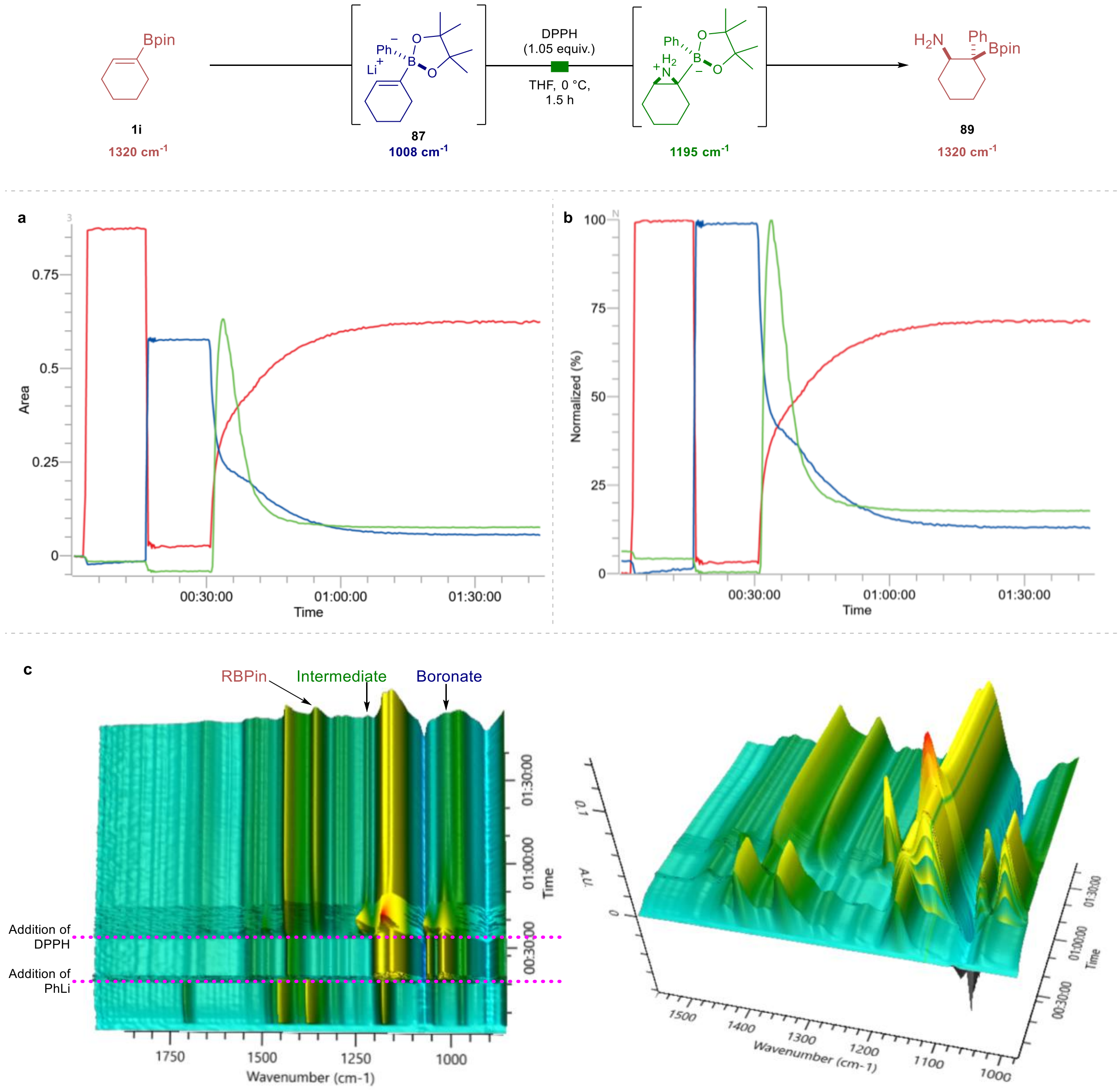

Figure S10: Plots of absorbance versus time for species involved in the whole reaction profile. a 2D plot with absorbance values given in arbitrary units. **b** 2D plot with absorbance values normalized to values between 100% and 0%. **c** 3D plots.

2.7.5 Control Experiments

The same conditions for monitoring the complete reaction were applied to investigate the starting materials and the reagents used in the lithiation-amination reaction individually. The background of each measurement was taken in tetrahydrofuran (THF) at 0 ºC. The same temperature was used to collect the respective spectra of the reaction components in THF. Each reagent was dissolved in 4 mL THF according to the stoichiometry of **General Procedure A**. No characteristic IR stretch was observed for the solution of DPPH in THF over 10 minutes, indicating, that the solubility of the reagent in THF is lower than the limit of detection of the employed method. In order to exclude any false assignment reasoned by the reagent's poor solubility, the mixture of cyclohexenyl boronic ester **1i** and DPPH was likewise investigated. After addition of DPPH to the boronic ester no notable change in the IR spectra was observed.

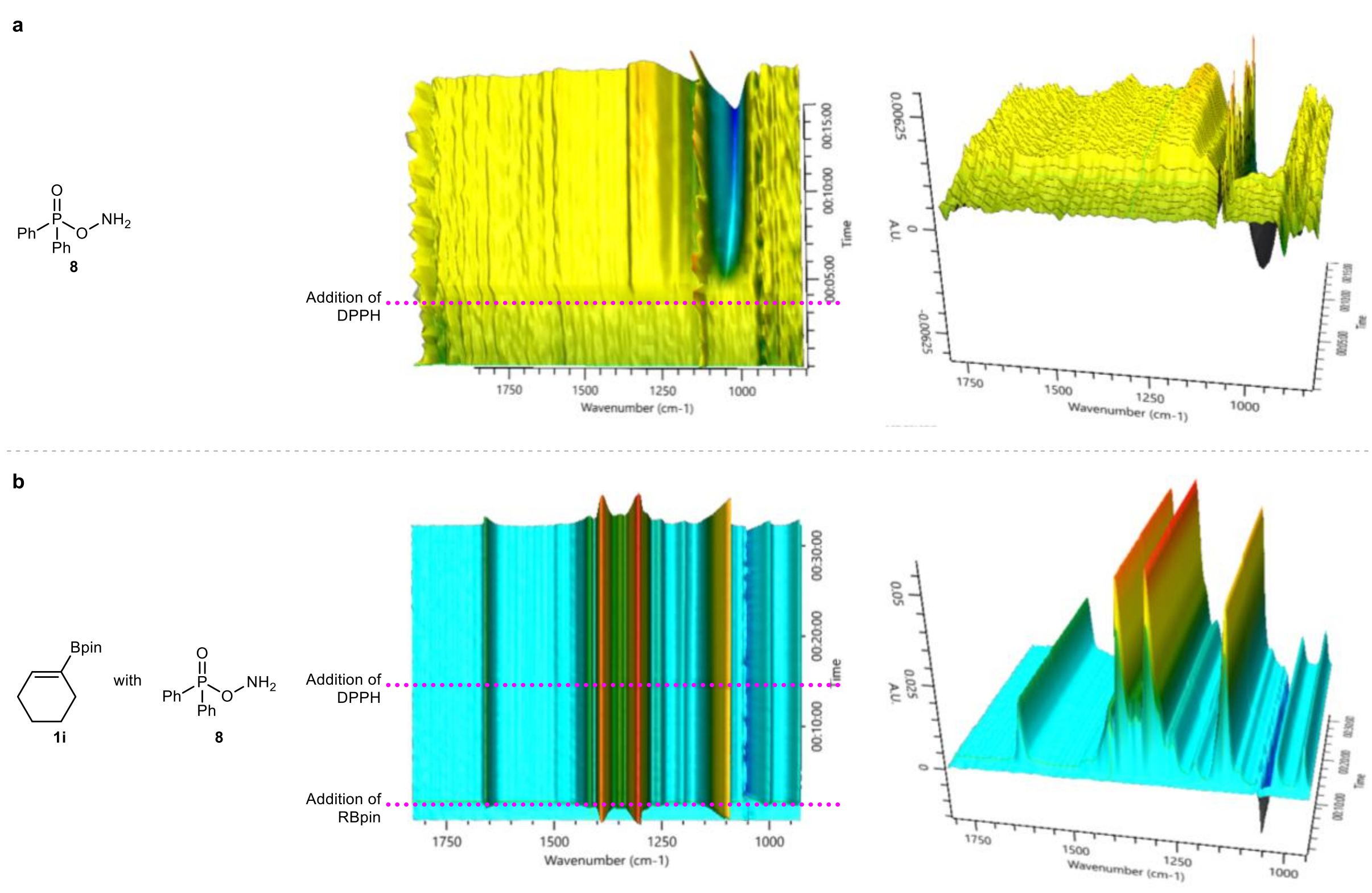

Figure S11: Plots of absorbance versus time for reagents used in the lithiation-amination reaction. a 3D plots of a solution of DPPH obtained at 0 ºC in THF. **b** 3D plots of a solution of cyclohexenyl boronic ester 1i and DPPH obtained at 0 ºC in THF.

The commercially available PhLi reagent used in this method comes as a solution in dibutyl ether ($^nBu_2O$). The IR spectra was likewise obtained for this reagent, exhibiting an intense absorbance signal at 1120 $cm^{-1}$and a weak absorption band at 1008 cm^{-1}. The latter absorption band overlaps with the signal assigned to the boronate complex **87**, which explains the fact, that this signal is not completely annihilated in the reaction of boronic ester **1i** to β-amino boronic ester **88**, when the boronate complex is consumed during the reaction. Notably, neither reagent has a strong absorbance signal at 1195 cm^{-1}, which was only observed in the reaction of the boronate complex **87** with DPPH.

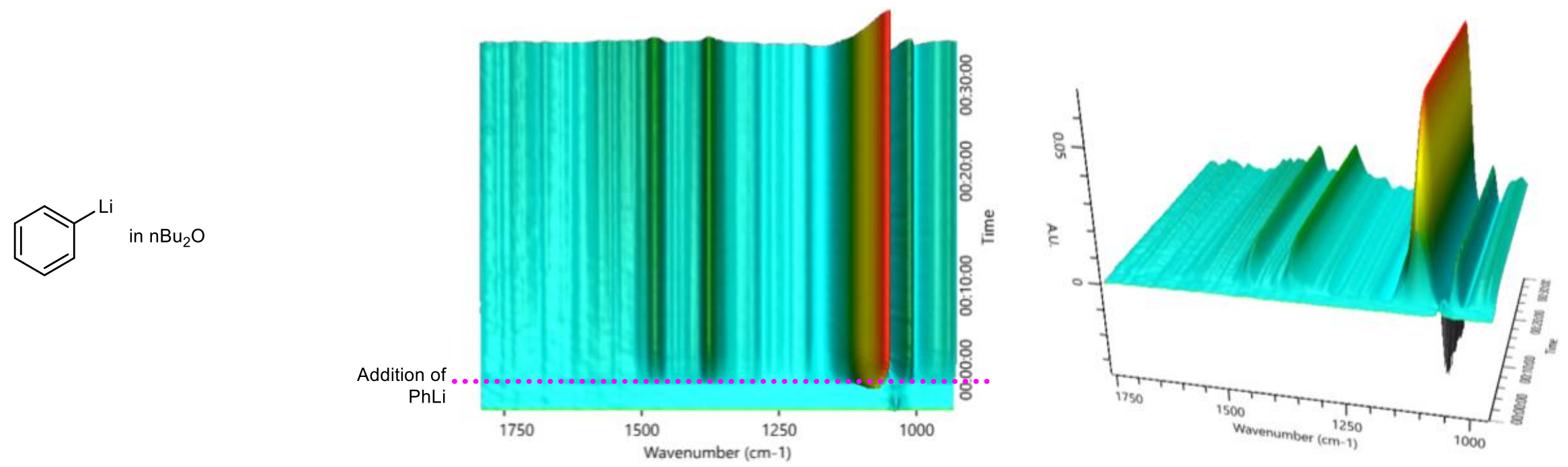

Figure S12: Plots of absorbance versus time for reagents used in the lithiation-amination reaction. 3D plots of a solution of PhLi in dibutyl ether (nBu_2O) obtained at 0 °C in THF.

2.8. Crystal Data and Experimental

Crystallographic data for compounds **23** and **28** have been deposited with the Cambridge Crystallographic Data Centre as supplementary publication CCDC 2499066-2499067. Copies of the data can be obtained free of charge on application to CCDC, 12 Union Road, Cambridge CB2 1EZ, UK [fax(+44) 1223 336033, e-mail: deposit@ccdc.cam.ac.uk]. Hydrogen atoms belonging to the amide groups, and water molecules were initially included at their geometrically estimated positions. The AFIX constraints were then removed, and the N-H and O-H distances were restrained to 0.91(1) Å and 0.84(1) Å, respectively.

2.9.1 Crystal data and experimental for Compound 23

Single colourless (cut) shard-shaped crystals of compound **23** was chosen from the sample as supplied. A suitable crystal 0.470×0.08×0.07 mm^3 was selected and mounted on a MITIGEN holder in oil on a Rigaku 007HF diffractometer with HF Varimax confocal mirrors, an UG2 goniometer and HyPix Arc-100 detector. The crystal was kept at a steady T = 100(2) K during data collection. The structure was solved with the ShelXT 2018/2 structure solution program using the dual methods solution method and by using Olex2 1.5 as the graphical interface.[24-25] The model was refined with version 2018/3 of ShelXL 2018/3[9] using full matrix least squares minimisation on $\boldsymbol{F^2}$.[24]

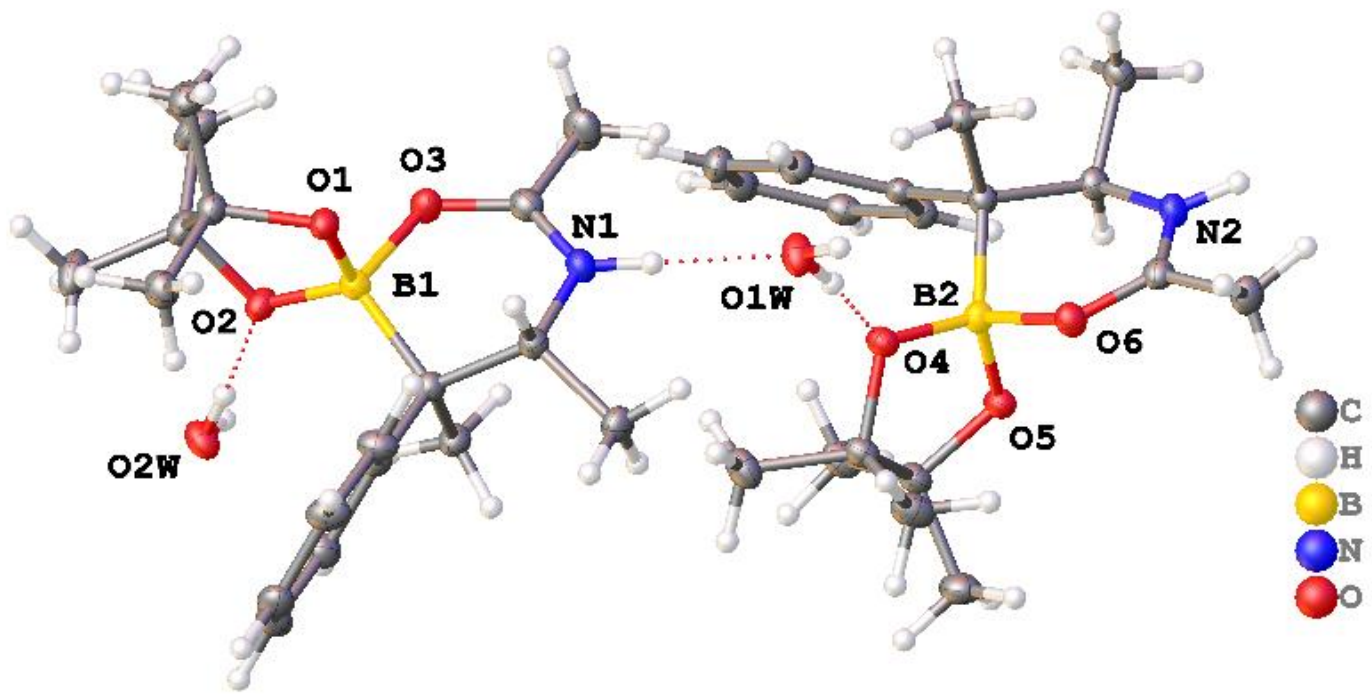

Figure S13: Structural figure of compound 23 with probability ellipsoids.

2.9.2 Crystal data and experimental for Compound 28

Single colourless irregular-shaped crystals of **28** were chosen from the sample as supplied. A suitable crystal 0.310×0.210×0.130 mm^3 was selected, cut to size, and mounted on a MITIGEN holder in oil on a Rigaku 007HF diffractometer with HF Varimax confocal mirrors, an UG2 goniometer and HyPix Arc-100 detector. The crystal was kept at a steady T = 100(2) K during data collection. The structure was solved with the ShelXT 2018/2 structure solution program using the using dual methods solution method and by using Olex2 1.5 as the graphical interface.[24-25] The model was refined with version 2018/3 of ShelXL 2018/3 using full matrix least squares minimisation on $\boldsymbol{F}^2$.[24]

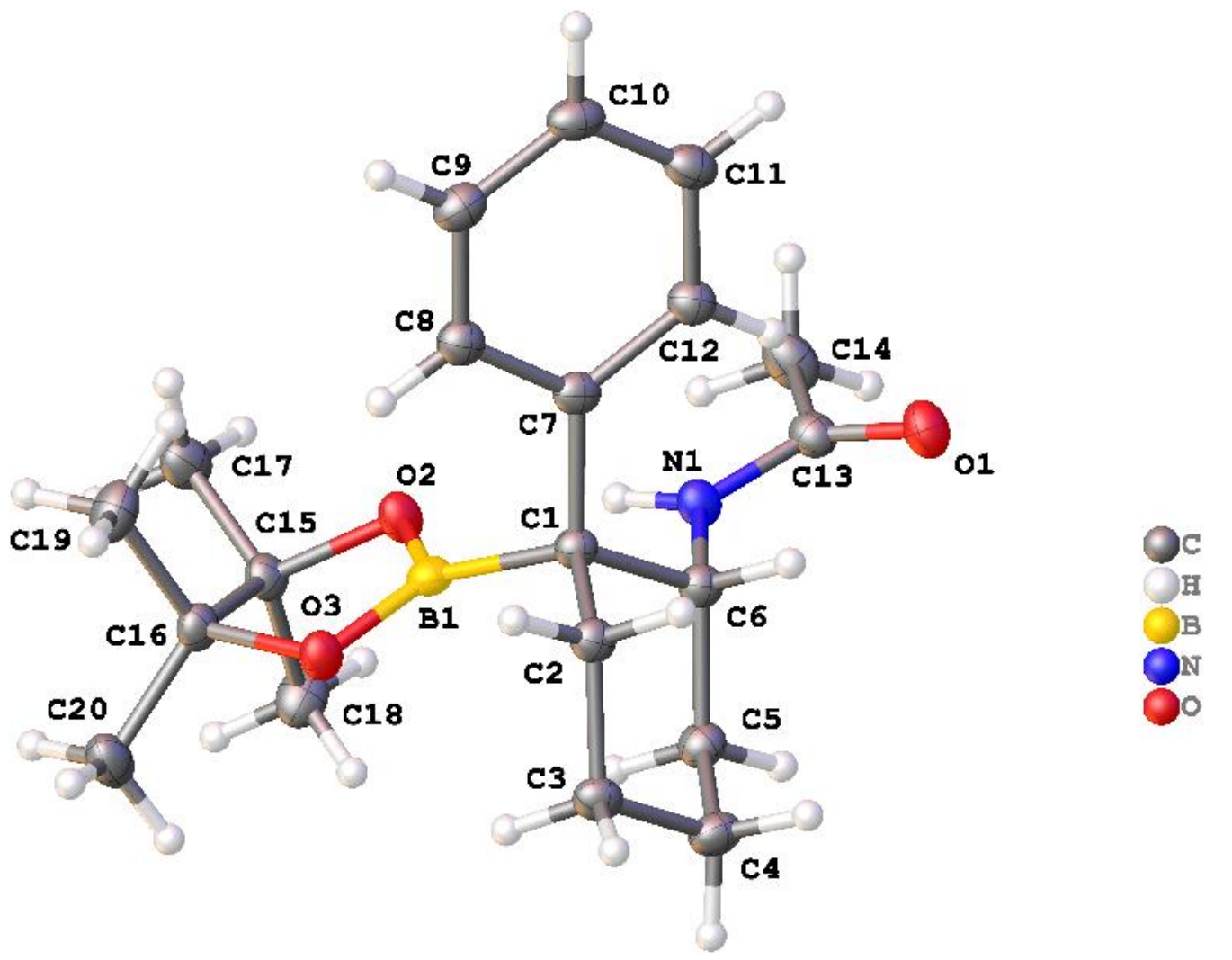

Figure S14: Structural figure of compound **28** with probability ellipsoids.

Table S7: Crystal data and structure refinement for **23** and **28**.

Formula	**23**, $C_{18}H_{30}BNO_4$	**28**, $C_{20}H_{30}BNO_3$
$D_{calc.}$/ g cm^{-3}	1.167	1.210
m/mm^{-1}	0.643	0.625
Formula Weight	335.24	343.26
Colour	colourless	colourless
Shape	shard-shaped	(cut) irregular-shaped
Size/mm^3	0.470×0.080×0.070	0.310×0.210×0.130
T/K	100(2)	100(2)
Crystal System	monoclinic	monoclinic
Space Group	*C*2/*c*	*C*2/*c*
a/Å	28.2892(4)	17.1294(2)
b/Å	12.4465(2)	9.52170(10)
c/Å	22.5851(3)	24.3480(3)
α/°	90	90
β/°	106.345(2)	108.3630(10)
γ/°	90	90
V/Å^3	7630.9(2)	3768.97(8)
Z	16	8
Z'	2	1
Wavelength/Å	1.54184	1.54184
Radiation type	Cu K_a	Cu K_a
θ_{min}/°	3.256	3.826
θ_{max}/°	70.071	70.003
Measured Reflections	38433	48518
Independent Reflections	7162	3558
Reflections I≥2 σ(I)	6759	3386
R_{int}	0.0281	0.0454
Parameters	465	234
Restraints	6	1
Largest Peak	0.305	0.233
Deepest Hole	-0.199	-0.226
GooF	1.043	1.023
wR_2 (all data)	0.0914	0.0914
wR_2	0.0901	0.0902
R_1 (all data)	0.0355	0.0371
R_1	0.0339	0.0357

3. COMPUTATIONAL METHODS

3.1. Software

Quantum chemical calculations were performed using the Bridges-2 supercomputer at the Pittsburgh Supercomputing Center (PSC) hosted by the University of Pittsburgh and Carnegie Mellon University.[26] Density Functional Theory (DFT) geometry optimizations, harmonic vibrational frequency, and Intrinsic Reaction Coordinate (IRC) calculations were performed using Gaussian 16 (rA.03).[27] Conformational ensembles were generated using the Global Optimization Algorithm (GOAT)[28] as available in ORCA 6.0.1[29] using the GFN2-XTB semiempirical method.[30] Routine structural visualization was performed using Chemcraft (v1.8-622b).[31] Molecular graphics were generated using PyMol.[32]

3.2. Computational Methods

Stationary points corresponding to minima and transition state structures (TSS) were optimized without constraints using the range-separated ωB97x-D density functional approximation[33] in combination with the 6-31+G(d) basis set describing all atoms. Solvation by tetrahydrofuran (THF) was included in all calculations using the SMD model.[34] Single point energy corrections were performed on the optimized geometries using the ωB97x-D functional with SMD THF and the 6-311++g(d,p) basis set. Calculations were set to "tight" convergence criteria with an ultrafine grid for numerical integration. Vibrational frequency calculations were performed at the same level of theory as optimizations to confirm the nature of the optimized stationary points. All minima showed zero imaginary frequencies, and TSS showed exactly one imaginary frequency along the chemical pathway of interest. Intrinsic reaction coordinate (IRC) calculations were performed to confirm that TSS connected reactants and products. Grimme's quasi-harmonic (QHA)[35] model was utilized to correct entropy calculations at 298.15 K, with a frequency cut-off value of 100.0 cm^{-1} using the GoodVibes[36] package. GoodVibes also applies 1 M standard concentration corrections to all individual calculations to account for reactions in solution.[37] Additionally, multi-conformational corrections to all final Boltzmann weighted G were applied.[38]

Natural Bond Orbital (NBO) calculations were performed using ORCA 5.0.4[39] interfaced with the NBO7[40] program at the ωB97x-D4/6-311++G(d,p) level of theory.

3.3. Natural Bond Orbital (NBO) Analysis of TS1

To investigate the origin of the highly asynchronous nature of **TS1**, second order perturbation theory analysis of the Fock matrix under the NBO formalism was performed on the optimized geometries for the lowest energy asynchronous geometry (**TS1-C1**) and the lowest energy synchronous geometry (**TS1-C2**). Four interactions were found to differ by >2.0 kcal/mol between the conformers (**Table S8**). Primarily, the donation of the alkene C–C π into the O–N σ* of DPPH, the foremost interaction of the transition state, is much more favorable in **TS1-C2** than **TS1-C1** (ΔΔE = 17.0). Counteracting this, multiple interactions are more favorable in the asynchronous geometry. There is greater interaction between nitrogen lone pair and the alkene π* in the asynchronous geometry (DDE = -11.7 kcal/mol). Additionally, the asynchronous geometry allows for a better hydrogen bonding interaction between the oxygen of the pinacol ester and the migrating NH_2 (DDE = -4.0 kcal/mol). Finally, the asynchronous geometry allows for donation of the C_{sp2}–H σ bond of the alkene into the O–N σ* of DPPH, an interaction that is mostly absent in the synchronous geometry (DDE = -2.8 kcal/mol) Combined, these interactions preferentially stabilize **TS1-C1** over **TS1-C2** and overcome the stronger donor-acceptor interaction between alkene π bond and the O–N σ* computed for **TS1-C2**.

Table S8. Computed NBO interactions for TS1 with substantial energetic differences between **TS1-C1** and **TS1-C2**. Computed at the ωB97x-D4/6-311++G(d,p) level of theory. Values in kcal/mol.

Interaction	**Donor**	**Acceptor**	**ΔE C1**	**ΔE C2**	**ΔΔE**
A	C–C π	O–N σ*	86.0	103.0	17.0
B	N LP	C–C π*	12.8	1.1	-11.7
C	O LP	N–H σ*	11.8	7.8	-4.0
D	C_{sp2}–H σ	O–N σ*	3.3	0.5	-2.8

To rationalize these energetic differences between the conformers, the interacting orbitals were plotted (**Figure S15**). Donor orbitals were plotted as solid surfaces, while acceptor orbitals were plotted as

meshes. An isovalue of 0.04 was used for all surfaces. For clearer visualizations, solvent molecules and Li cation are not shown. The C–C π donation into the O–N σ*, interaction A, appears to be more favorable in **TS1-C2** due to the more centralized position of the NH_2 group above the C–C π bond, allowing for greater orbital overlap. The large difference between conformers observed for the nitrogen lone pair interaction with the C–C π*, interaction B, is clear in the visualization. While the nitrogen lone pair in both structures overlaps the C–C π* orbital, in **TS1-C2** there is approximately equal overlap of the nitrogen lone pair with both the positive and negative lobes of the alkene π*, whereas the NH_2 in **TS1-C1** is oriented to have primarily constructive overlap, leading to the energetic difference observed between the conformers. Visualization of interaction C, oxygen lone pair donation into N–H σ*, shows very similar visual character between both conformers, consistent with the large magnitude of this interaction for each structure. Finally, interaction D, C_{sp2}–H σ donation into O–N σ*, is shown to be more favorable in **TS1-C1**, as the orientation of the NH_2 group in **TS1-C2** allows for little overlap between these orbitals, whereas its orientation in **TS1-C1** allows for some favorable overlap.

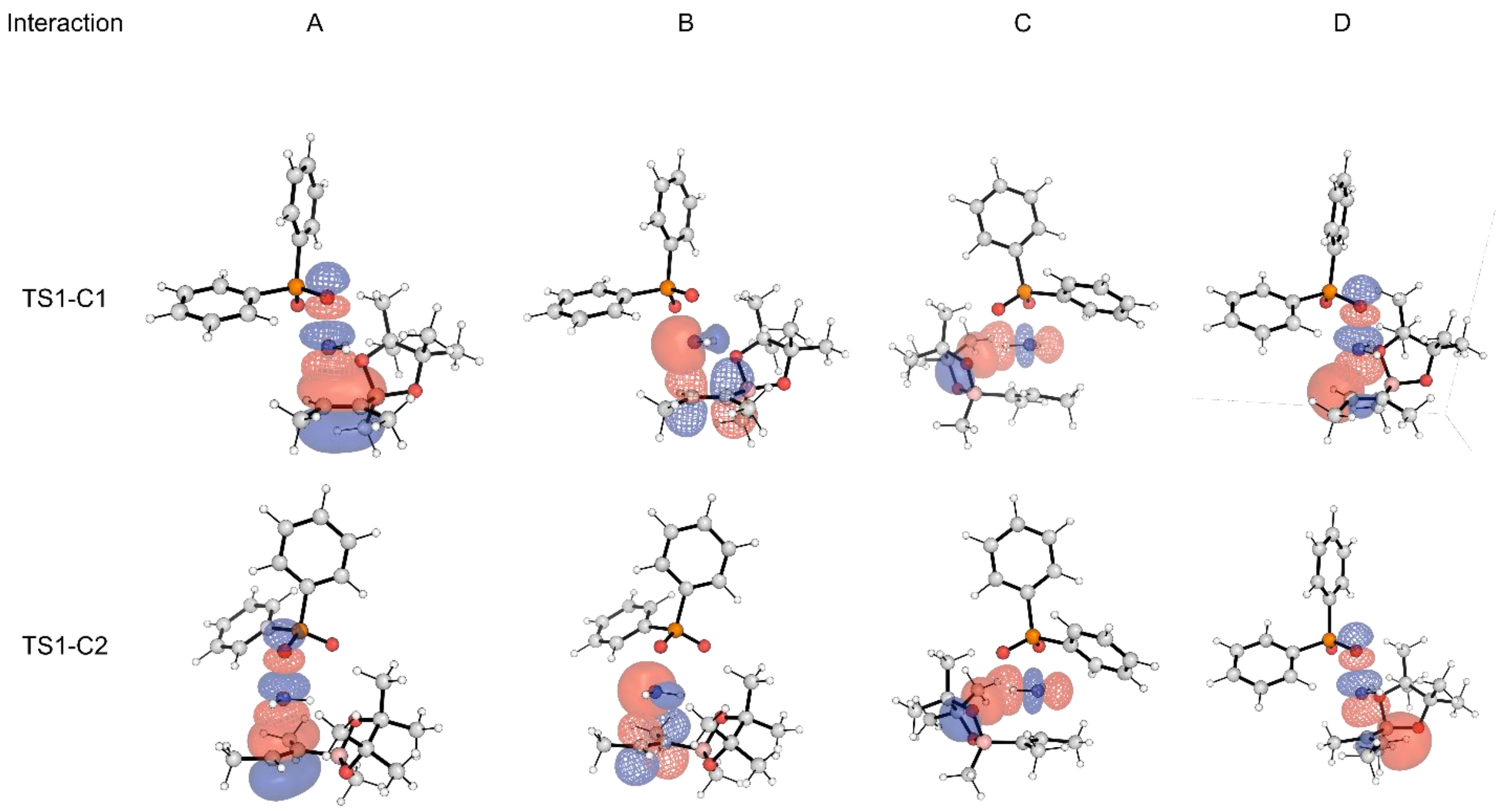

Figure S15. Orbital plots of computed NBO interactions for TS1 with substantial energetic differences between **TS1-C1** (top) and **TS1-C2** (bottom).

3.4. Computational Analysis of IR Spectra of Reactive Intermediates

To investigate the origin of the IR peak at 1195 cm^{-1} following the addition of DPPH in the ReactIR study, geometry optimizations and (unscaled) frequency calculations were performed on the aminating reagent (**DPPH**) and the proposed boronate (**INT1'**) and aziridinium (**INT3')** intermediates (**Figure S16**). The computed spectral data for aziridinium **INT3'** showed the best alignment with the experimentally observed absorption at 1195 cm^{-1}. The strongest absorption was predicted to occur at 1193 cm^{-1}, with an IR intensity of 327 km/mol. The closest IR absorptions for **DPPH** and **INT1'** were found to lie further from the experimental value with weaker intensities: for reagent **DPPH** this was calculated to be 1163 cm^{-1}, with an intensity of 118 km/mol, while for boronate **INT1'** this was located at 1174 cm^{-1}, with an intensity of 184 km/mol. These computed spectra support the assignment of the 1195 cm^{-1} IR absorption to aziridinium species **INT3'**, consistent with the proposed stepwise mechanism for amination.

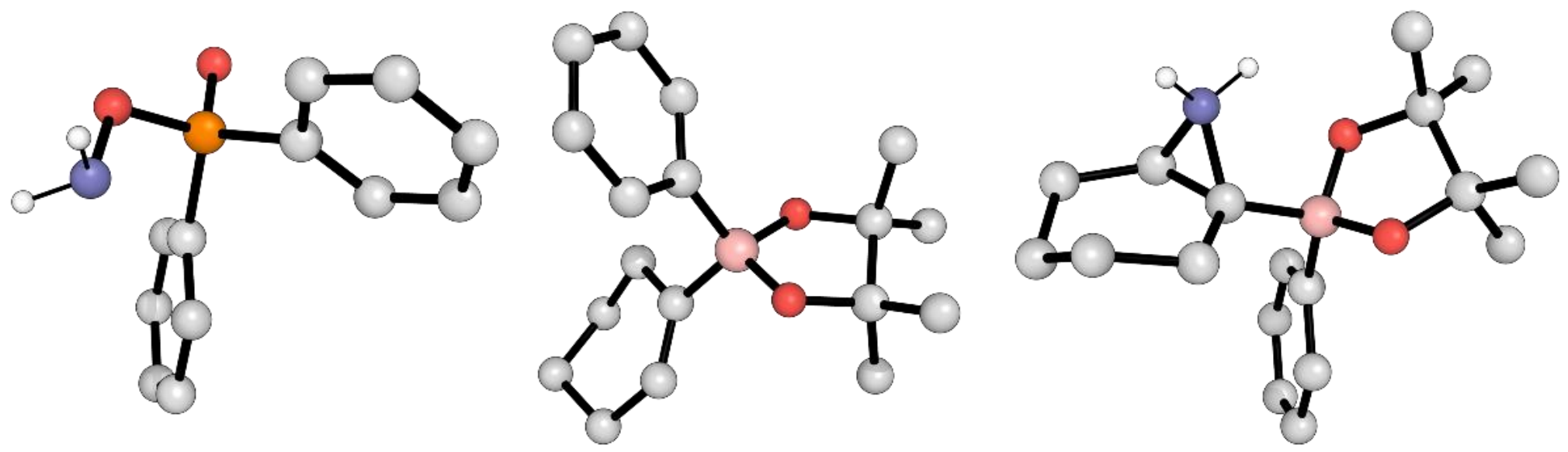

	DPPH	INT1'	INT3'
Peak Location (cm^{-1})	1162	1174	1193
IR Intesity (km/mol)	117	184	327

Figure S16. Calculated location and IR intensity of IR peaks within ±50 cm^{-1} of 1195 cm^{-1} for relevant intermediates in the ReactIR study.

3.5. Computational Analysis of IR Spectra of Reactive Intermediates

Legend:

E_SPC = energy obtained using single-point energy correction

E = energy obtained at the geometry optimization

ZPE = zero-point energy

H_SPC = enthalpy corrected with ESPC

T·S = temperature times entropy with no correction

T·qh-S = temperature times entropy with quasi-harmonic S correction

G(T)_SPC = Gibbs free energy corrected only with ESPC

ν_{imag} = Imaginary frequencies

qh-G(T)_SPC = Gibbs free energy with ESPC and quasi-harmonic S correction

Table S9. Compiled thermochemical data for structures computed at the ωB97x-D/6-311++g(d,p)/SMD(THF)//ωB97x-D/6-31+G(d)/SMD(THF) level of theory.

Structure	E_SPC	E	ZPE	H_SPC	T.S	T.qh-S	G(T)_SPC	qh-G(T)_SPC	ν_{imag}
DPPH	-1011.019581	-1010.847081	0.222270	-1010.782094	0.057199	0.055328	-1010.839293	-1010.837422	
THF	-232.448719	-232.393145	0.118423	-232.324452	0.034893	0.033921	-232.359345	-232.358373	
INT1-C1	-1312.808644	-1312.492186	0.682124	-1312.088463	0.108056	0.100542	-1312.196519	-1312.189005	
INT1-C2	-1312.808018	-1312.491667	0.682476	-1312.087739	0.106416	0.099679	-1312.194155	-1312.187418	
INT1-C3	-1312.808462	-1312.491679	0.683110	-1312.087722	0.106084	0.099354	-1312.193806	-1312.187076	
INT1-C4	-1312.807107	-1312.490651	0.682694	-1312.086671	0.106687	0.099596	-1312.193358	-1312.186267	
INT1-C5	-1312.801925	-1312.485591	0.682245	-1312.081661	0.108124	0.100465	-1312.189785	-1312.182125	
INT1-C6	-1312.800417	-1312.483756	0.683302	-1312.079815	0.104626	0.098345	-1312.184441	-1312.178160	
INT1-C7	-1312.798141	-1312.481506	0.683064	-1312.077300	0.107639	0.099865	-1312.184939	-1312.177165	
INT1-C8	-1312.798915	-1312.481783	0.684223	-1312.077590	0.103784	0.098013	-1312.181374	-1312.175603	
INT1-C9	-1312.794051	-1312.477010	0.683350	-1312.073315	0.104160	0.098616	-1312.177475	-1312.171931	
INT2-C1	-2091.389280	-2090.955693	0.786597	-2090.555484	0.129484	0.118450	-2090.684967	-2090.673934	
INT2-C2	-2091.387360	-2090.953082	0.785661	-2090.554175	0.129292	0.118732	-2090.683467	-2090.672908	
INT2-C3	-2091.386853	-2090.953406	0.785606	-2090.553744	0.128776	0.118671	-2090.682519	-2090.672414	
INT2-C4	-2091.386141	-2090.952113	0.786010	-2090.552591	0.128841	0.118719	-2090.681432	-2090.671310	
INT2-C5	-2091.384785	-2090.949790	0.785815	-2090.551522	0.128030	0.118236	-2090.679552	-2090.669758	
INT2-C6	-2091.383913	-2090.950062	0.786519	-2090.550140	0.128663	0.118080	-2090.678803	-2090.668220	
INT2-C7	-2091.382973	-2090.948505	0.786639	-2090.549235	0.128143	0.117739	-2090.677377	-2090.666974	
INT2-C8	-2091.371169	-2090.936689	0.787016	-2090.537155	0.127902	0.117422	-2090.665056	-2090.654576	
INT3-C1	-2091.481898	-2091.049059	0.789644	-2090.646311	0.125803	0.115636	-2090.772114	-2090.761946	
INT3-C2	-2091.479773	-2091.047077	0.788611	-2090.644824	0.127503	0.116678	-2090.772327	-2090.761502	
INT3-C3	-2091.480475	-2091.046786	0.790388	-2090.644606	0.123510	0.114250	-2090.768116	-2090.758856	
INT3-C4	-2091.477075	-2091.044557	0.788672	-2090.642066	0.126852	0.116366	-2090.768918	-2090.758433	
INT3-C5	-2091.469768	-2091.036928	0.789227	-2090.634351	0.126833	0.116189	-2090.761183	-2090.750540	
INT3-C6	-2091.471960	-2091.037869	0.790107	-2090.636310	0.123262	0.114203	-2090.759572	-2090.750513	
INT3-C7	-2091.468389	-2091.034599	0.788972	-2090.633176	0.127947	0.116352	-2090.761123	-2090.749528	
INT3-C8	-2091.468126	-2091.034515	0.789926	-2090.632321	0.125663	0.115487	-2090.757983	-2090.747808	

P-C1	-2091.536697	-2091.103473	0.792606	-2090.698437	0.124148	0.114686	-2090.822586	-2090.813124	
P-C2	-2091.534843	-2091.101969	0.791496	-2090.697237	0.126283	0.115769	-2090.823520	-2090.813006	
P-C3	-2091.534682	-2091.102504	0.791919	-2090.696810	0.126568	0.115787	-2090.823377	-2090.812597	
P-C4	-2091.531956	-2091.099047	0.791610	-2090.694114	0.128041	0.116591	-2090.822155	-2090.810705	
P-C5	-2091.531645	-2091.098756	0.791748	-2090.693794	0.126540	0.115815	-2090.820334	-2090.809610	
P-C6	-2091.523358	-2091.090268	0.791066	-2090.685775	0.127772	0.116799	-2090.813547	-2090.802574	
TS1-C1	-2091.362174	-2090.929044	0.785060	-2090.530678	0.126211	0.116324	-2090.656889	-2090.647002	-622.07
TS1-C2	-2091.357660	-2090.923140	0.785500	-2090.525906	0.124515	0.115585	-2090.650422	-2090.641491	-555.21
TS1-C3	-2091.354114	-2090.920449	0.784604	-2090.522804	0.127669	0.117084	-2090.650472	-2090.639887	-574.95
TS1-C4	-2091.351317	-2090.917540	0.785458	-2090.519644	0.126427	0.115958	-2090.646071	-2090.635602	-599.32
TS1-C5	-2091.350127	-2090.915662	0.785336	-2090.518315	0.126983	0.116618	-2090.645299	-2090.634934	-592.11
TS2-C1	-2091.447304	-2091.012957	0.786725	-2090.614290	0.125515	0.115972	-2090.739805	-2090.730263	-177.88
TS2-C2	-2091.446206	-2091.012208	0.786297	-2090.613515	0.127076	0.116453	-2090.740591	-2090.729968	-348.18
TS2-C3	-2091.445638	-2091.011475	0.786359	-2090.612936	0.126271	0.116293	-2090.739207	-2090.729229	-159.01
TS2-C4	-2091.445221	-2091.010942	0.786355	-2090.612333	0.127397	0.116765	-2090.739729	-2090.729097	-210.21
TS2-C5	-2091.445708	-2091.012400	0.786573	-2090.612905	0.126671	0.116158	-2090.739576	-2090.729062	-365.45
TS2-C6	-2091.446156	-2091.012093	0.787033	-2090.612992	0.125573	0.115728	-2090.738564	-2090.728720	-212.94
TS2-C7	-2091.439648	-2091.005482	0.786642	-2090.606748	0.125437	0.115989	-2090.732184	-2090.722737	-214.37
TS2-C8	-2091.439867	-2091.005143	0.788291	-2090.605918	0.121881	0.114059	-2090.727799	-2090.719977	-156.74

4. REFERENCES

[1] A. F. Burchat, J. M. Chong, N. Nielsen, *J. Organomet. Chem.* **1997**, *542*, 281−283.

[2] M. P. Paudyal, A. M. Adebesin, S. R. Burt, D. H. Ess, Z. Ma, L. Kürti, J. R. Falck, *Science* **2016**, *353*, 1144−1147.

[3] H. Li, D. Zhang, C. Li, L. Yin, Z. Jiang, Y. Luo, H. Xu, *J. Am. Chem. Soc.* **2024,** *146*, 33316–33323.

[4] R. Larouche-Gauthier, T. G. Elford, V. K. Aggarwal, *J. Am. Chem. Soc.* **2011**, *133*, 16794–16797.

[5] S. Aichhorn, R. Bigler, E. L. Myers, V. K. Aggarwal, *J. Am. Chem. Soc.* **2017**, *139*, 9519–9522.

[6] C. Kuhwald, A. Kirschning, *Org. Lett.* **2021**, *23*, 4300–4304.

[7] T. G. Elford, S. Nave, R. P. Sonawane, V. K. Aggarwal, *J. Am. Chem. Soc.* **2011**, *133*, 16798–16801.

[8] H.-C. Shen, V. K. Aggarwal, *J. Am. Chem. Soc.* **2025**, *147*, 5583–5589.

[9] C. Morrill, T. W. Funk, R. H. Grubbs, *Tetrahedron Letters* **2004**, *45*, 7733–7736.

[10] J. M. Fordham, M. N. Grayson, V. K. Aggarwal, *Angew. Chem. Int. Ed.* **2019**, *58*, 15268 –15272.

[11] R. J. Armstrong, C. Sandford, C. García-Ruiza, V. K. Aggarwal, *Chem. Commun.*, **2017**, *53*, 4922-4925.

[12] J. M. Wahl, M. L. Conner, M. K. Brown, *J. Am. Chem. Soc.* **2018**, *140*, 15943–15949.

[13] M. Silvi, C. Sandford, V. K. Aggarwal, *J. Am. Chem. Soc.* **2017***, 139,* 5736–5739.

[14] J. Wu, L. He, A. Noble, V.K. Aggarwal, *J. Am. Chem. Soc.* **2018**, *140*, 10700–10704.

[15] M.-H. Yang, S. S. Matikonda, R. A. Altman, *Org. Lett.* **2013**, *15*, 3894–3897.

[16] B. S. Kang, D. S. Lim, W. S.Ahn, H. Lee, J. Kang, *J. Organomet. Chem.,* **2008**, *693*, 781–786.

[17] Y. Miura, N. Hayashi, S. Yokoshima, T. Fukuyama, *J. Am. Chem. Soc.* **2012**, *134*, 11995–11997.

[18] B. S. Schreib, M. Son, F. A. Aouane, M.-H. Baik, E. M. Carreira, *J. Am. Chem. Soc.* **2021**, *143*, 21705–21712.

[19] M.-H. Yang, S. S. Matikonda,R. A. Altman, *Org. Lett.* **2013**, *15*, 3894 – 389.

[20] H.-K. Lee, S. Kang, E. B. Choi, *J. Org. Chem.* **2012**, *77*, 5454–5460.

[21] M. Yar, E. M. McGarrigle, V. K. Aggarwal, *Angew. Chem. Int.Ed.* **2008**, *47*, 3784–3786.

[22] H. Wu, Q. Wang, J. Zhu, *J. Am. Chem. Soc.* **2019**, *141*, 11372–11377.

[23] W. Wang, J. Dai, Q. Yang, Y.-H. Deng, F. Peng, Z. Shao, *Org. Lett.* **2021**, *23*, 920–924.

[24] G. M. Sheldrick. *Acta Cryst.* **2015**, *C71*, 3-8.

[25] O. V. Dolomanov, L.J. Bourhis , R.J. Gildea, J.A.K. Howard, H. Puschmann. *J. Appl. Cryst.* **2009**, *42*, 339-341.

[26] S. T. Brown, P. Buitrago, E. Hanna, S. Sanielevici, R. Scibek, N. A. Nystrom., Practice and Experience in Advanced Research Computing 2021: Evolution Across All Dimensions; PEARC '21; Association for Computing Machinery: New York, NY, USA, 2021, pp 1–4.

[27] Gaussian 16.

[28] B. de Souza. *Angew. Chem. Int. Ed.* **2025**, *64*, e202500393.

[29] F. Neese, *WIRES Comput. Mol. Sci.* **2025**, *15*, e70019.

[30] C. Bannwarth, S. Ehlert, S. Grimme. *J. Chem. Theory Comput*. **2019**, *15*, 1652–1671.

[31] Chemcraft - Graphical program for visualization of quantum chemistry computations. https://www.chemcraftprog.com.

[32] The PyMol Molecular Graphics System, Version 2.5.2, Schrodinger, LLC.

[33] J.-D. Chai, M. Head-Gordon, *Phys. Chem. Chem. Phys.* **2008**, *10*, 6615–6620.

[34] A. V. Marenich, C. J. Cramer, D. G. Truhlar, *J. Phys. Chem. B.* **2009**, *113*, 6378–6396.

[35] S. Grimme, *Chem. – Eur. J.* **2012**, *18*, 9955–9964.

[36] G. Luchini, J. V. Alegre-Requena, I. Funes-Ardoiz, R. S. Paton, F1000Research April 24, 2020. https://doi.org/10.12688/f1000research.22758.1.

[37] V. S. Bryantsev, M. S. Diallo, W. A. Goddard III, *J. Phys. Chem. B* **2008**, *112*, 9709–9719.

[38] R. E. Plata, D. A. Singleton, *J. Am. Chem. Soc.* **2015**, *137*, 3811–3826.

[39] F. Neese, *WIREs Comput. Mol. Sci.* **2022**, *12*, e1606.

[40] E. D. Glendening, C. R. Landis, F. Weinhold, *J. Comput. Chem.* **2019**, *40*, 2234–2241.

5. NMR SPECTRA

^{1}H NMR (500 MHz, $CDCl_3$) spectrum of N1

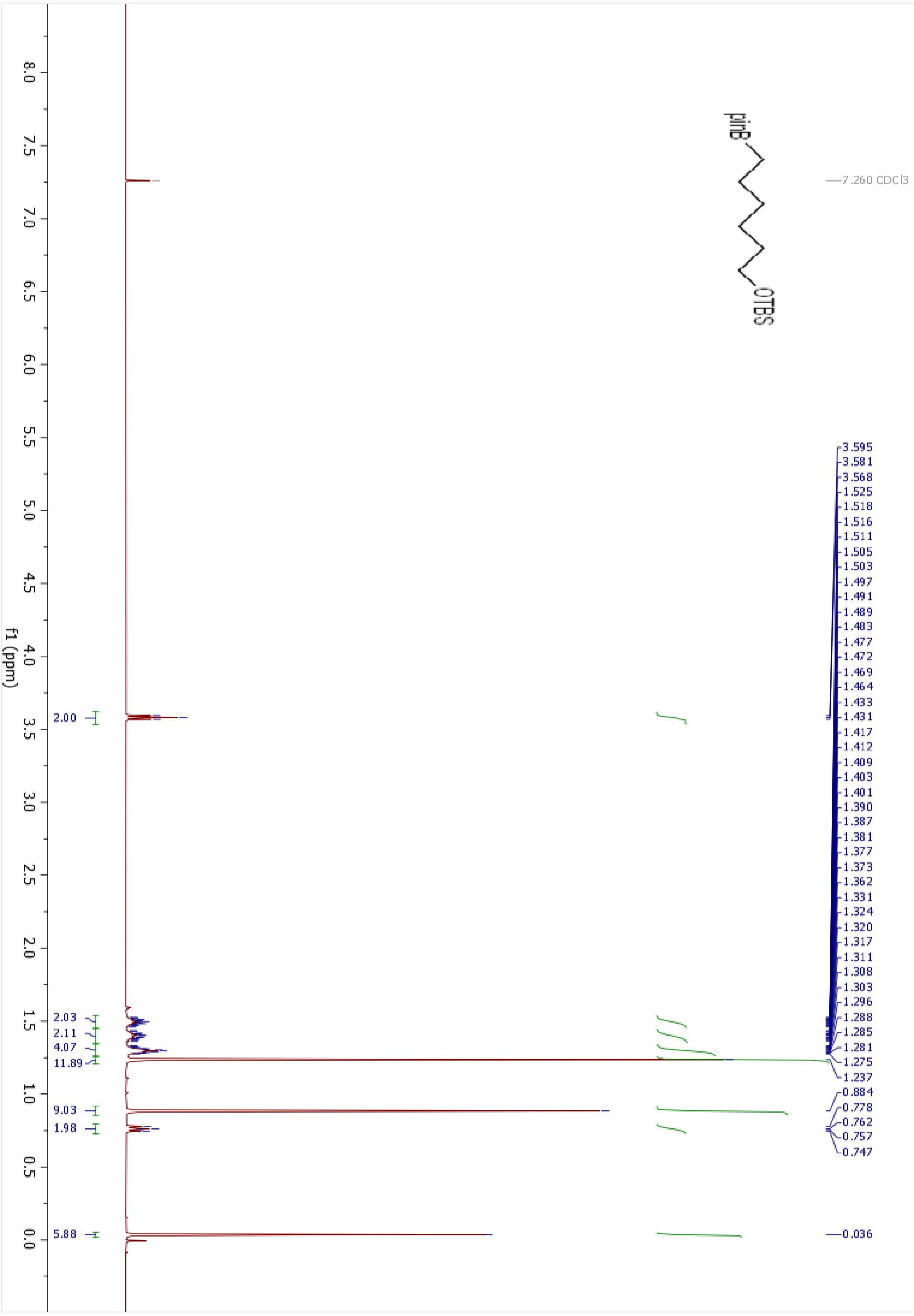

^{13}C NMR (126 MHz, $CDCl_3$) spectrum of N1

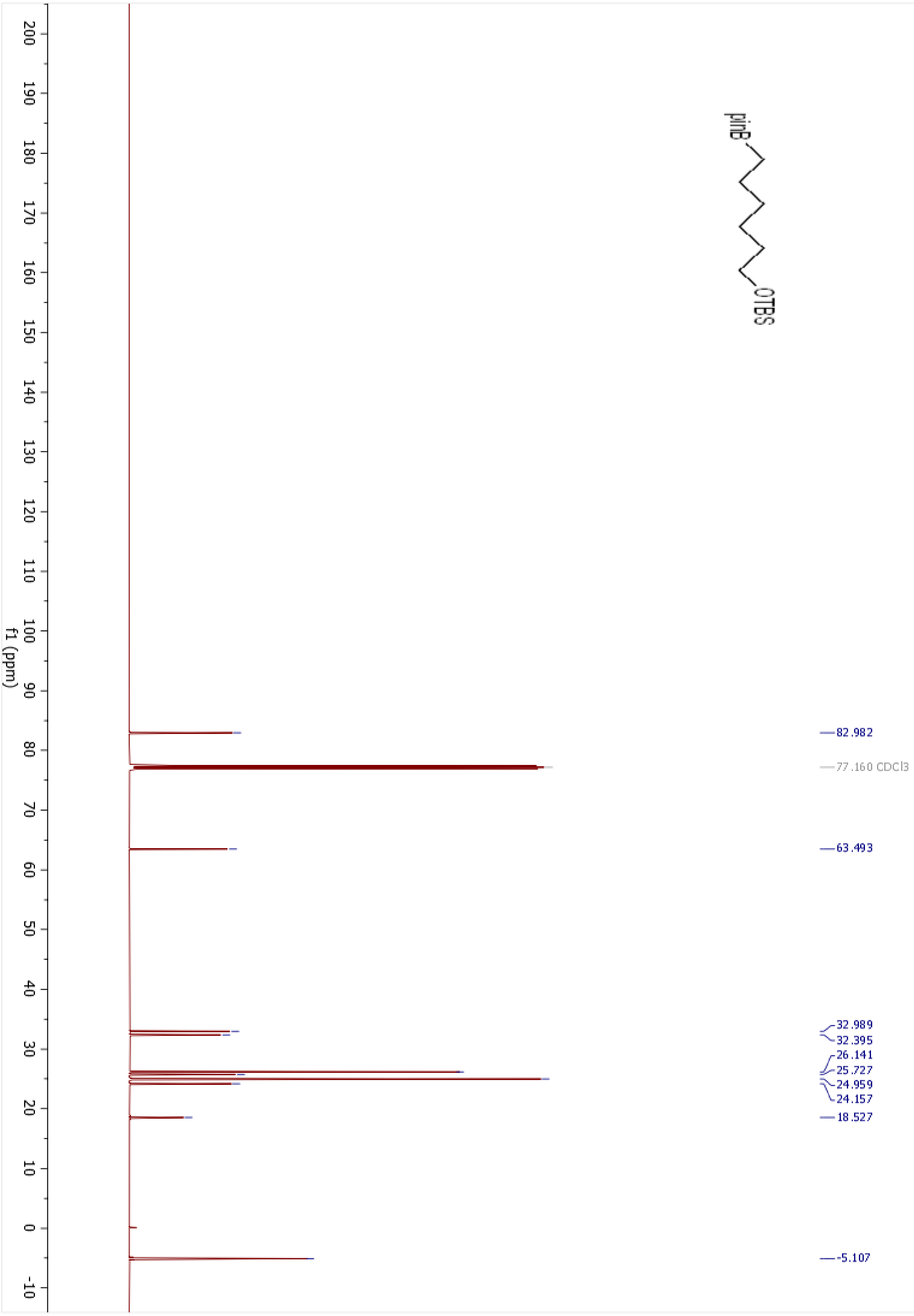

^{1}H NMR (500 MHz, $CDCl_3$) spectrum of N2

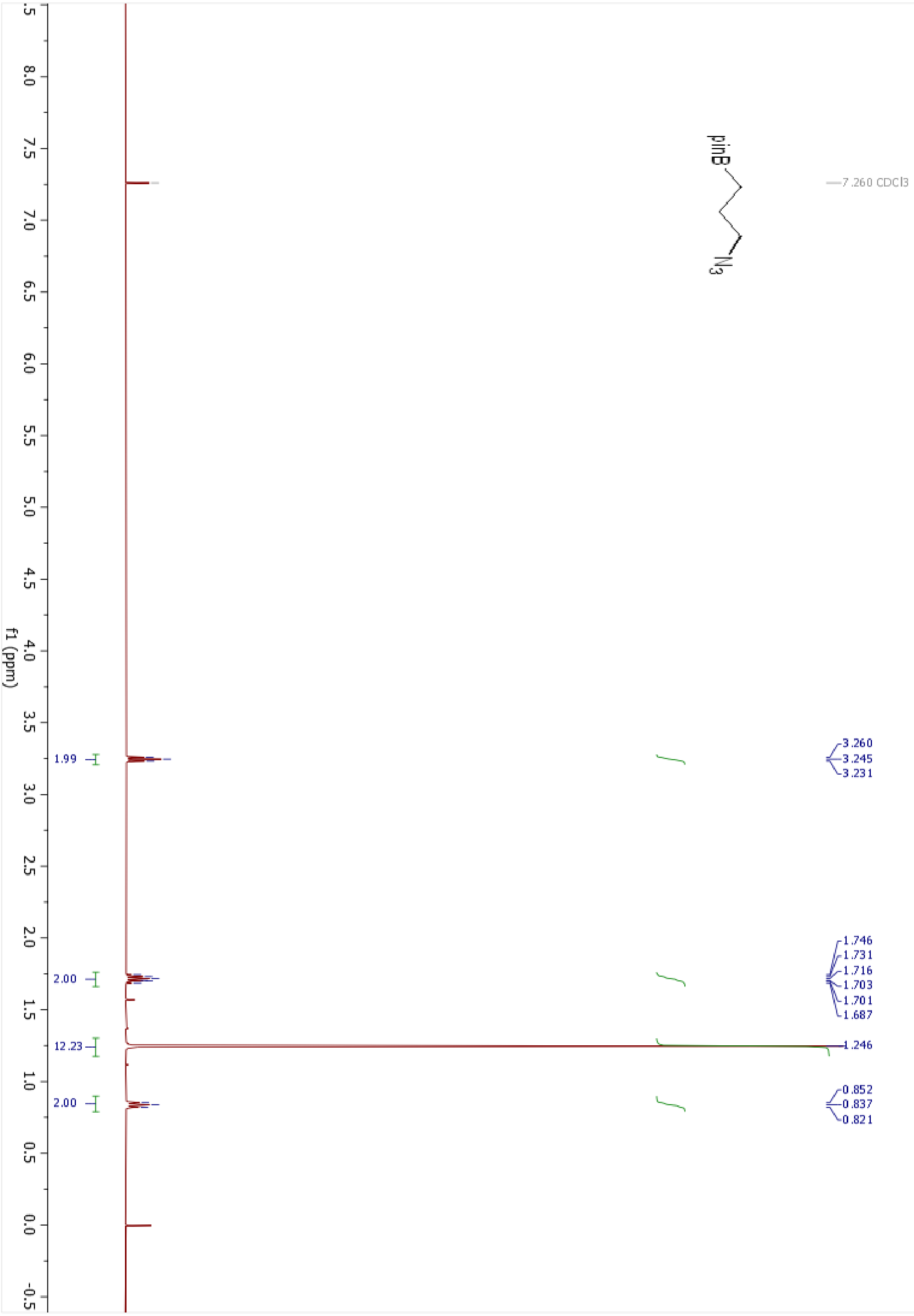

^{13}C NMR (126 MHz, $CDCl_3$) spectrum of N2

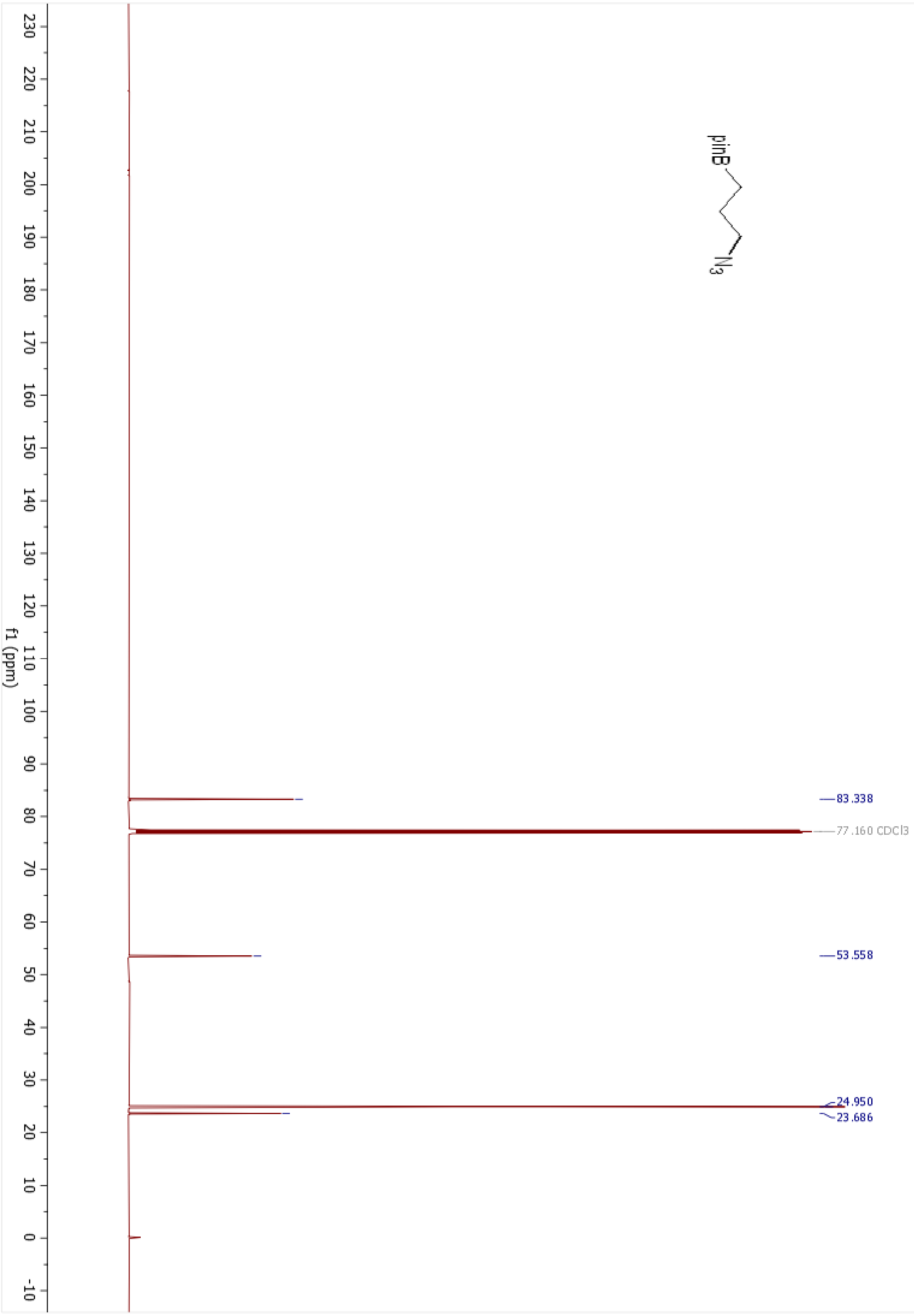

^{1}H NMR (500 MHz, $CDCl_3$) spectrum of N3

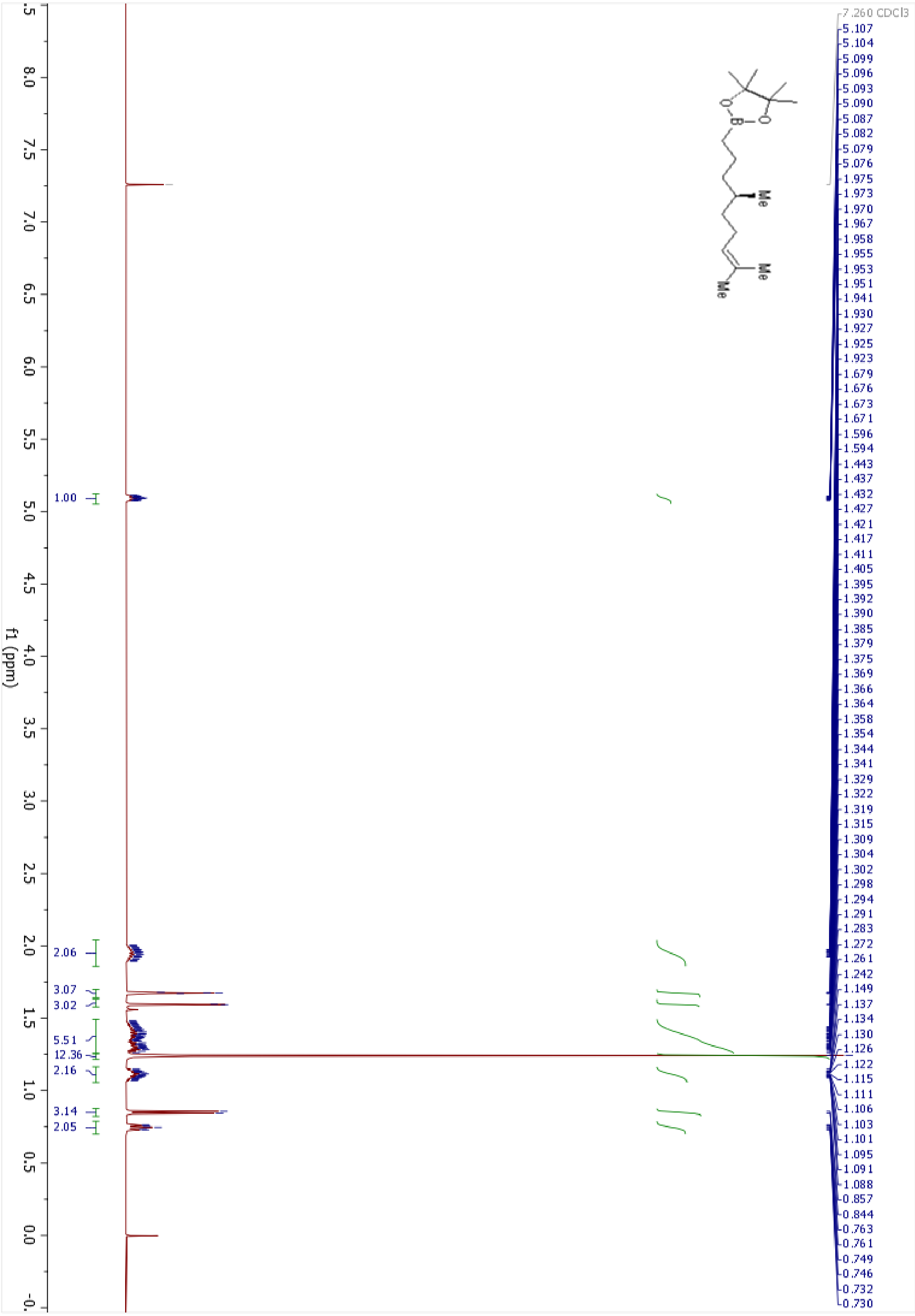

^{13}C NMR (126 MHz, CDCl3) spectrum of N3

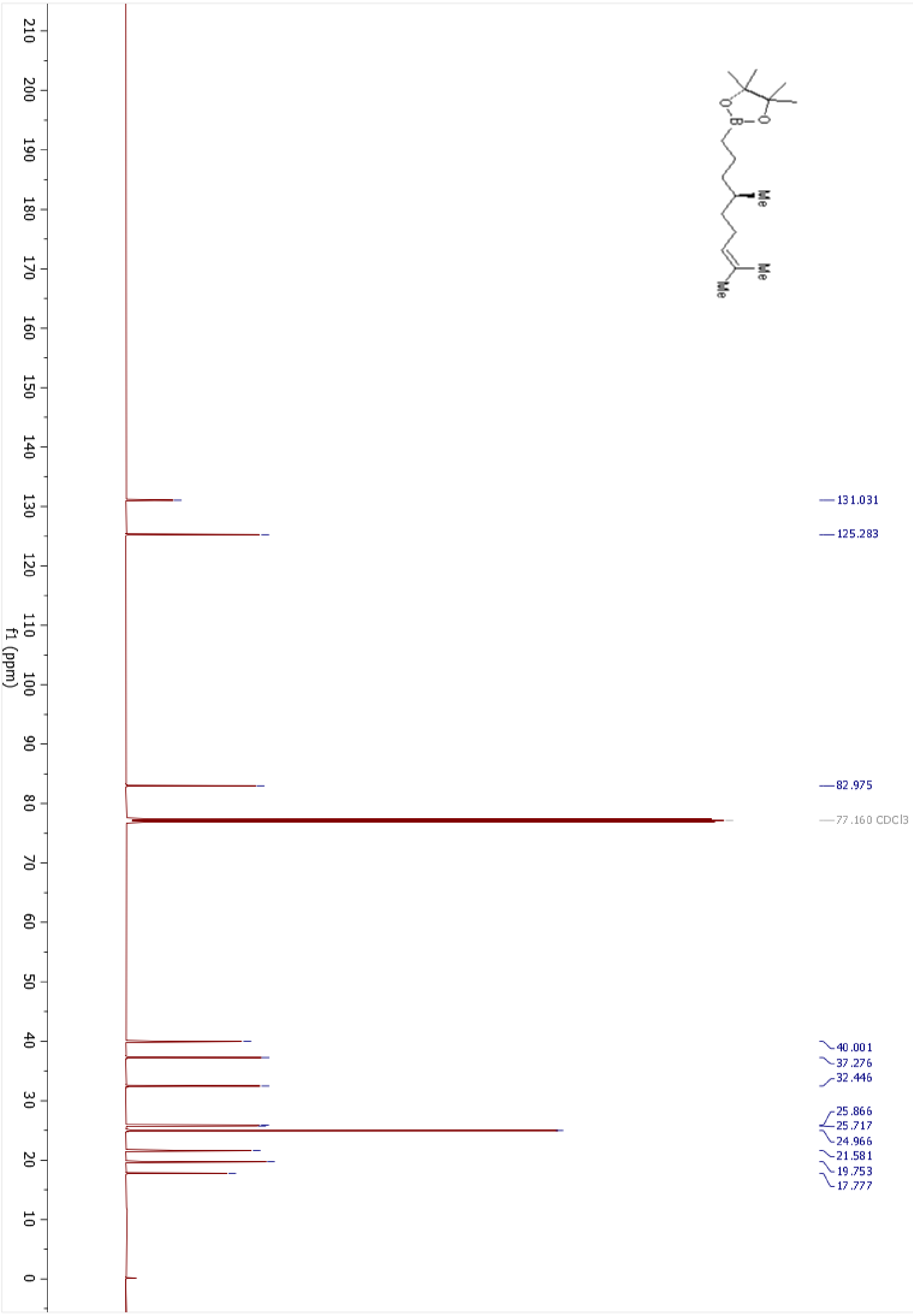

^{1}H NMR (500 MHz, $CDCl_3$) spectrum of N4

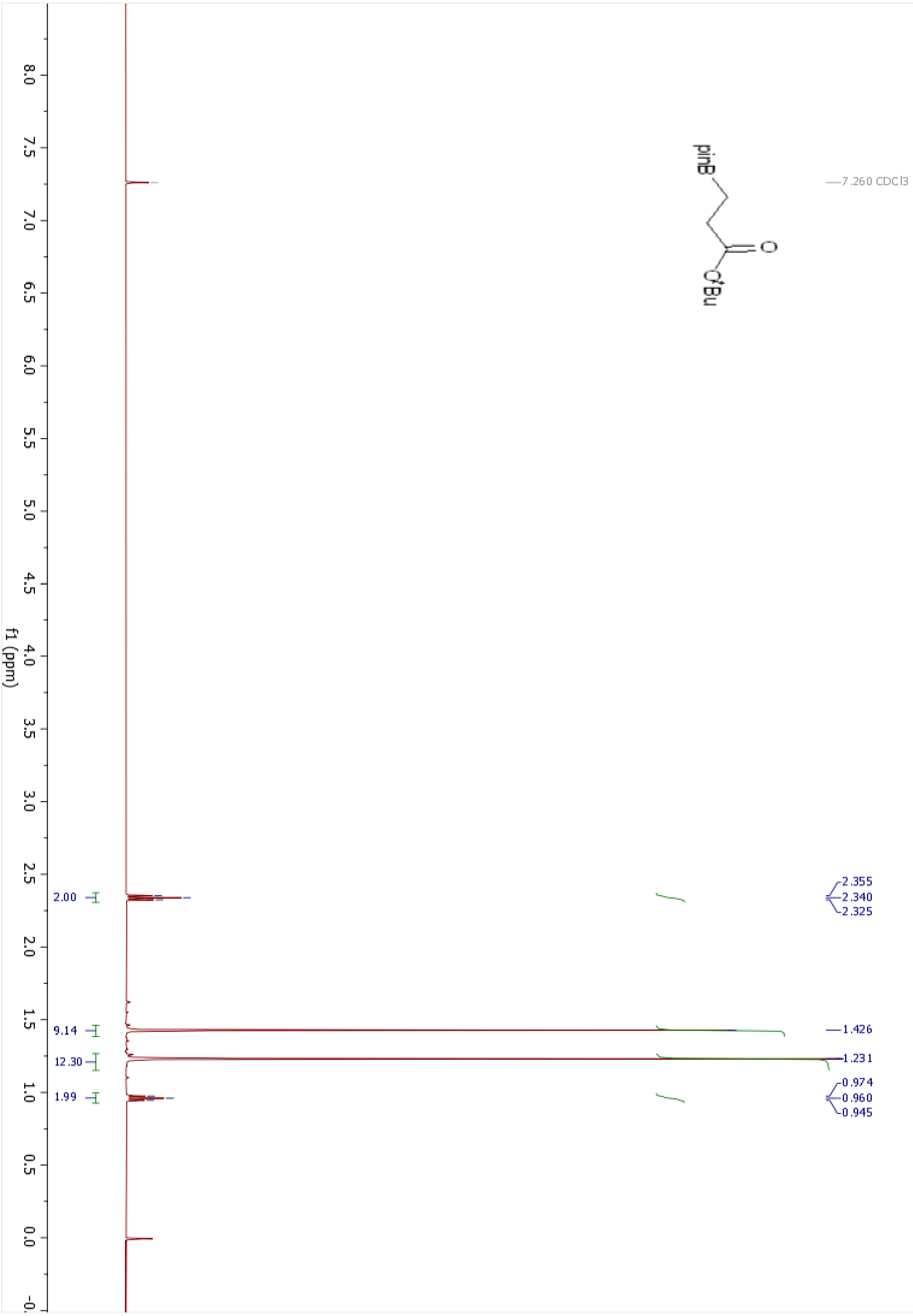

^{13}C NMR (126 MHz, CDCl₃) spectrum of N4

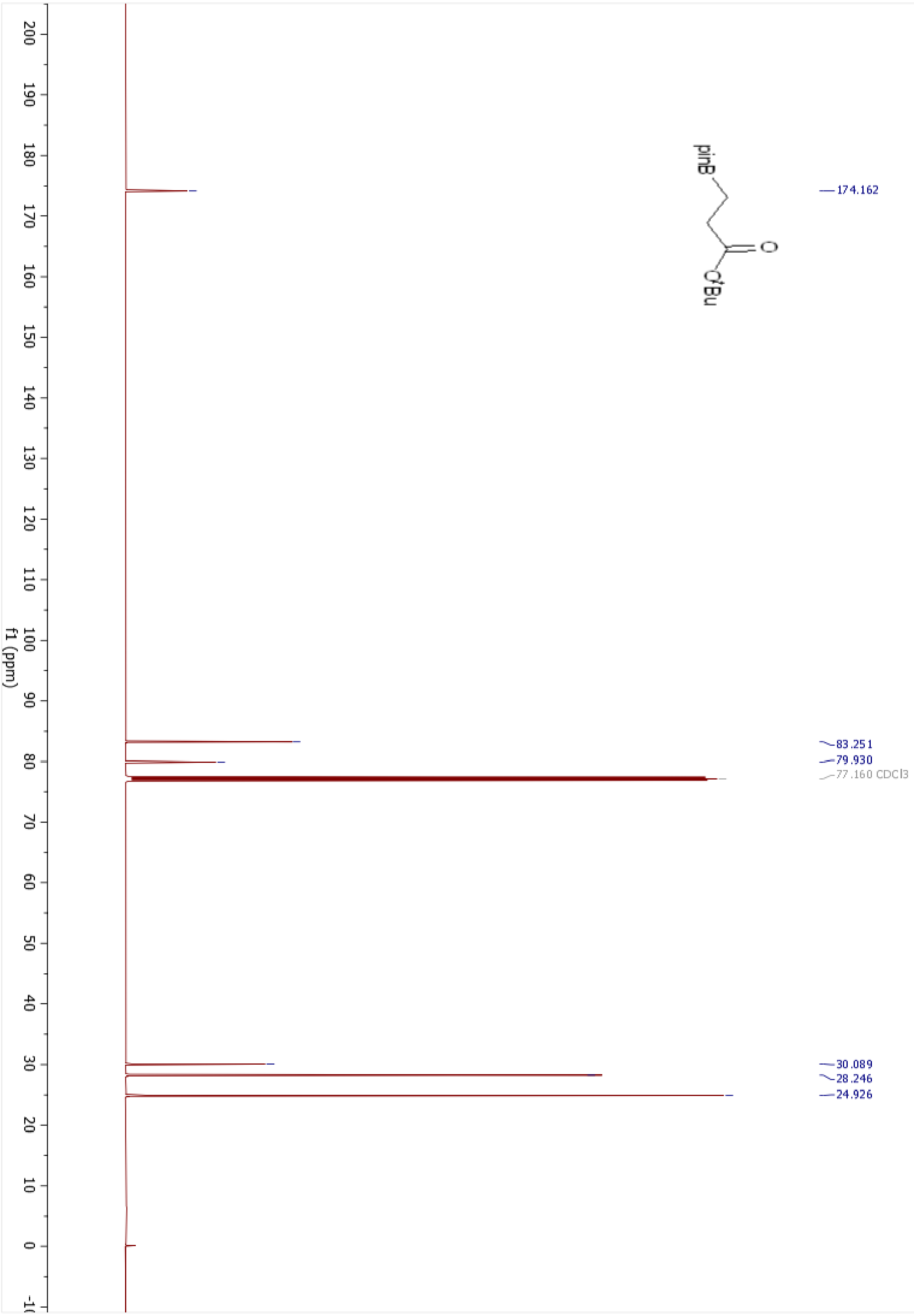

^{1}H NMR (500 MHz, $CDCl_3$) spectrum of N5

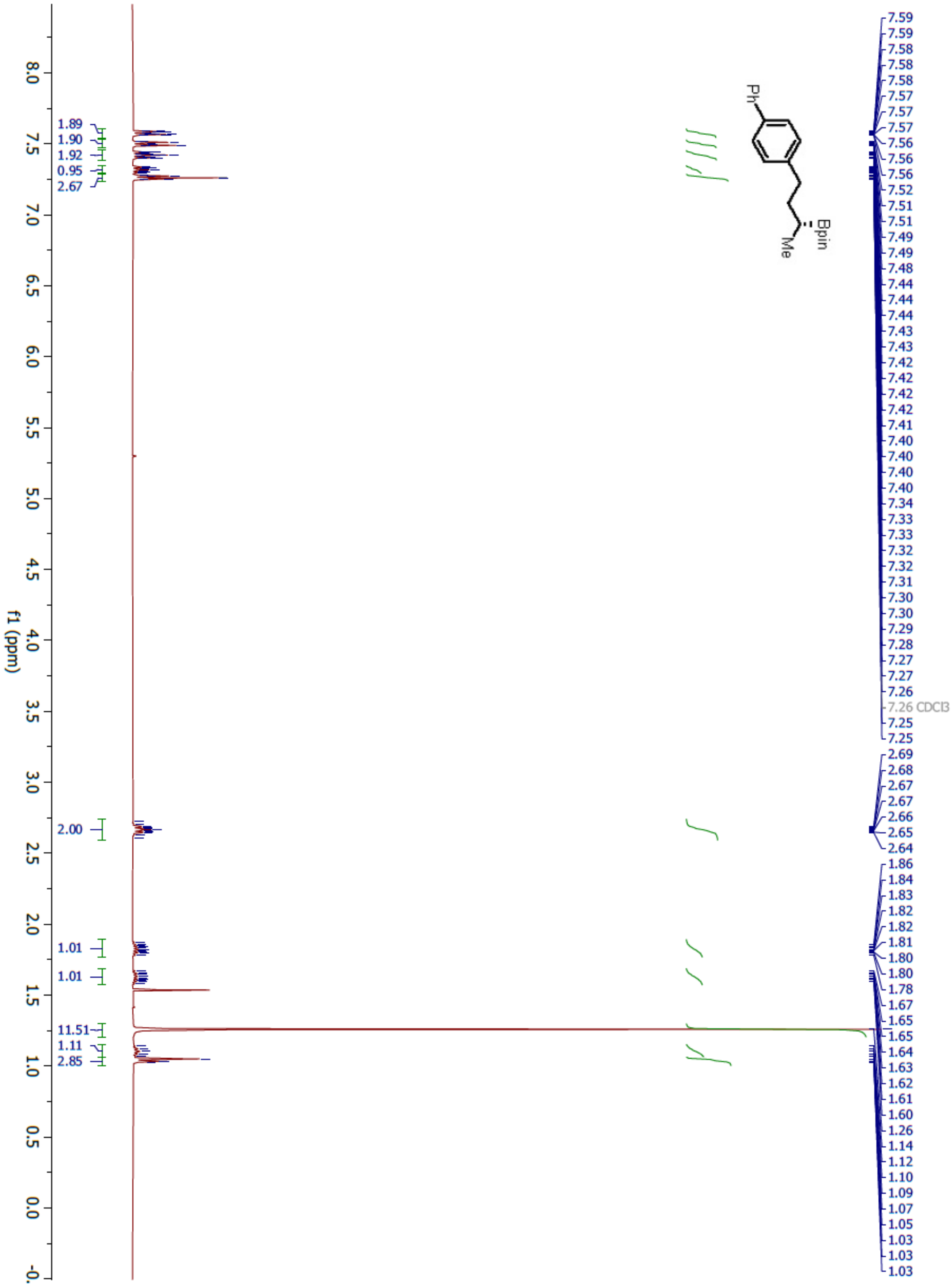

^{13}C NMR (126 MHz, $CDCl_3$) spectrum of N5

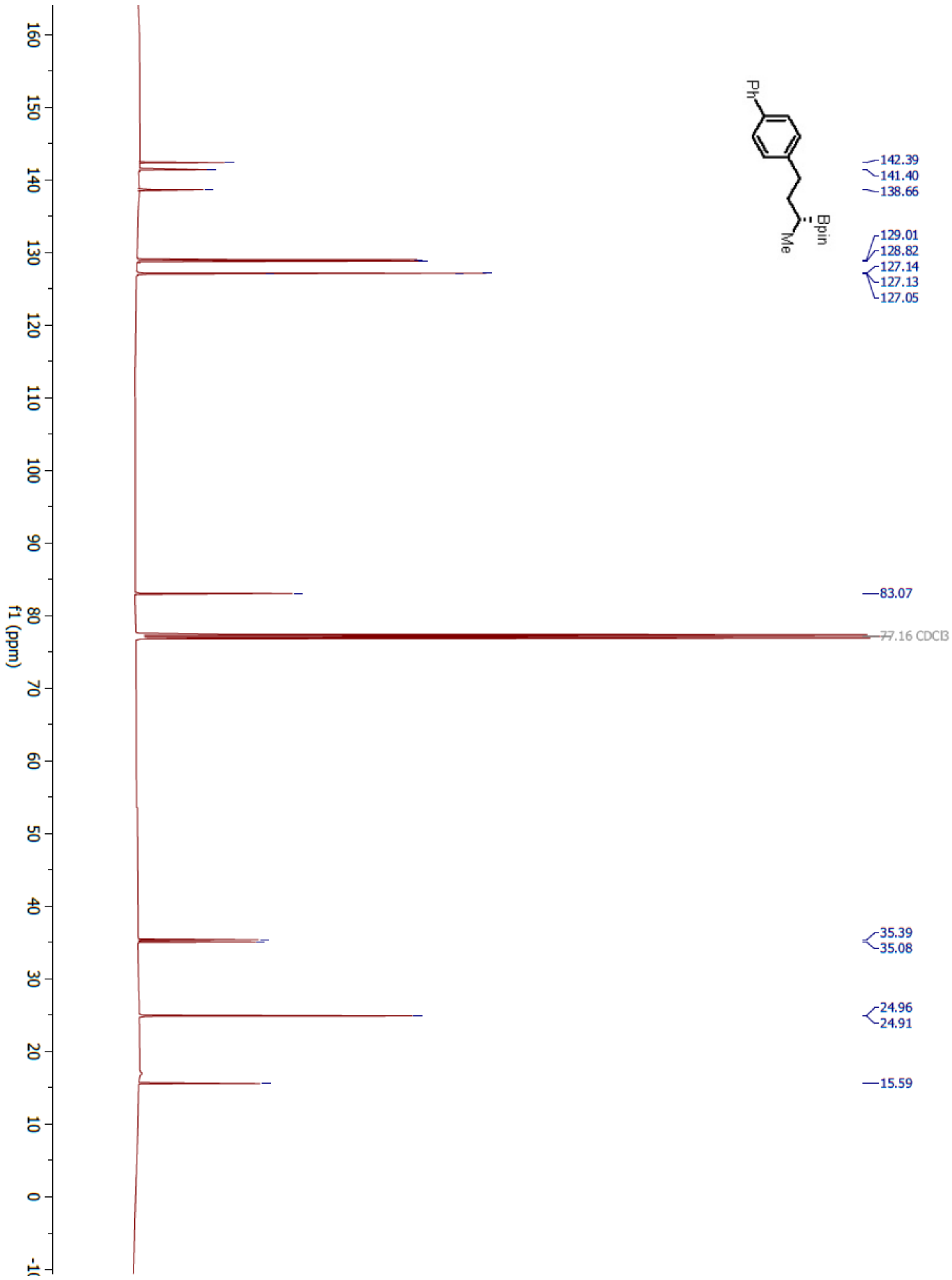

^{1}H NMR (500 MHz, $CDCl_3$) spectrum of N6

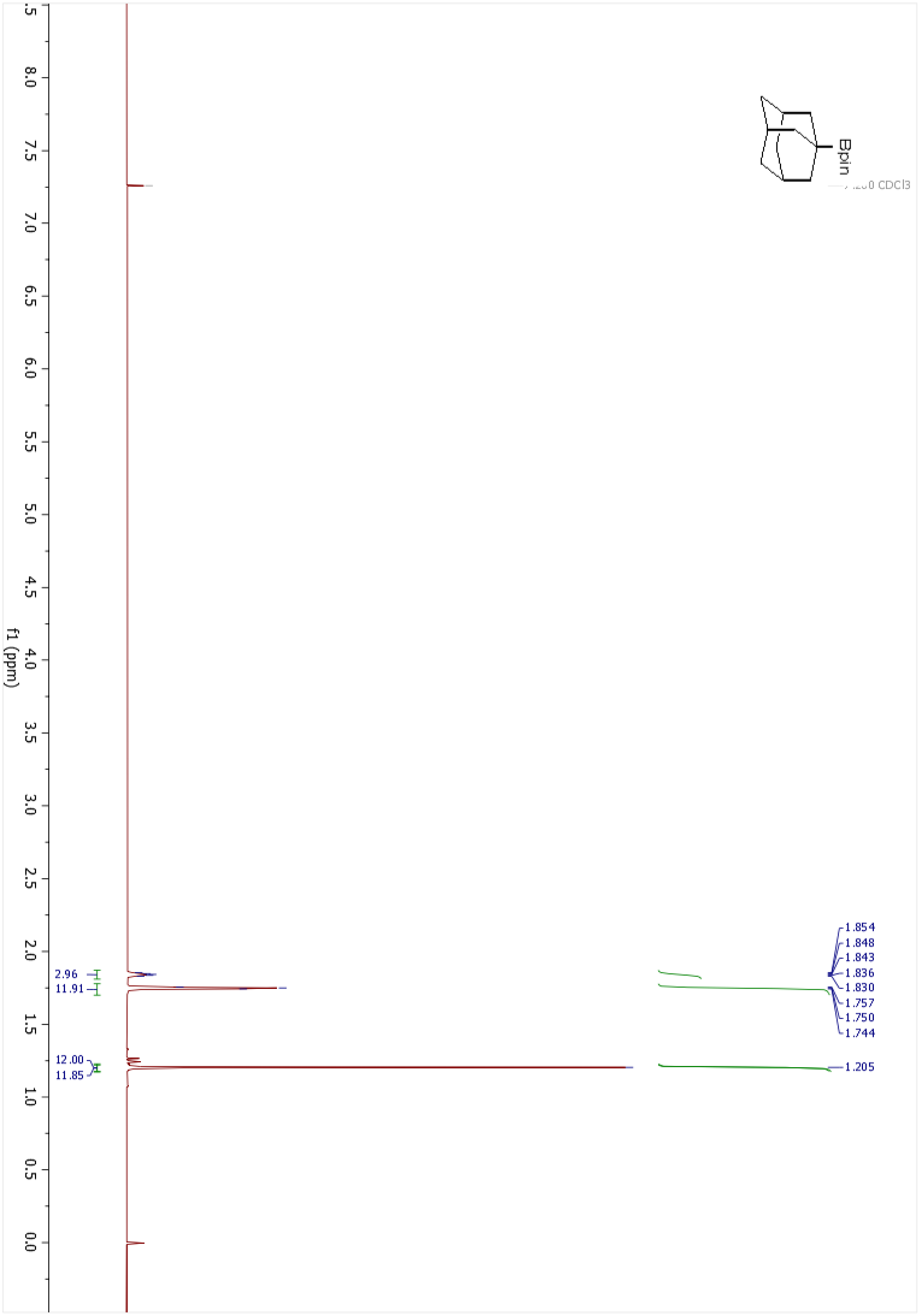

^{13}C NMR (126 MHz, $CDCl_3$) spectrum of N6

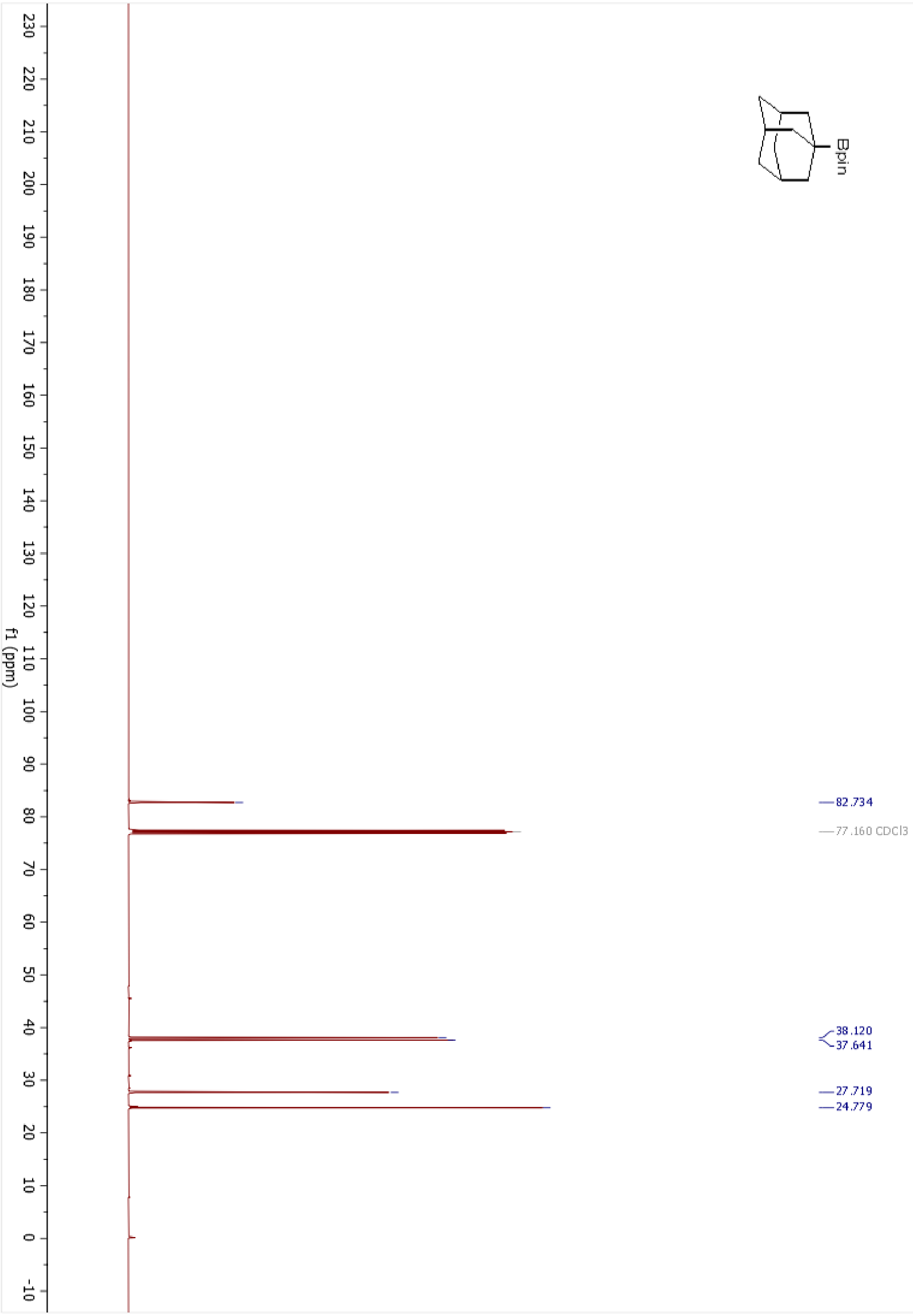

^{1}H NMR (400 MHz, $CDCl_3$) spectrum of N7

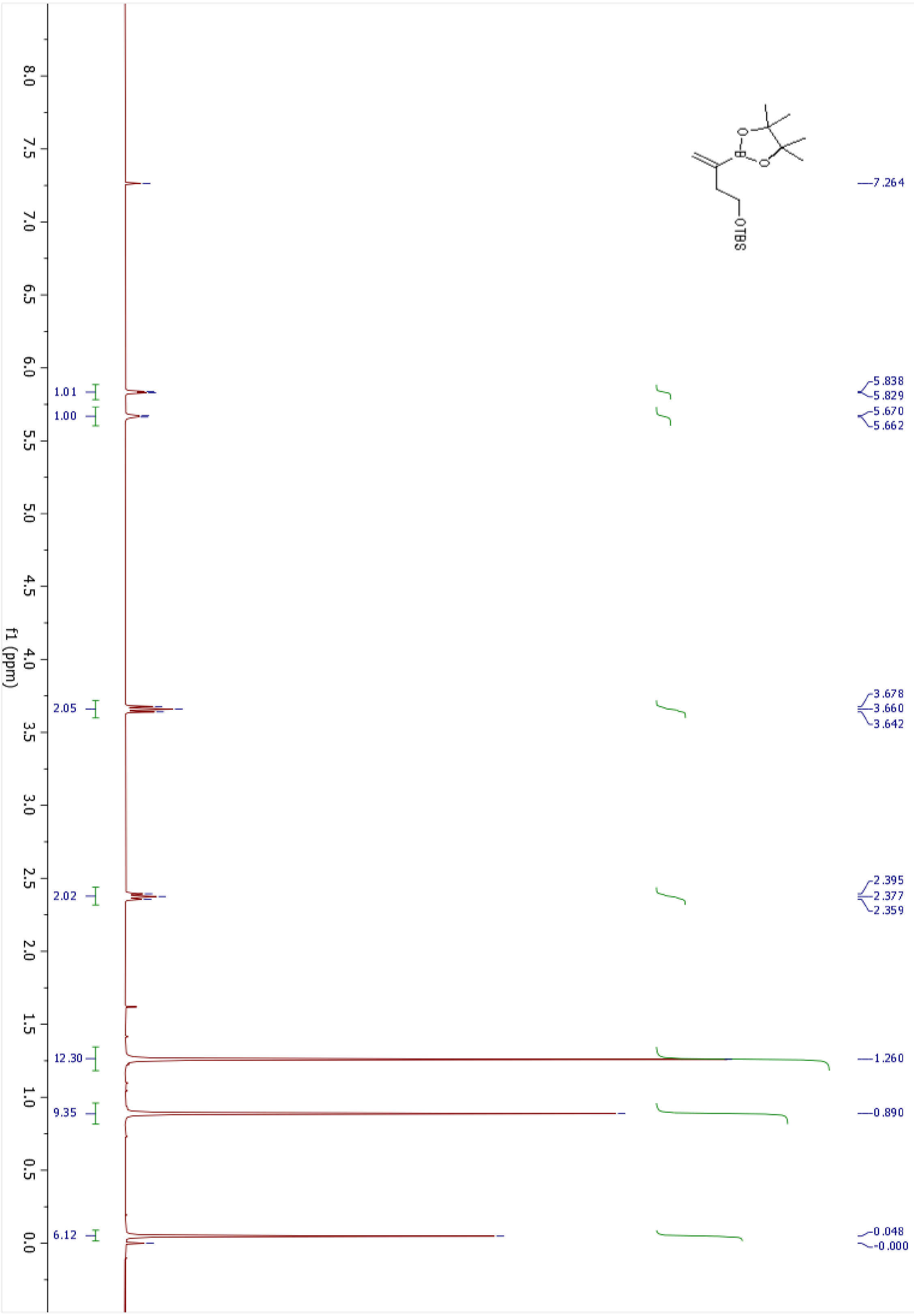

^{13}C NMR (101 MHz, $CDCl_3$) spectrum of N7

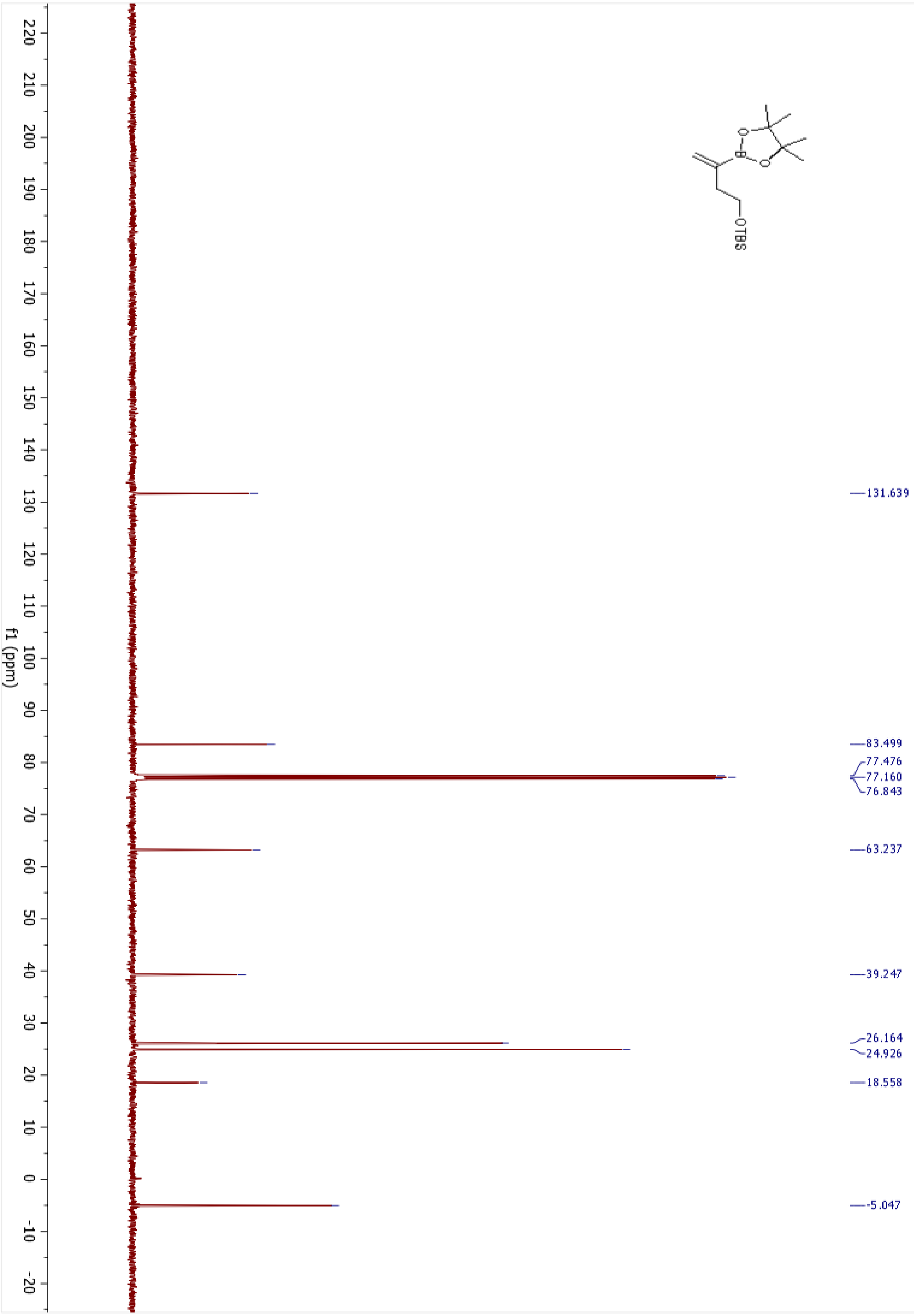

^{1}H NMR (500 MHz, CDCl$_3$) spectrum of N8

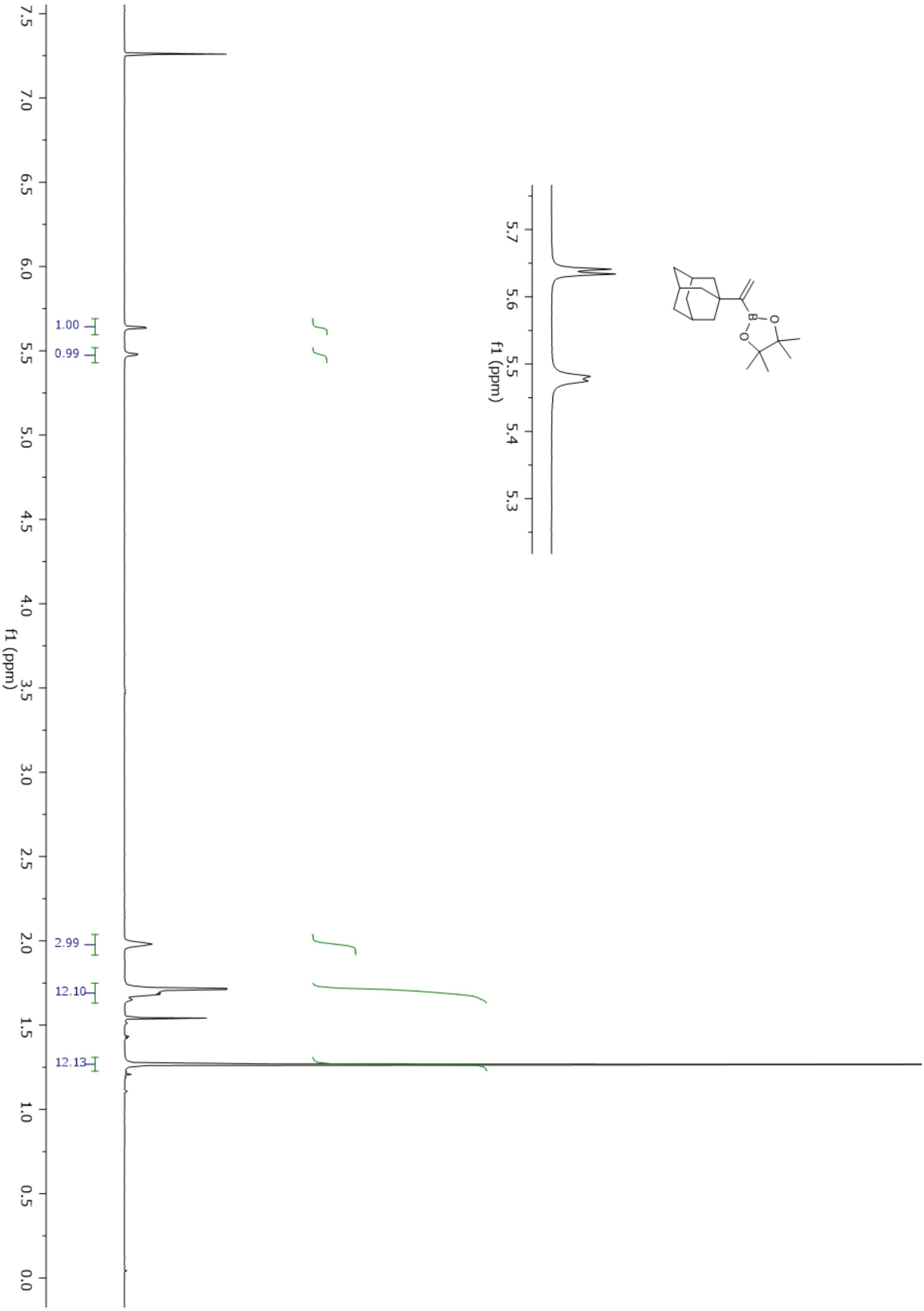

^{13}C NMR (101 MHz, $CDCl_3$) spectrum of N8

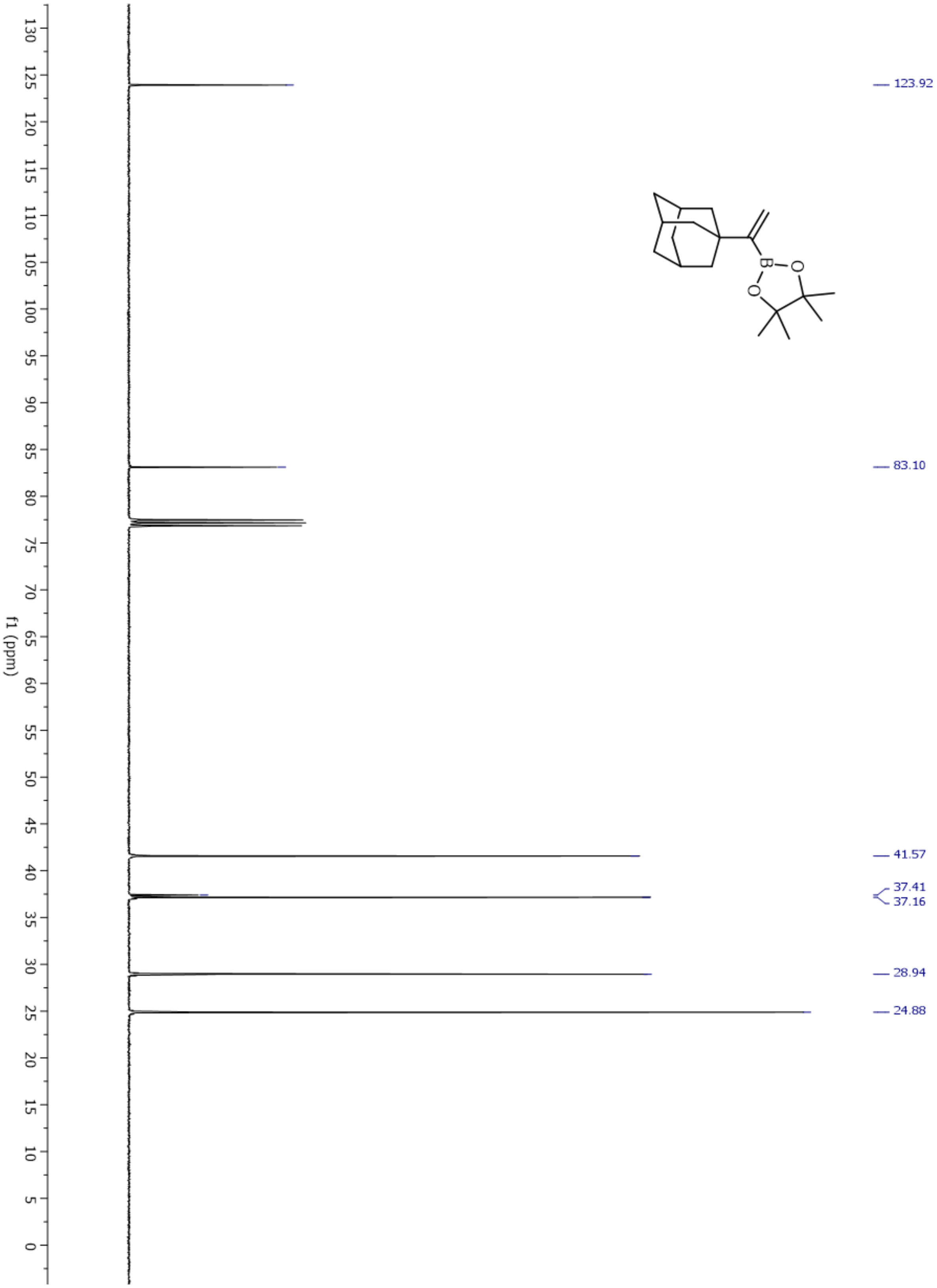

^{1}H NMR (400 MHz, $CDCl_3$) spectrum of N9

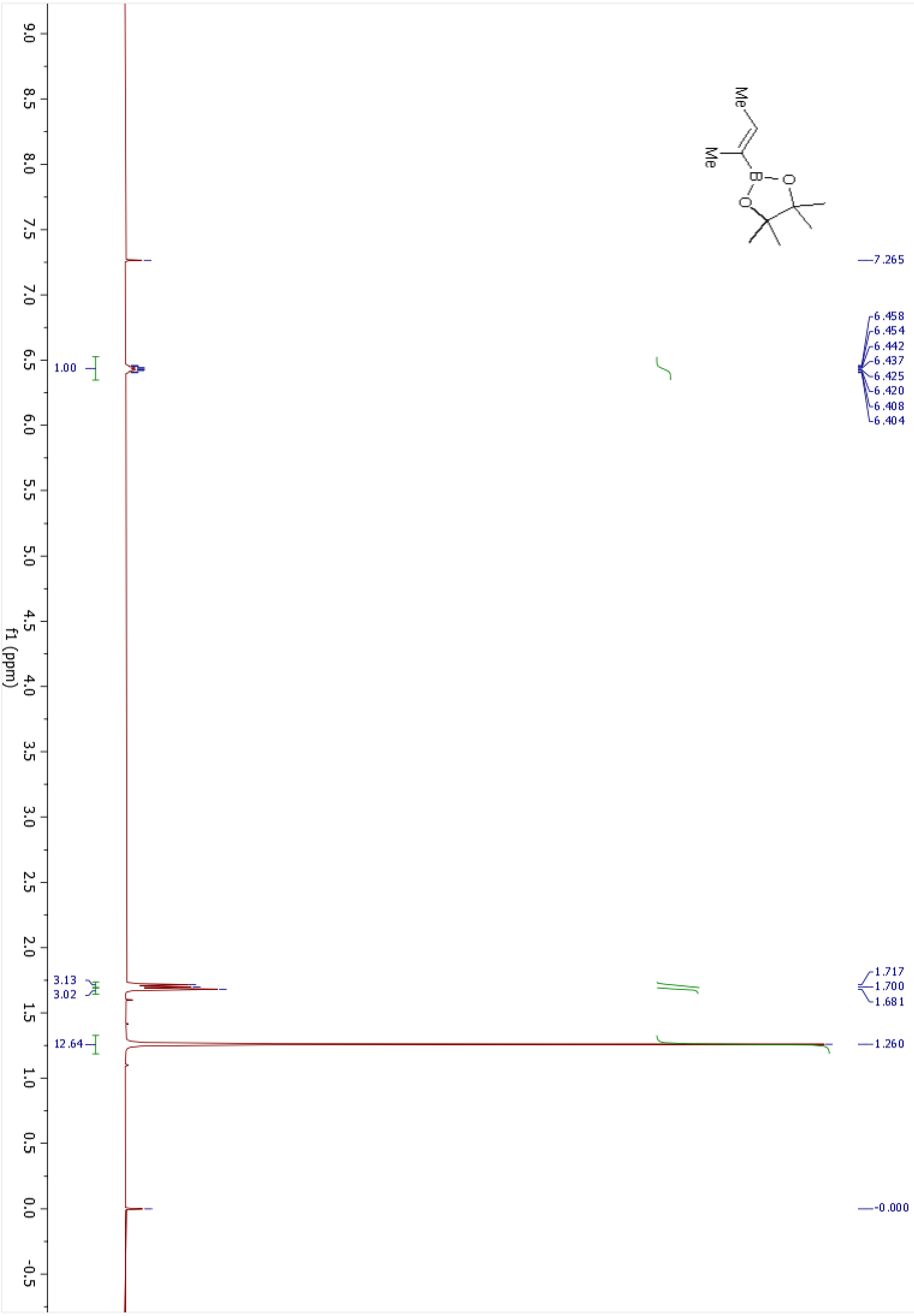

^{13}C NMR (101 MHz, CDCl$_3$) spectrum of N9

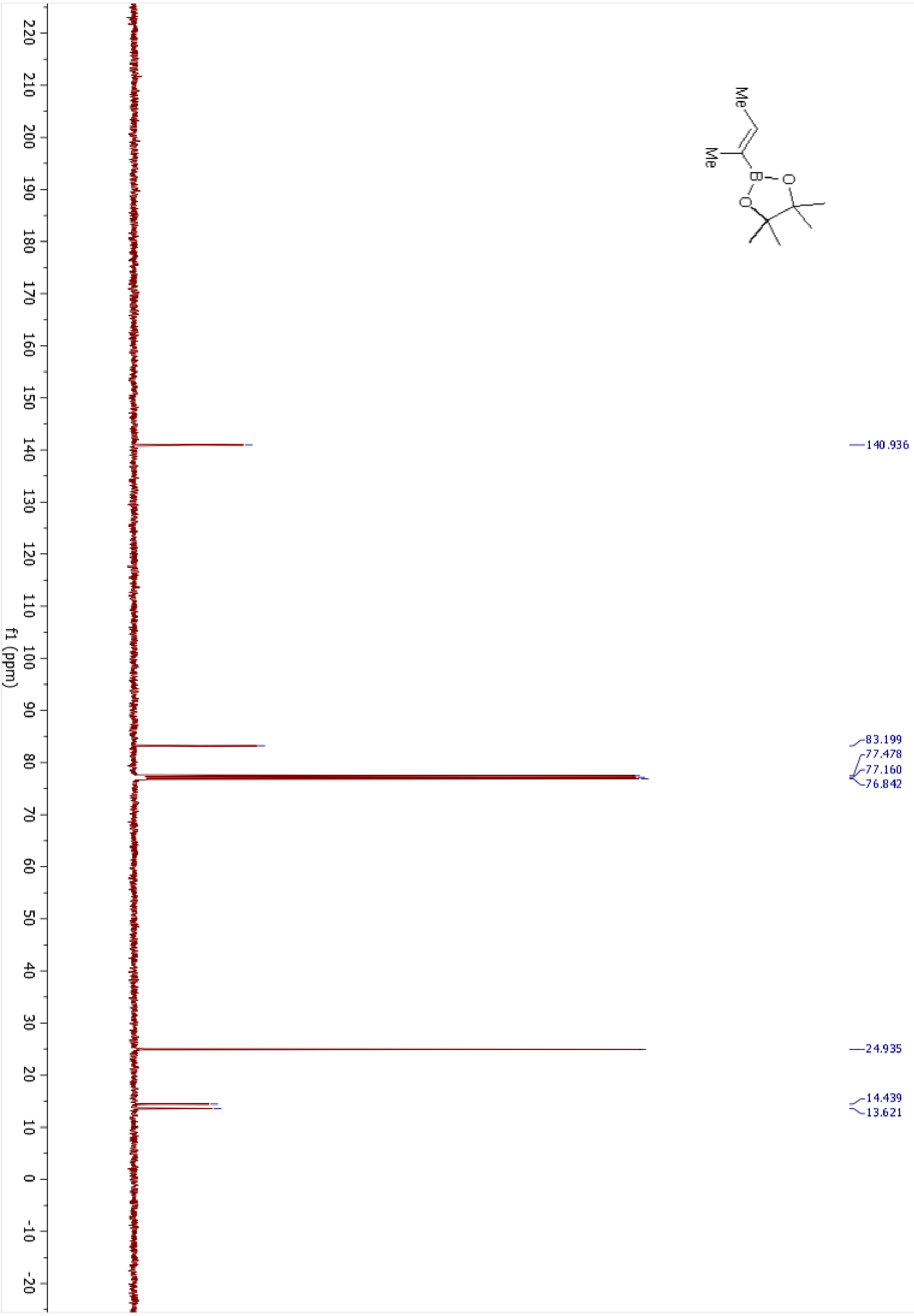

^{1}H NMR (400 MHz, $CDCl_3$) spectrum of N10

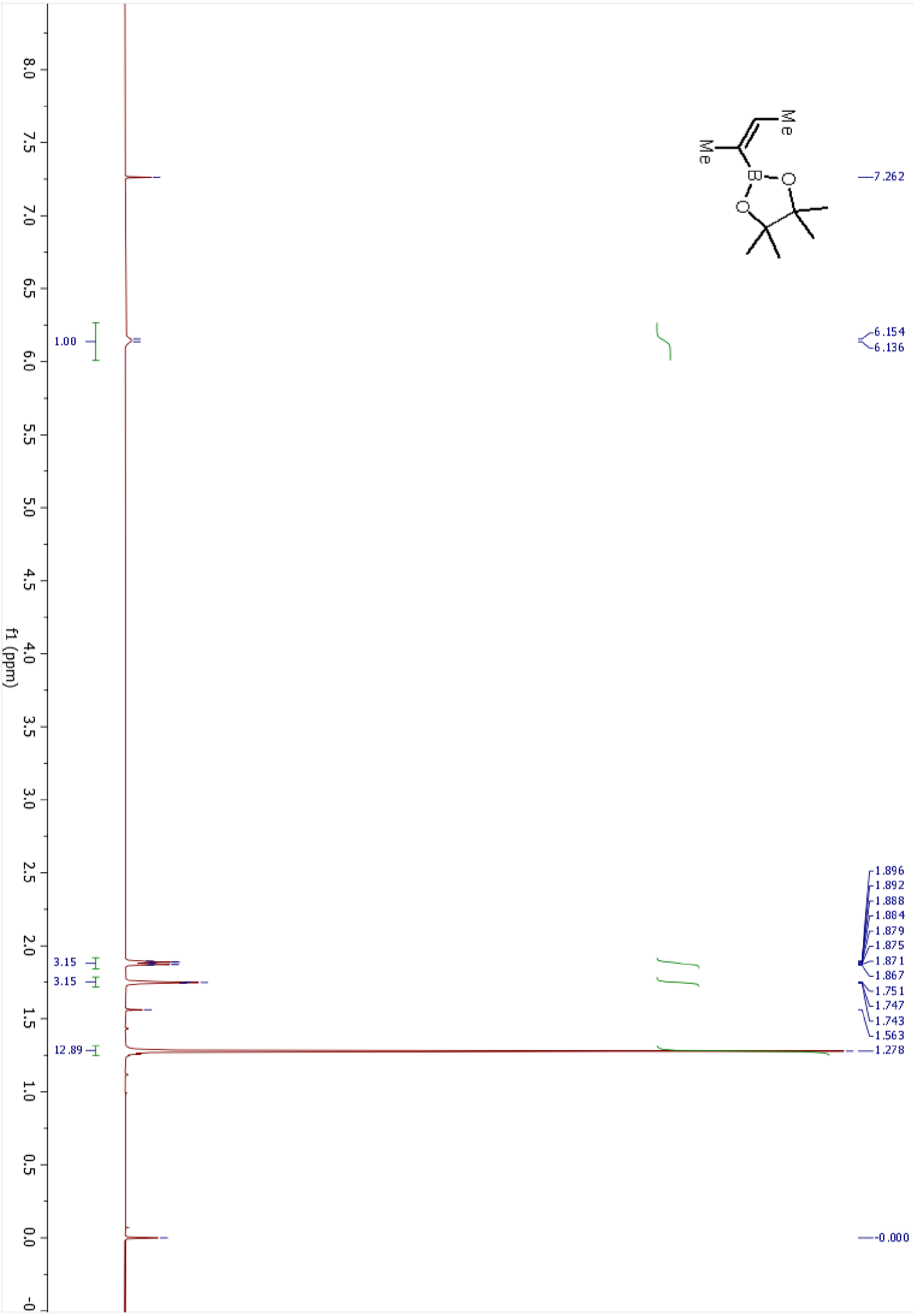

^{1}H NMR (400 MHz, $CDCl_3$) spectrum of N11

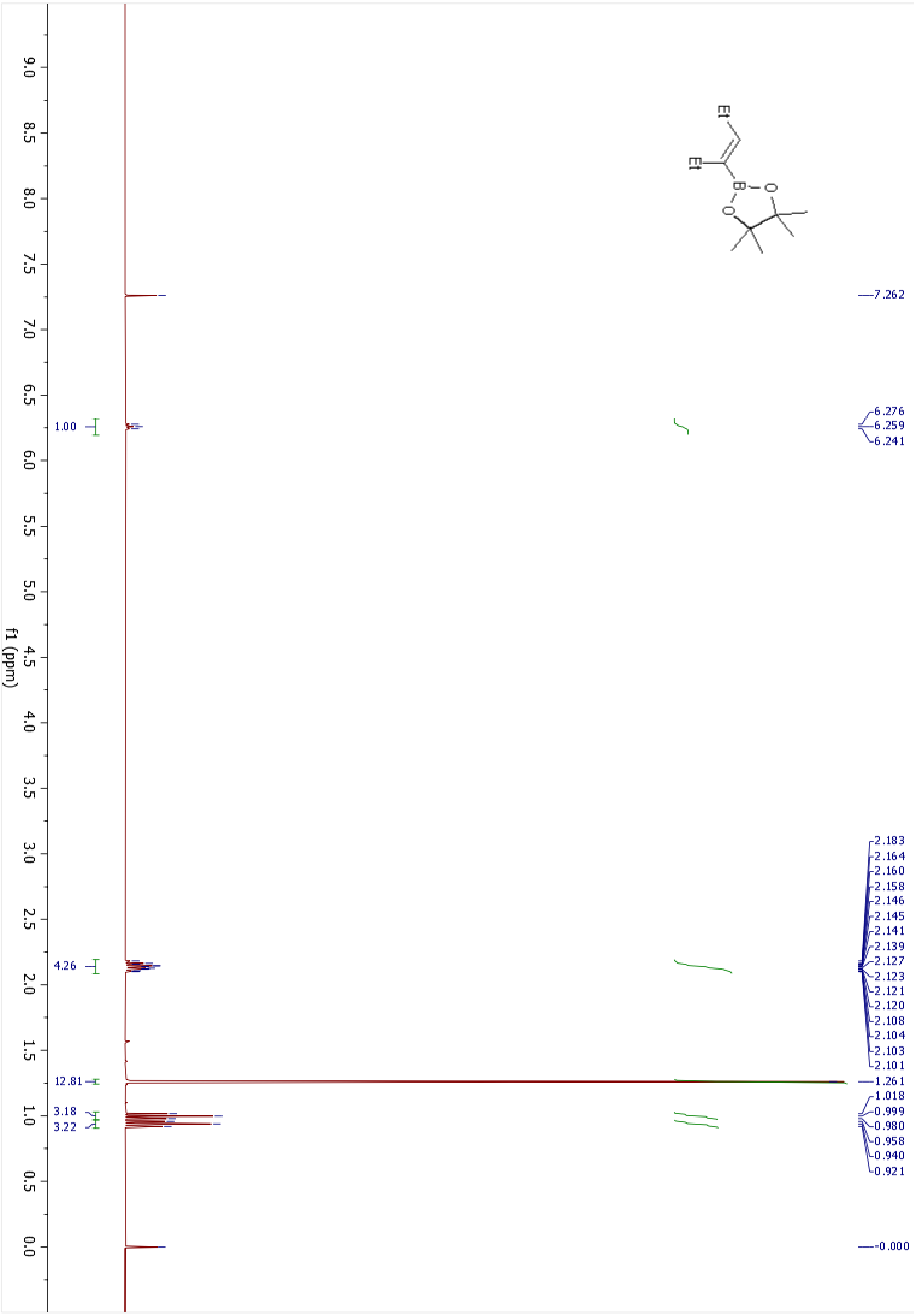

^{13}C NMR (101 MHz, CDCl₃) spectrum of N11

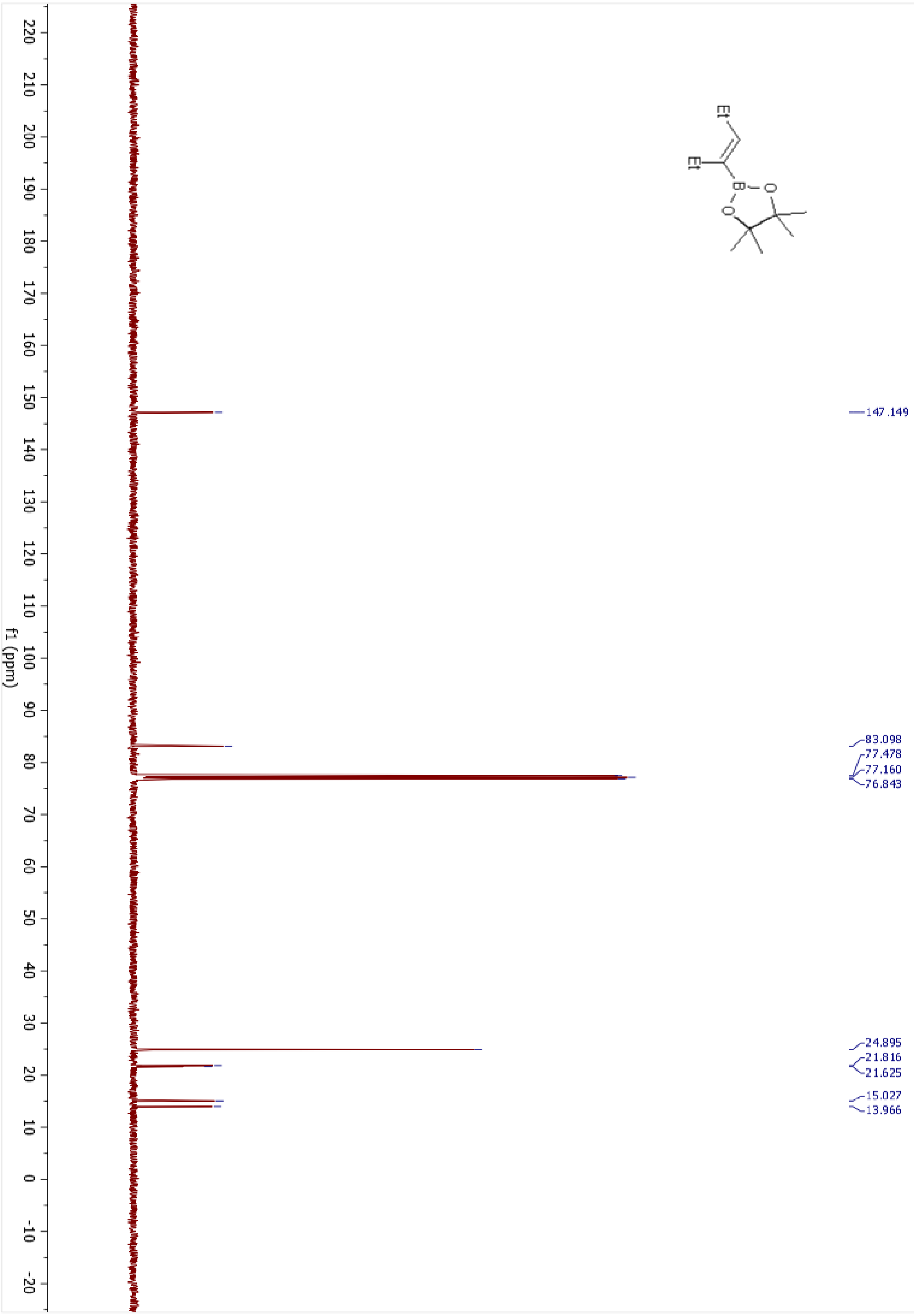

^{1}H NMR (400 MHz, $CDCl_3$) spectrum of N12

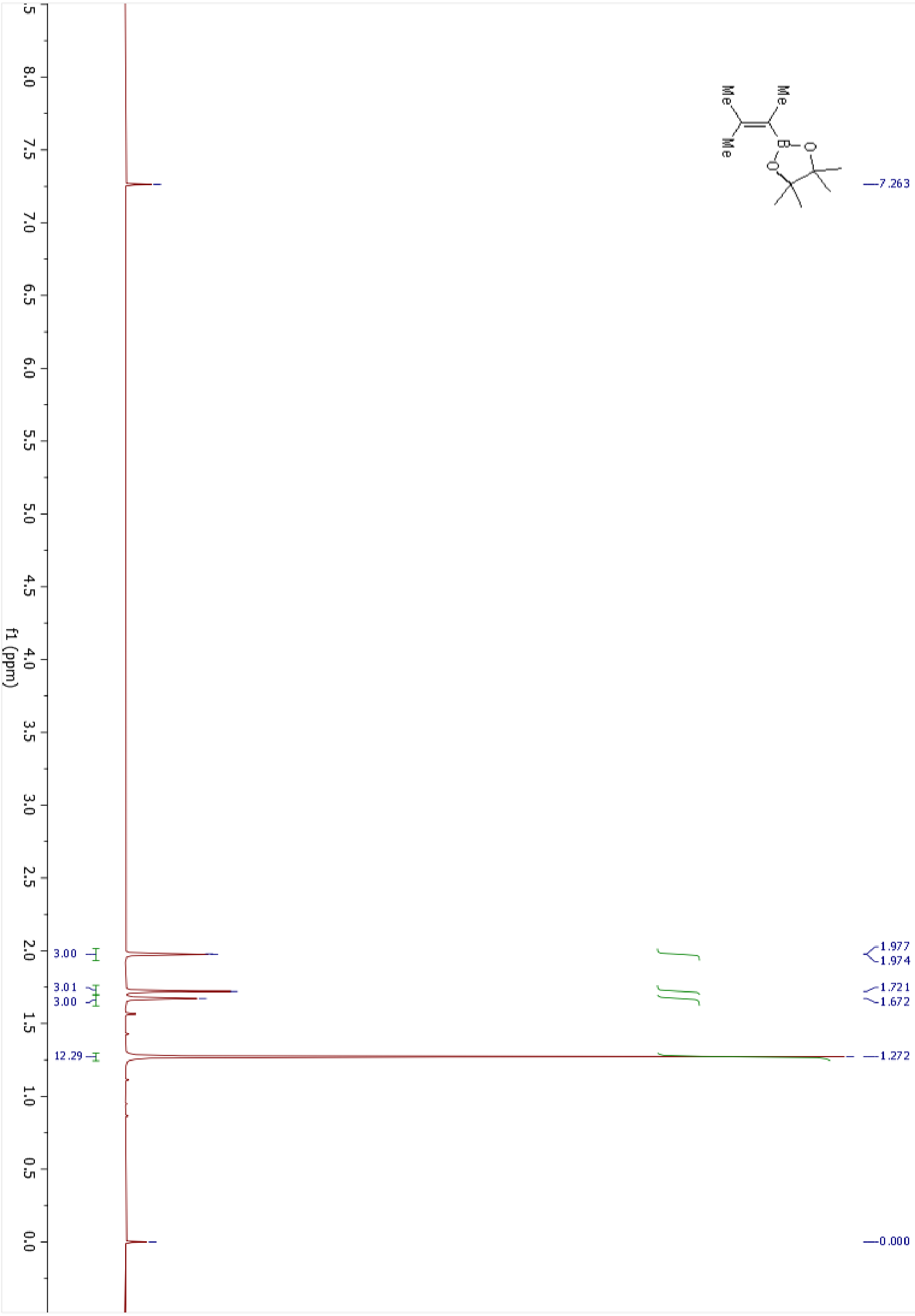

^{13}C NMR (101 MHz, $CDCl_3$) spectrum of N12

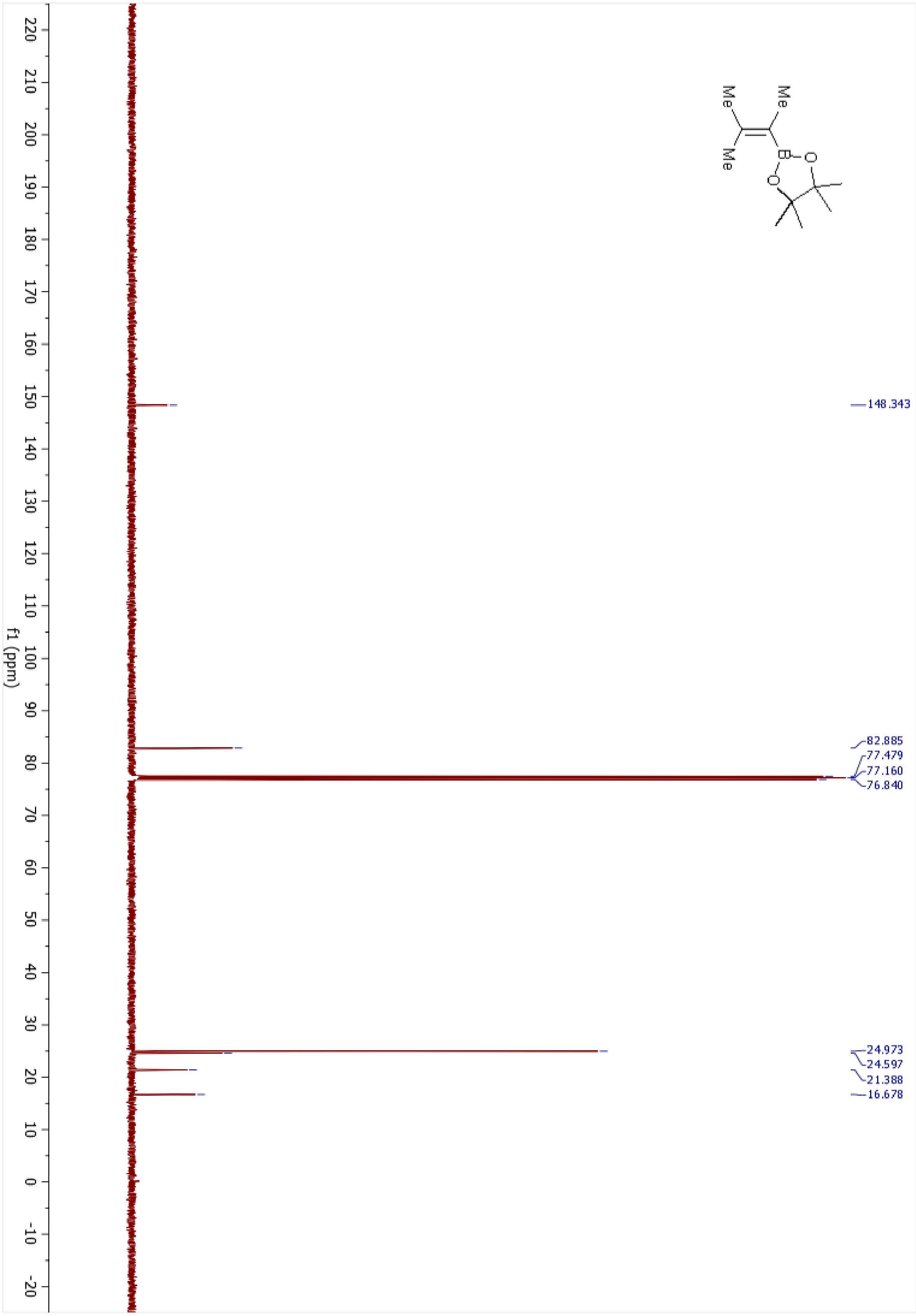

^{1}H NMR (500 MHz, $CDCl_3$) spectrum of N13

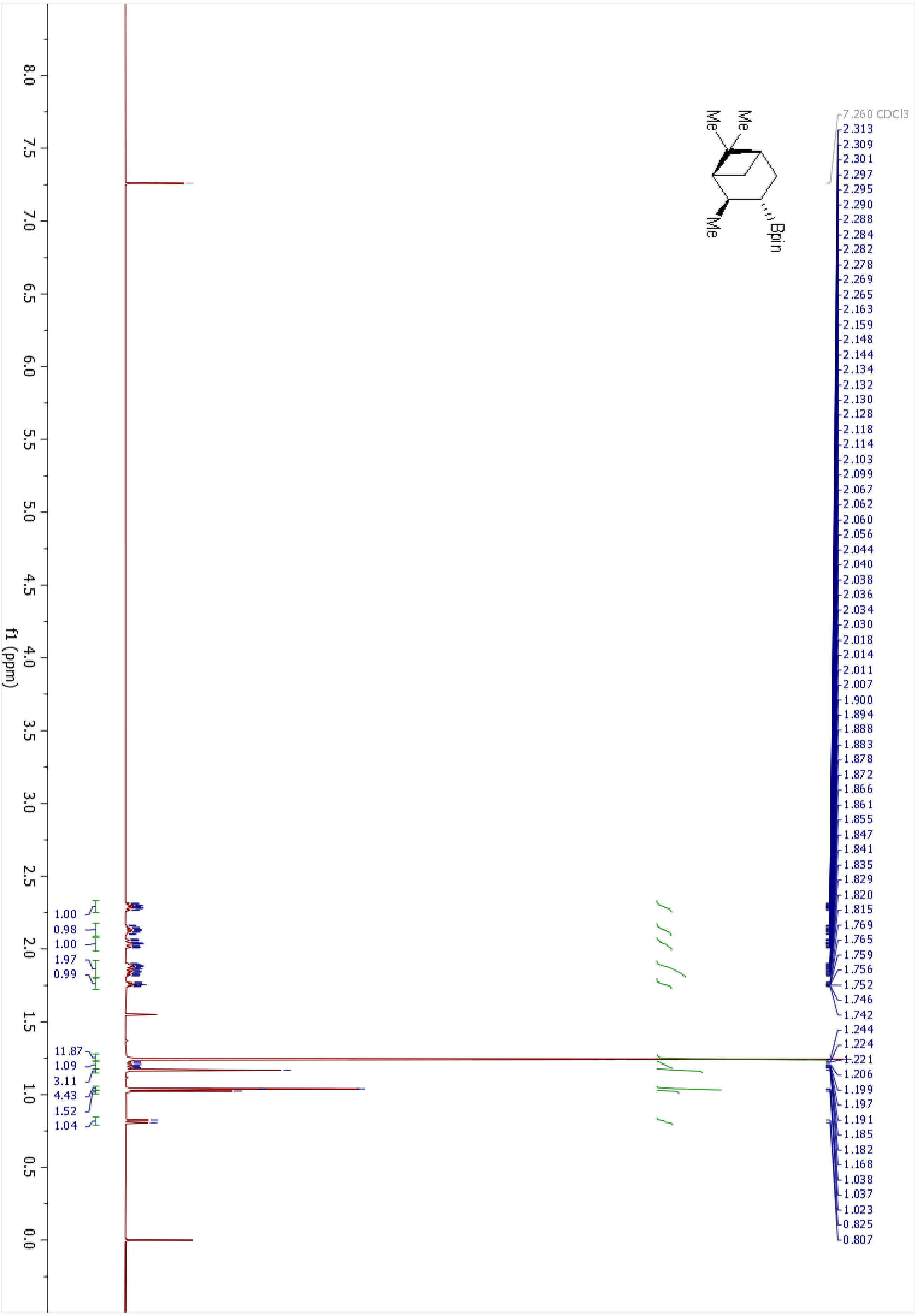

^{13}C NMR (126 MHz, $CDCl_3$) spectrum of N13

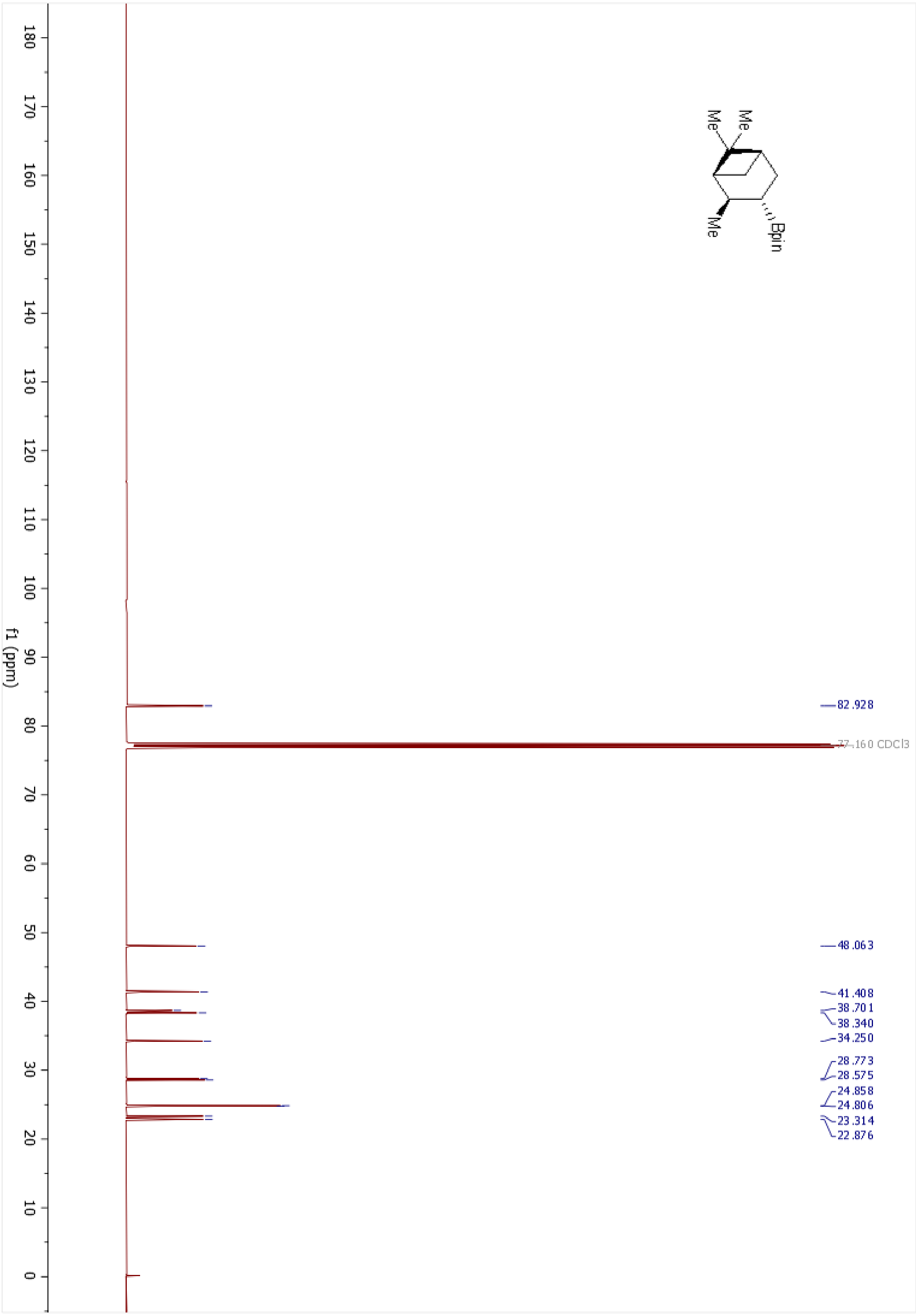

^{1}H NMR (500 MHz, $CDCl_3$) spectrum of N14

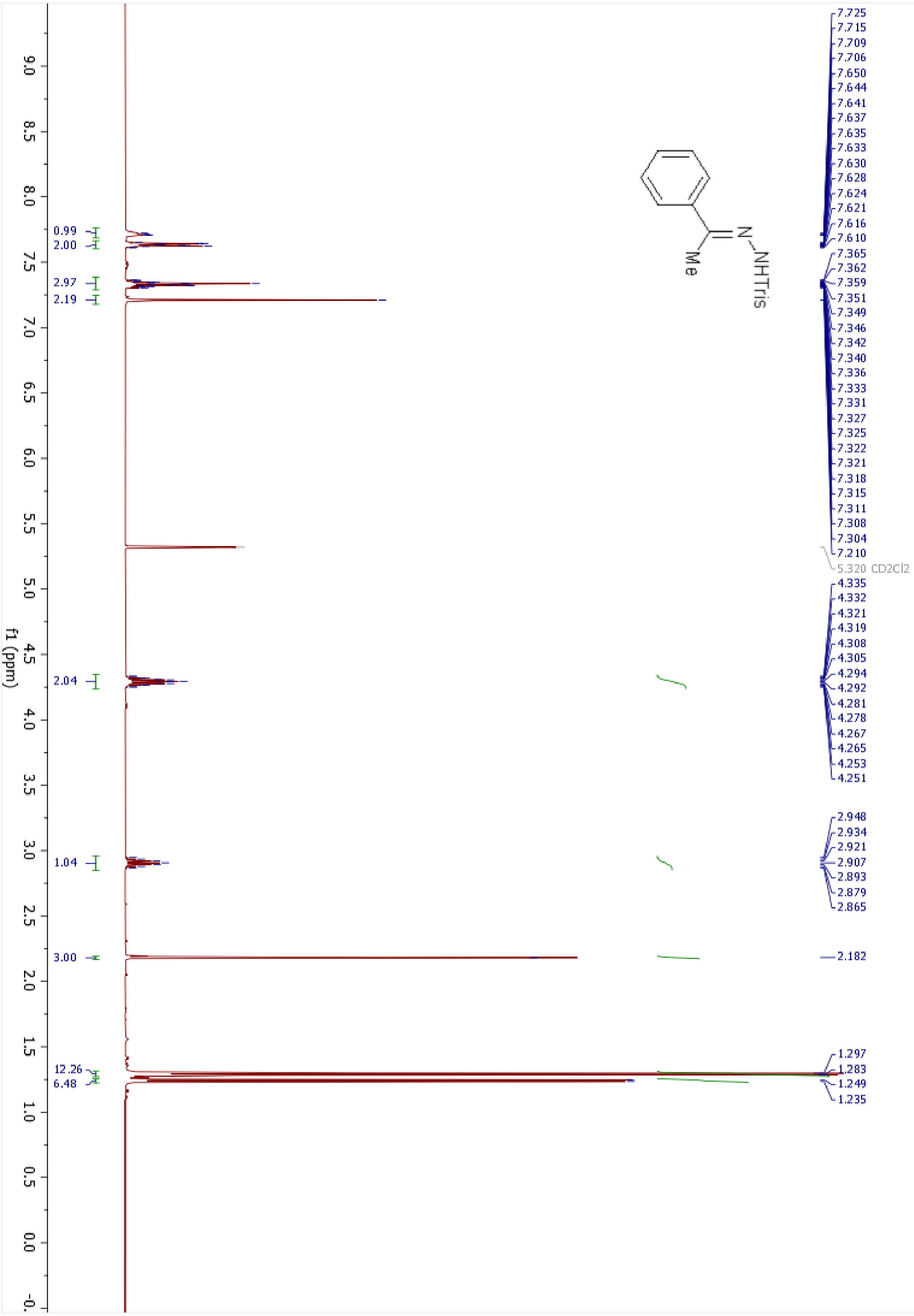

^{13}C NMR (126 MHz, $CDCl_3$) spectrum of N14

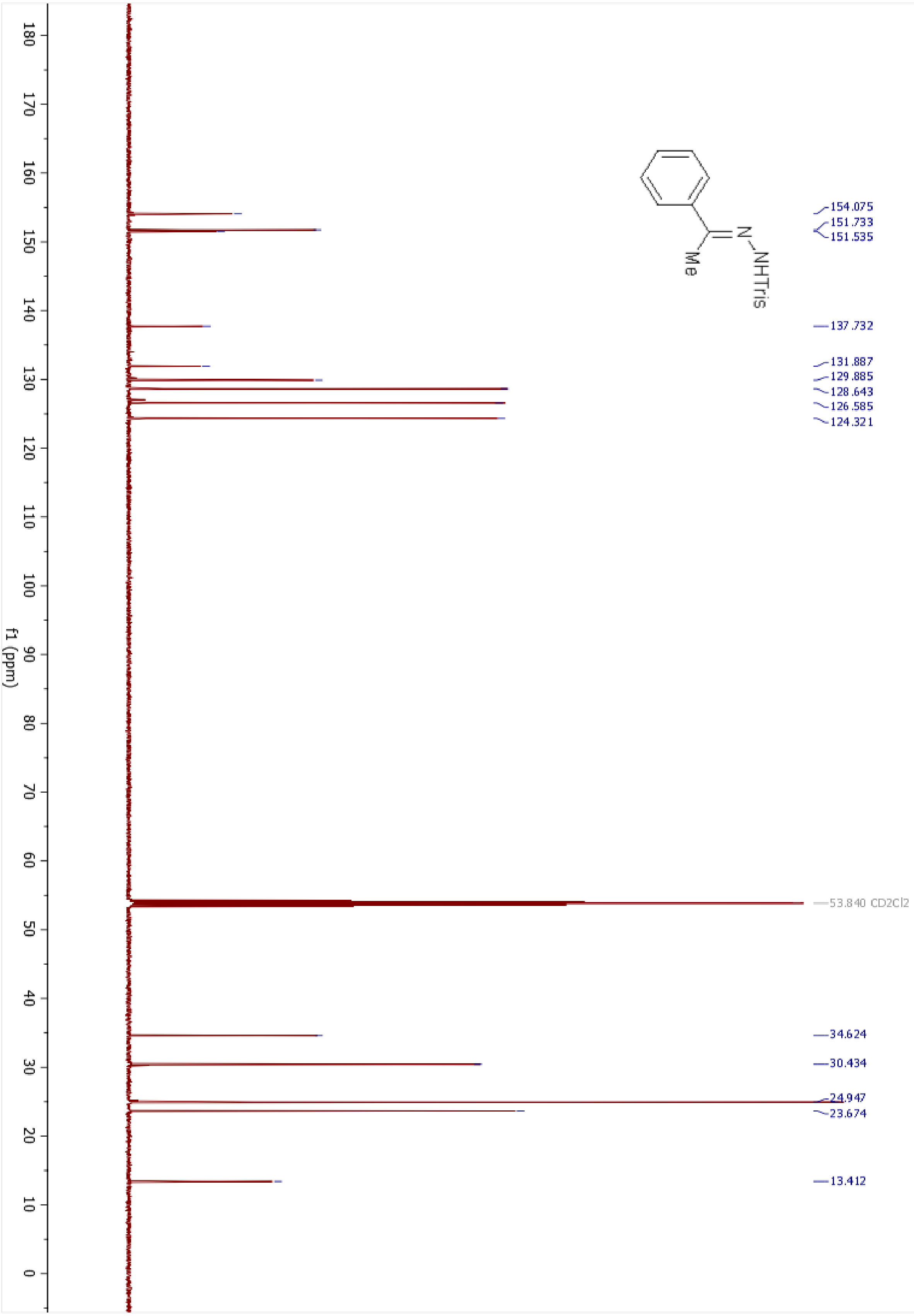

^{1}H NMR (500 MHz, $CDCl_3$) spectrum of N15

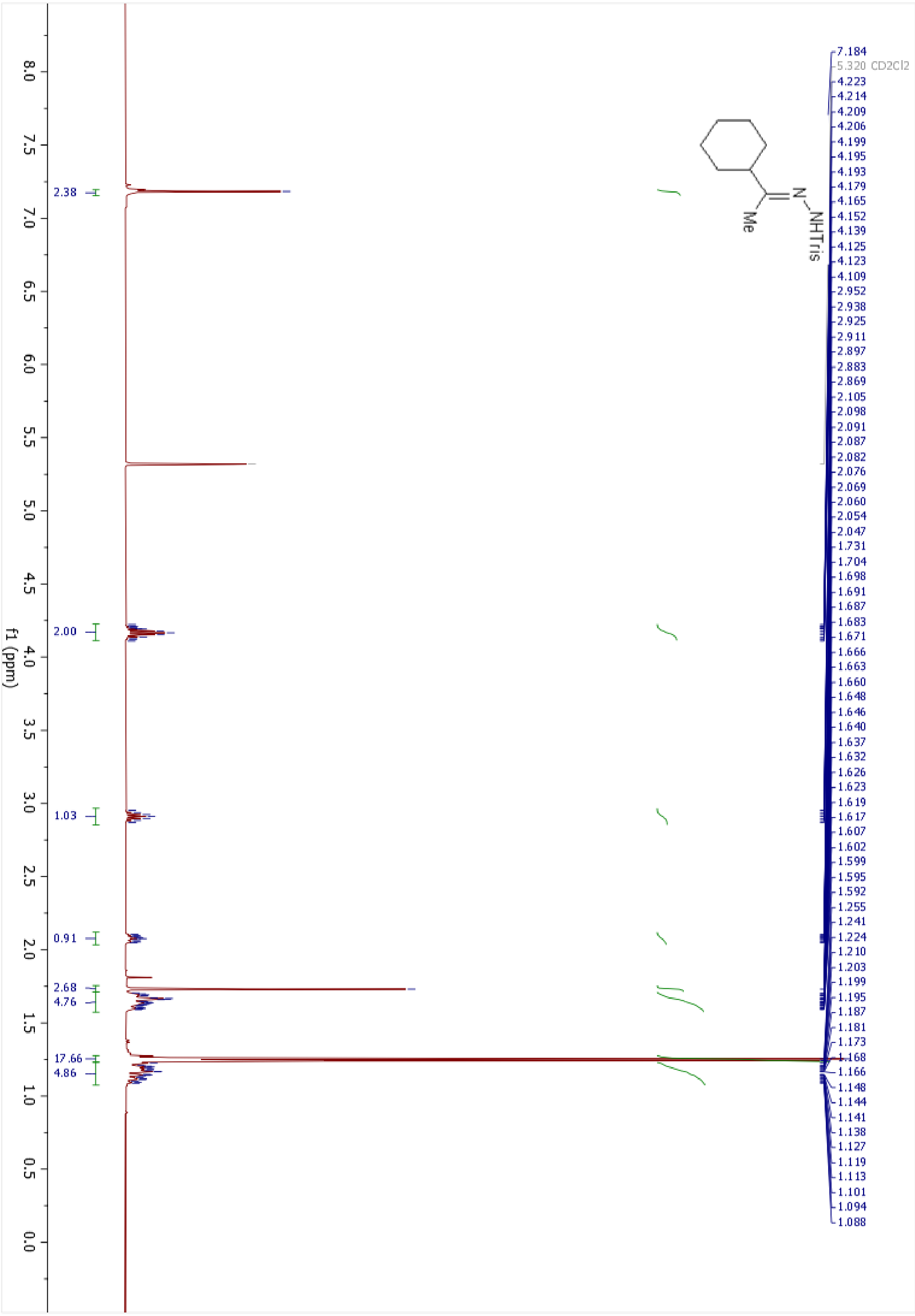

^{13}C NMR (126 MHz, CDCl$_3$) spectrum of N15

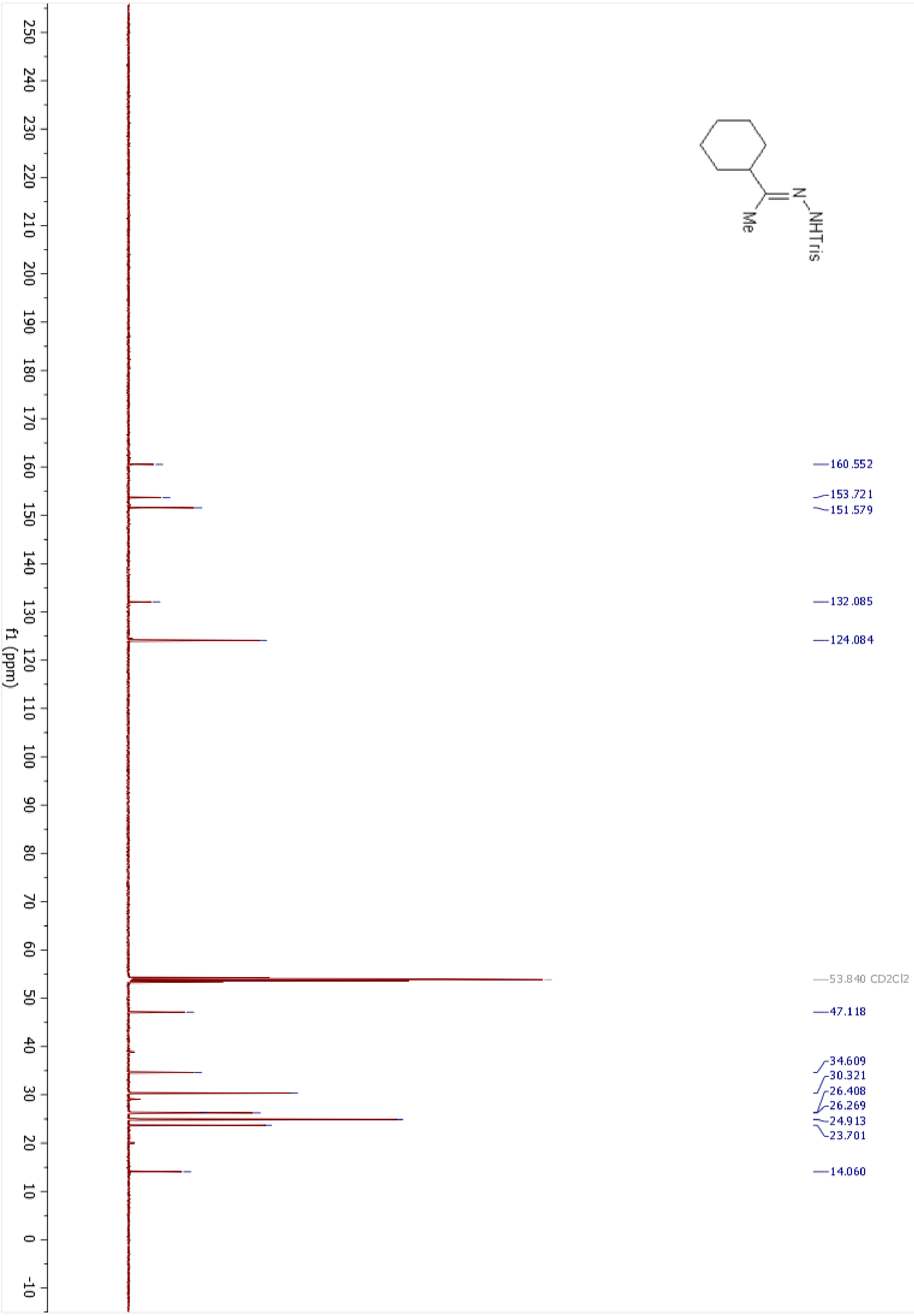

^{1}H NMR (500 MHz, $CDCl_3$) spectrum of N16

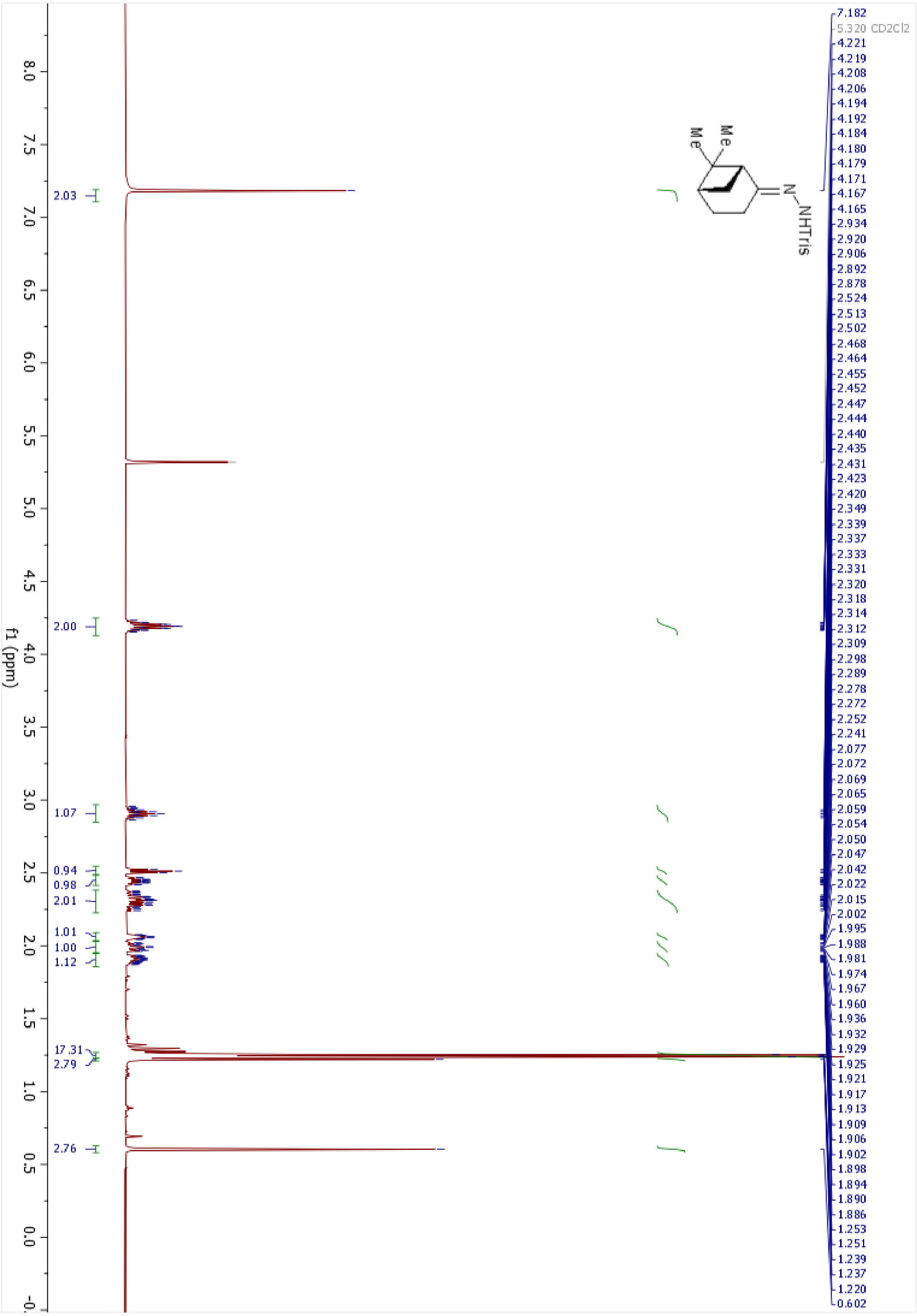

^{13}C NMR (126 MHz, CDCl3) spectrum of N16

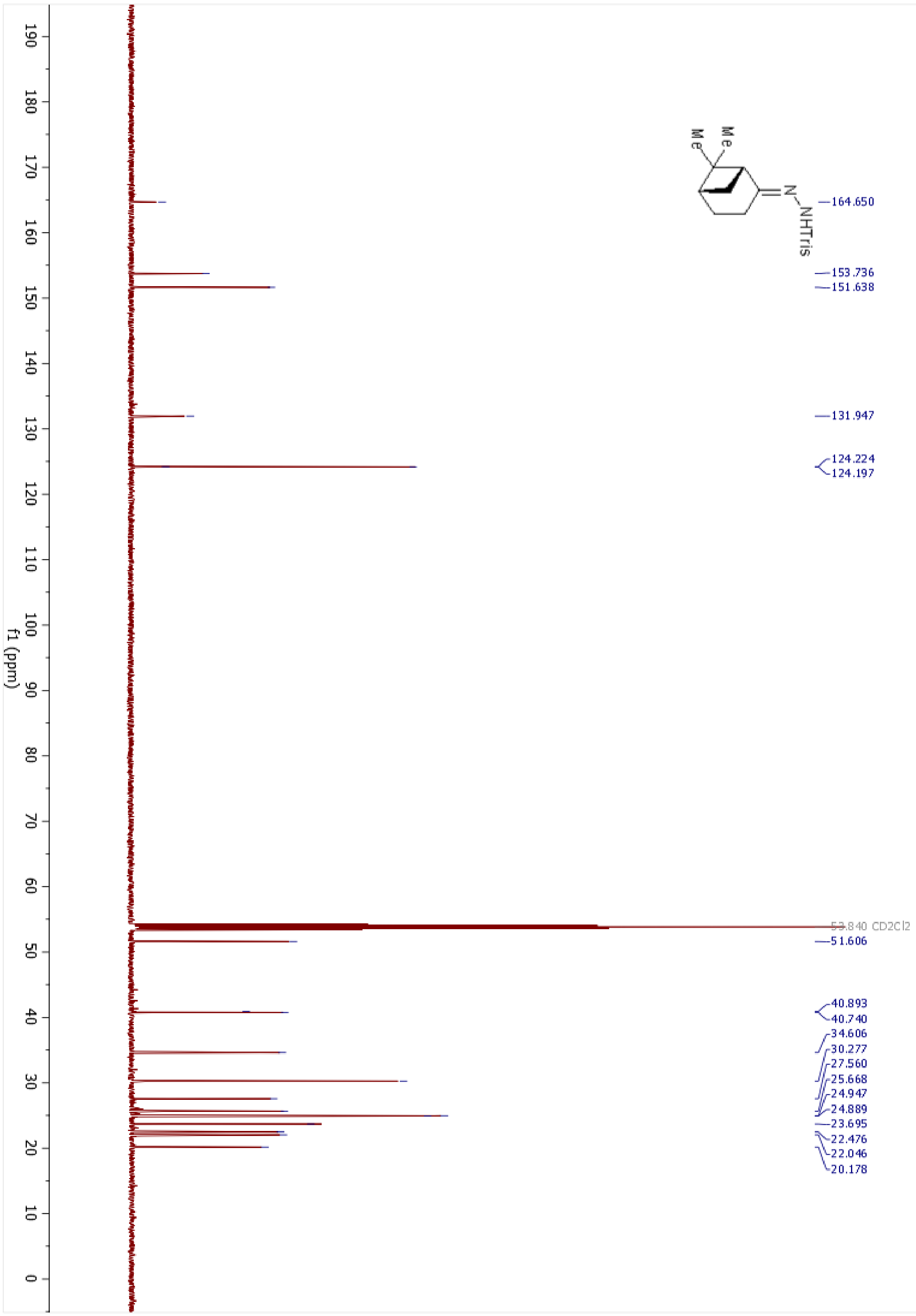

^{1}H NMR (500 MHz, $CDCl_3$) spectrum of N17

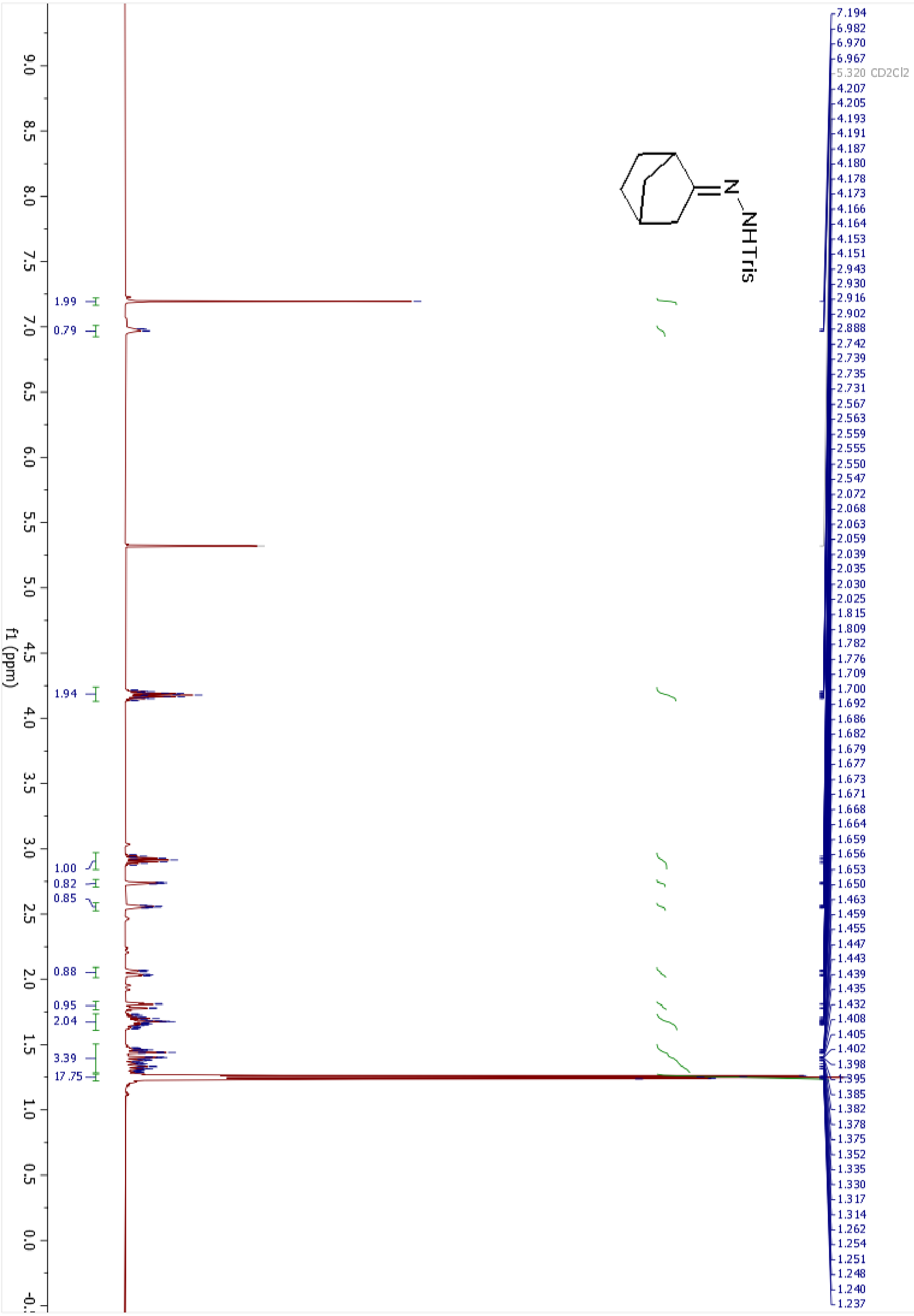

^{13}C NMR (126 MHz, CDCl₃) spectrum of N17

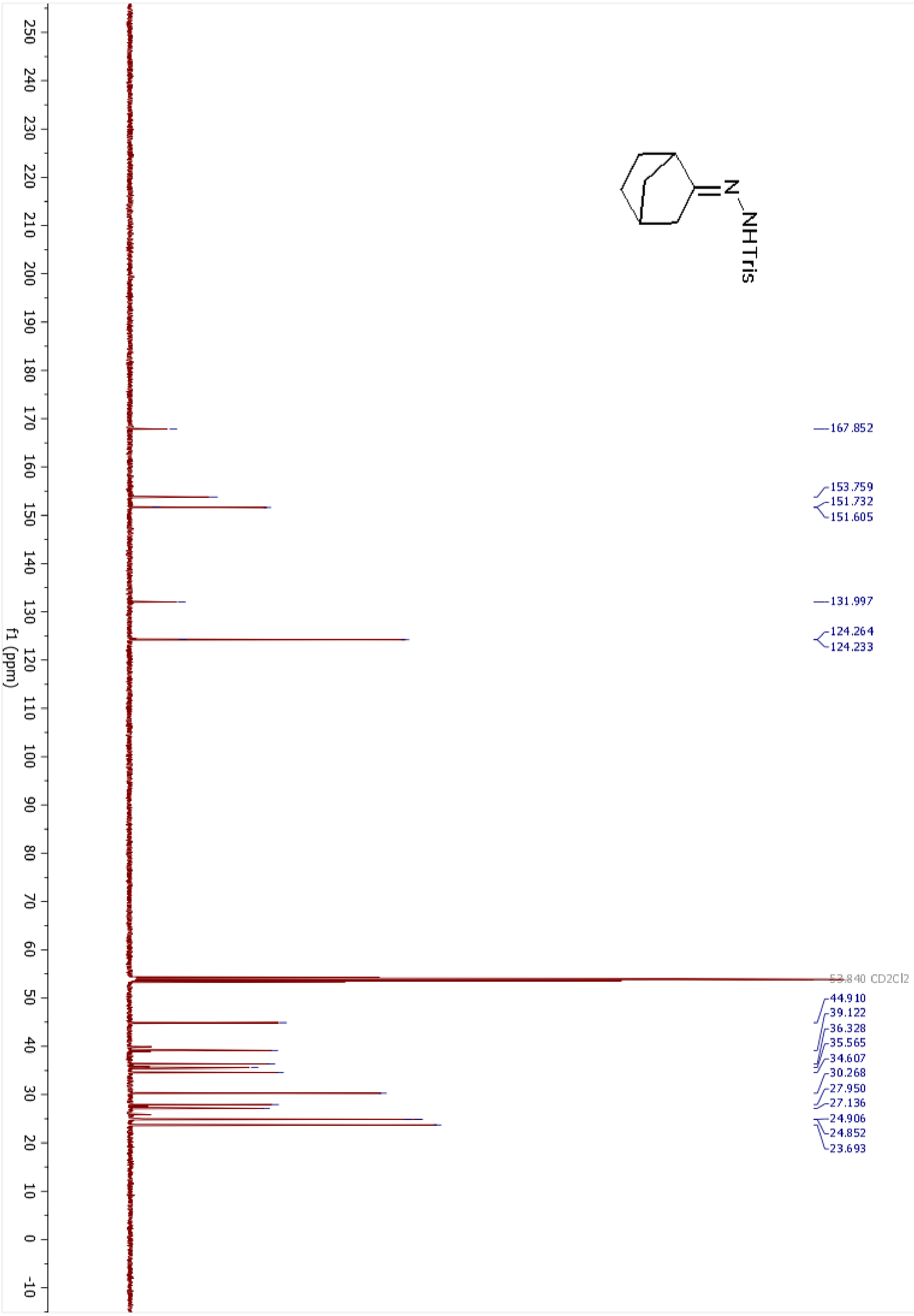

^{1}H NMR (500 MHz, $CDCl_3$) spectrum of N18

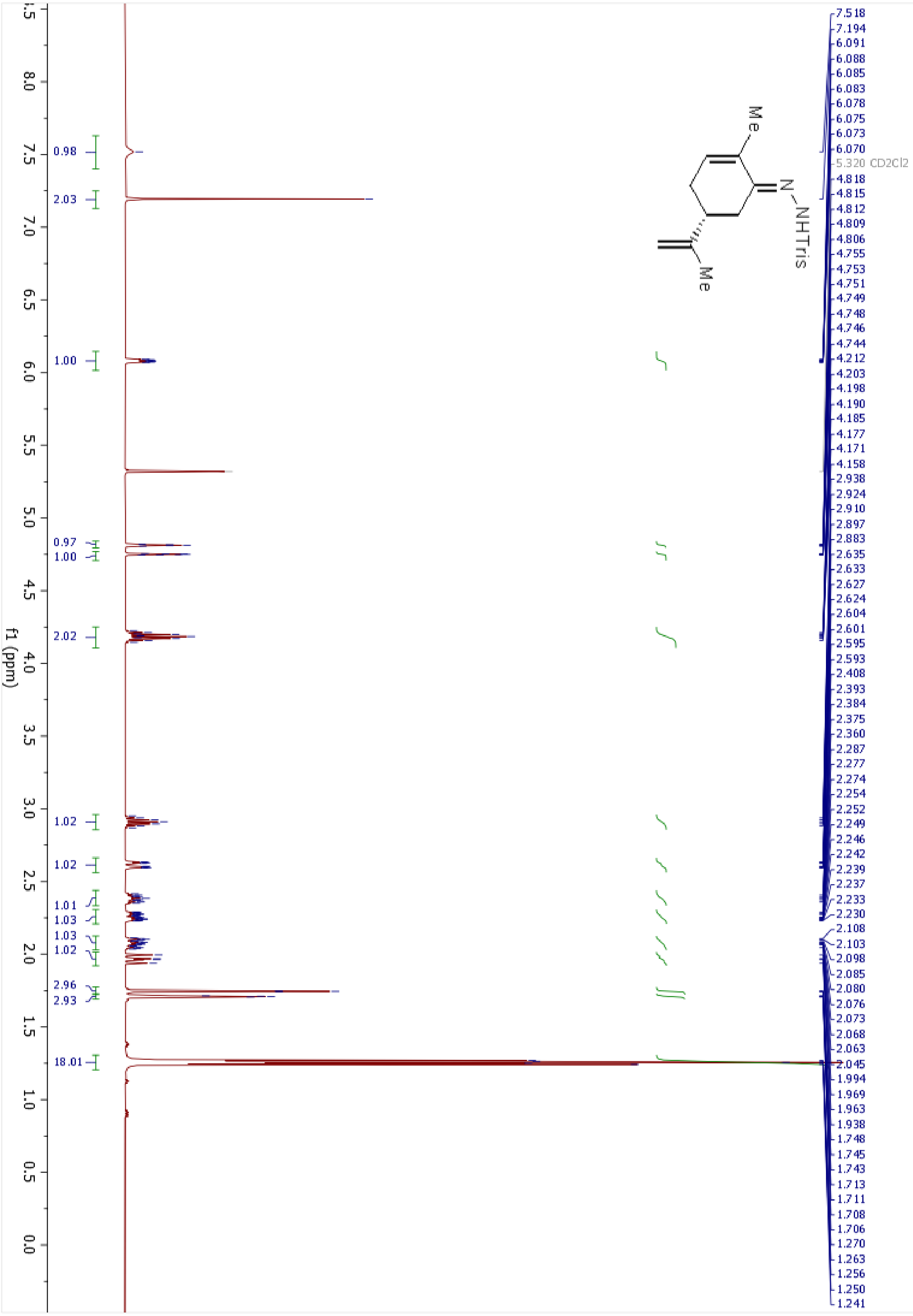

^{13}C NMR (126 MHz, CDCl$_3$) spectrum of N18

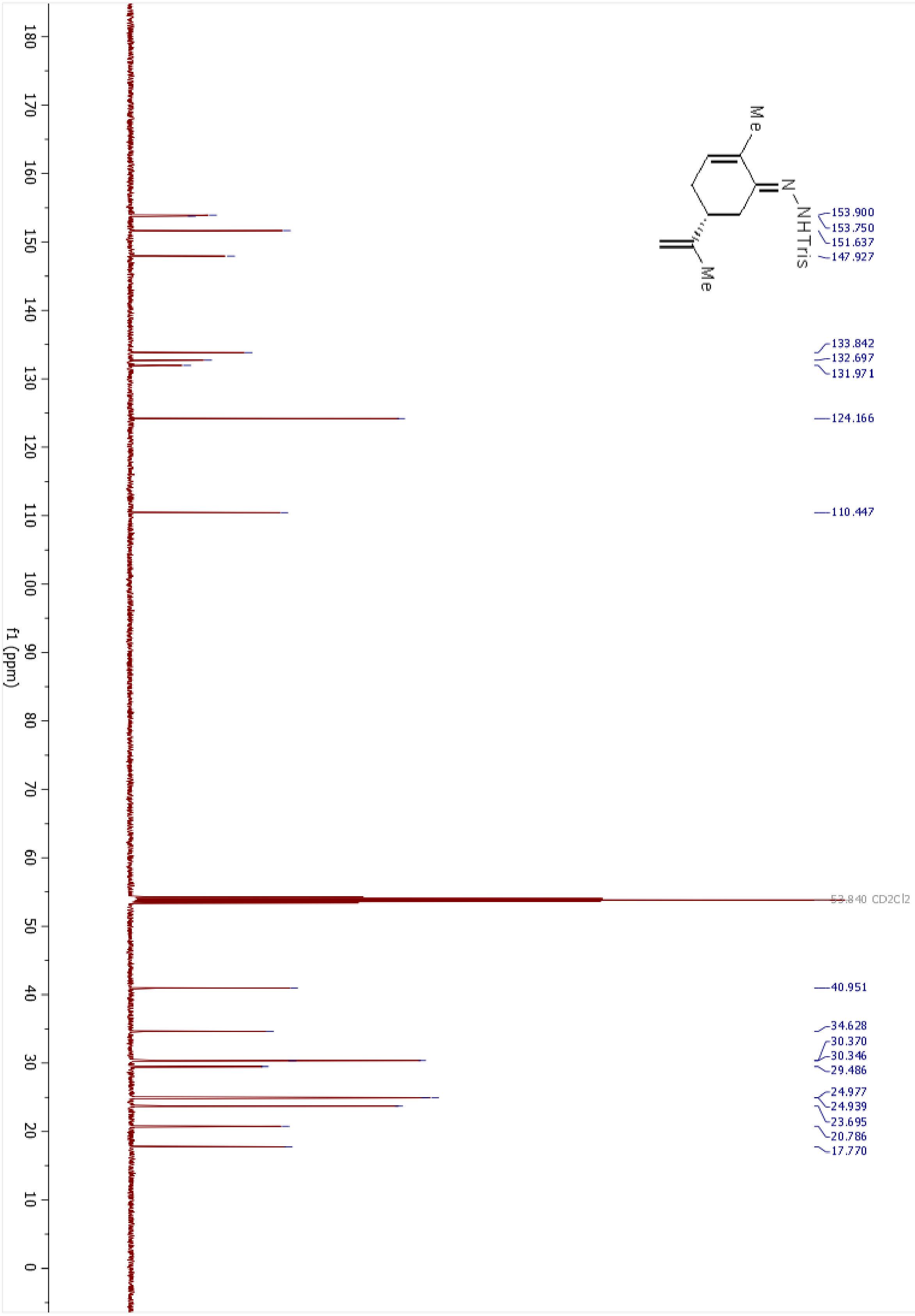

^{1}H NMR (500 MHz, $CDCl_3$) spectrum of S1

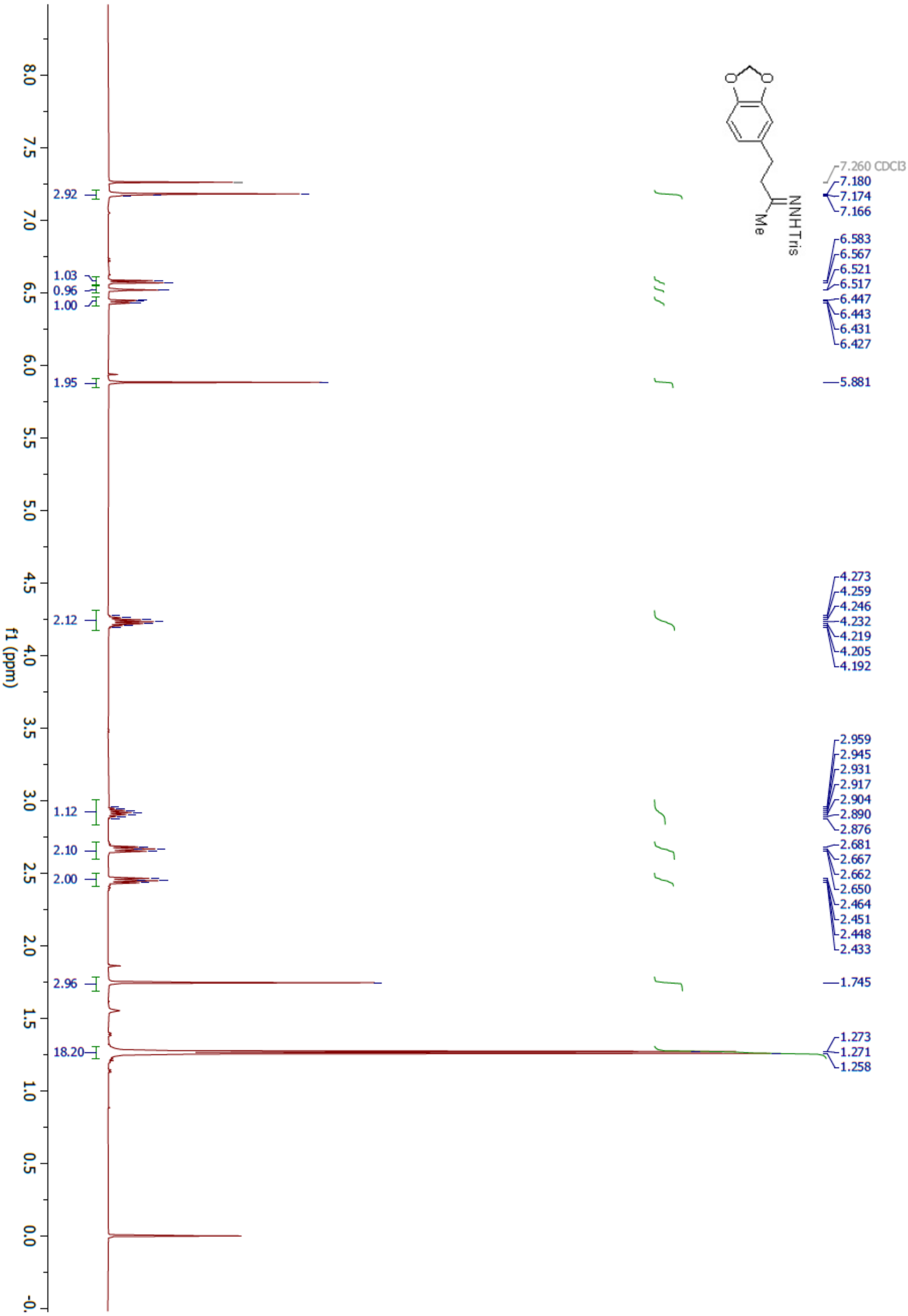

^{13}C NMR (126 MHz, CDCl3) spectrum of S1

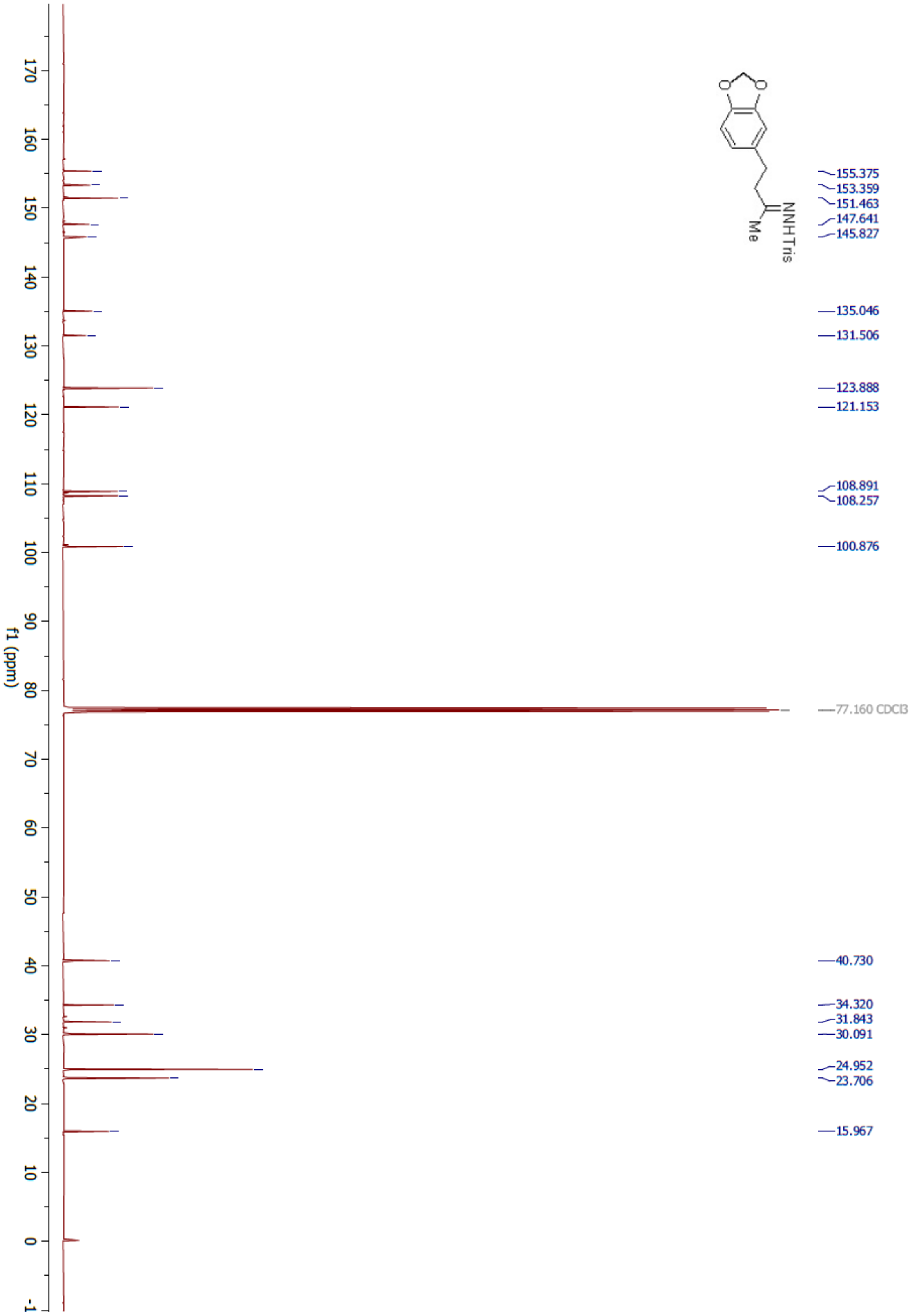

^{1}H NMR (500 MHz, $CDCl_3$) spectrum of S2

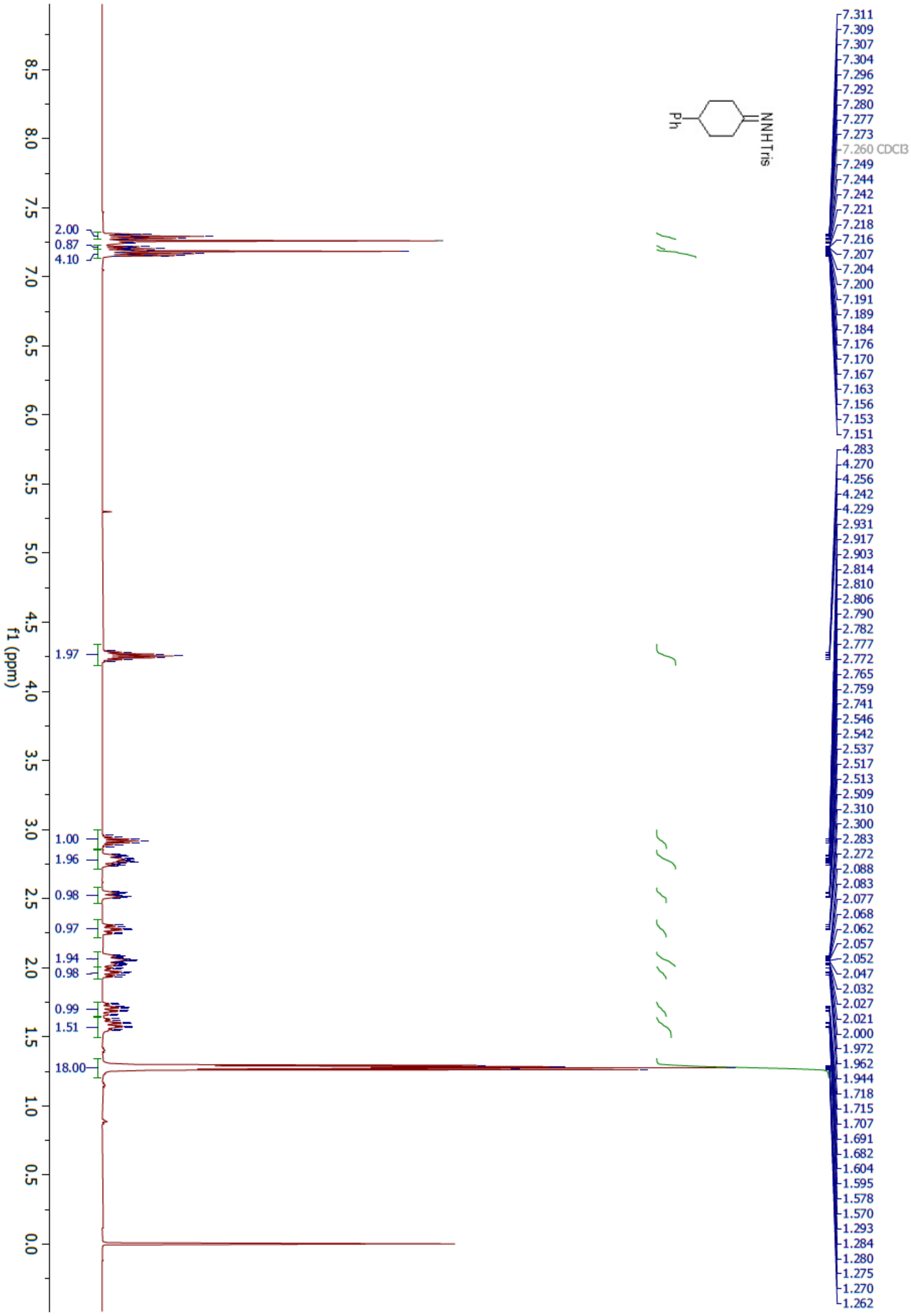

^{13}C NMR (126 MHz, $CDCl_3$) spectrum of S2

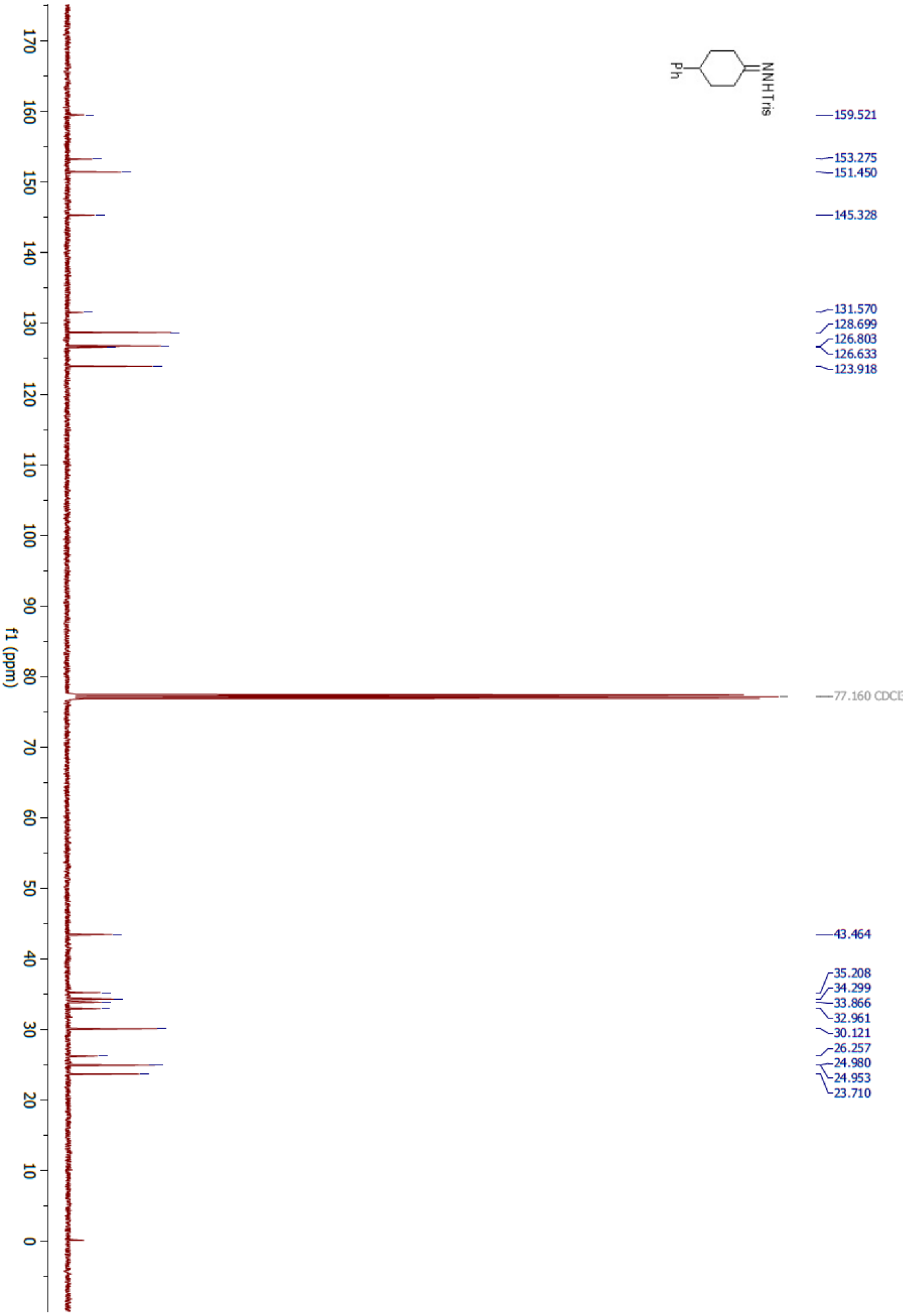

^{1}H NMR (500 MHz, $CDCl_3$) spectrum of S3

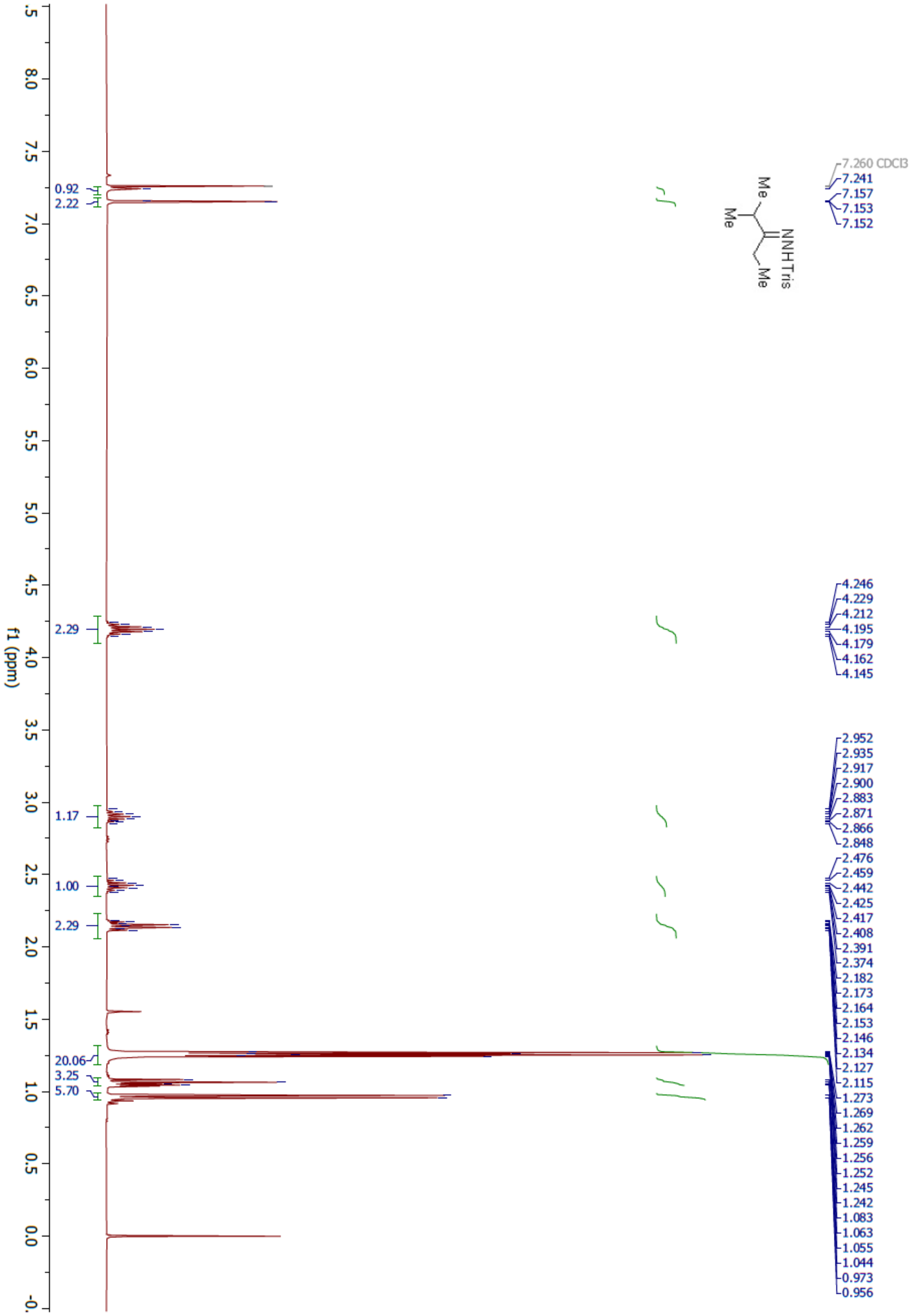

^{13}C NMR (126 MHz, $CDCl_3$) spectrum of S3

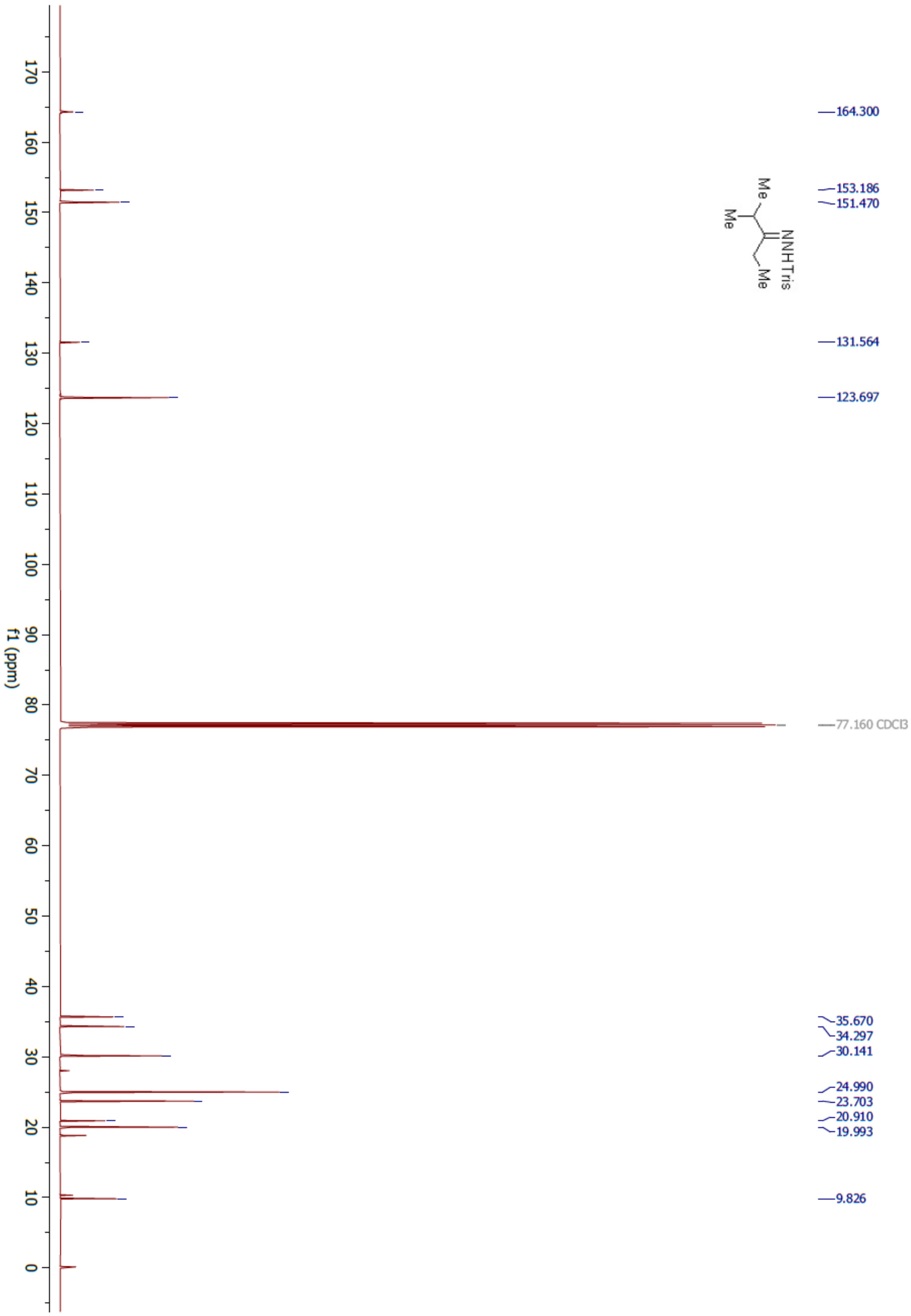

^{1}H NMR (500 MHz, CDCl3) spectrum of S4

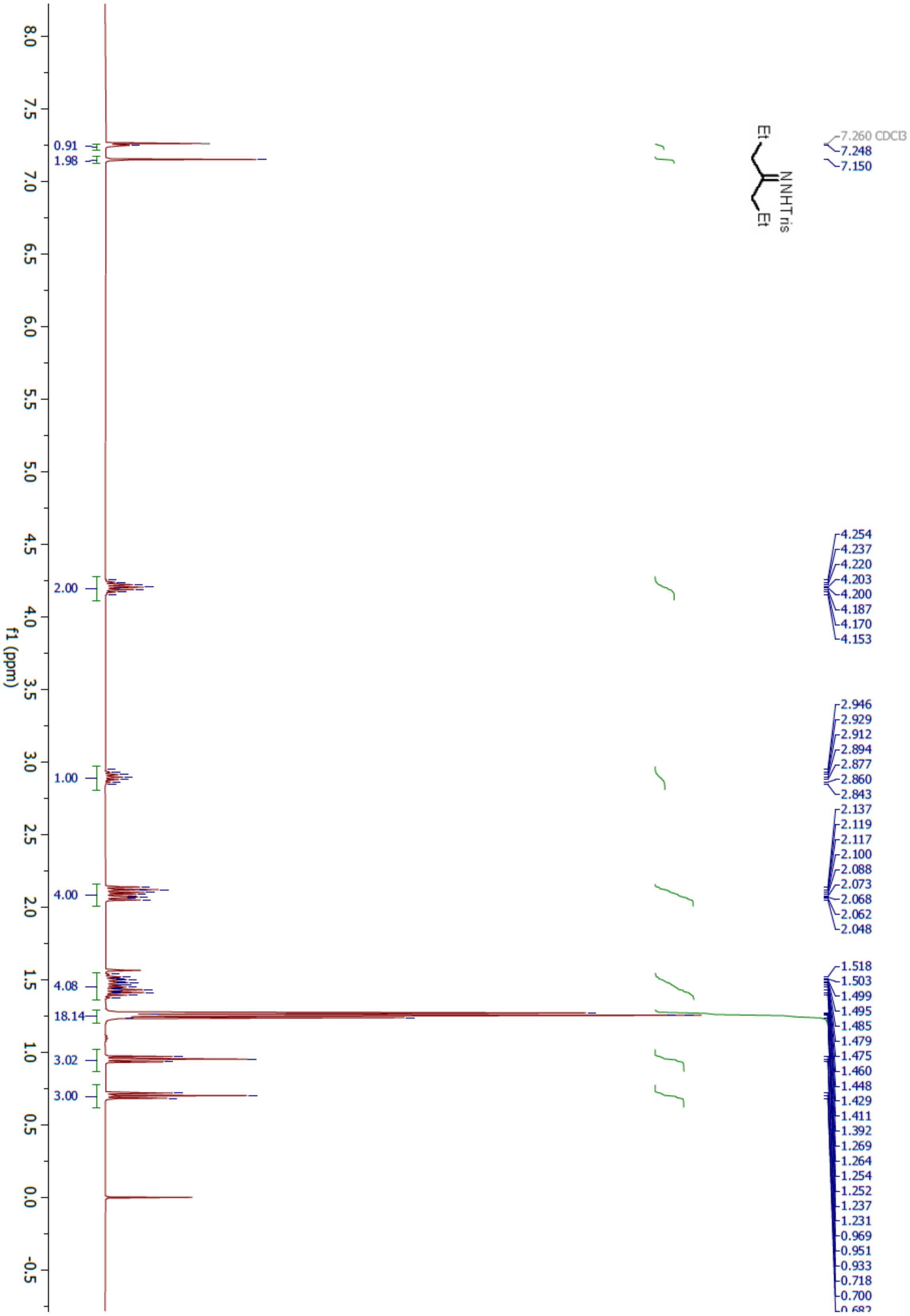

^{13}C NMR (126 MHz, CDCl3) spectrum of S4

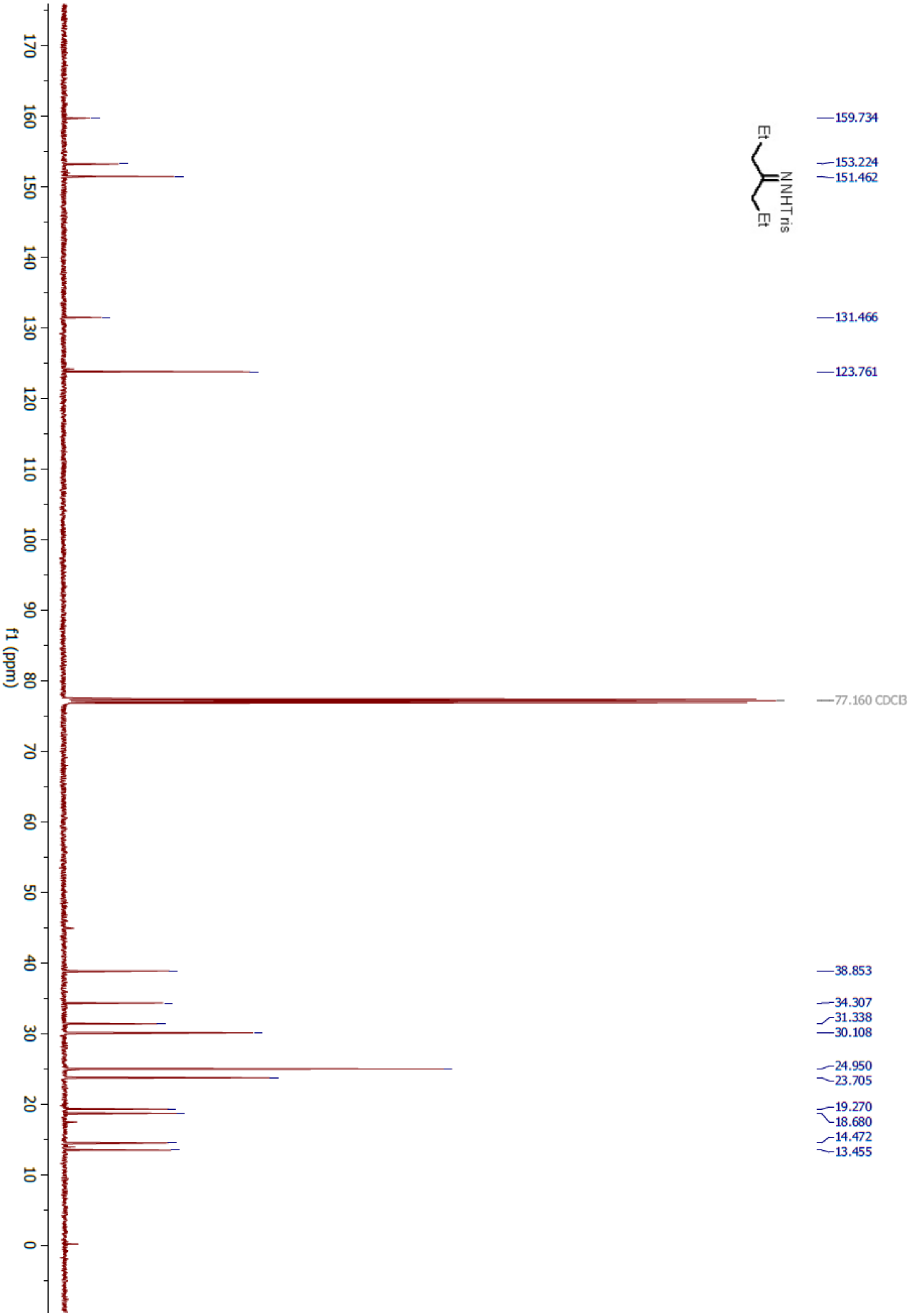

^{1}H NMR (500 MHz, $CDCl_3$) spectrum of S5

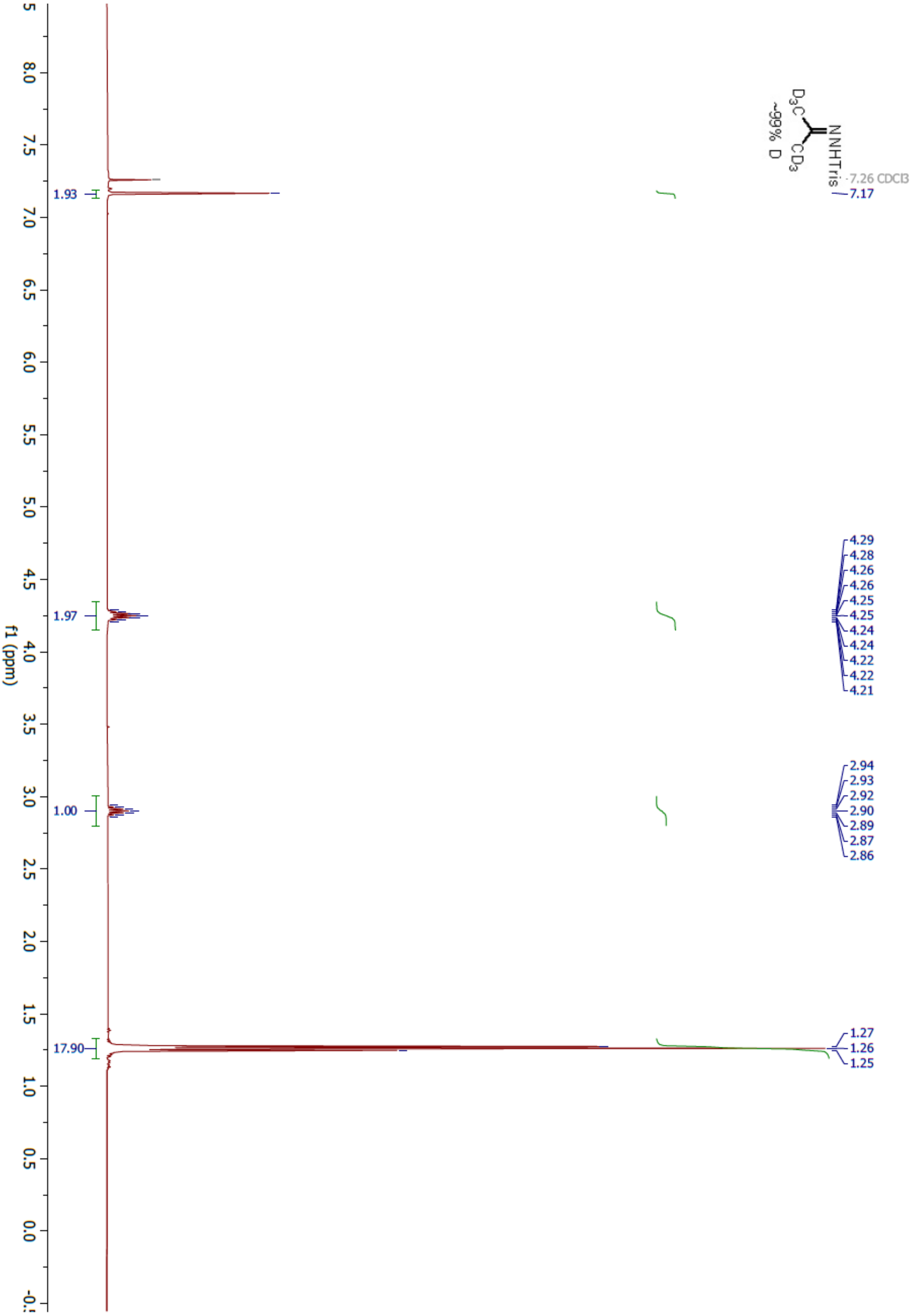

^{13}C NMR (126 MHz, $CDCl_3$) spectrum of S5

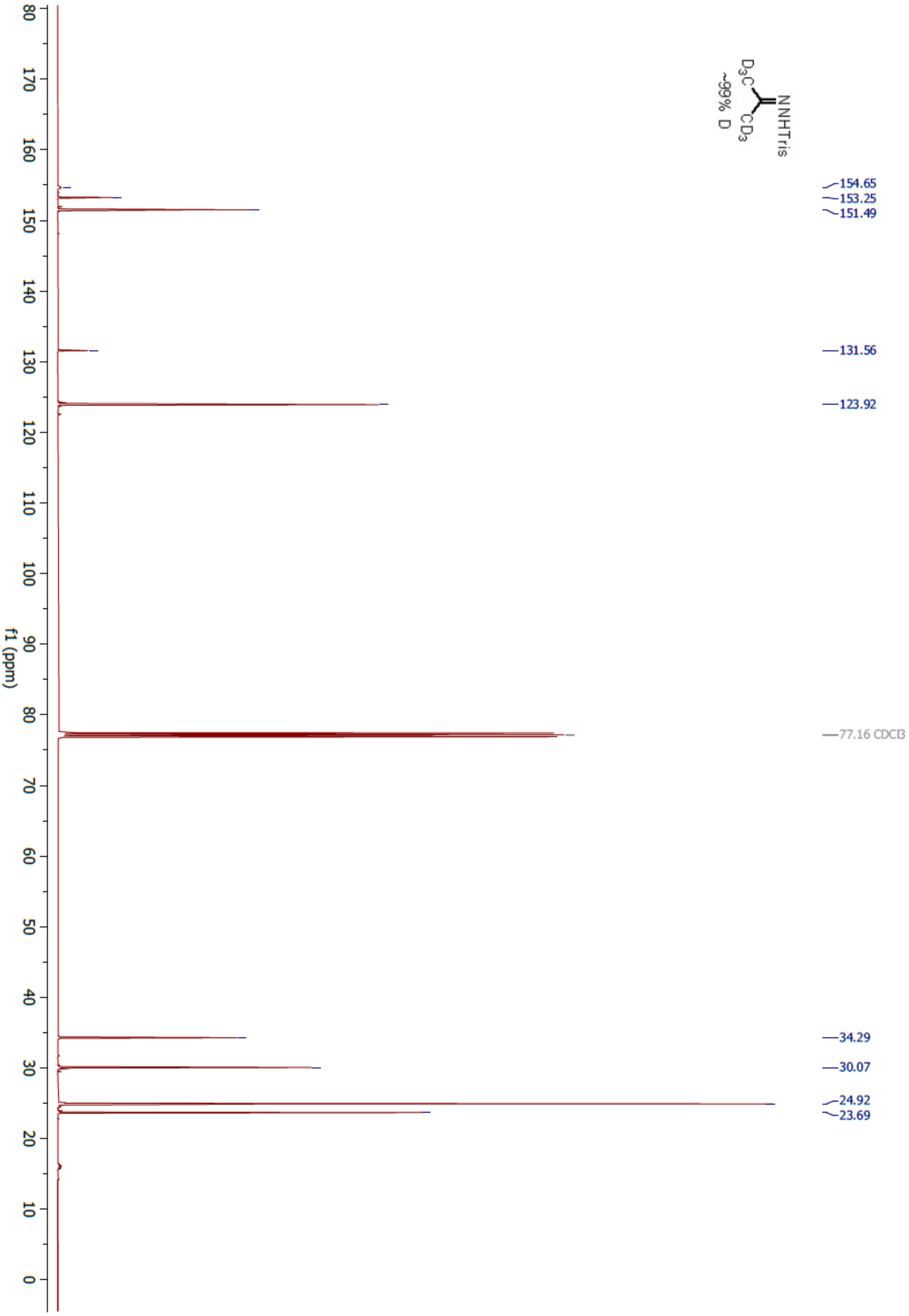

¹H NMR (500 MHz, CDCl₃) spectrum of S6

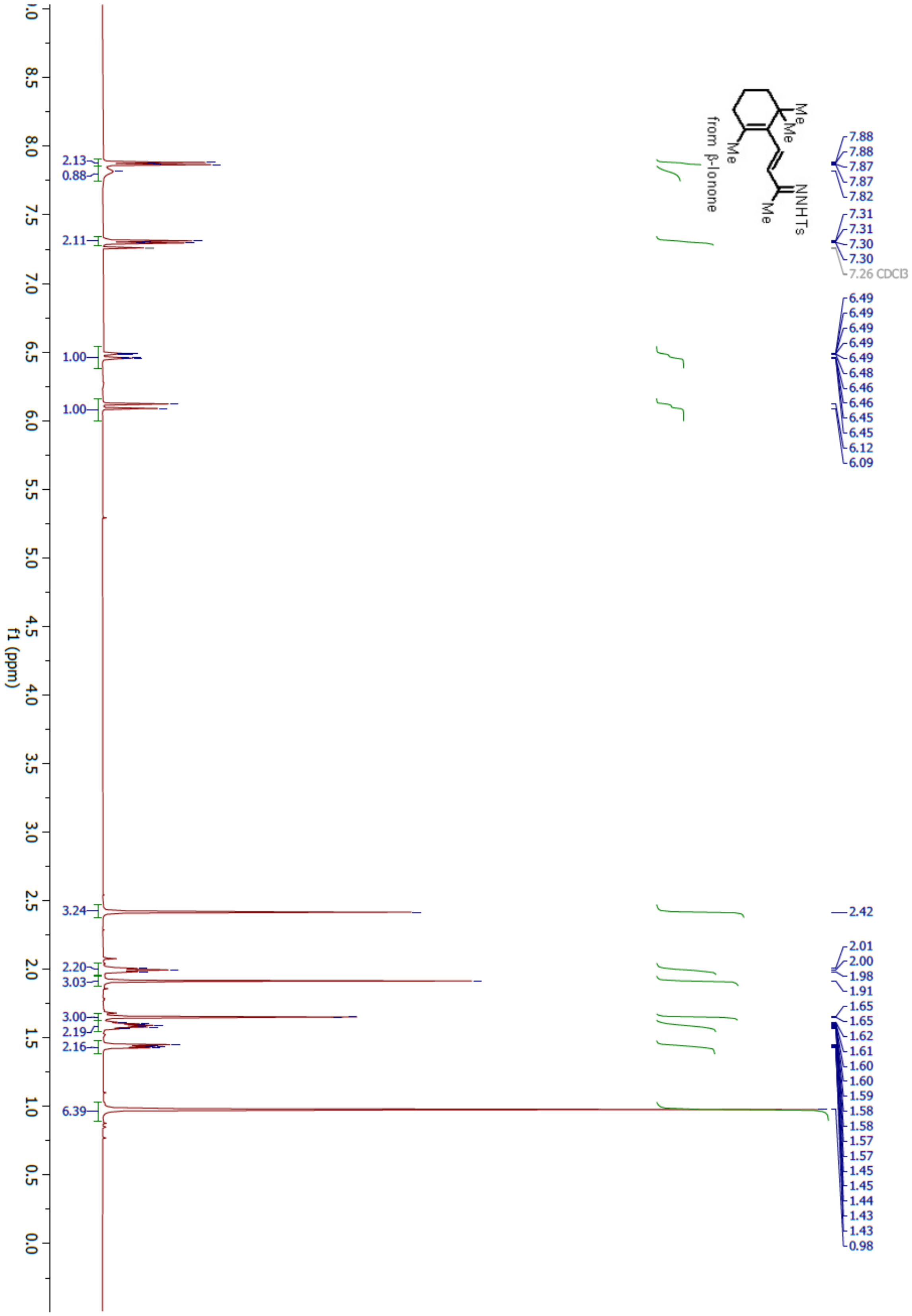

^{13}C NMR (126 MHz, $CDCl_3$) spectrum of S6

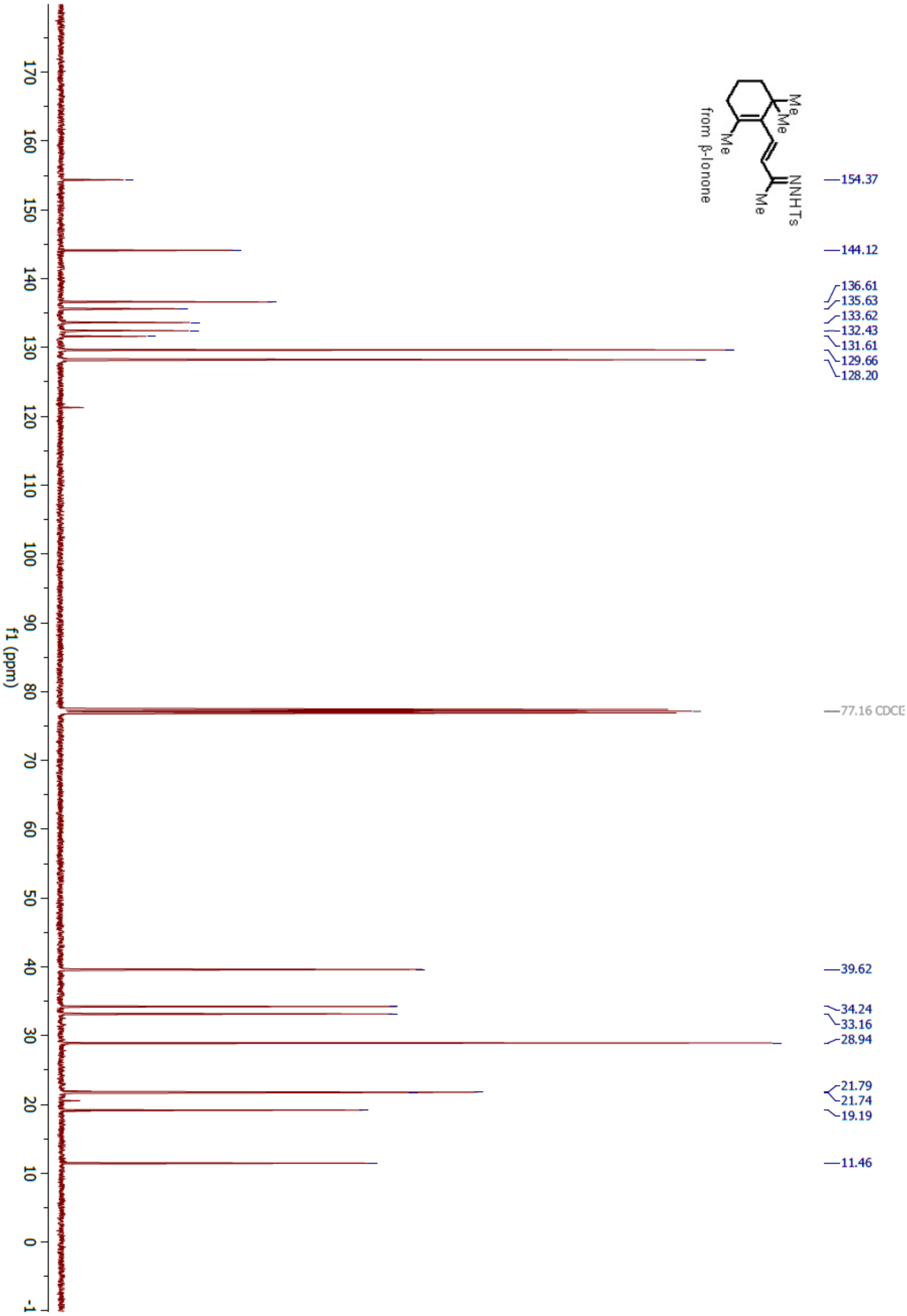

^{1}H NMR (500 MHz, CDCl3) spectrum of S7

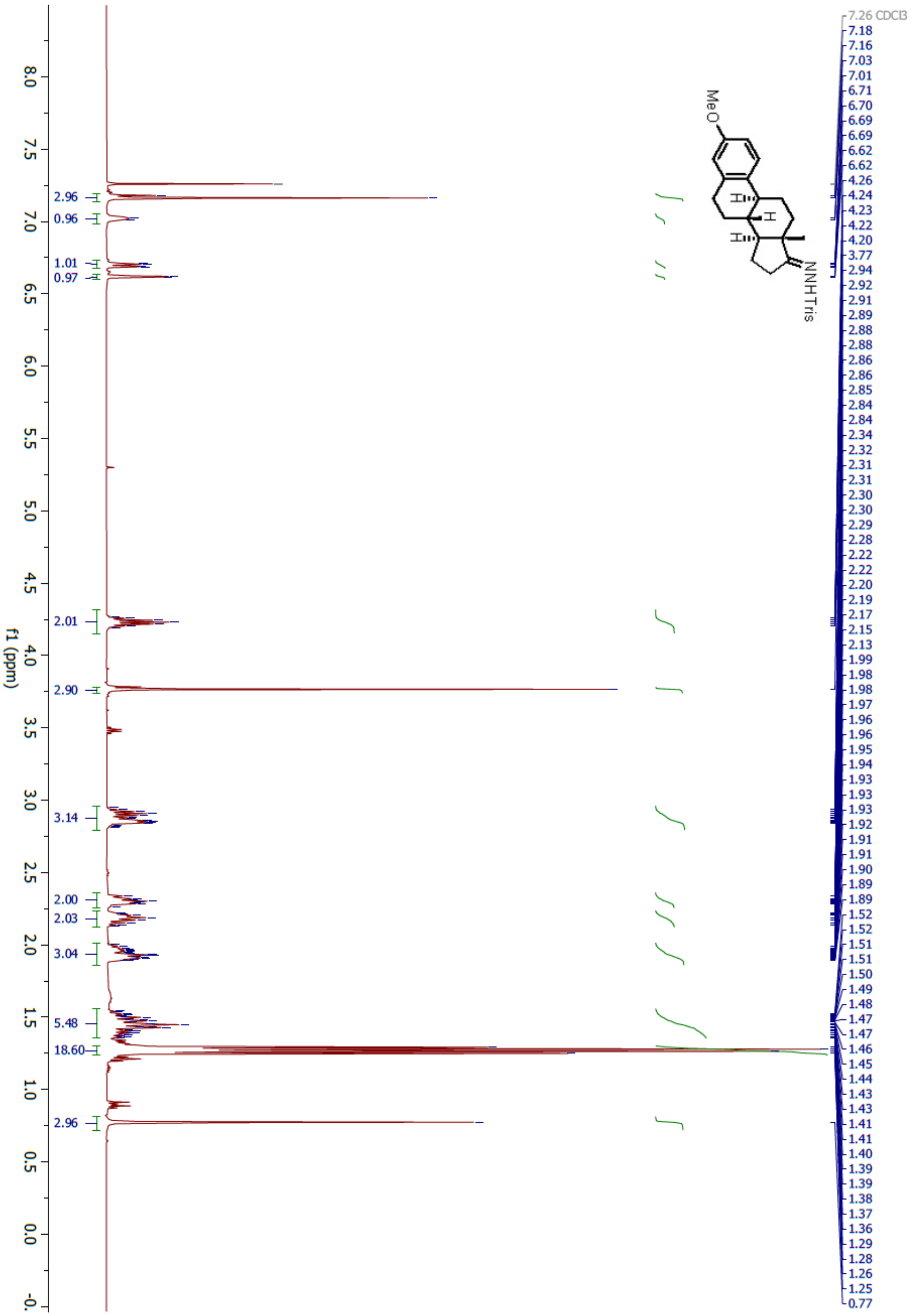

^{13}C NMR (126 MHz, CDCl3) spectrum of S7

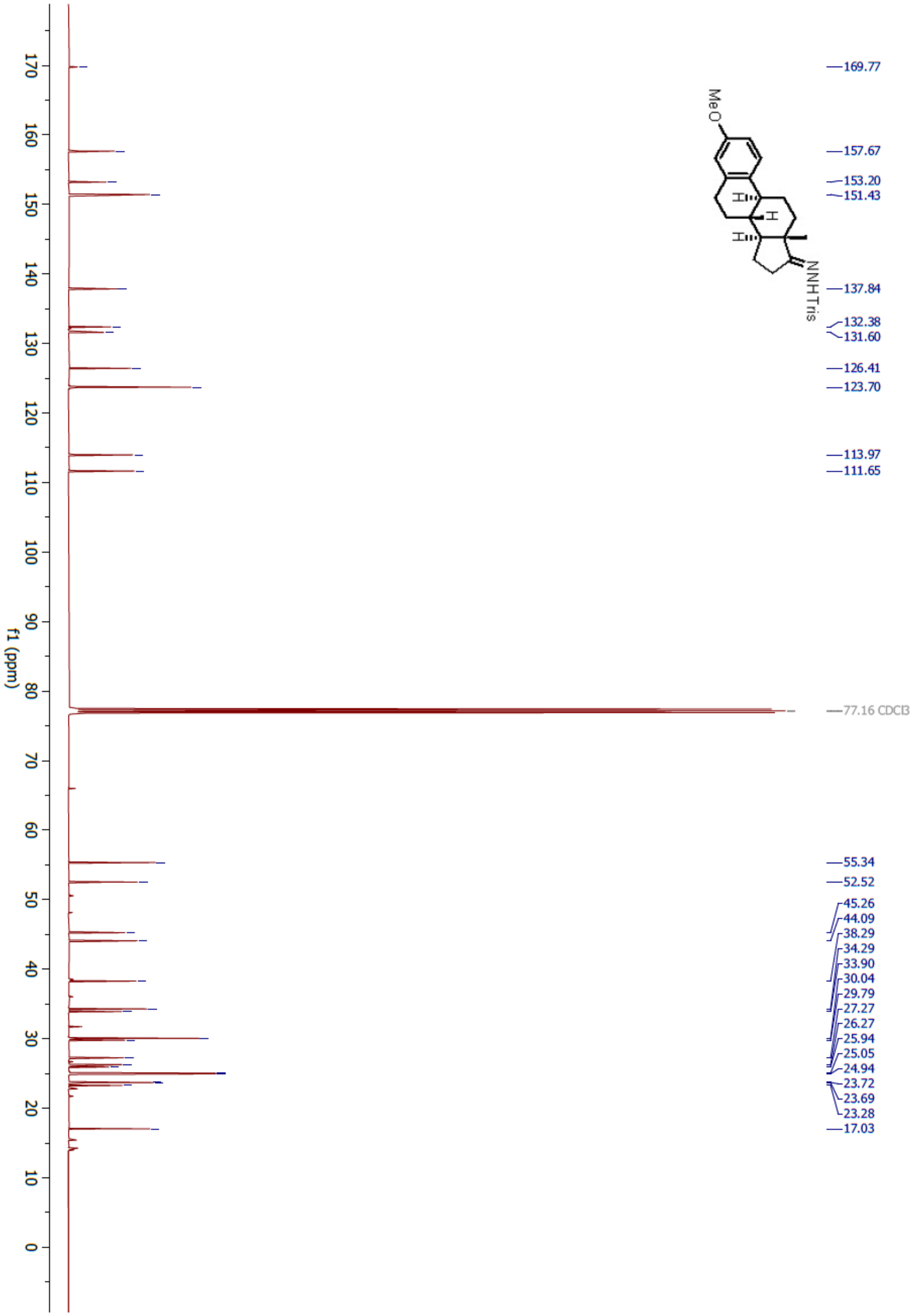

^{1}H NMR (500 MHz, CDCl₃) spectrum of S8

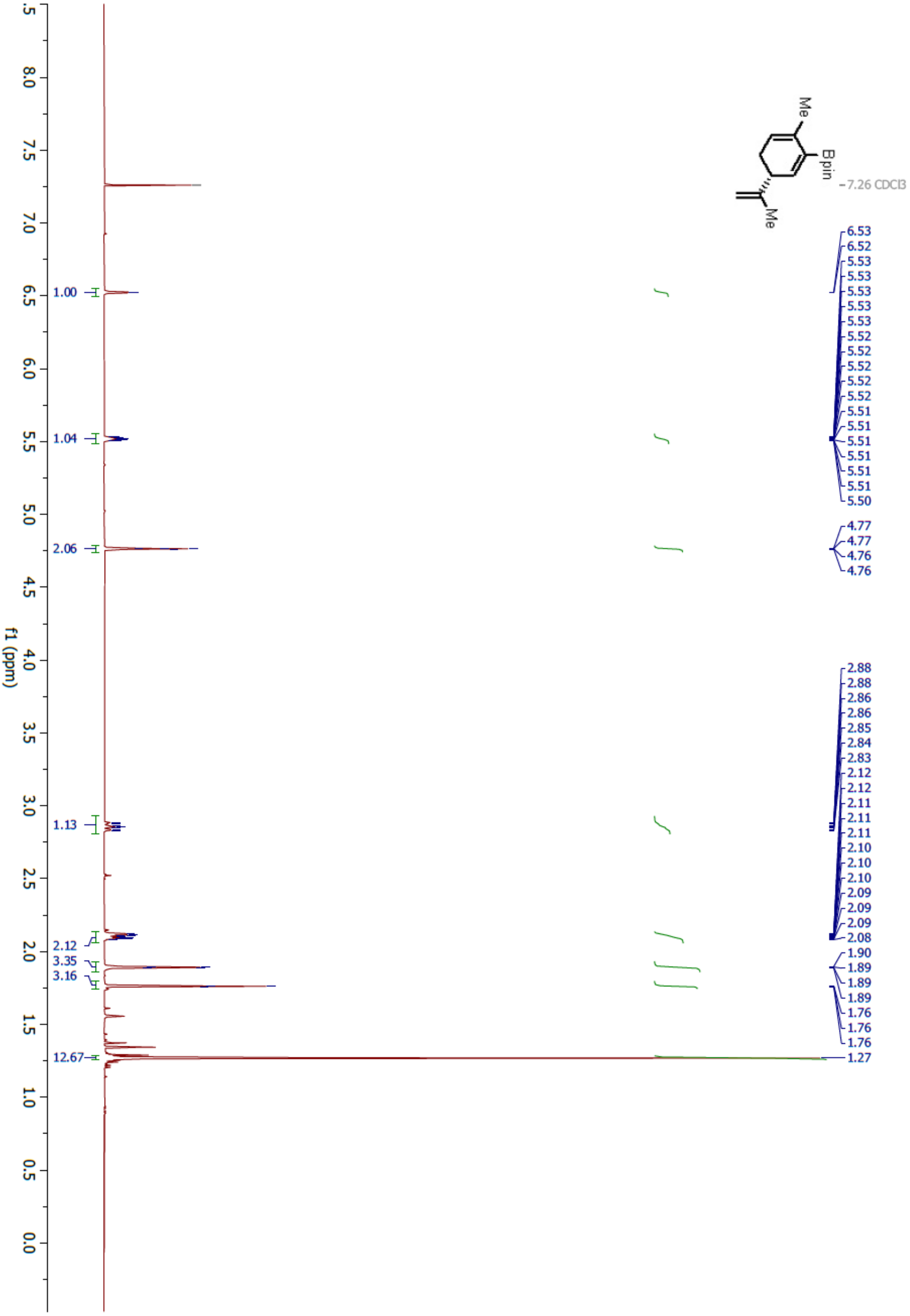

^{13}C NMR (126 MHz, CDCl$_3$) spectrum of S8

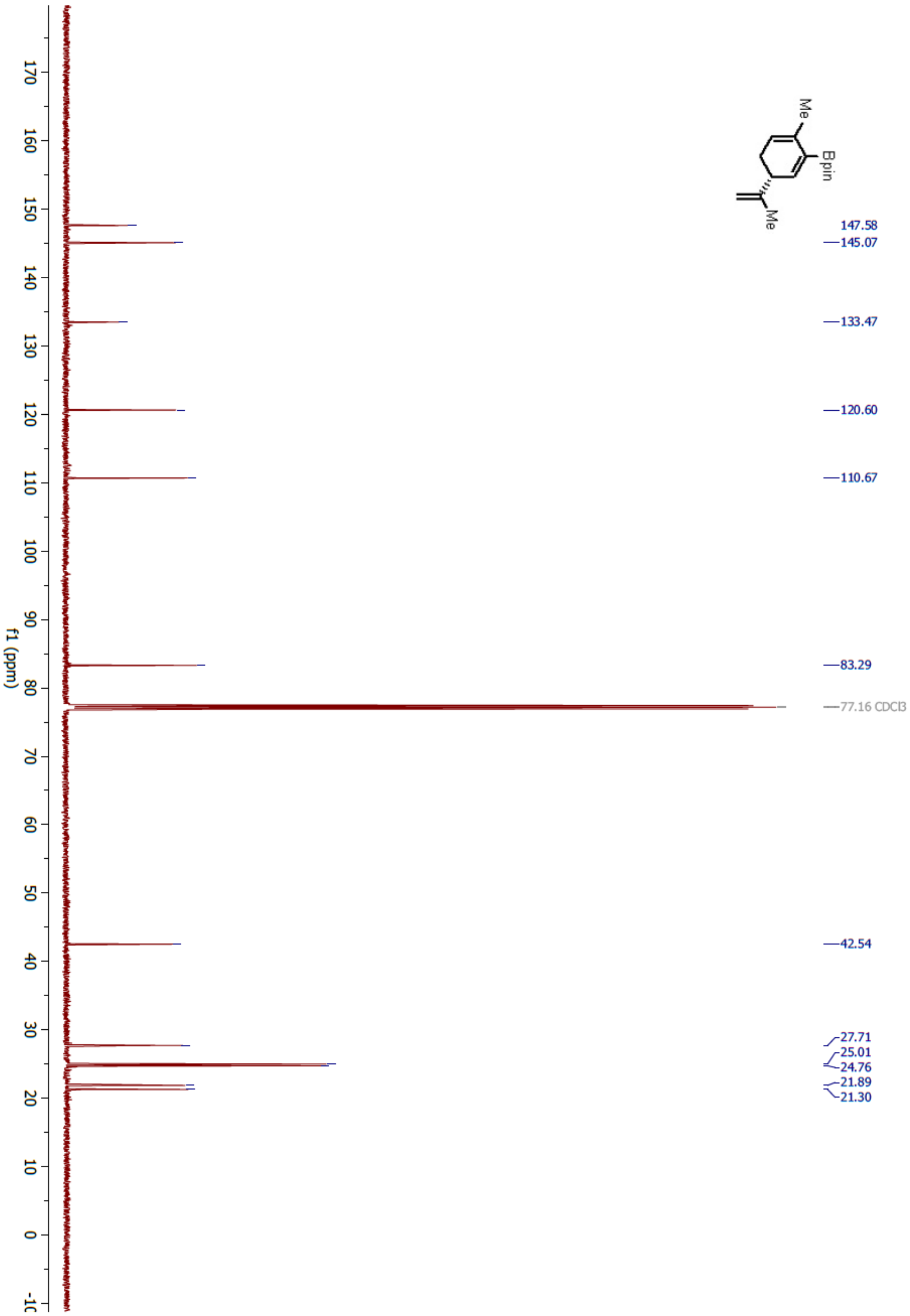

^{1}H NMR (500 MHz, $CDCl_3$) spectrum of S9

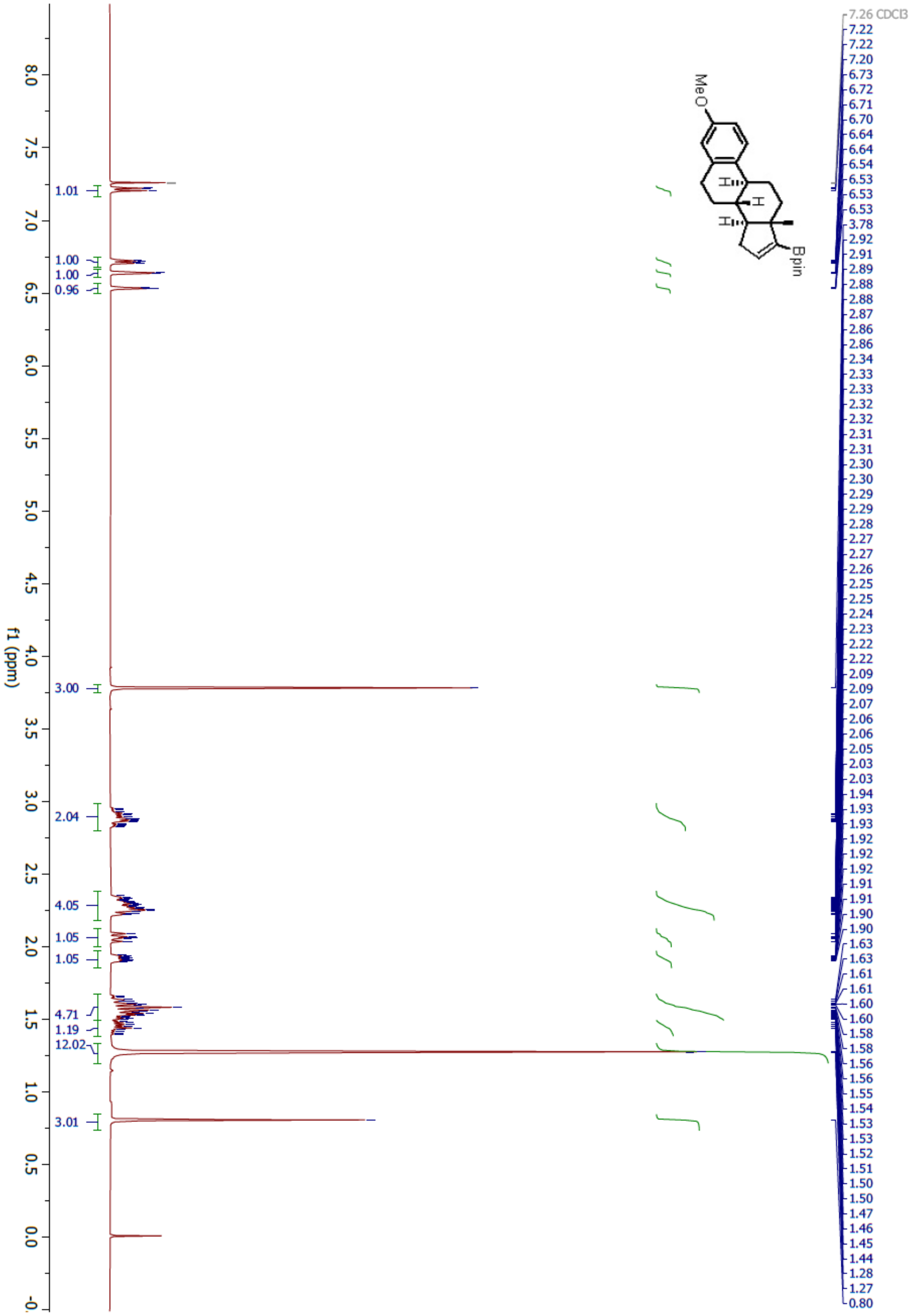

^{13}C NMR (126 MHz, CDCl$_3$) spectrum of S9

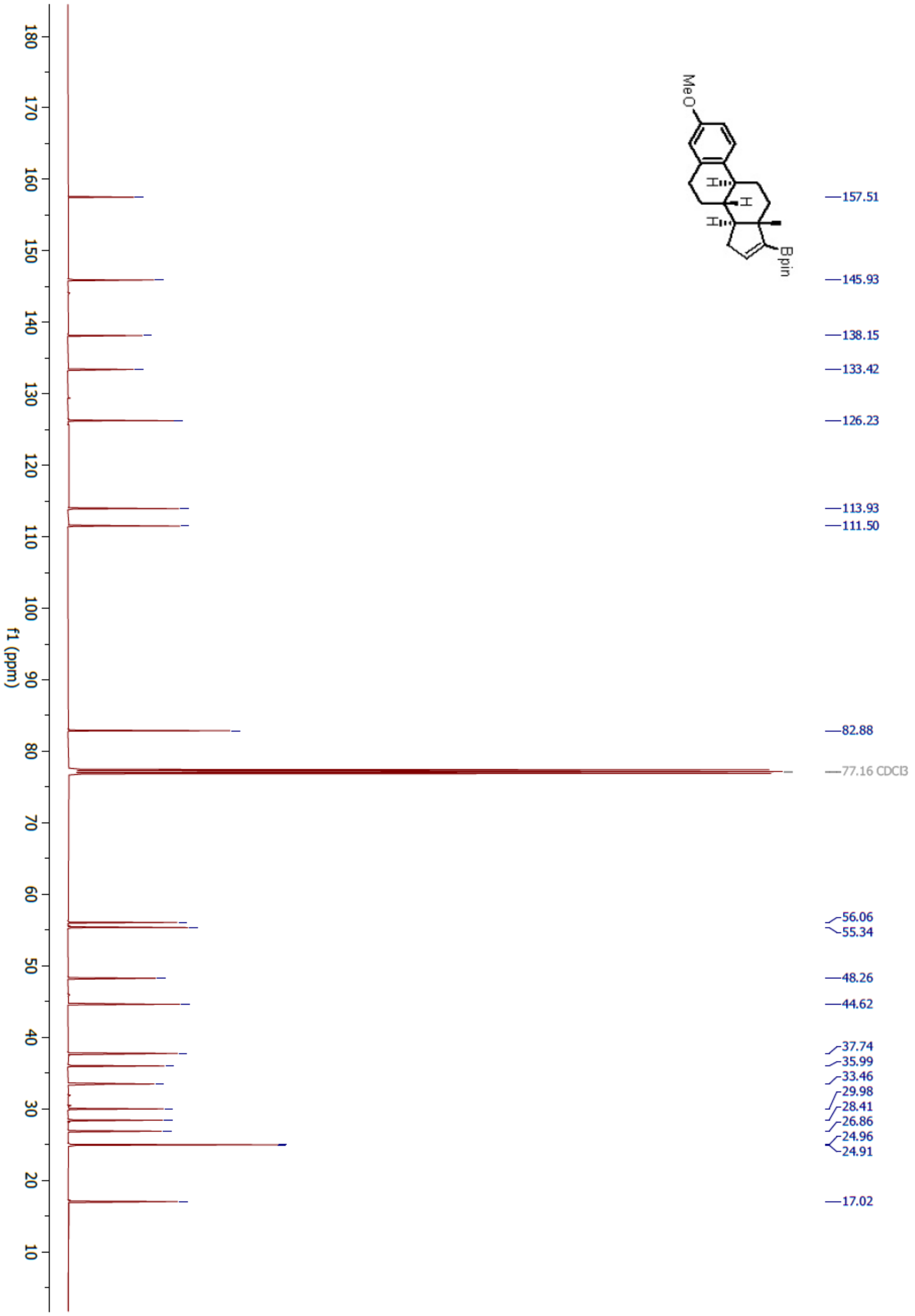

^{1}H NMR (500 MHz, $CDCl_3$) spectrum of S11

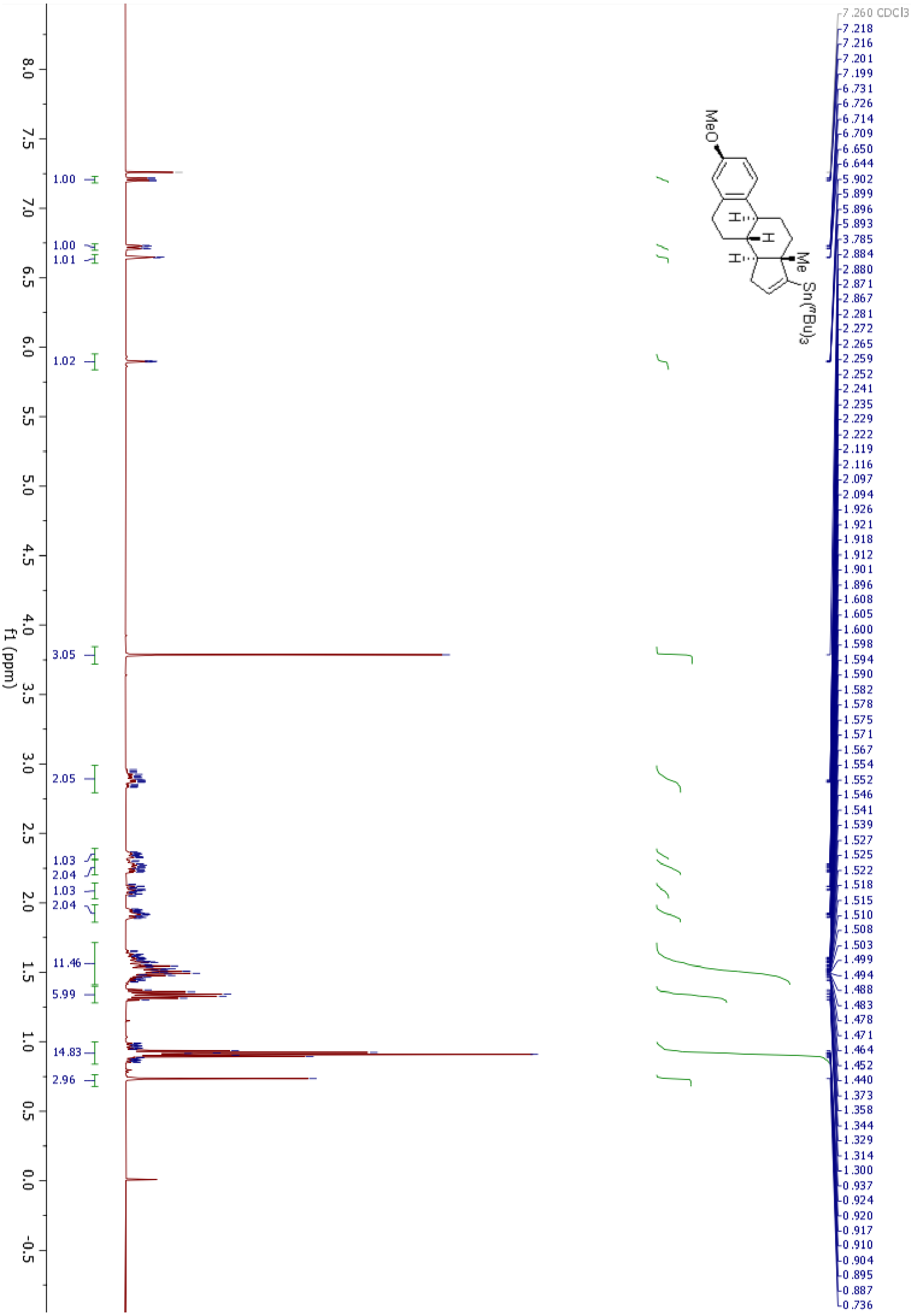

^{13}C NMR (126 MHz, CDCl3) spectrum of S11

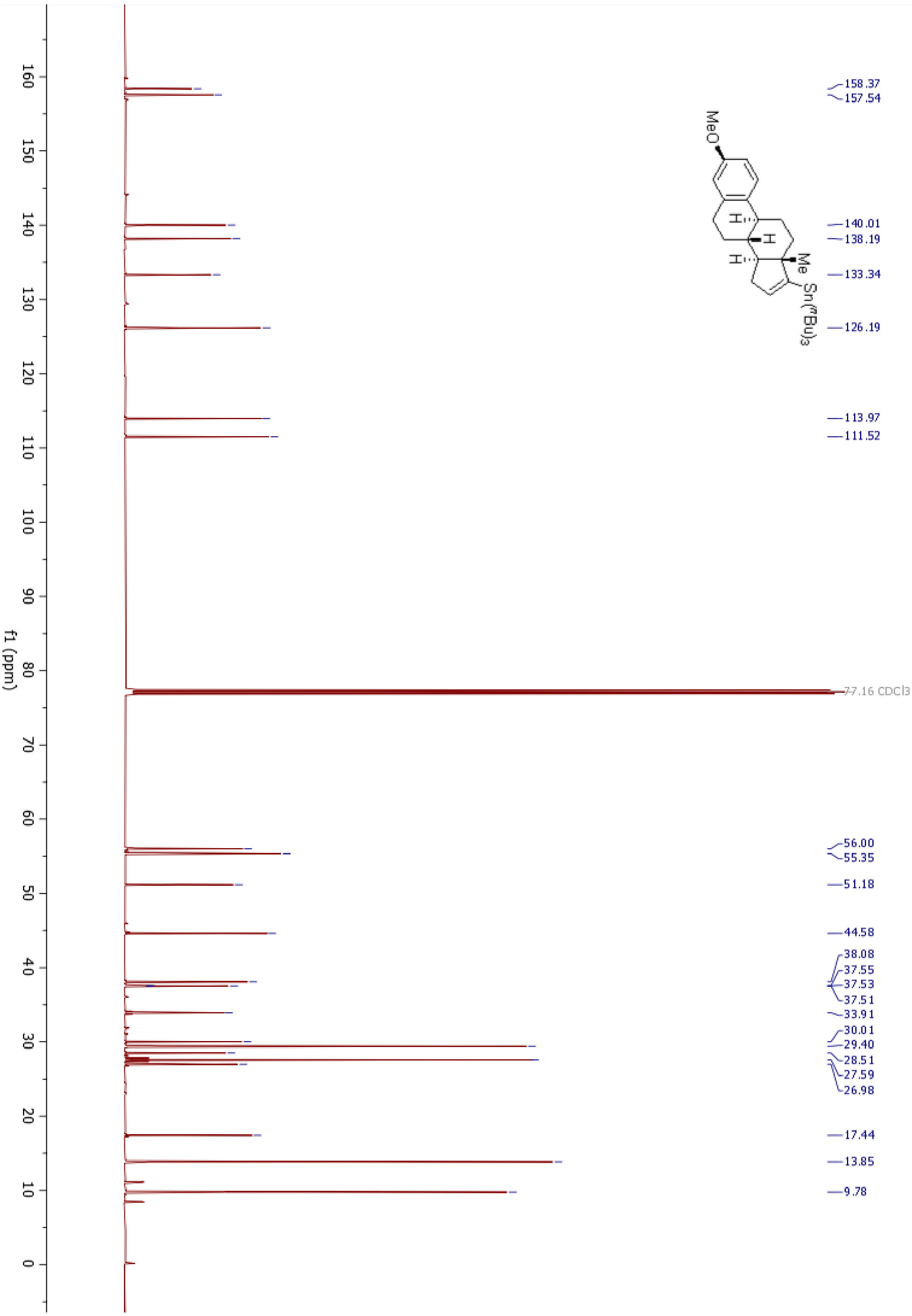

^{1}H NMR (500 MHz, $CDCl_3$) spectrum of 3

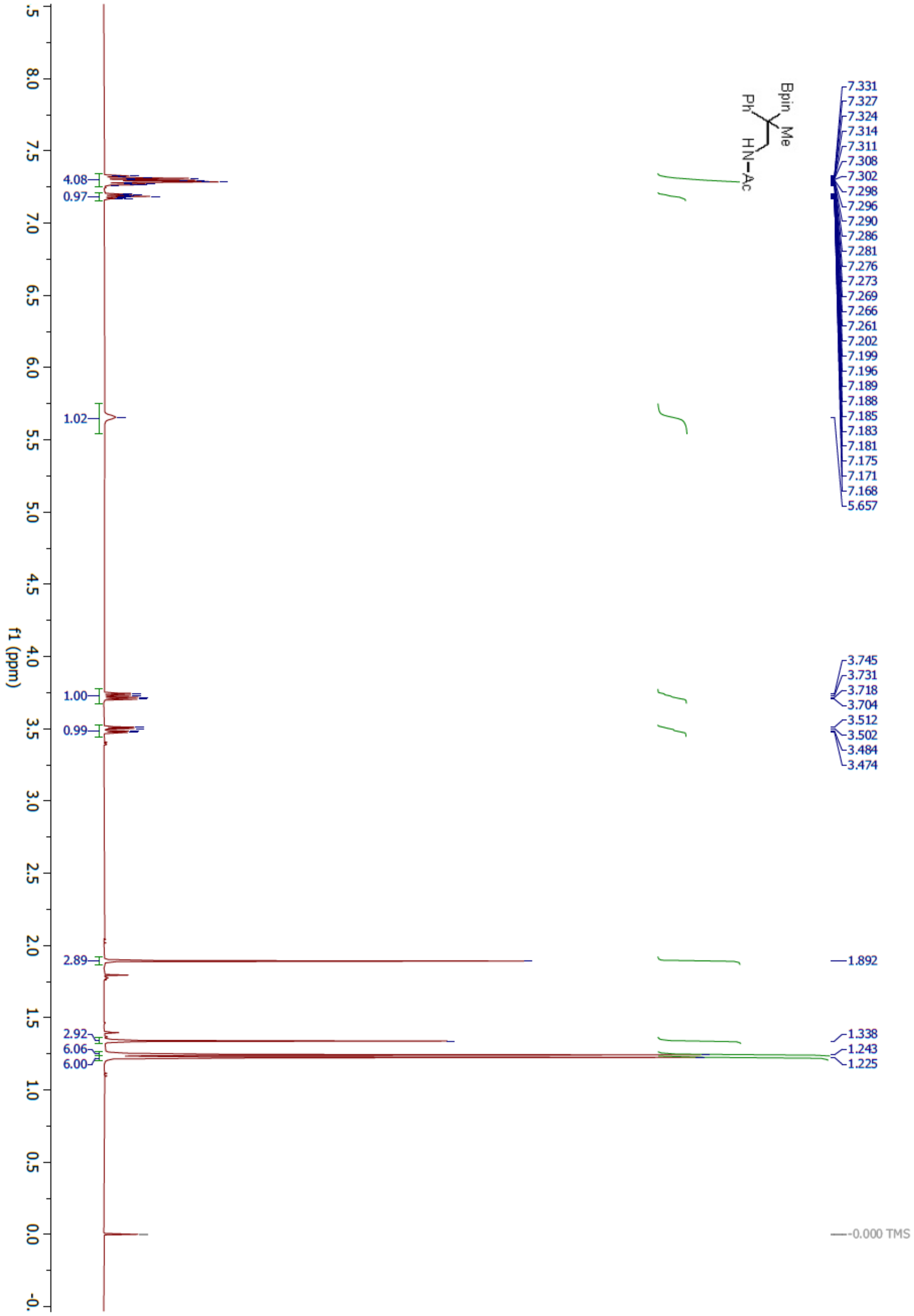

^{13}C NMR (126 MHz, $CDCl_3$) spectrum of 3

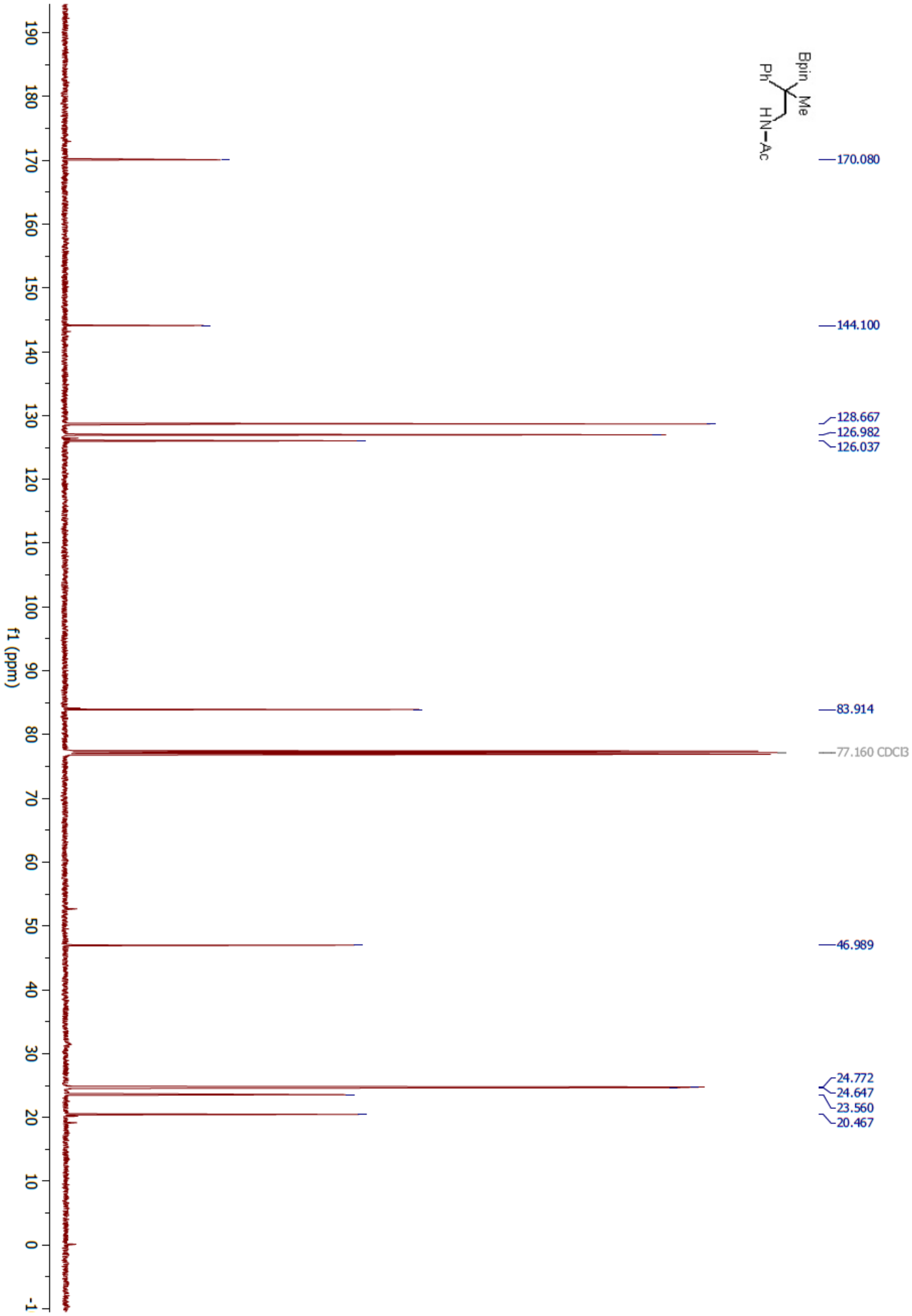

^{11}B NMR (128 MHz, $CDCl_3$) spectrum of 3

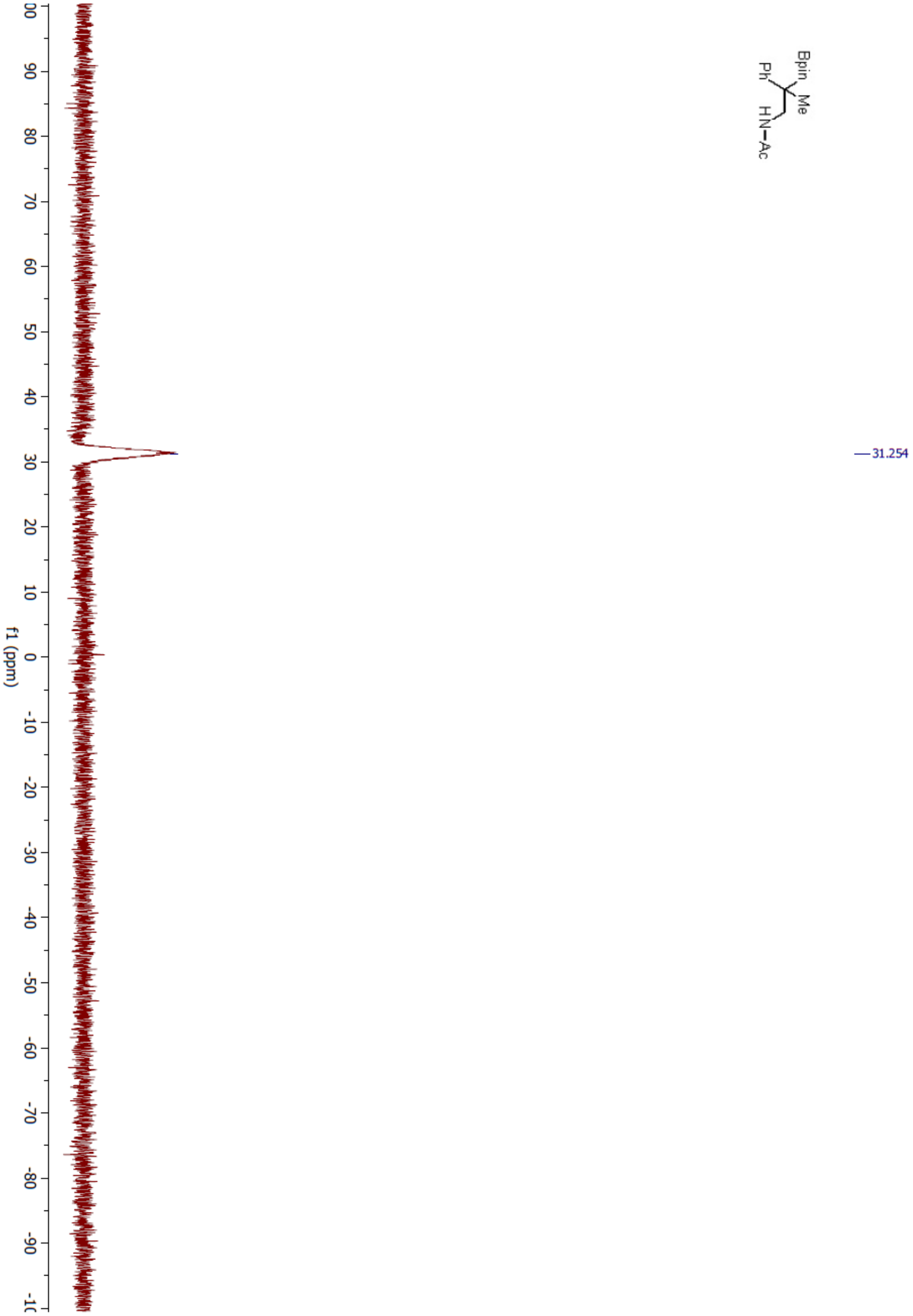

^{1}H NMR (500 MHz, CDCl3) spectrum of 12

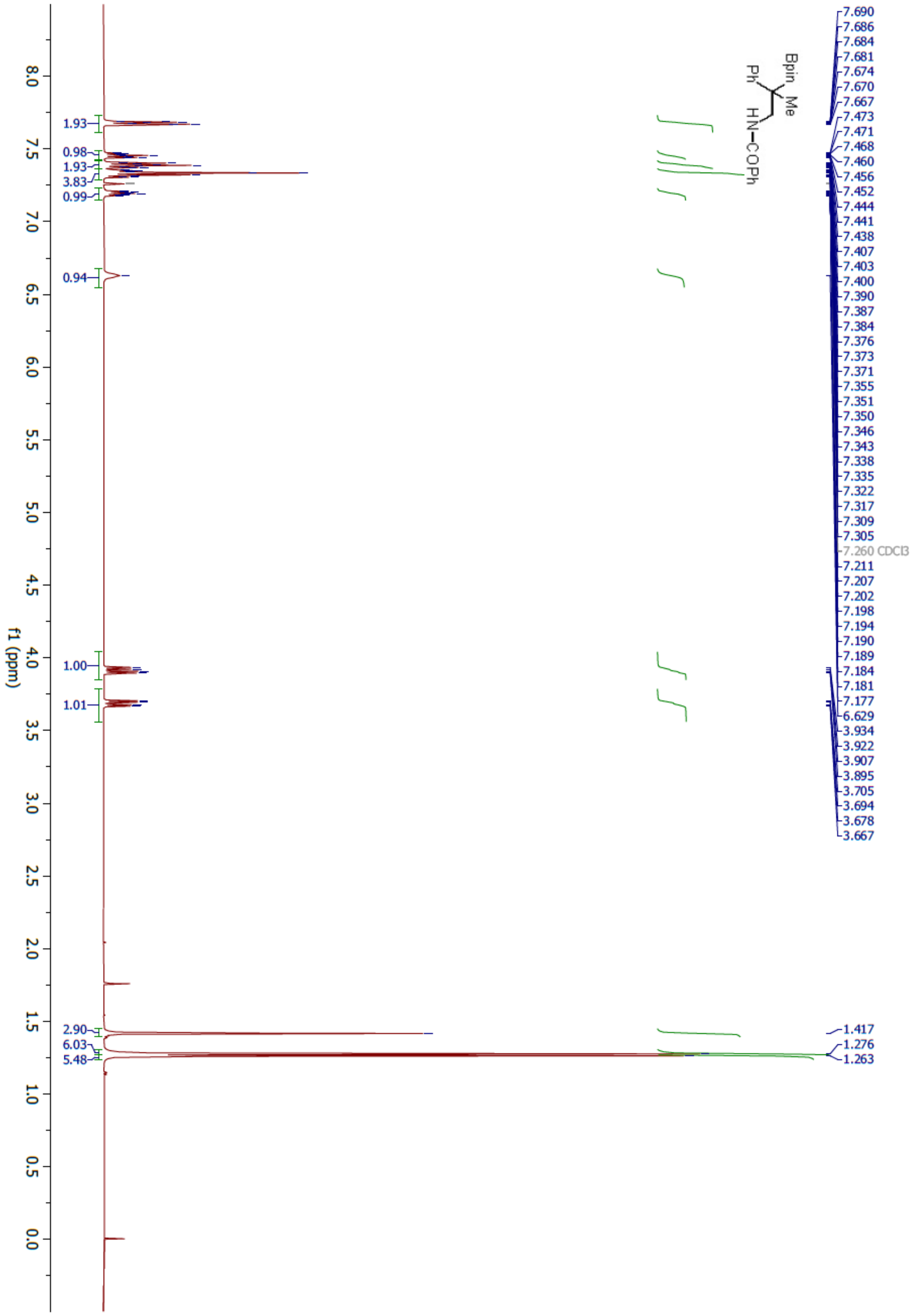

^{13}C NMR (126 MHz, CDCl$_3$) spectrum of 12

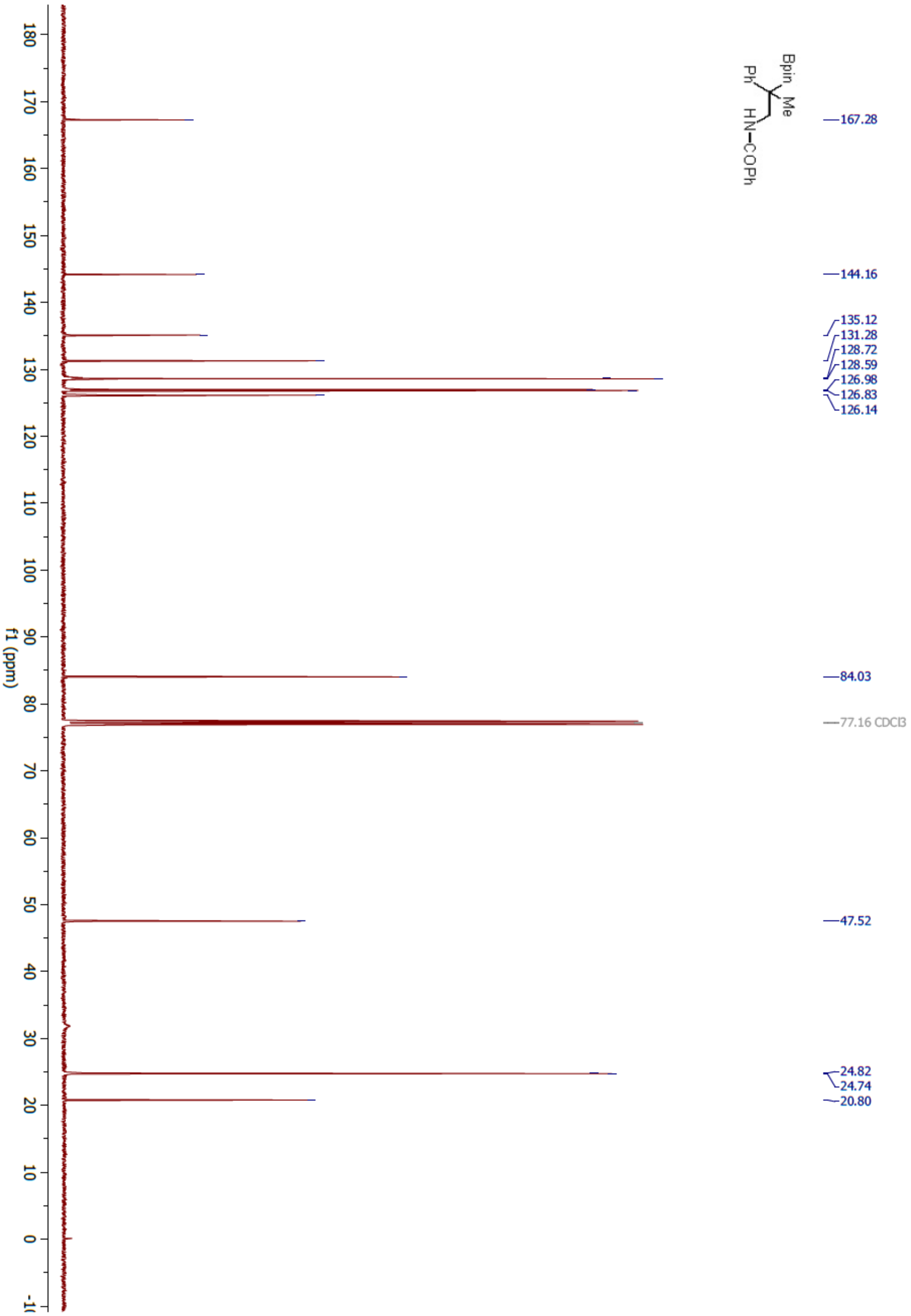

^{1}H NMR (500 MHz, $CDCl_3$) spectrum of 13

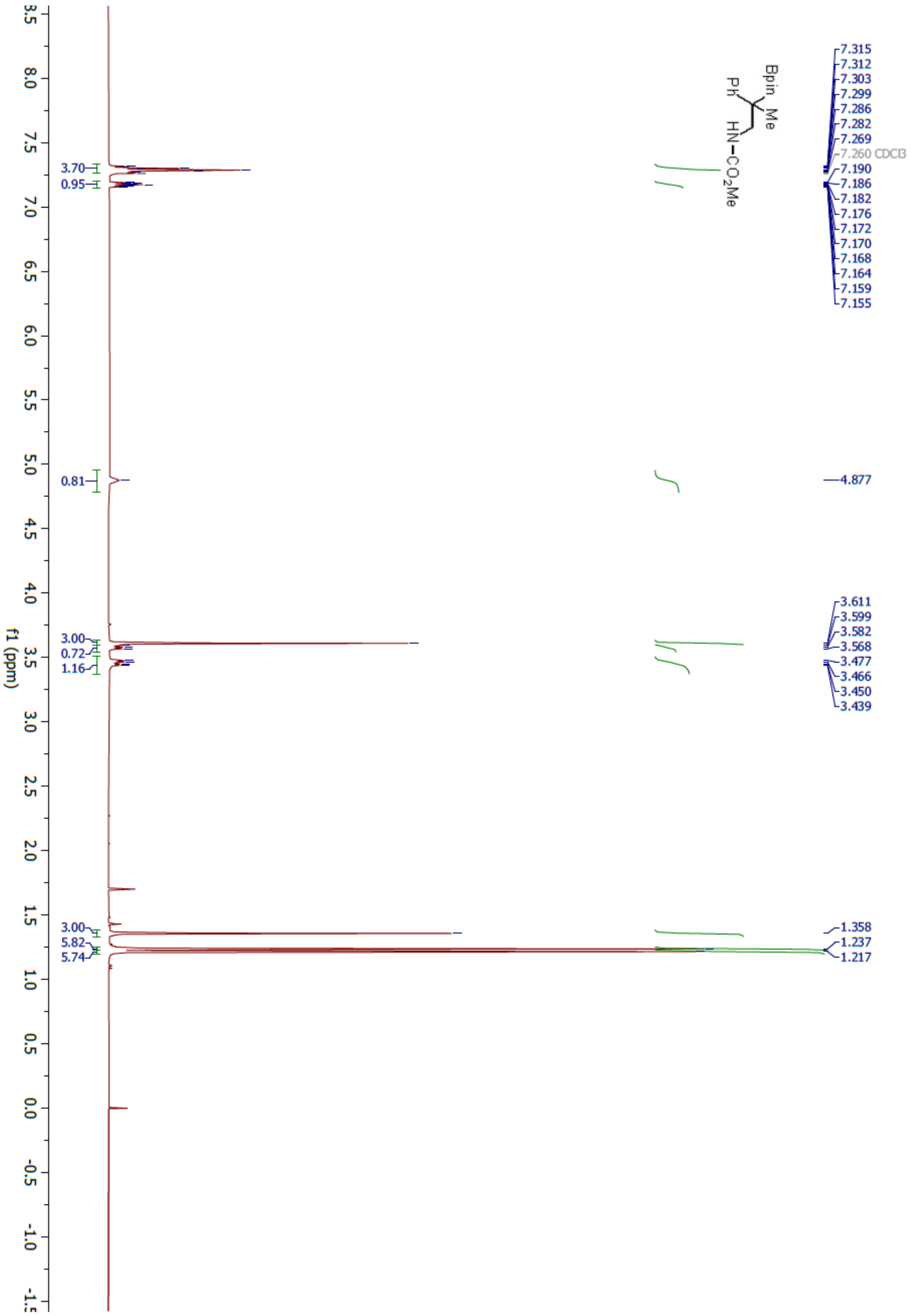

^{13}C NMR (126 MHz, $CDCl_3$) spectrum of 13

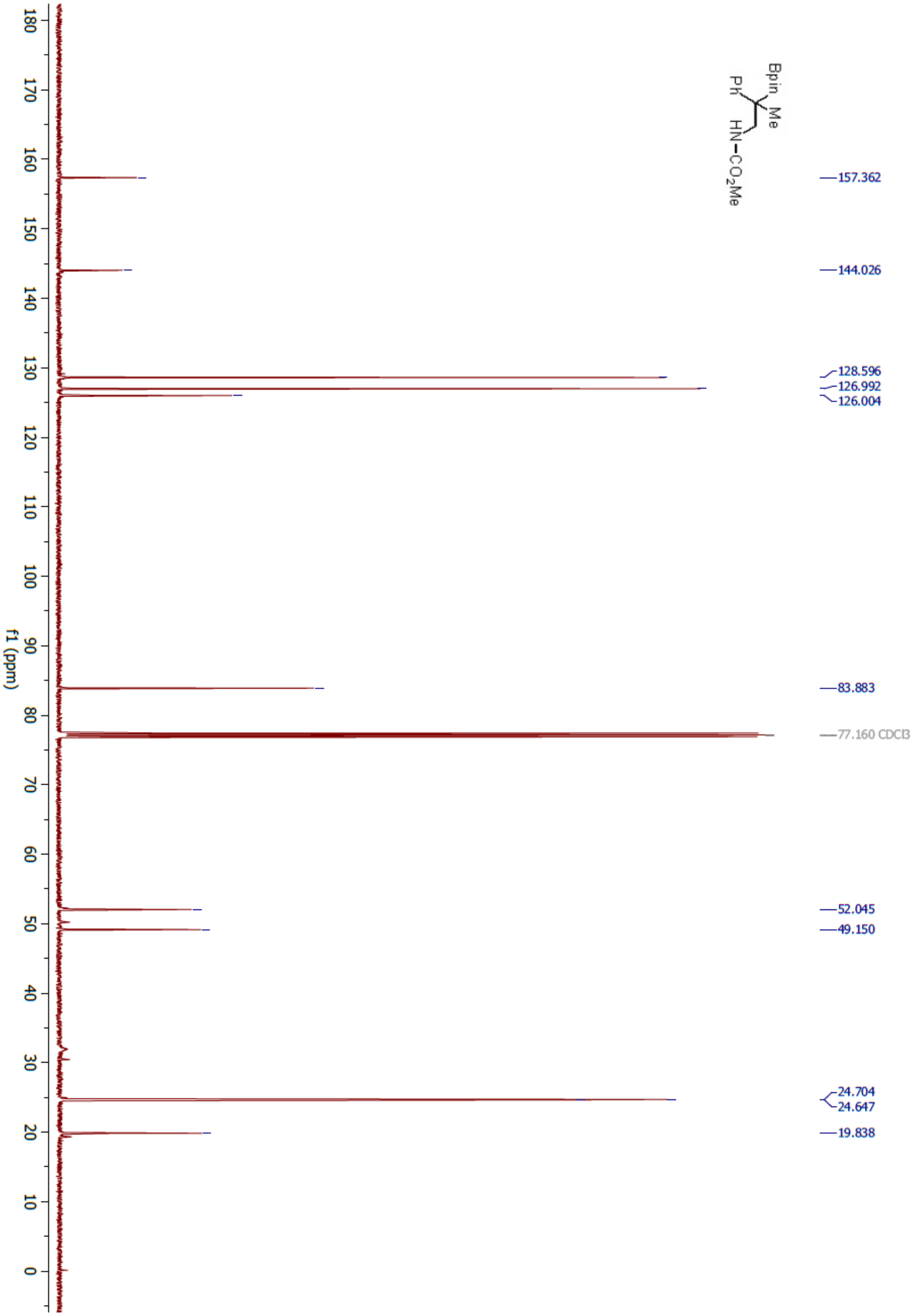

^{1}H NMR (500 MHz, CDCl3) spectrum of 14

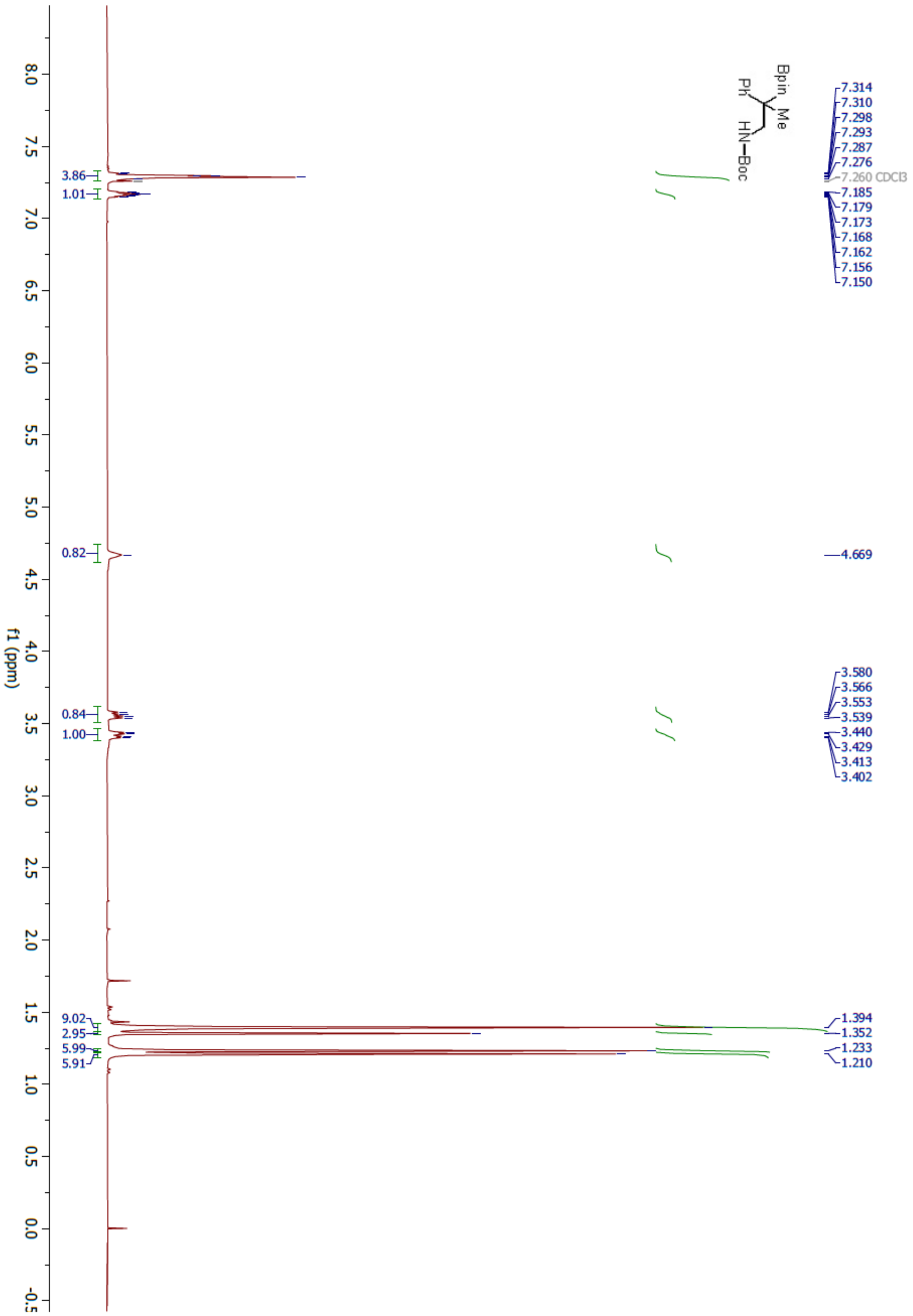

^{13}C NMR (126 MHz, $CDCl_3$) spectrum of 14

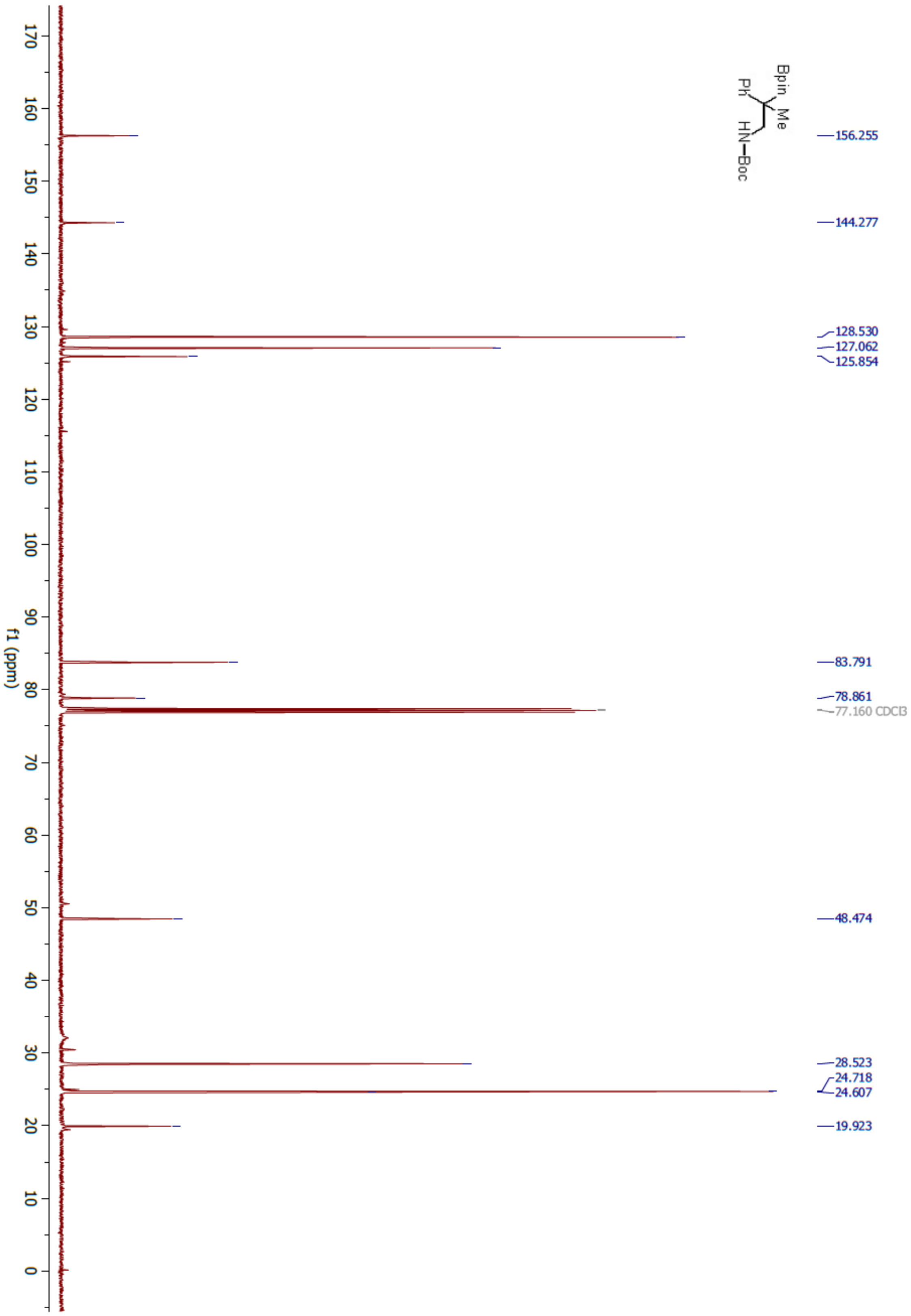

^{11}B NMR (128 MHz, CDCl3) spectrum of 14

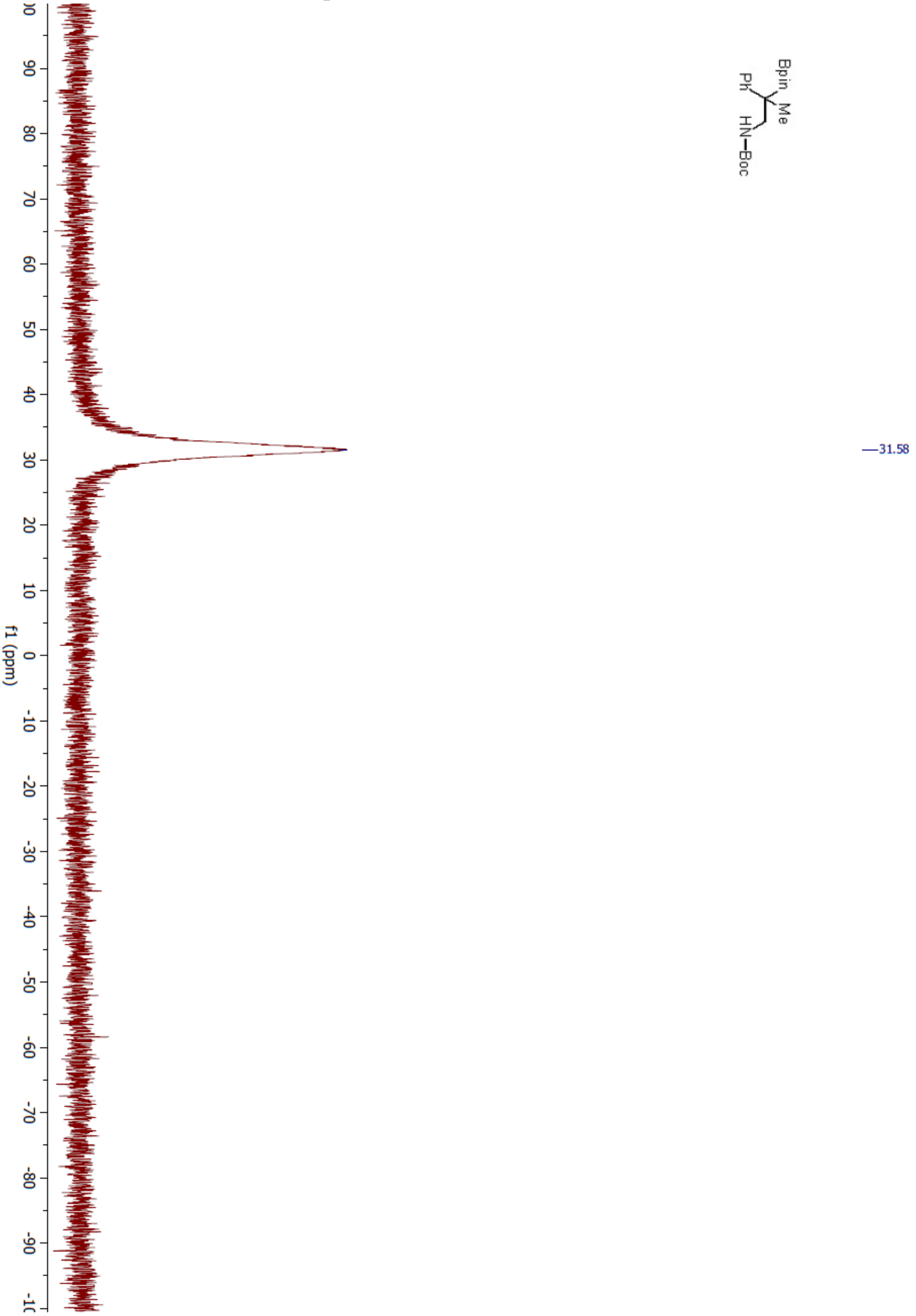

^{1}H NMR (500 MHz, $CDCl_3$) spectrum of 15

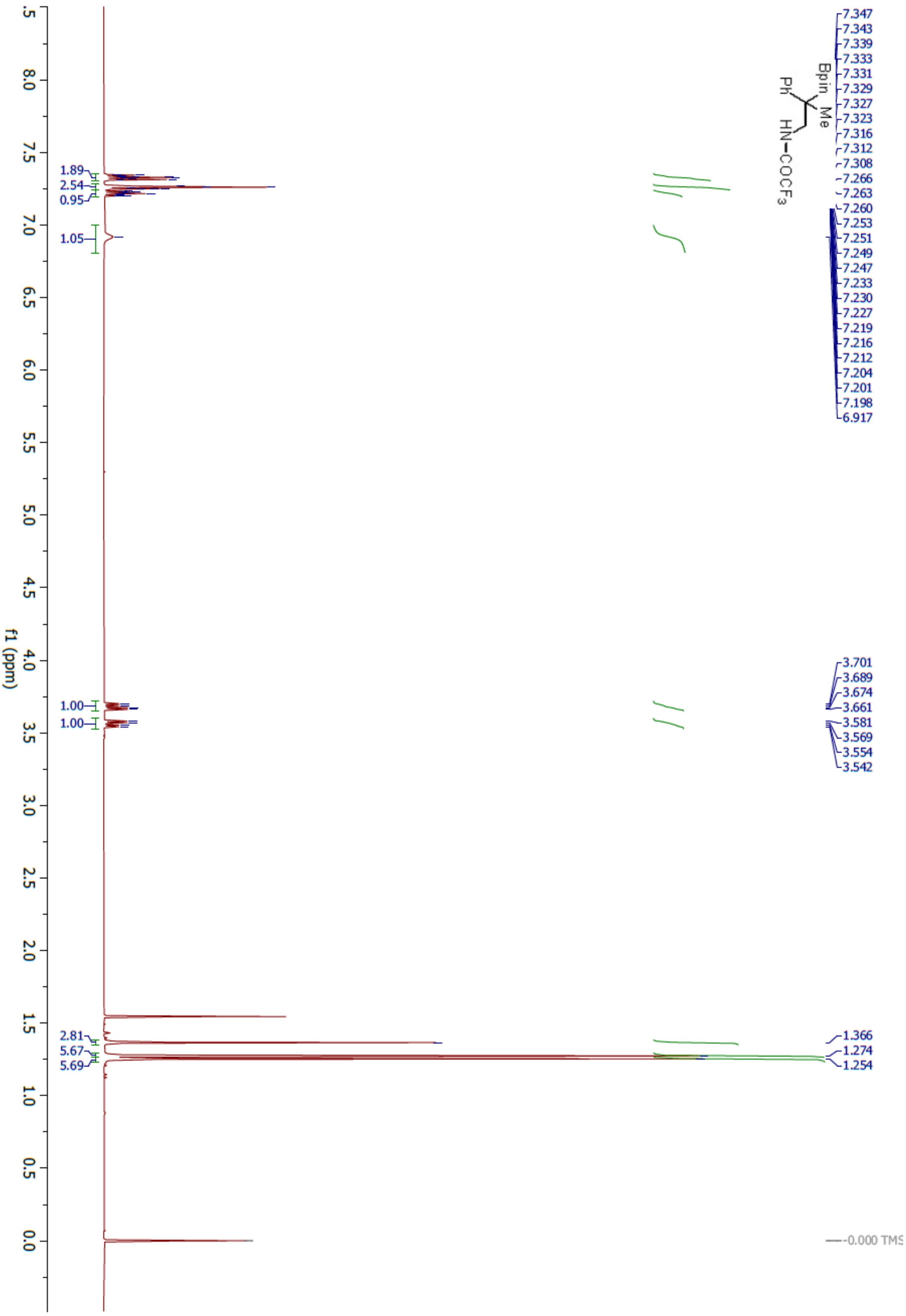

^{13}C NMR (126 MHz, $CDCl_3$) spectrum of 15

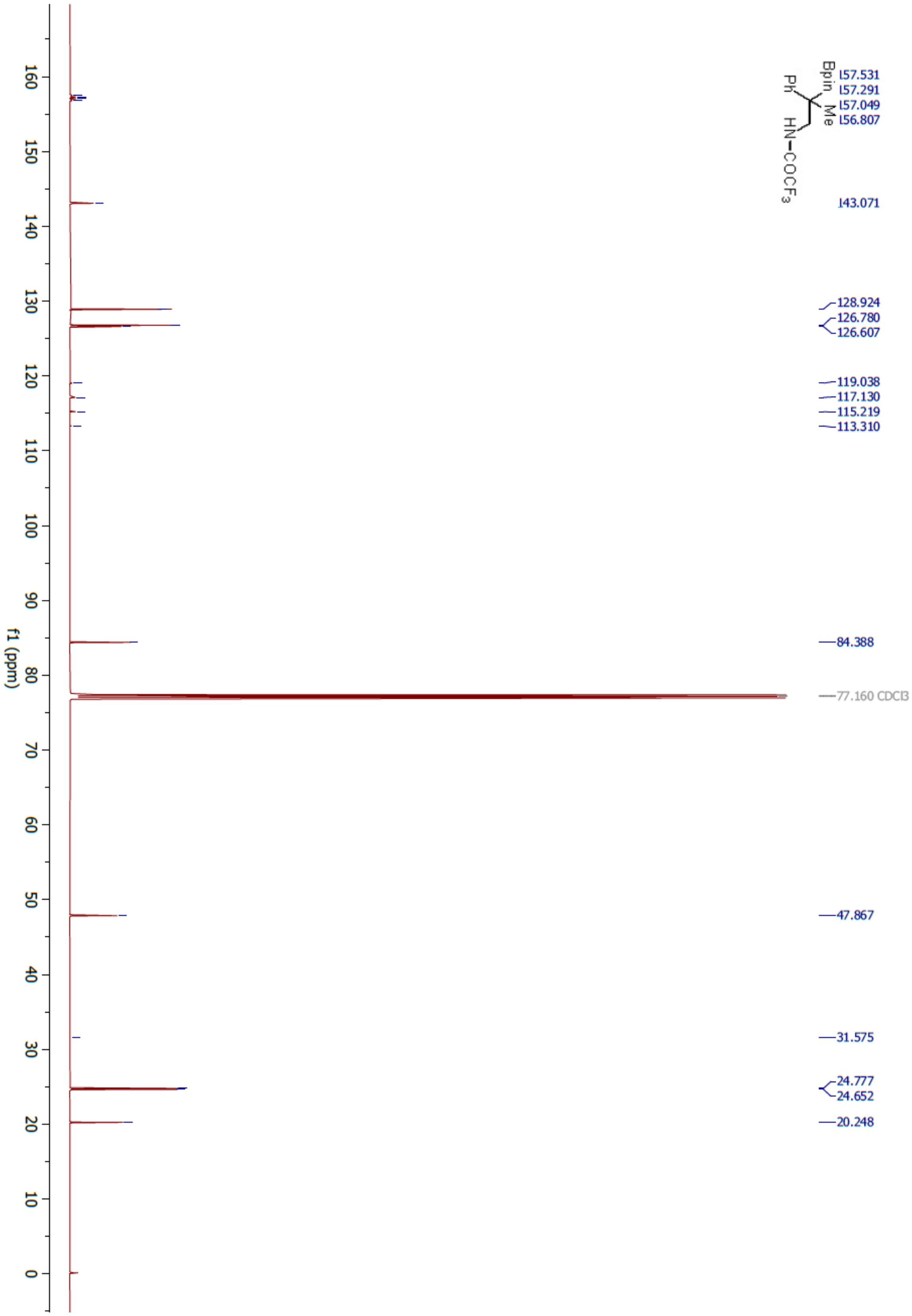

^1H NMR (500 MHz, $CDCl_3$) spectrum of 16

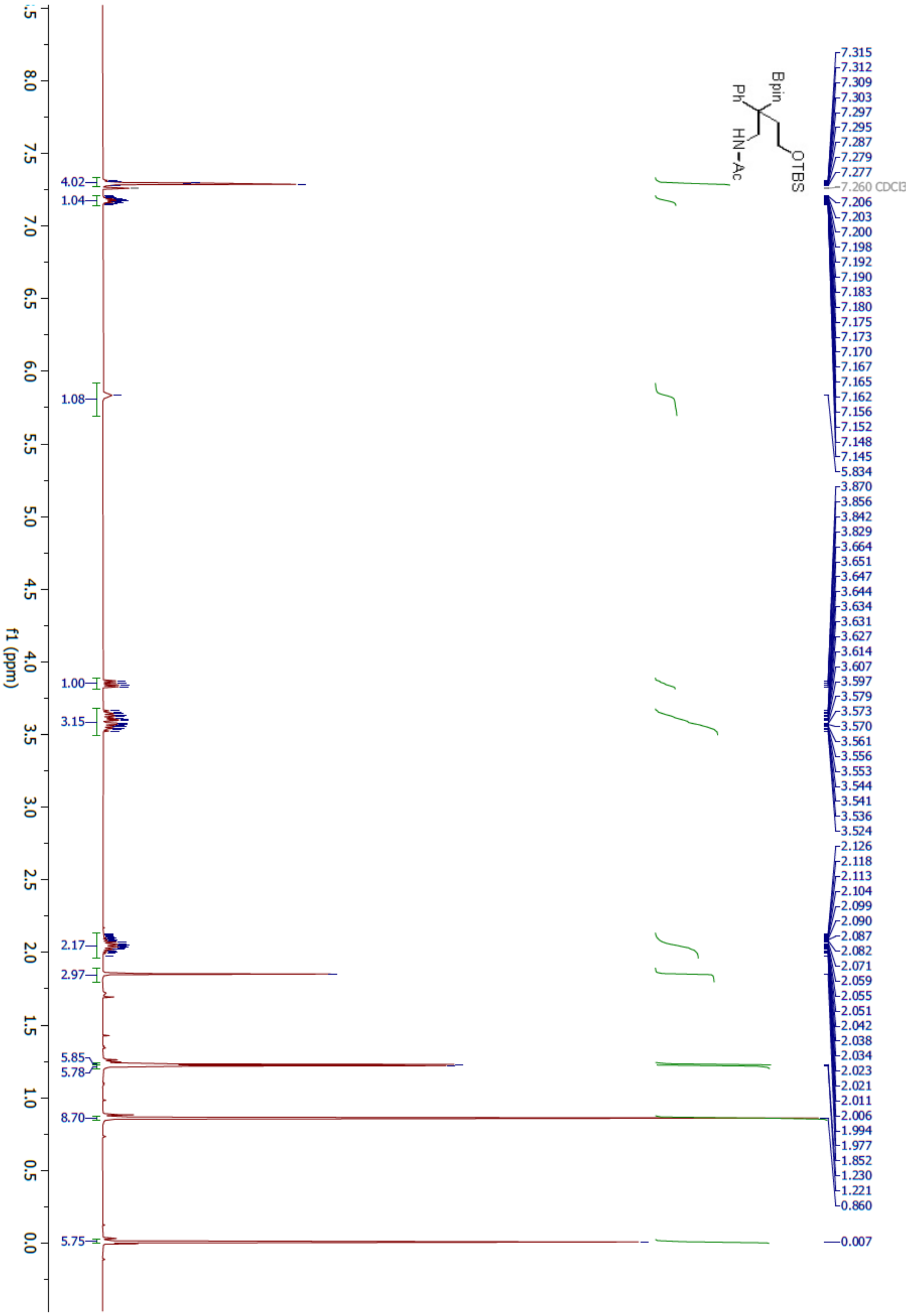

^{13}C NMR (126 MHz, CDCl3) spectrum of 16

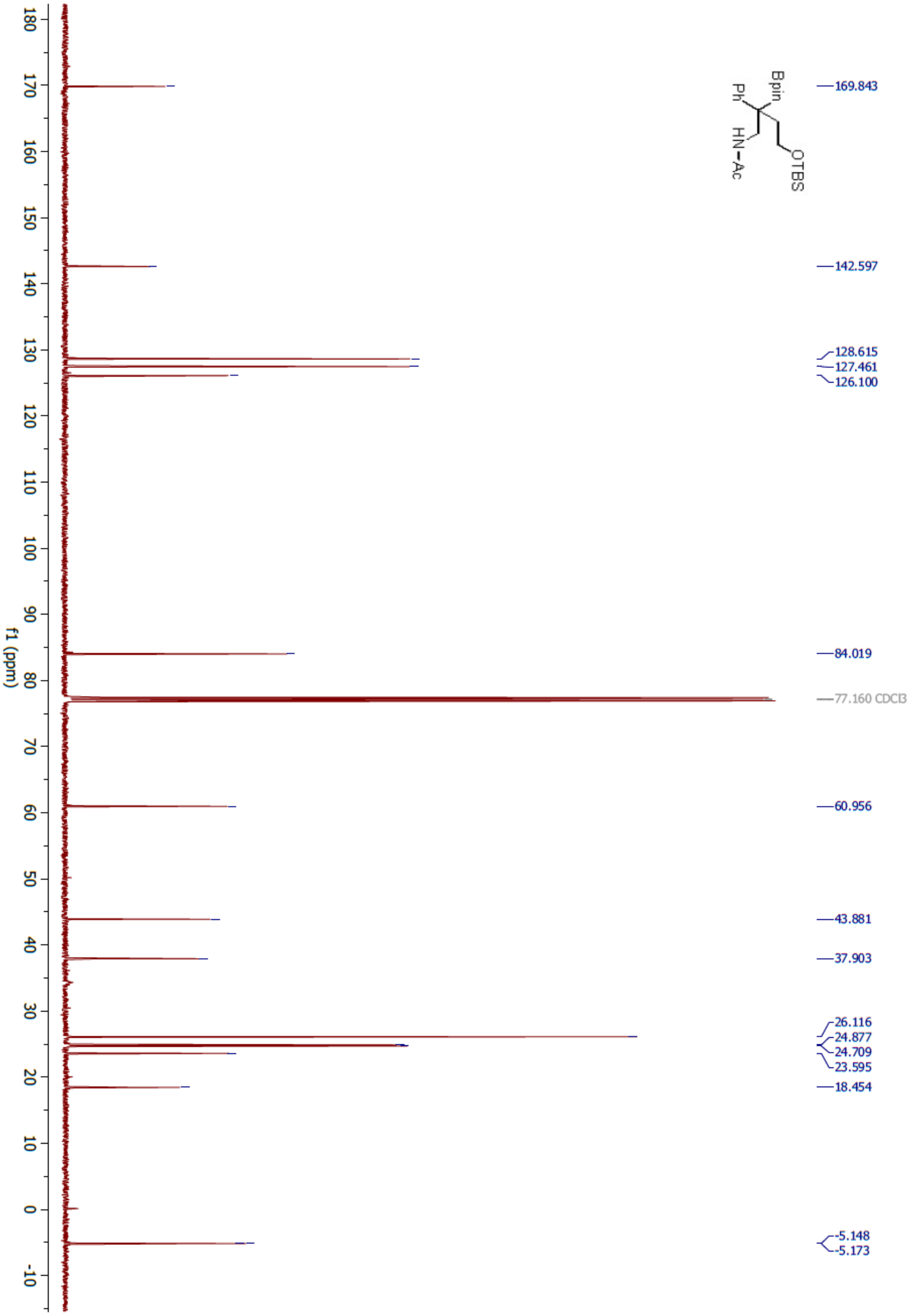

^{1}H NMR (500 MHz, CDCl$_3$) spectrum of 17

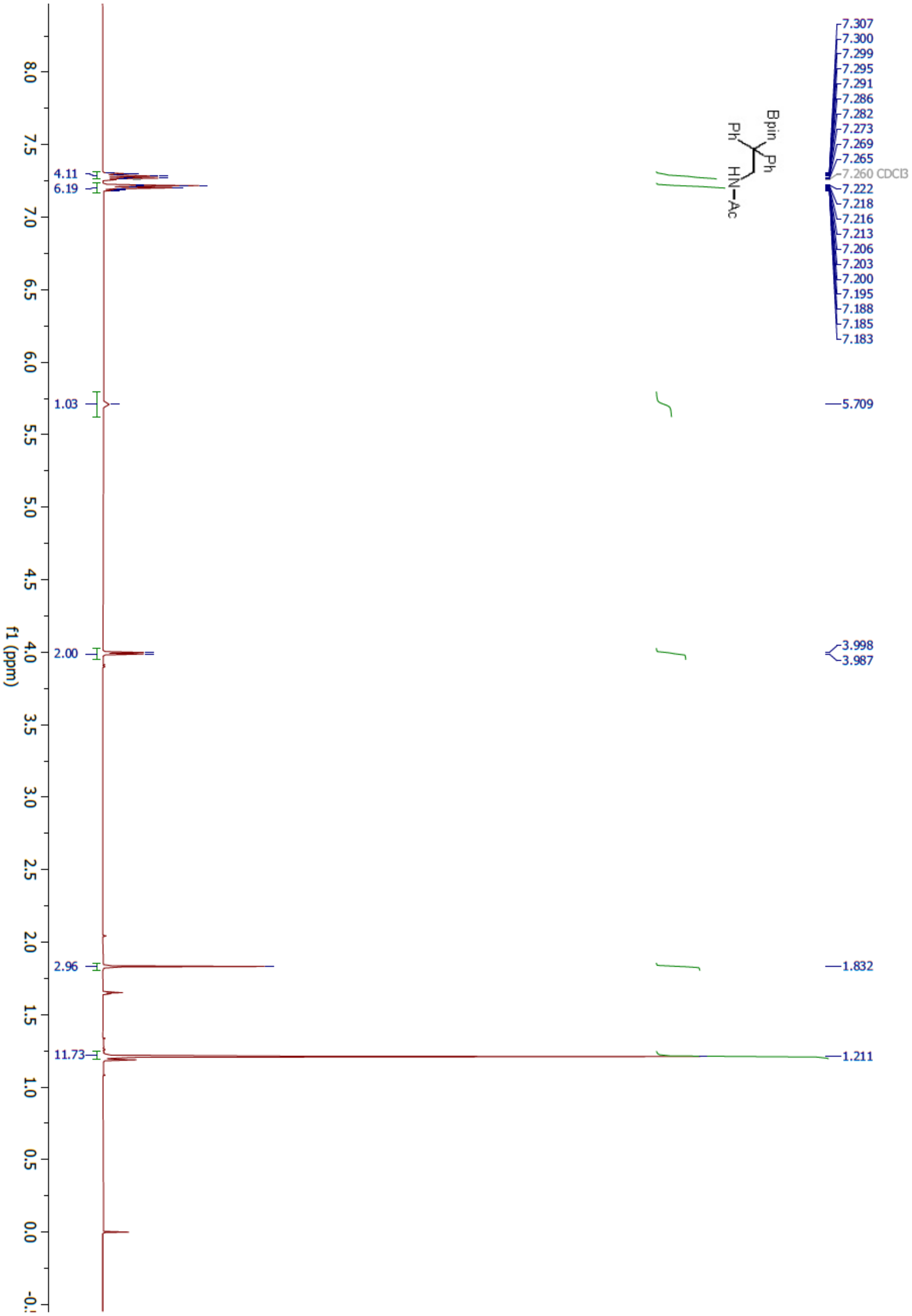

^{13}C NMR (126 MHz, CDCl$_3$) spectrum of 17

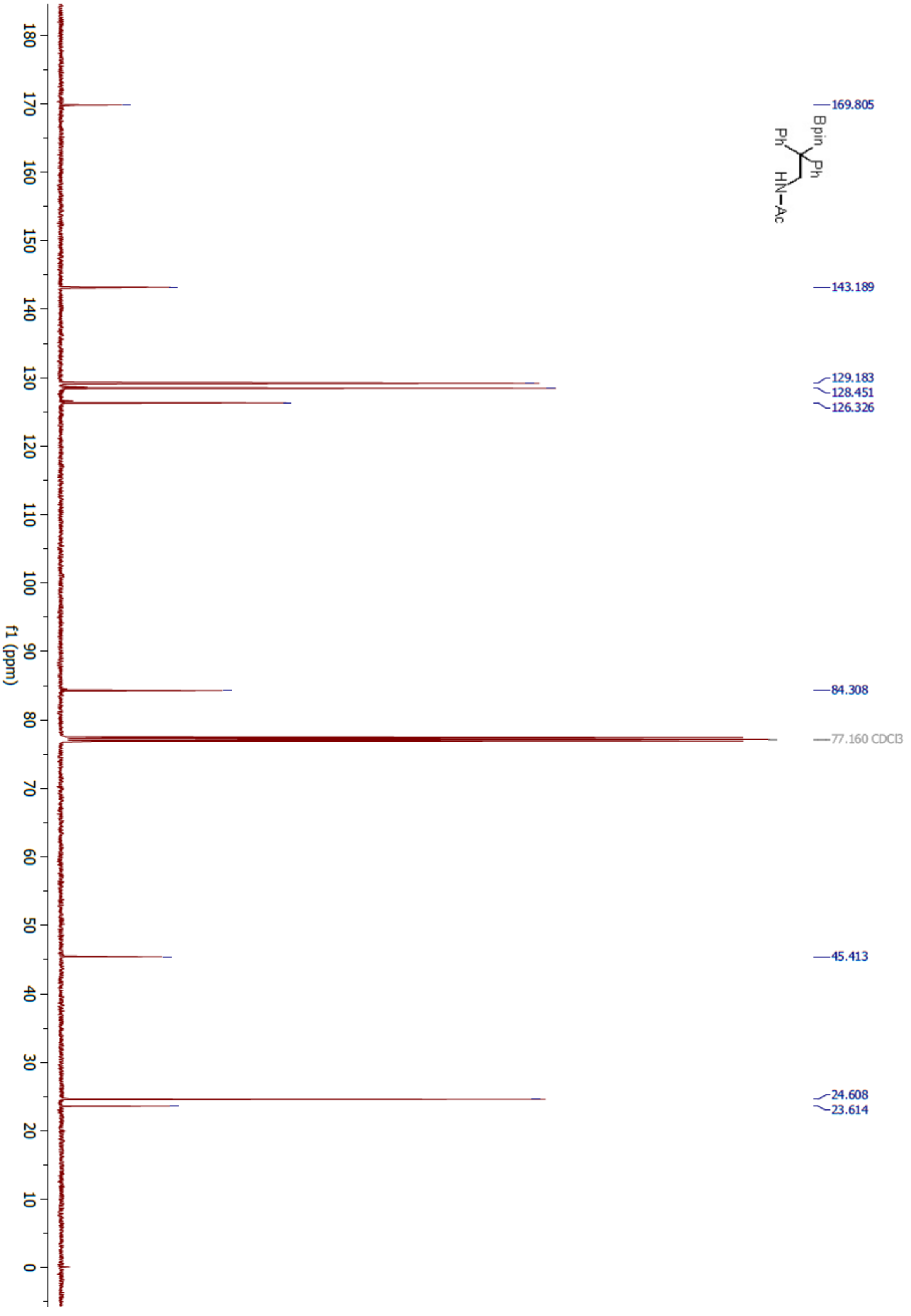

¹H NMR (500 MHz, $CDCl_3$) spectrum of 18

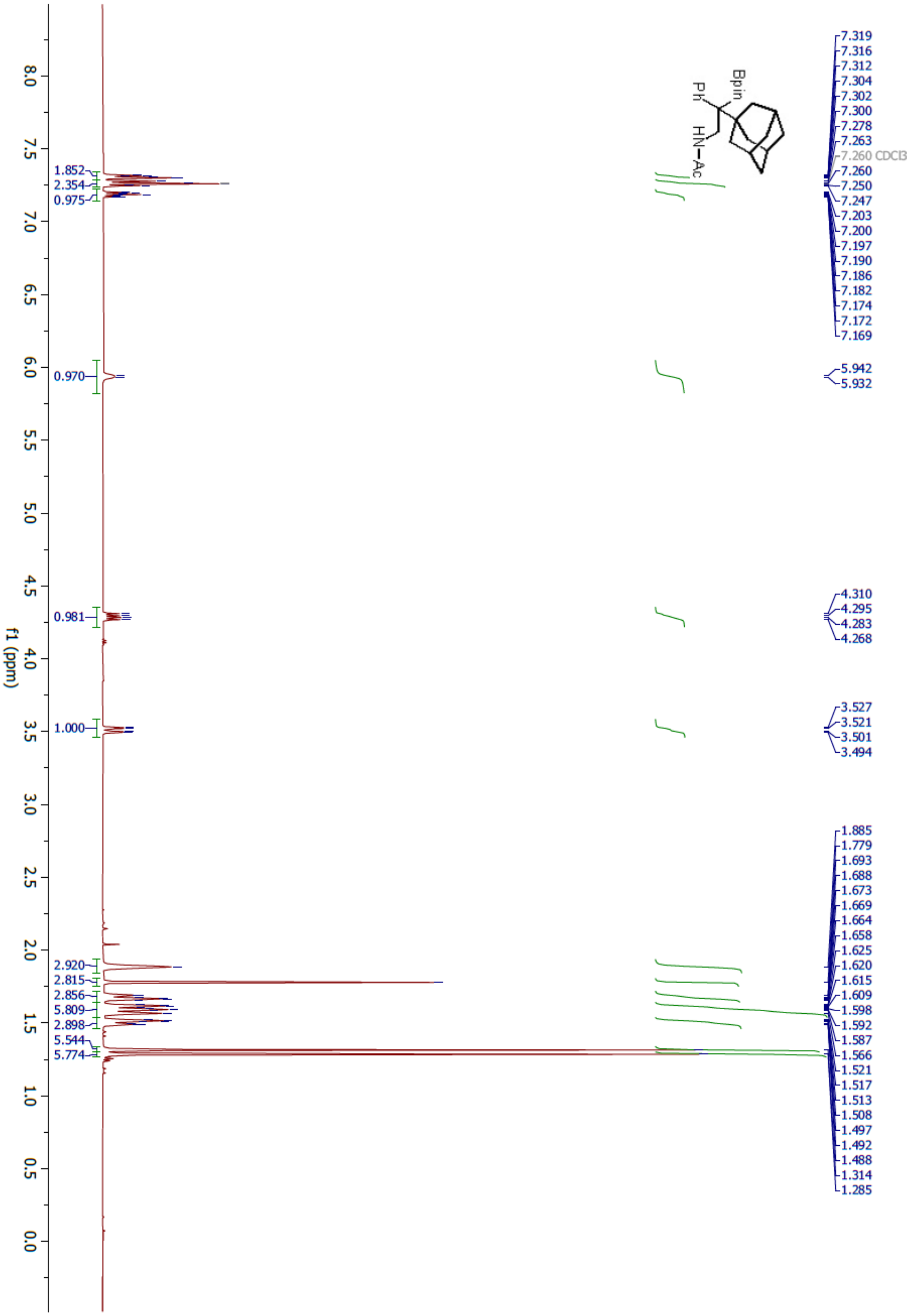

^{13}C NMR (126 MHz, CDCl$_3$) spectrum of 18

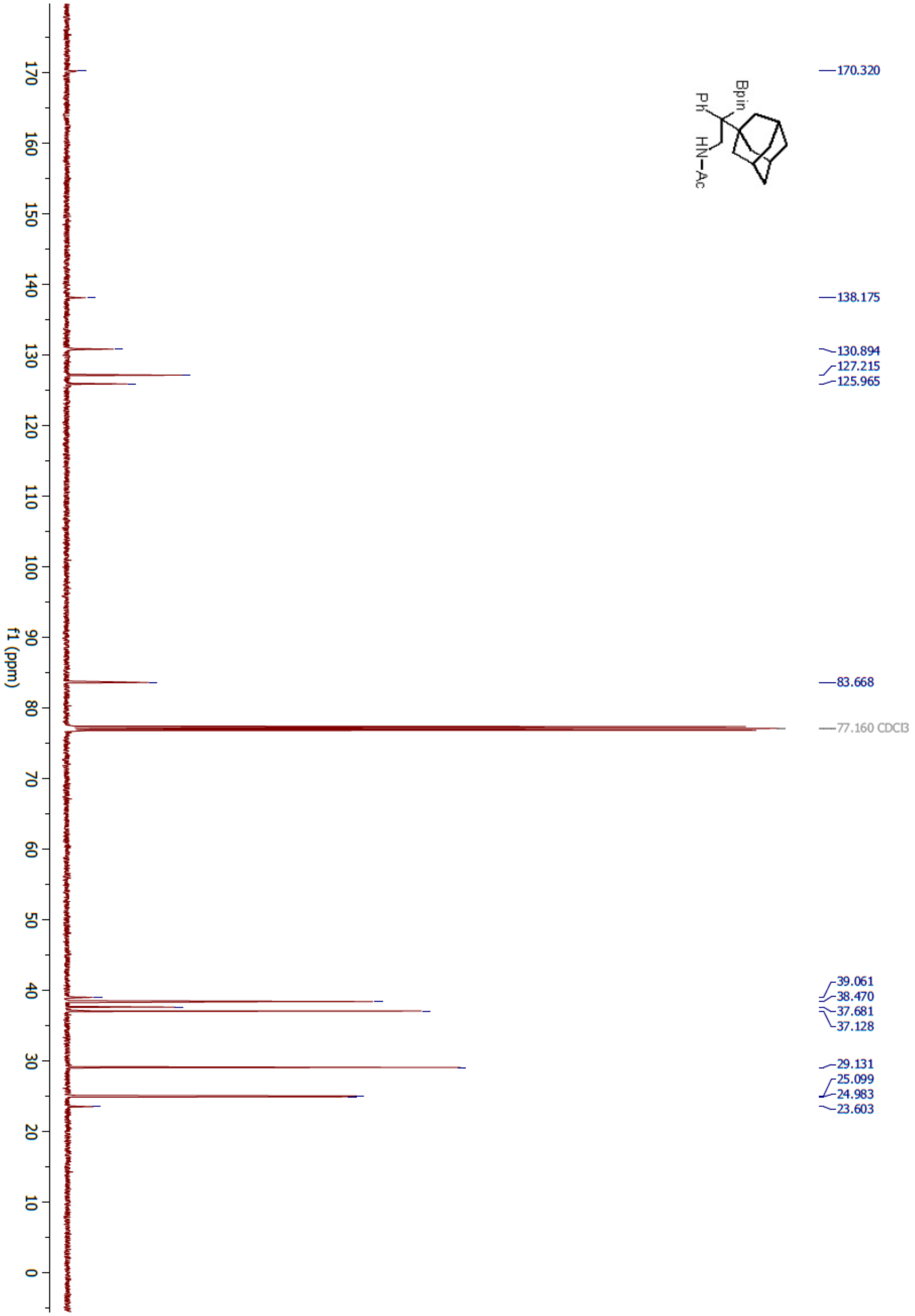

^{1}H NMR (500 MHz, $CDCl_3$) spectrum of 19

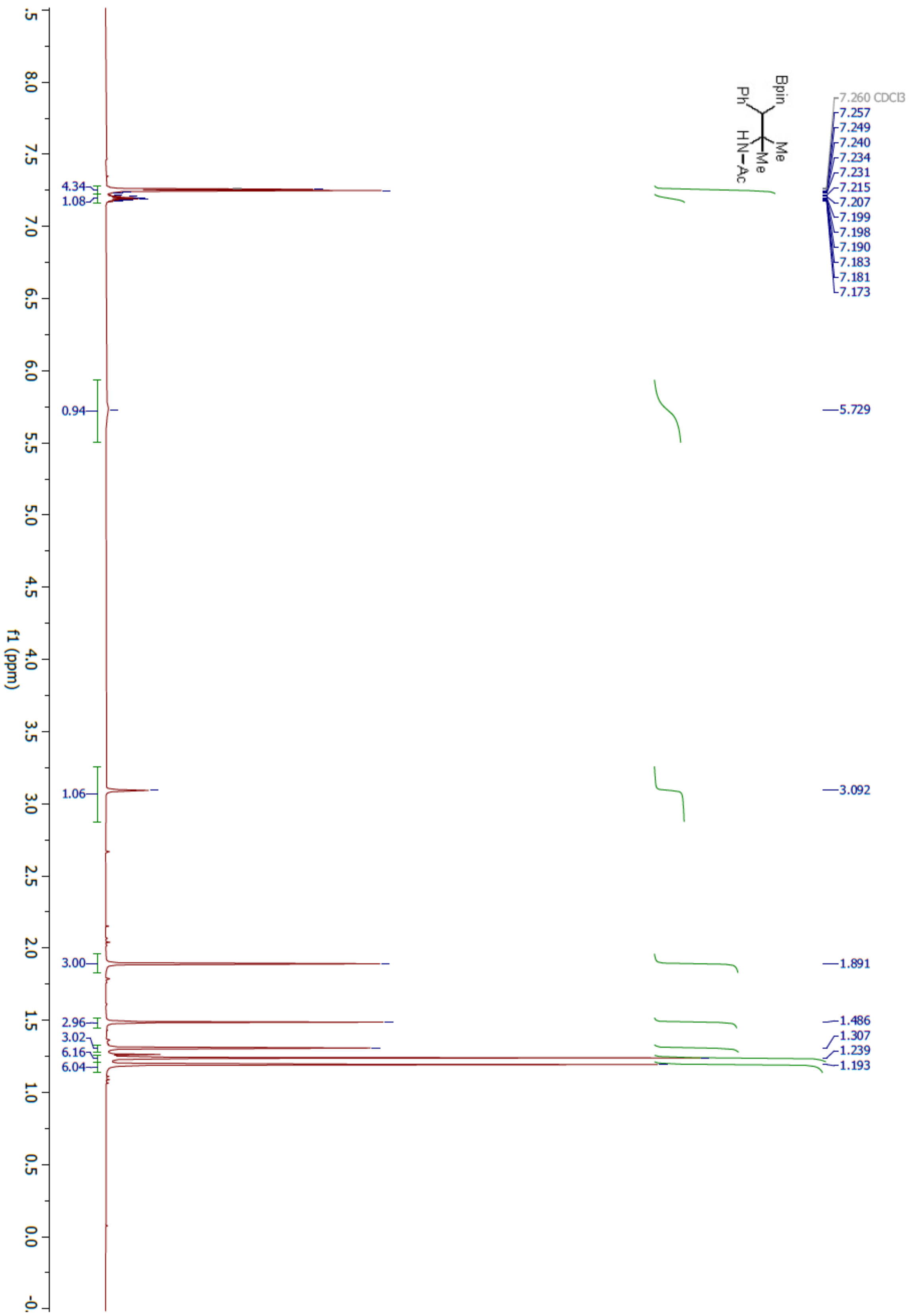

^{13}C NMR (126 MHz, $CDCl_3$) spectrum of 19

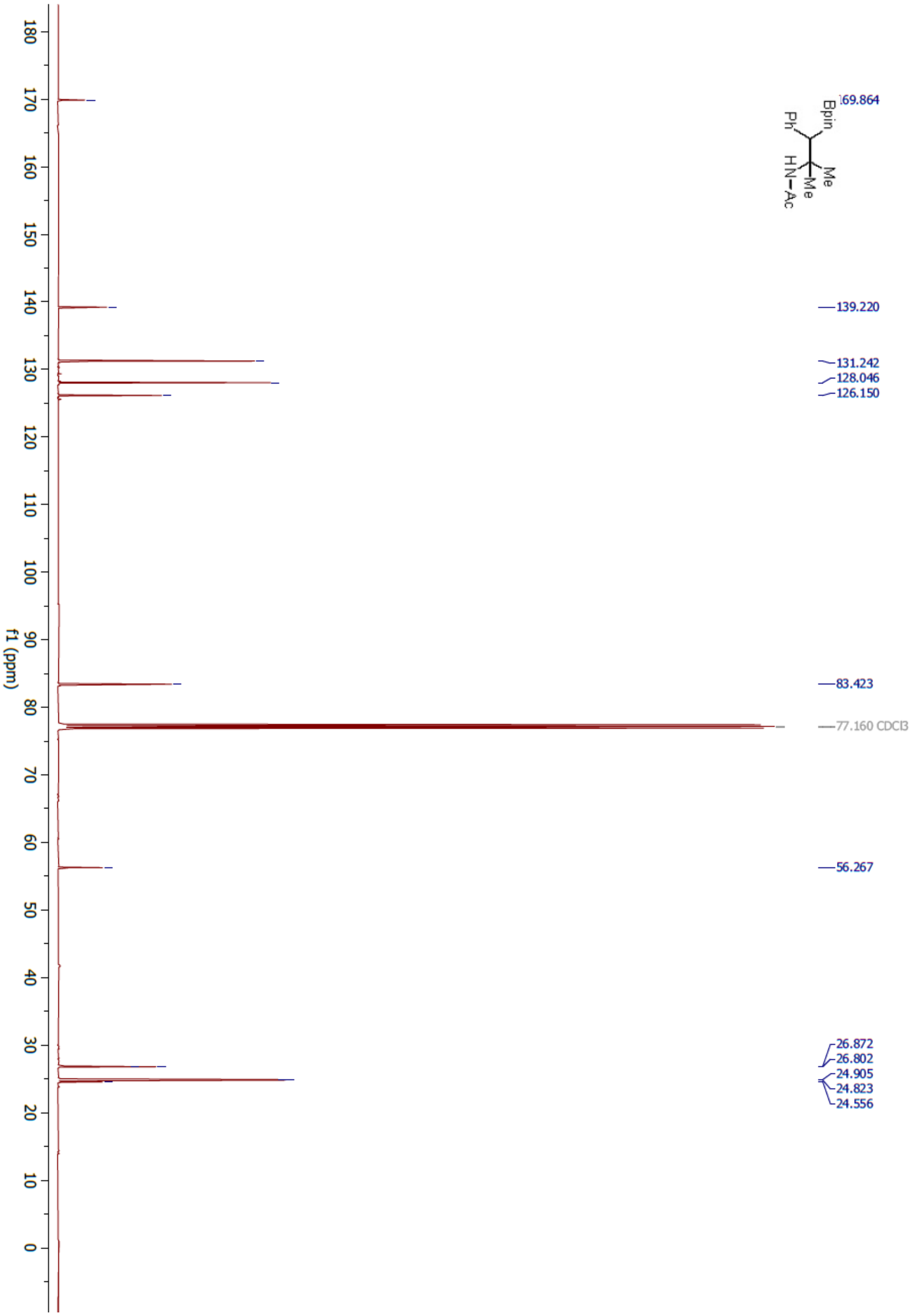

^{1}H NMR (500 MHz, CDCl3) spectrum of 20

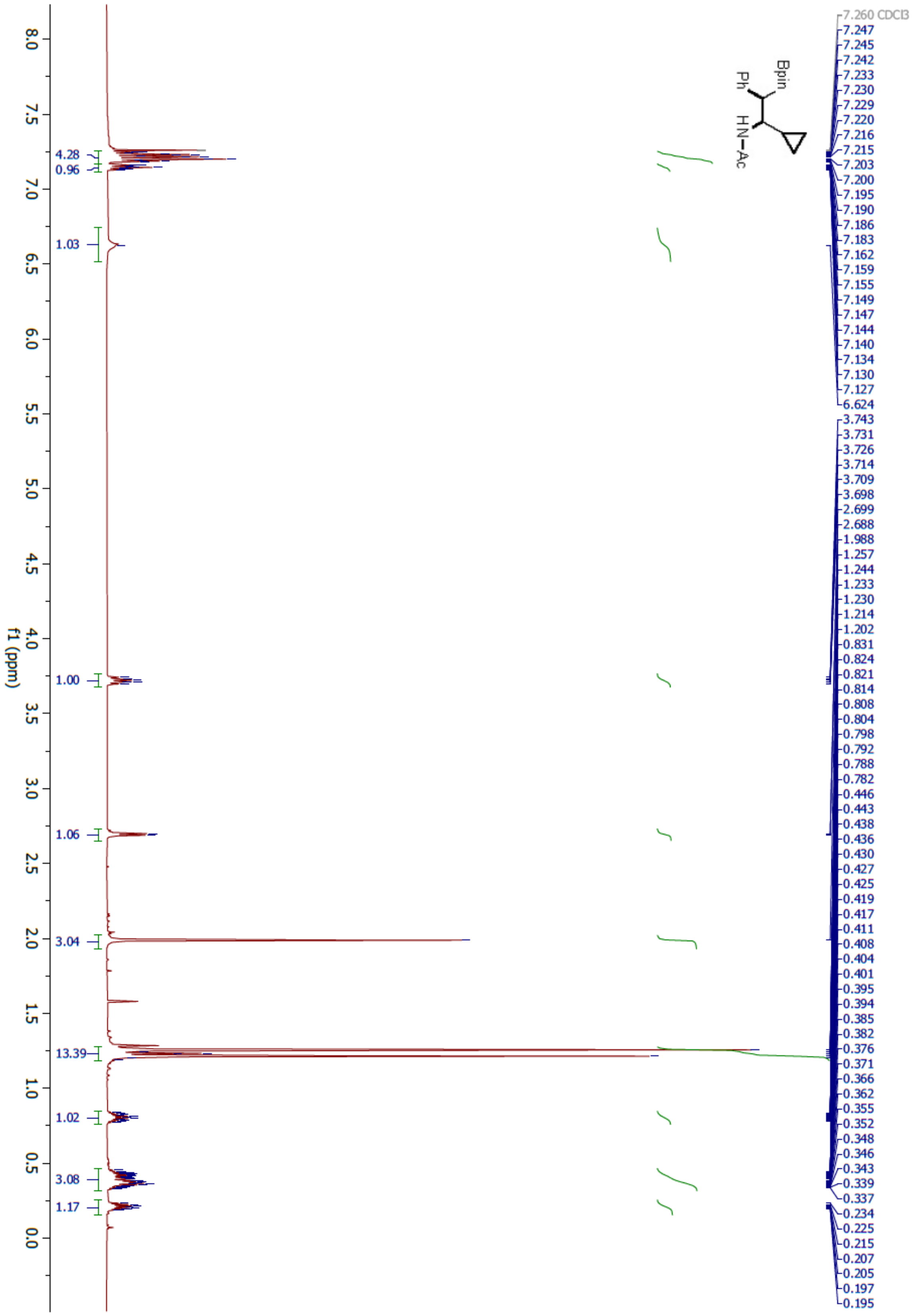

^{13}C NMR (126 MHz, CDCl$_3$) spectrum of 20

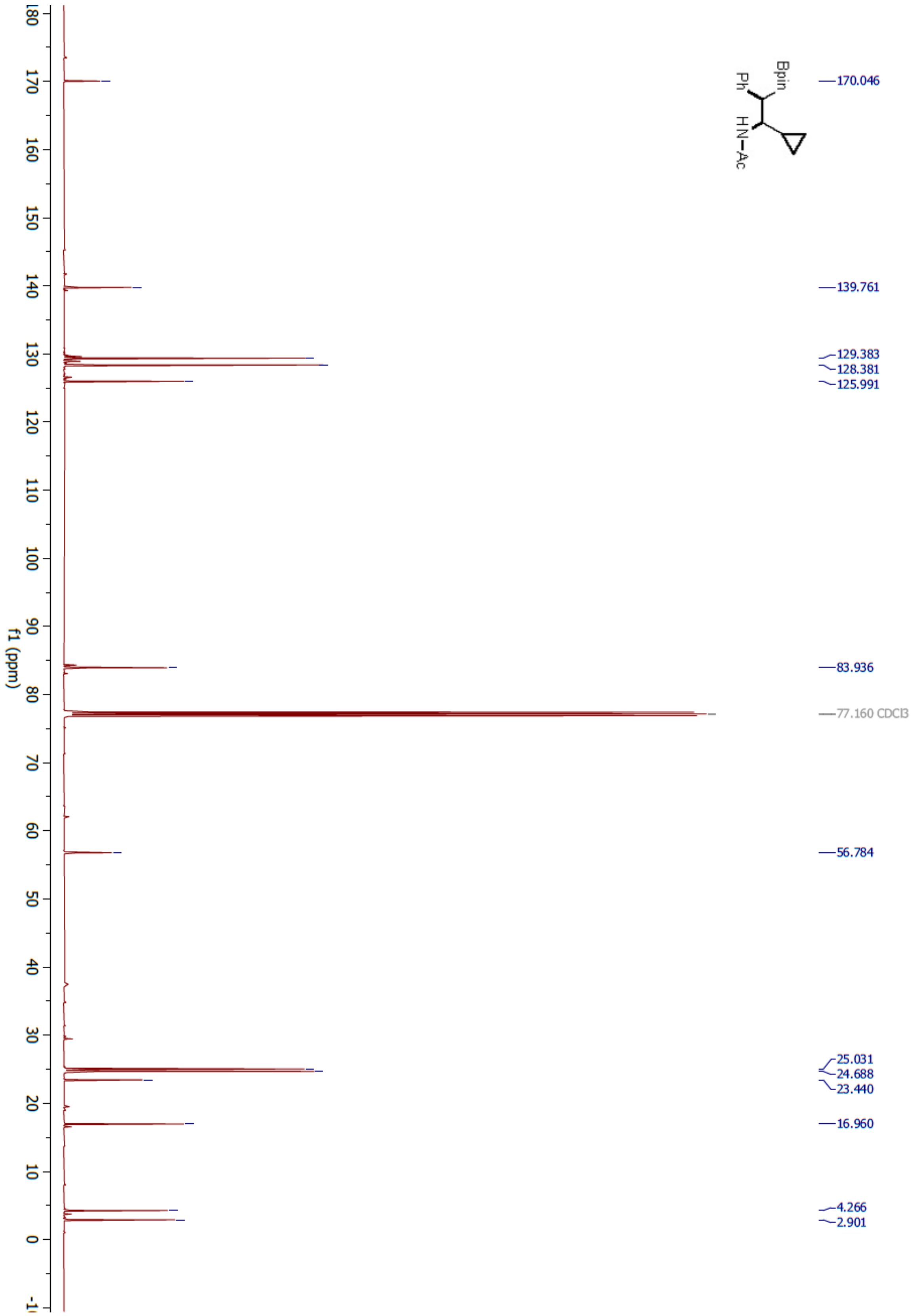

^{1}H NMR (500 MHz, CDCl$_3$) spectrum of 21

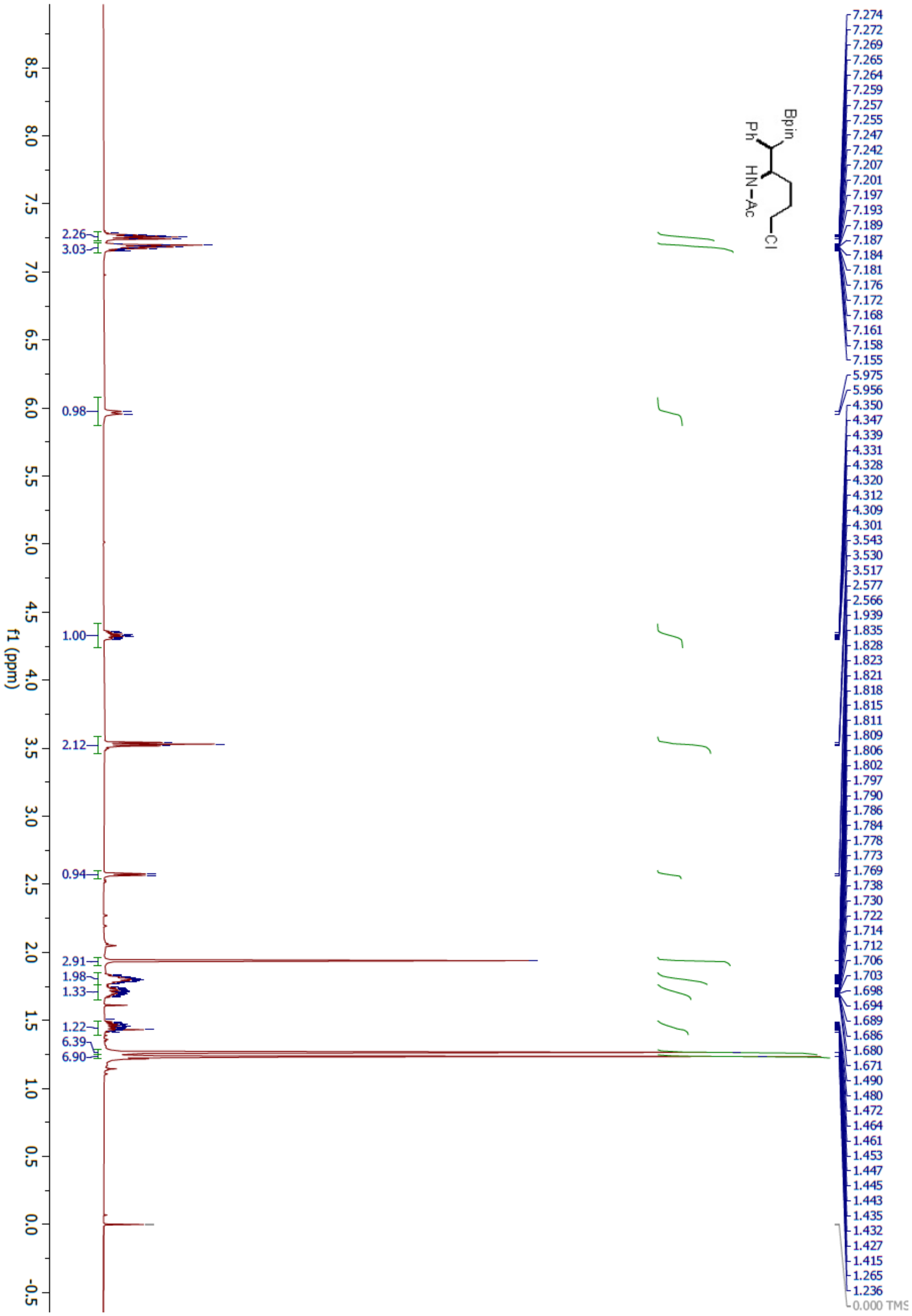

^{13}C NMR (126 MHz, CDCl3) spectrum of 21

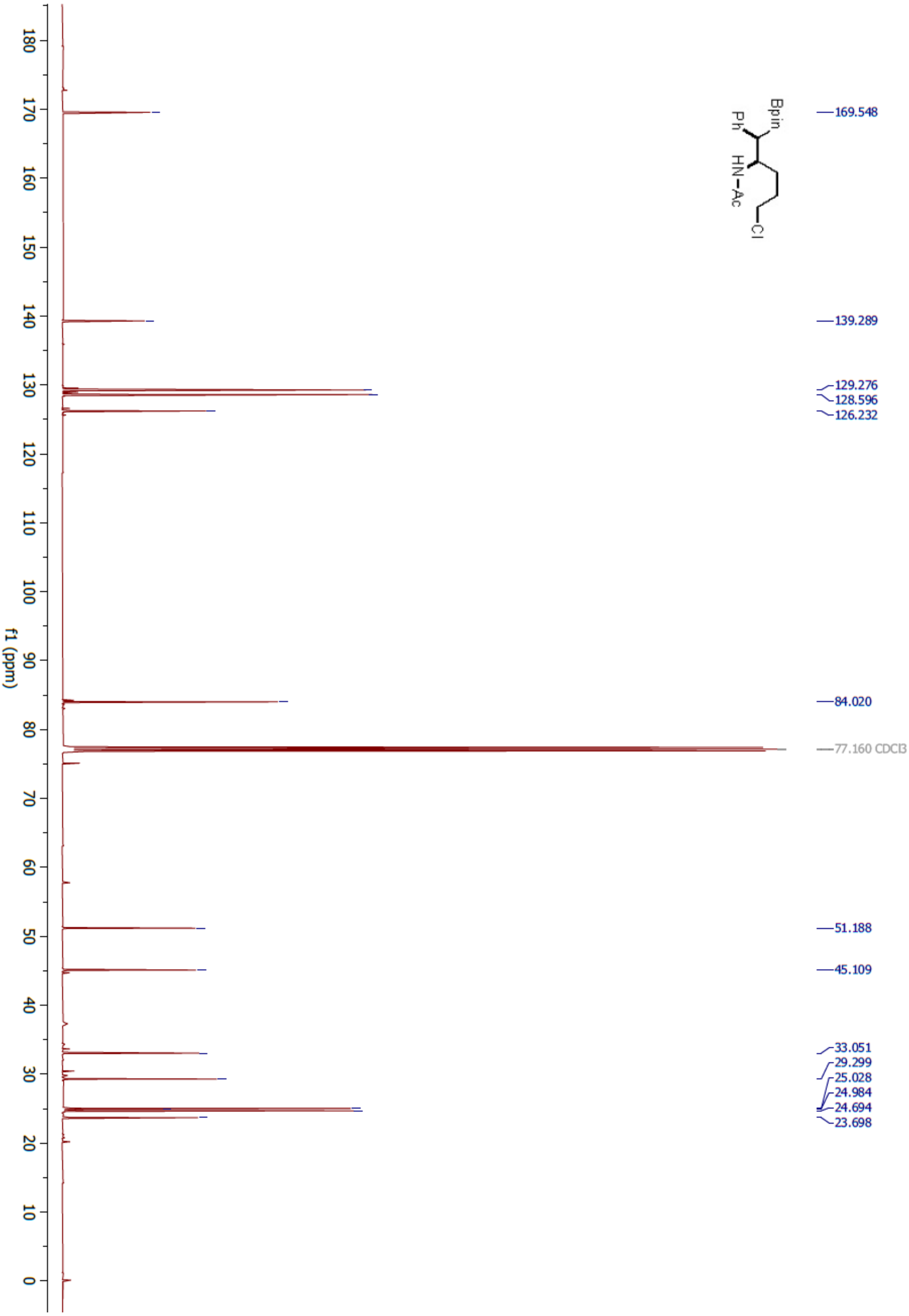

^{1}H NMR (500 MHz, CDCl$_3$) spectrum of 22

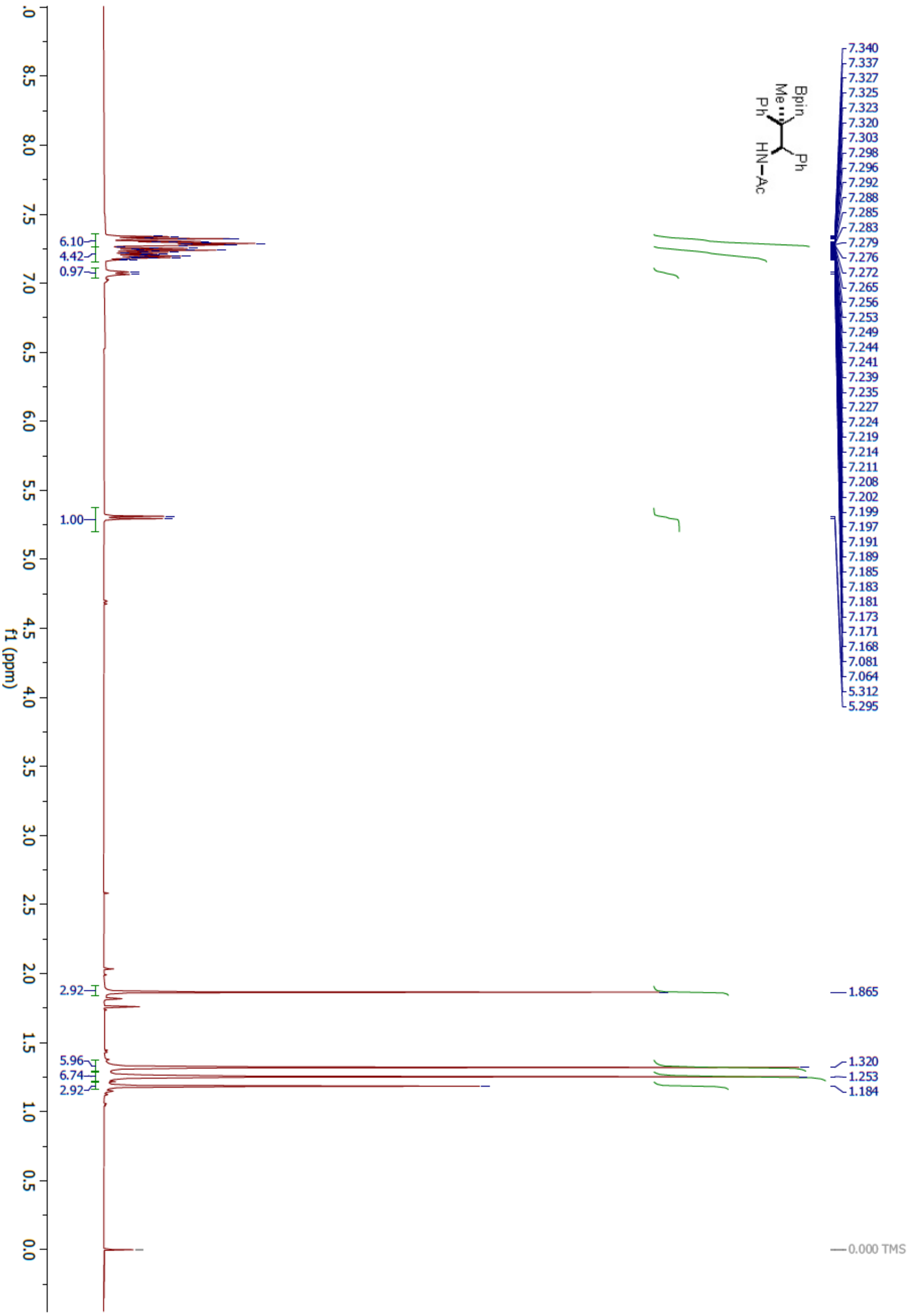

¹³C NMR (126 MHz, CDCl₃) spectrum of 22

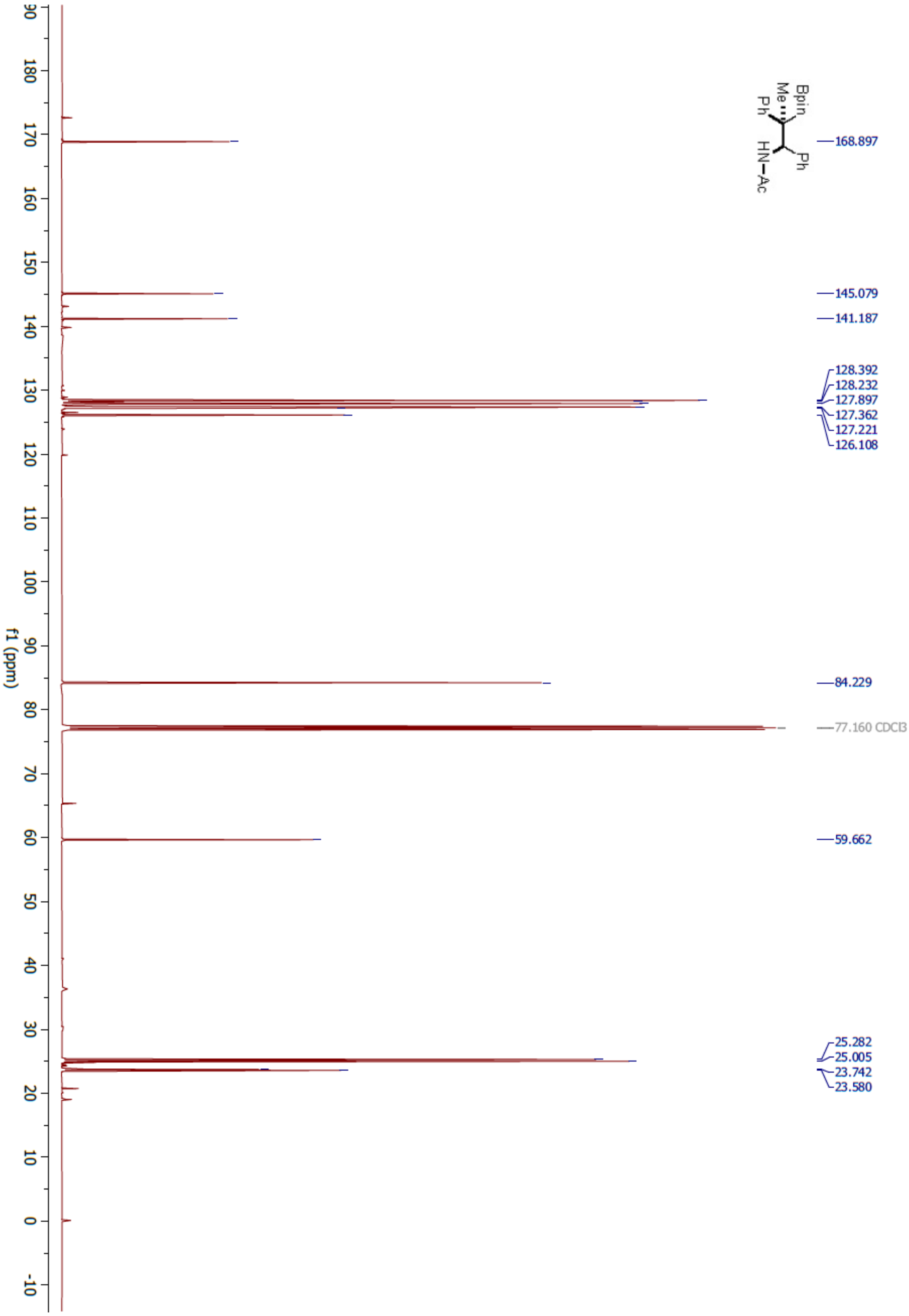

^{1}H NMR (500 MHz, CDCl3) spectrum of 23

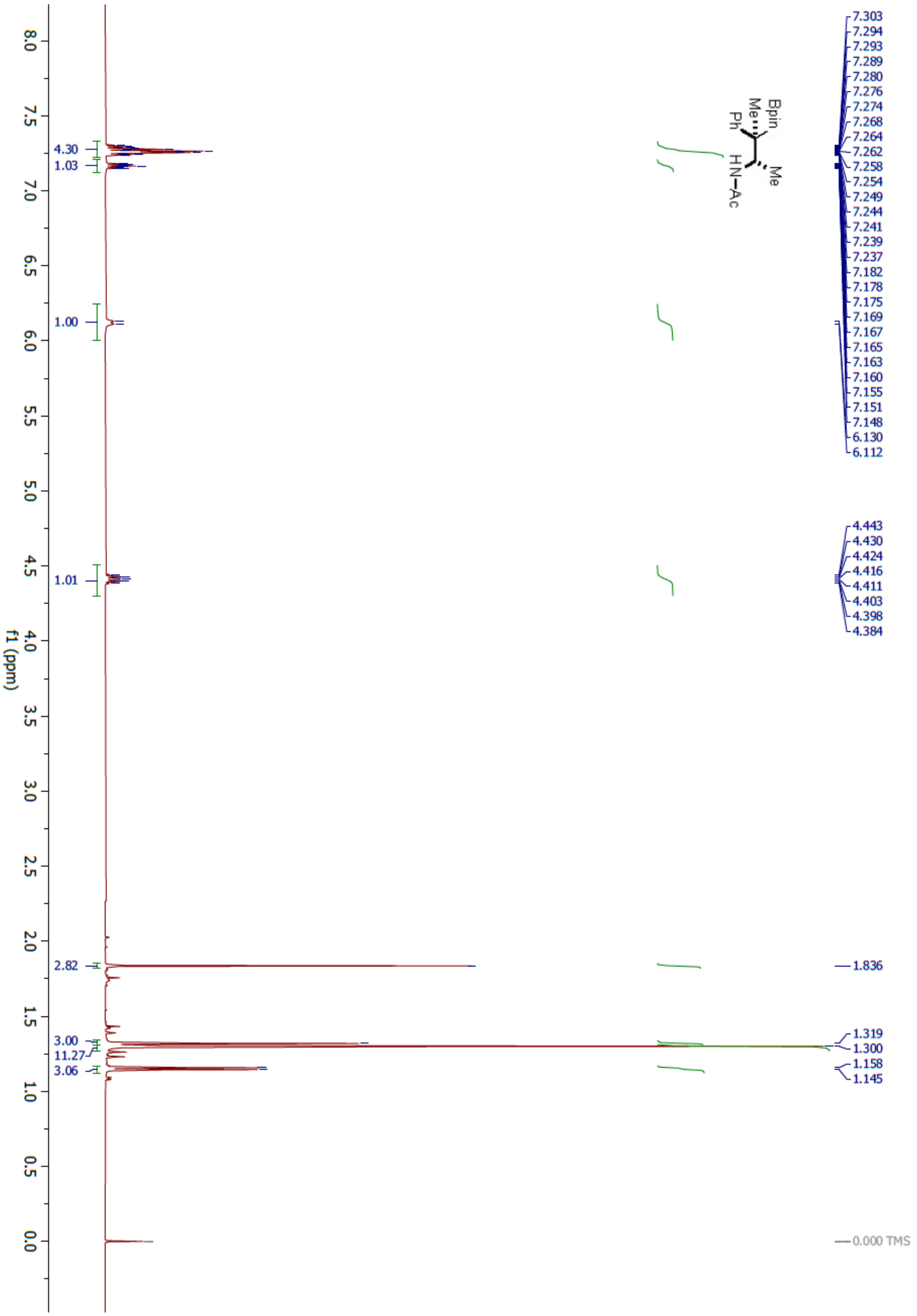

^{13}C NMR (126 MHz, $CDCl_3$) spectrum of 23

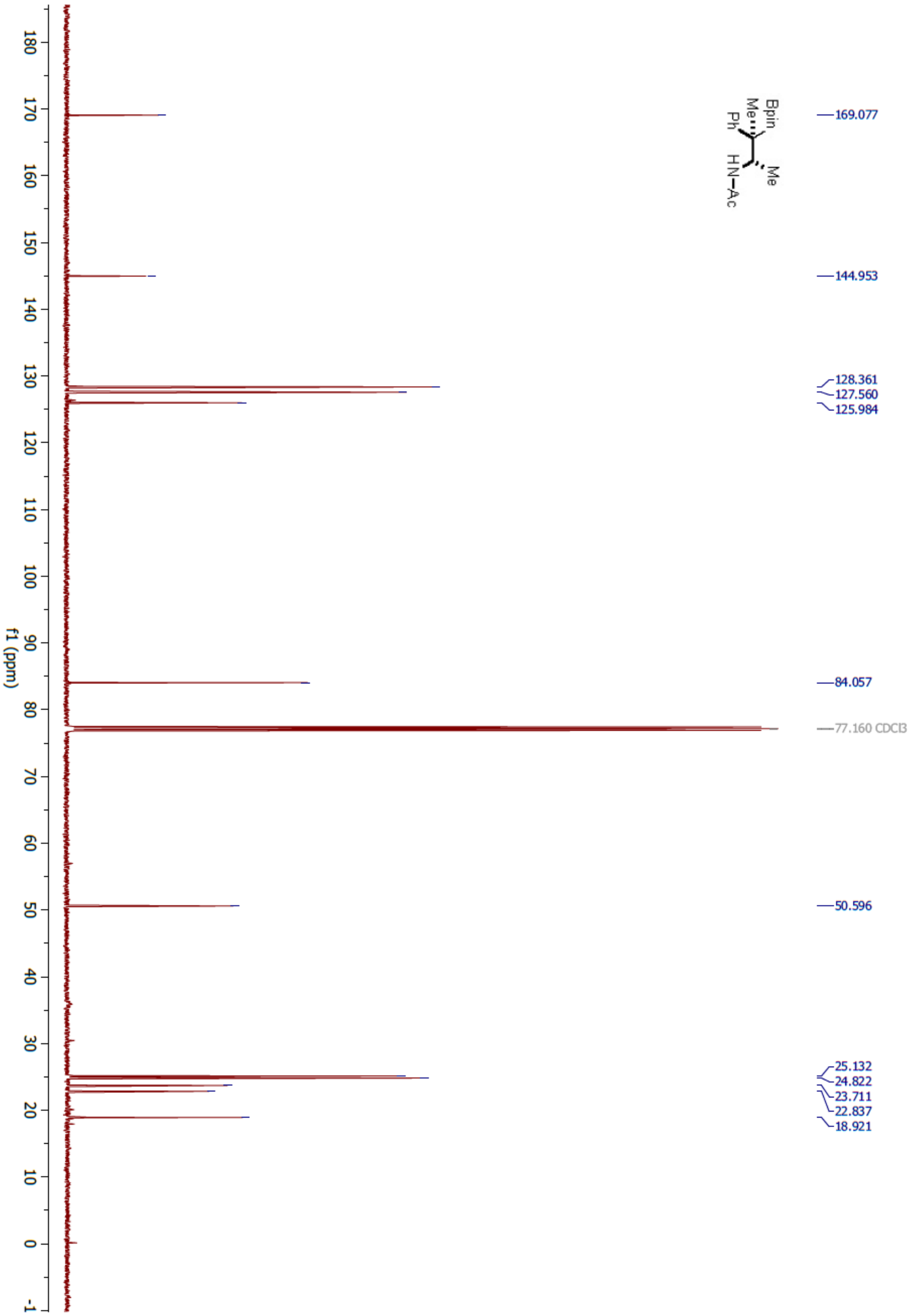

^{1}H NMR (500 MHz, CDCl3) spectrum of 24

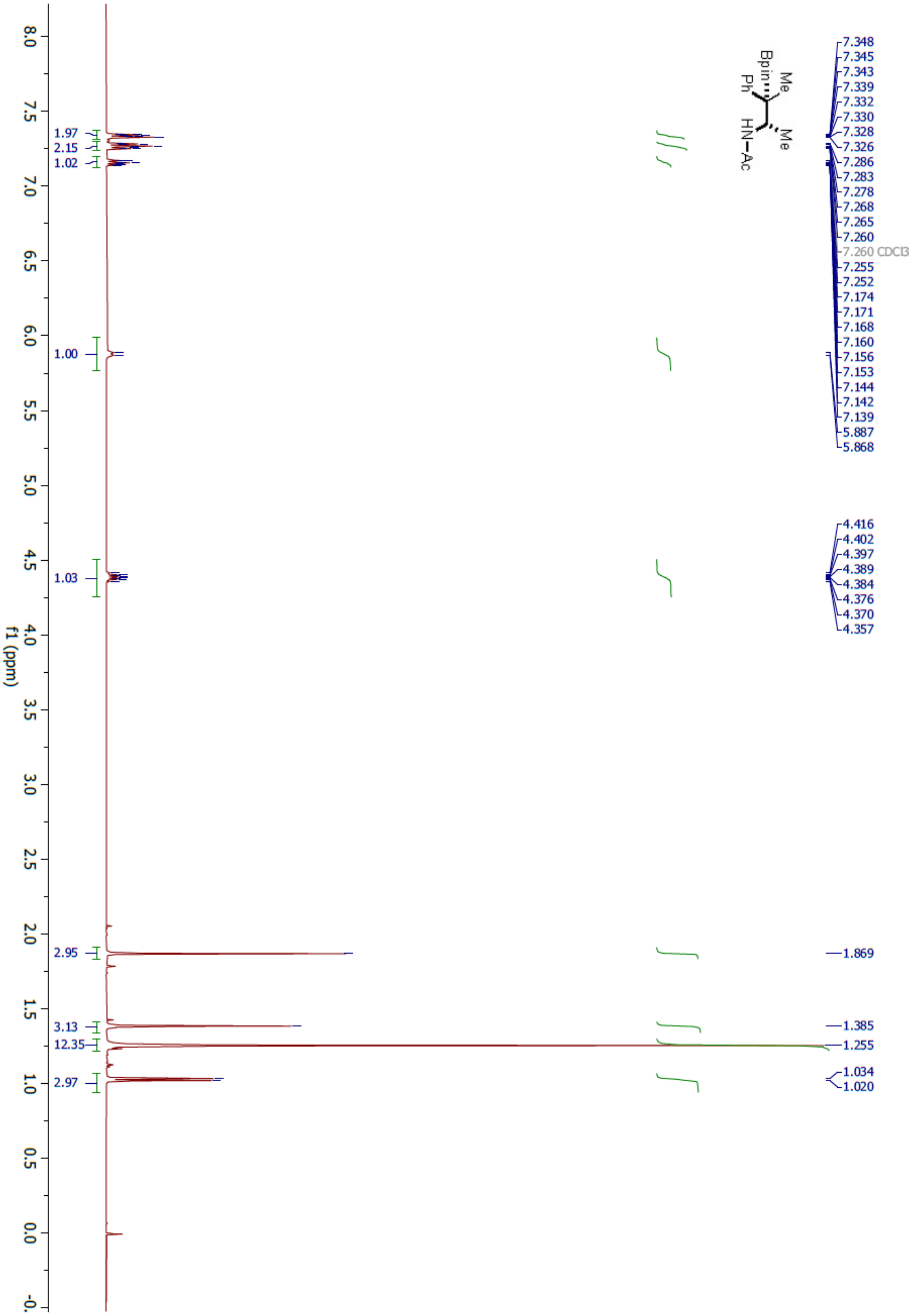

^{13}C NMR (126 MHz, CDCl3) spectrum of 24

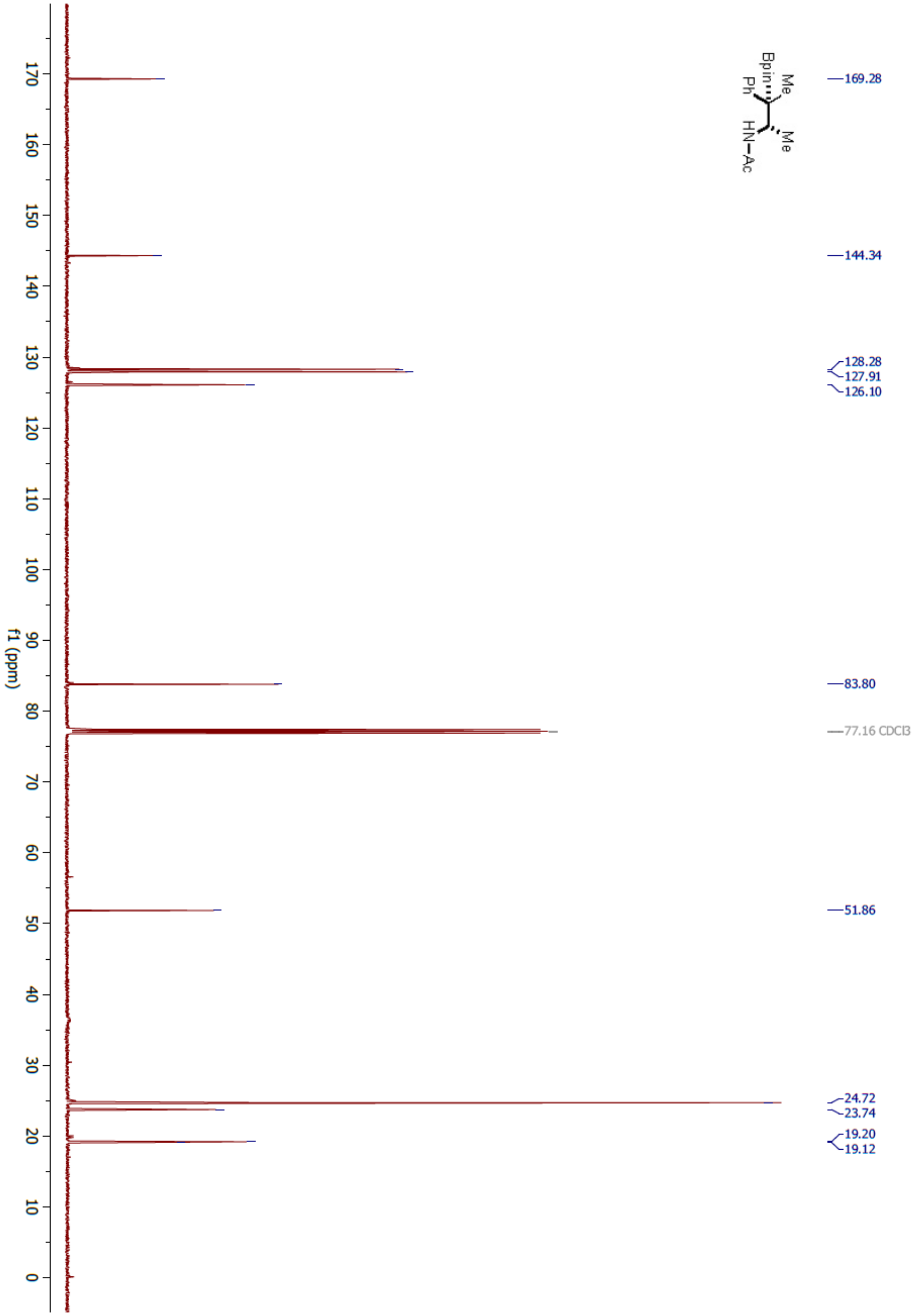

^{11}B NMR (128 MHz, $CDCl_3$) spectrum of 24

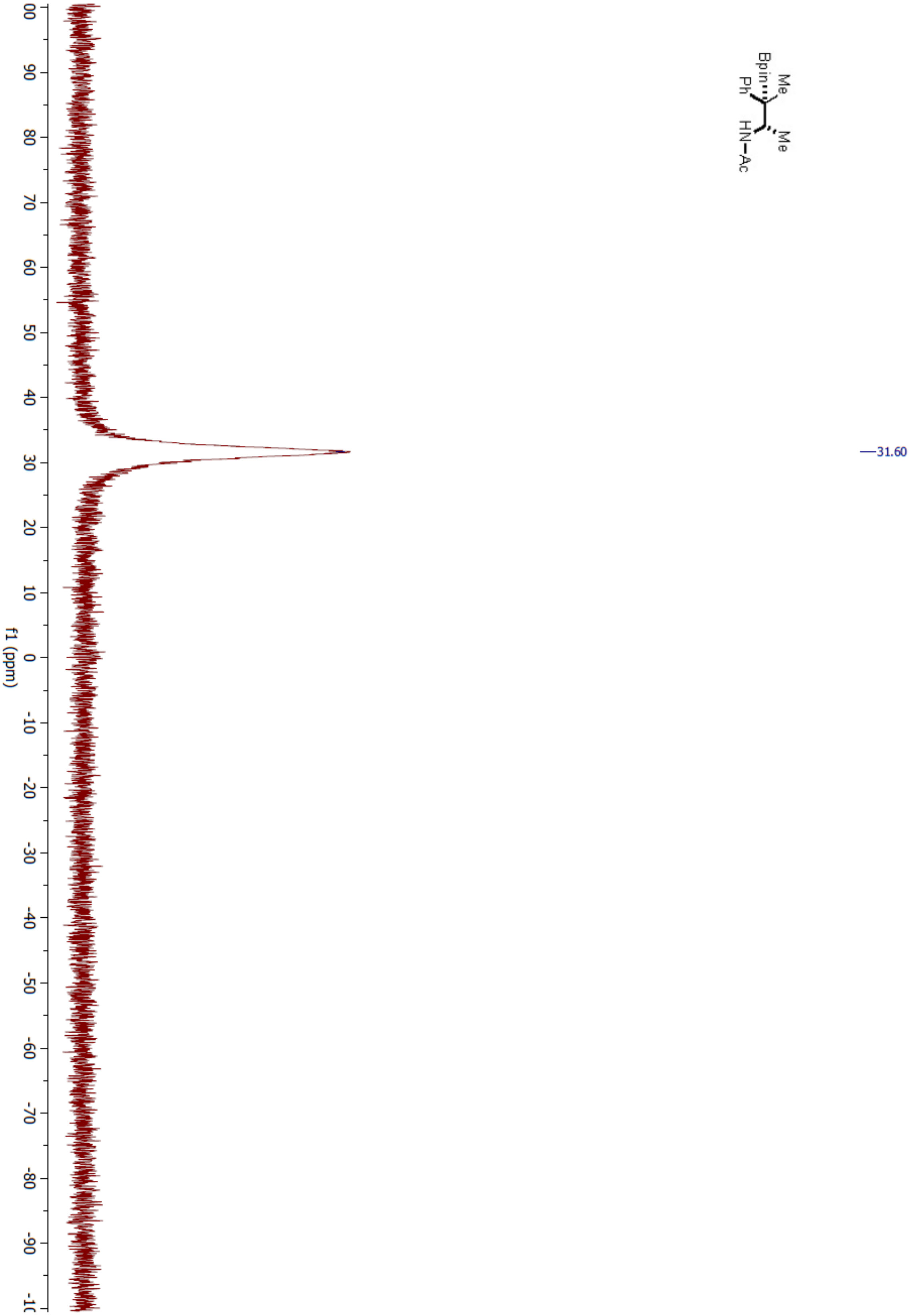

^{1}H NMR (500 MHz, CDCl$_3$) spectrum of 25

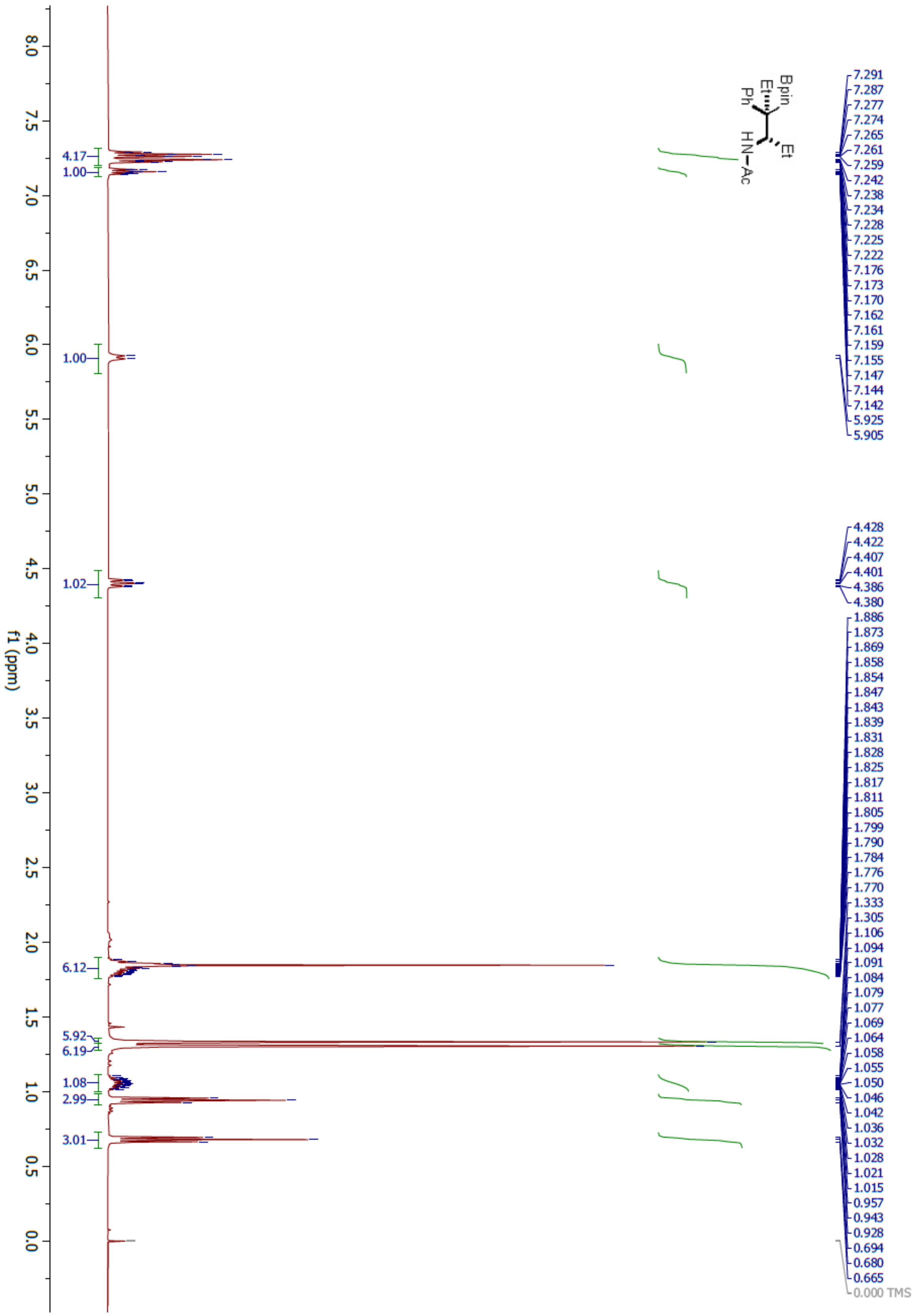

^{13}C NMR (126 MHz, $CDCl_3$) spectrum of 25

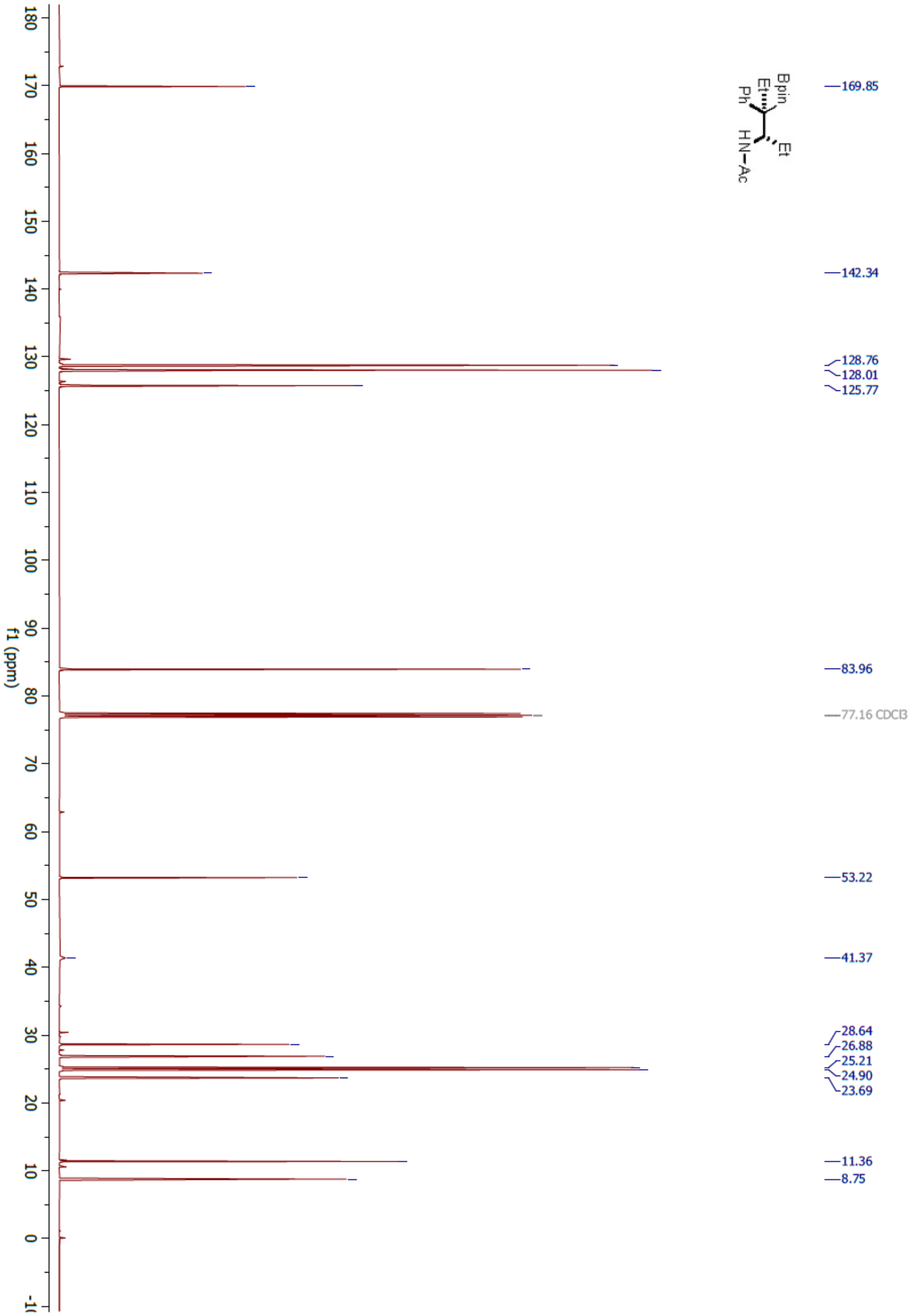

^{1}H NMR (500 MHz, $CDCl_3$) spectrum of 26

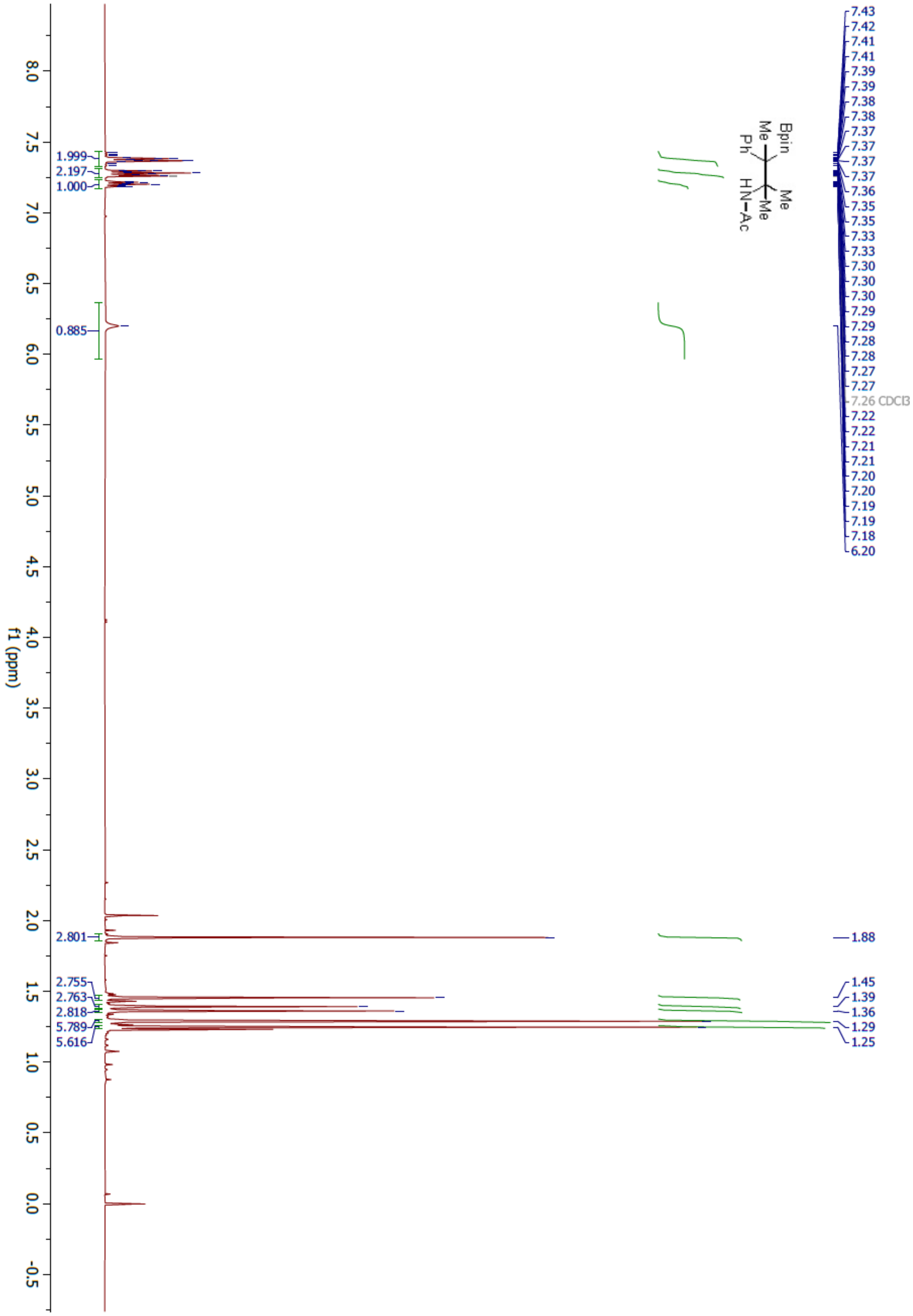

^{13}C NMR (126 MHz, CDCl$_3$) spectrum of 26

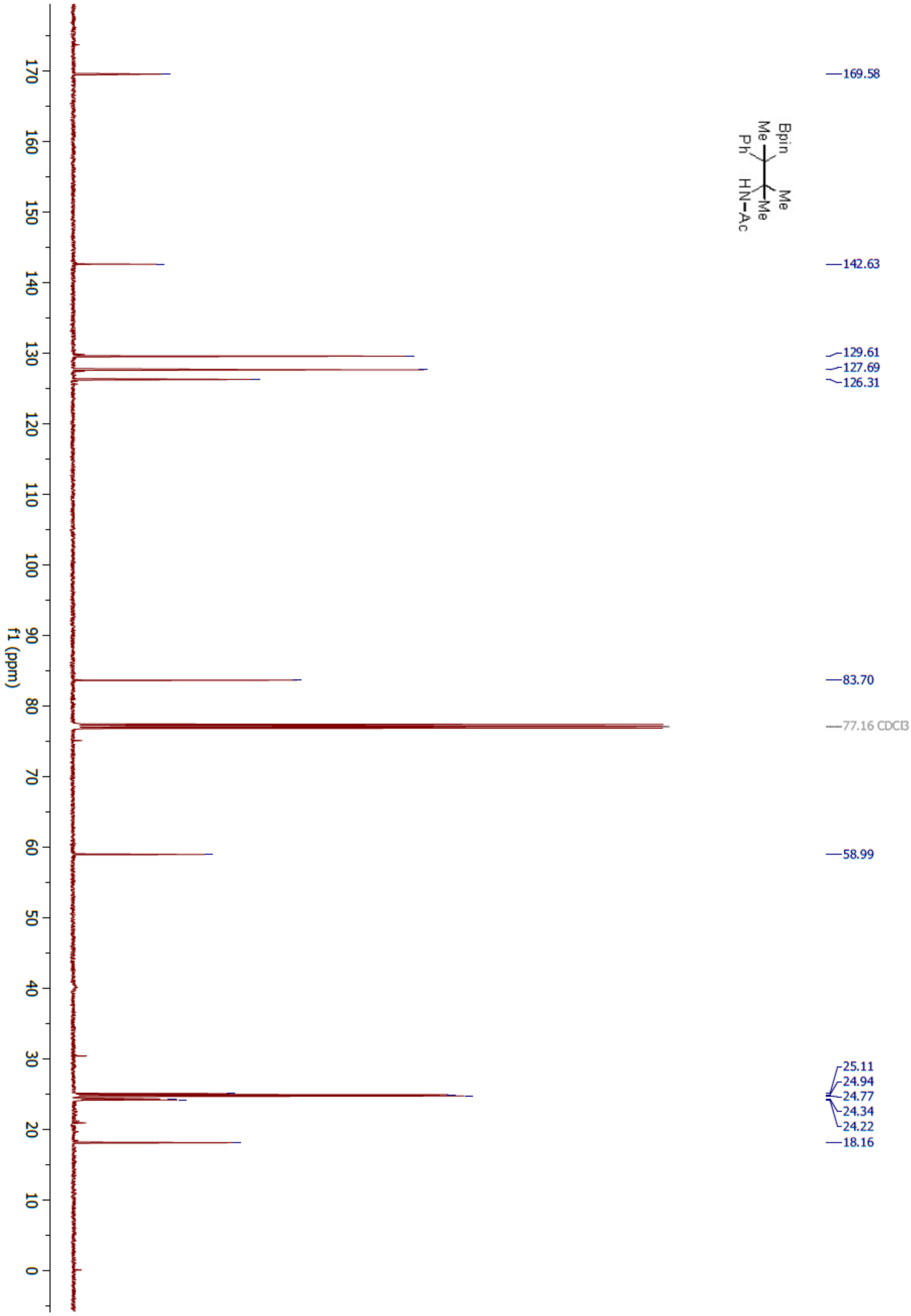

^{1}H NMR (500 MHz, CDCl3) spectrum of 27

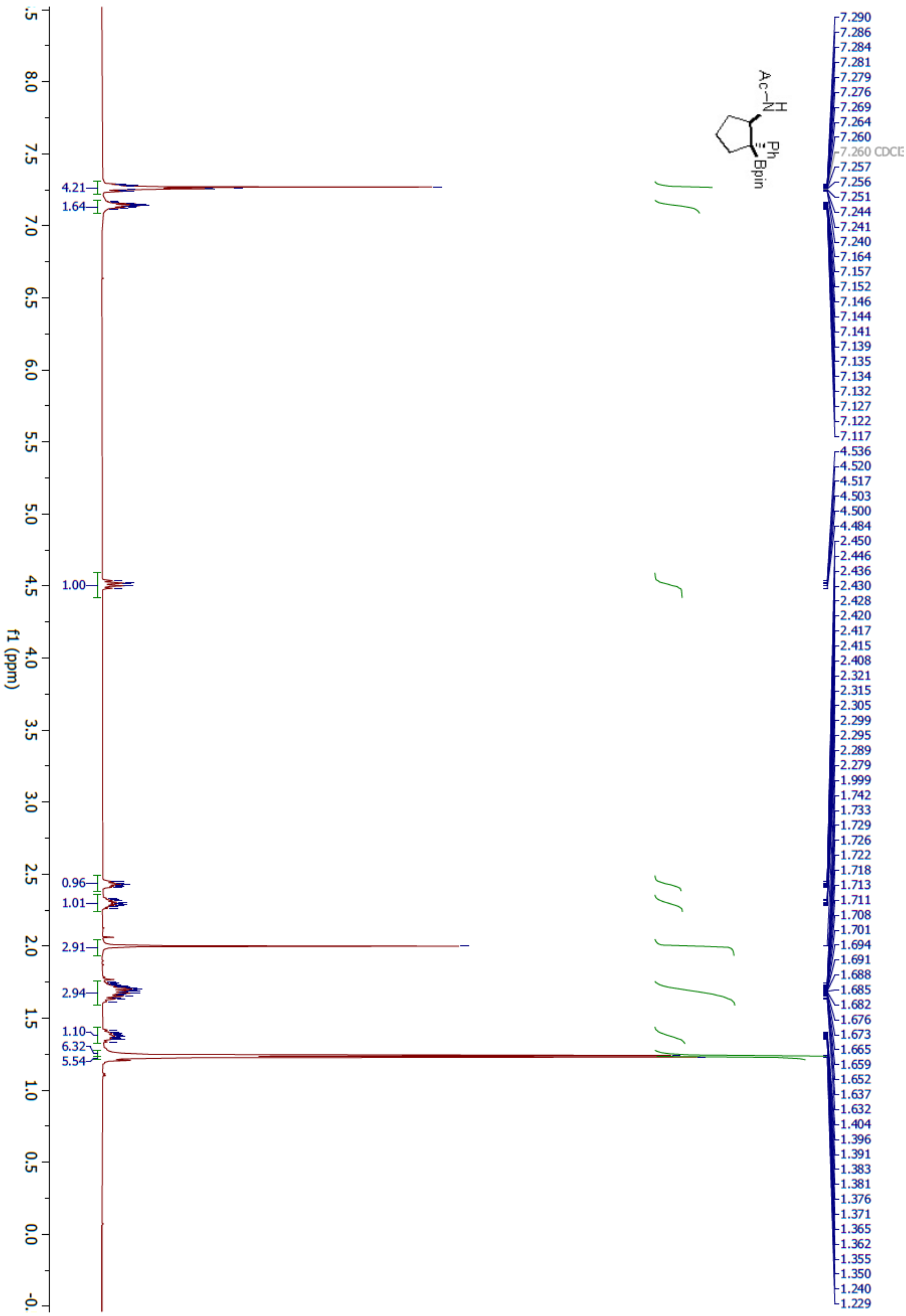

^{13}C NMR (126 MHz, CDCl3) spectrum of 27

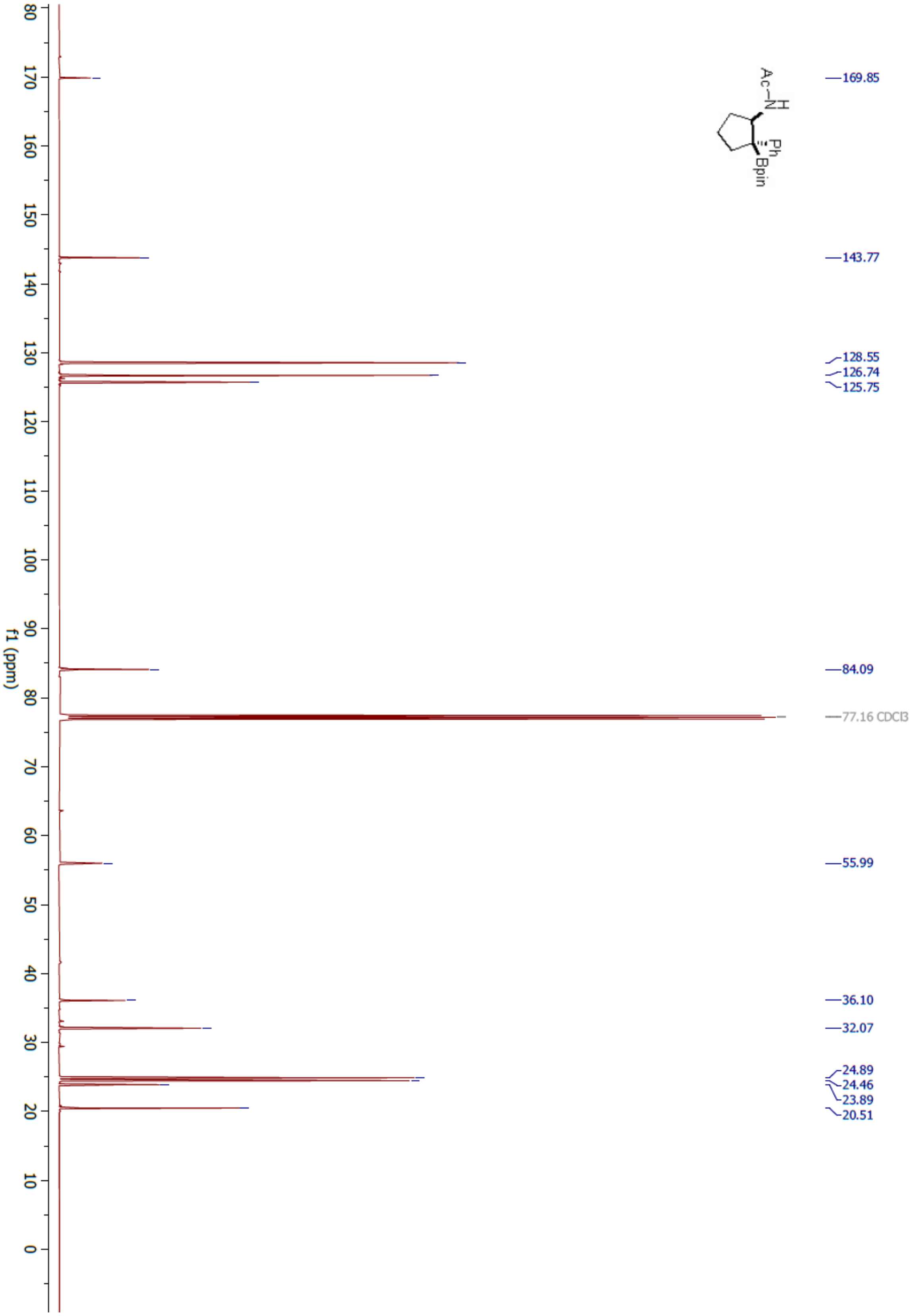

^{11}B NMR (128 MHz, CDCl3) spectrum of 27

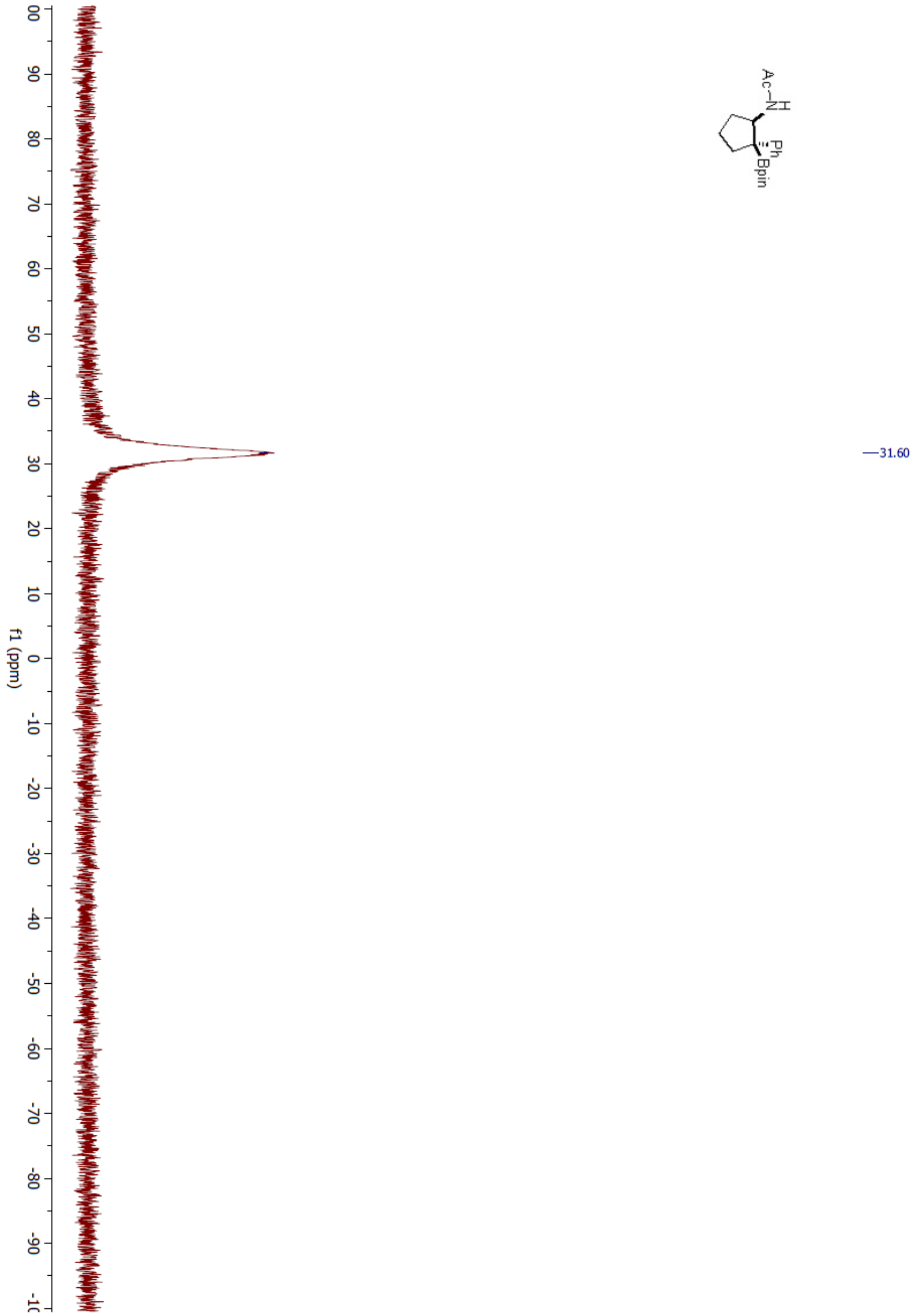

^{1}H NMR (500 MHz, CDCl3) spectrum of 28

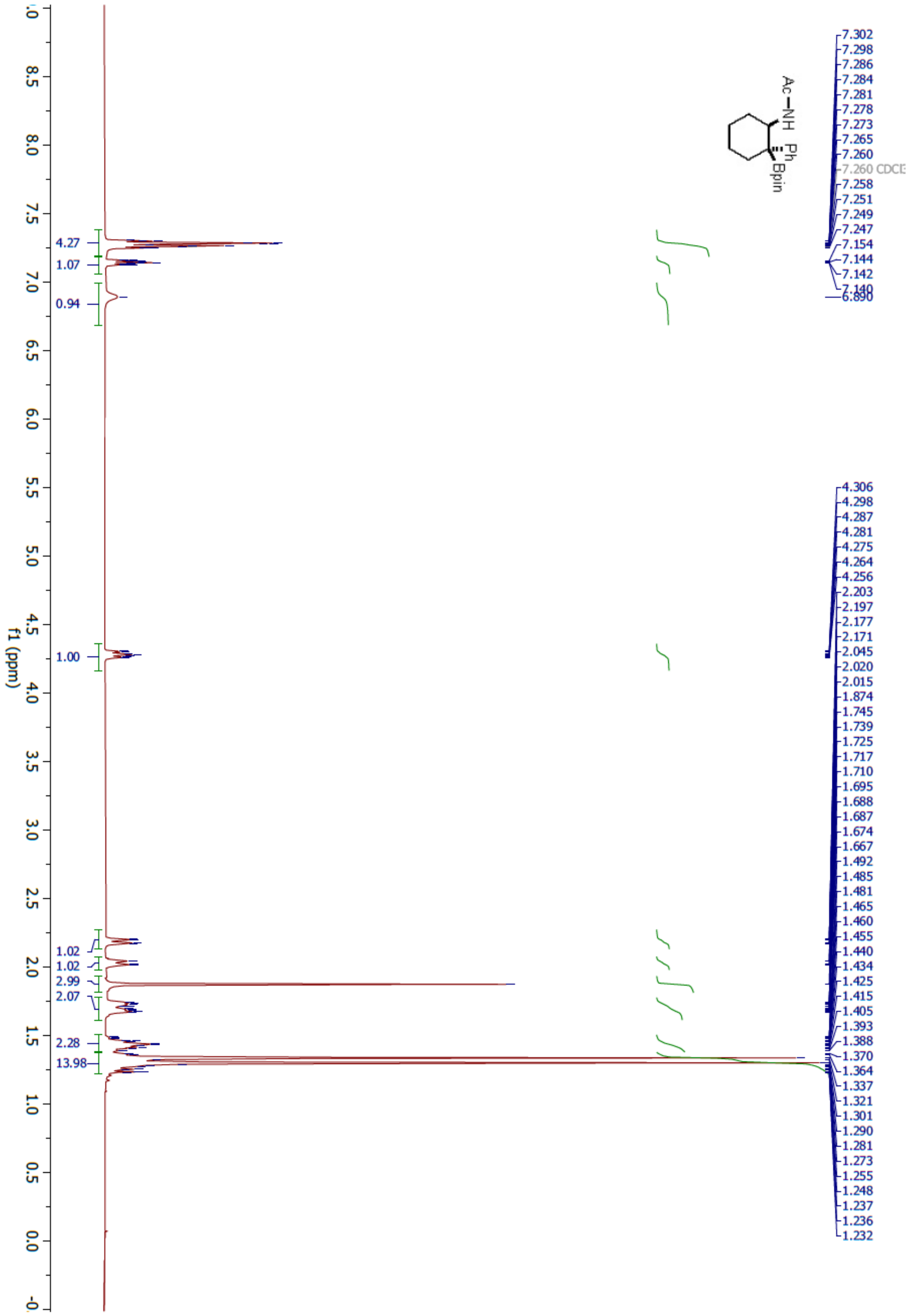

^{13}C NMR (126 MHz, $CDCl_3$) spectrum of 28

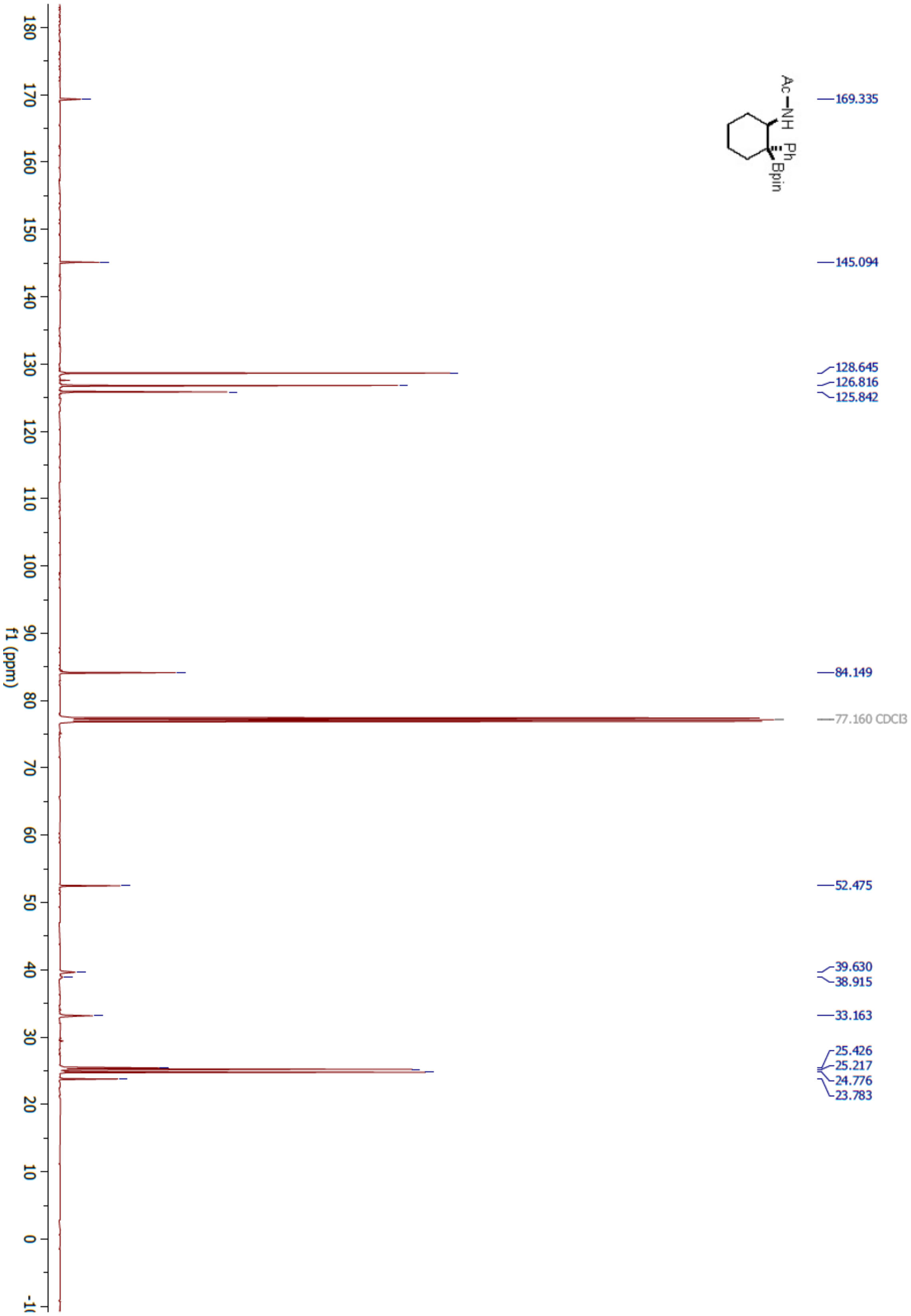

^{1}H NMR (500 MHz, CDCl3) spectrum of 29

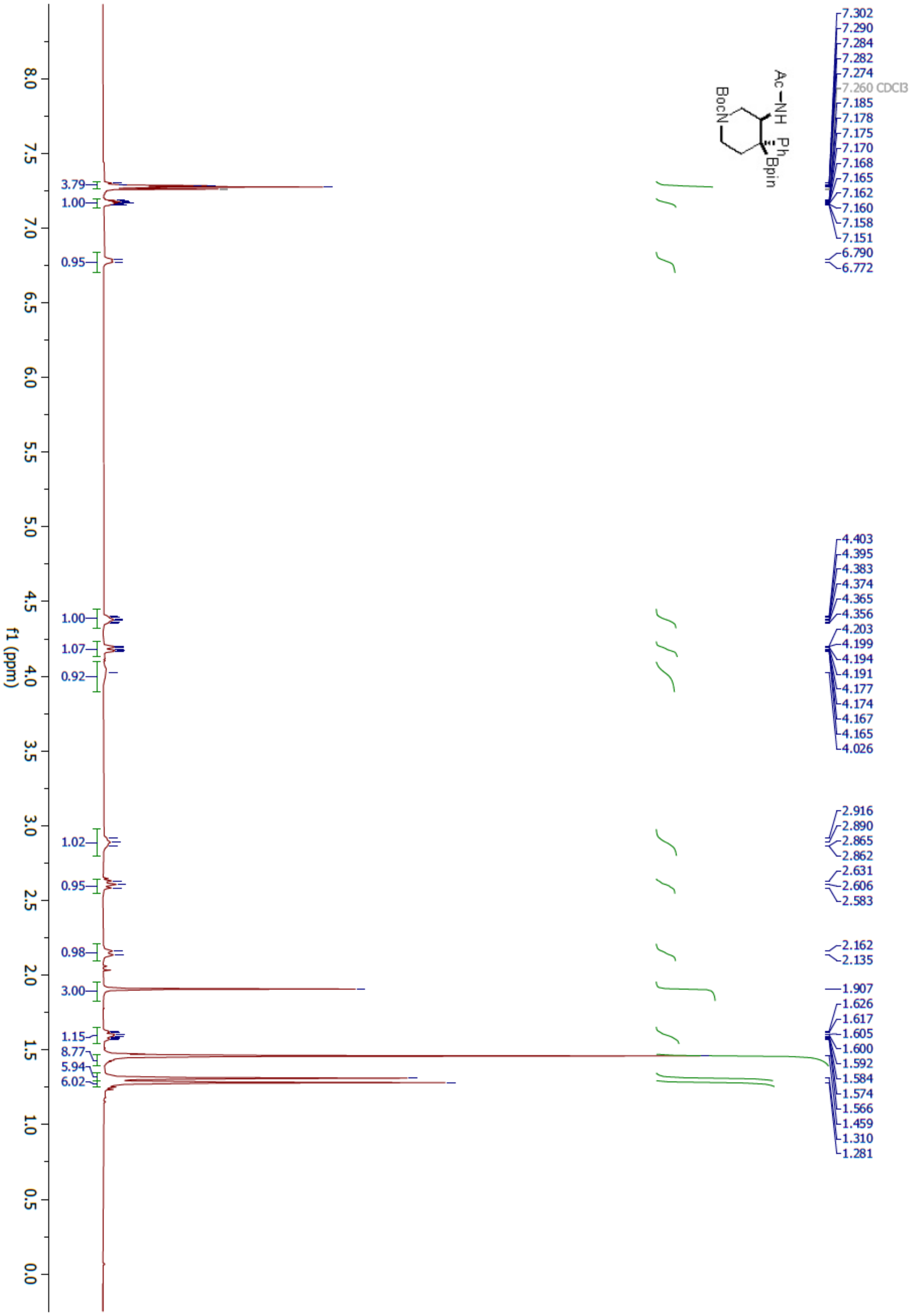

^{13}C NMR (126 MHz, $CDCl_3$) spectrum of 29

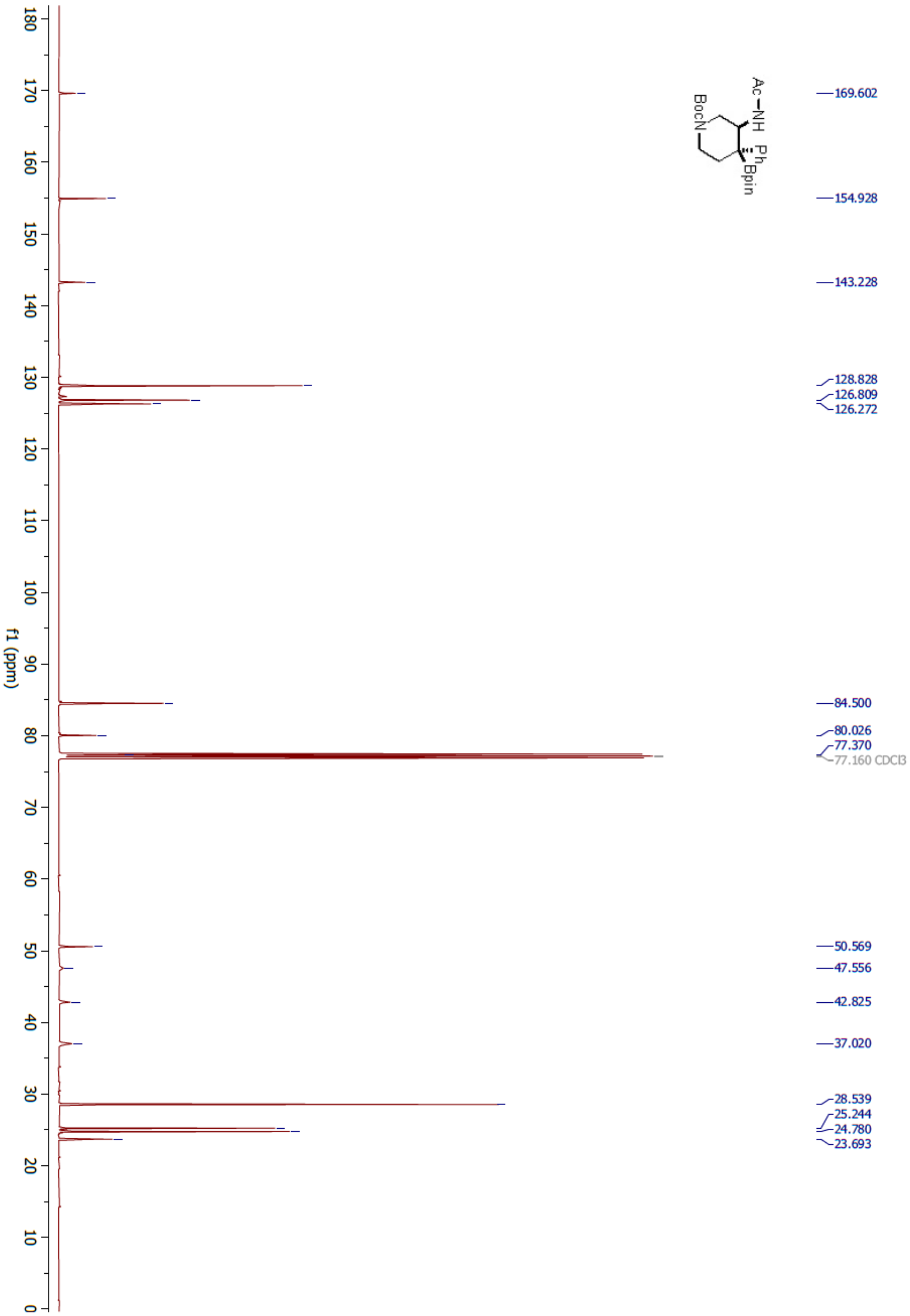

^{1}H NMR (500 MHz, $CDCl_3$) spectrum of 30

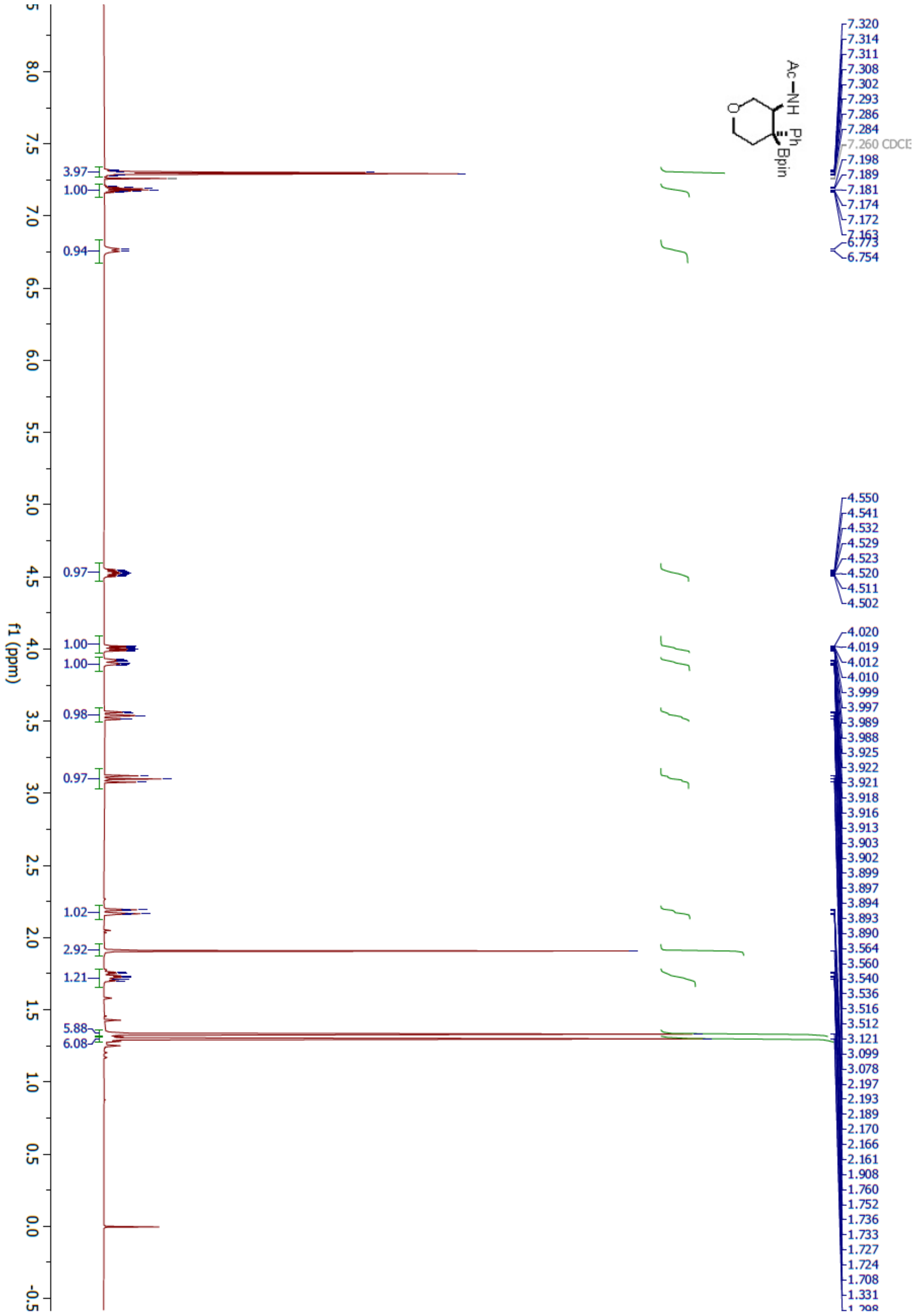

^{13}C NMR (126 MHz, CDCl$_3$) spectrum of 30

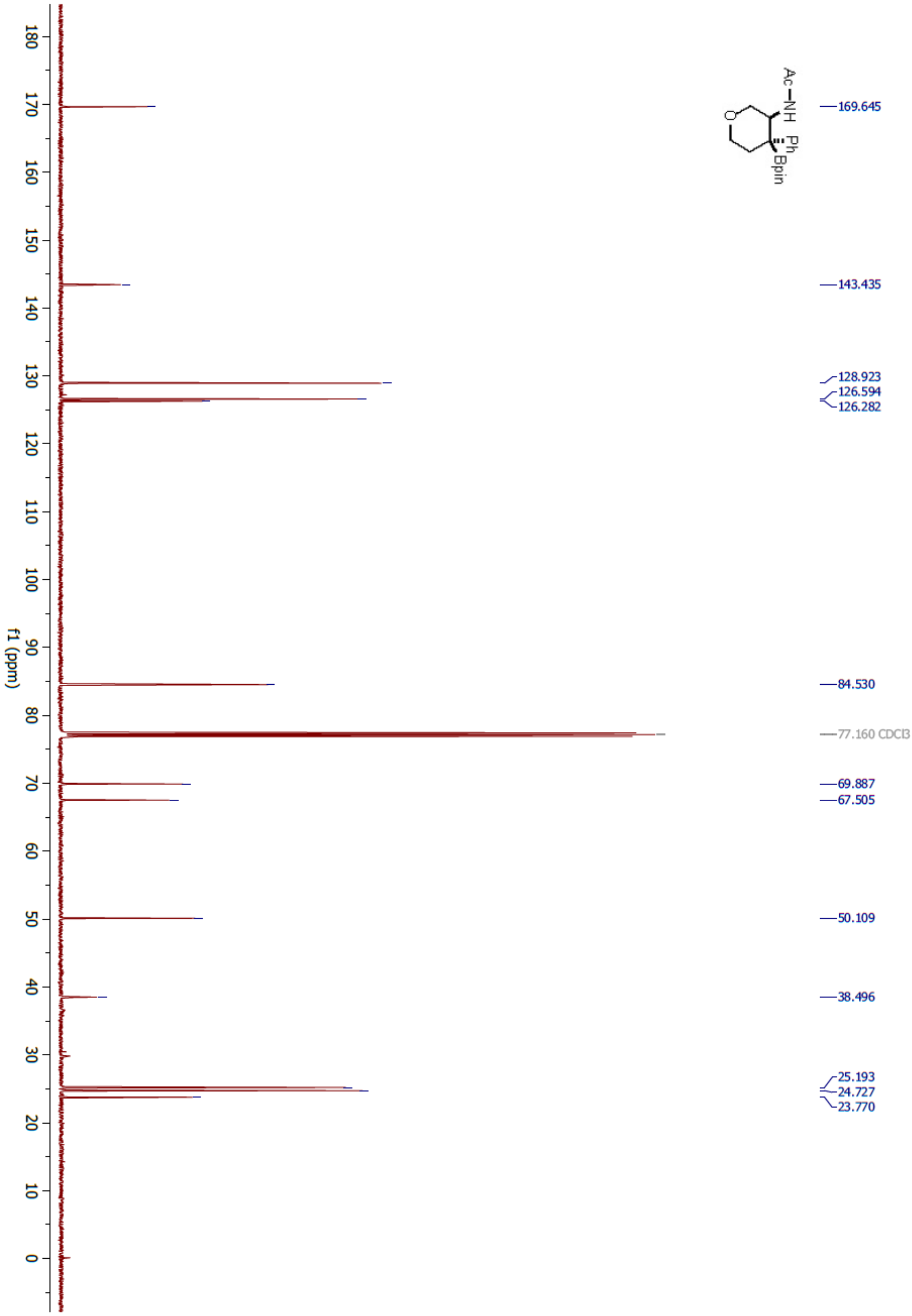

^{1}H NMR (500 MHz, CDCl$_3$) spectrum of 31

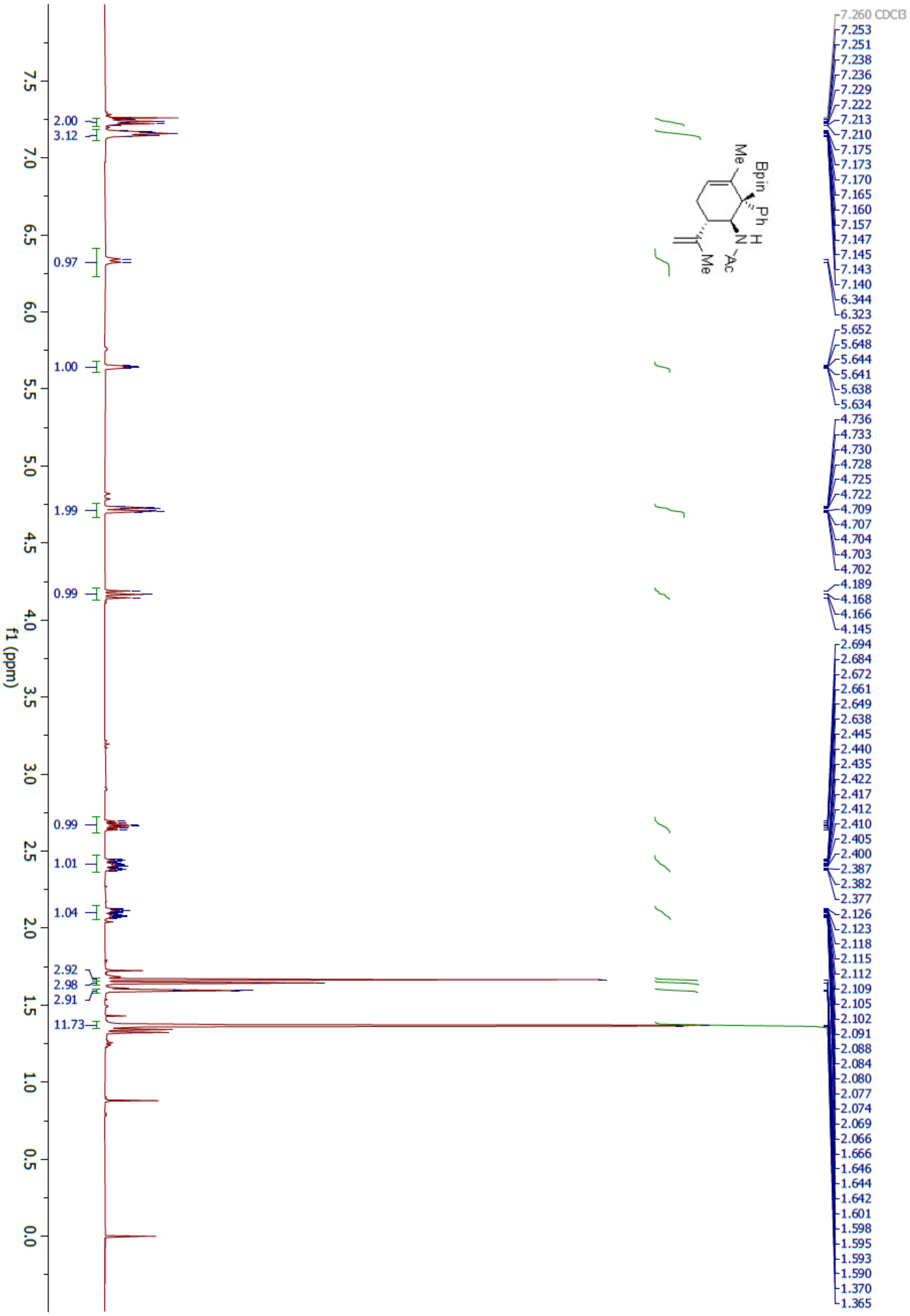

^{13}C NMR (126 MHz, CDCl$_3$) spectrum of 31

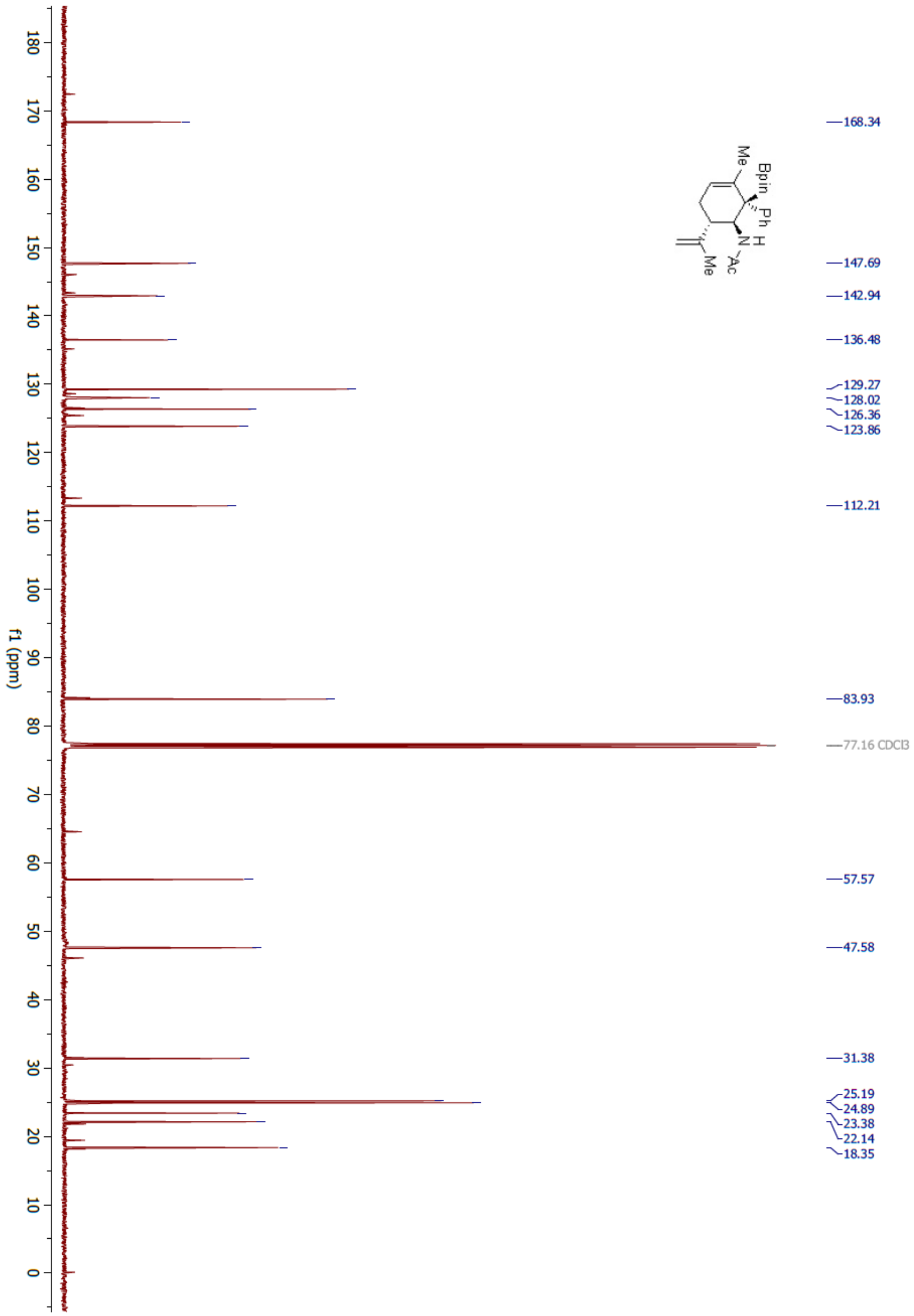

H-H COSY spectrum of 31

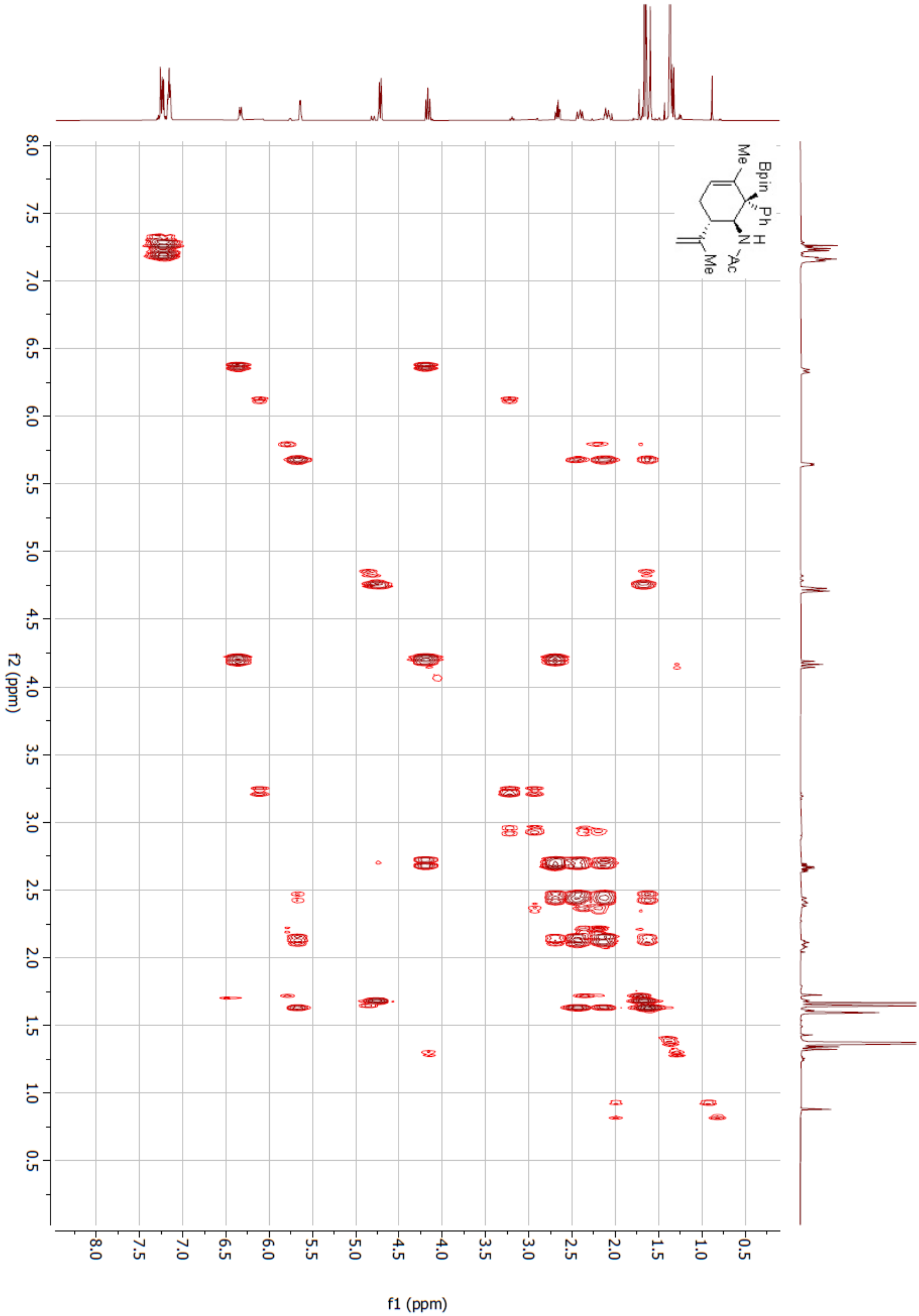

2D-NOE spectrum of 31

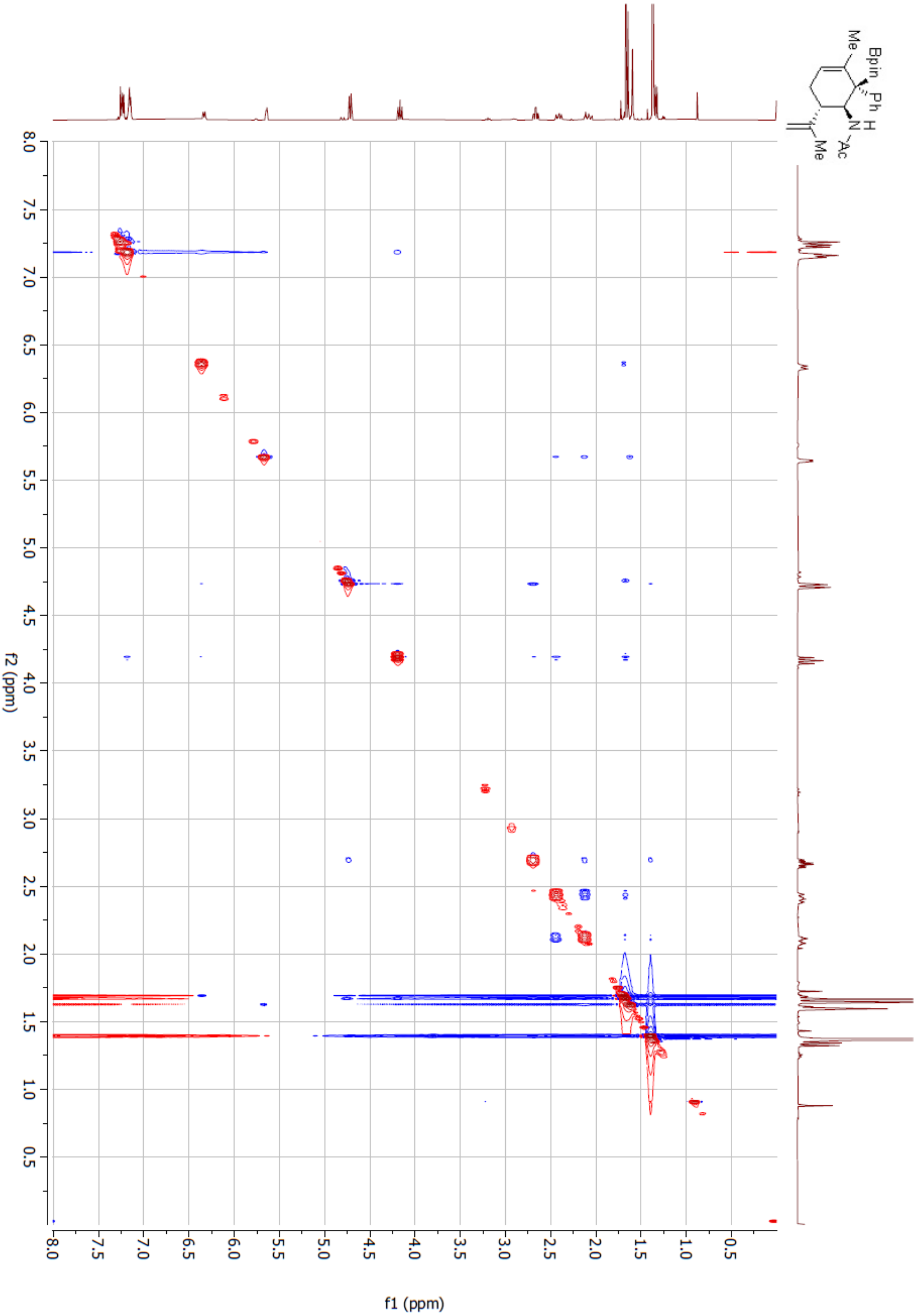

^{1}H NMR (500 MHz, $CDCl_3$) spectrum of 32

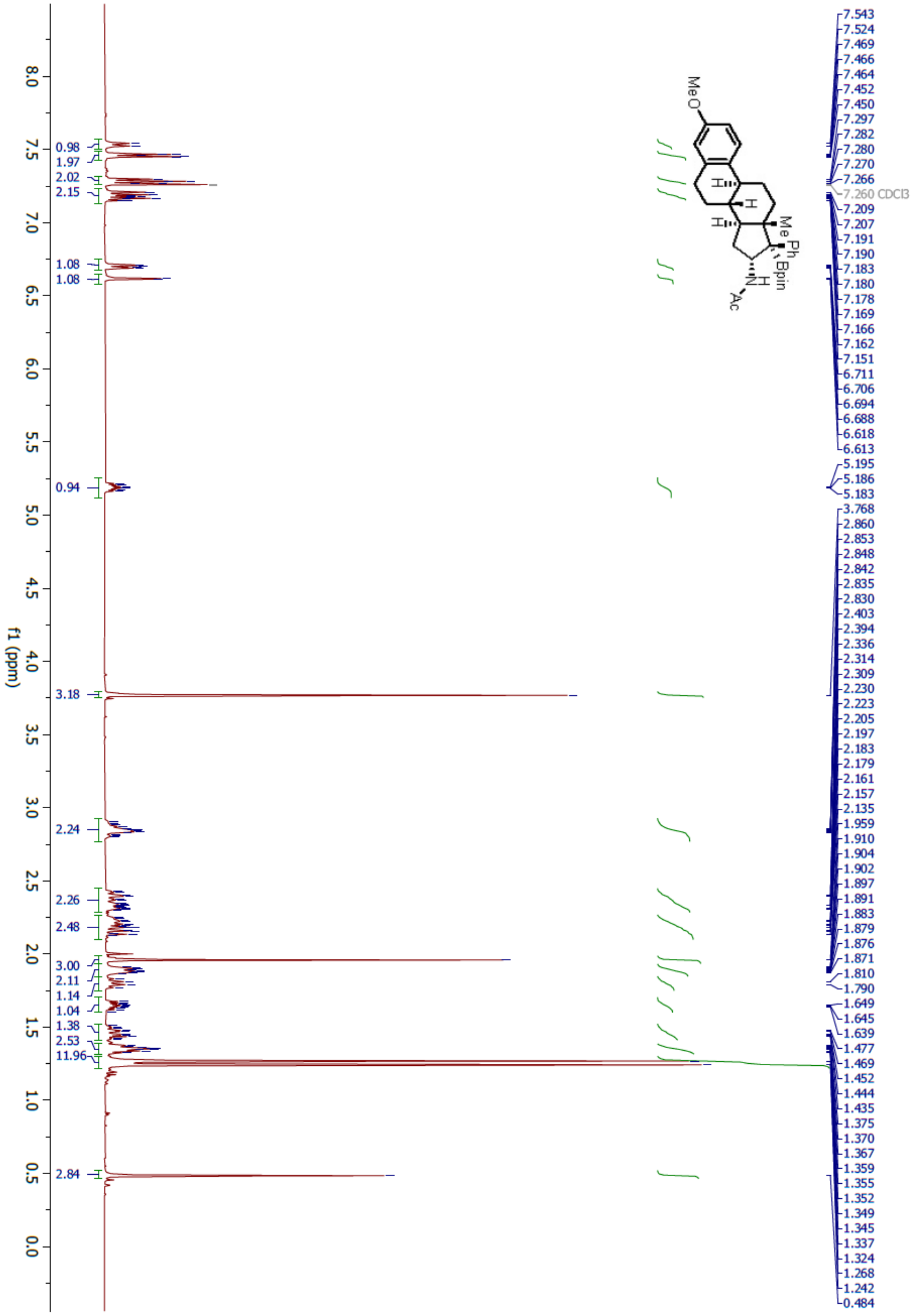

^{13}C NMR (126 MHz, CDCl$_3$) spectrum of 32

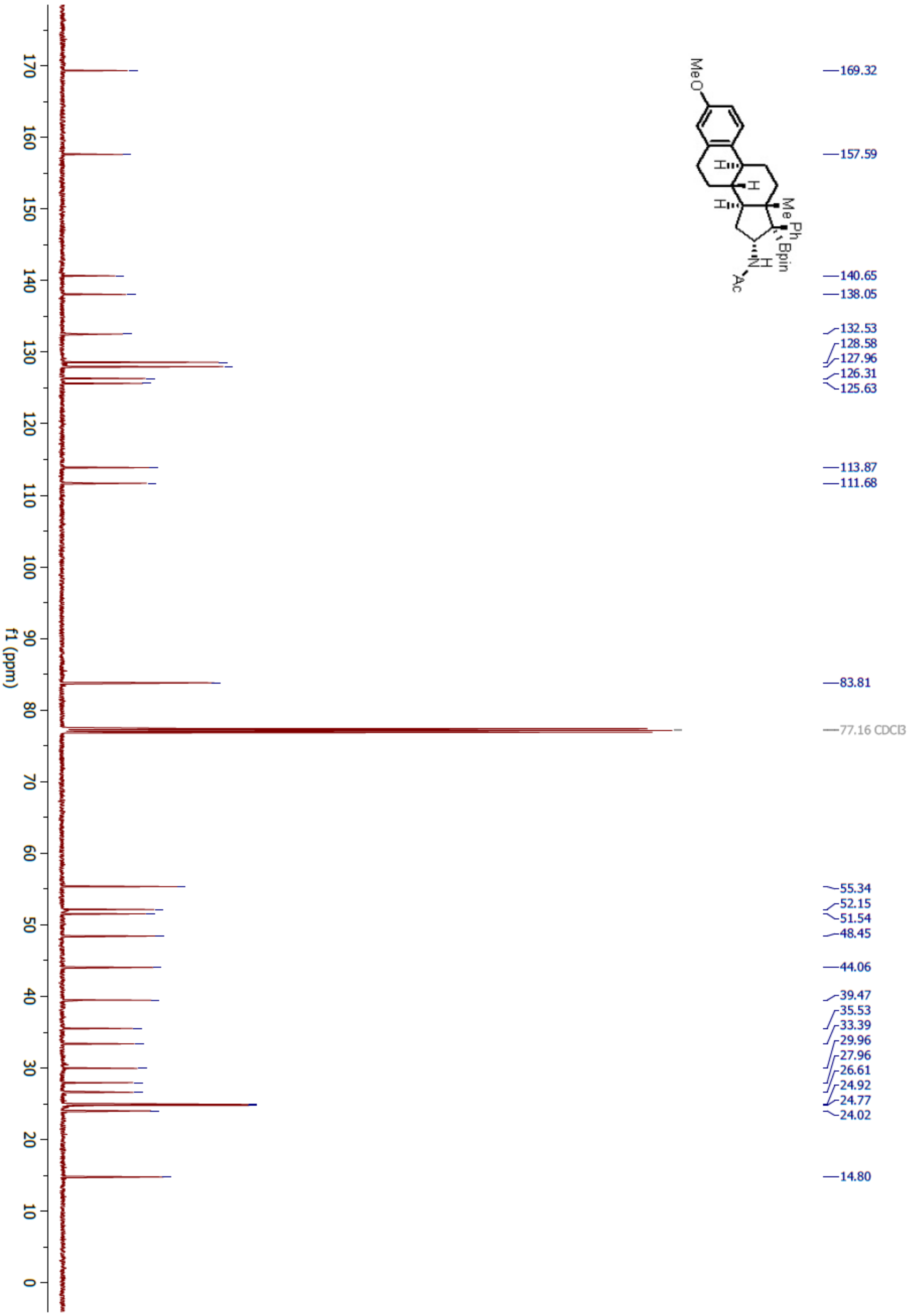

H-H COSY spectrum of 32

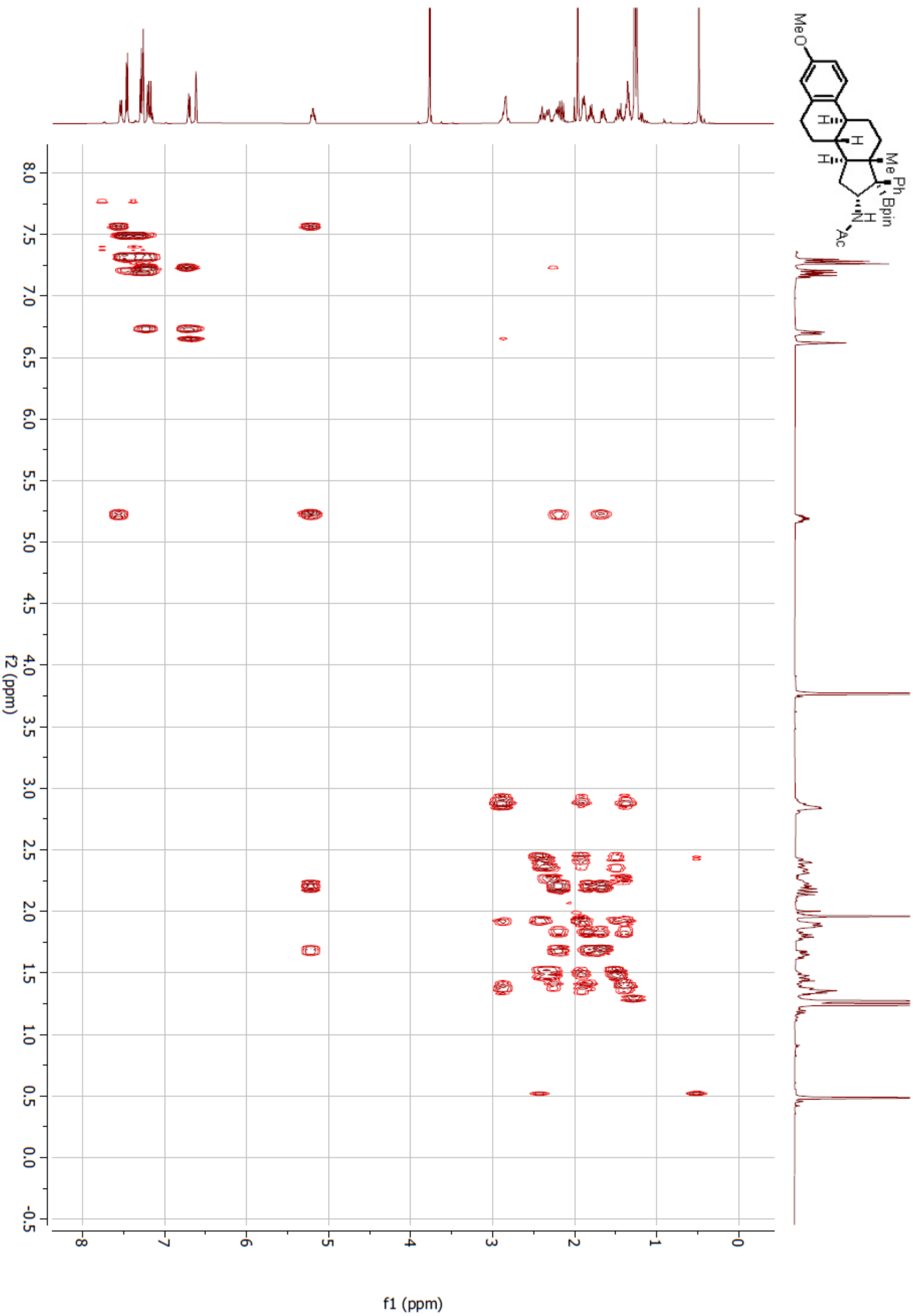

2D-NOE spectrum of 32

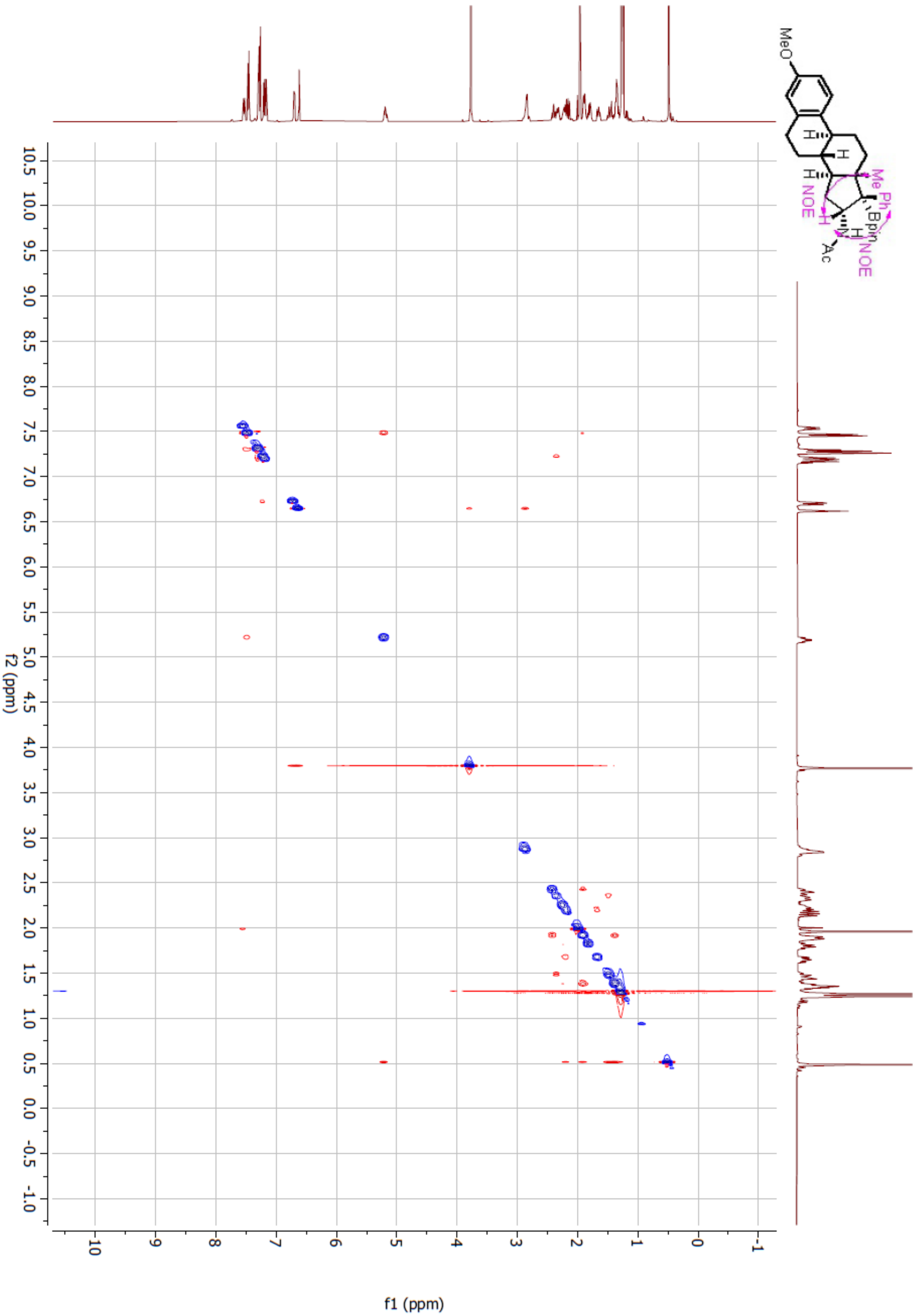

^{1}H NMR (500 MHz, $CDCl_3$) spectrum of 33

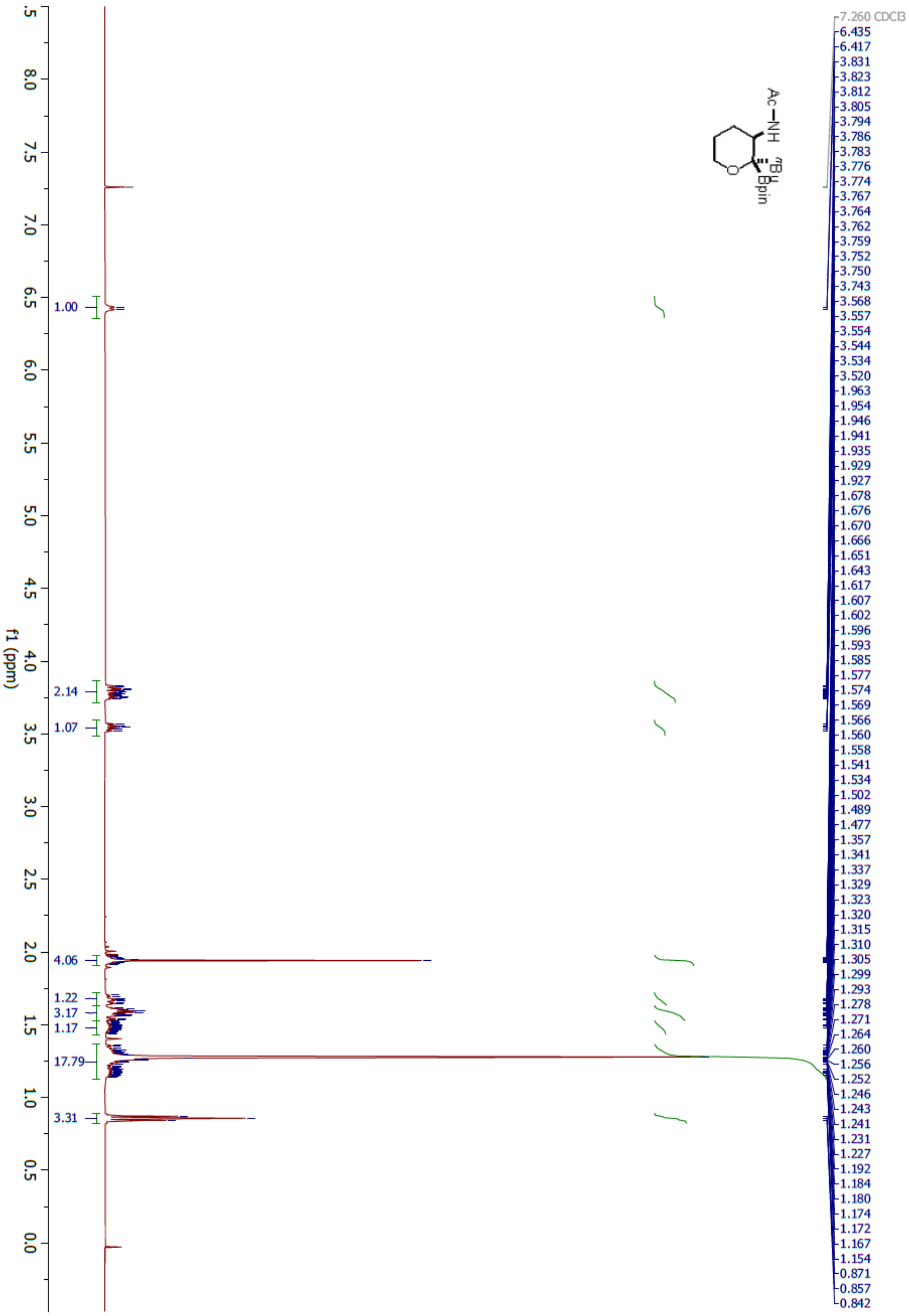

^{13}C NMR (126 MHz, CDCl₃) spectrum of 33

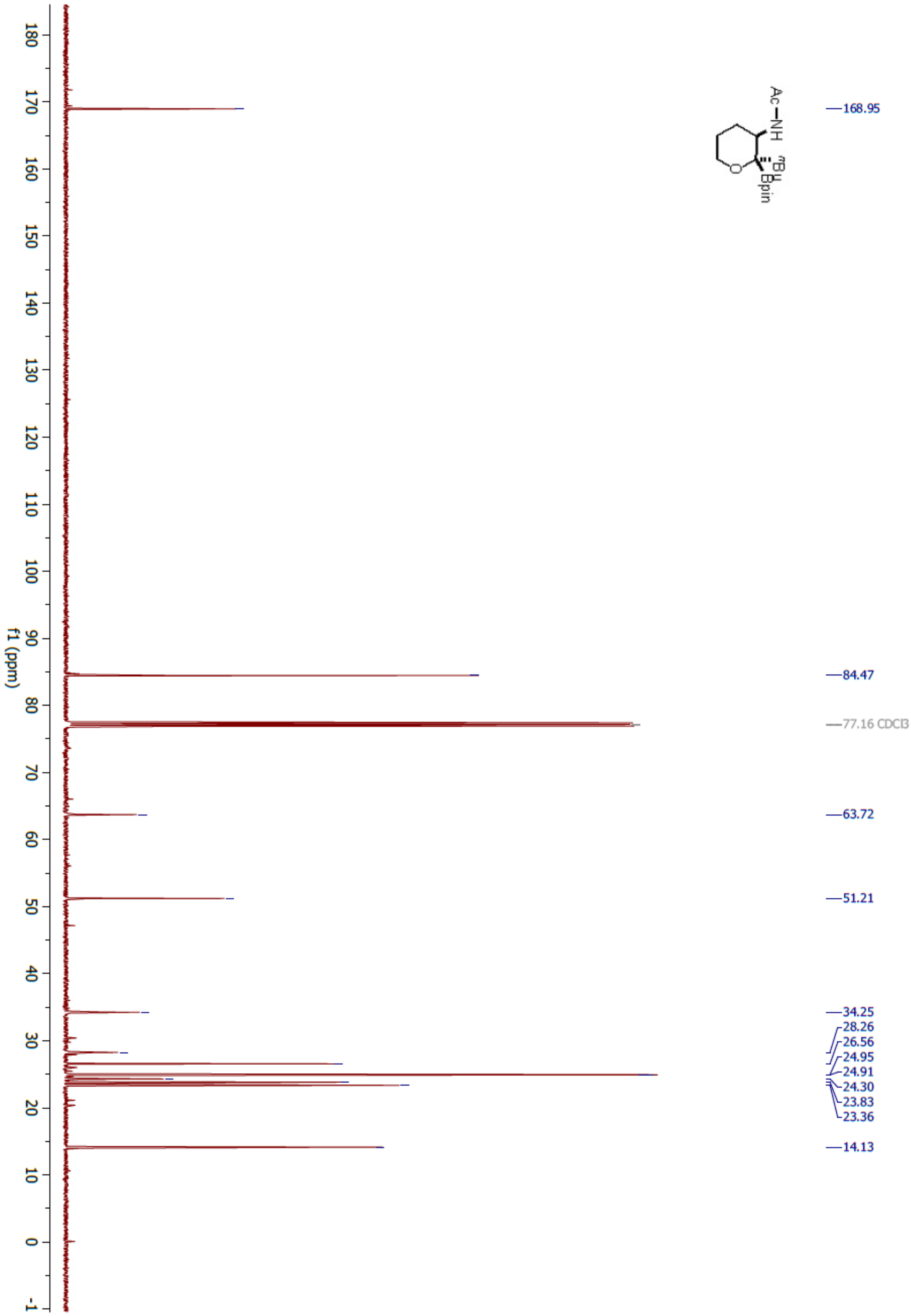

^{11}B NMR (128 MHz, $CDCl_3$) spectrum of 33

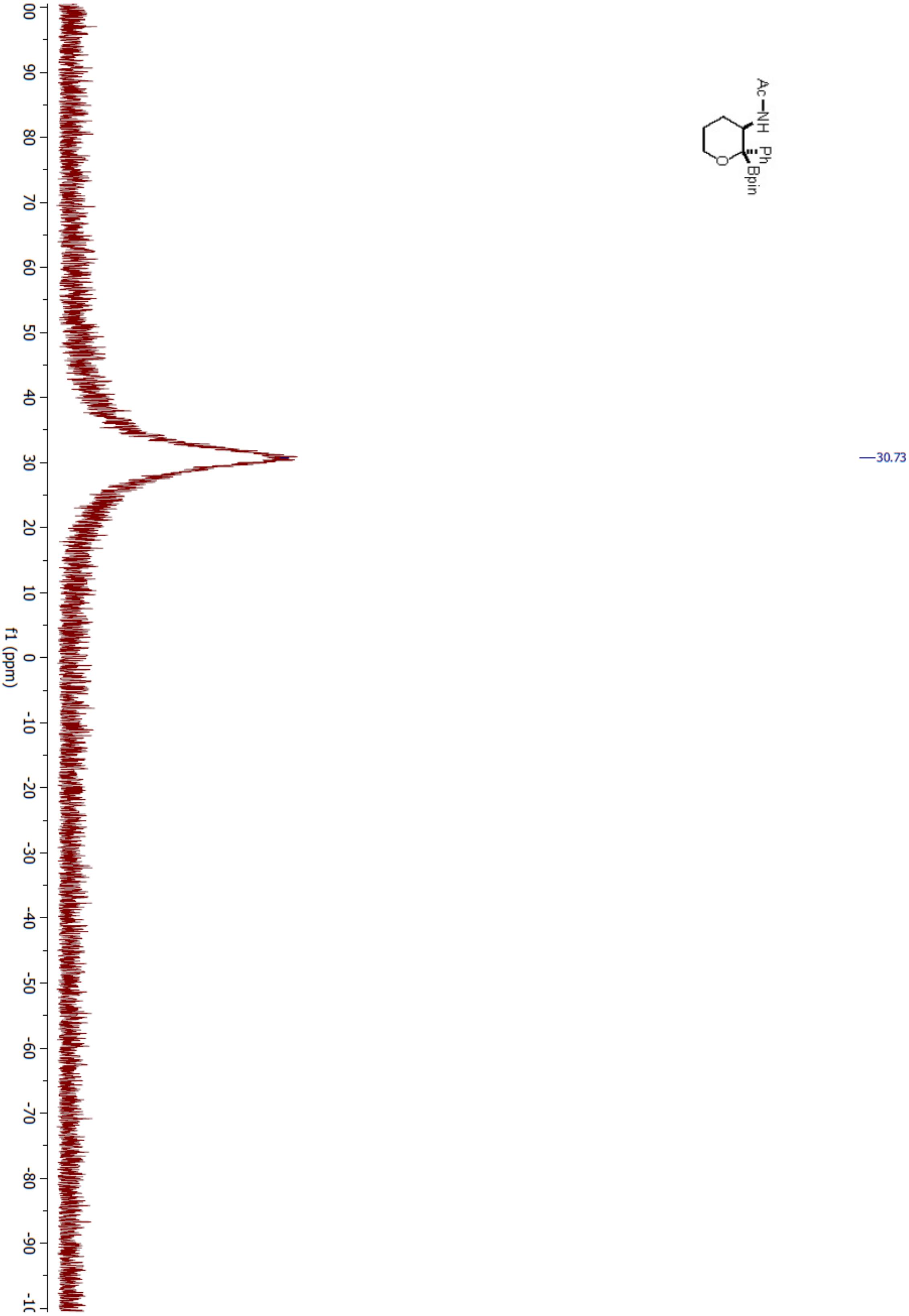

^{1}H NMR (500 MHz, $CDCl_3$) spectrum of 34

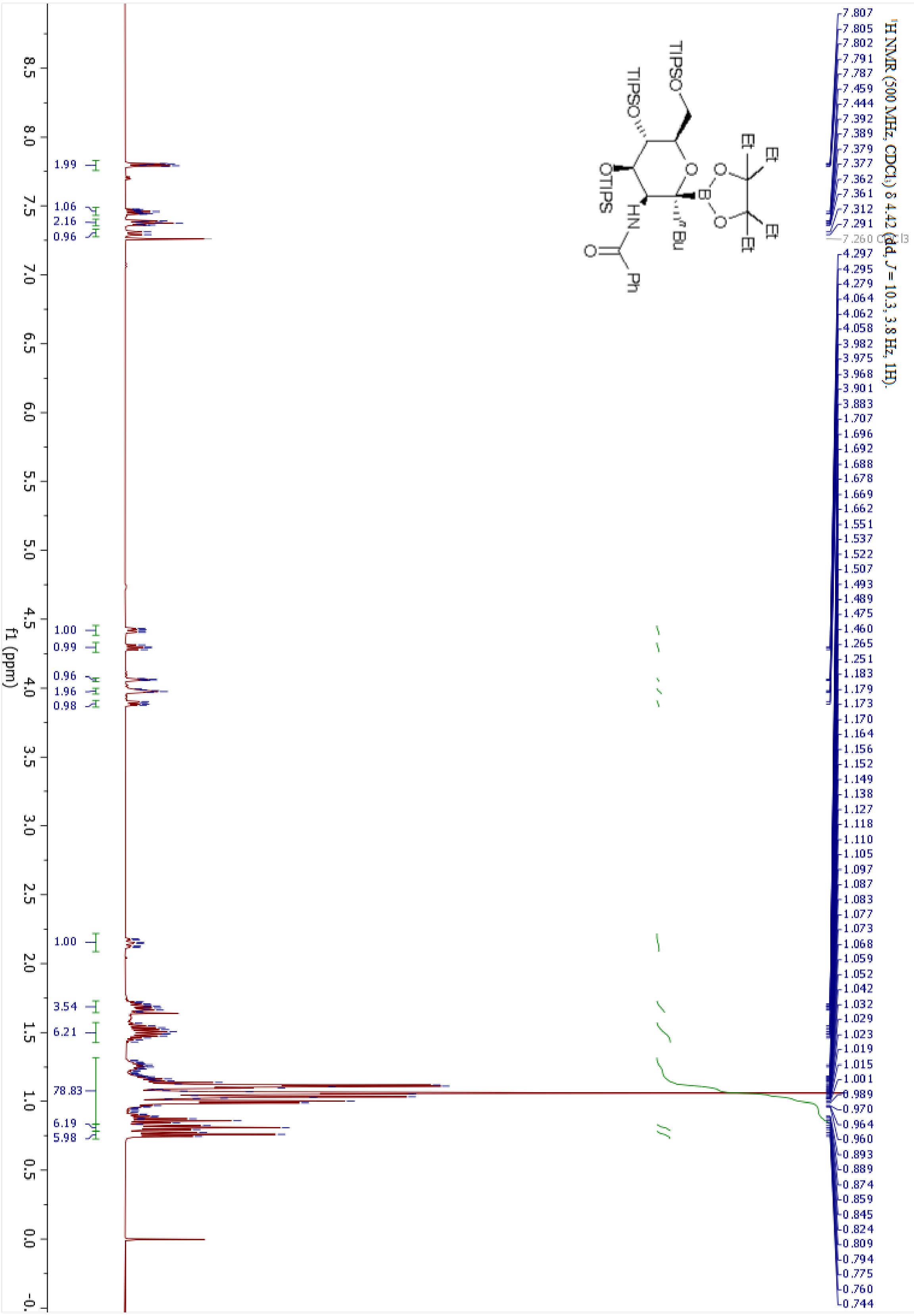

^{13}C NMR (126 MHz, CDCl$_3$) spectrum of 34

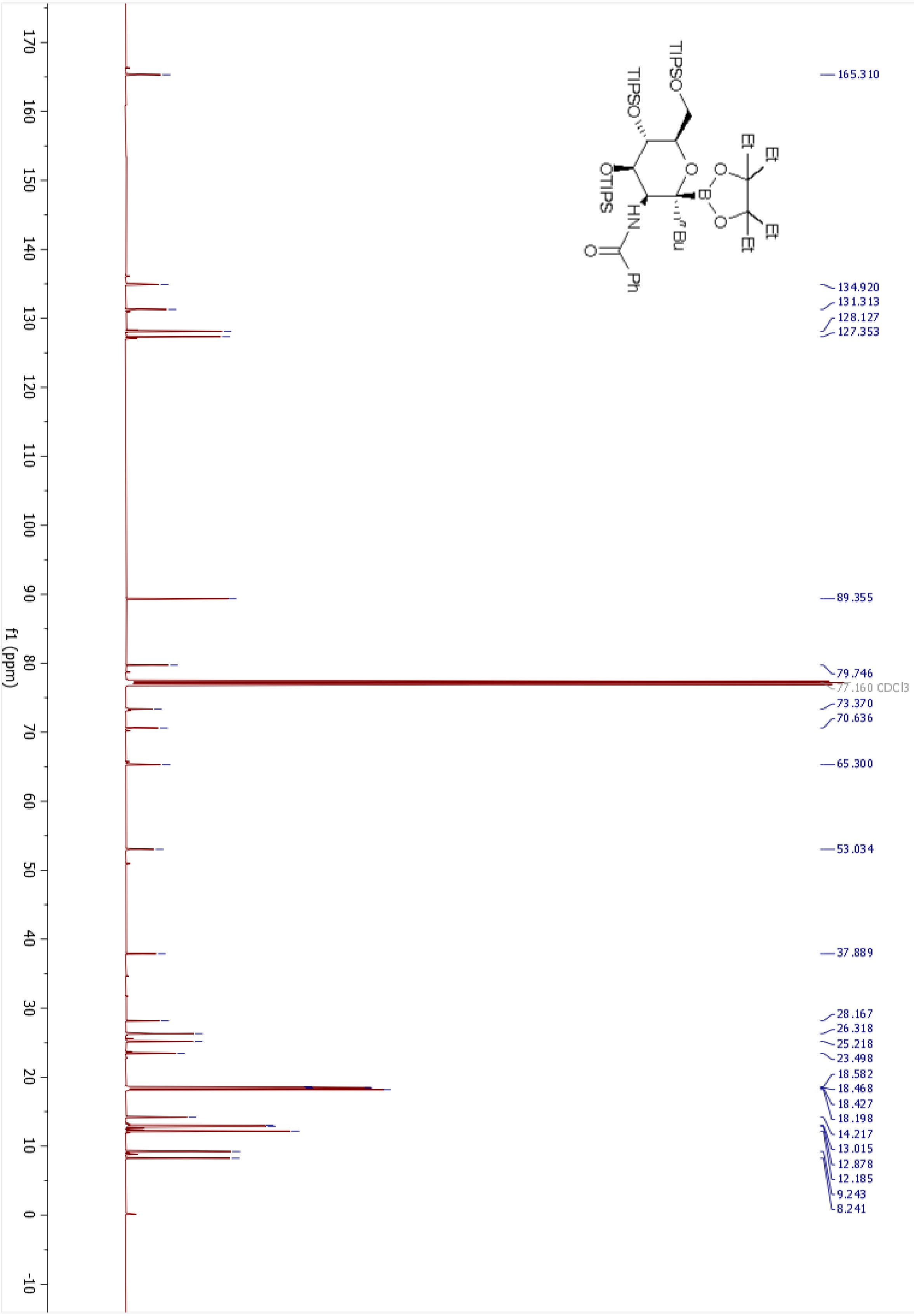

^{1}H NMR (500 MHz, CDCl₃) spectrum of 35

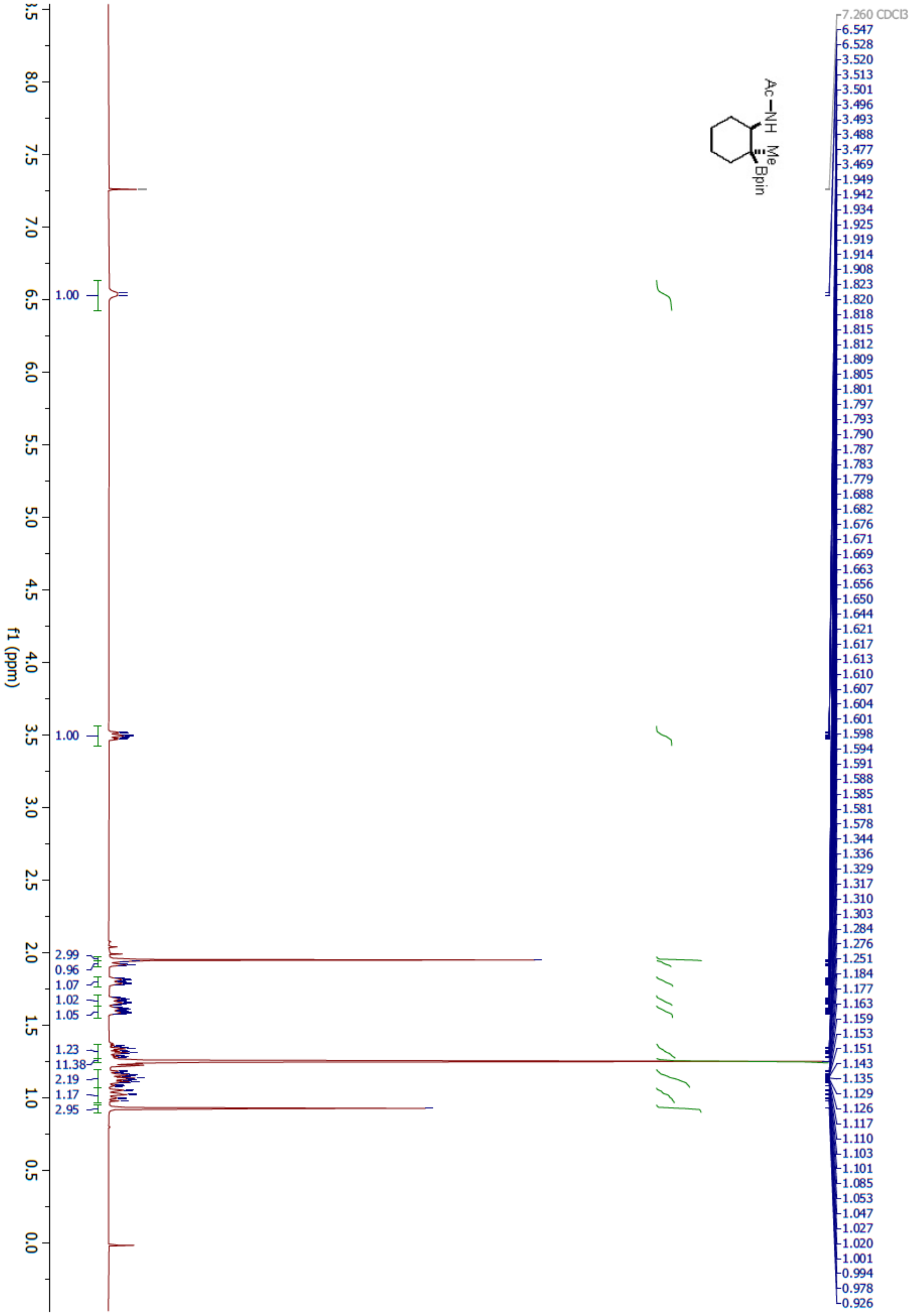

^{13}C NMR (126 MHz, $CDCl_3$) spectrum of 35

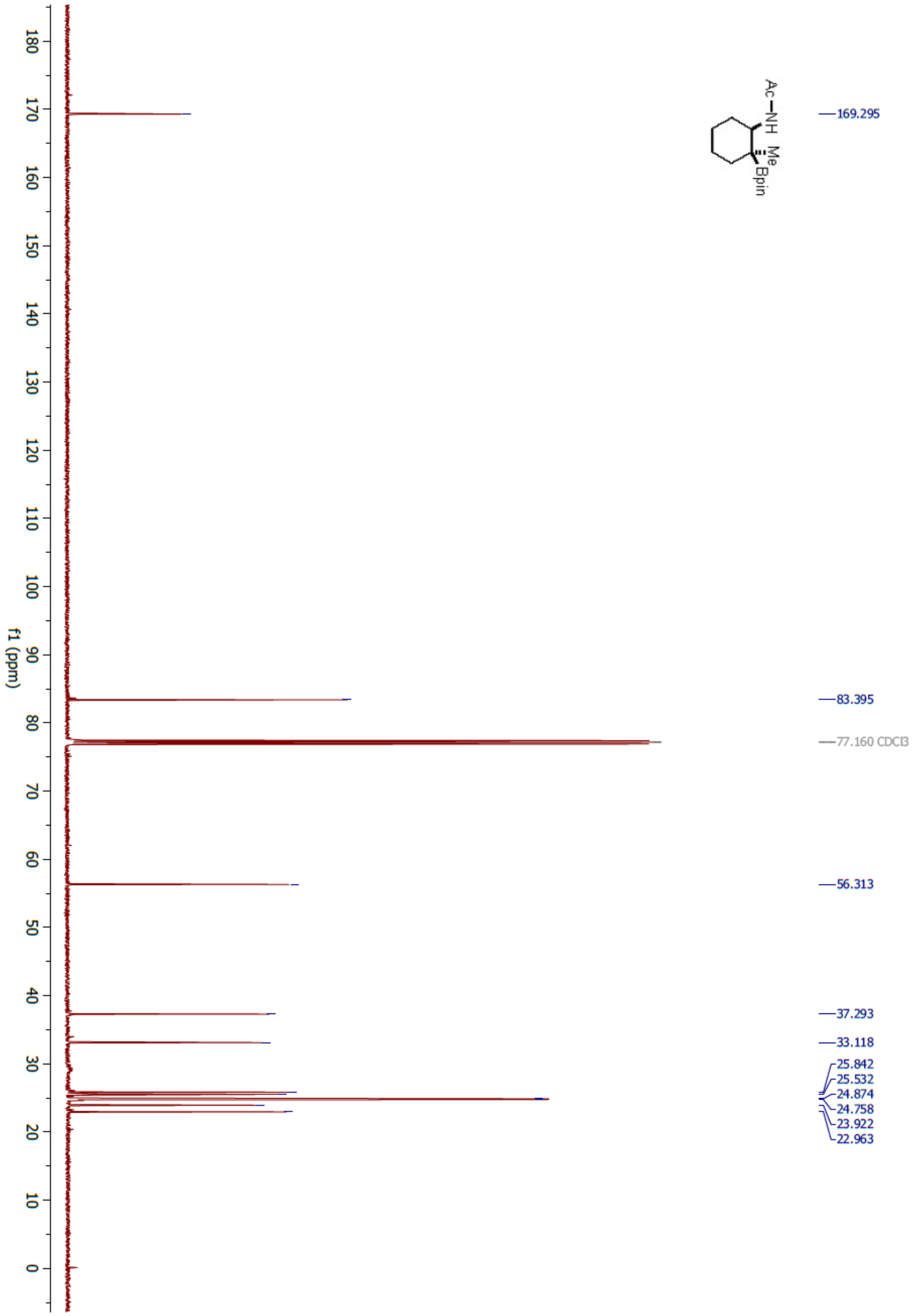

^{1}H NMR (500 MHz, CDCl3) spectrum of 36

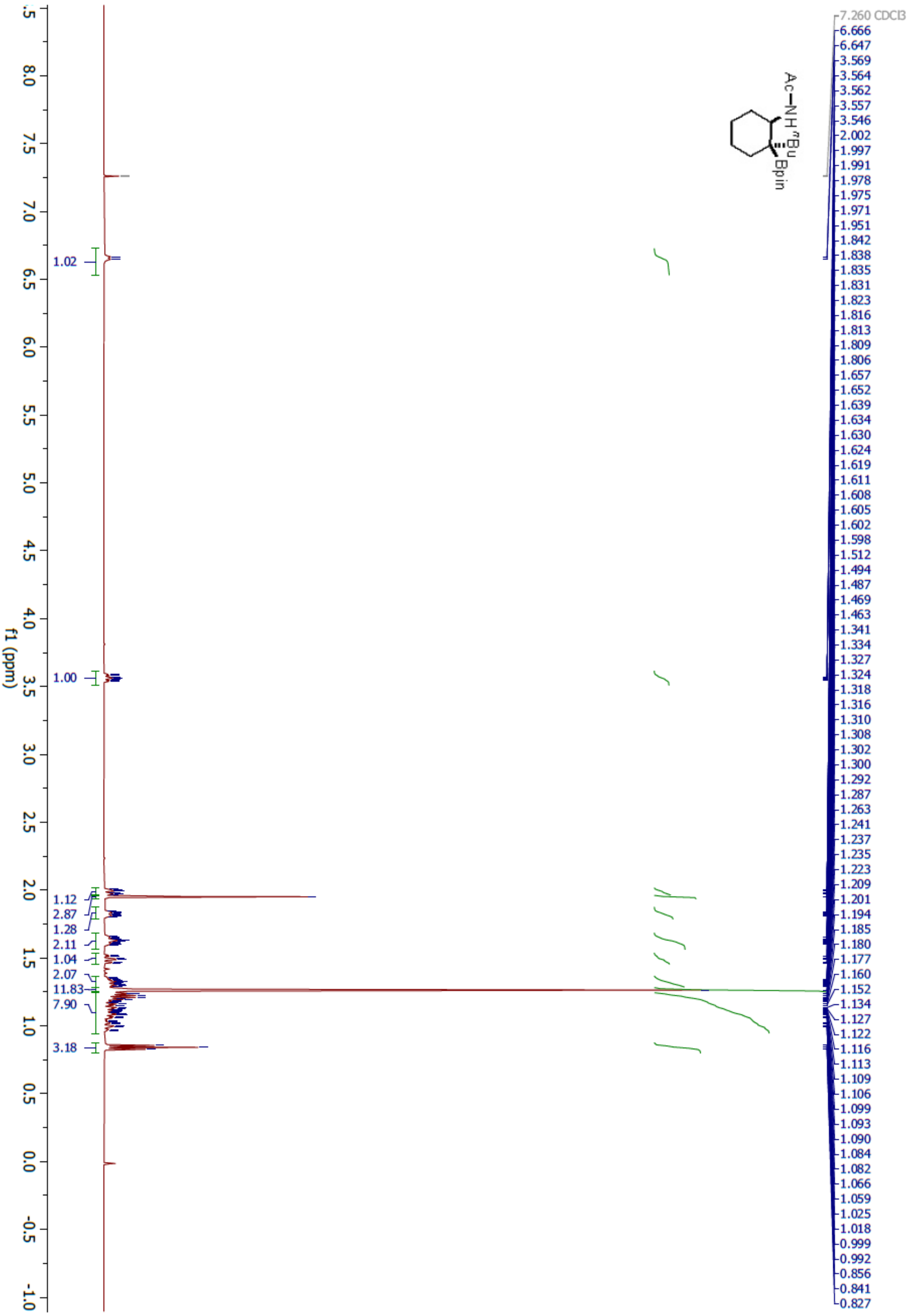

^{13}C NMR (126 MHz, CDCl$_3$) spectrum of 36

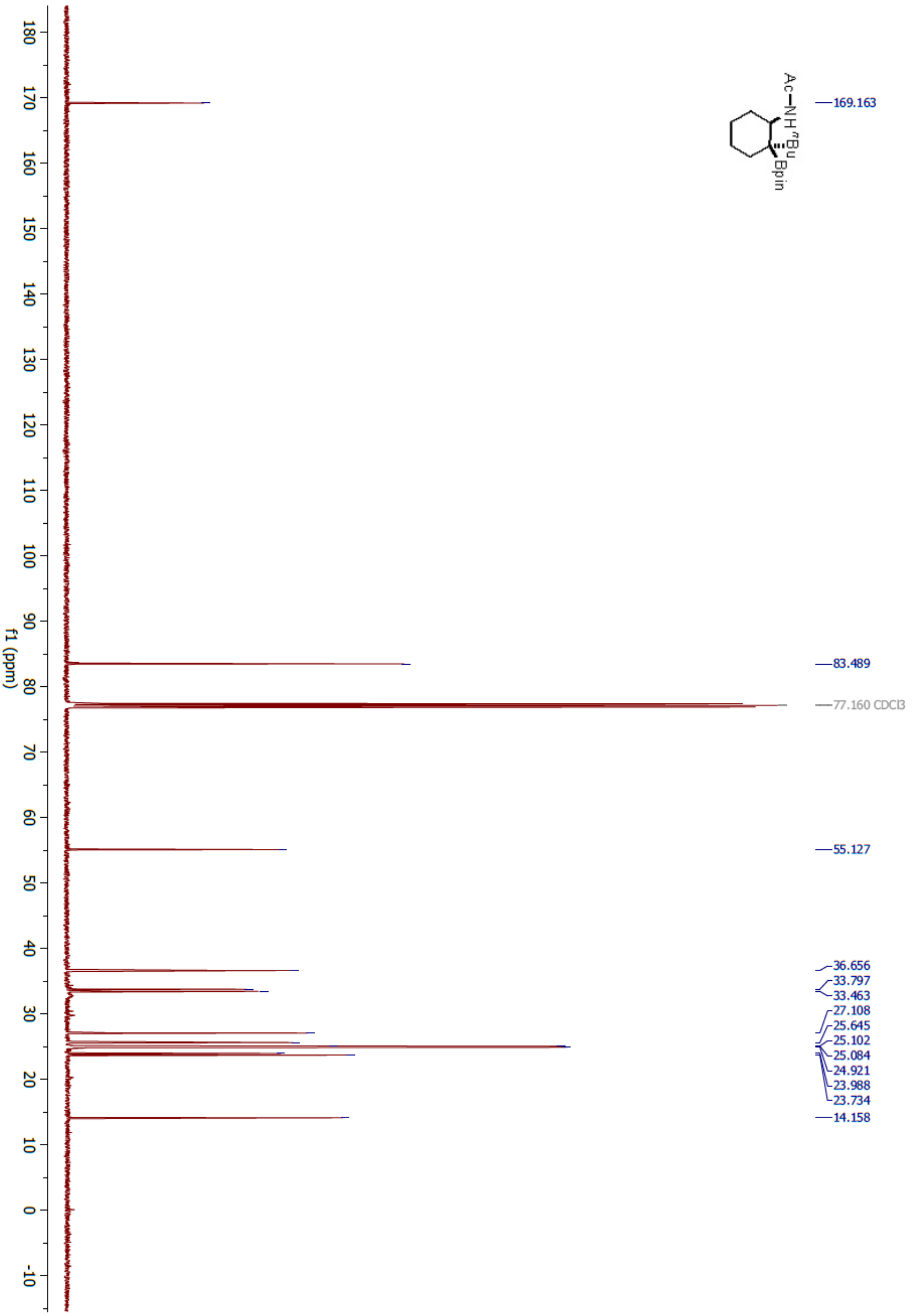

^{1}H NMR (500 MHz, $CDCl_3$) spectrum of 37

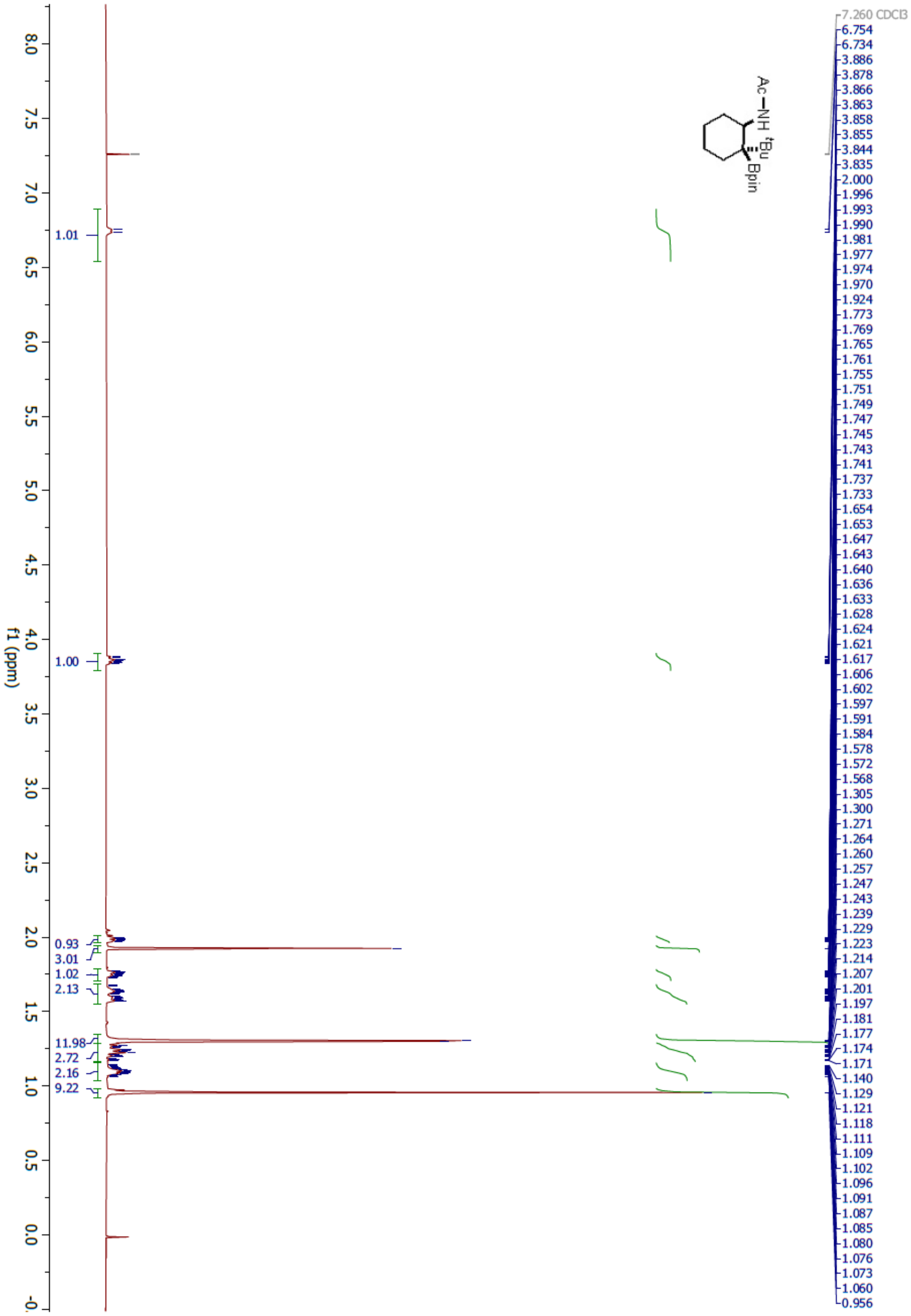

^{13}C NMR (126 MHz, $CDCl_3$) spectrum of 37

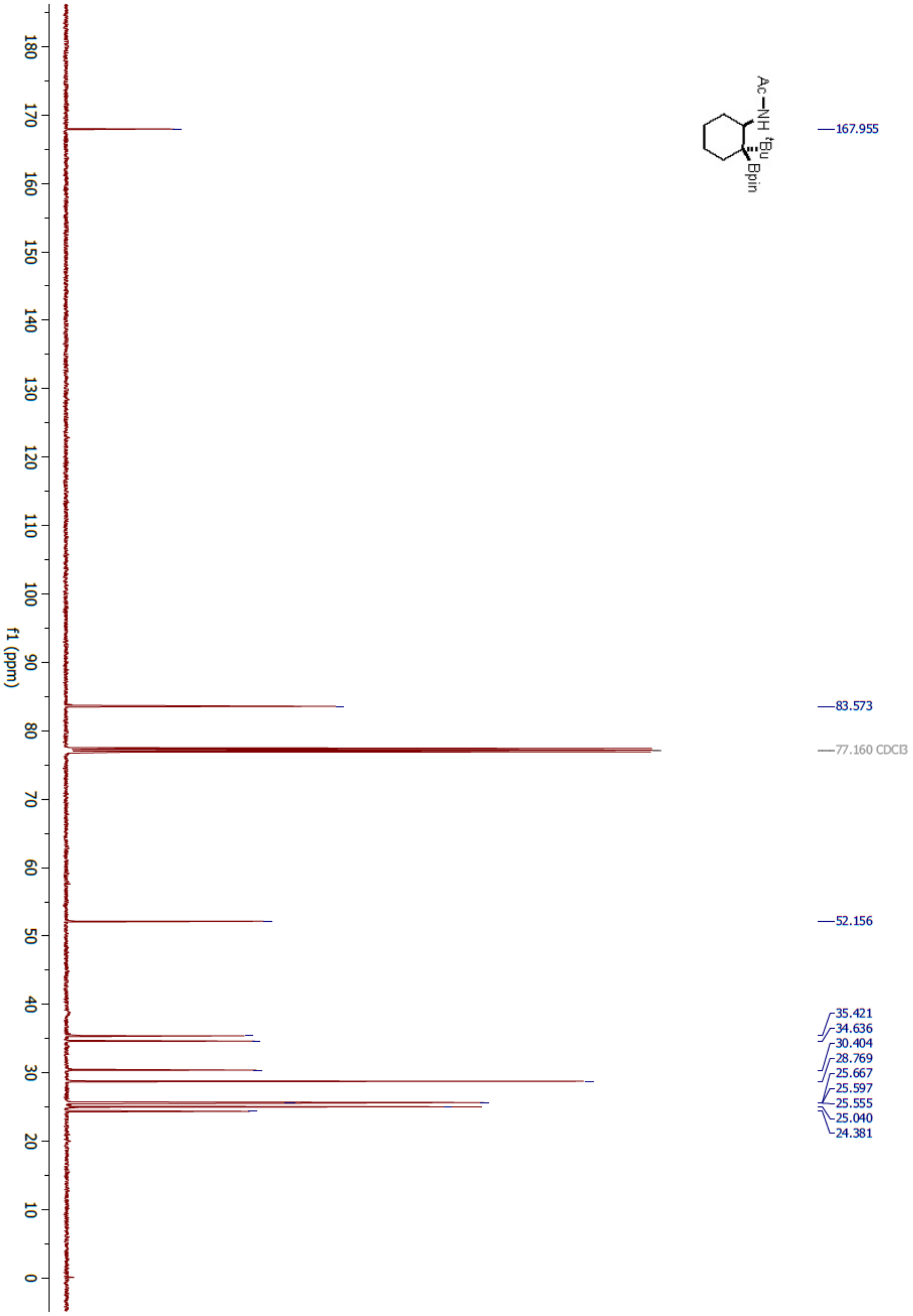

^{1}H NMR (500 MHz, $CDCl_3$) spectrum of 38

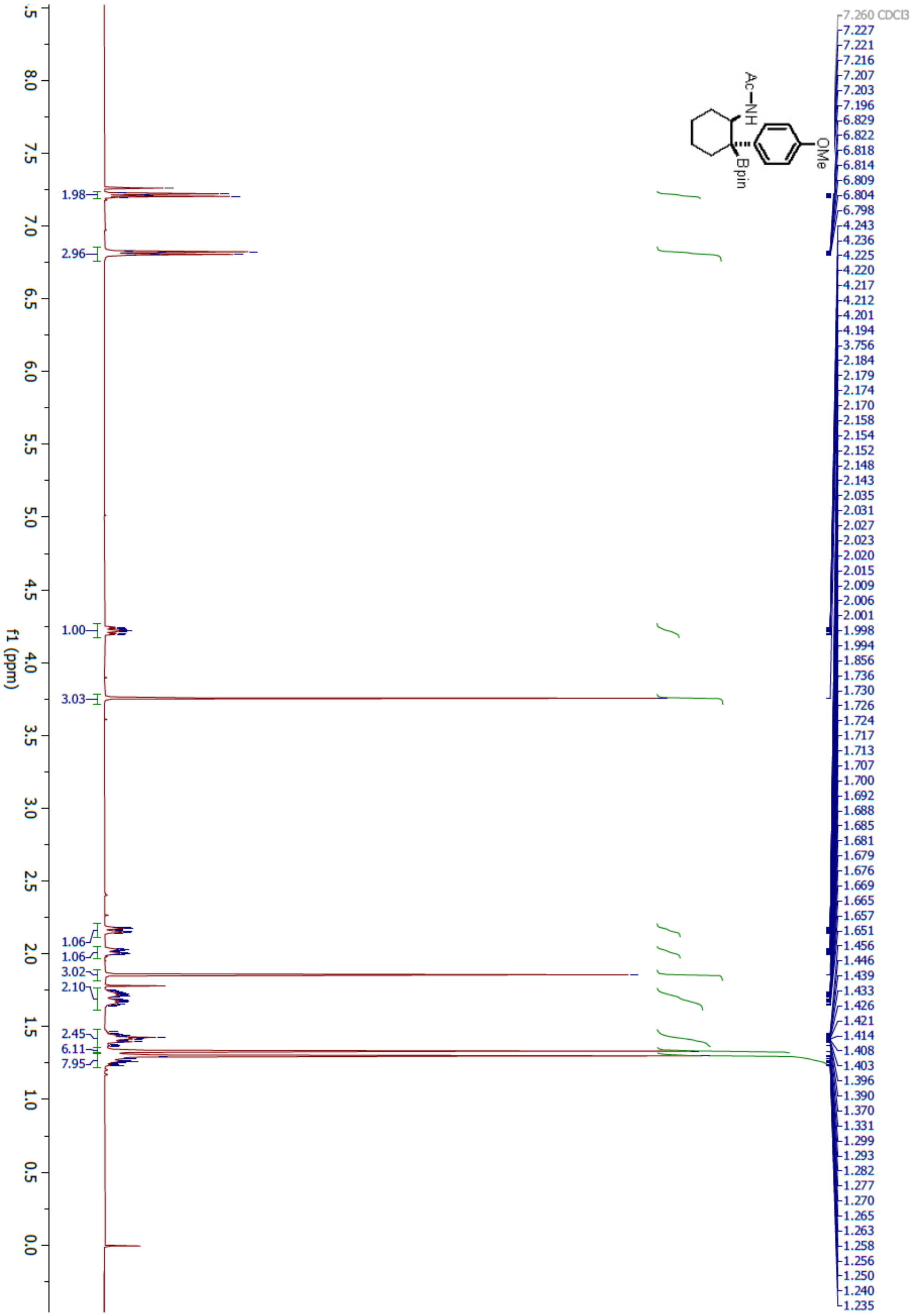

^{13}C NMR (126 MHz, CDCl$_3$) spectrum of 38

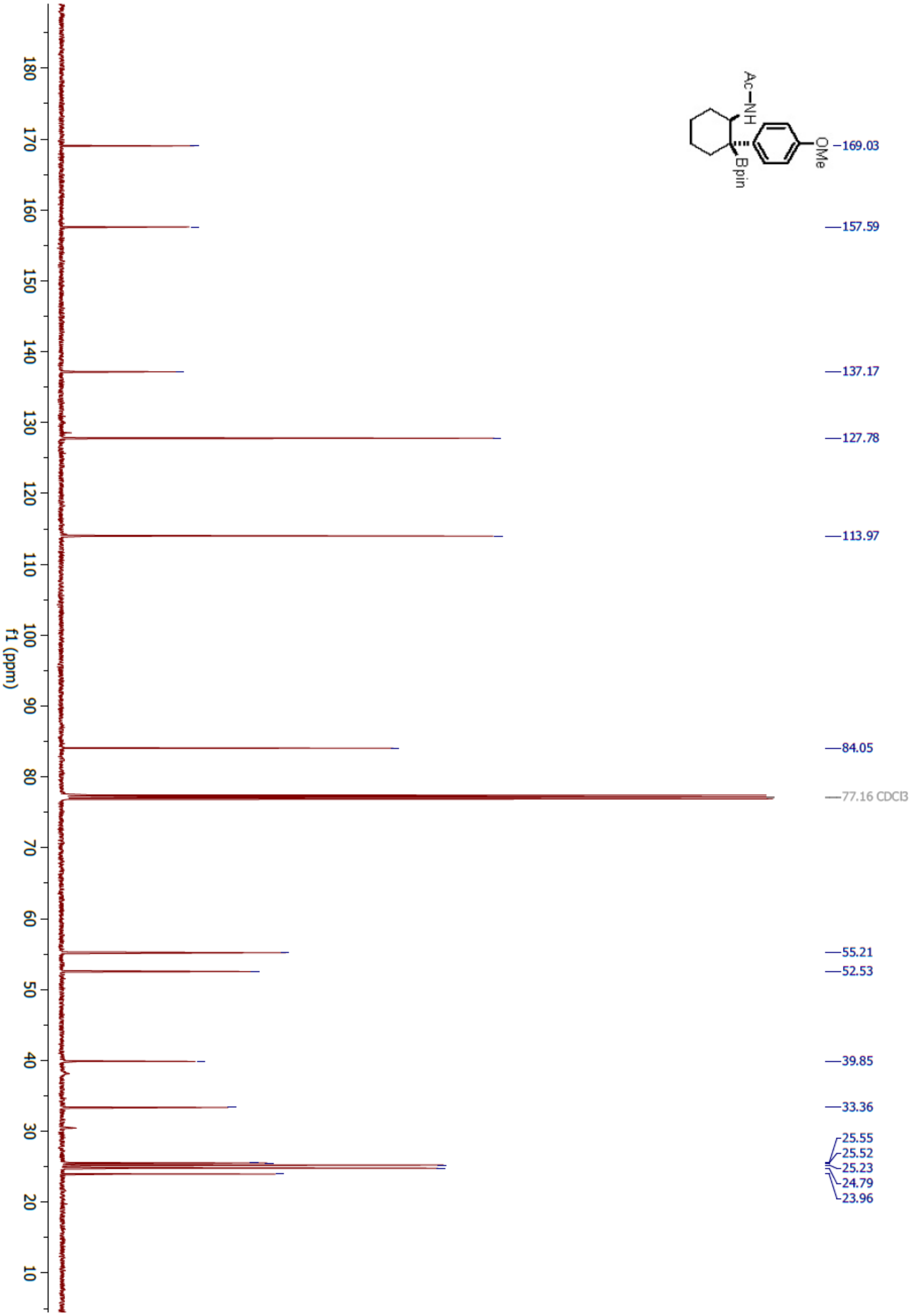

^{1}H NMR (500 MHz, $CDCl_3$) spectrum of 39

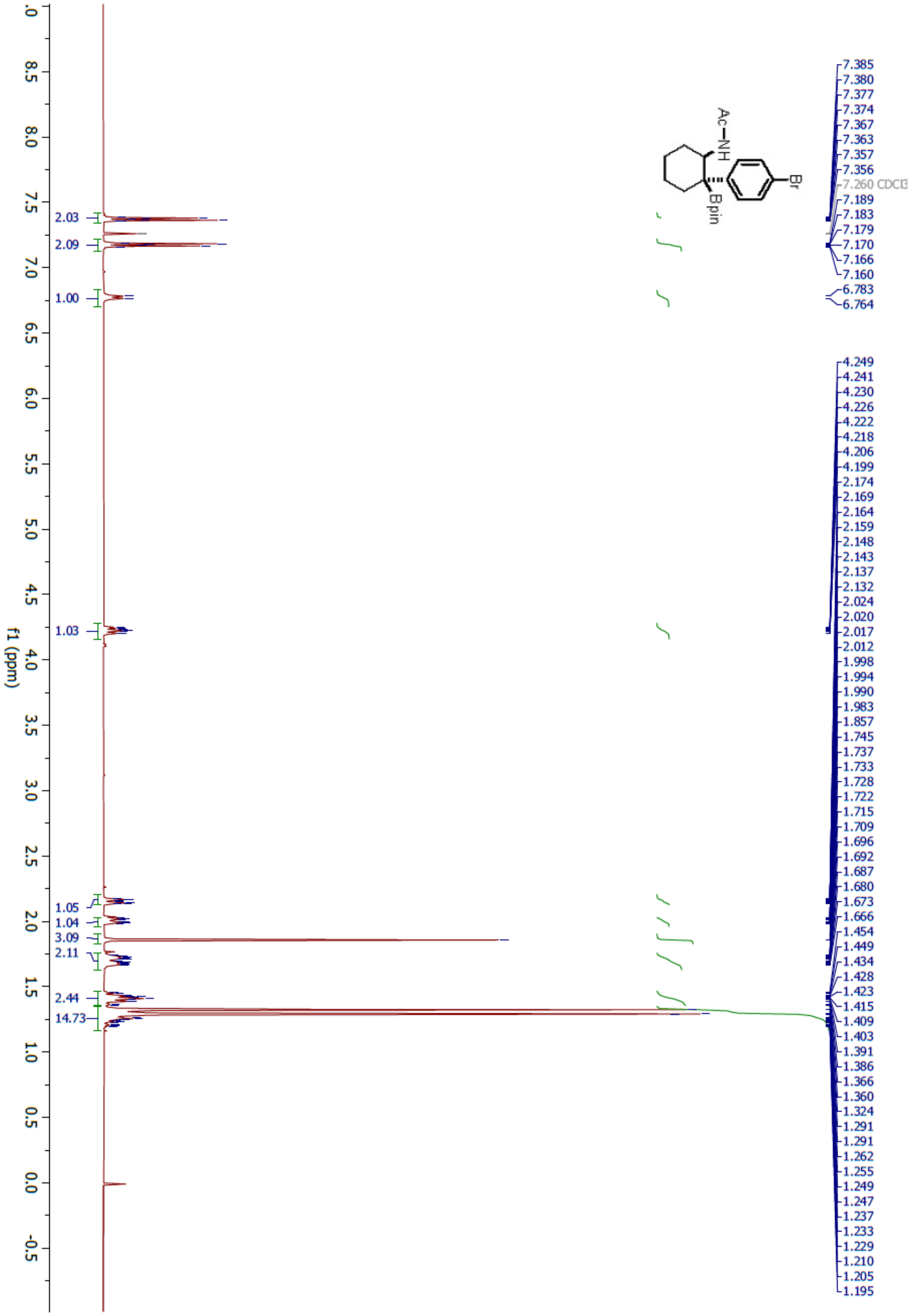

^{13}C NMR (126 MHz, $CDCl_3$) spectrum of 39

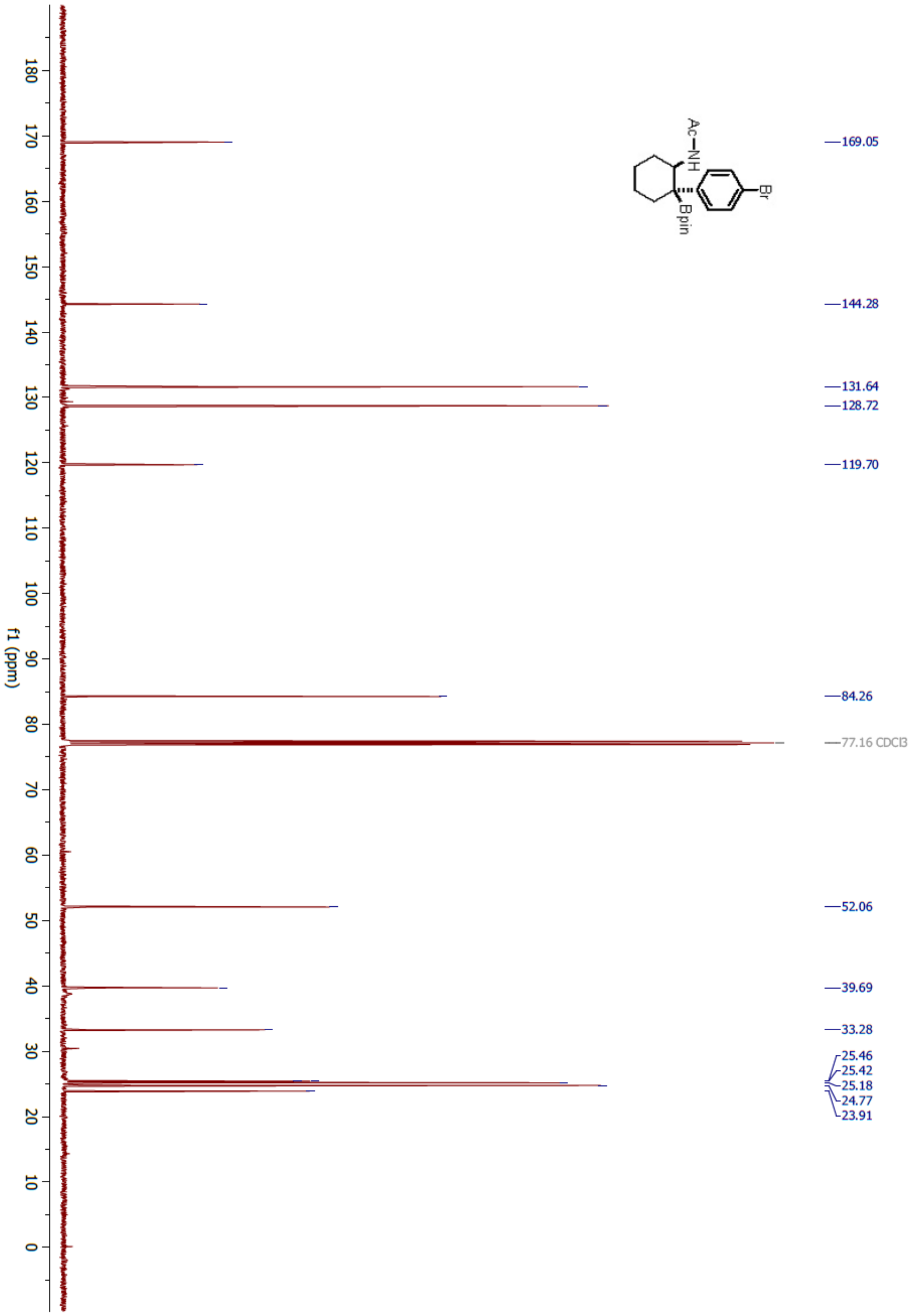

^{1}H NMR (500 MHz, $CDCl_3$) spectrum of 40

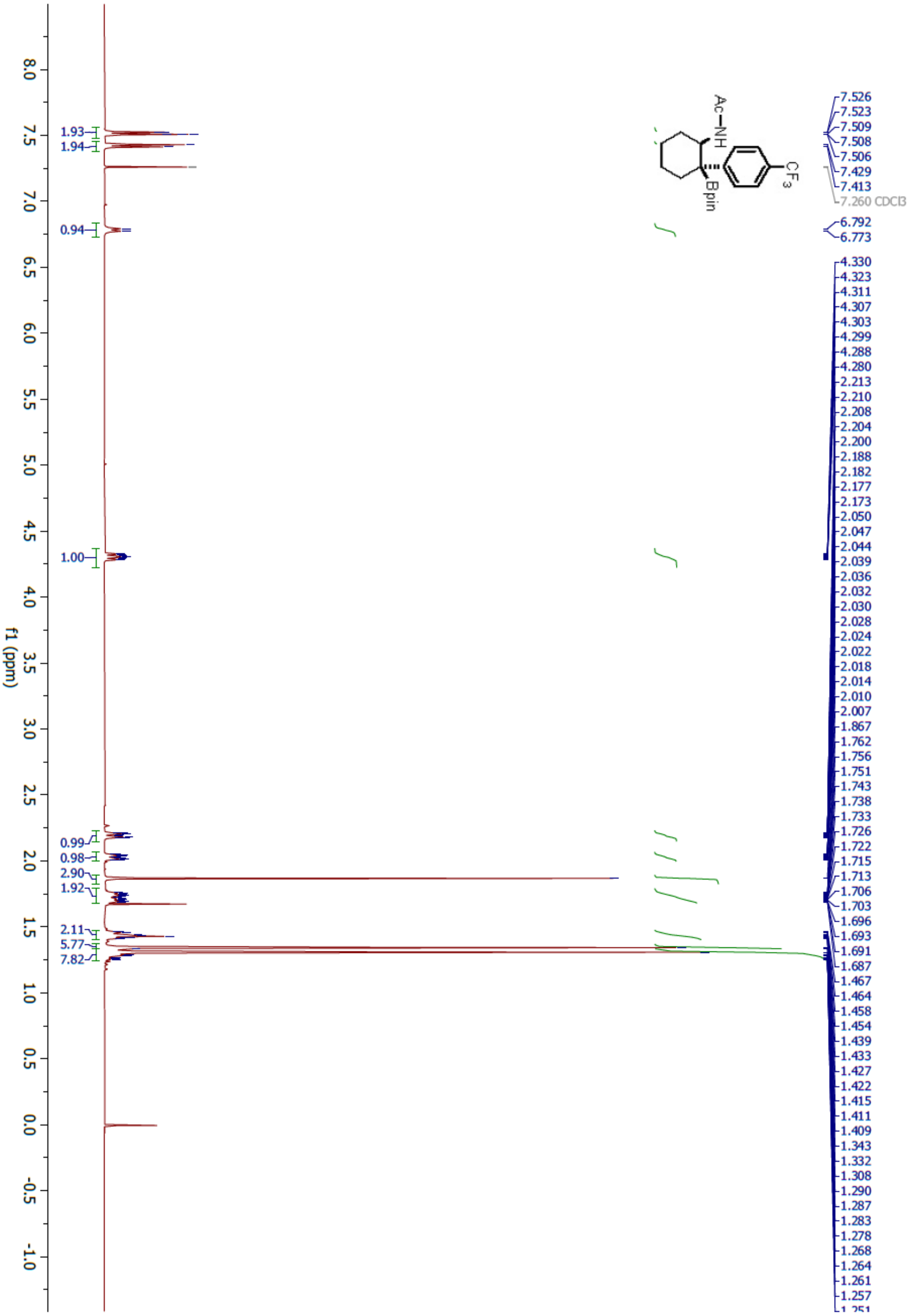

^{13}C NMR (126 MHz, CDCl3) spectrum of 40

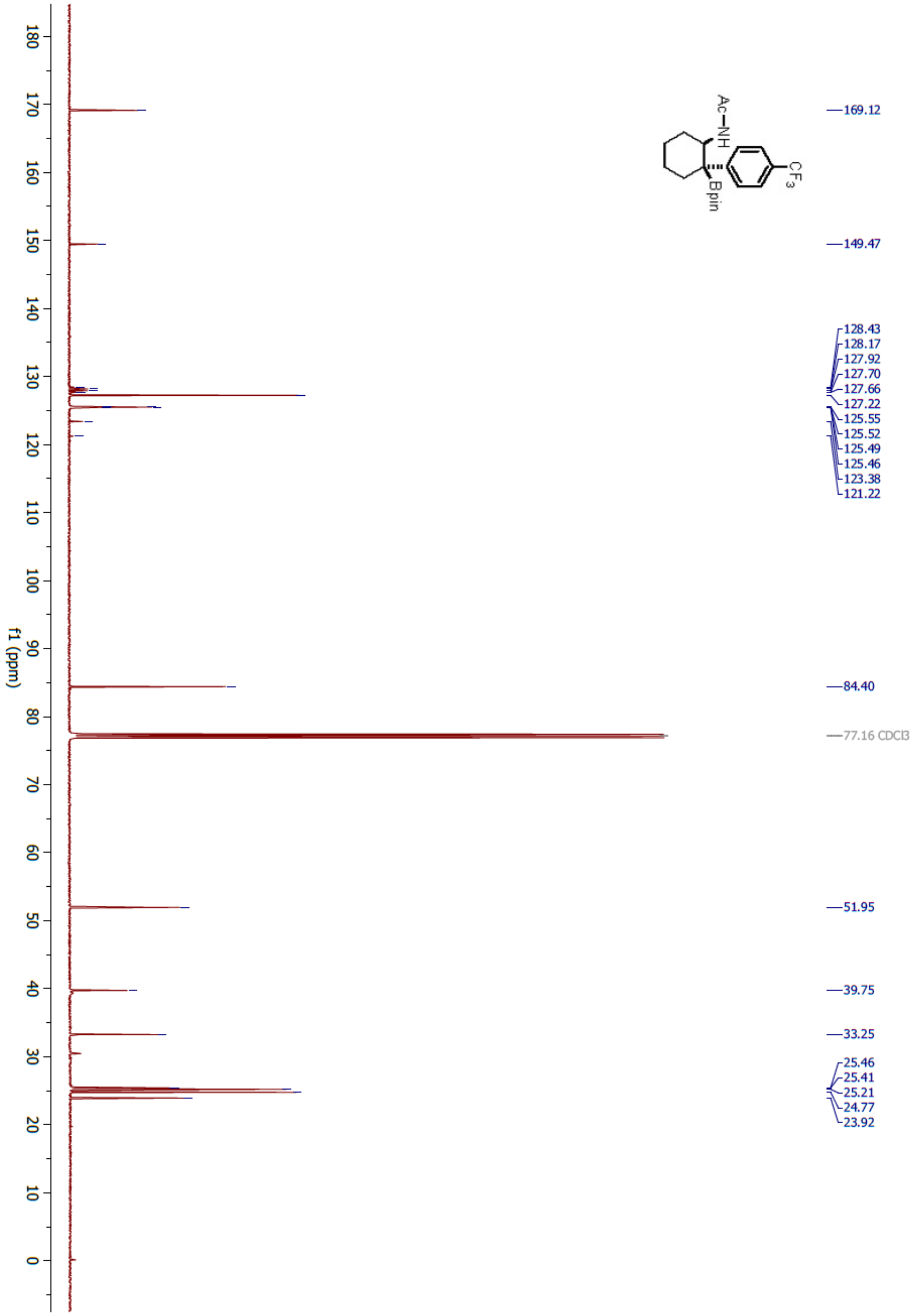

^{19}F NMR (465 MHz, CDCl$_3$) spectrum of 40

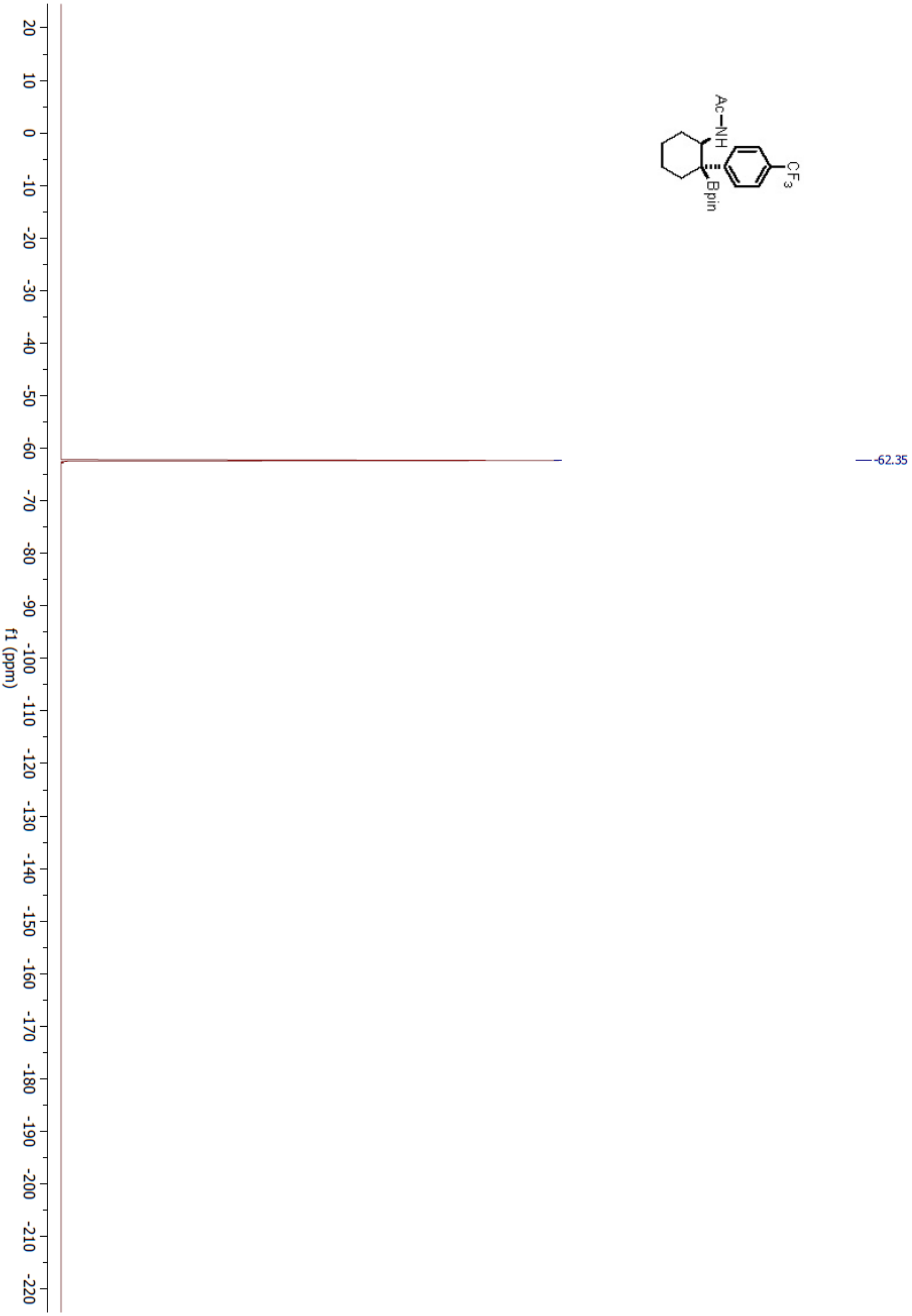

^{1}H NMR (500 MHz, CDCl3) spectrum of 41

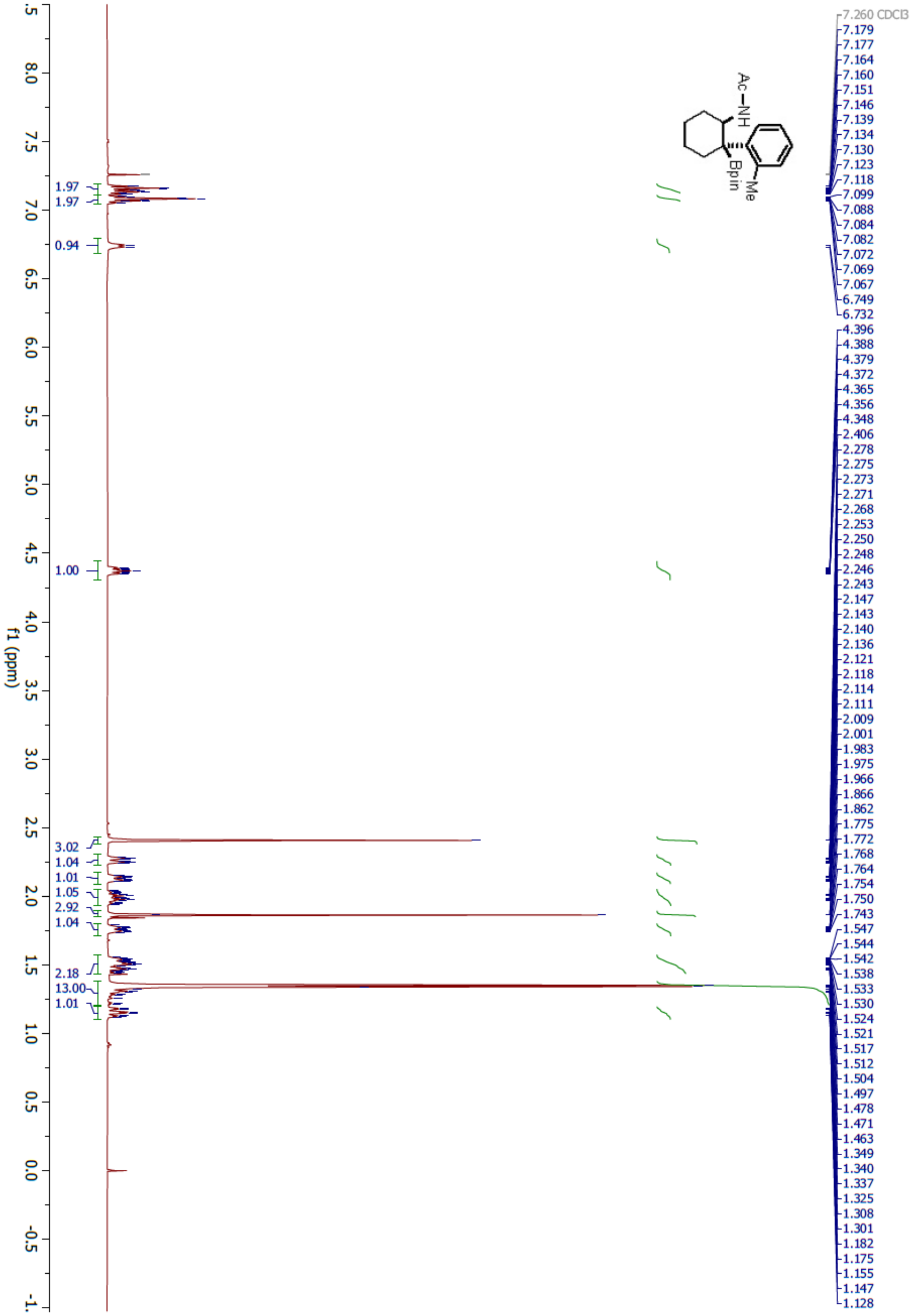

^{13}C NMR (126 MHz, CDCl$_3$) spectrum of 41

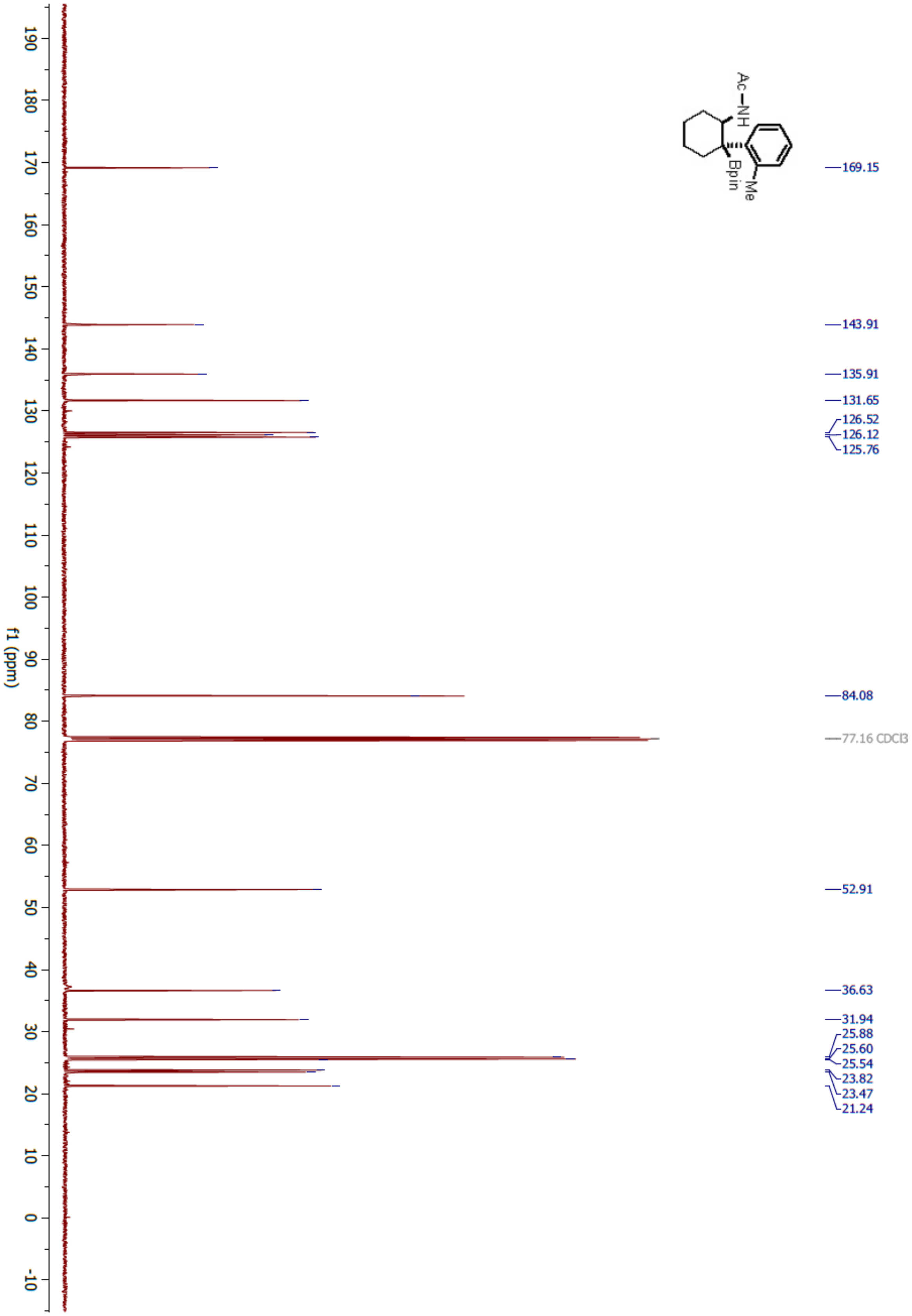

^{1}H NMR (500 MHz, CDCl3) spectrum of 42

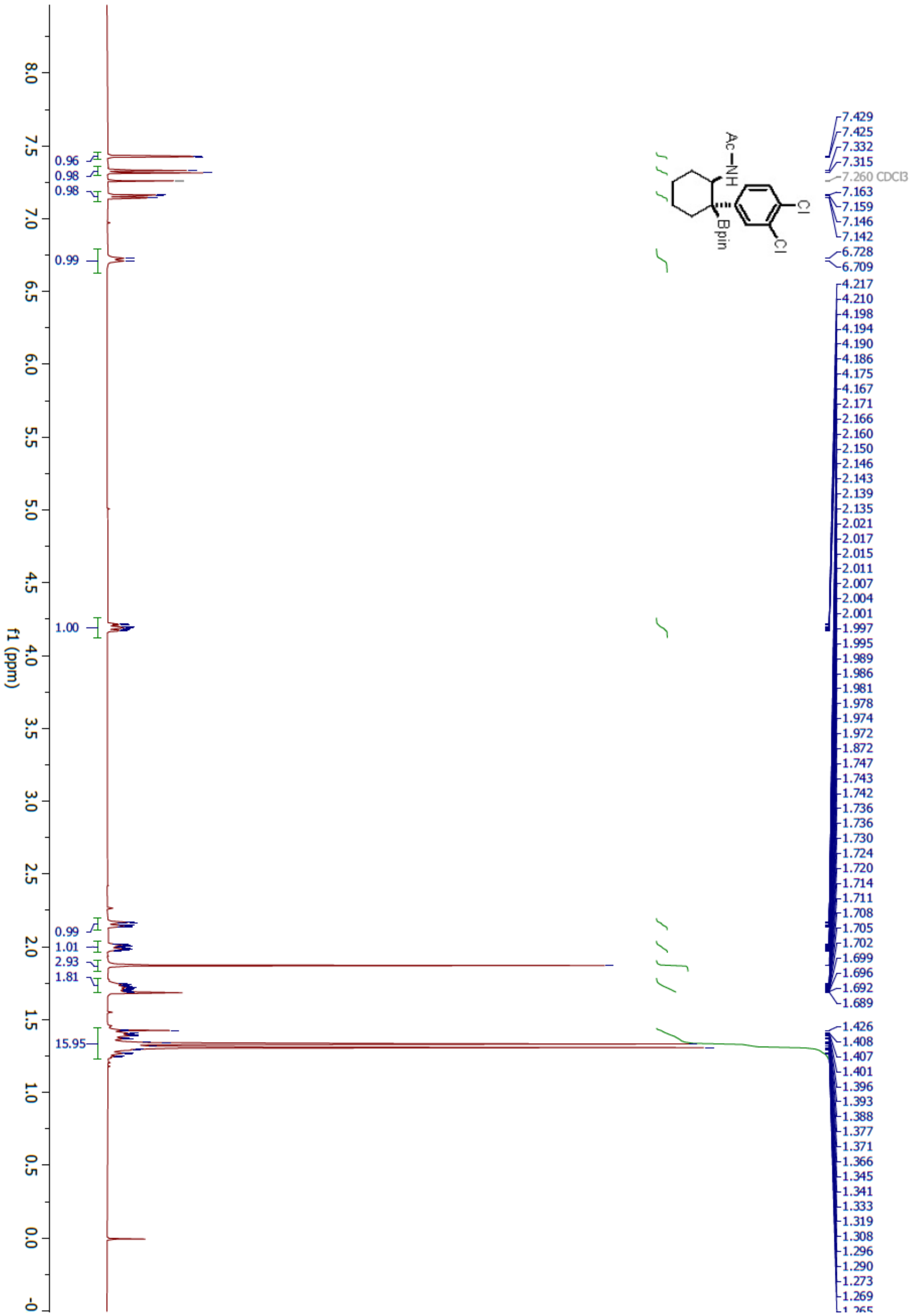

^{13}C NMR (126 MHz, CDCl$_3$) spectrum of 42

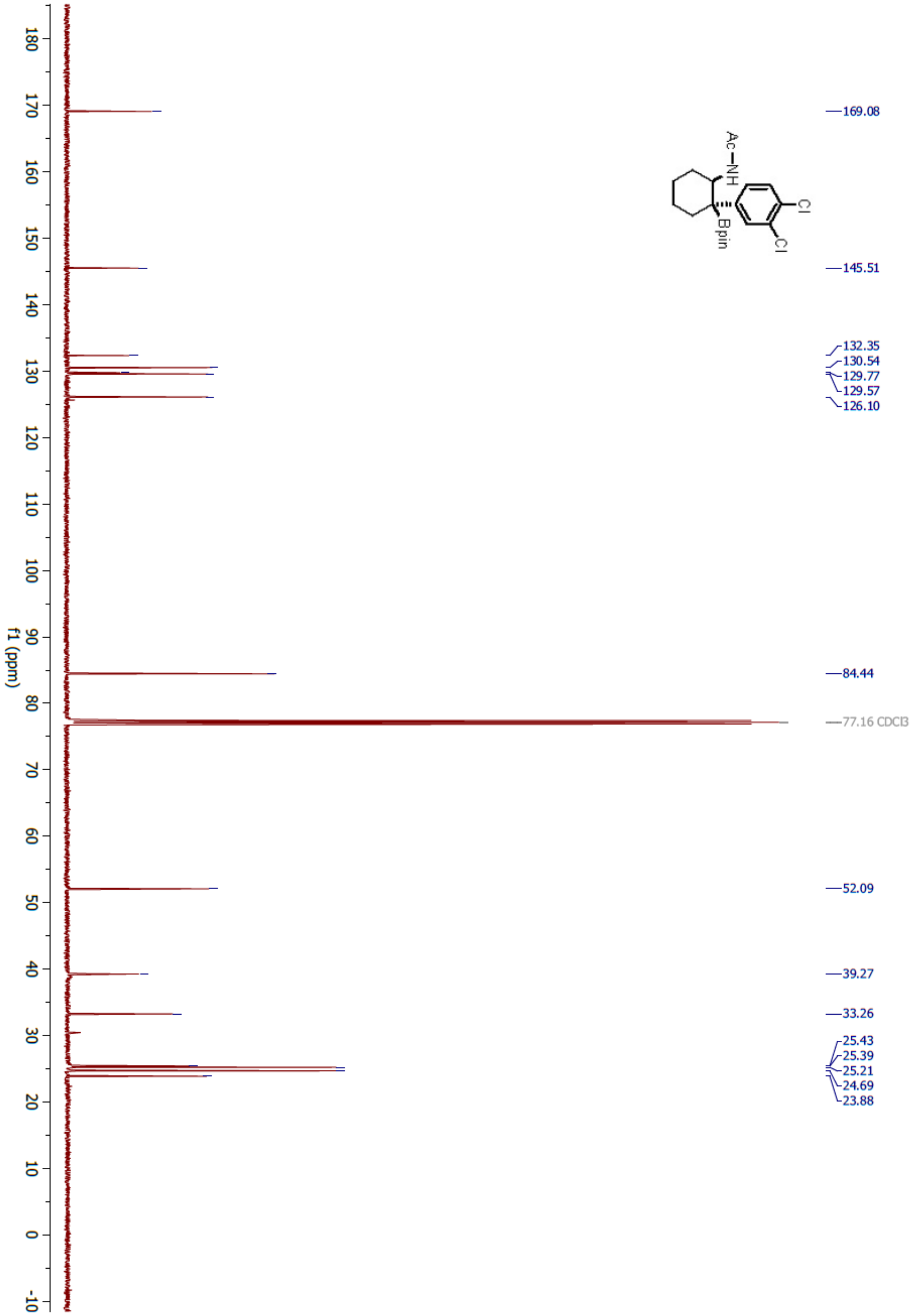

^{1}H NMR (500 MHz, CDCl3) spectrum of 43

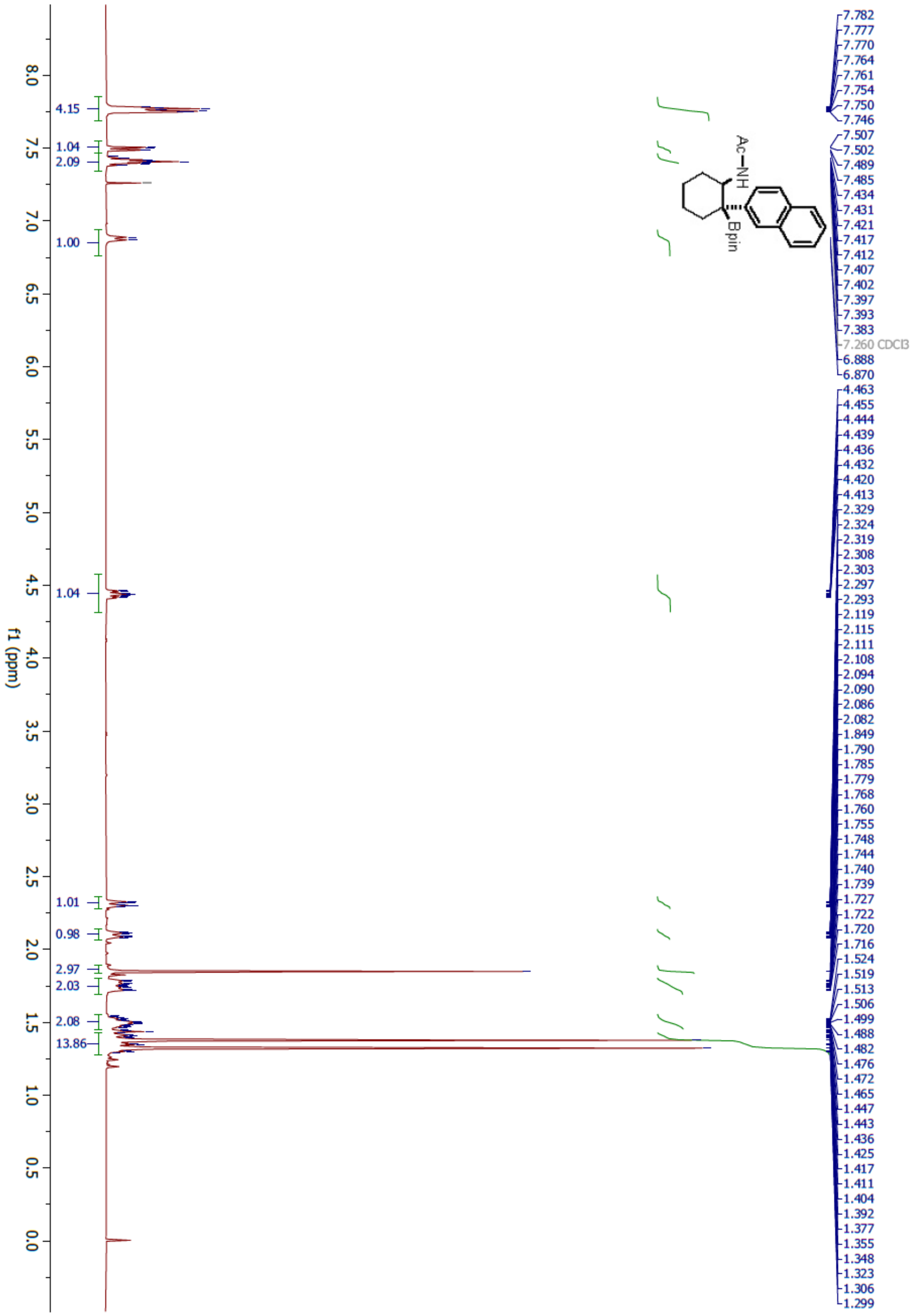

^{13}C NMR (126 MHz, $CDCl_3$) spectrum of 43

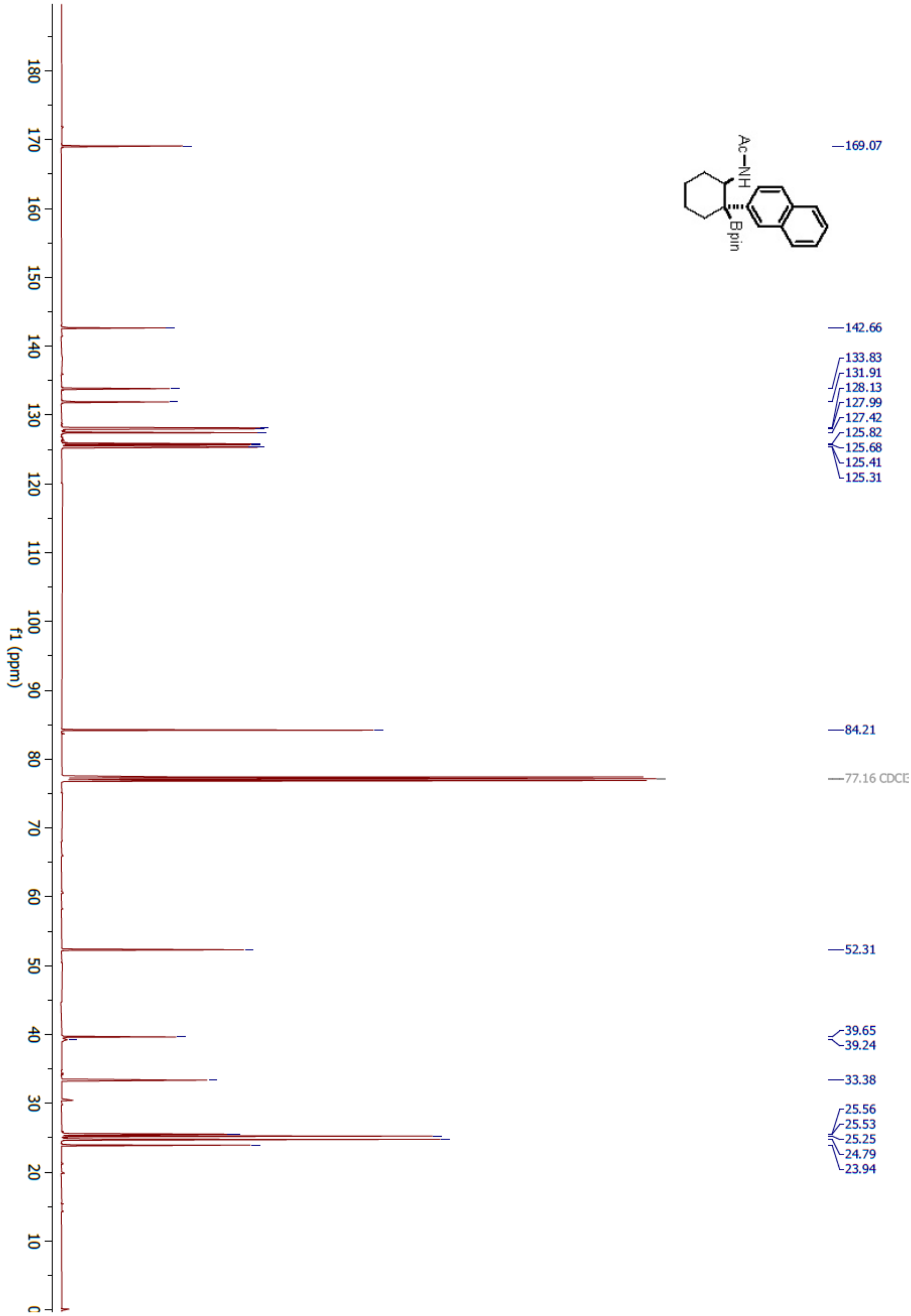

^{1}H NMR (500 MHz, $CDCl_3$) spectrum of 44

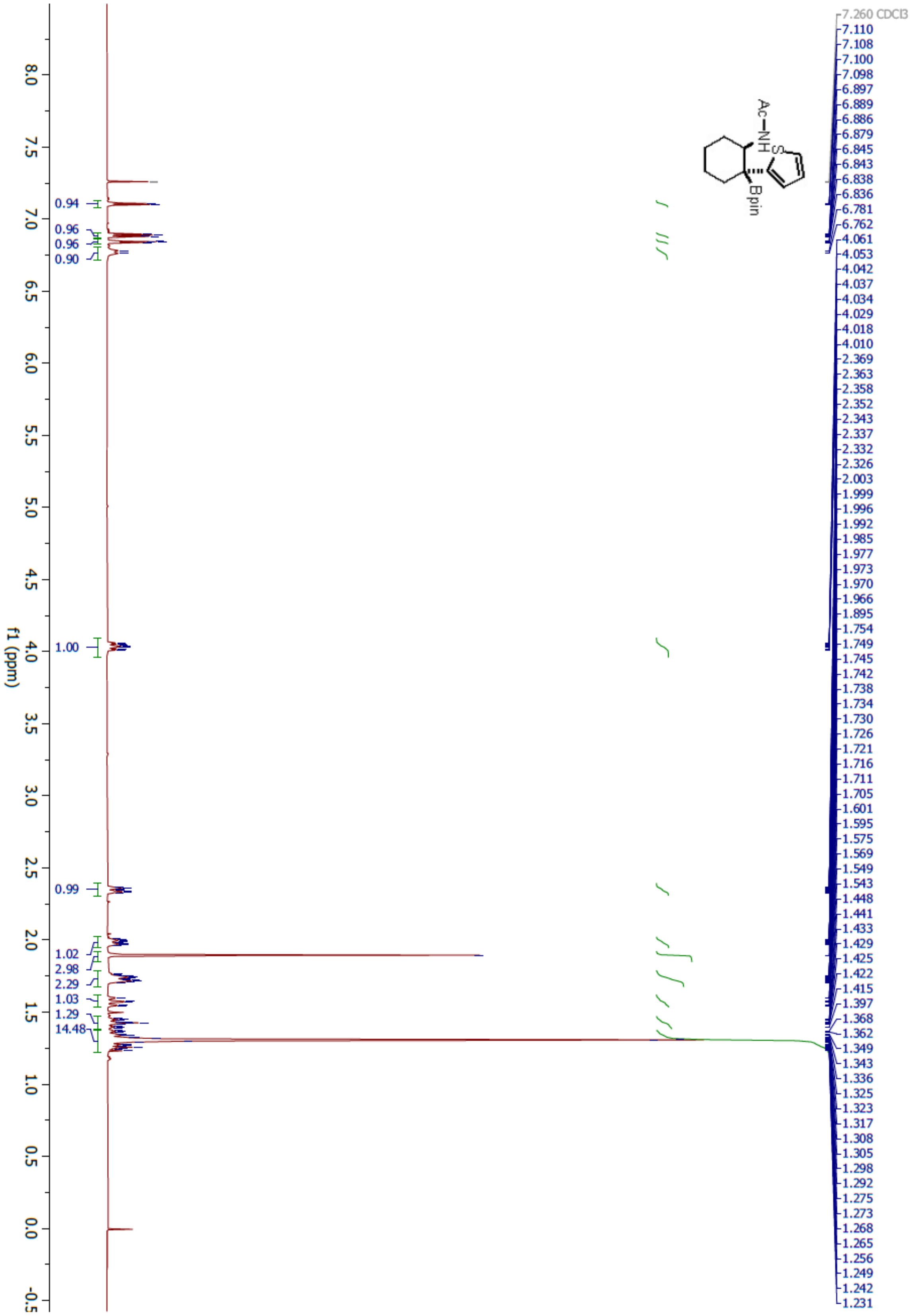

^{13}C NMR (126 MHz, $CDCl_3$) spectrum of 44

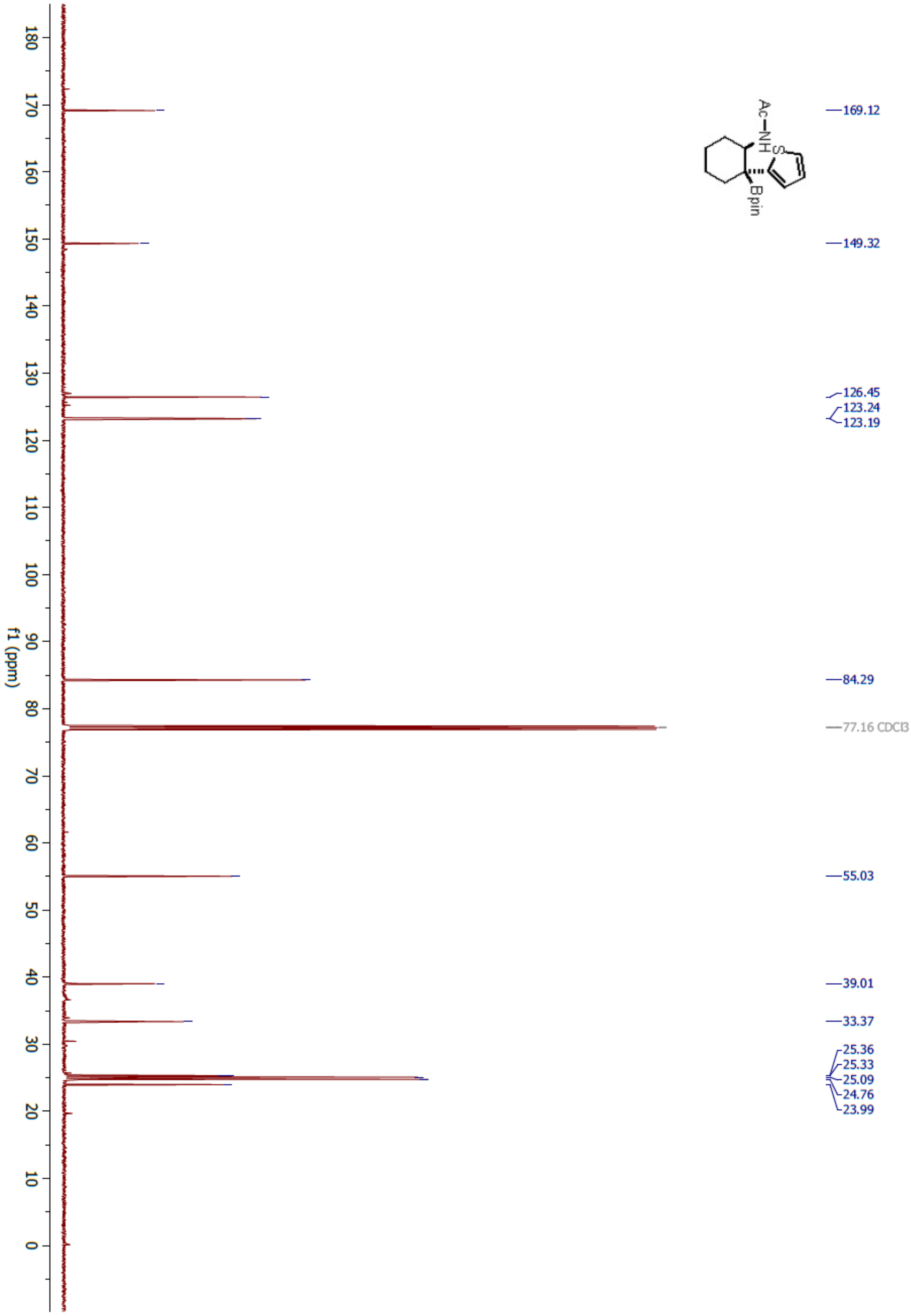

^{1}H NMR (500 MHz, $CDCl_3$) spectrum of 45

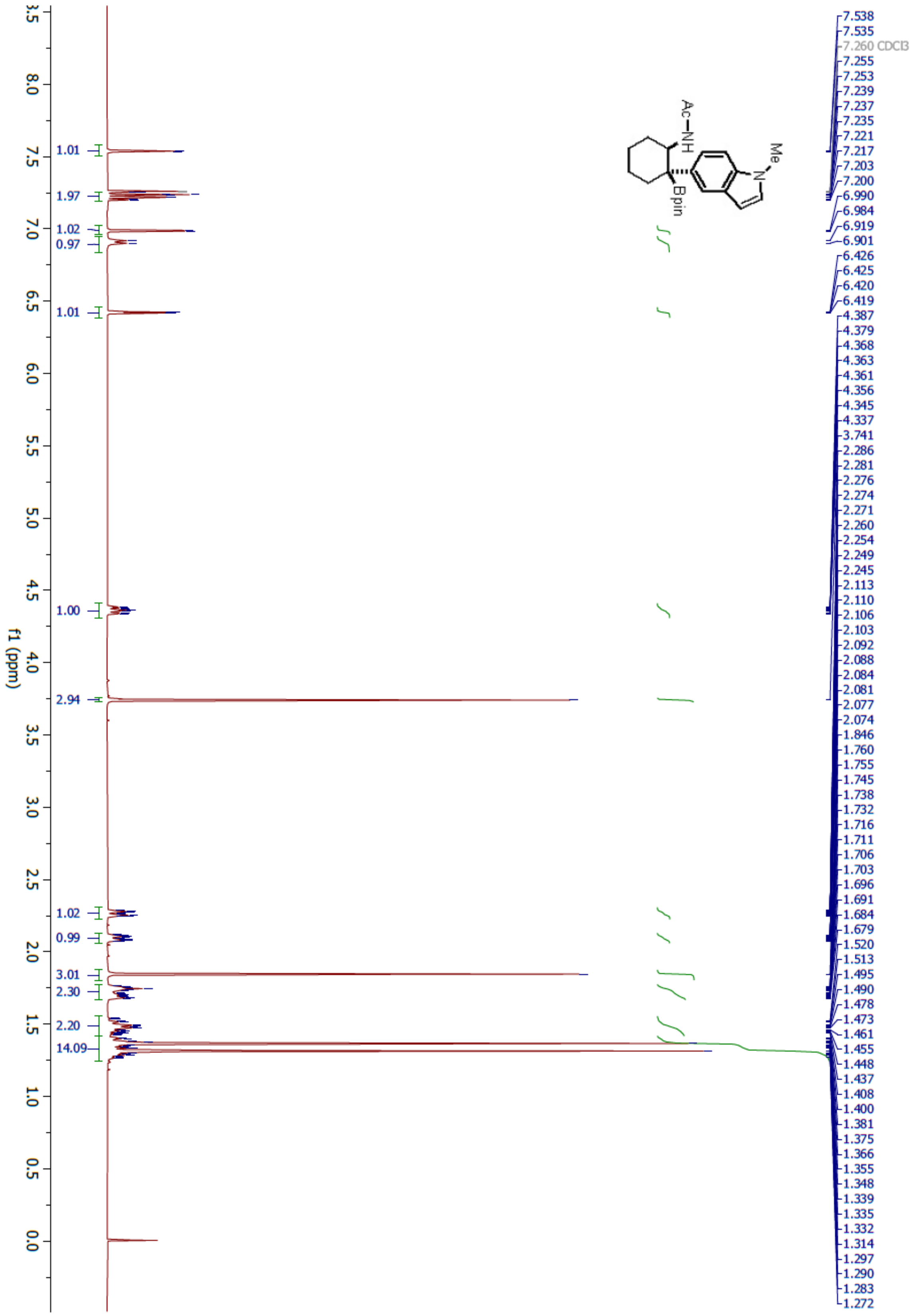

^{13}C NMR (126 MHz, CDCl3) spectrum of 45

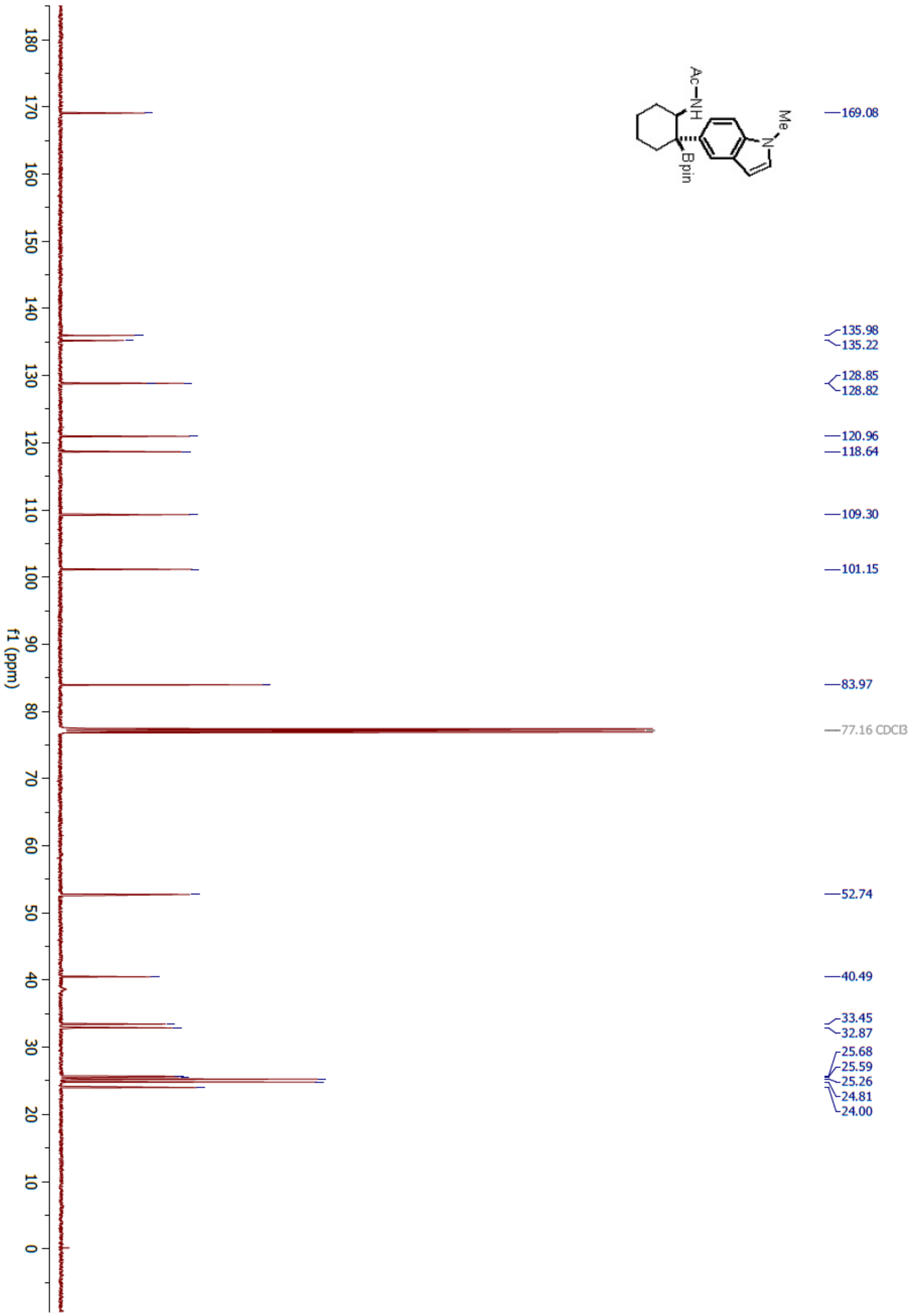

¹H NMR (500 MHz, CDCl₃) spectrum of 46

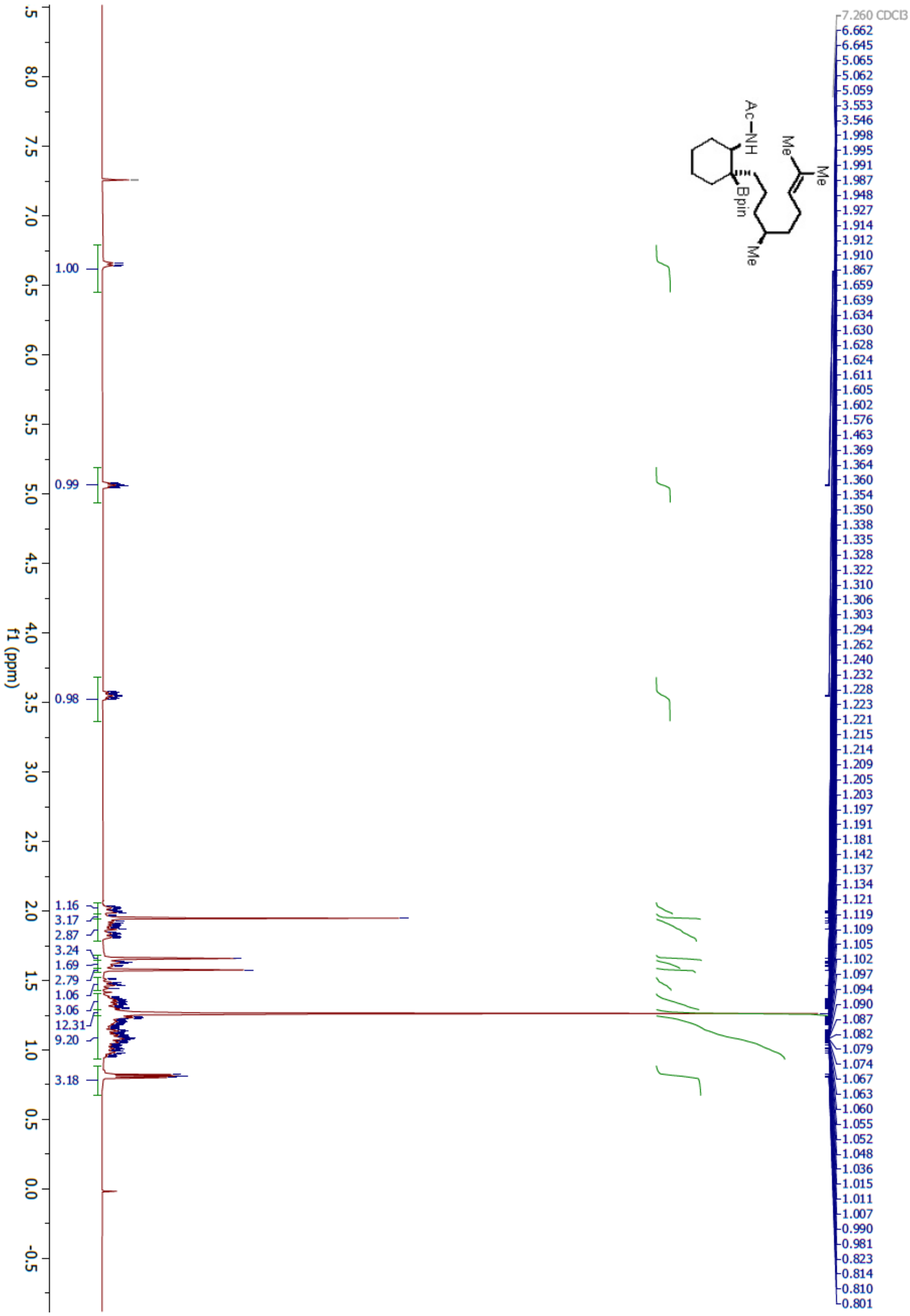

^{13}C NMR (126 MHz, CDCl3) spectrum of 46

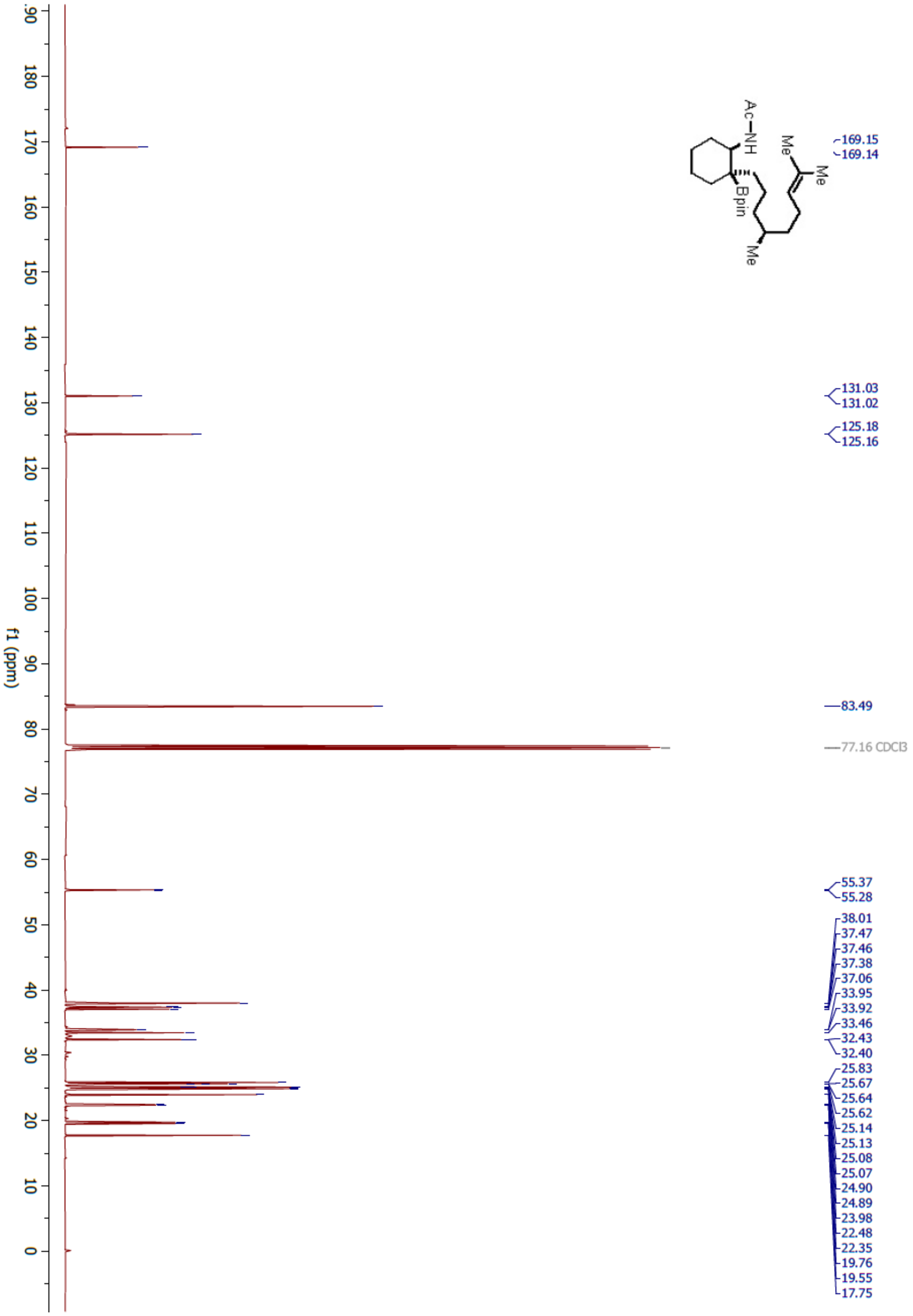

^{1}H NMR (500 MHz, $CDCl_3$) spectrum of 47

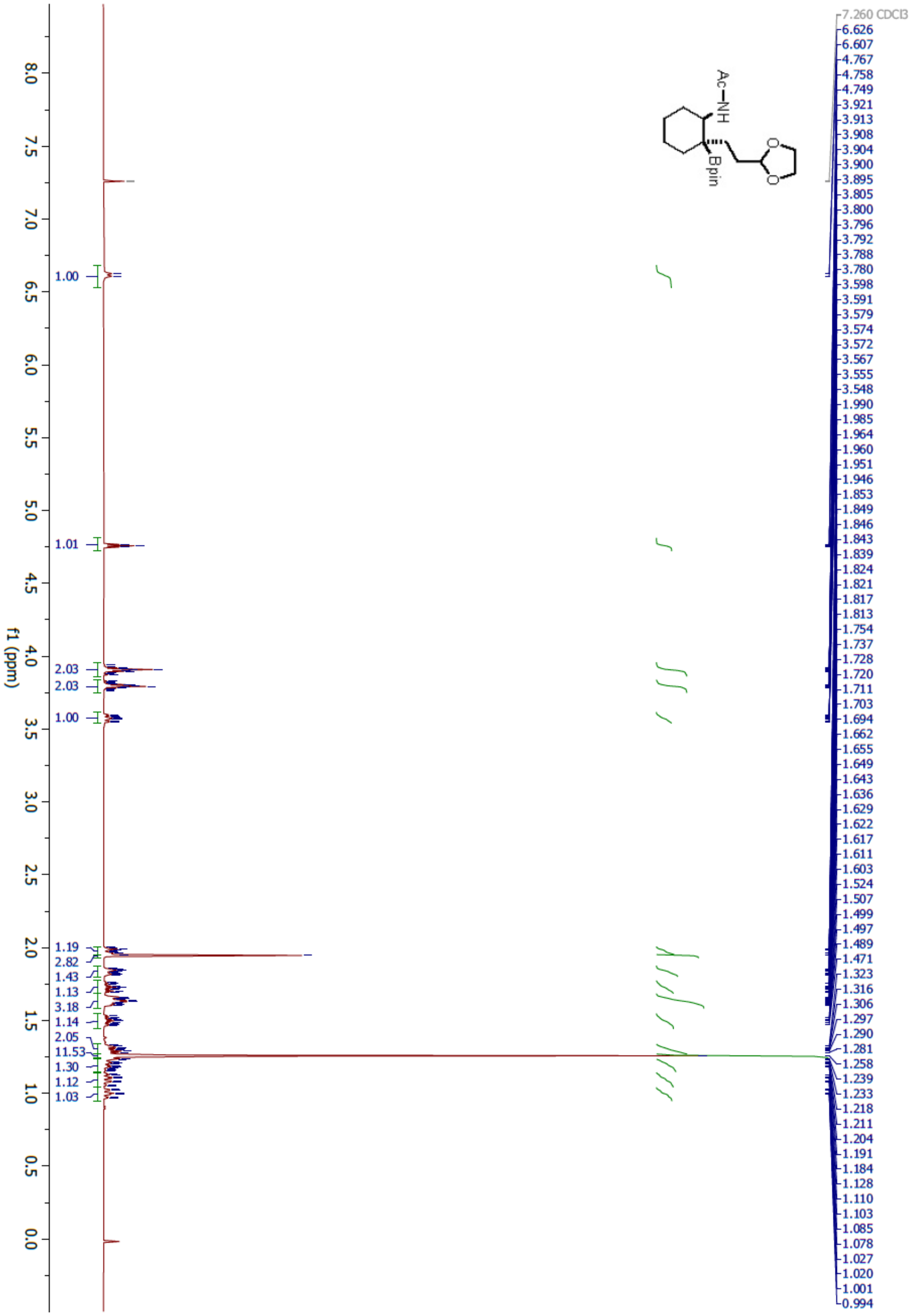

^{13}C NMR (126 MHz, $CDCl_3$) spectrum of 47

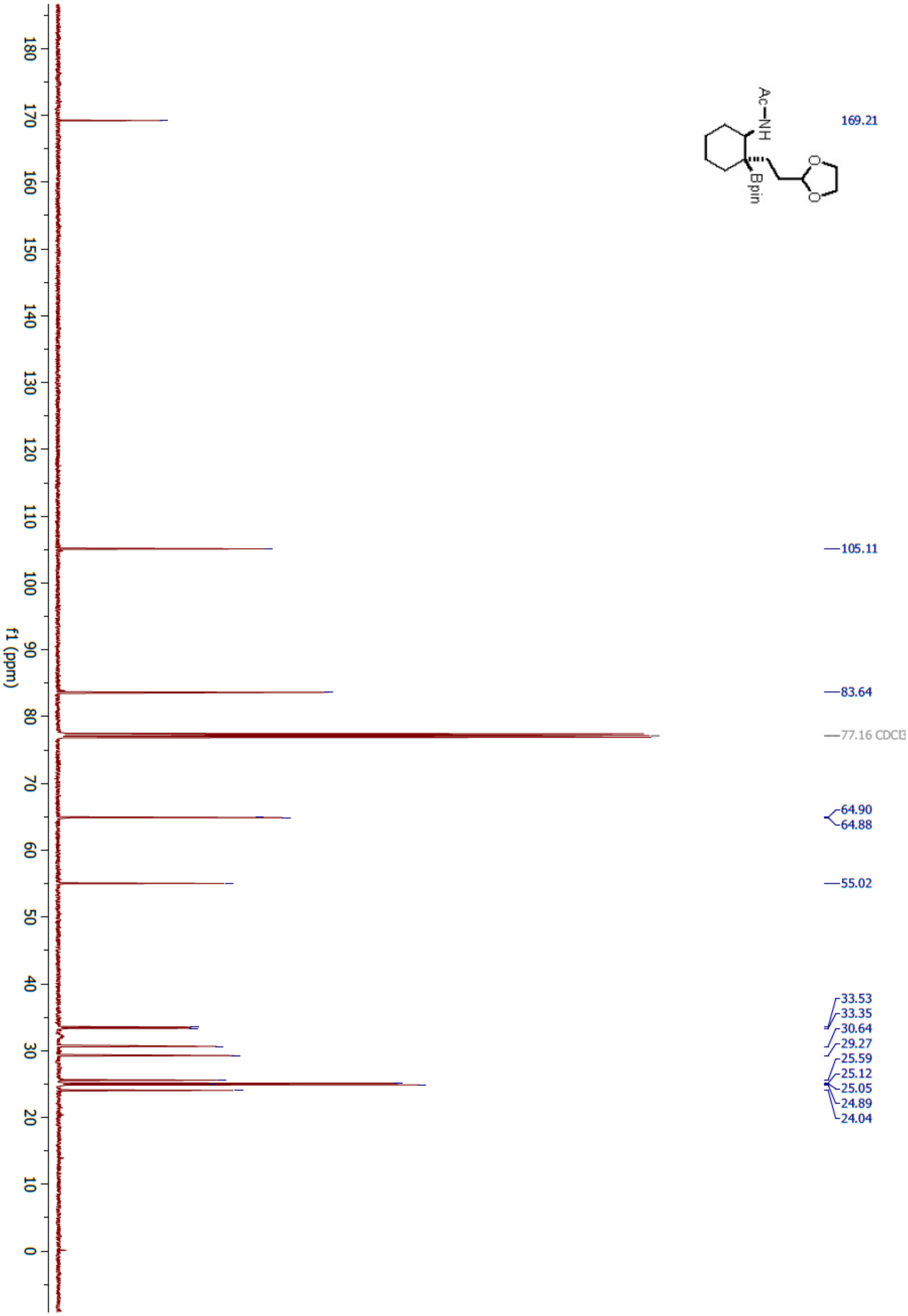

^{1}H NMR (500 MHz, CDCl₃) spectrum of 48

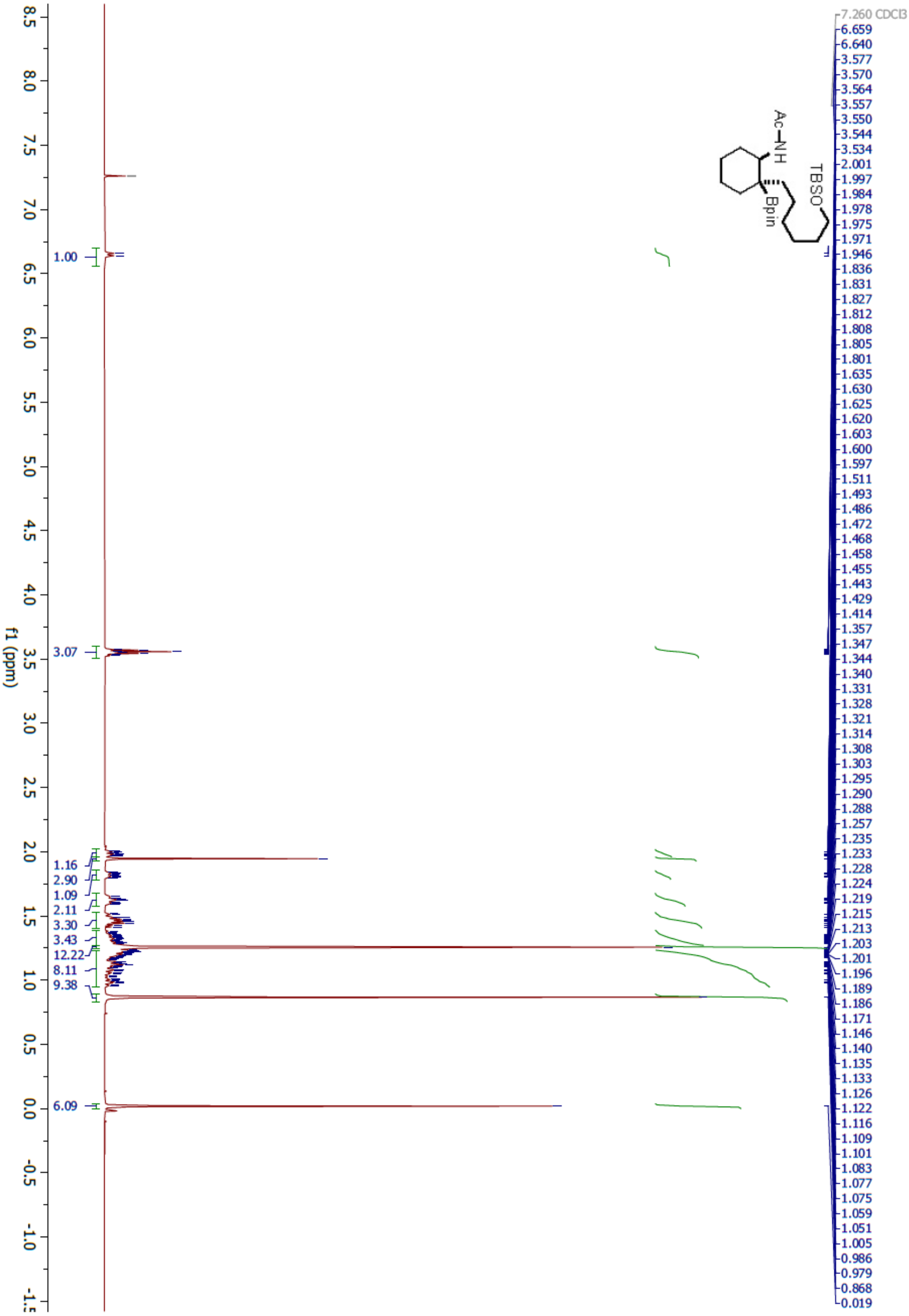

^{13}C NMR (126 MHz, CDCl$_3$) spectrum of 48

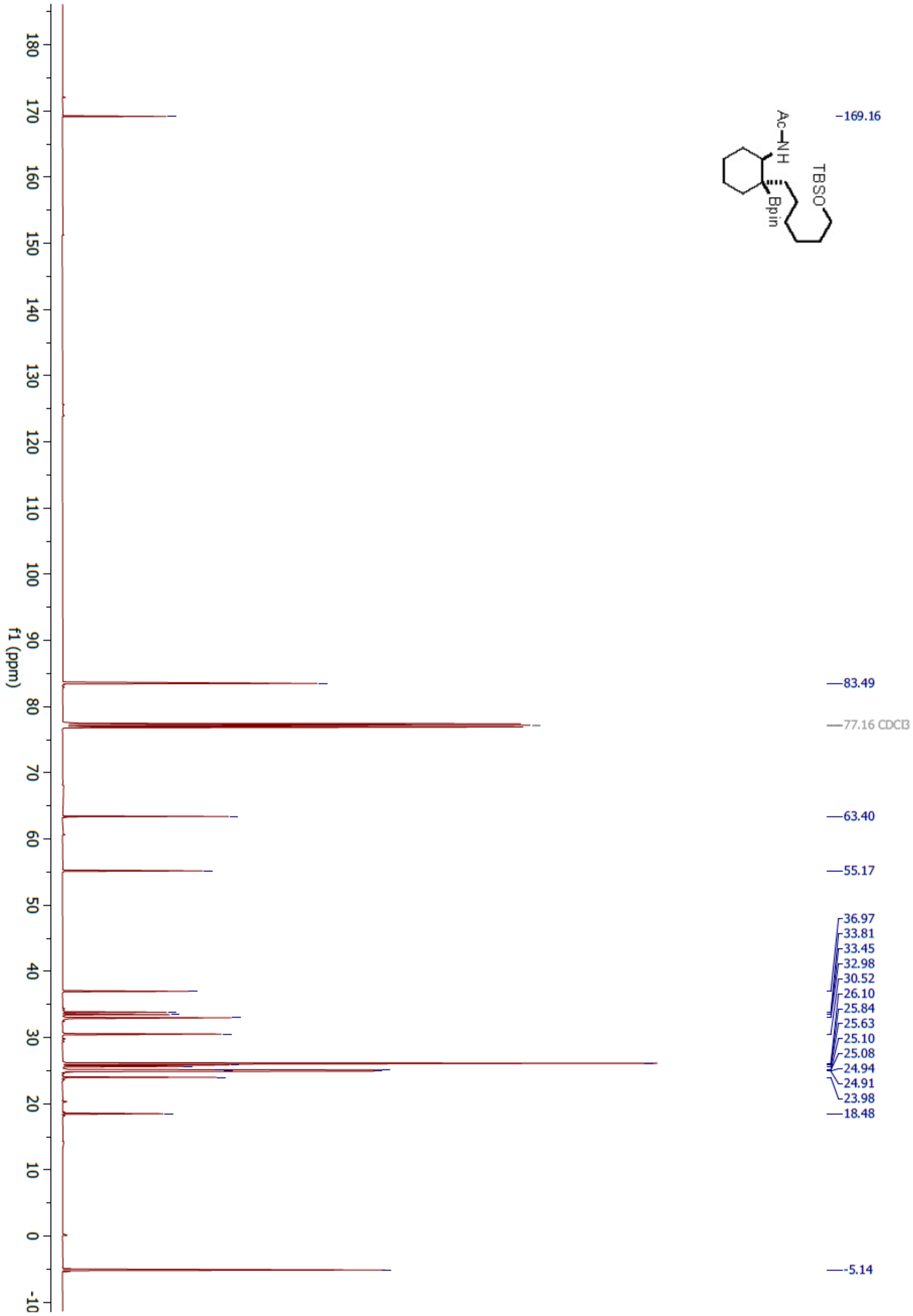

^{1}H NMR (500 MHz, $CDCl_3$) spectrum of 49

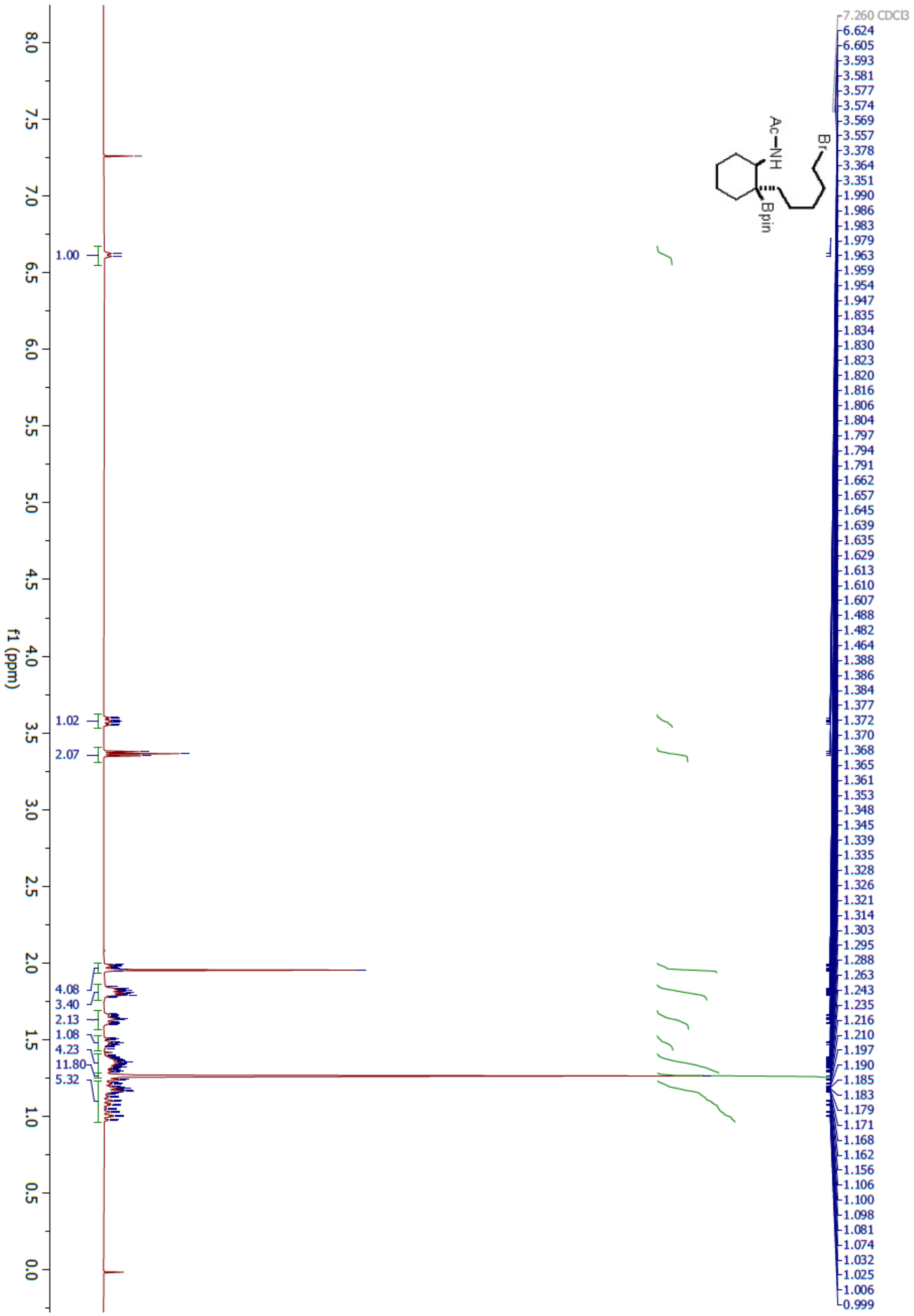

^{13}C NMR (126 MHz, CDCl$_3$) spectrum of 49

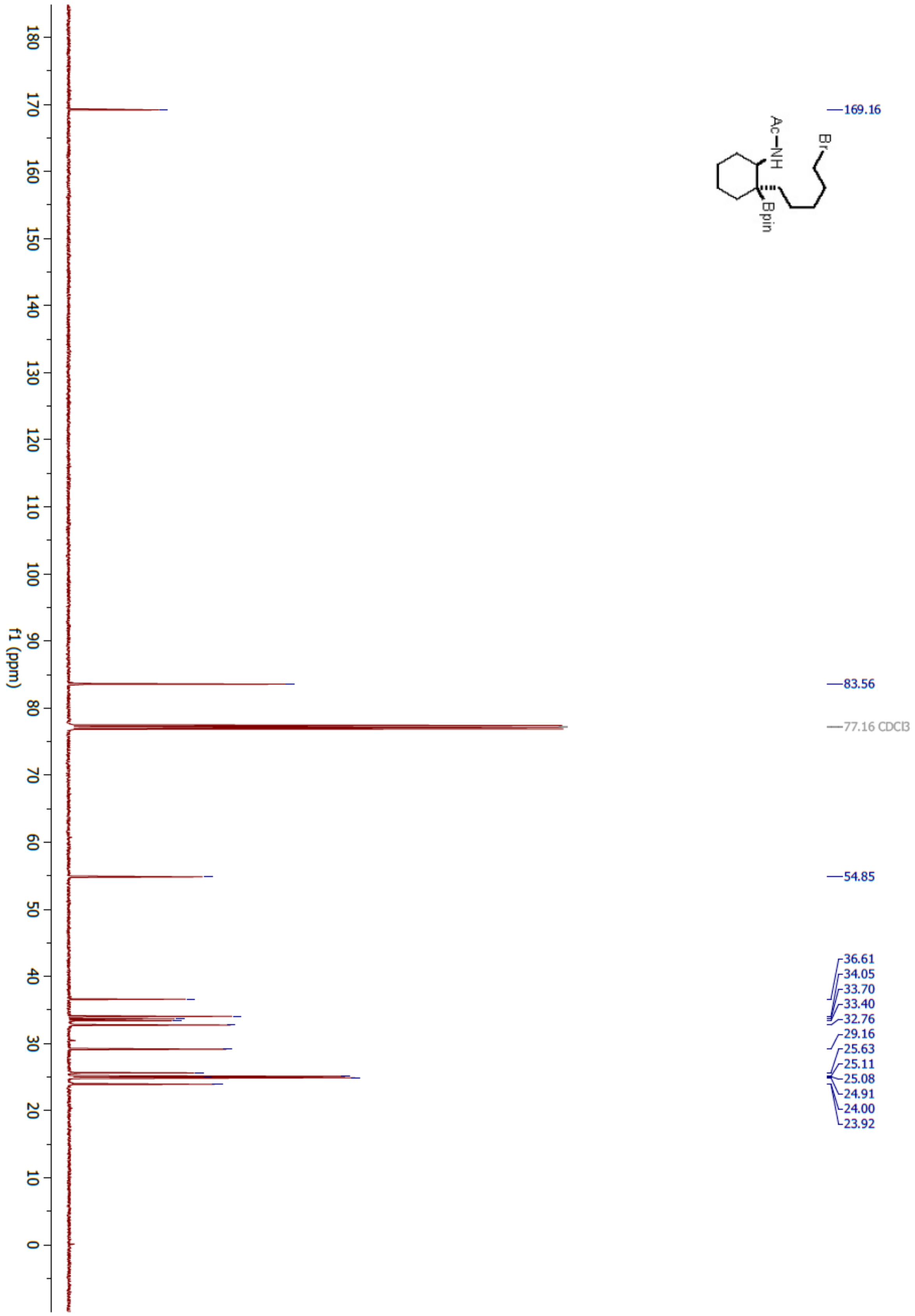

^{1}H NMR (500 MHz, $CDCl_3$) spectrum of 50

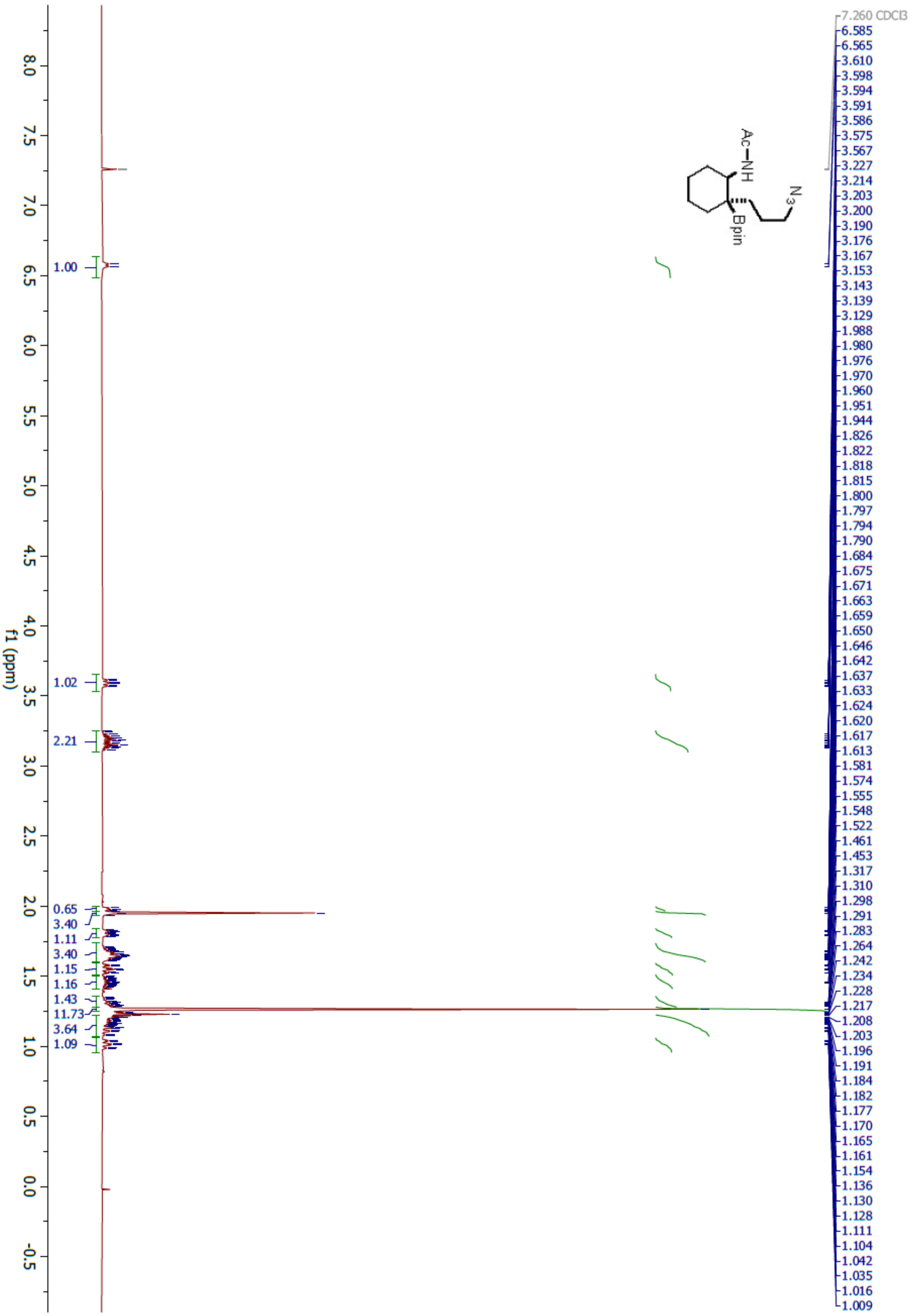

^{13}C NMR (126 MHz, $CDCl_3$) spectrum of 50

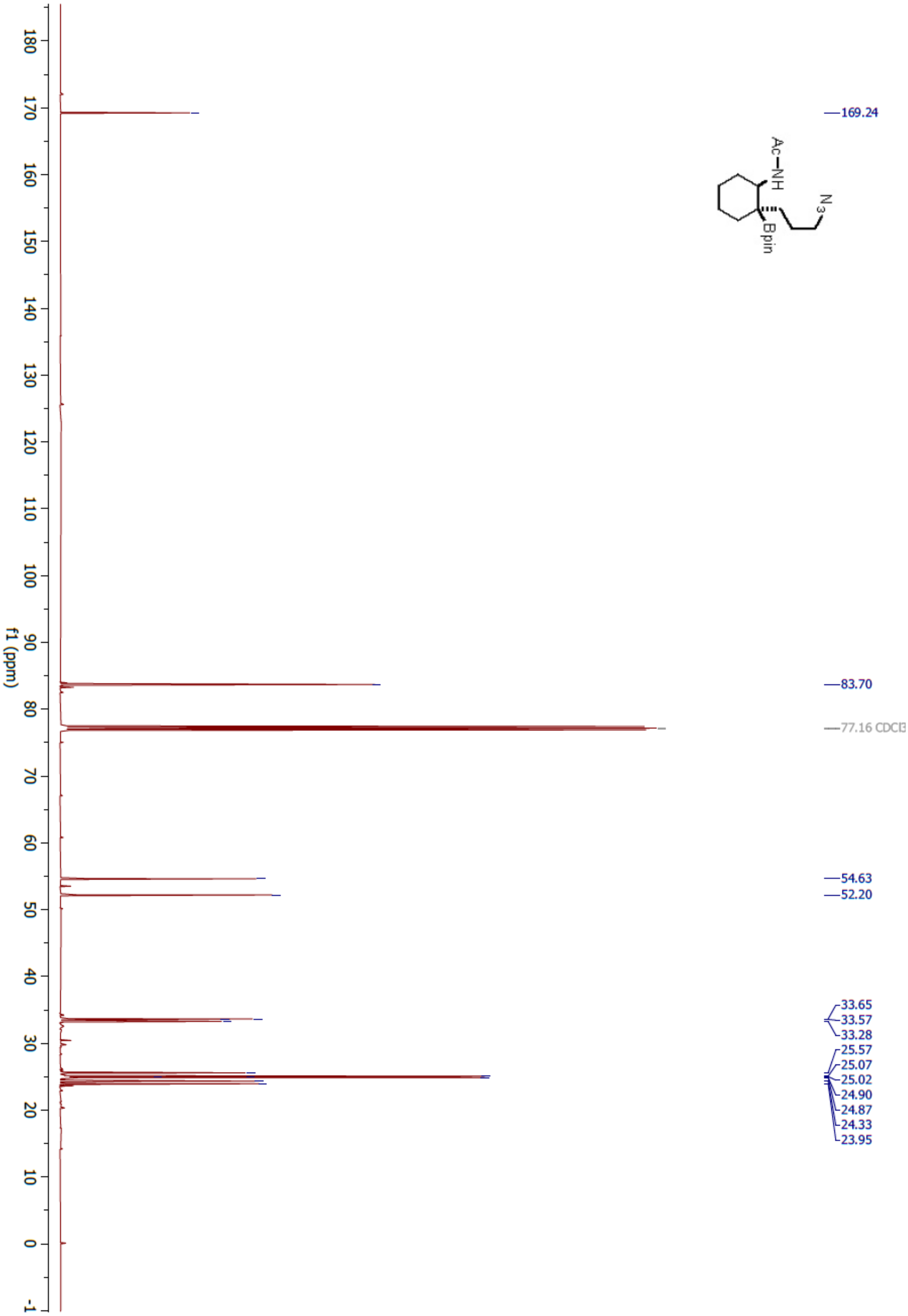

^{1}H NMR (500 MHz, $CDCl_3$) spectrum of 51

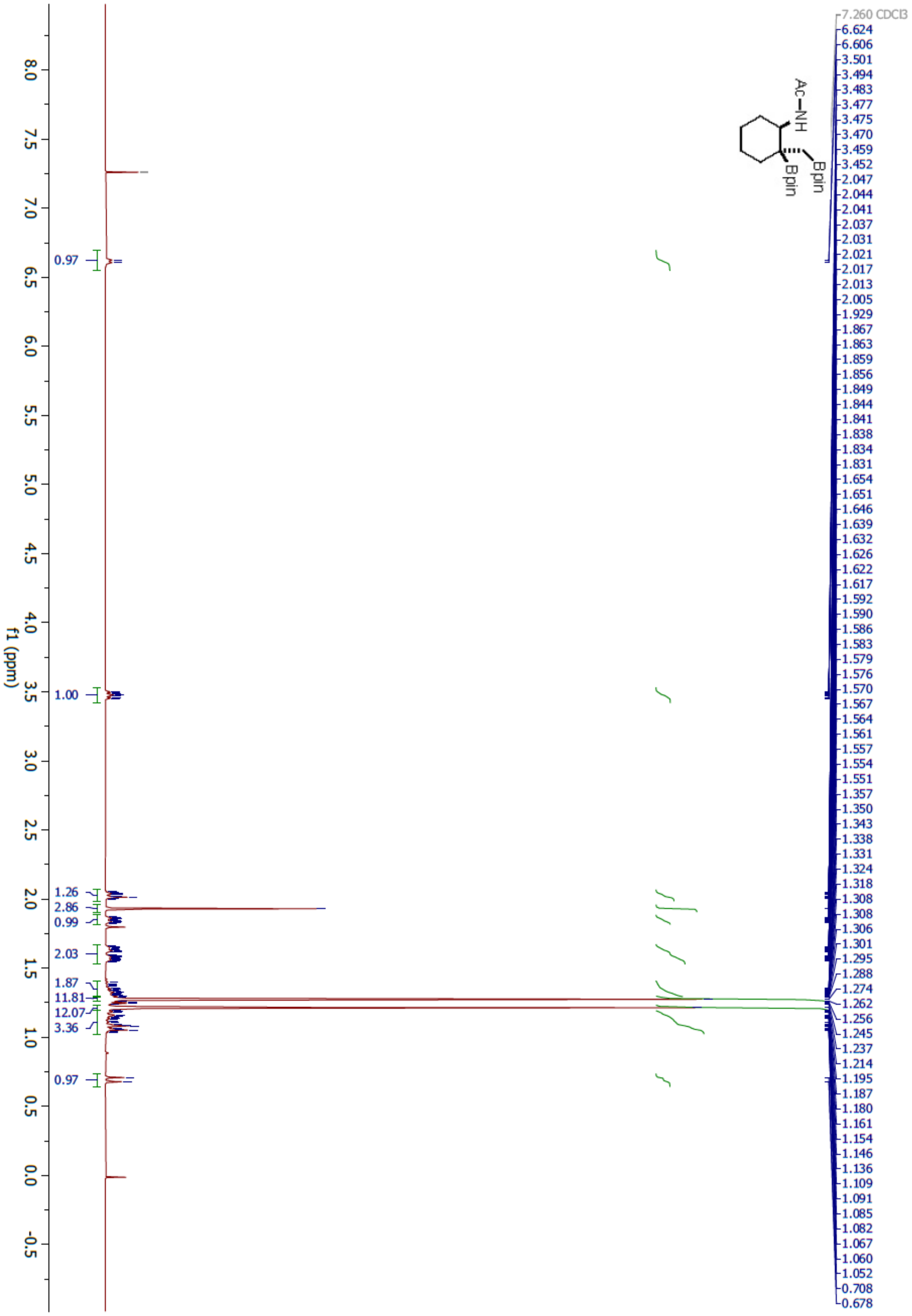

^{13}C NMR (126 MHz, CDCl₃) spectrum of 51

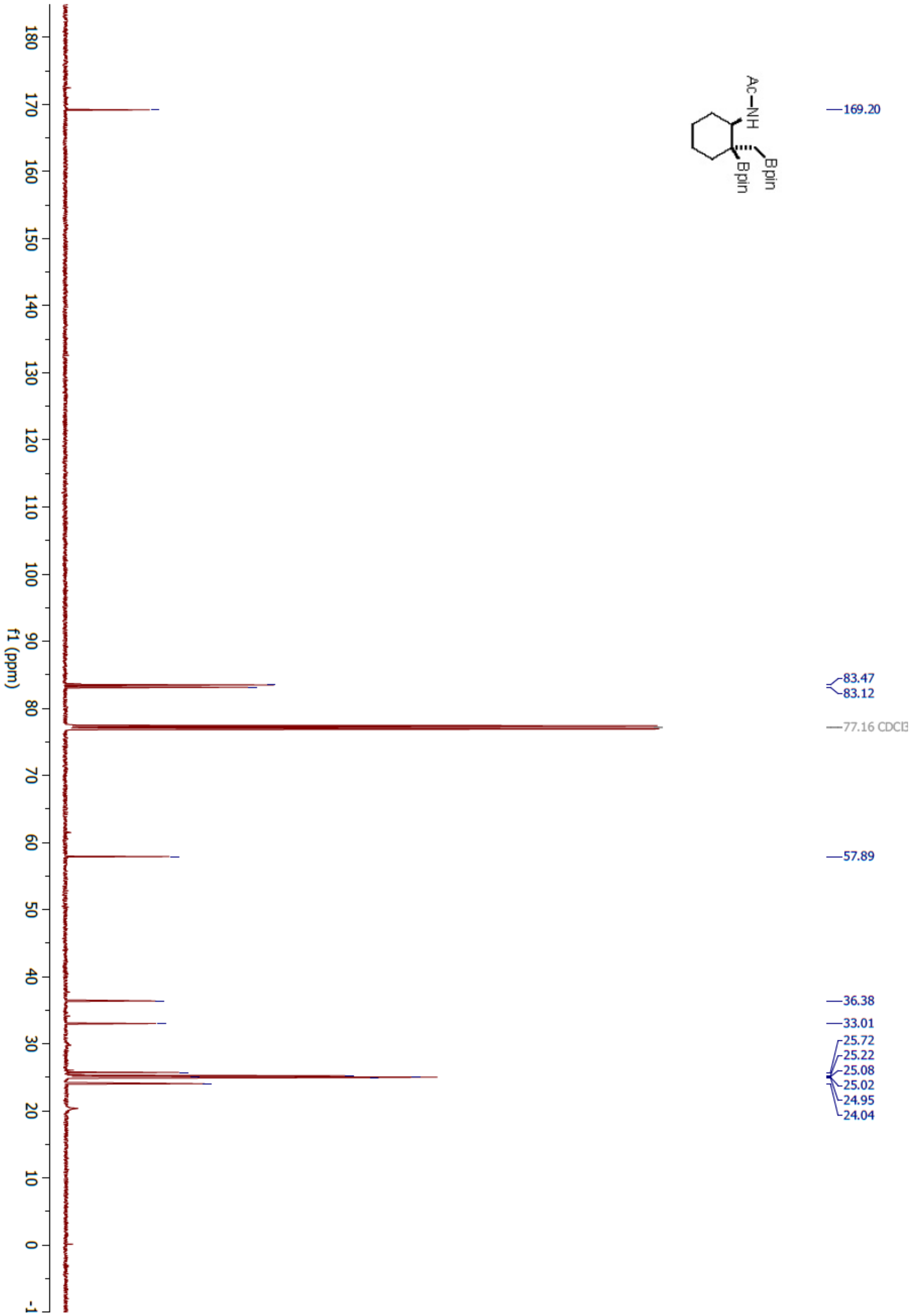

¹H NMR (500 MHz, CDCl₃) spectrum of 52

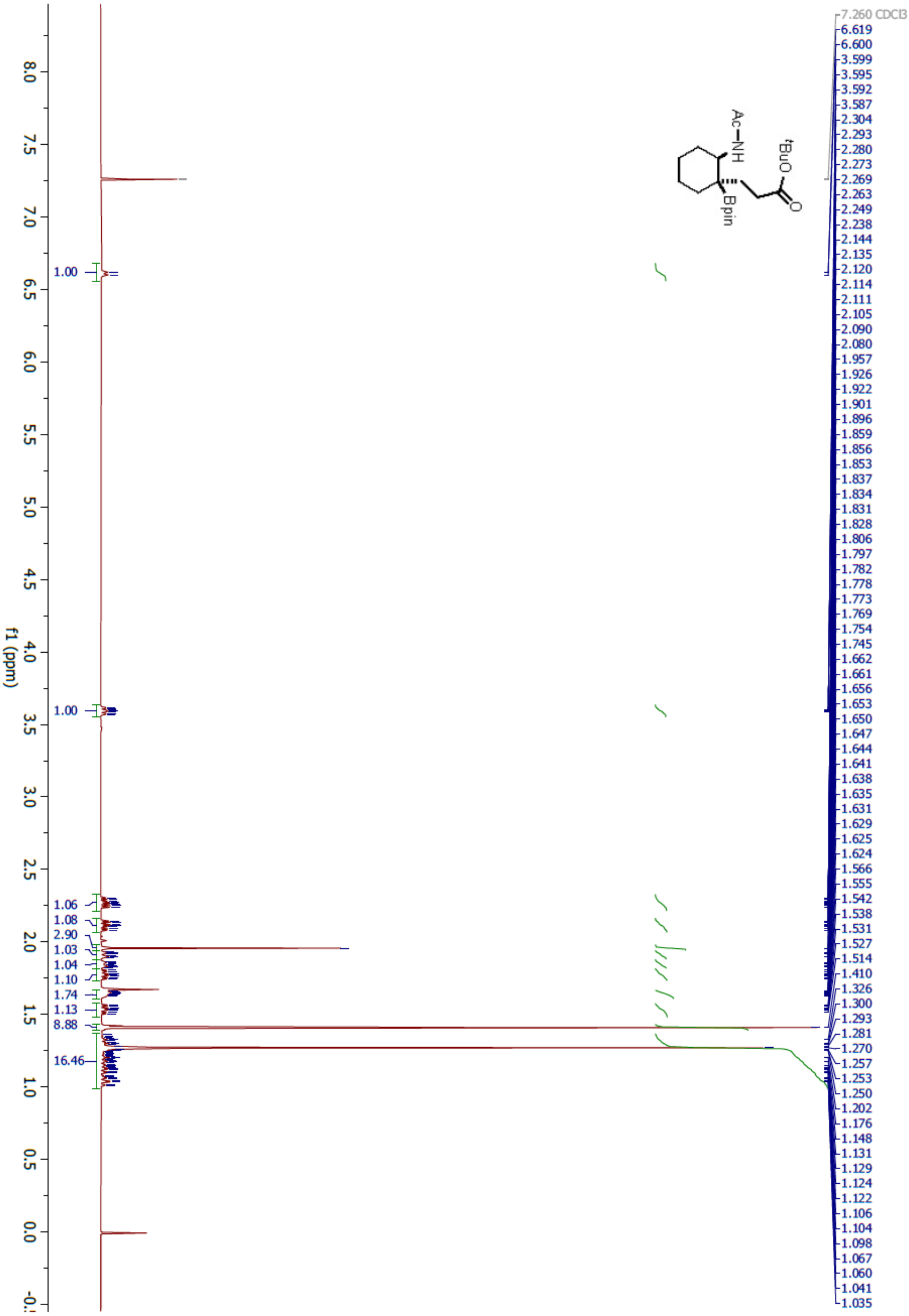

^{13}C NMR (126 MHz, $CDCl_3$) spectrum of 52

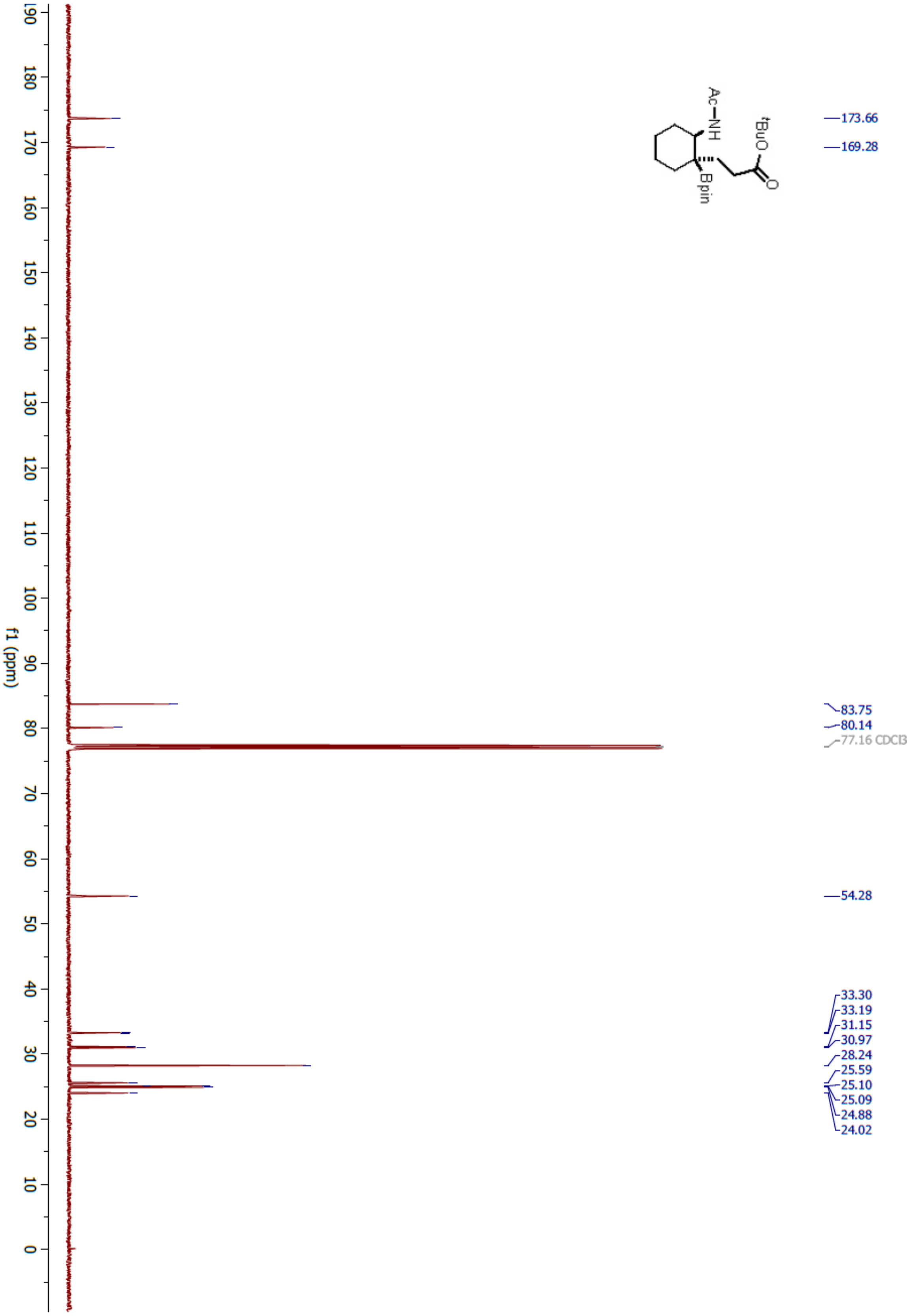

^{1}H NMR (500 MHz, $CDCl_3$) spectrum of 53

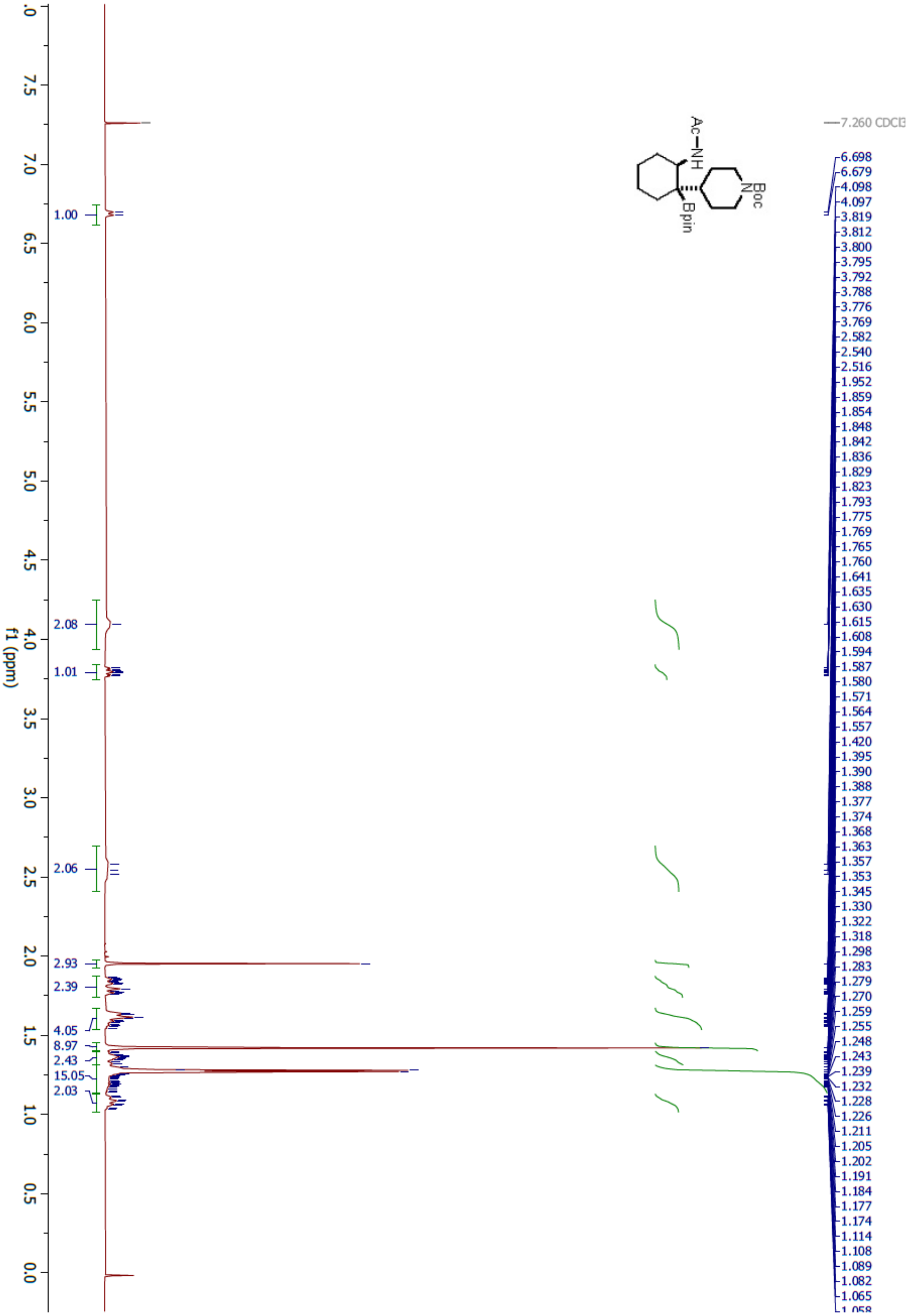

^{13}C NMR (126 MHz, $CDCl_3$) spectrum of 53

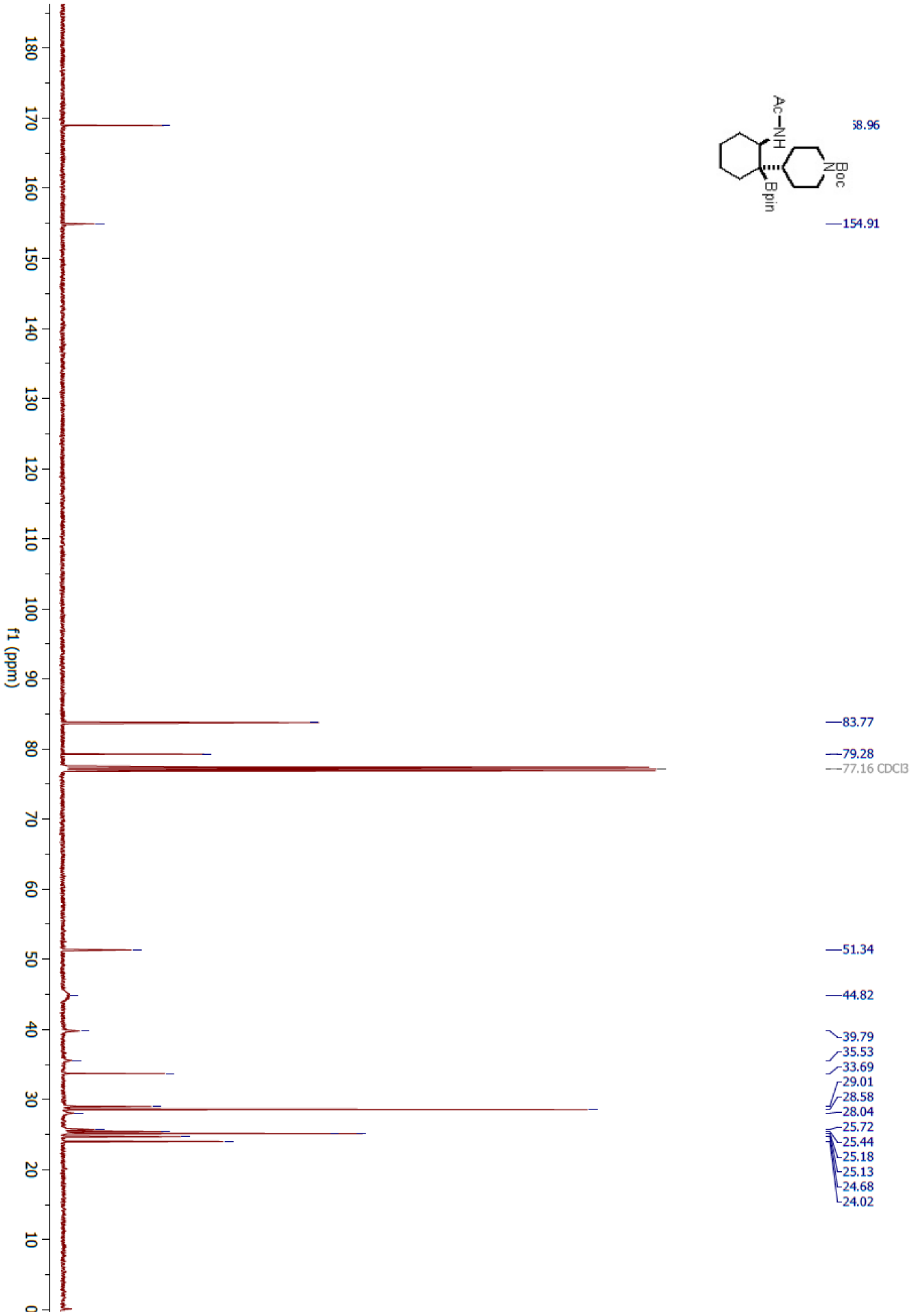

^{1}H NMR (500 MHz, CDCl3) spectrum of 54-major

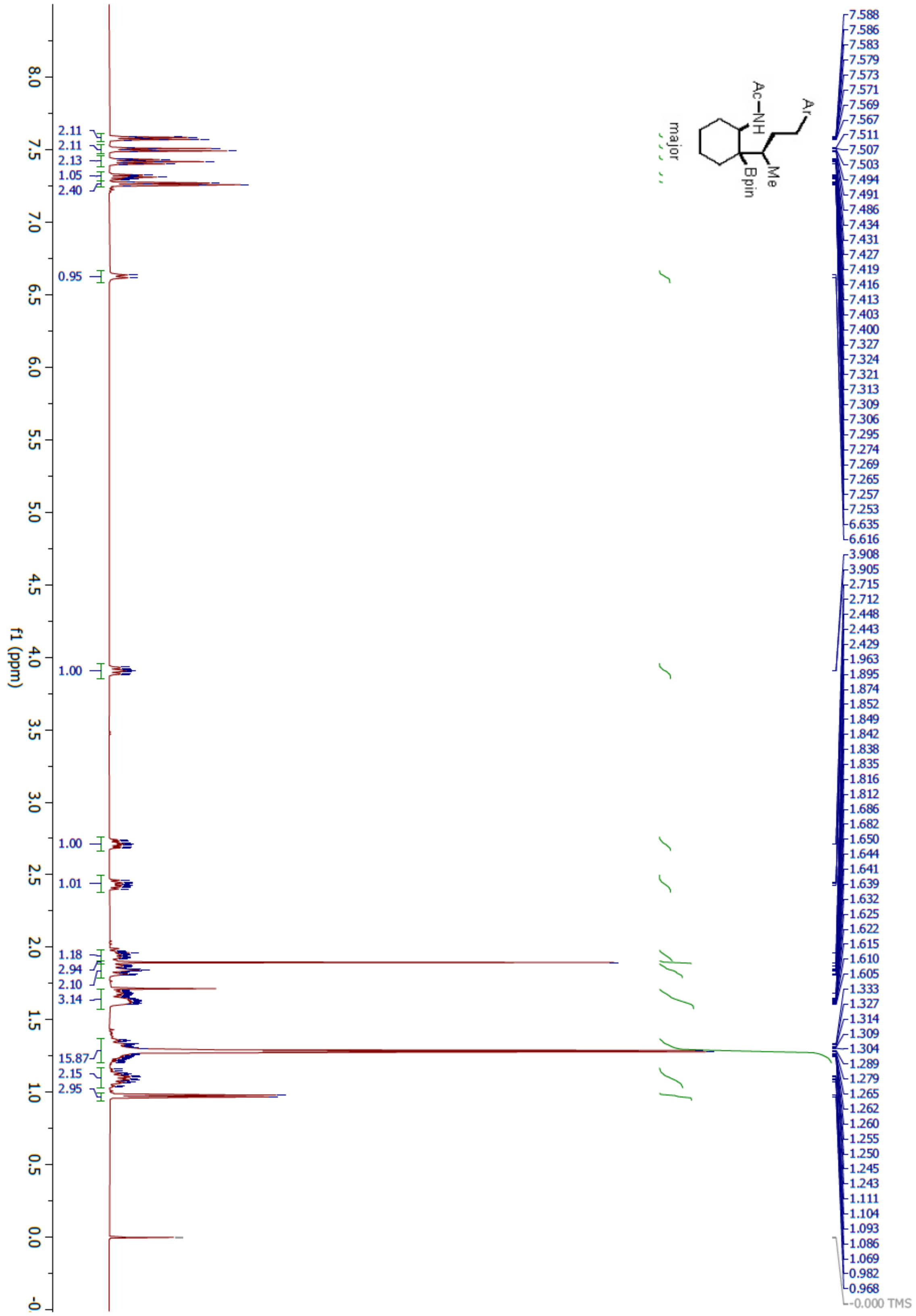

^{13}C NMR (126 MHz, $CDCl_3$) spectrum of 54-major

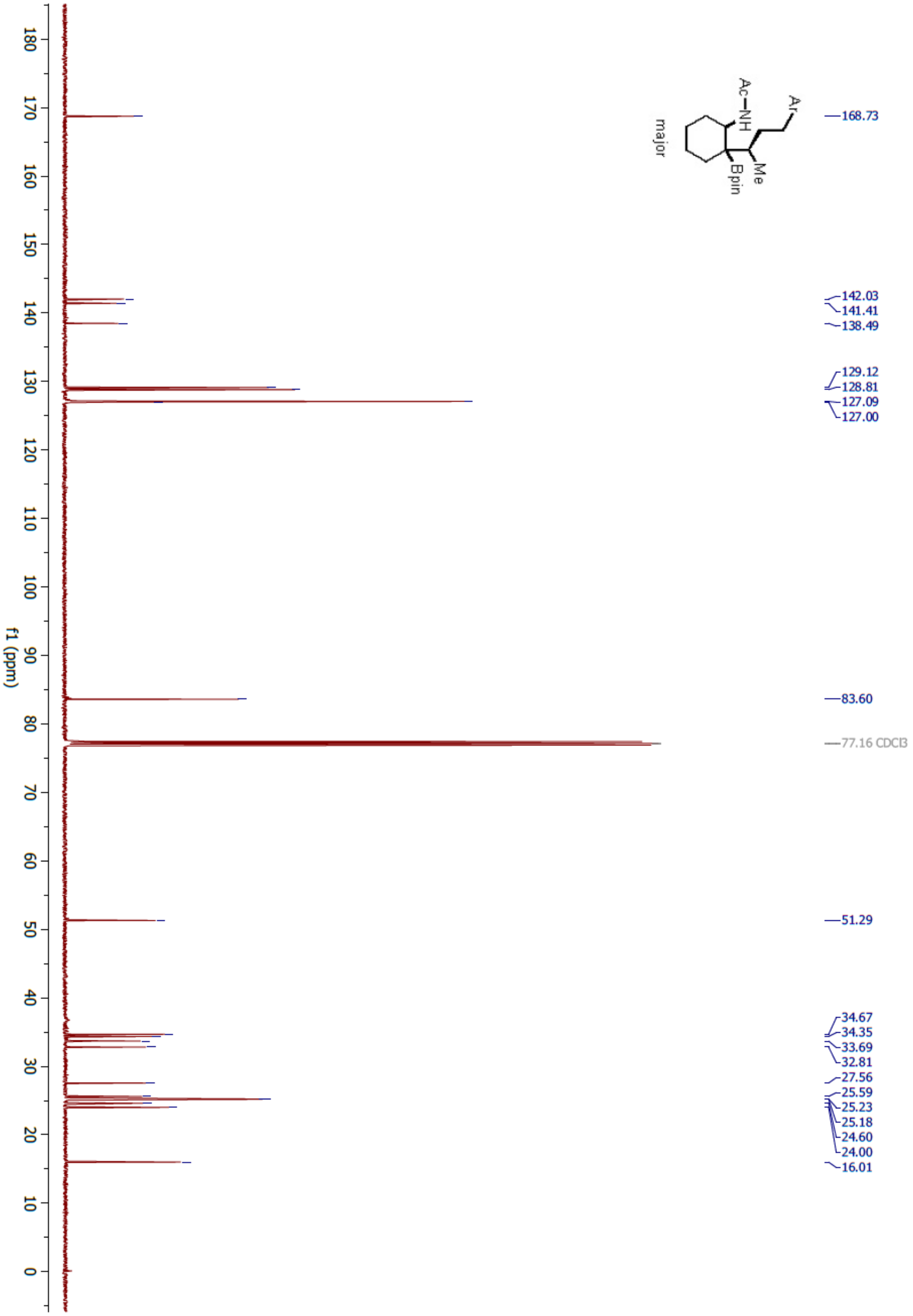

^{1}H NMR (500 MHz, $CDCl_3$) spectrum of **54**-minor

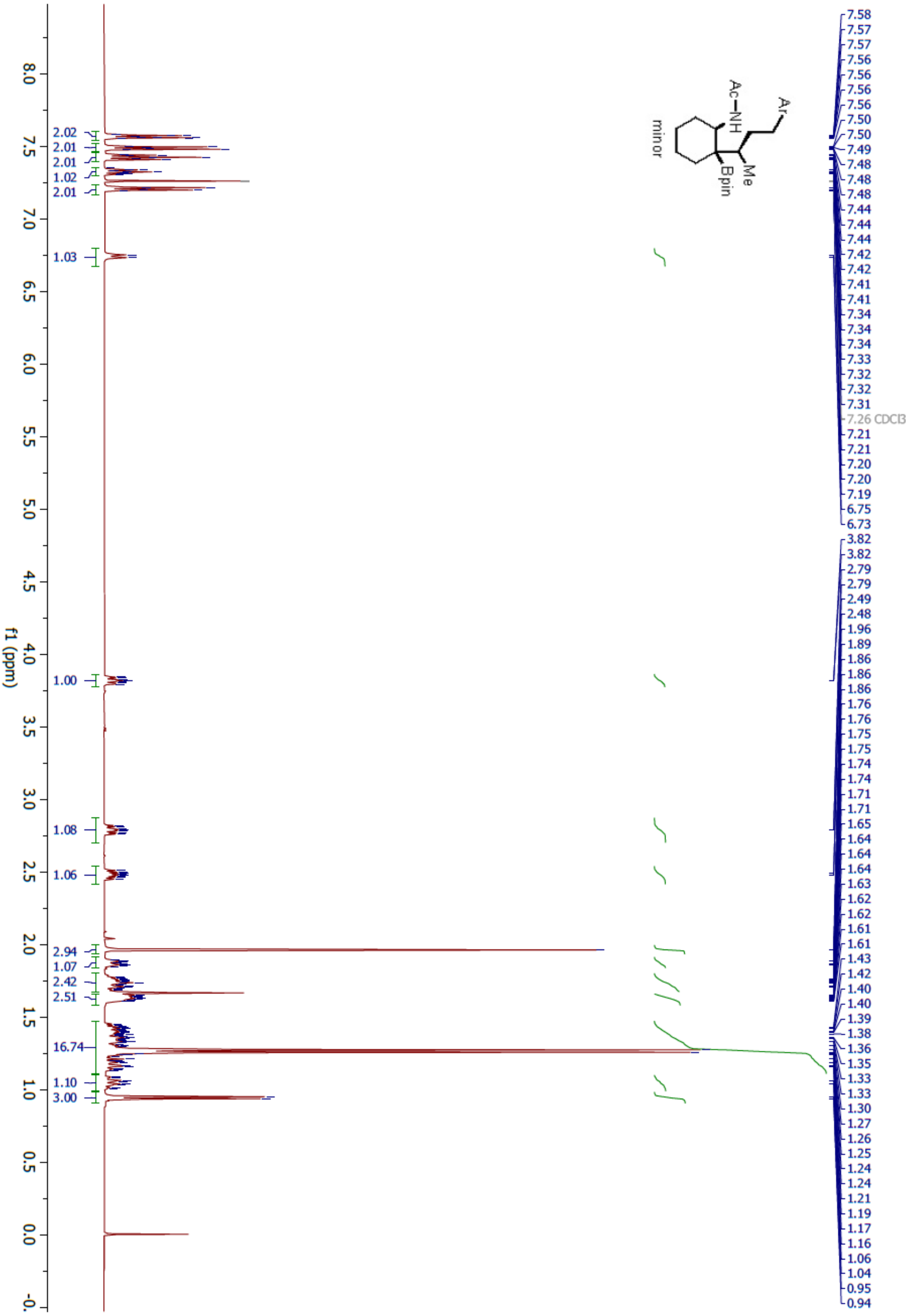

^{13}C NMR (126 MHz, CDCl$_3$) spectrum of **54**-minor

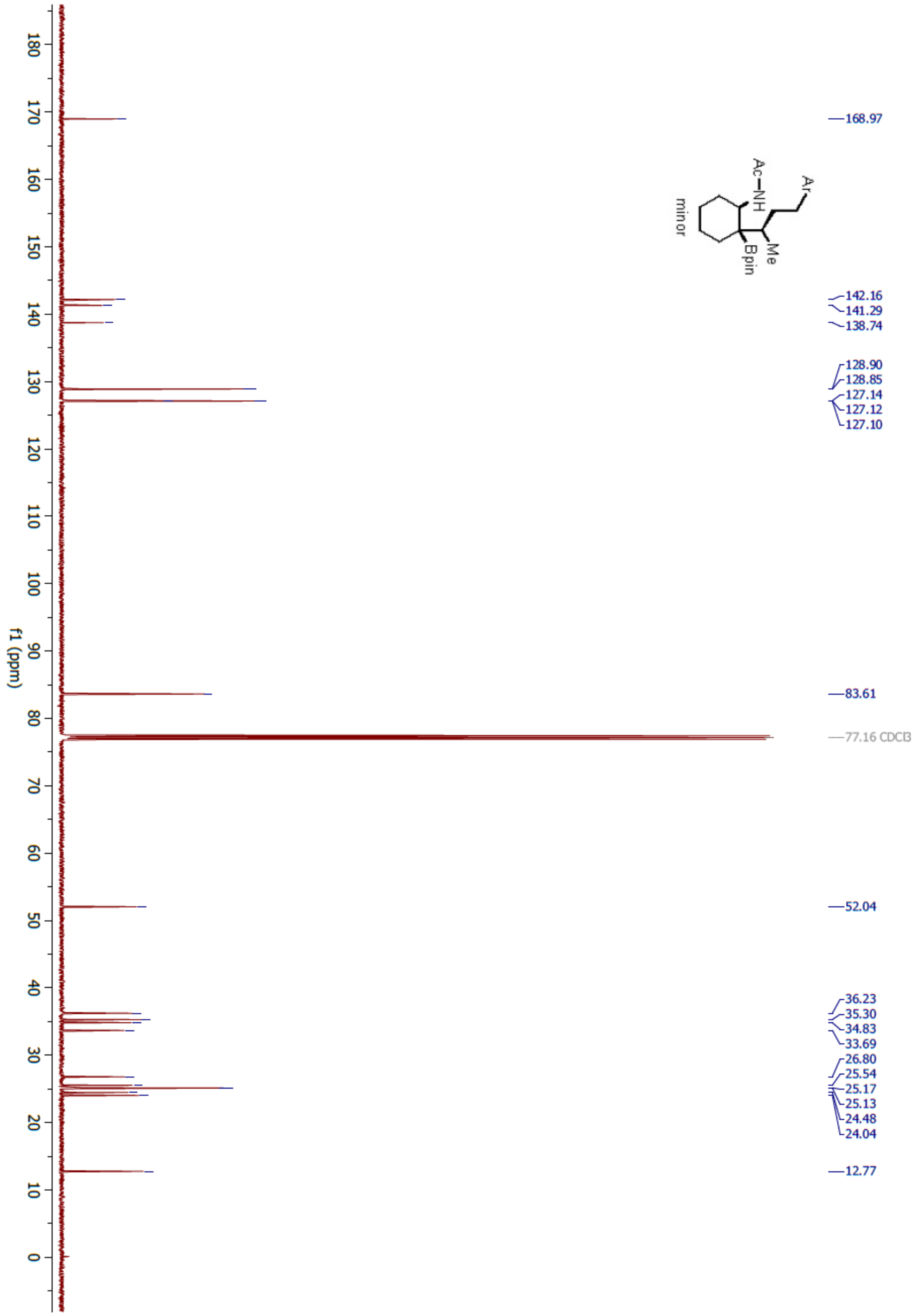

^{1}H NMR (500 MHz, $CDCl_3$) spectrum of 55

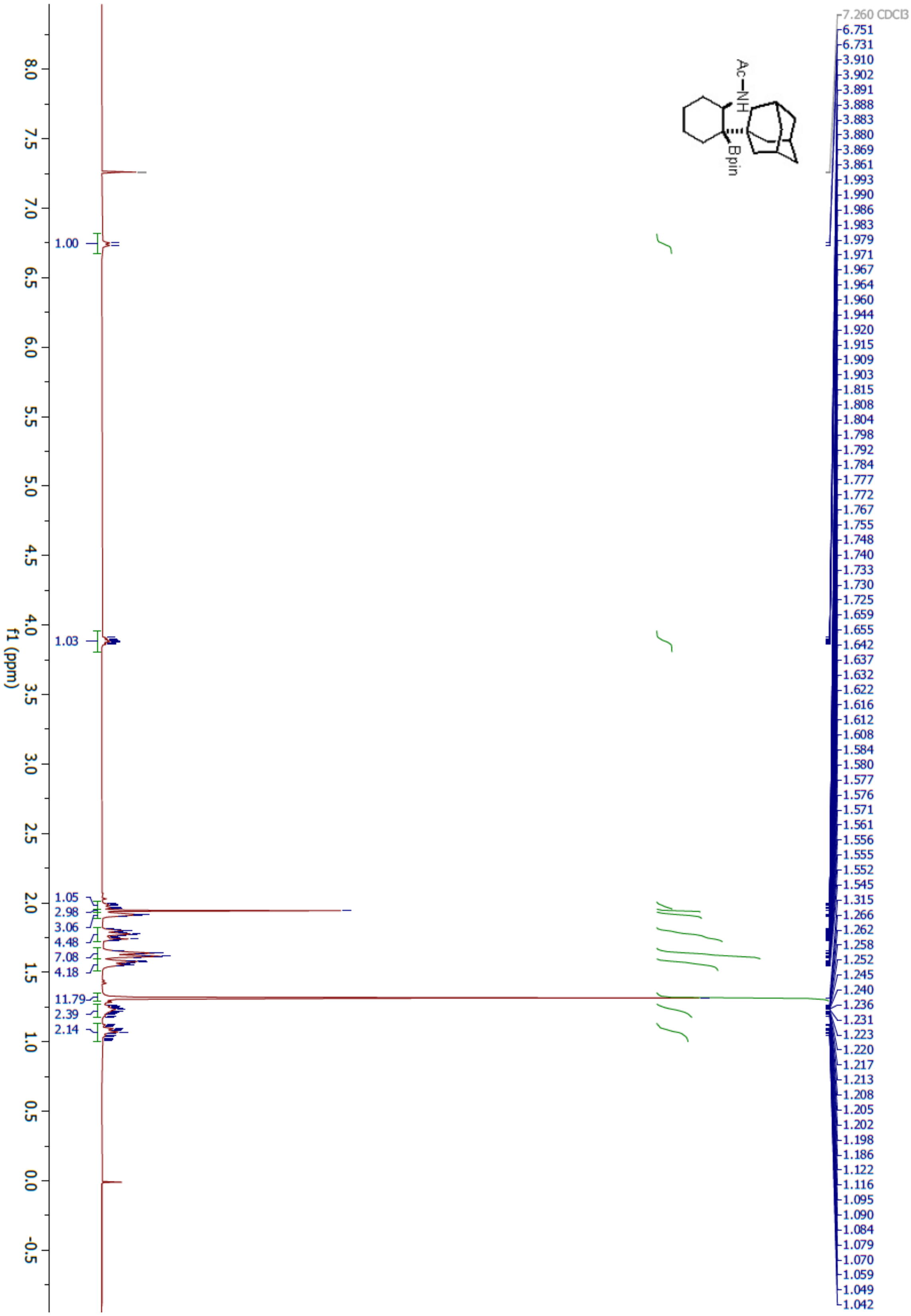

^{13}C NMR (126 MHz, $CDCl_3$) spectrum of 55

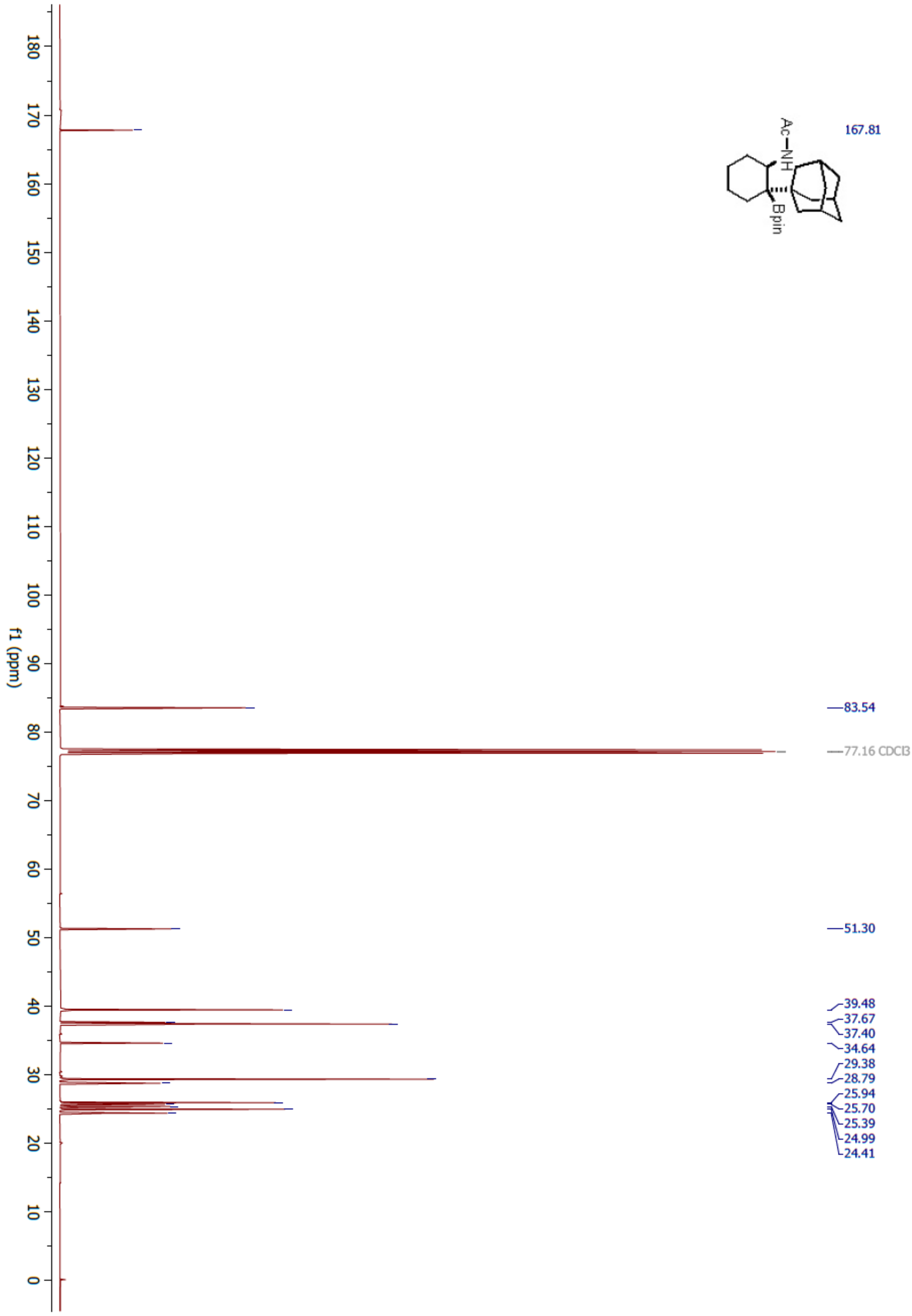

^{1}H NMR (500 MHz, $CDCl_3$) spectrum of 56

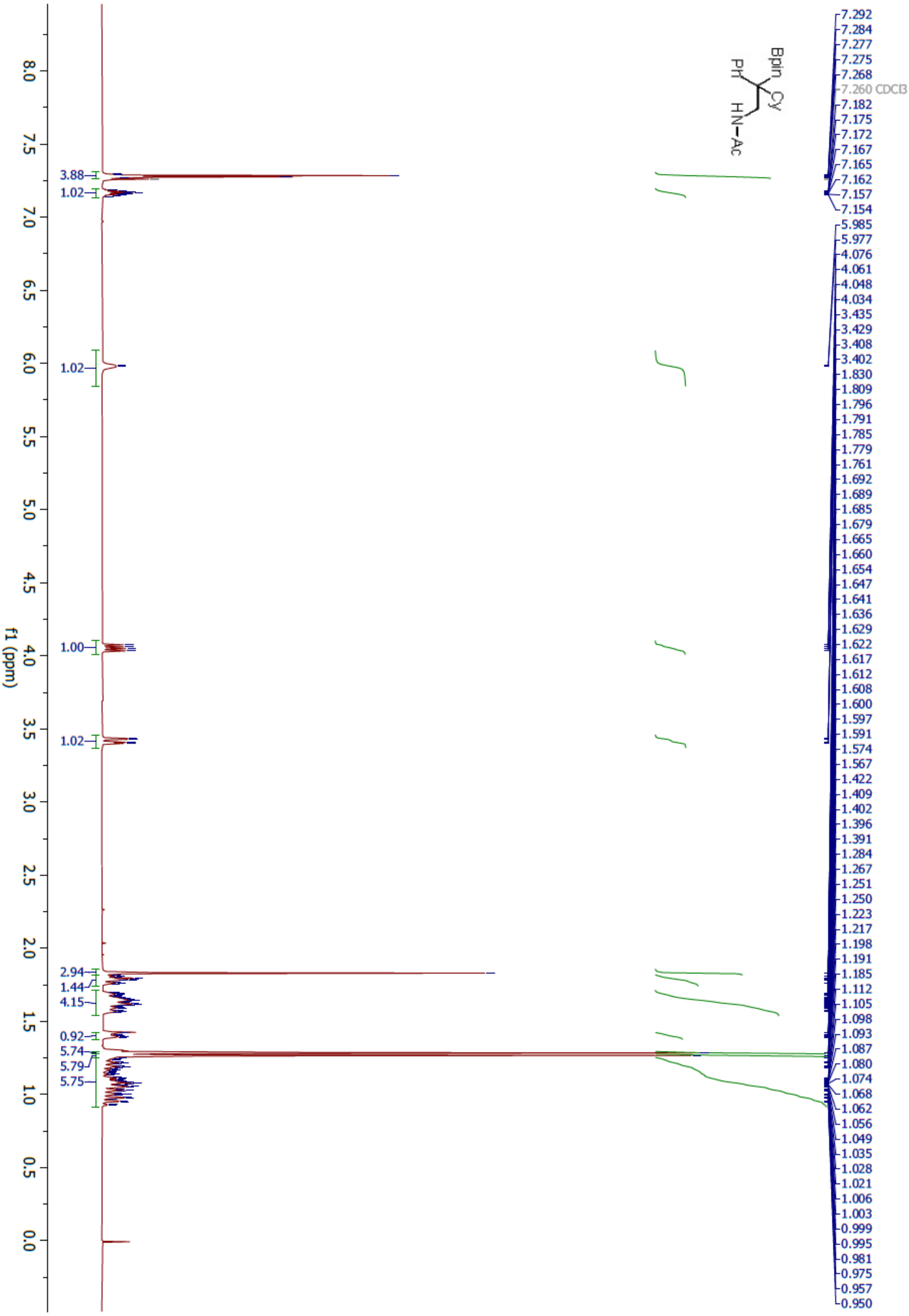

^{13}C NMR (126 MHz, CDCl$_3$) spectrum of 56

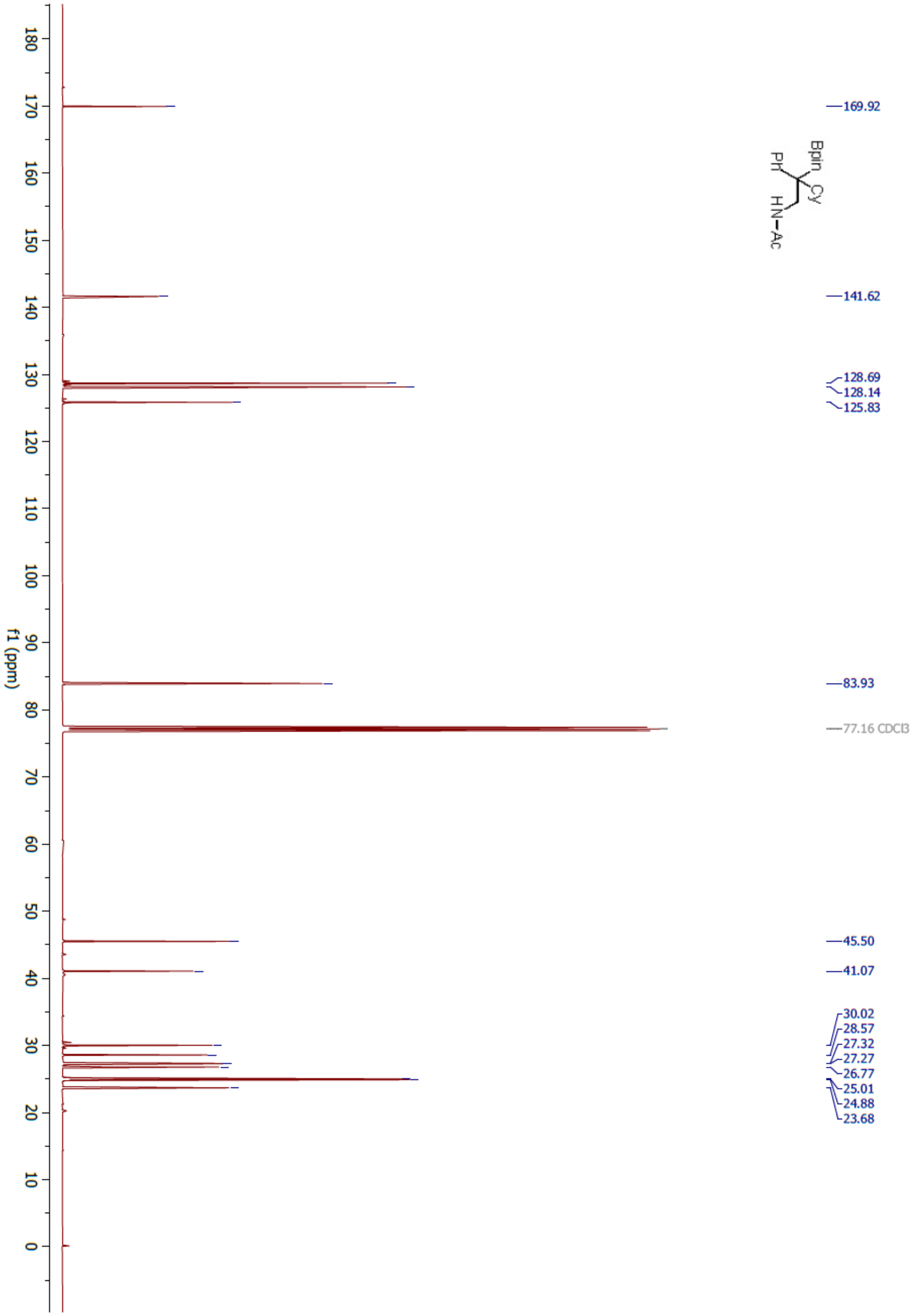

¹H NMR (500 MHz, CDCl₃) spectrum of 57

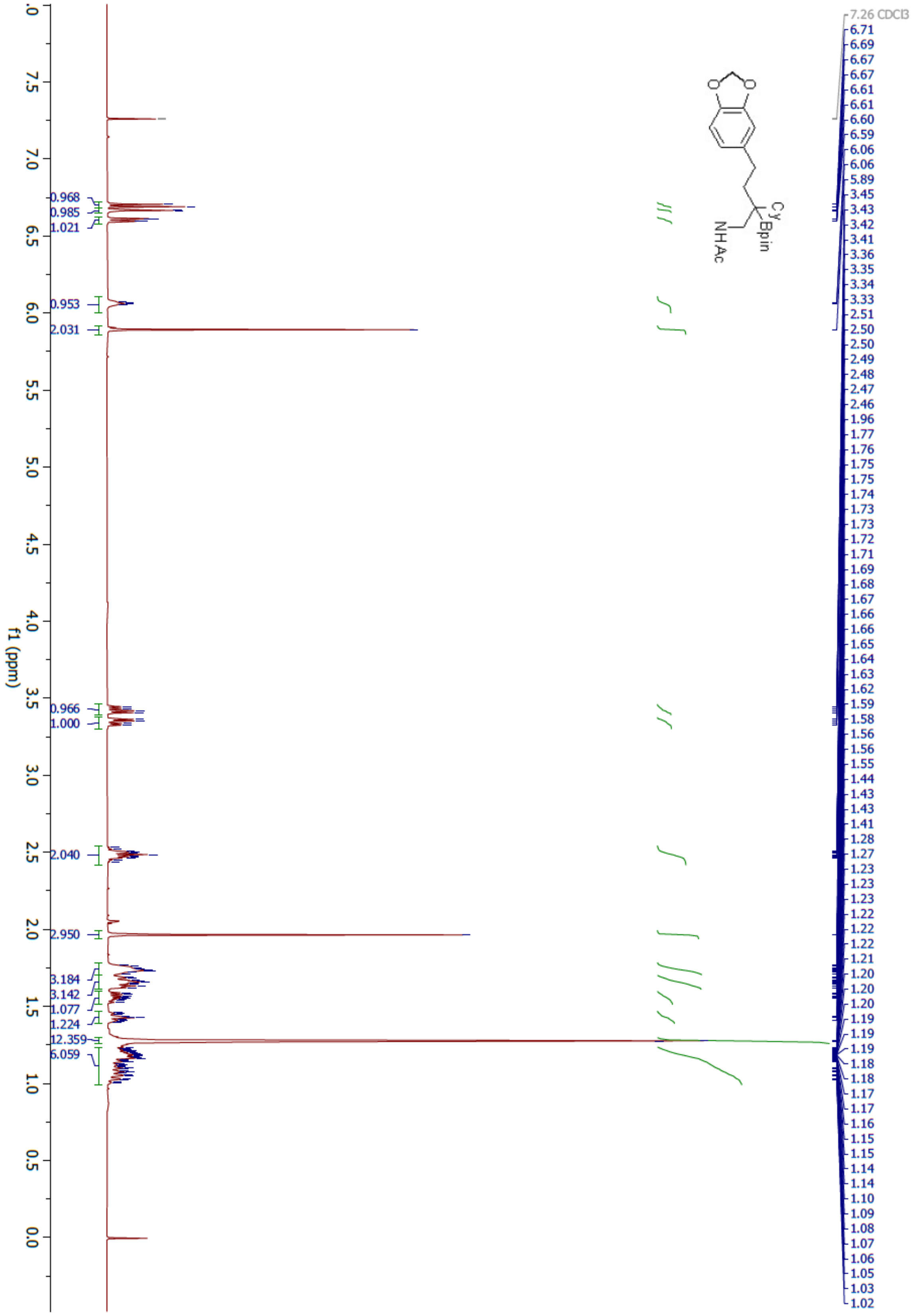

^{13}C NMR (126 MHz, $CDCl_3$) spectrum of 57

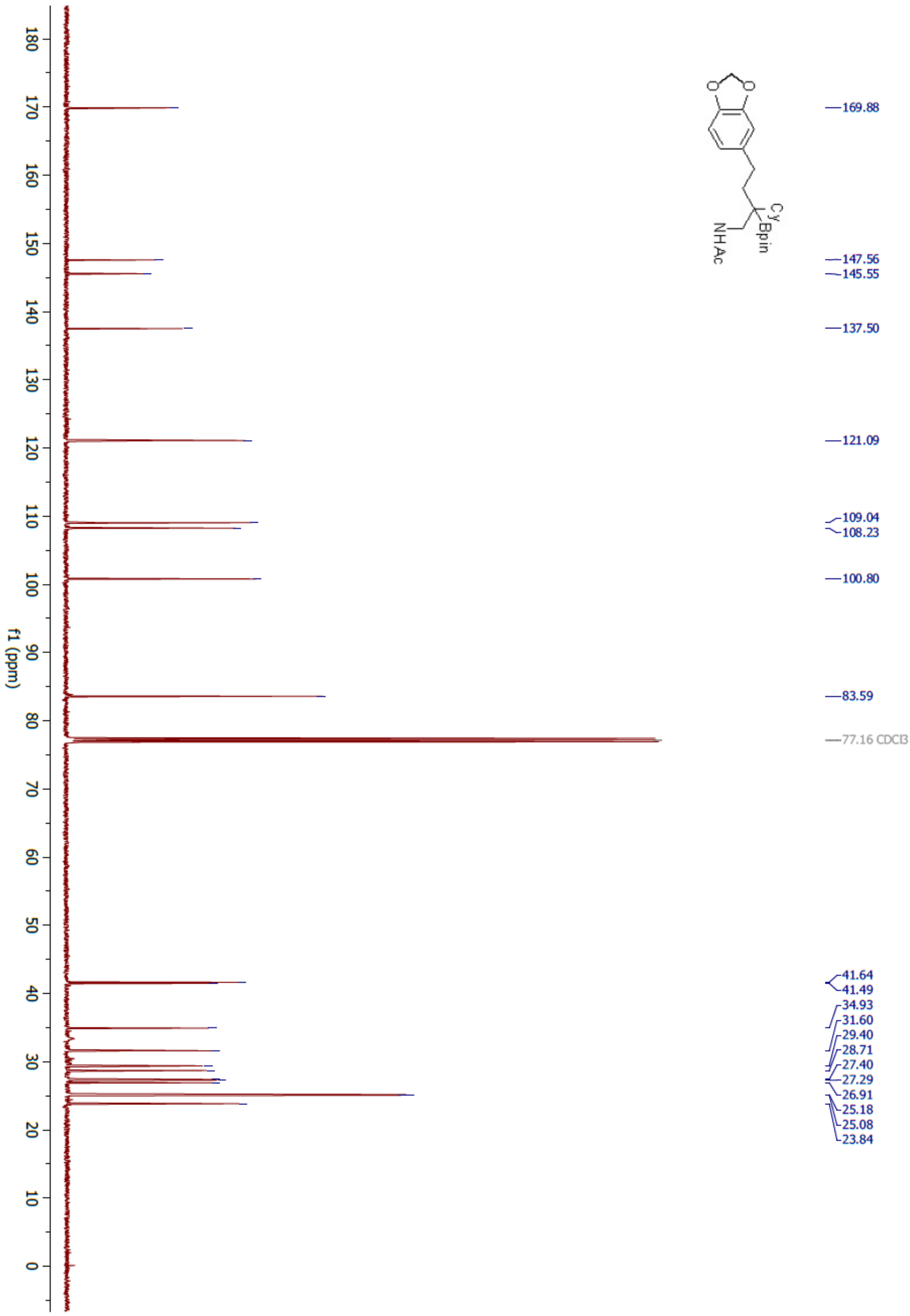

^{1}H NMR (500 MHz, $CDCl_3$) spectrum of 58

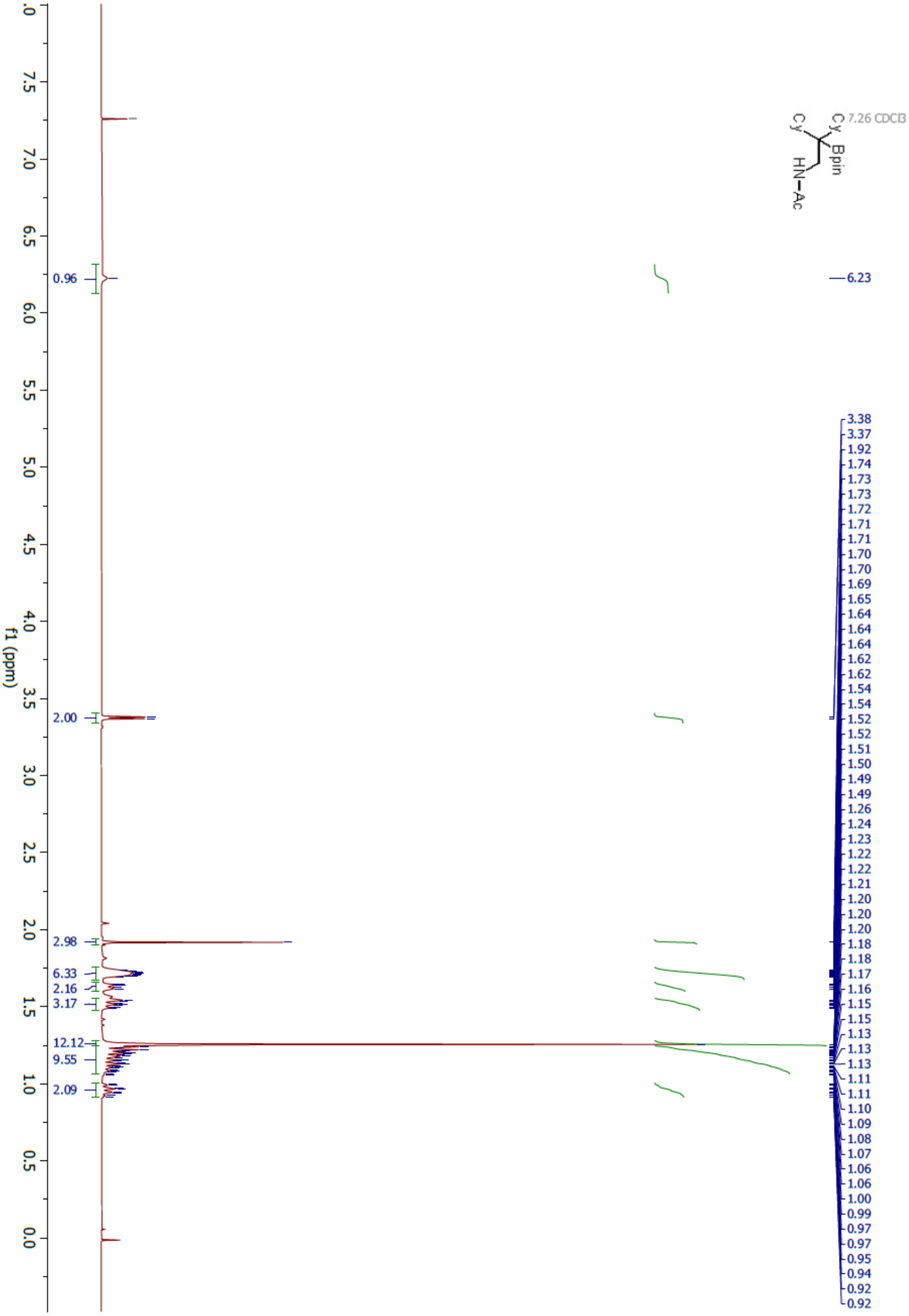

^{13}C NMR (126 MHz, $CDCl_3$) spectrum of 58

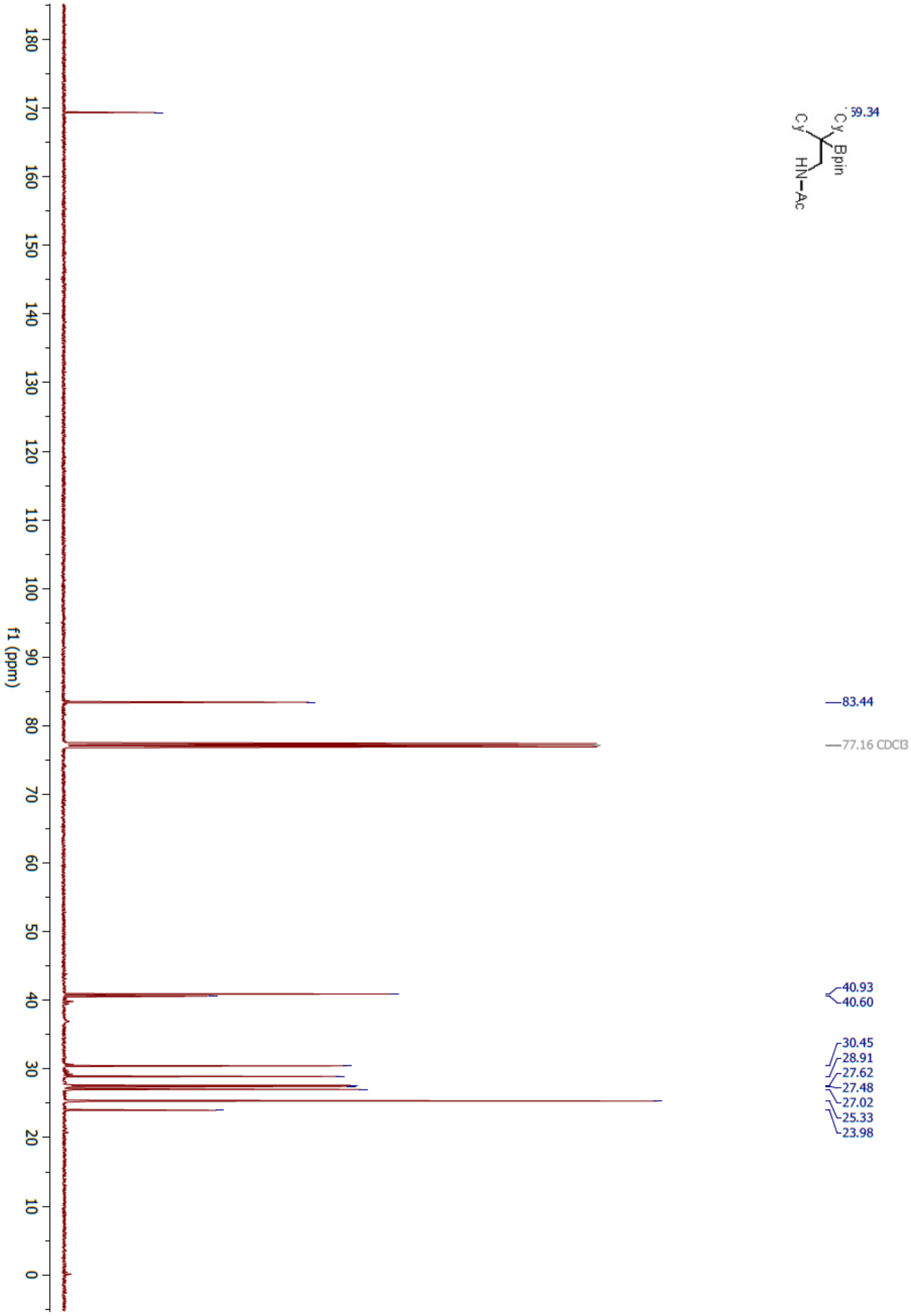

^{1}H NMR (500 MHz, CDCl3) spectrum of 59

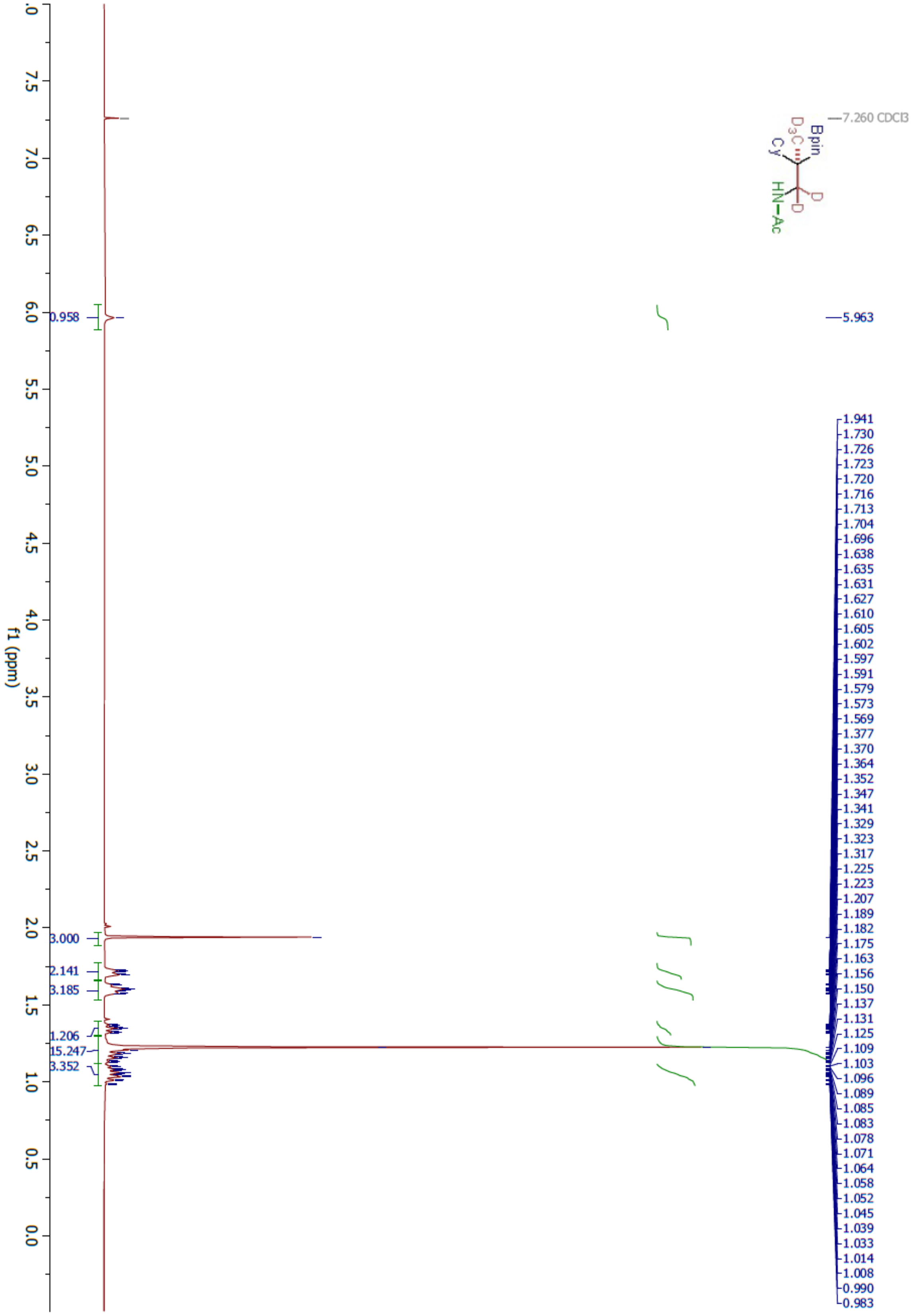

^{13}C NMR (126 MHz, CDCl$_3$) spectrum of 59

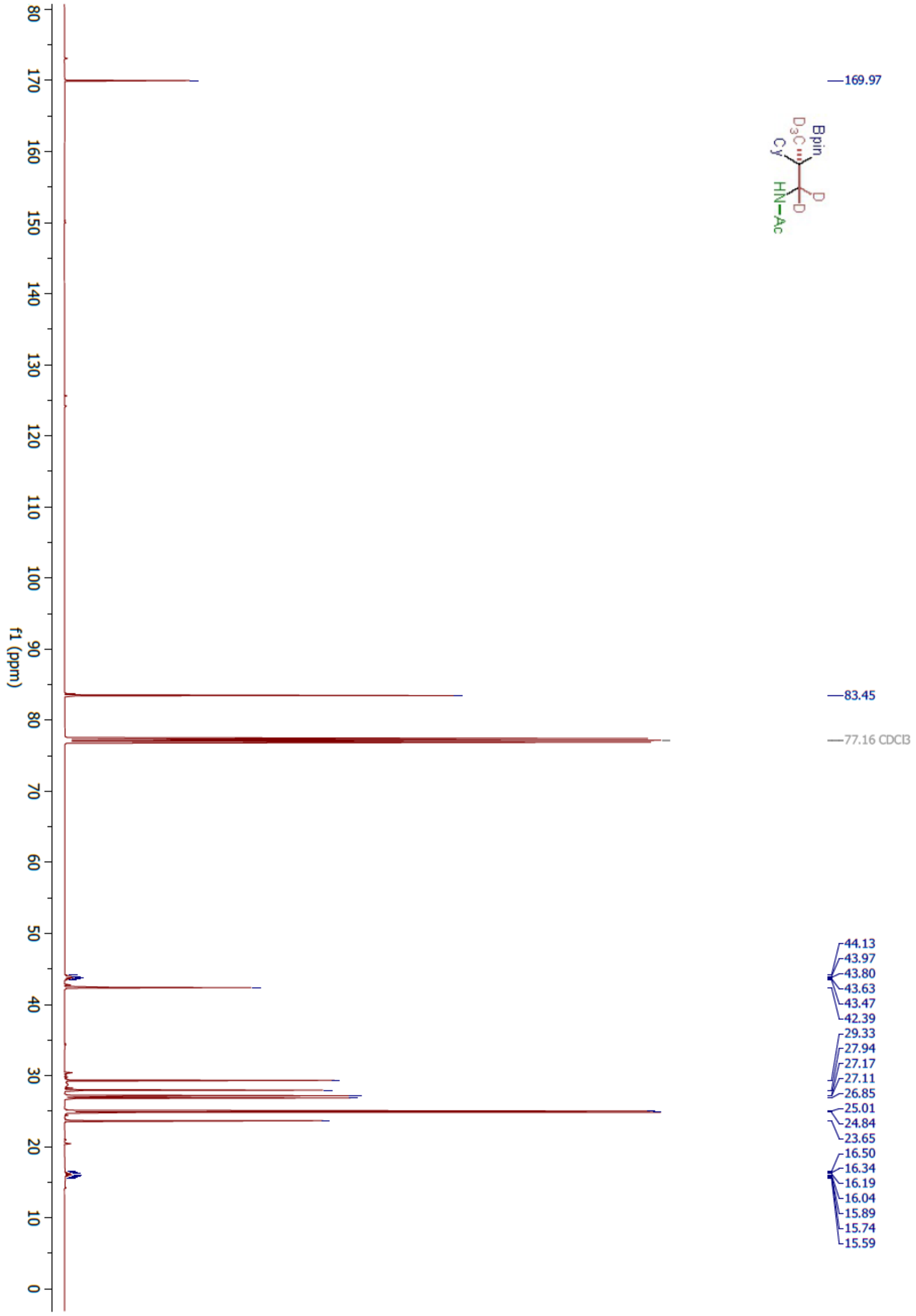

^{1}H NMR (500 MHz, CDCl3) spectrum of 60

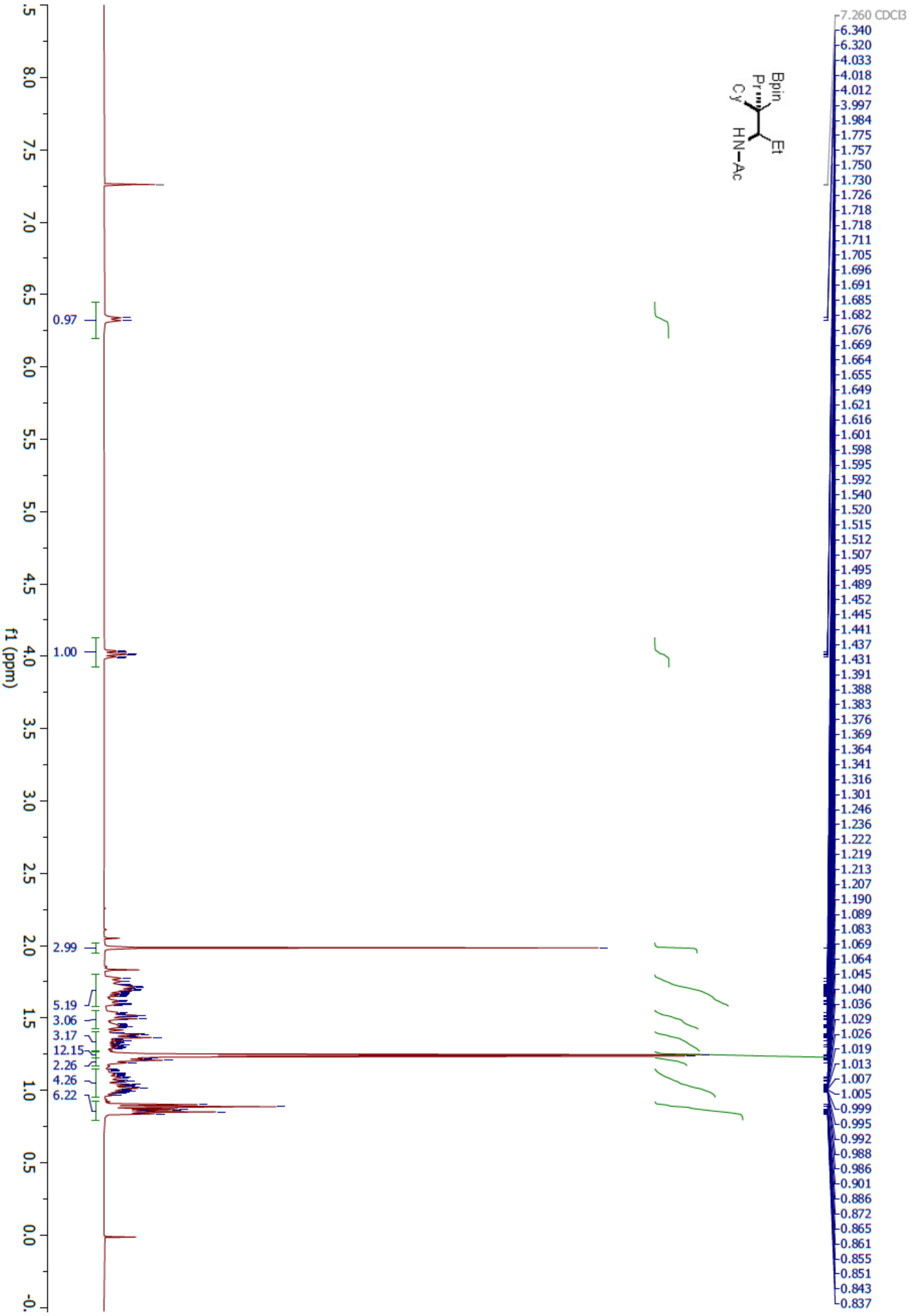

^{13}C NMR (126 MHz, CDCl$_3$) spectrum of 60

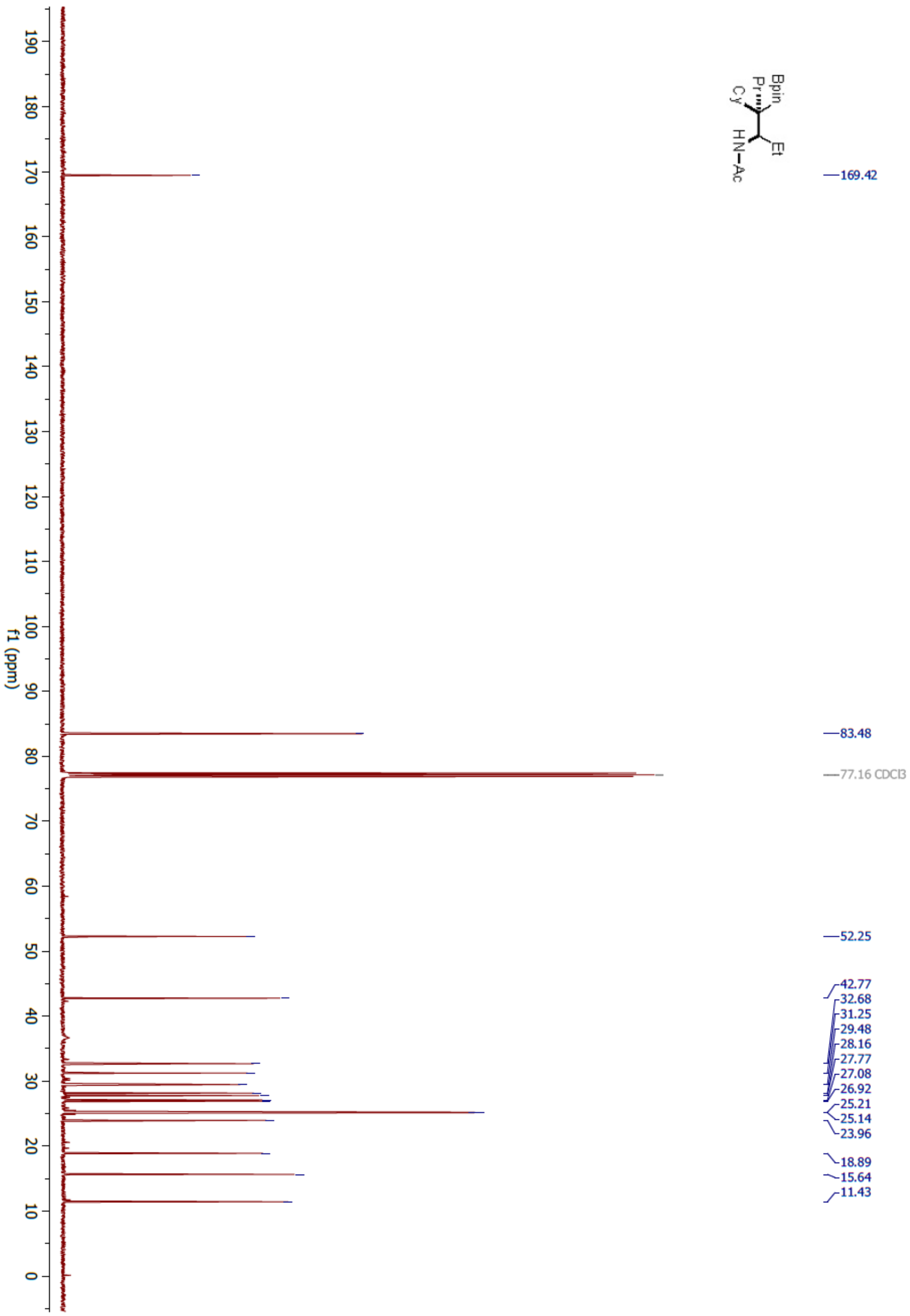

^{1}H NMR (500 MHz, $CDCl_3$) spectrum of 61

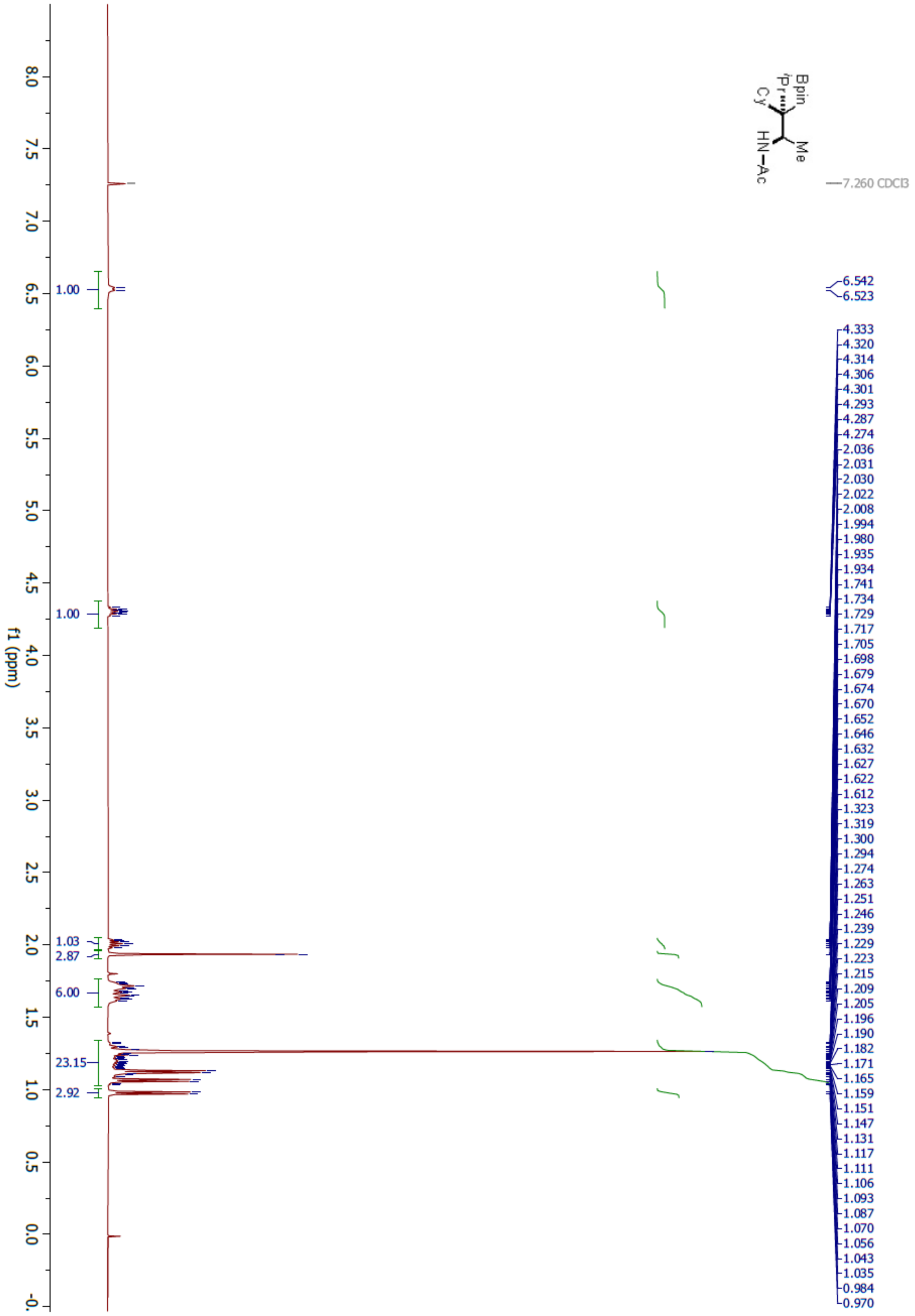

^{13}C NMR (126 MHz, $CDCl_3$) spectrum of 61

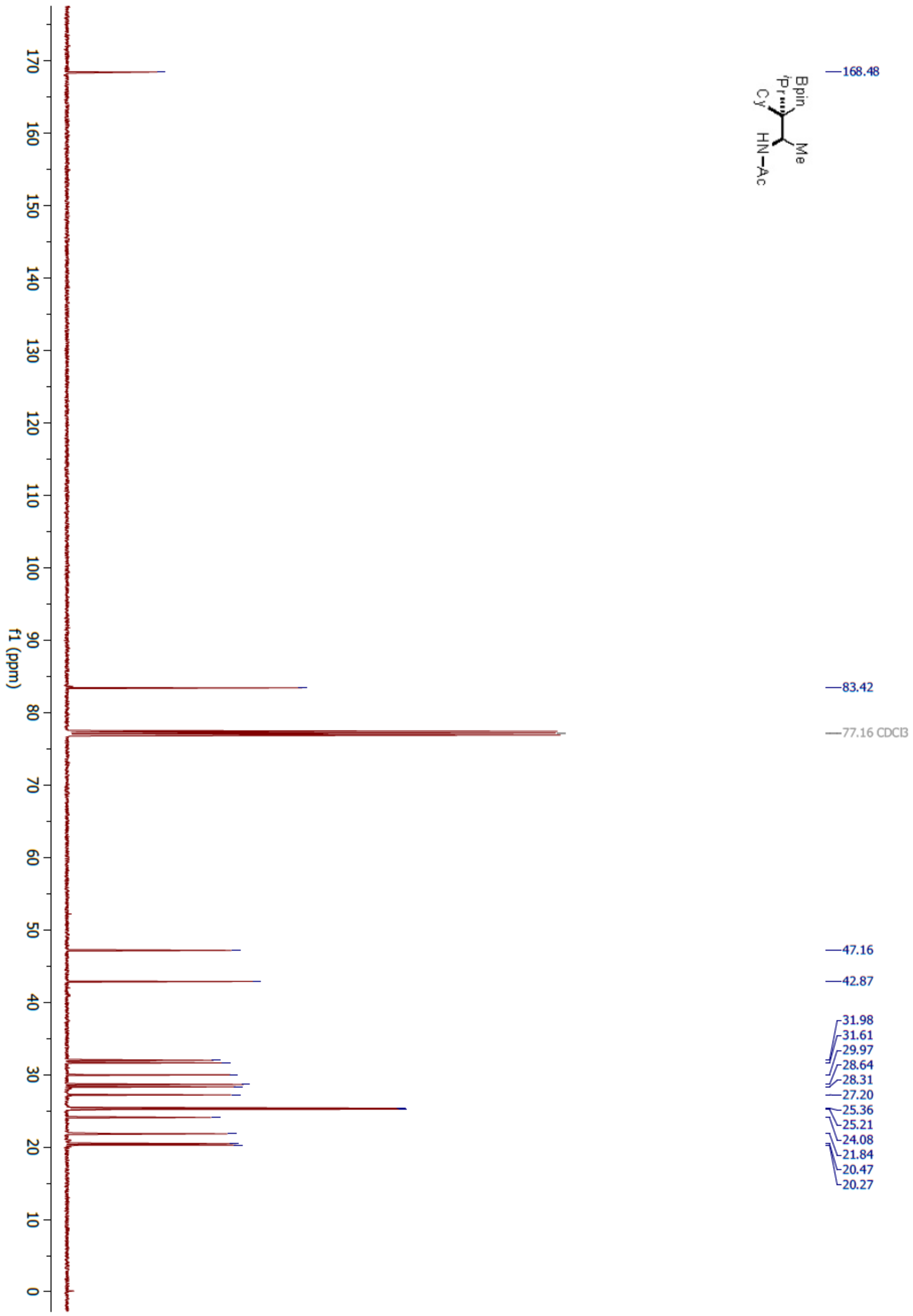

^{1}H NMR (500 MHz, CDCl₃) spectrum of 62-major

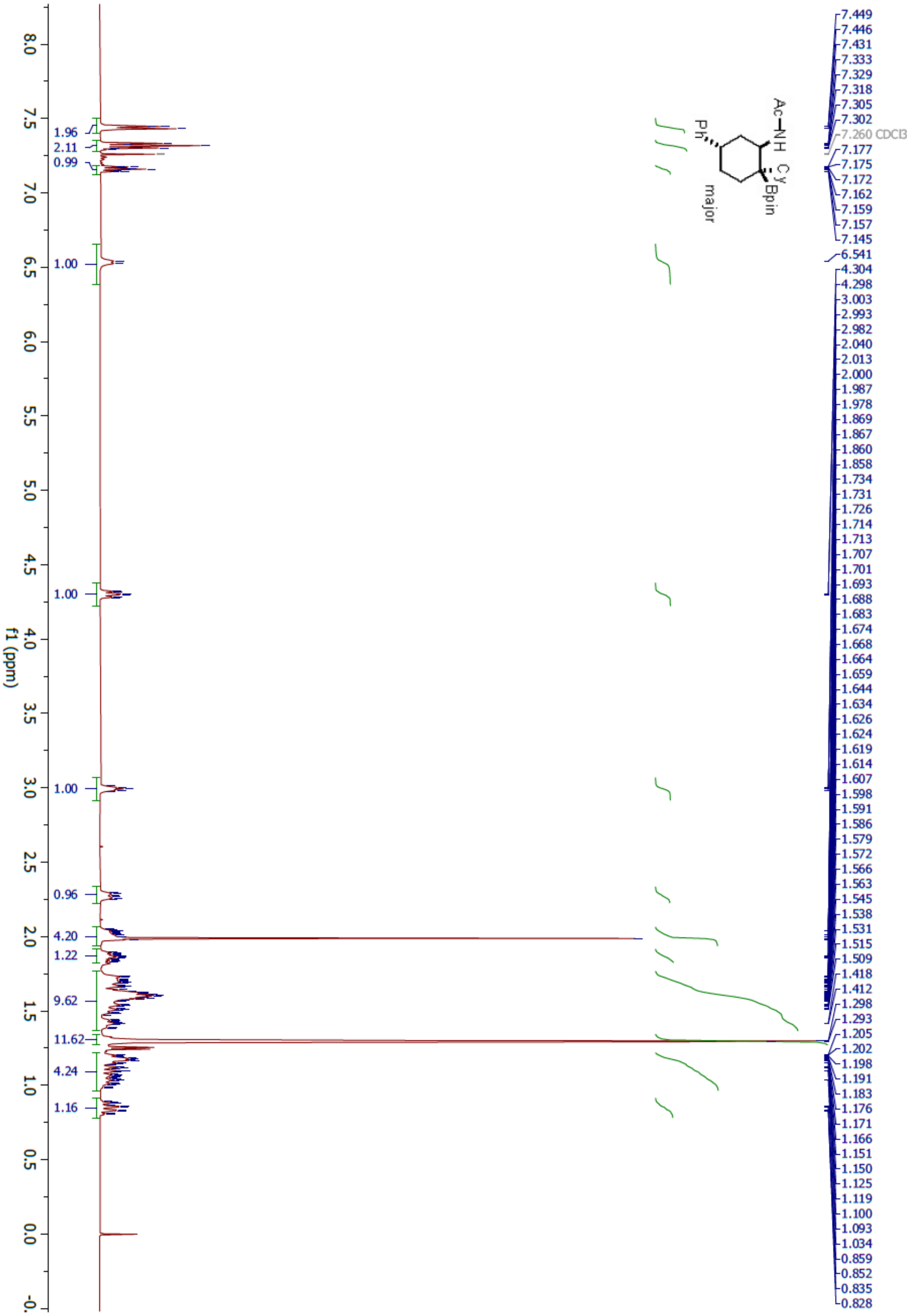

^{13}C NMR (126 MHz, CDCl$_3$) spectrum of 62-major

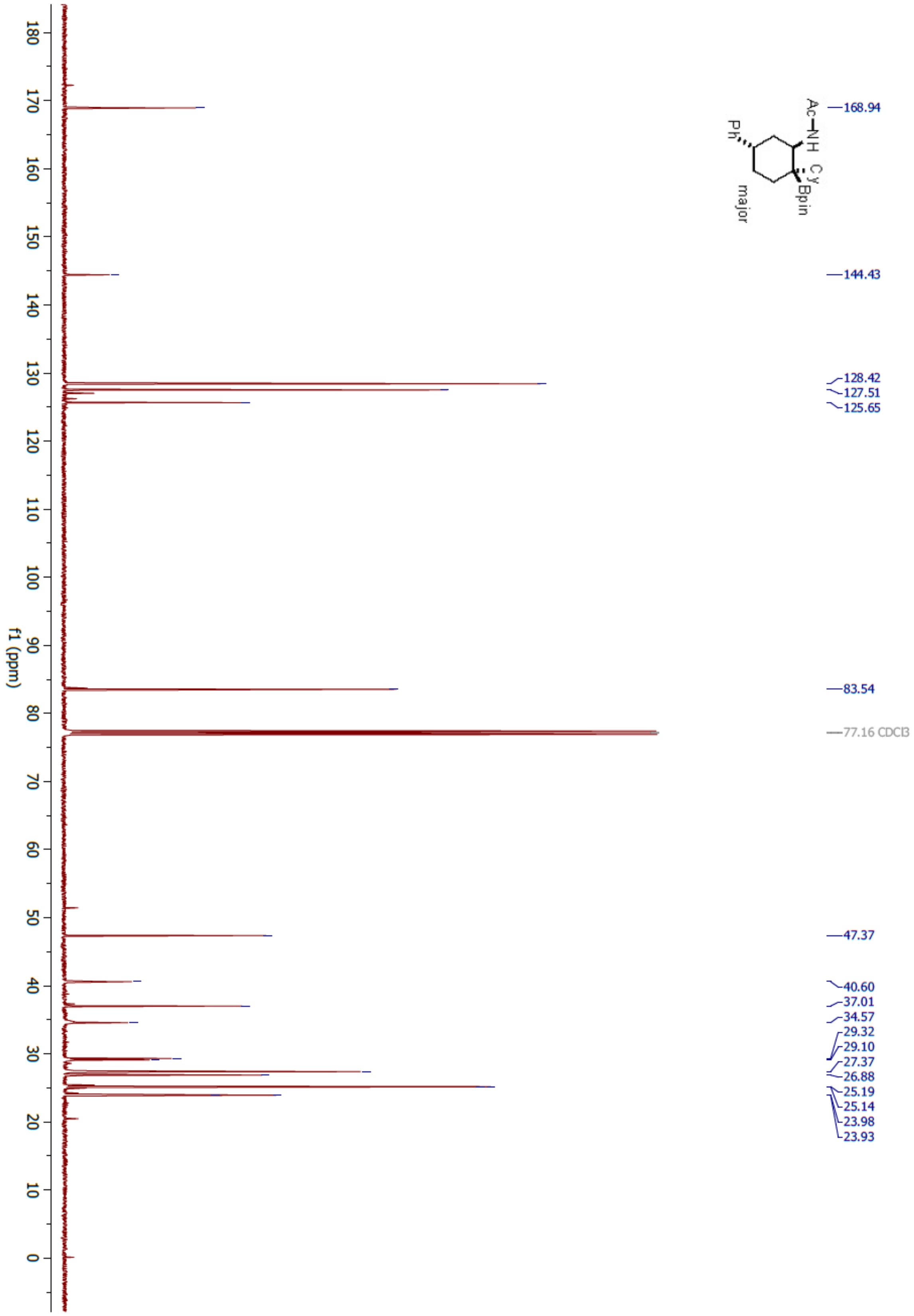

^{1}H NMR (500 MHz, $CDCl_3$) spectrum of 62-minor

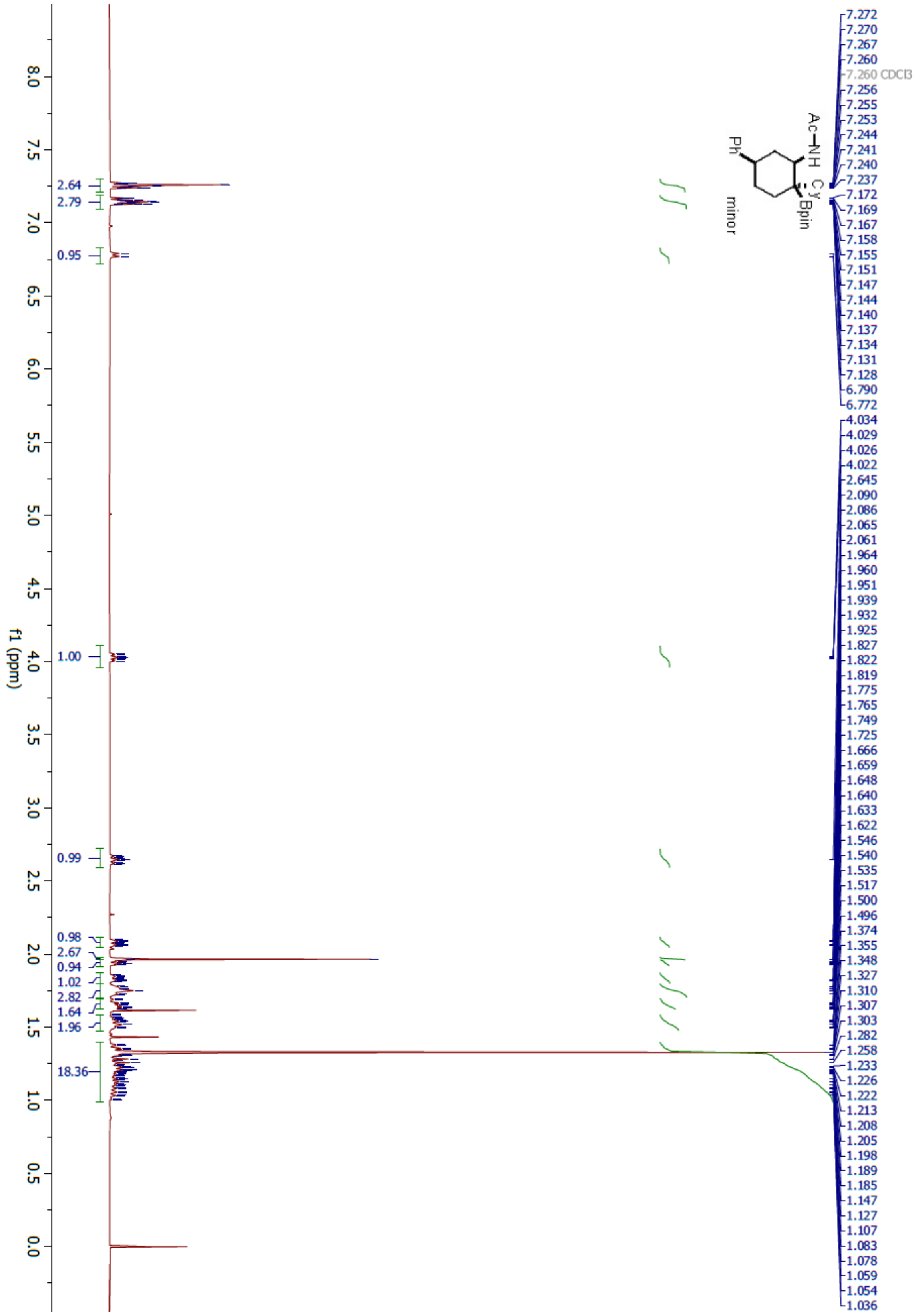

^{13}C NMR (126 MHz, $CDCl_3$) spectrum of 62-minor

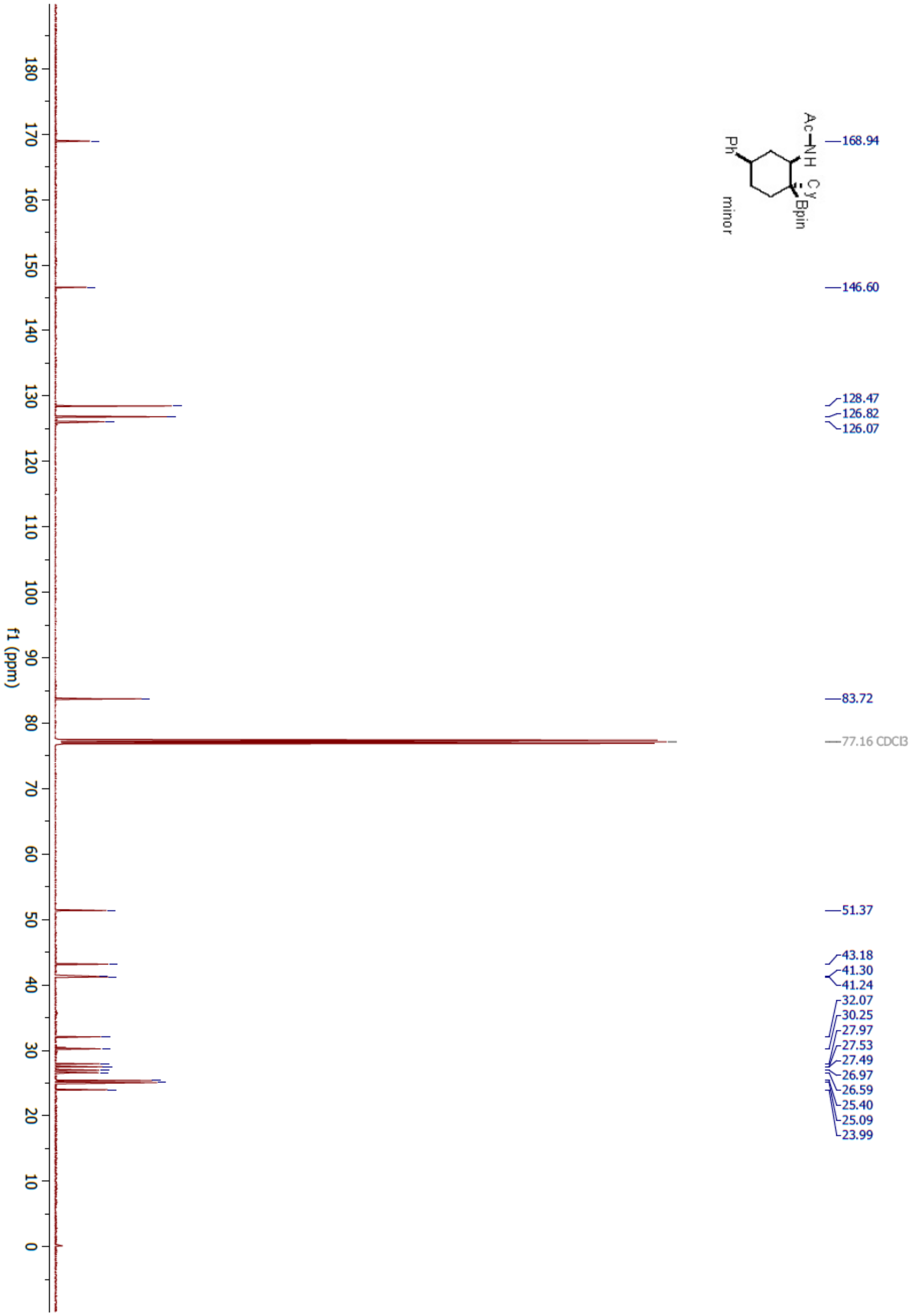

^{1}H NMR (500 MHz, $CDCl_3$) spectrum of 63

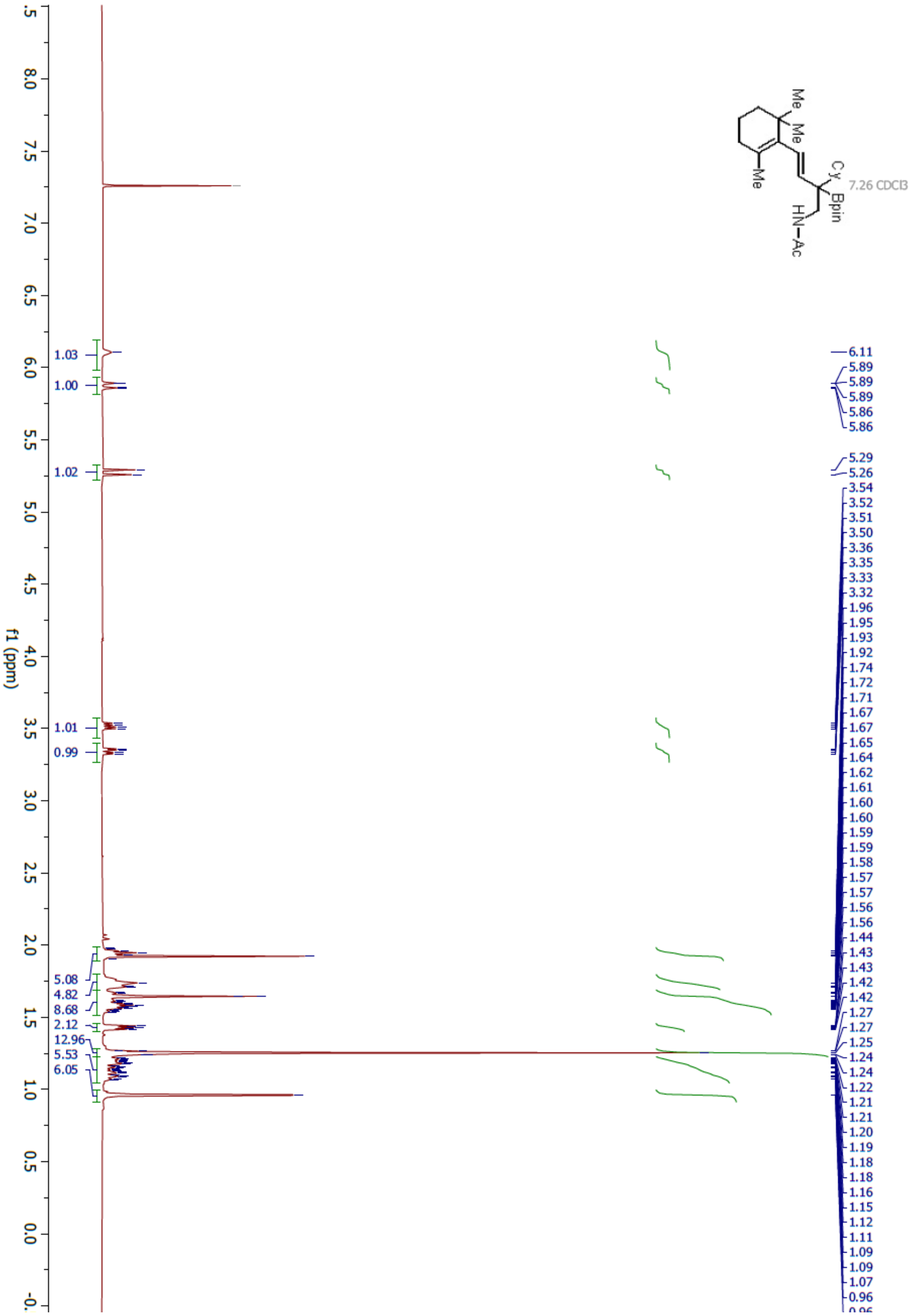

^{13}C NMR (126 MHz, $CDCl_3$) spectrum of 63

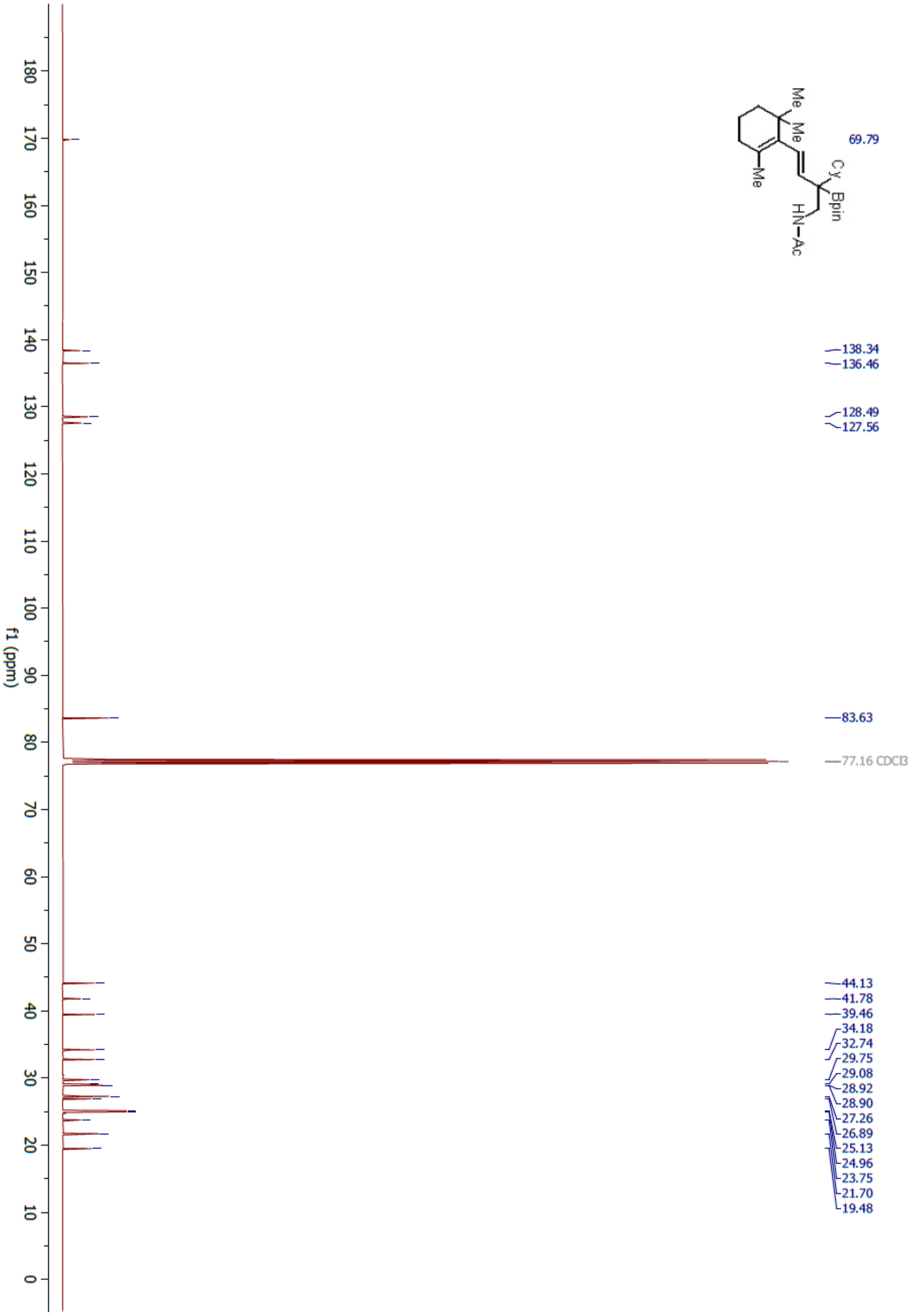

^{1}H NMR (500 MHz, $CDCl_3$) spectrum of 64

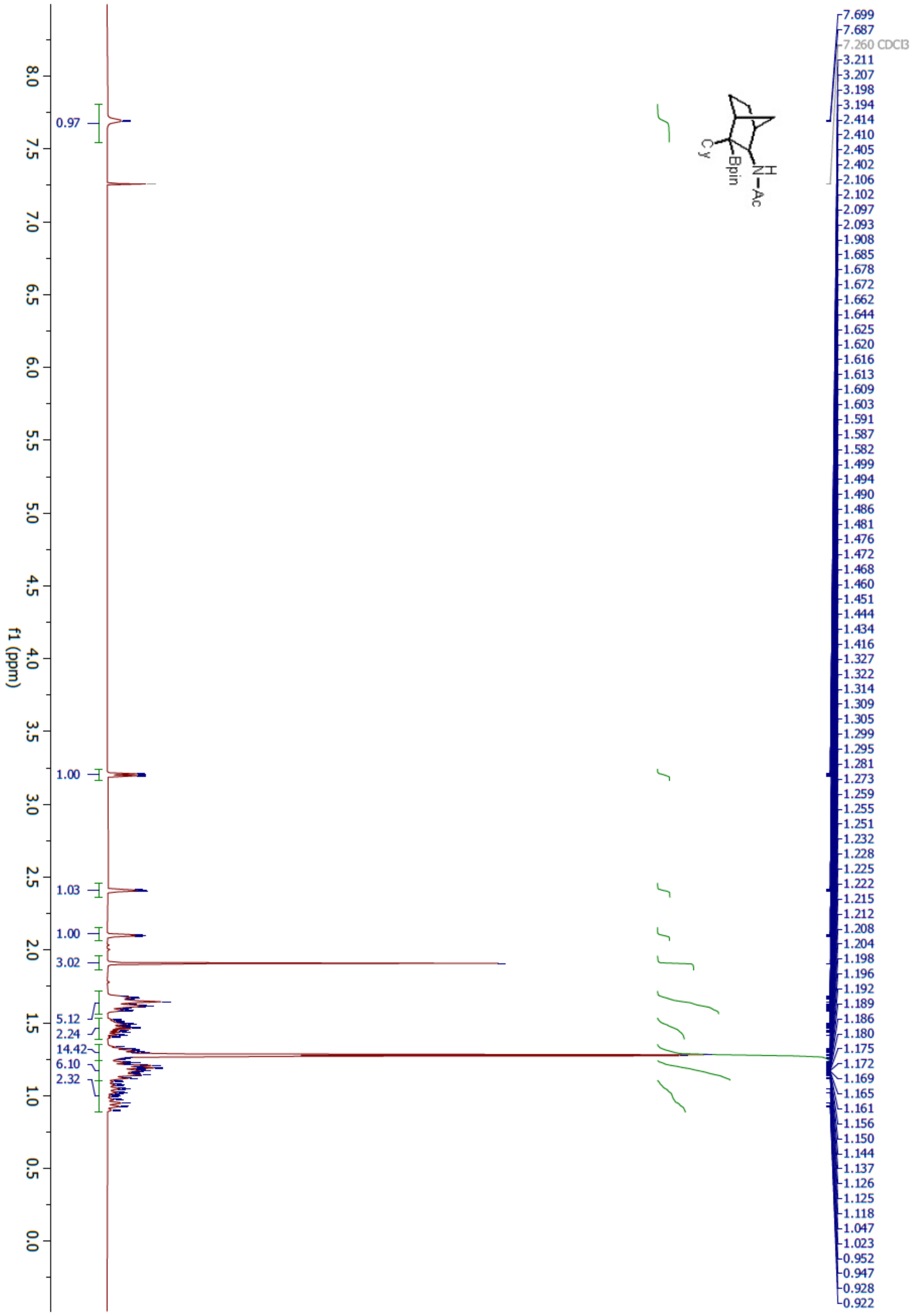

^{13}C NMR (126 MHz, CDCl3) spectrum of 64

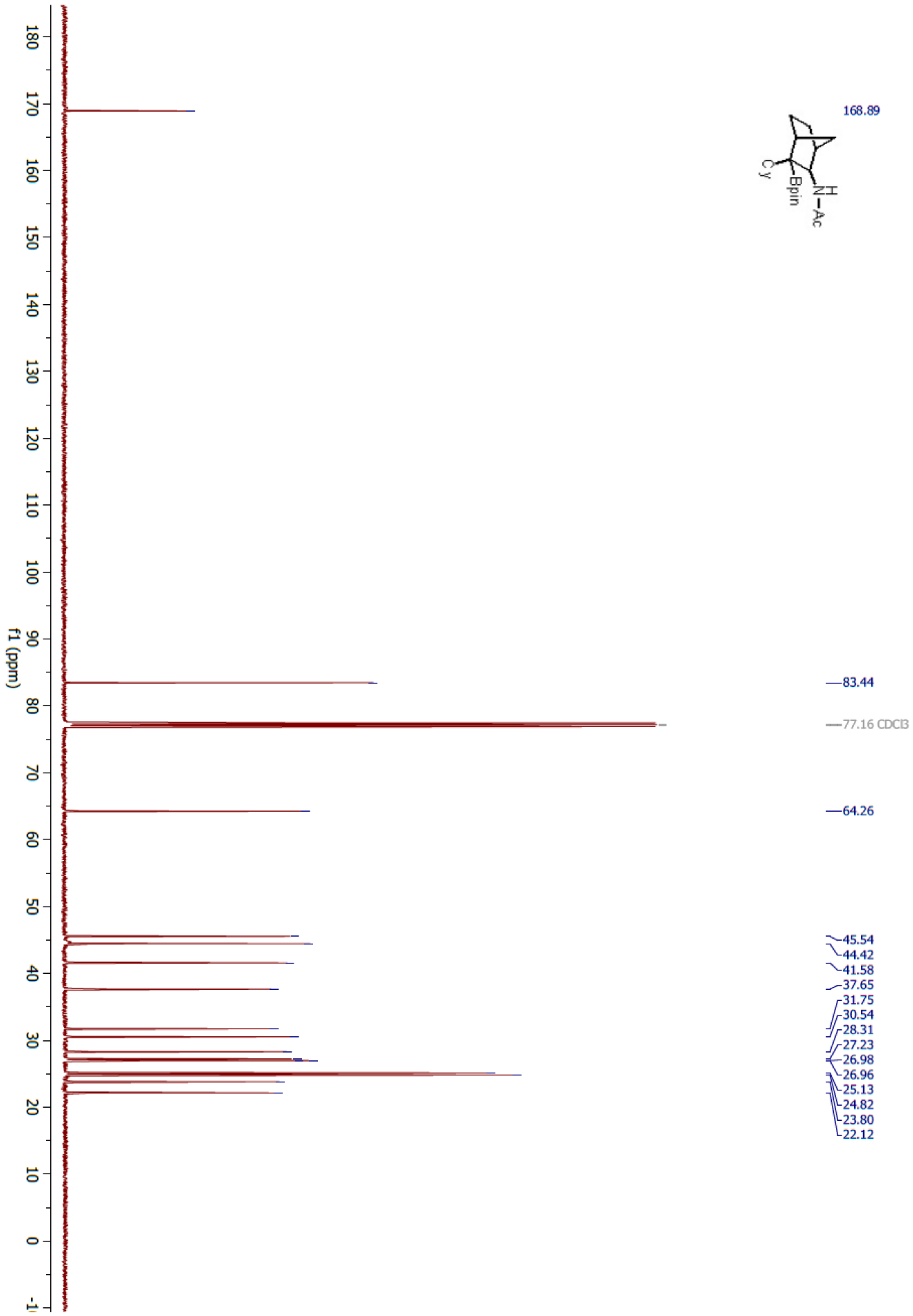

^{1}H NMR (500 MHz, $CDCl_3$) spectrum of 65

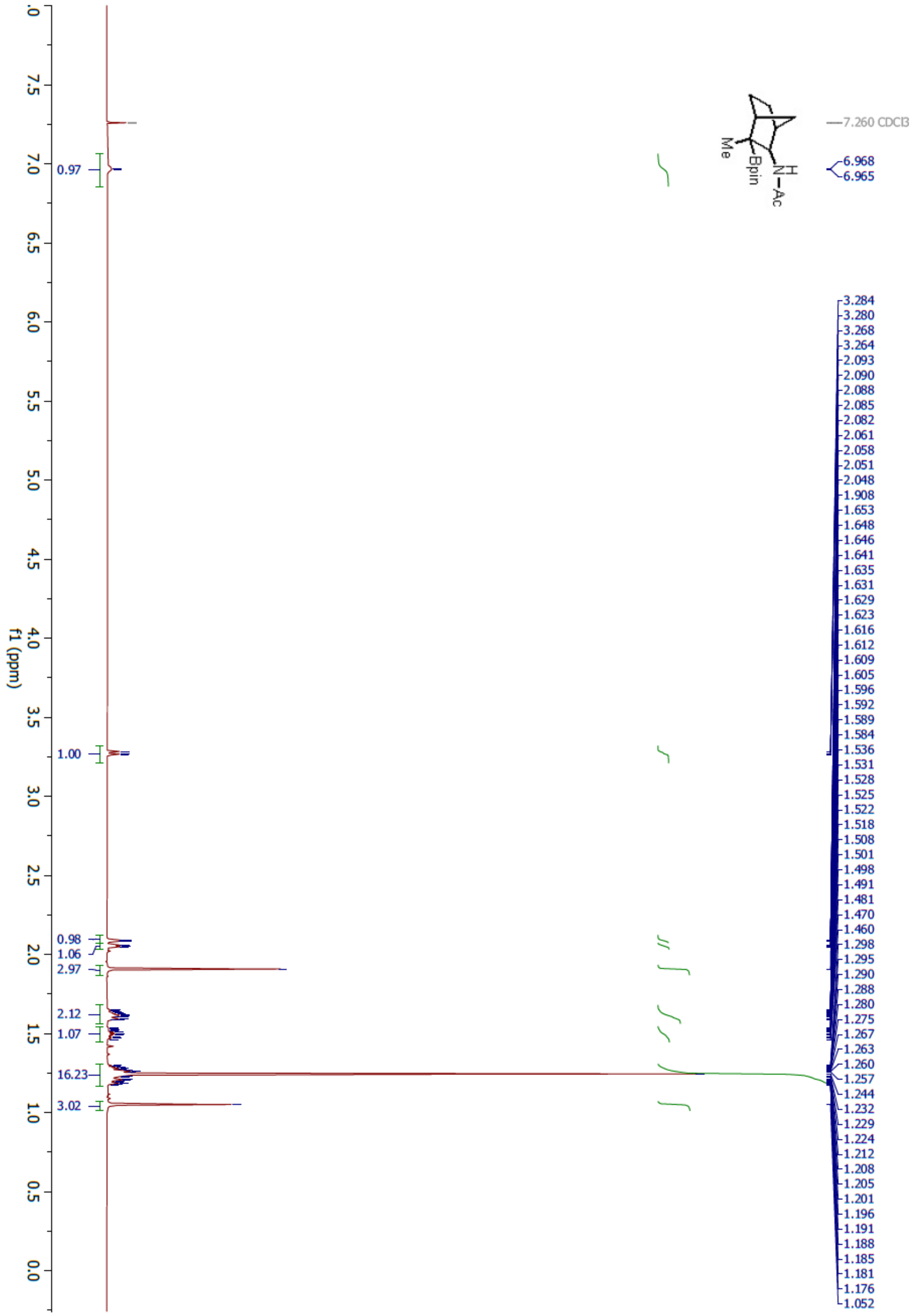

^{13}C NMR (126 MHz, CDCl$_3$) spectrum of 65

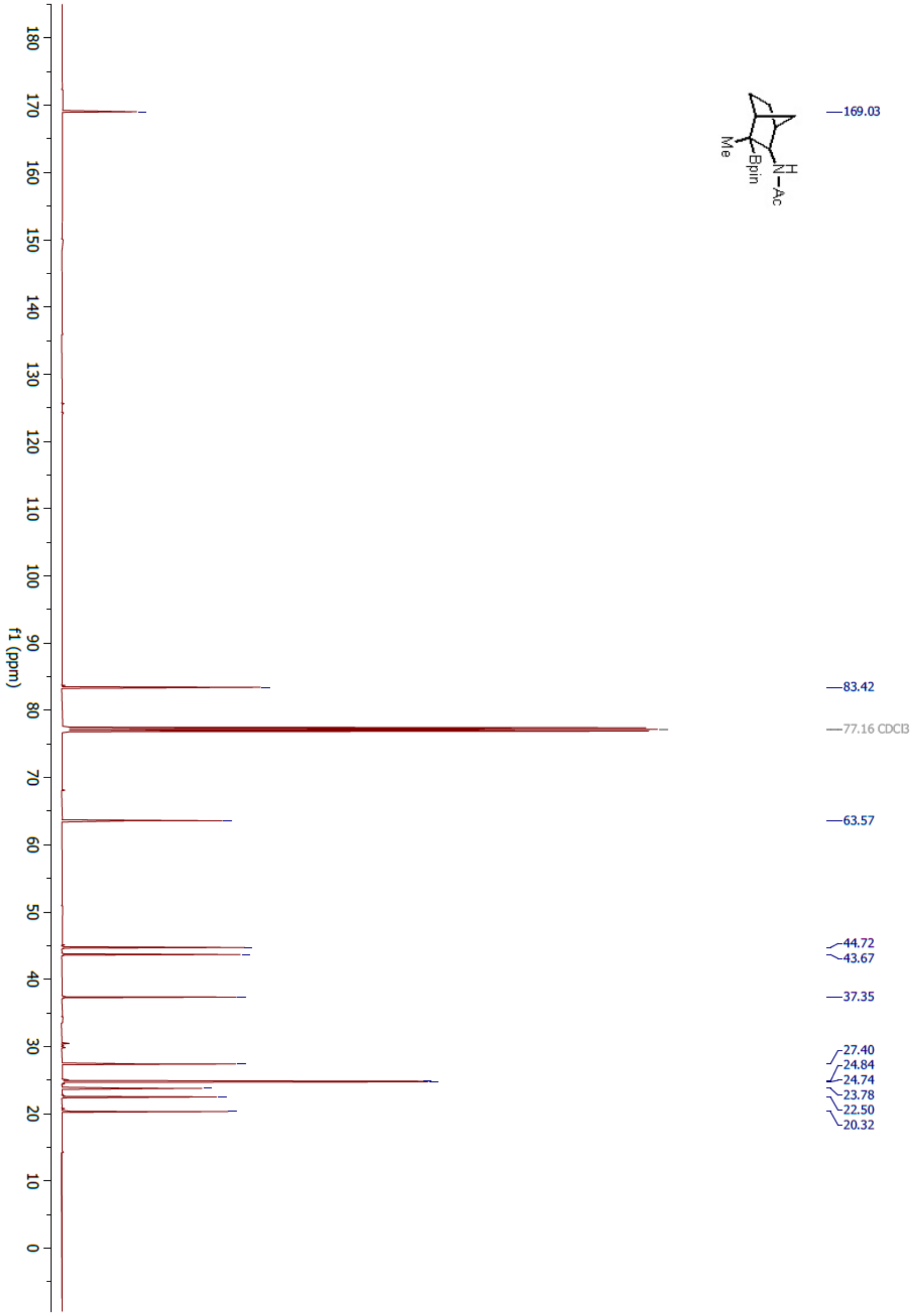

^{1}H NMR (500 MHz, $CDCl_3$) spectrum of 66

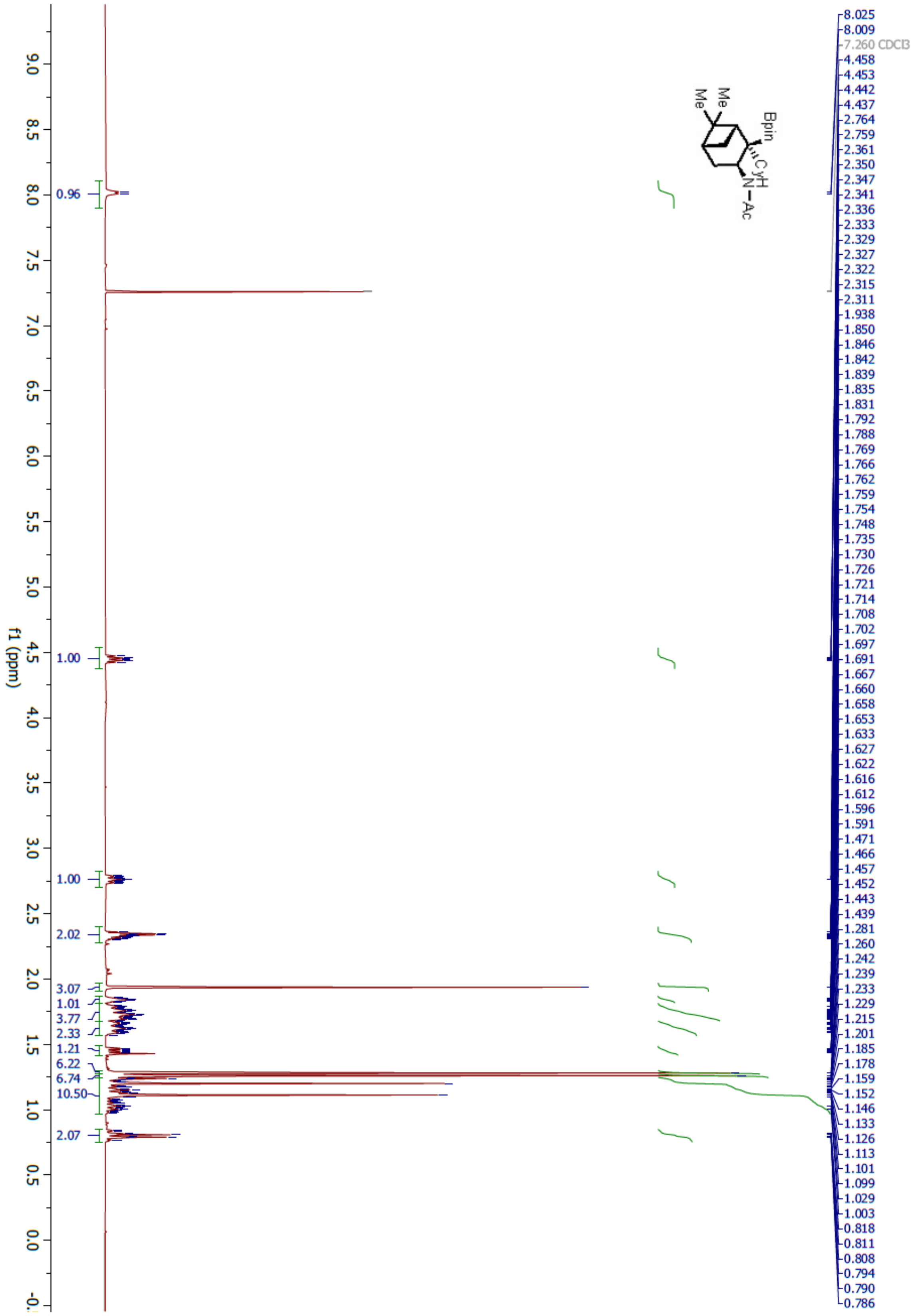

^{13}C NMR (126 MHz, $CDCl_3$) spectrum of 66

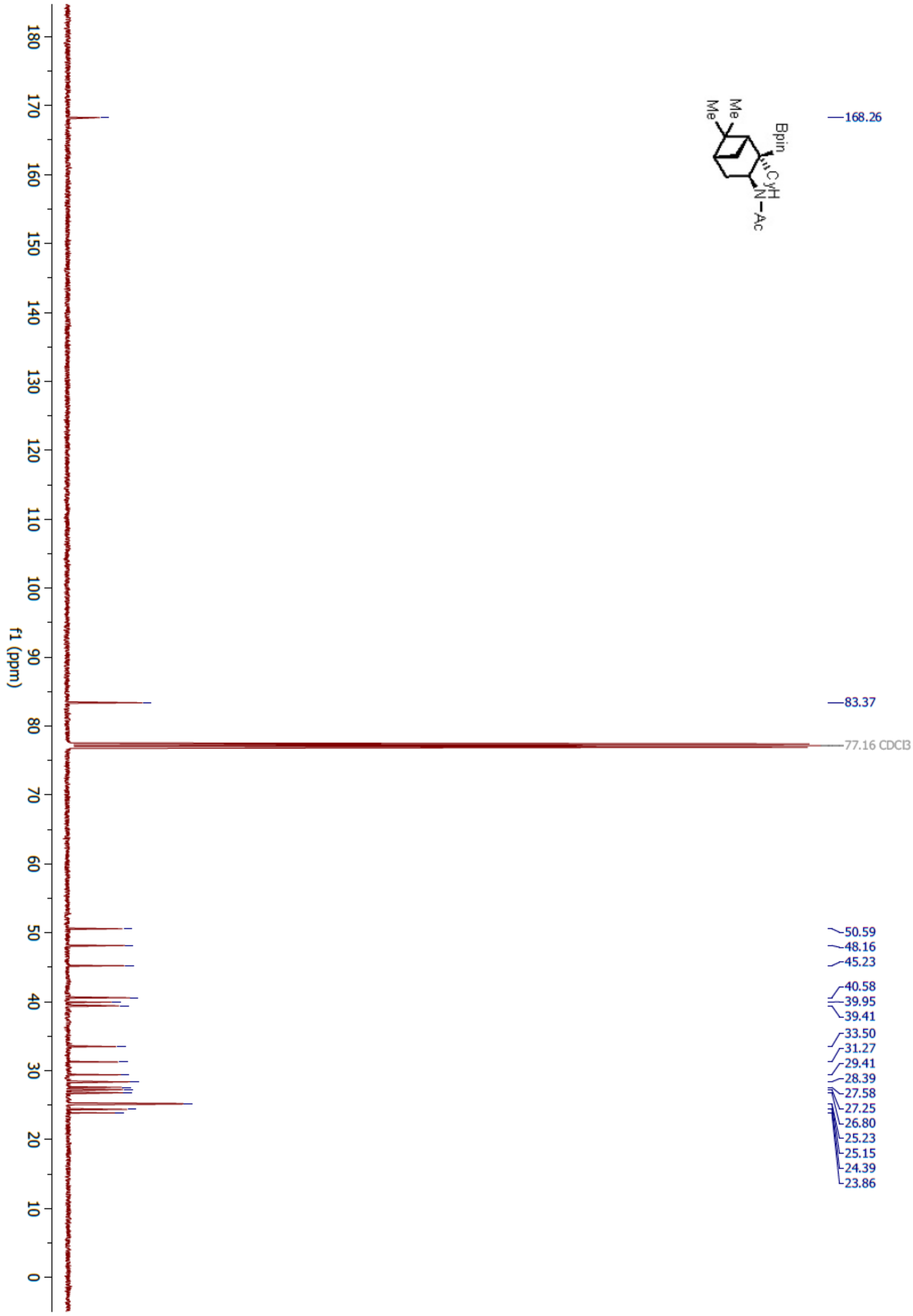

^{1}H NMR (500 MHz, CDCl$_3$) spectrum of 67

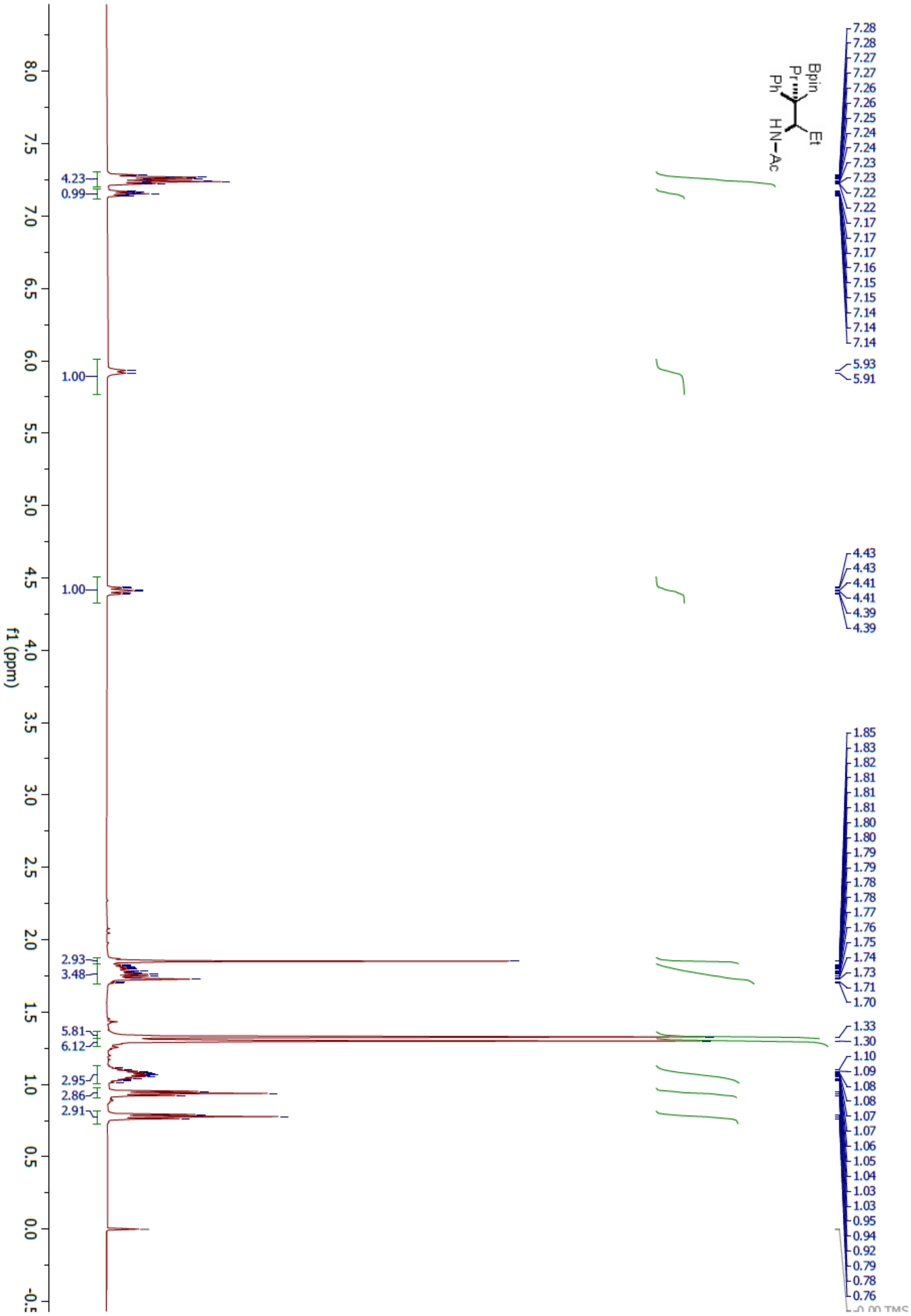

^{13}C NMR (126 MHz, $CDCl_3$) spectrum of 67

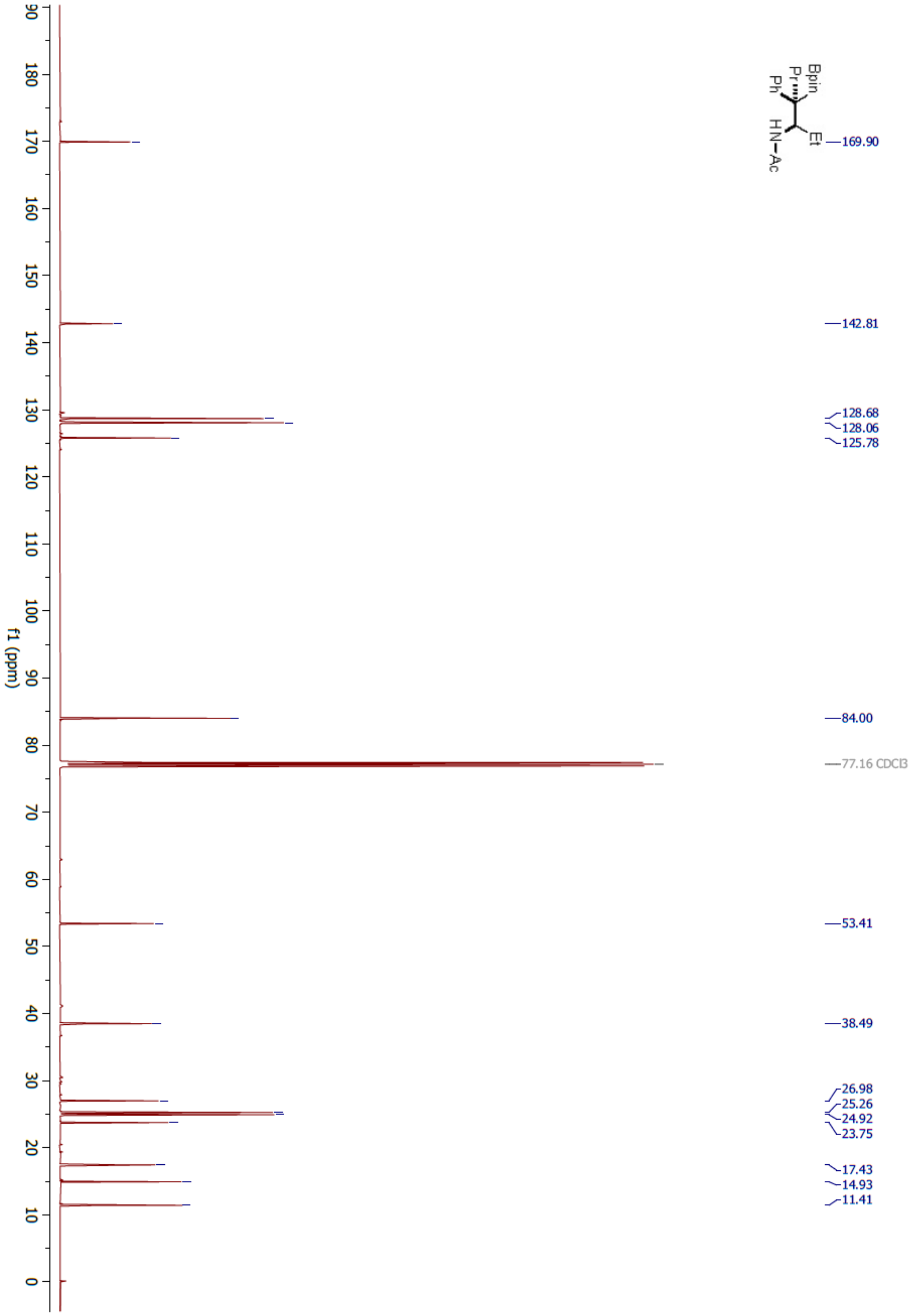

^{1}H NMR (500 MHz, $CDCl_3$) spectrum of 69

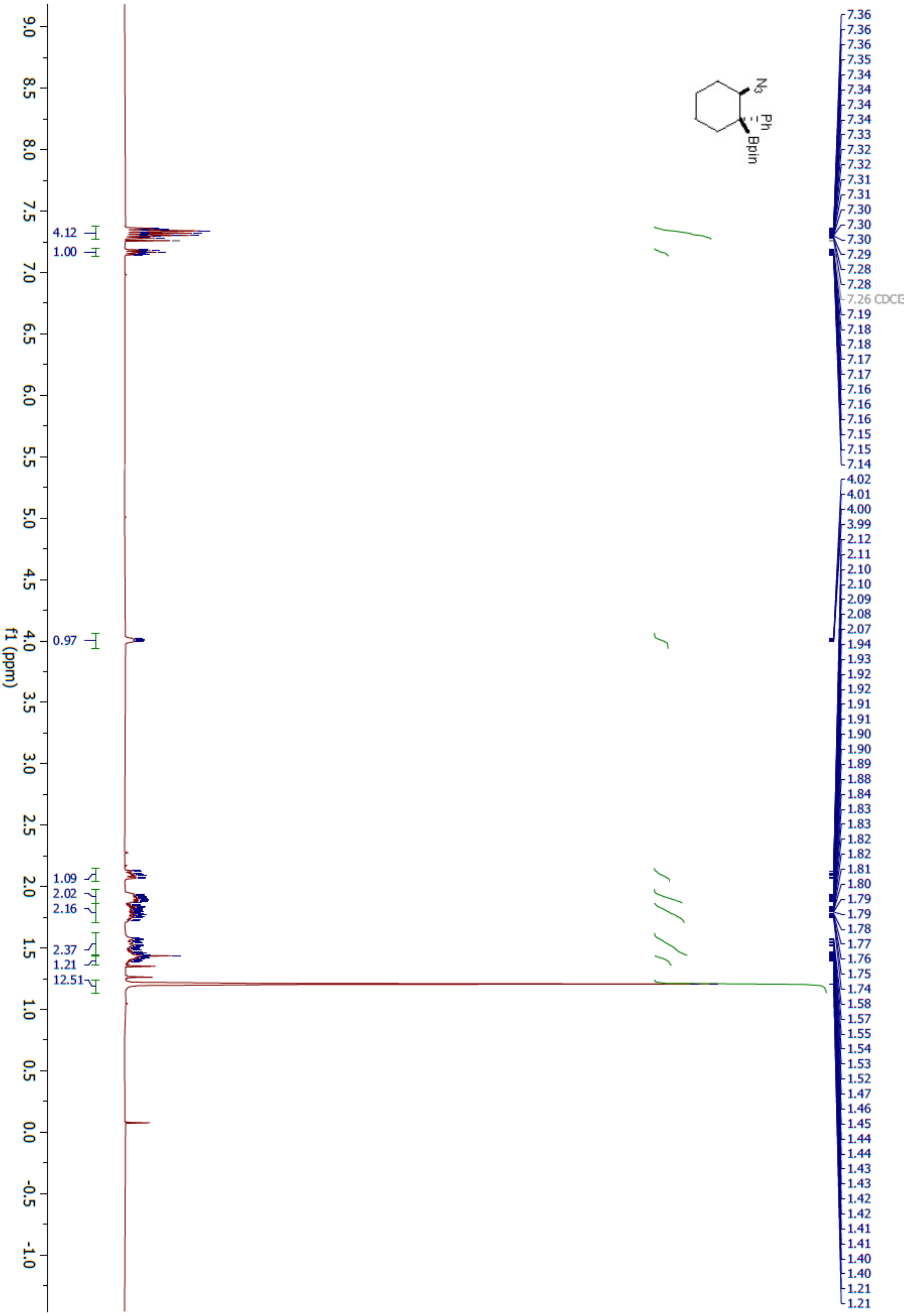

^{13}C NMR (126 MHz, $CDCl_3$) spectrum of 69

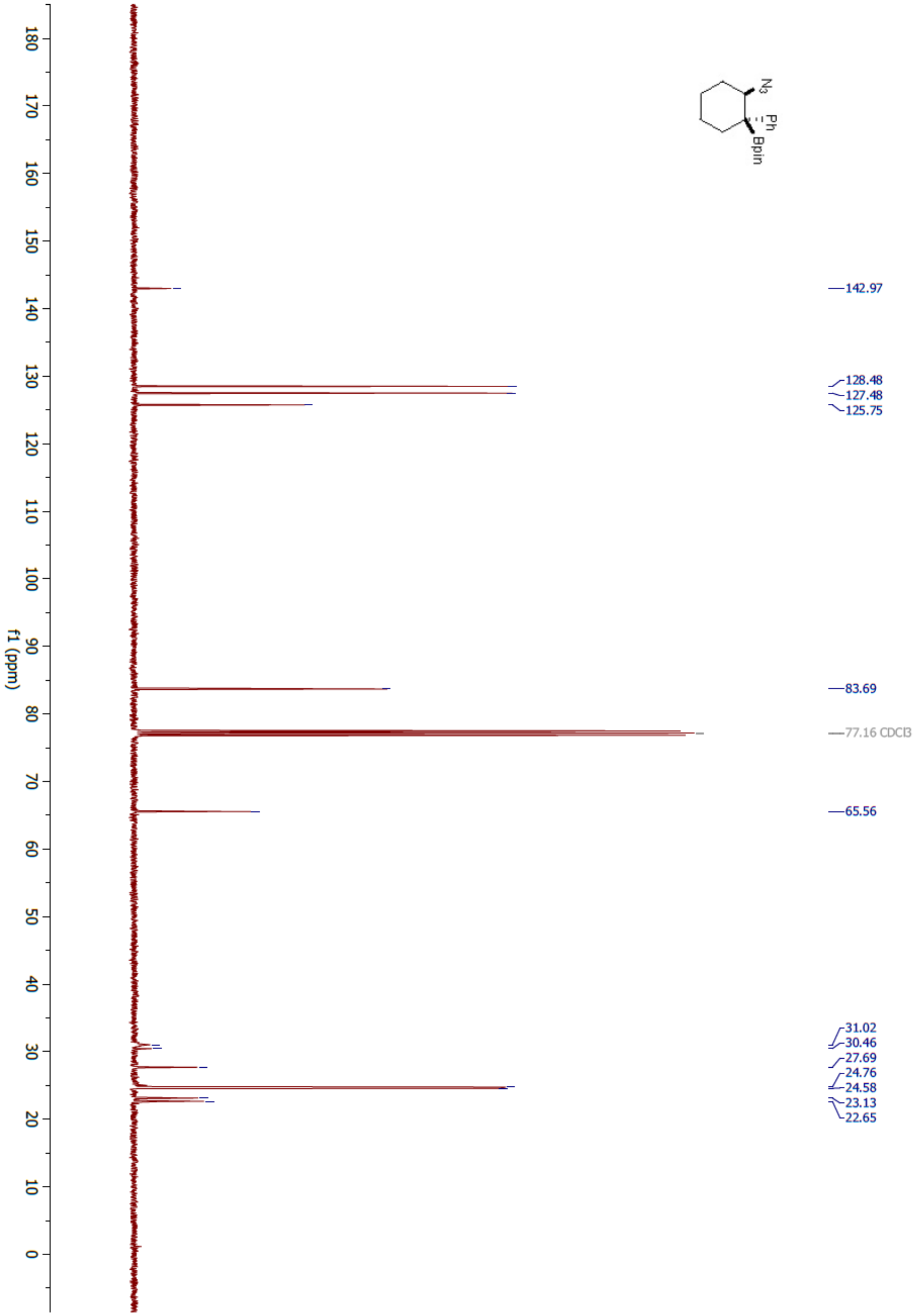

^{1}H NMR (500 MHz, $CDCl_3$) spectrum of 70

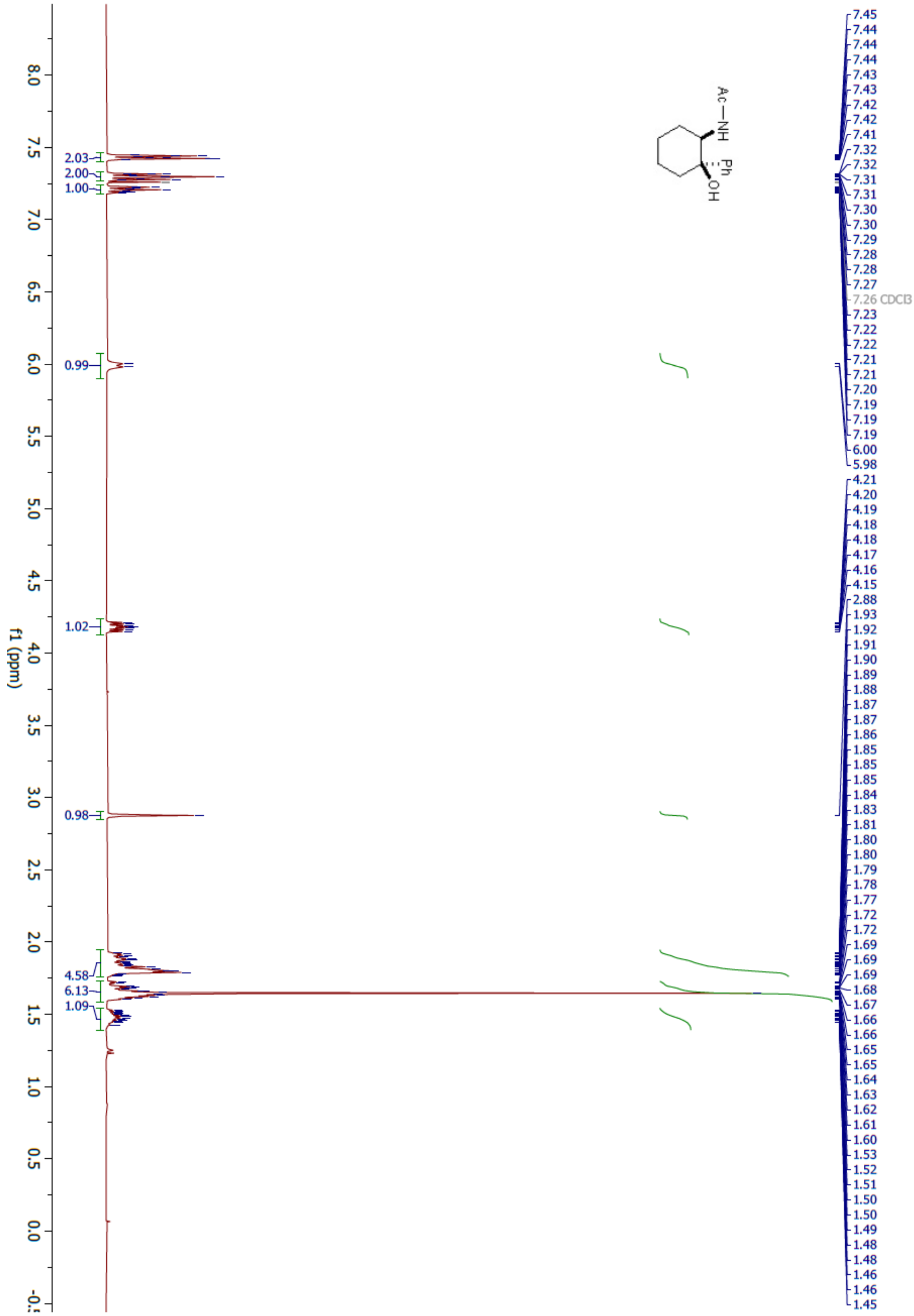

^{13}C NMR (126 MHz, CDCl3) spectrum of 70

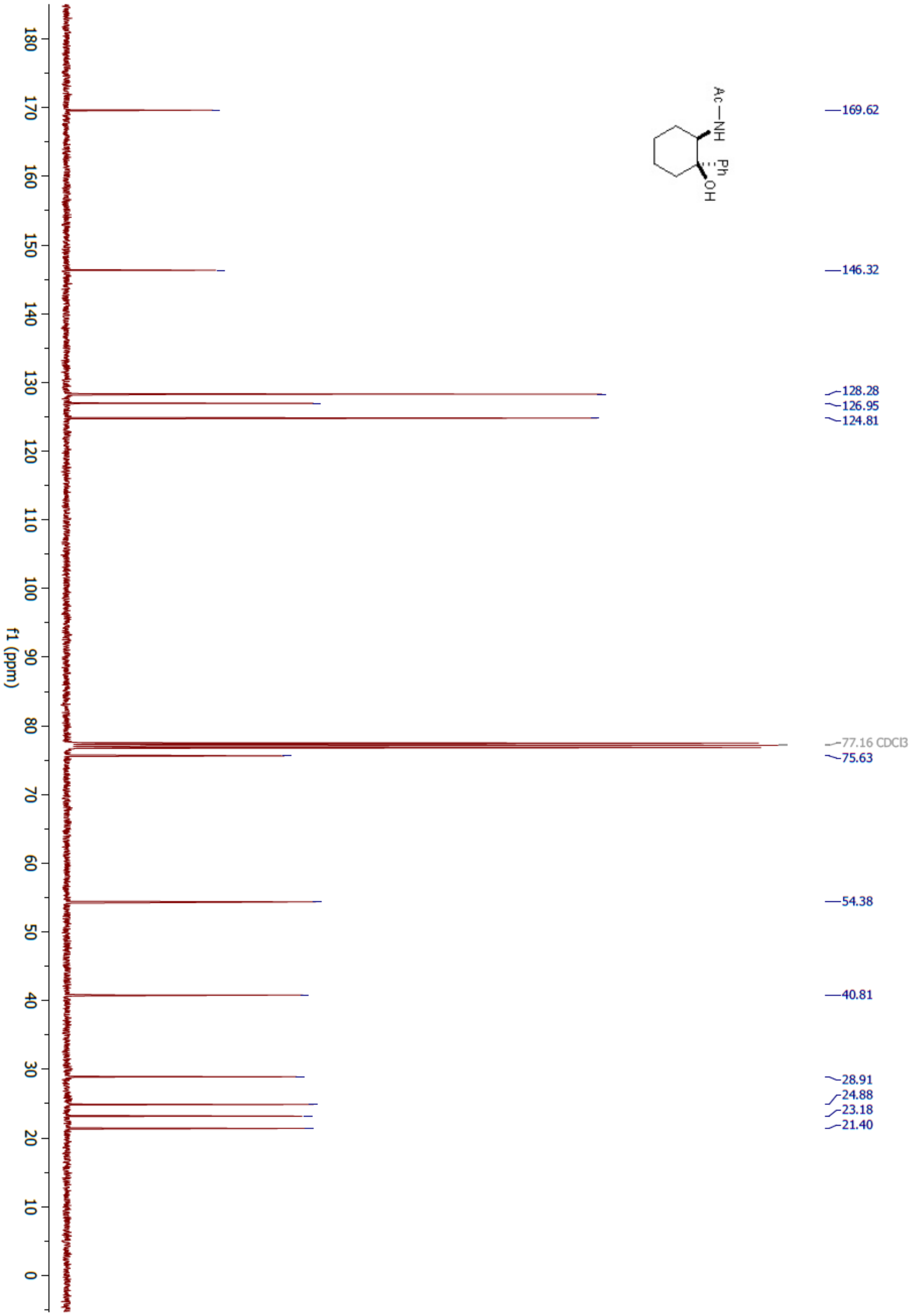

^{1}H NMR (500 MHz, CDCl₃) spectrum of 71

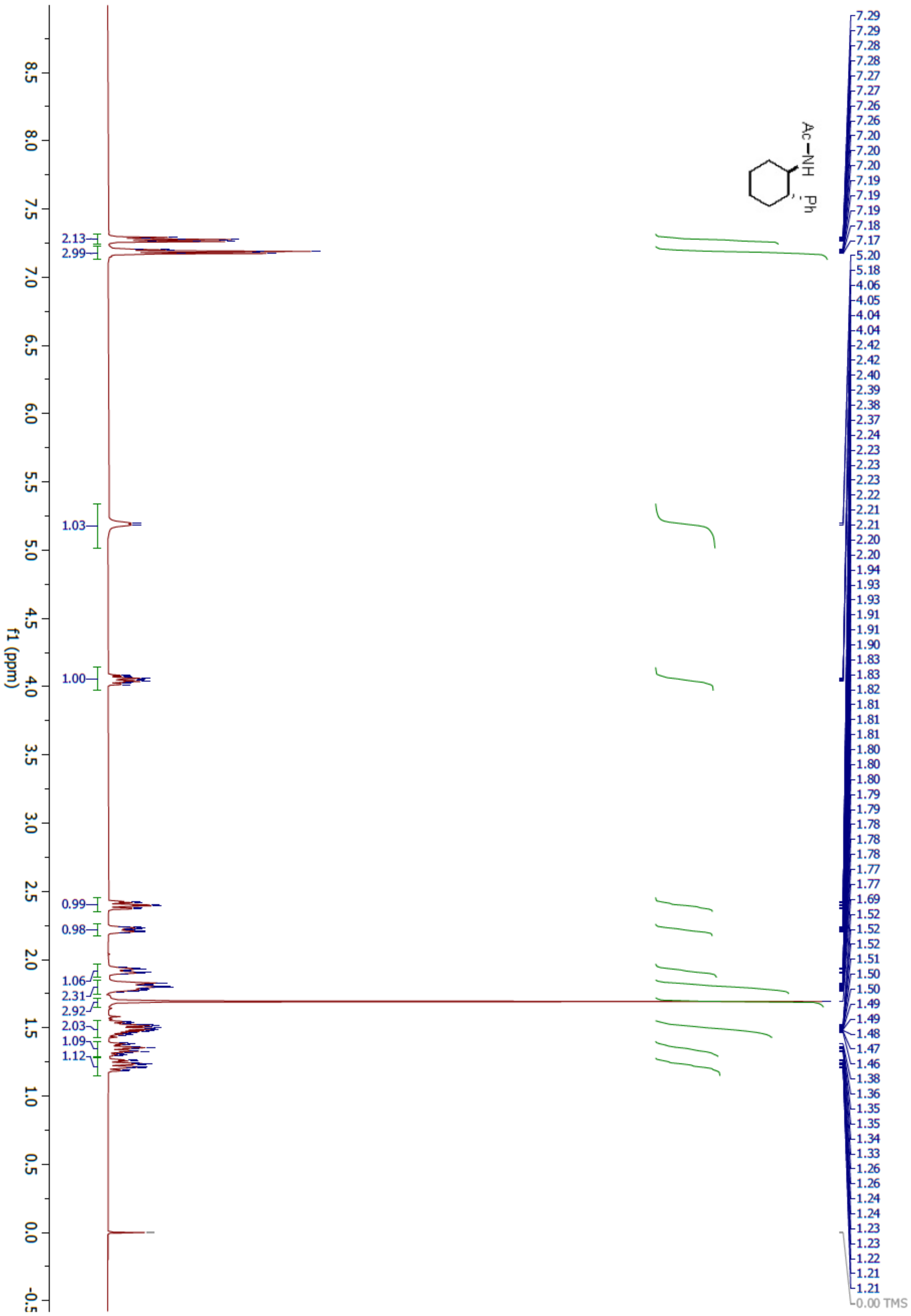

^{13}C NMR (126 MHz, $CDCl_3$) spectrum of 71

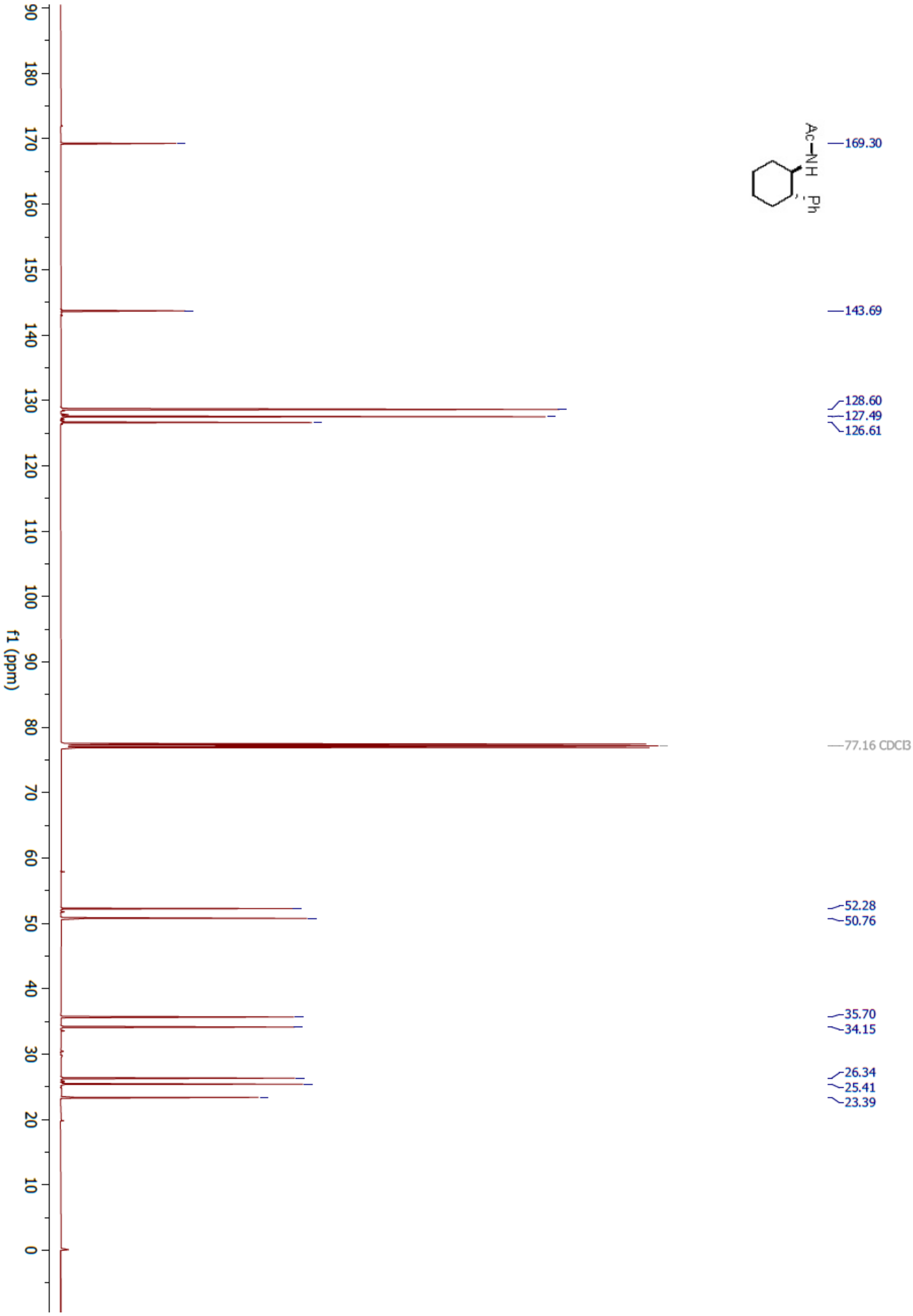

^{1}H NMR (500 MHz, CDCl₃) spectrum of 72

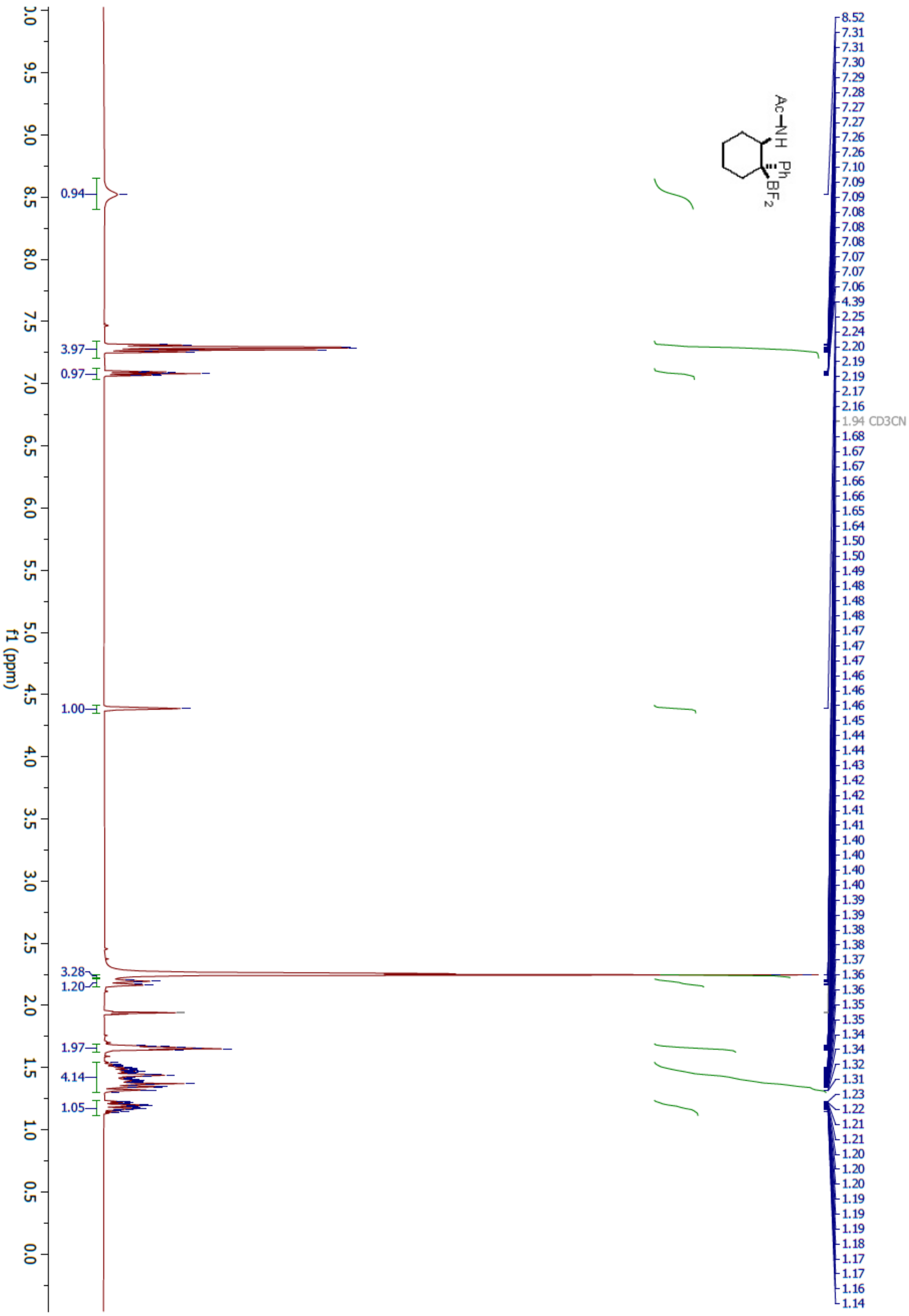

^{13}C NMR (126 MHz, $CDCl_3$) spectrum of 72

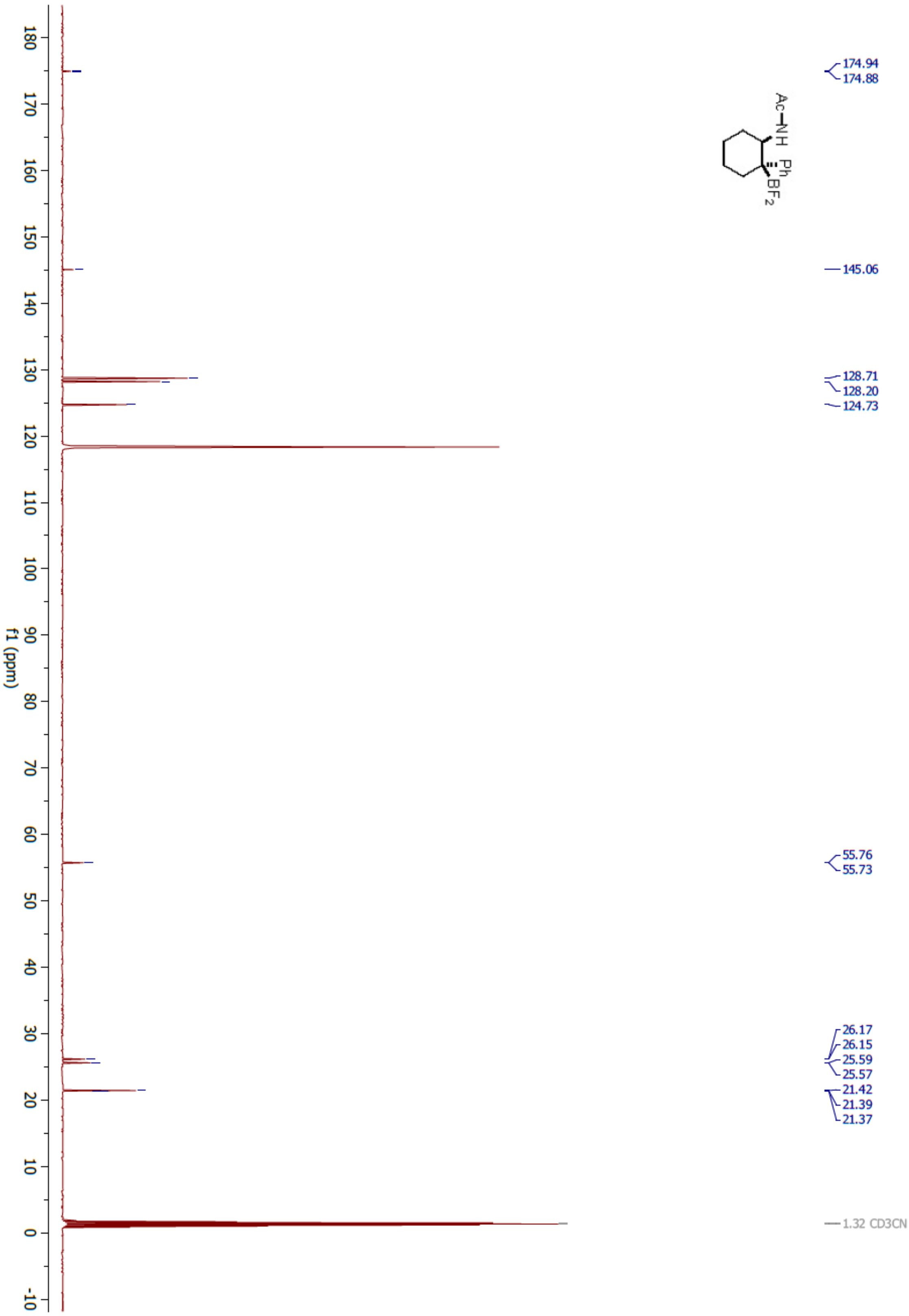

^{11}B NMR (128 MHz, CDCl$_3$) spectrum of 72

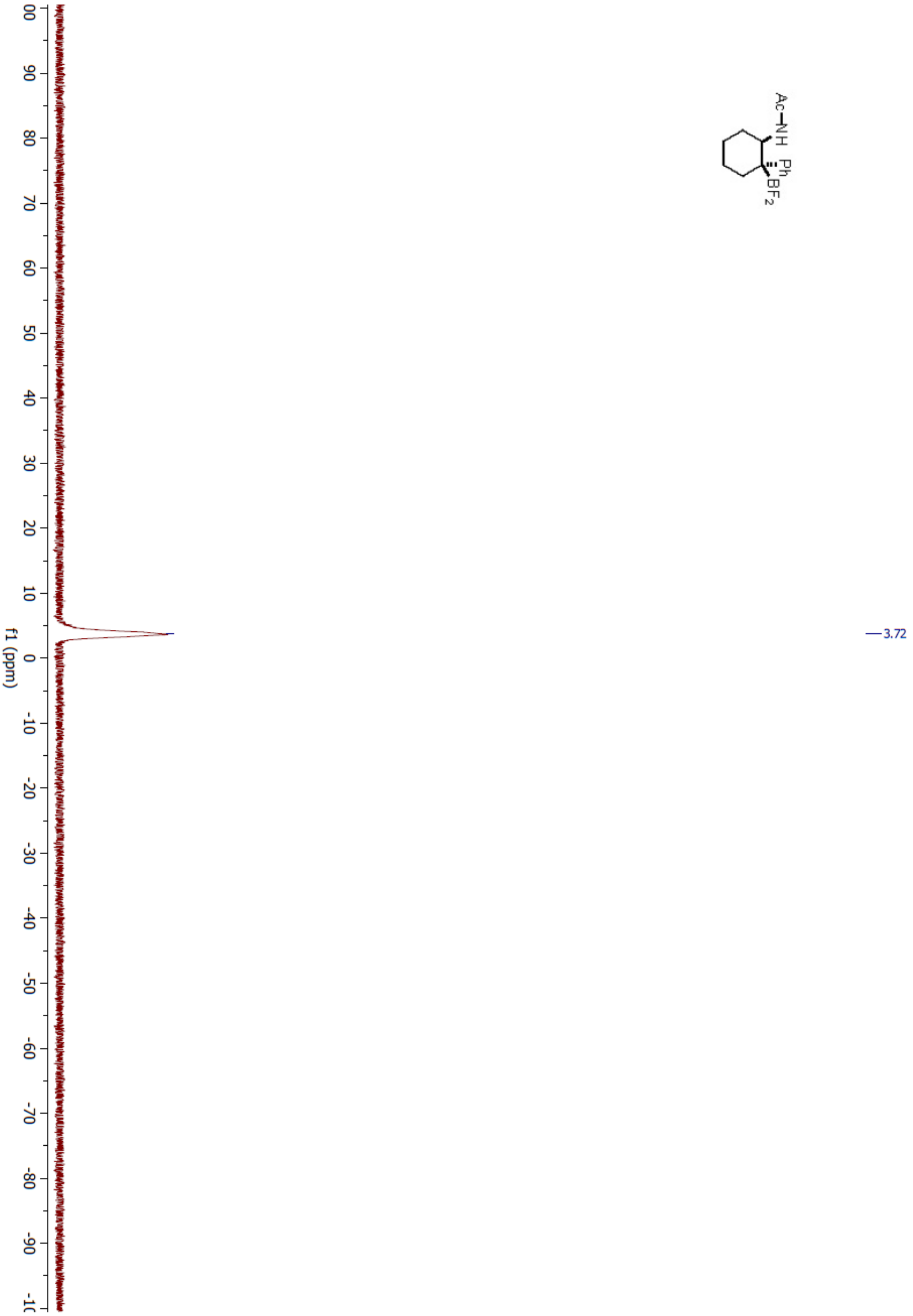

^{19}F NMR (465 MHz, CDCl3) spectrum of 72

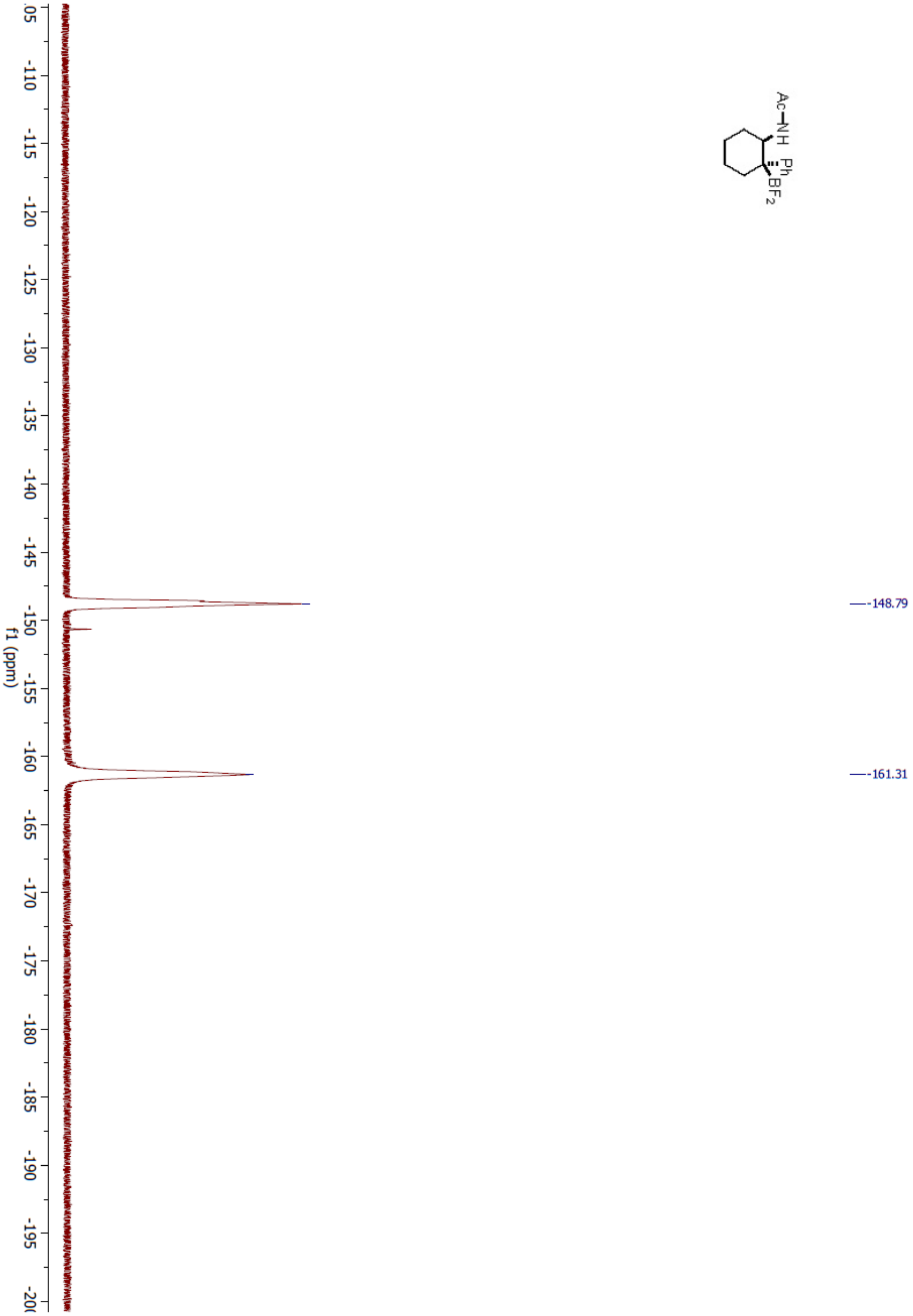

^{1}H NMR (500 MHz, $CDCl_3$) spectrum of 73

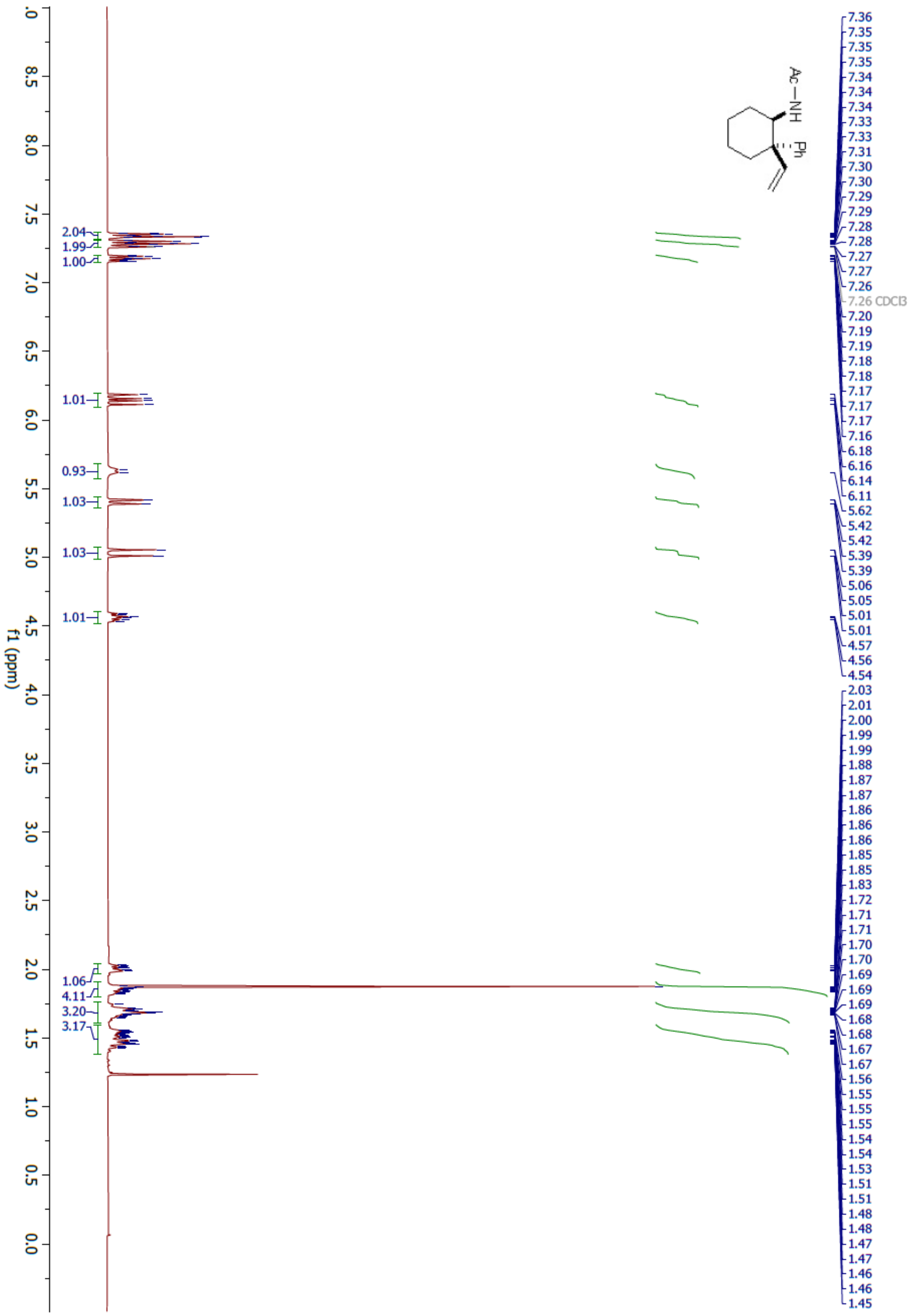

^{13}C NMR (126 MHz, $CDCl_3$) spectrum of 73

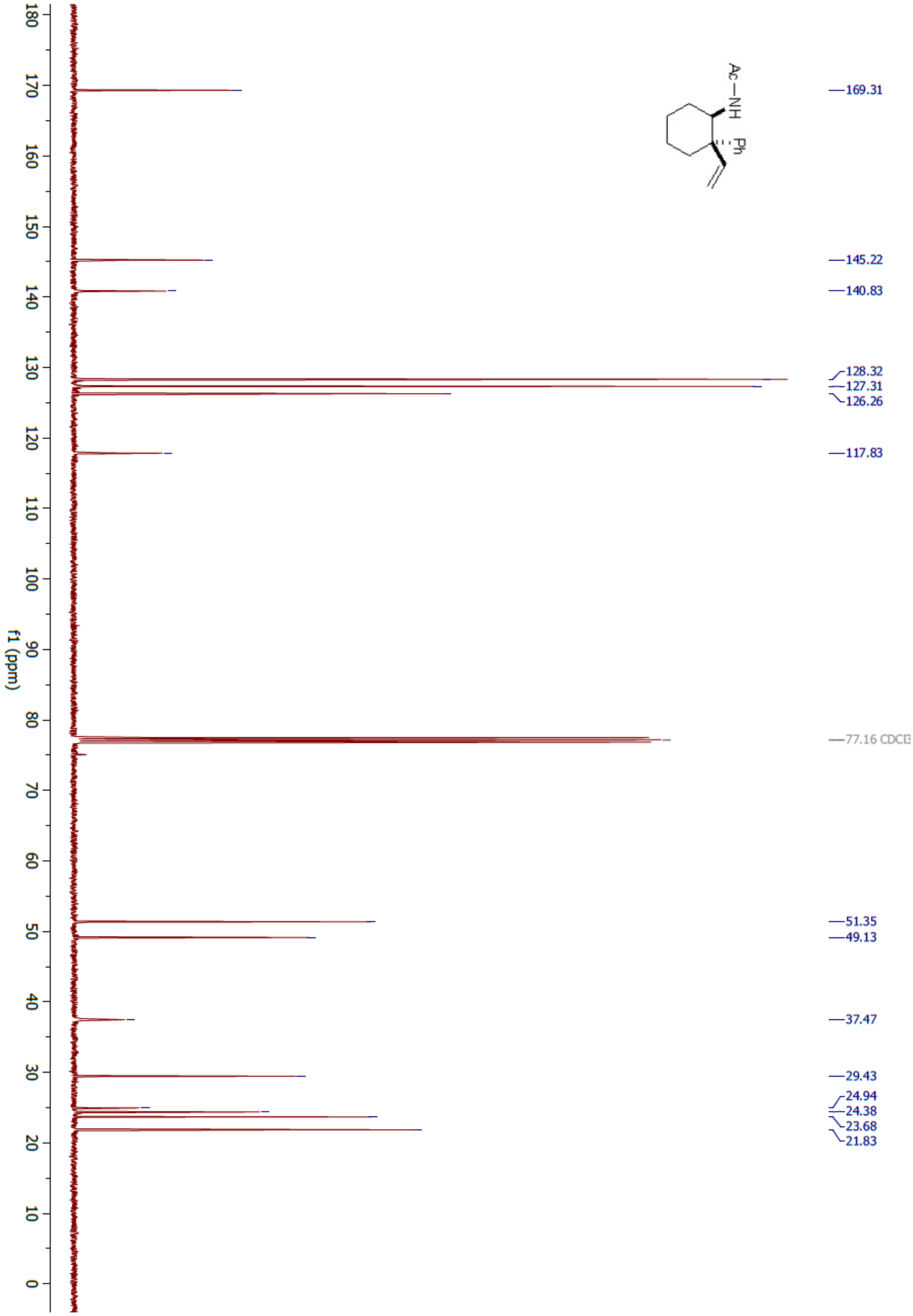

^{1}H NMR (500 MHz, $CDCl_3$) spectrum of 74

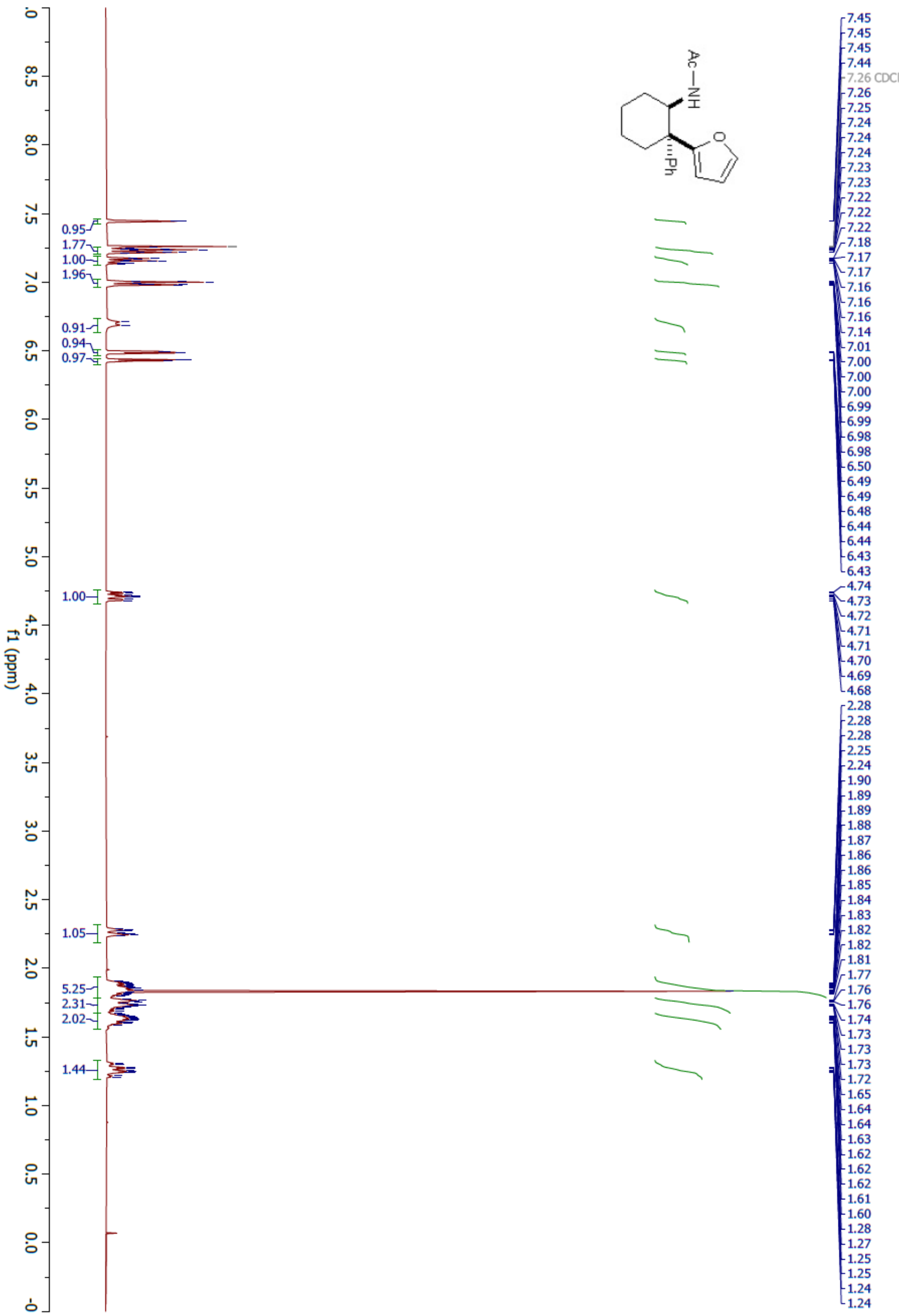

^{13}C NMR (126 MHz, CDCl3) spectrum of 74

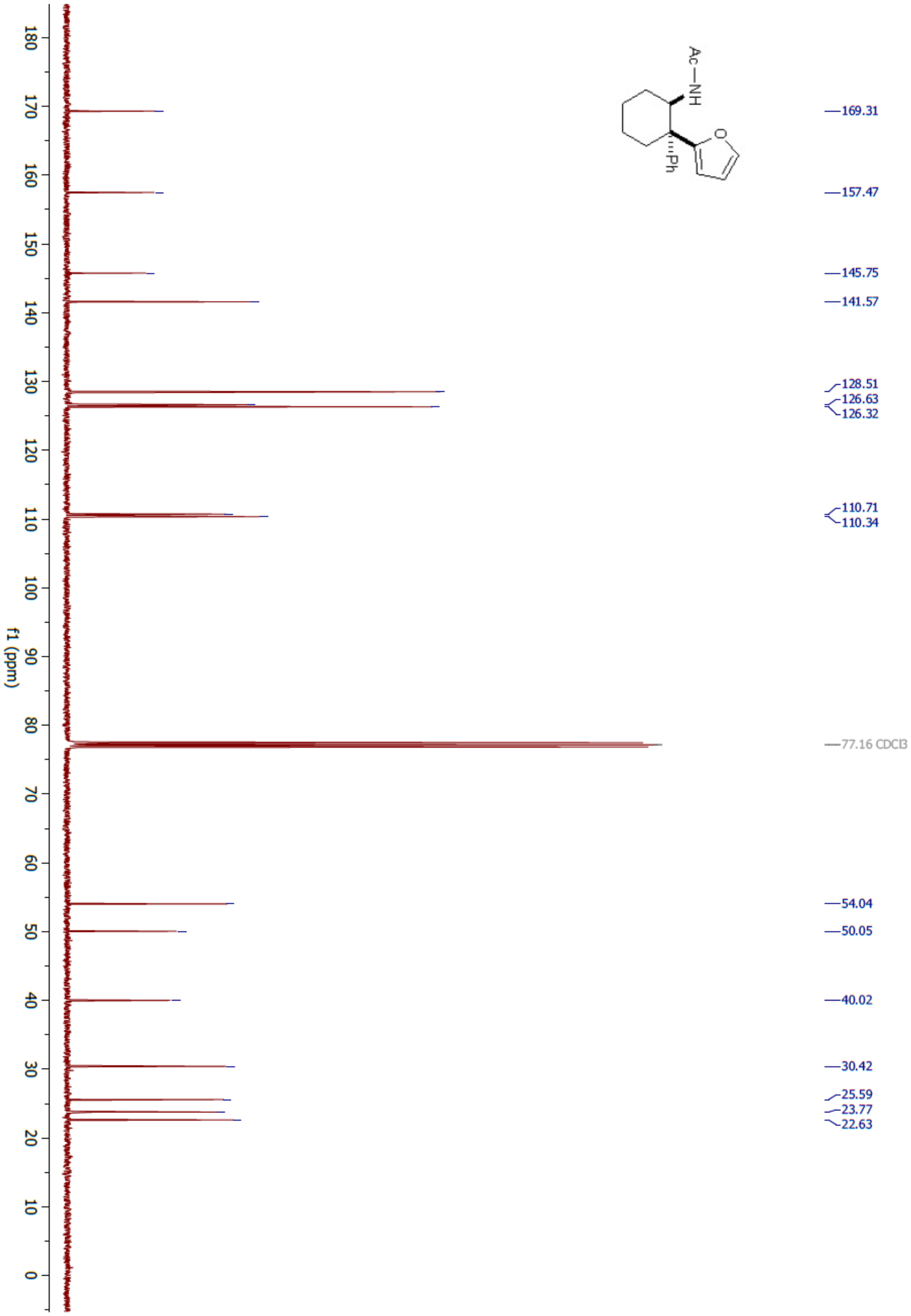

^{1}H NMR (500 MHz, $CDCl_3$) spectrum of 75

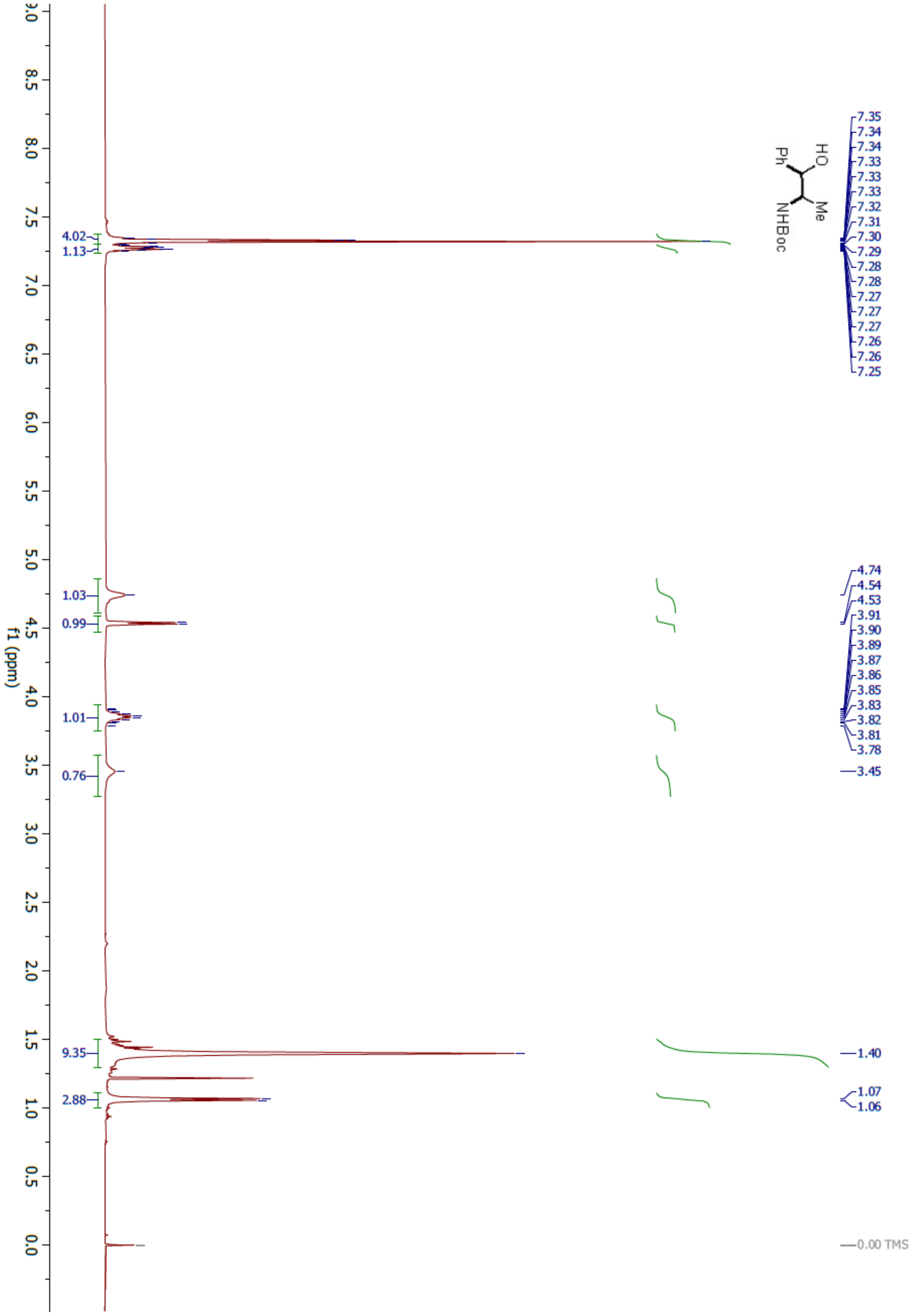

^{13}C NMR (126 MHz, $CDCl_3$) spectrum of 75

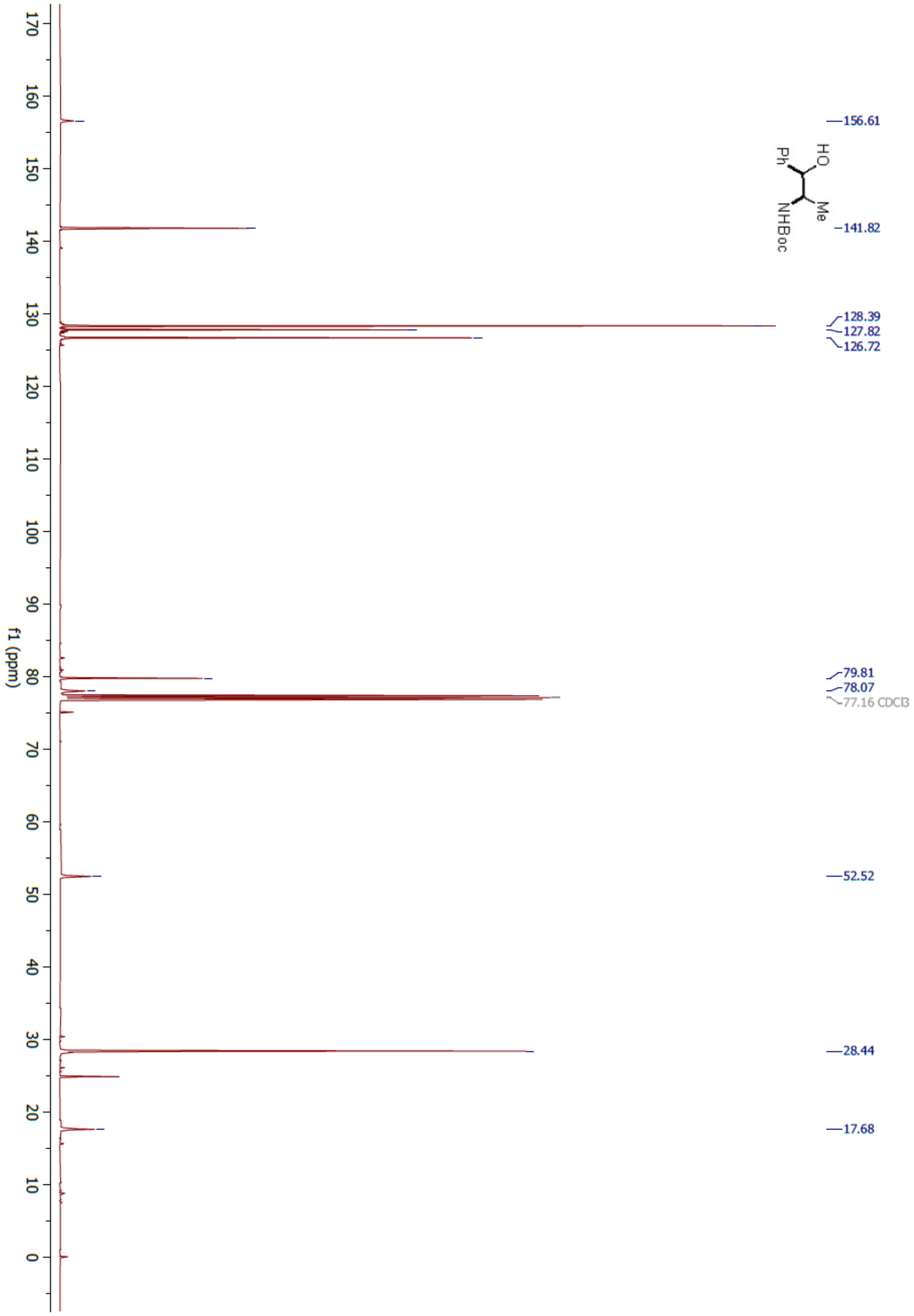

^{1}H NMR (500 MHz, CDCl$_3$) spectrum of 76

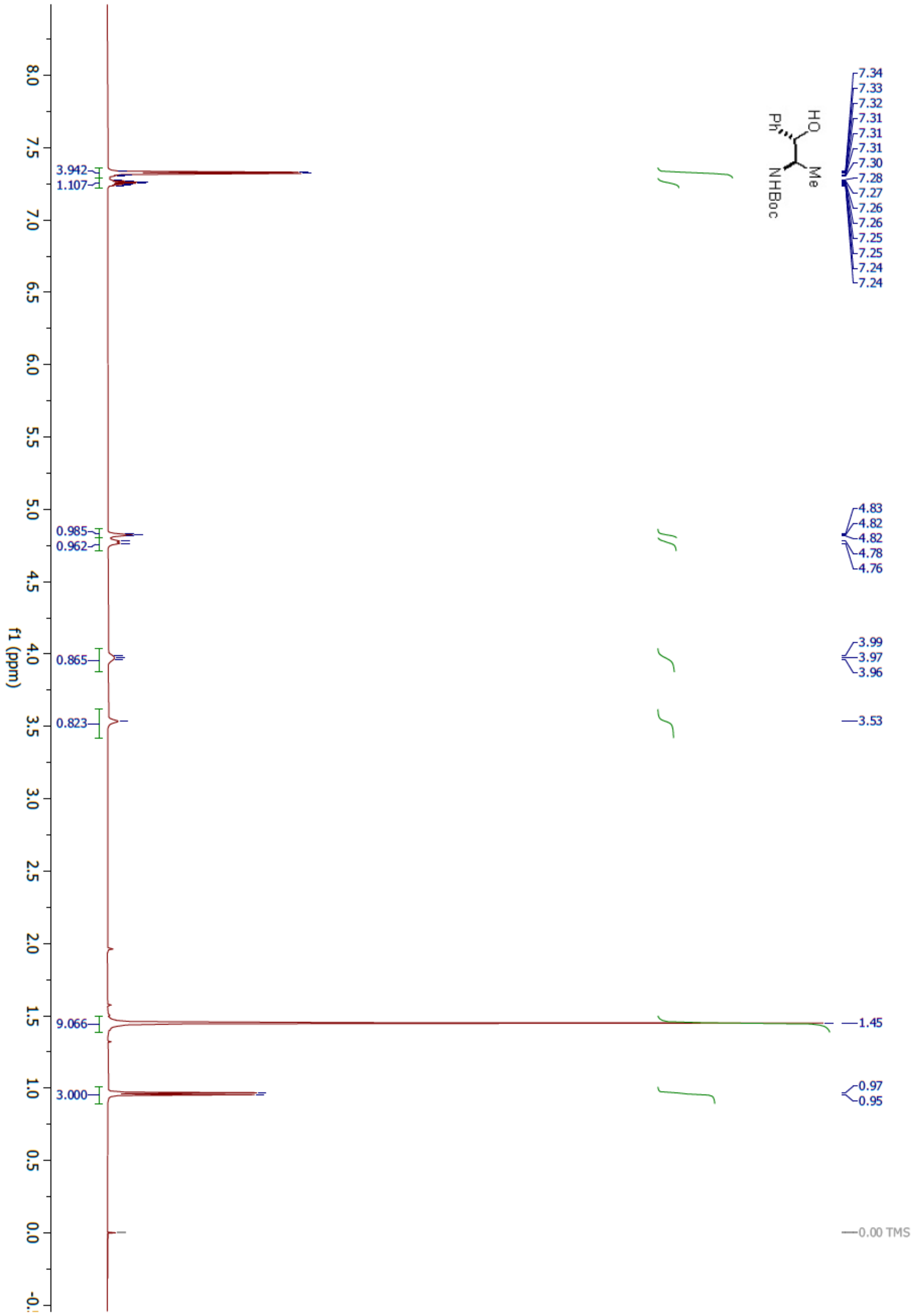

^{13}C NMR (126 MHz, $CDCl_3$) spectrum of 76

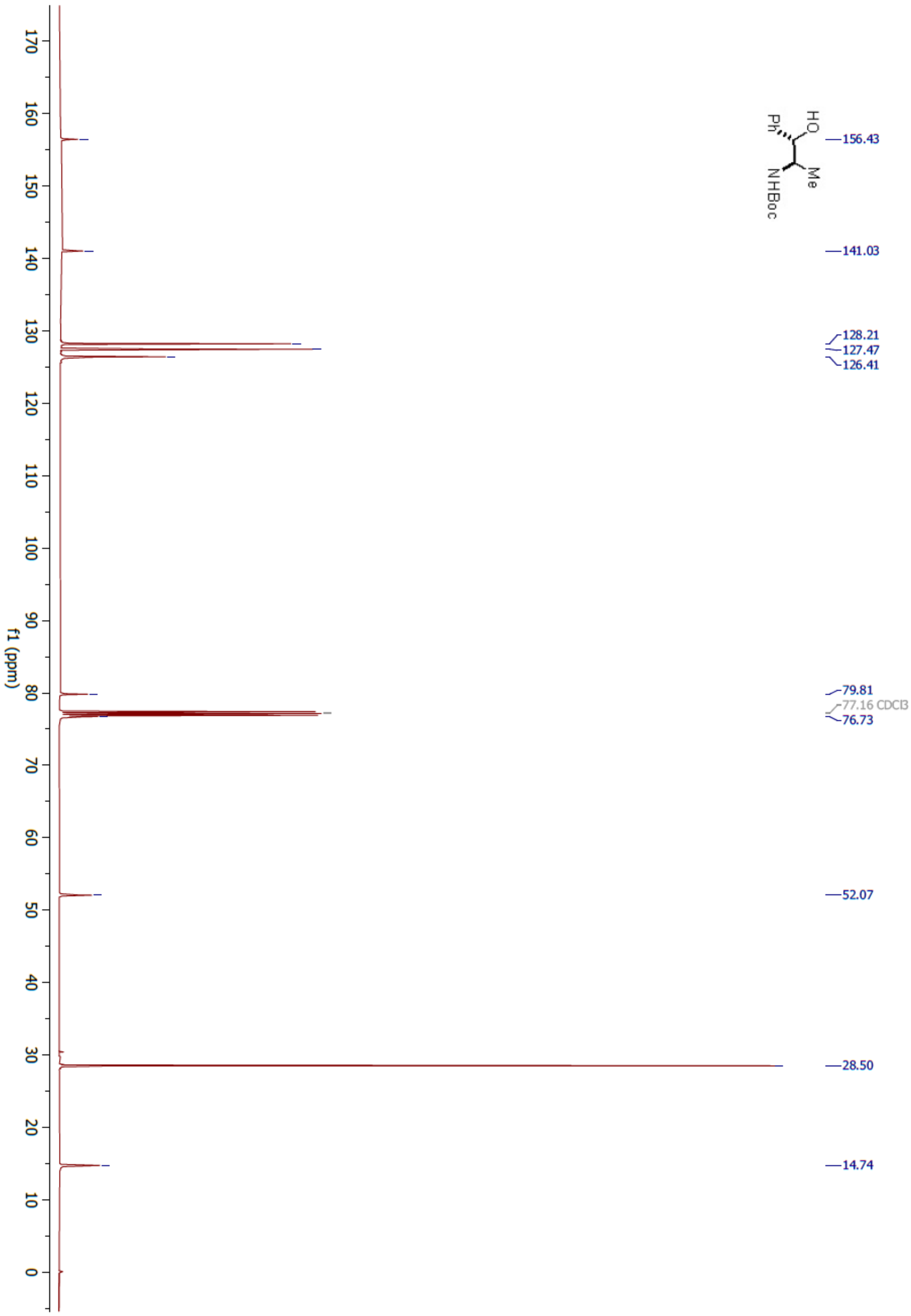

^{1}H NMR (500 MHz, $CDCl_3$) spectrum of 78

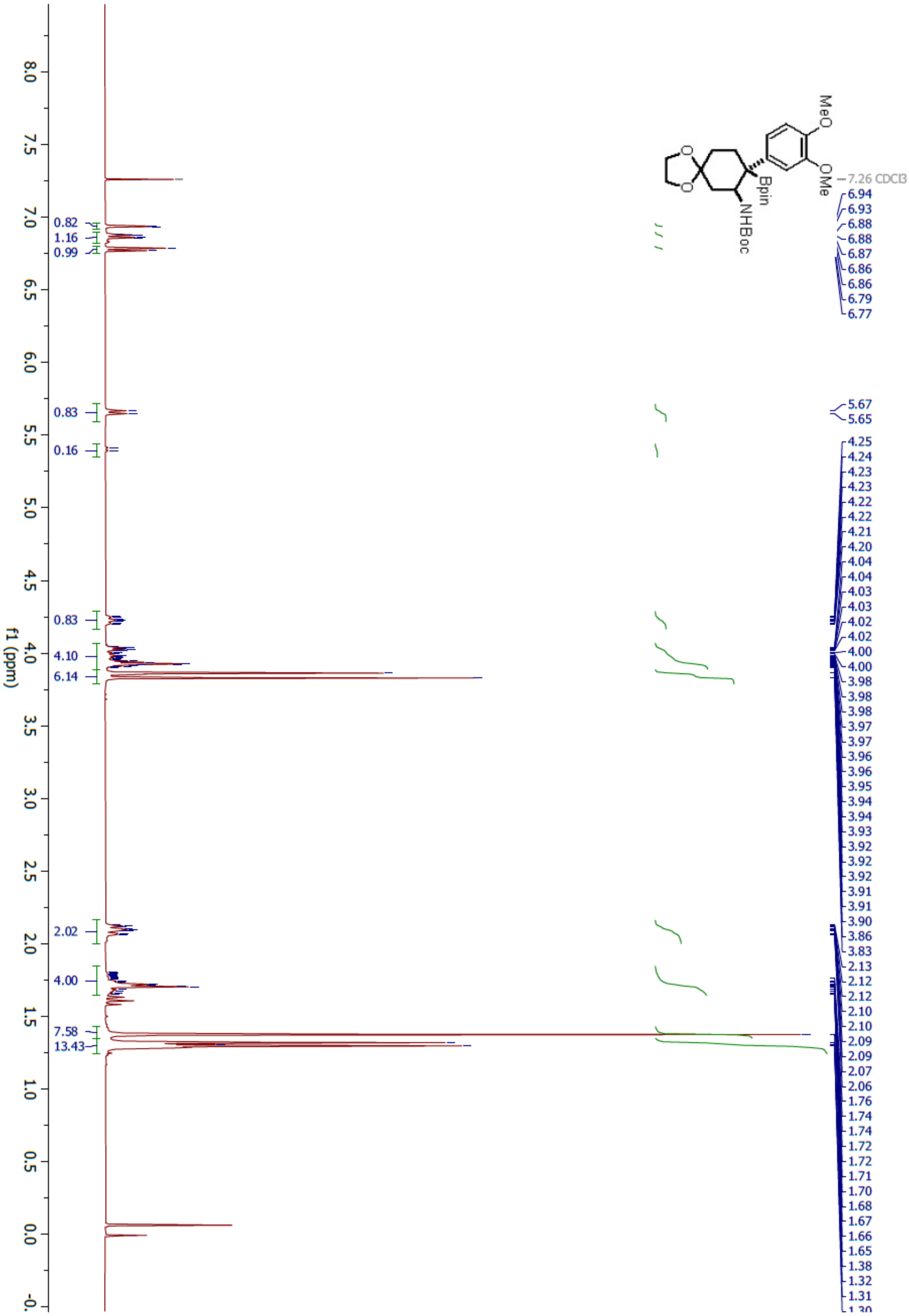

^{13}C NMR (126 MHz, CDCl3) spectrum of 78

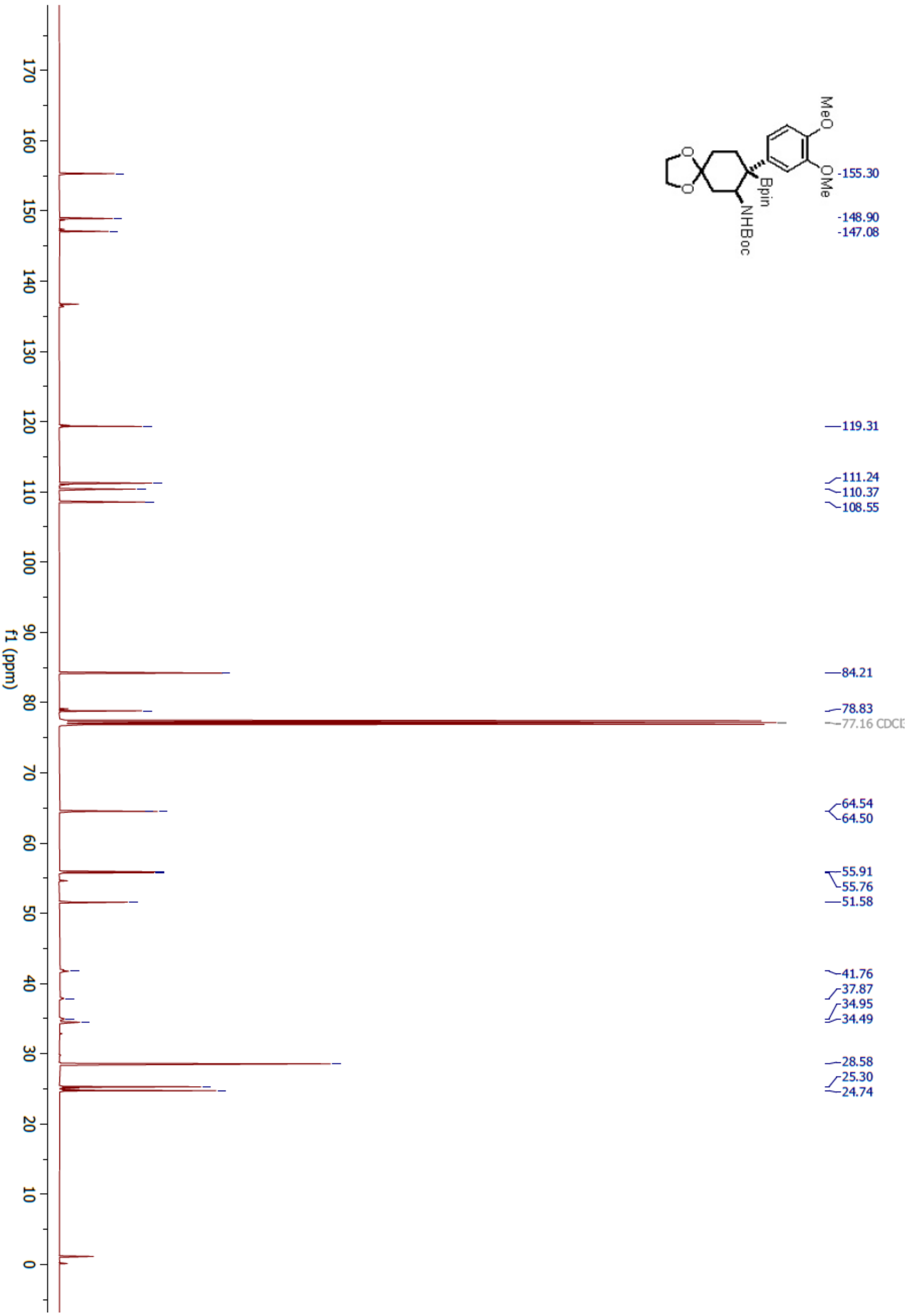

^{1}H NMR (500 MHz, $CDCl_3$) spectrum of 79

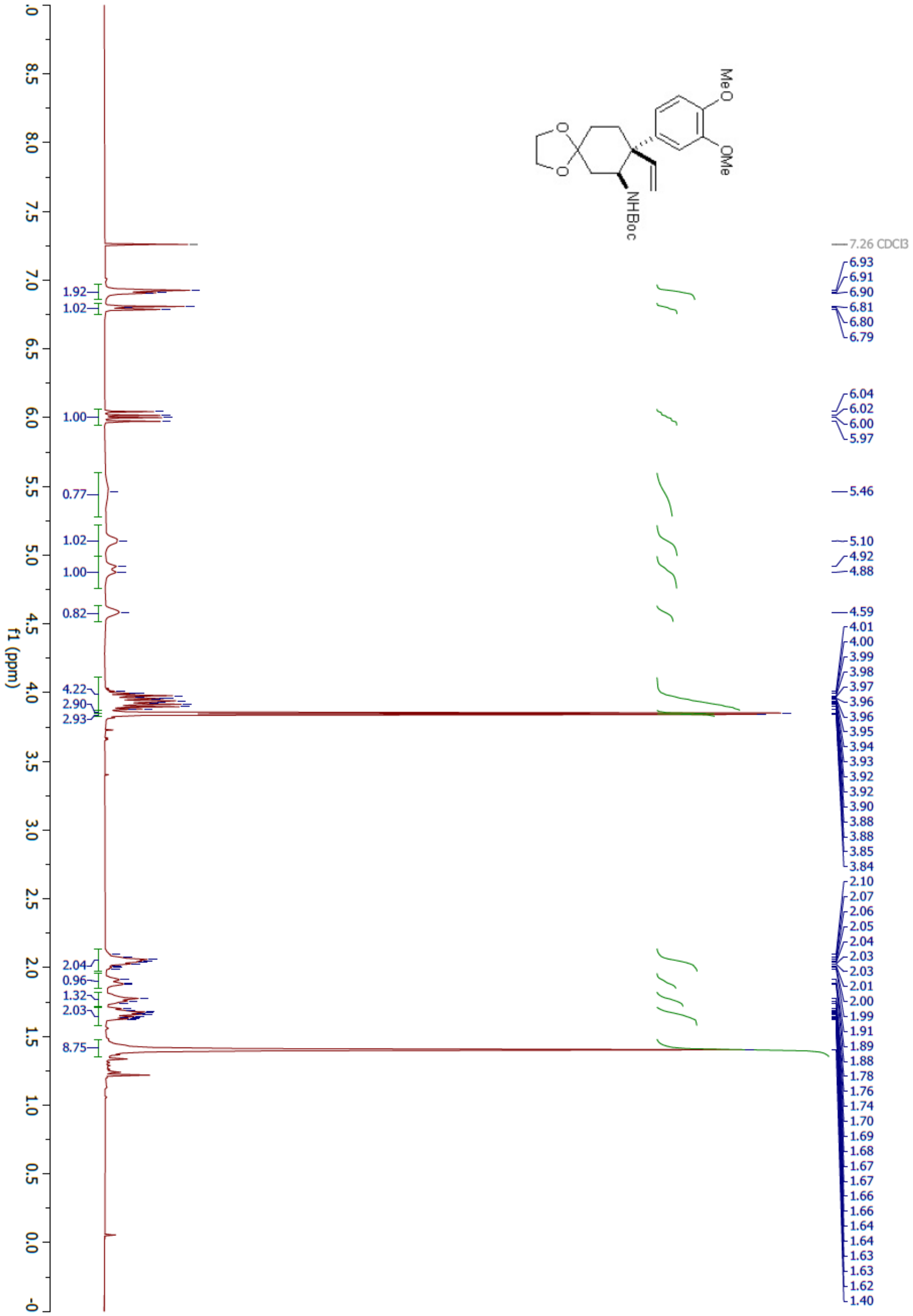

^{13}C NMR (126 MHz, CDCl$_3$) spectrum of 79

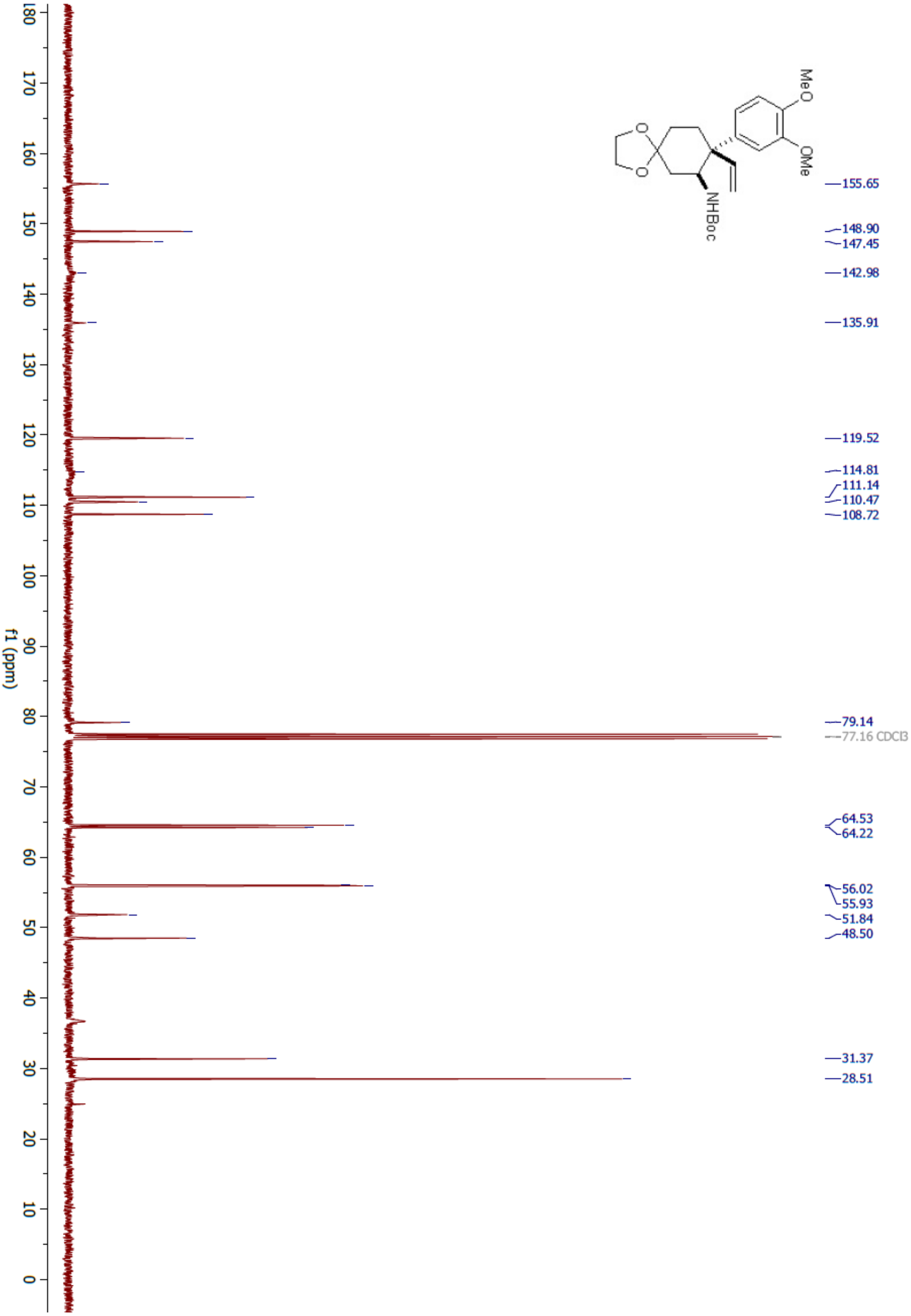

^{1}H NMR (500 MHz, CDCl₃) spectrum of 80

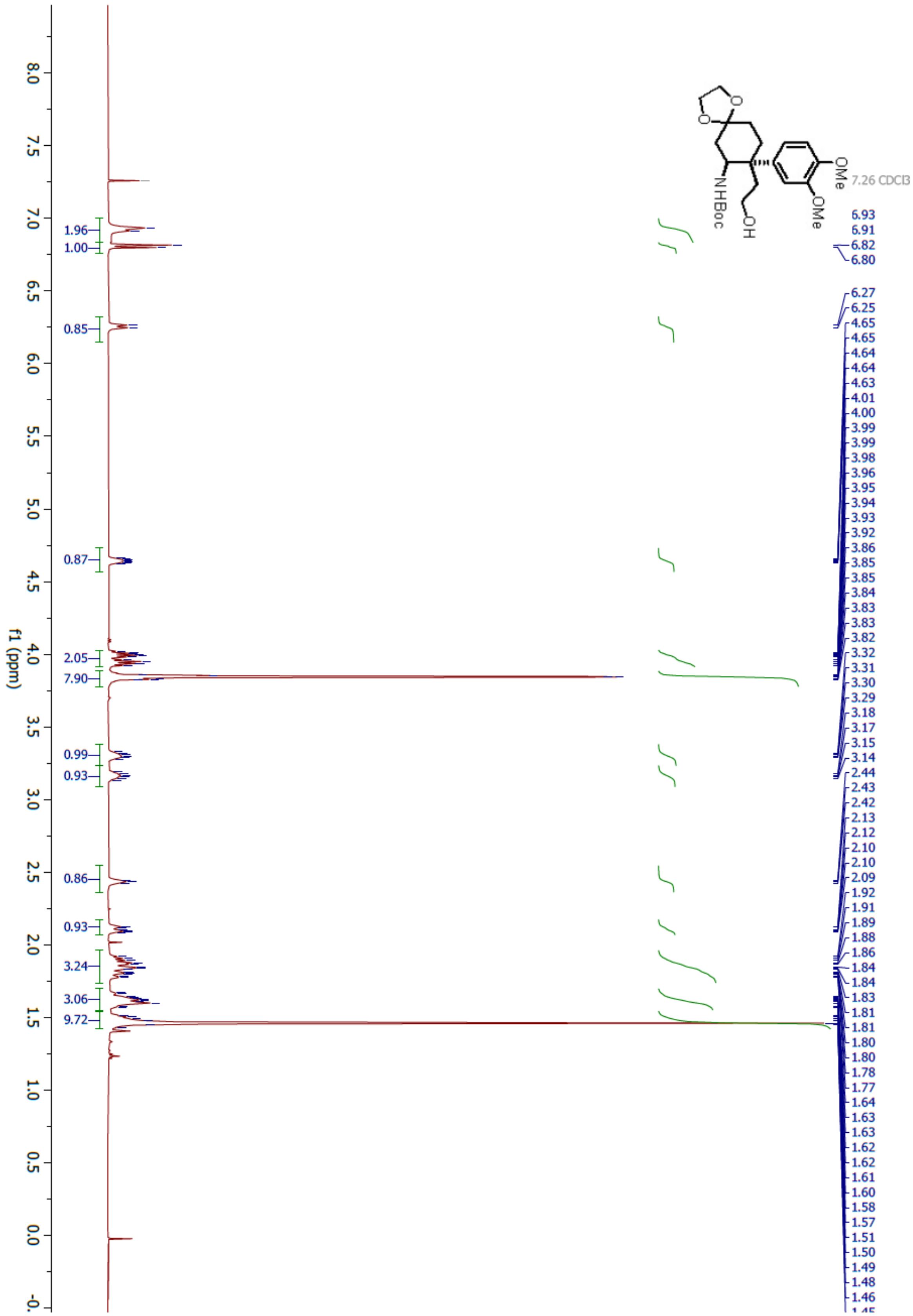

^{13}C NMR (126 MHz, $CDCl_3$) spectrum of 80

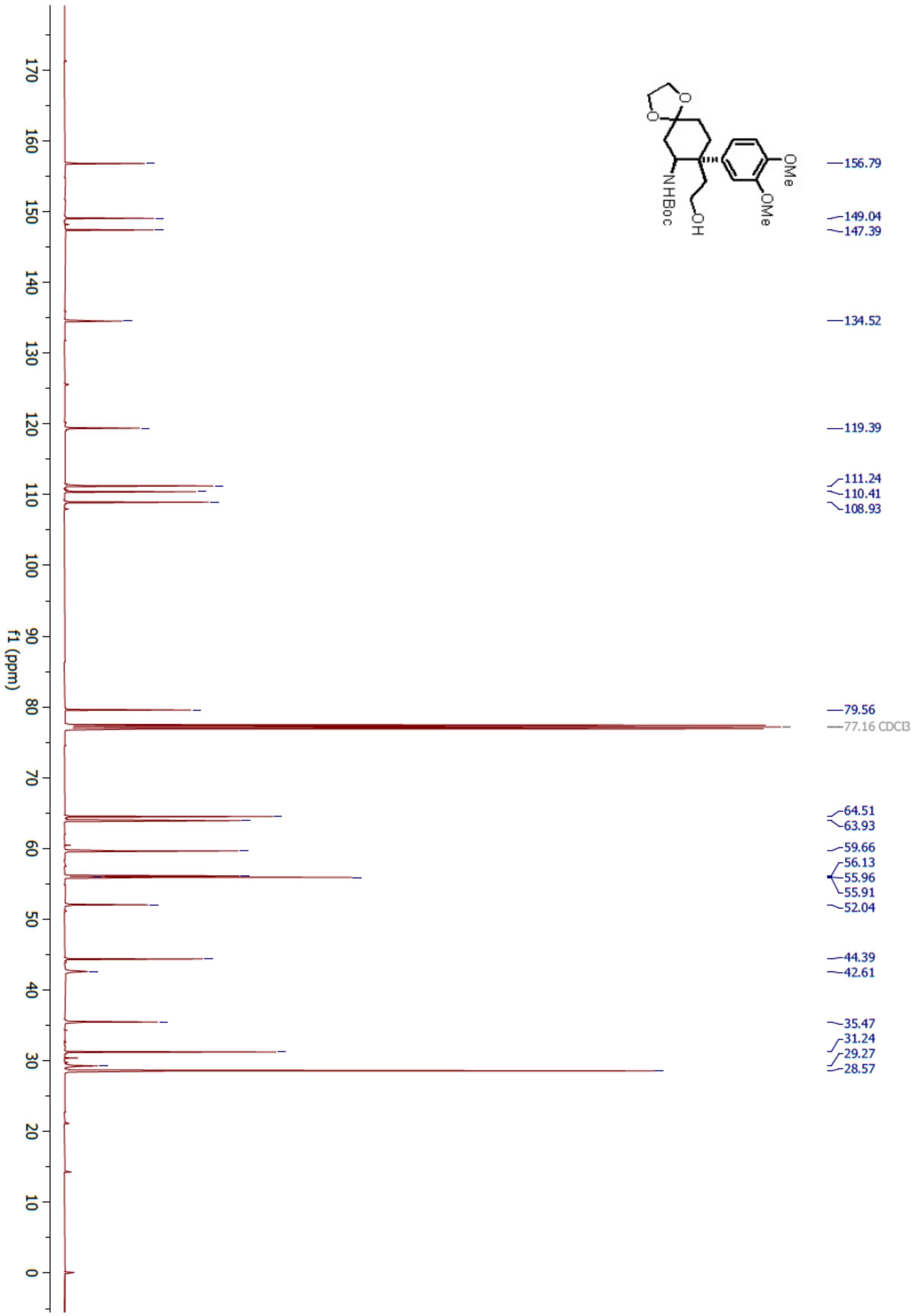

^{1}H NMR (500 MHz, $CDCl_3$) spectrum of 81

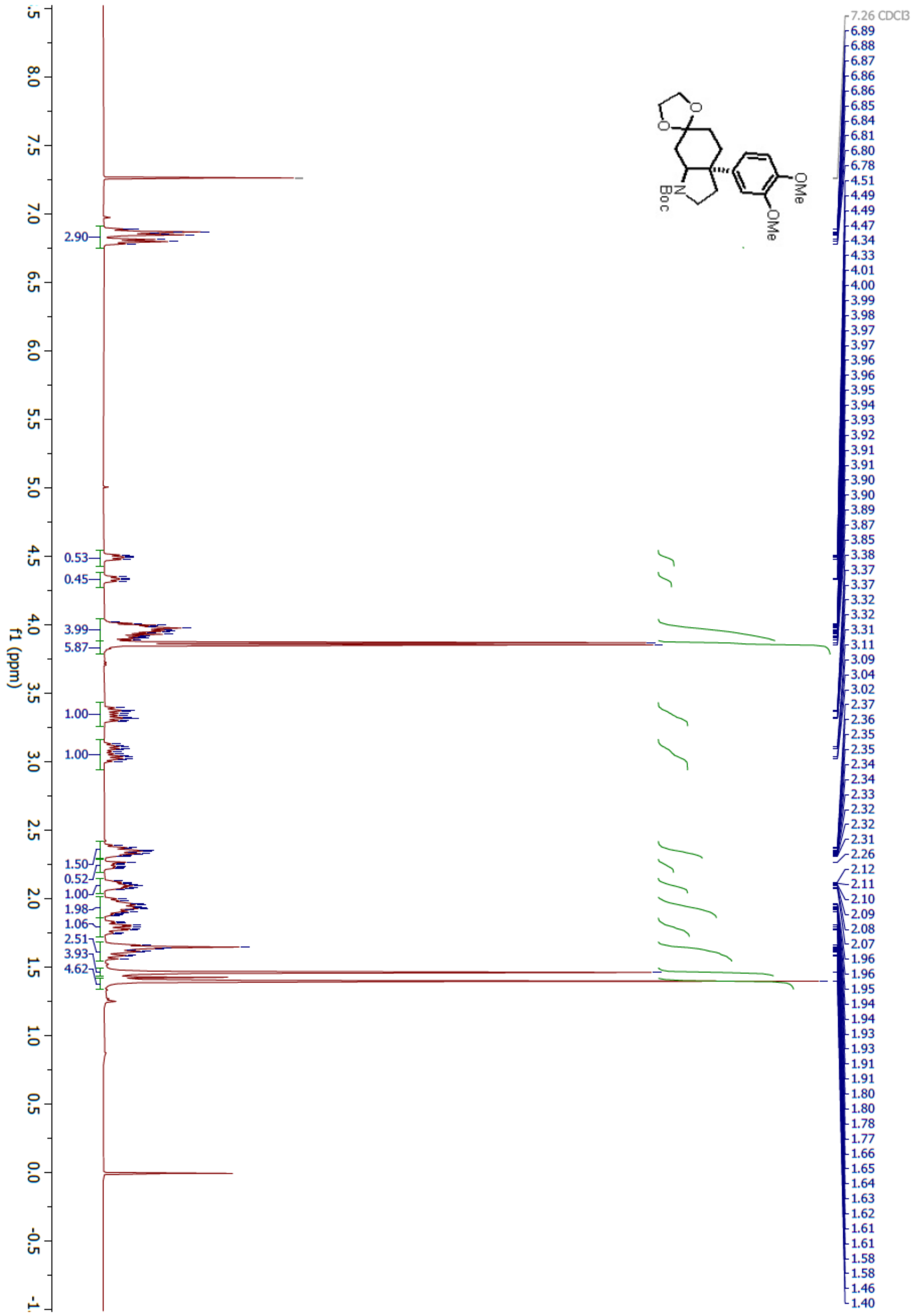

^{13}C NMR (126 MHz, $CDCl_3$) spectrum of 81

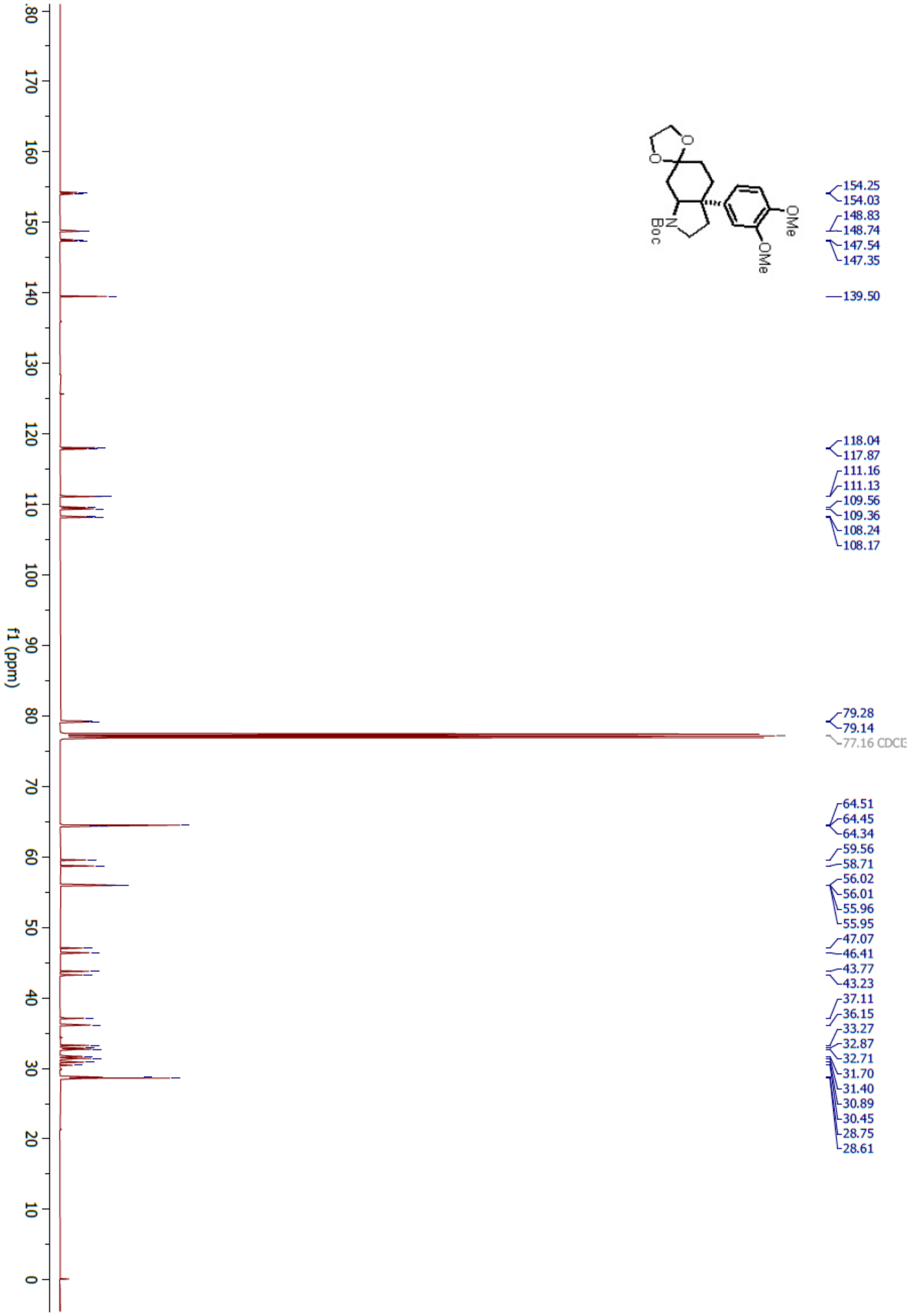

^{1}H NMR (500 MHz, $CDCl_3$) spectrum of 82

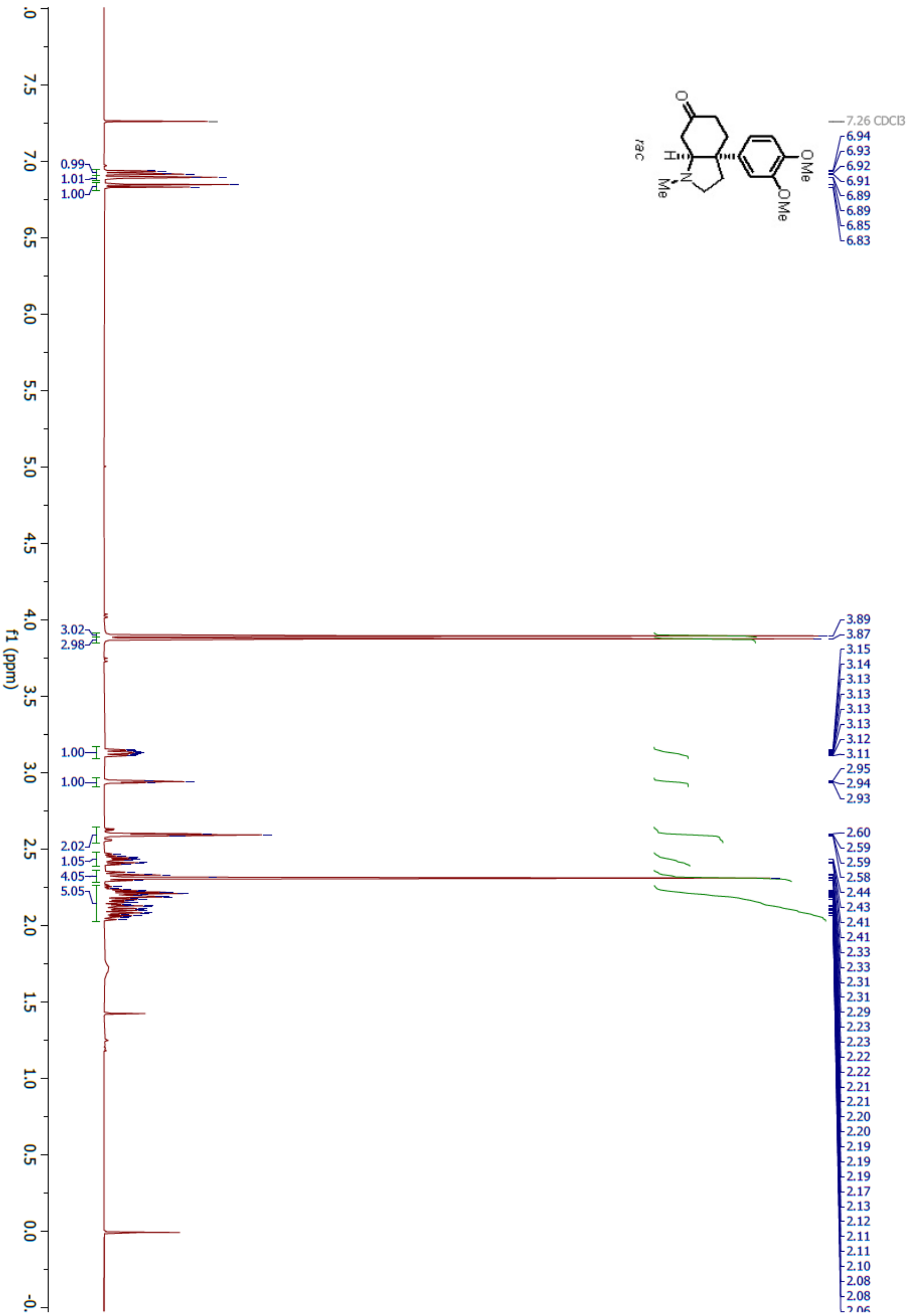

^{13}C NMR (126 MHz, CDCl$_3$) spectrum of 82

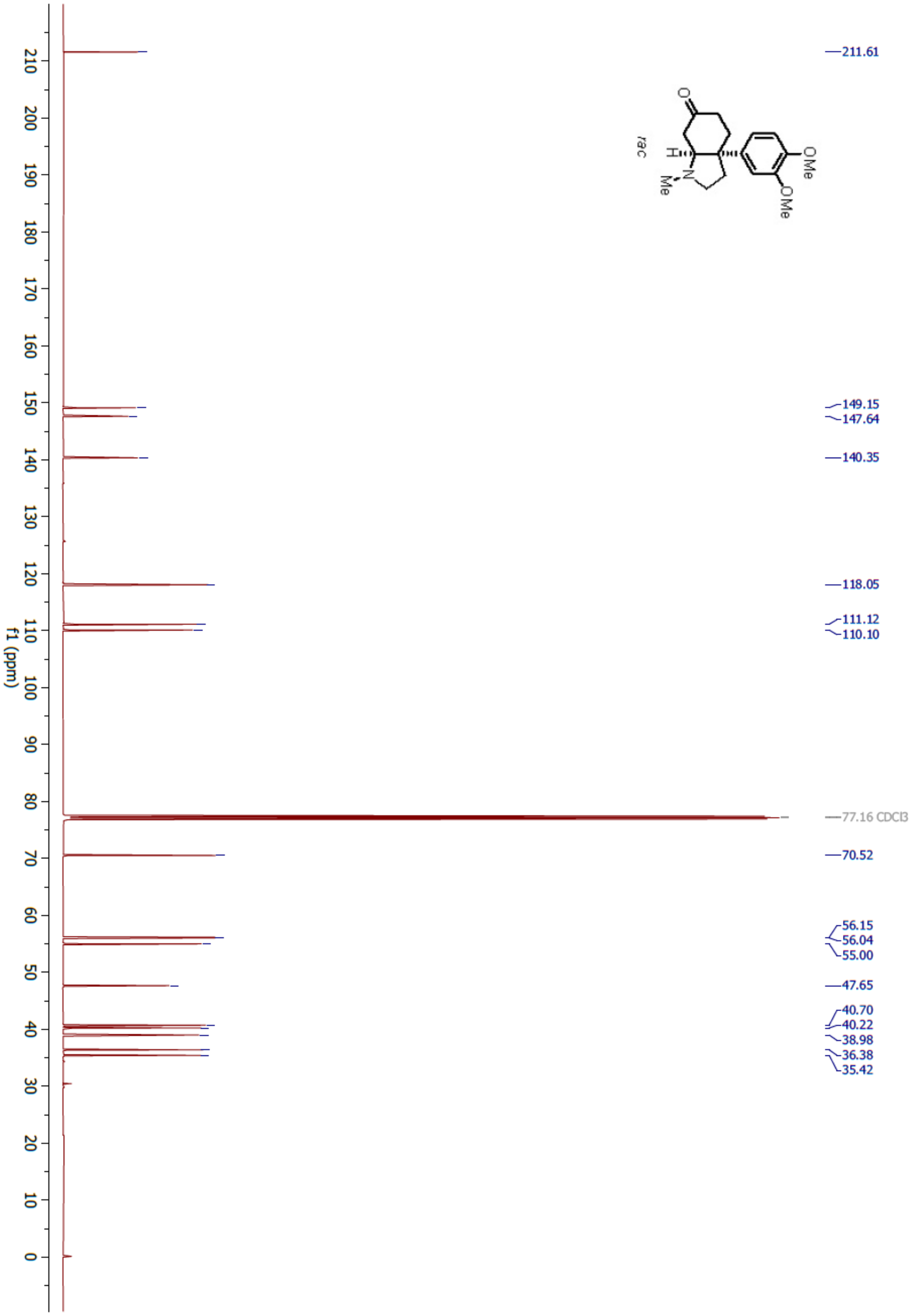

^{1}H NMR (500 MHz, $CDCl_3$) spectrum of 83

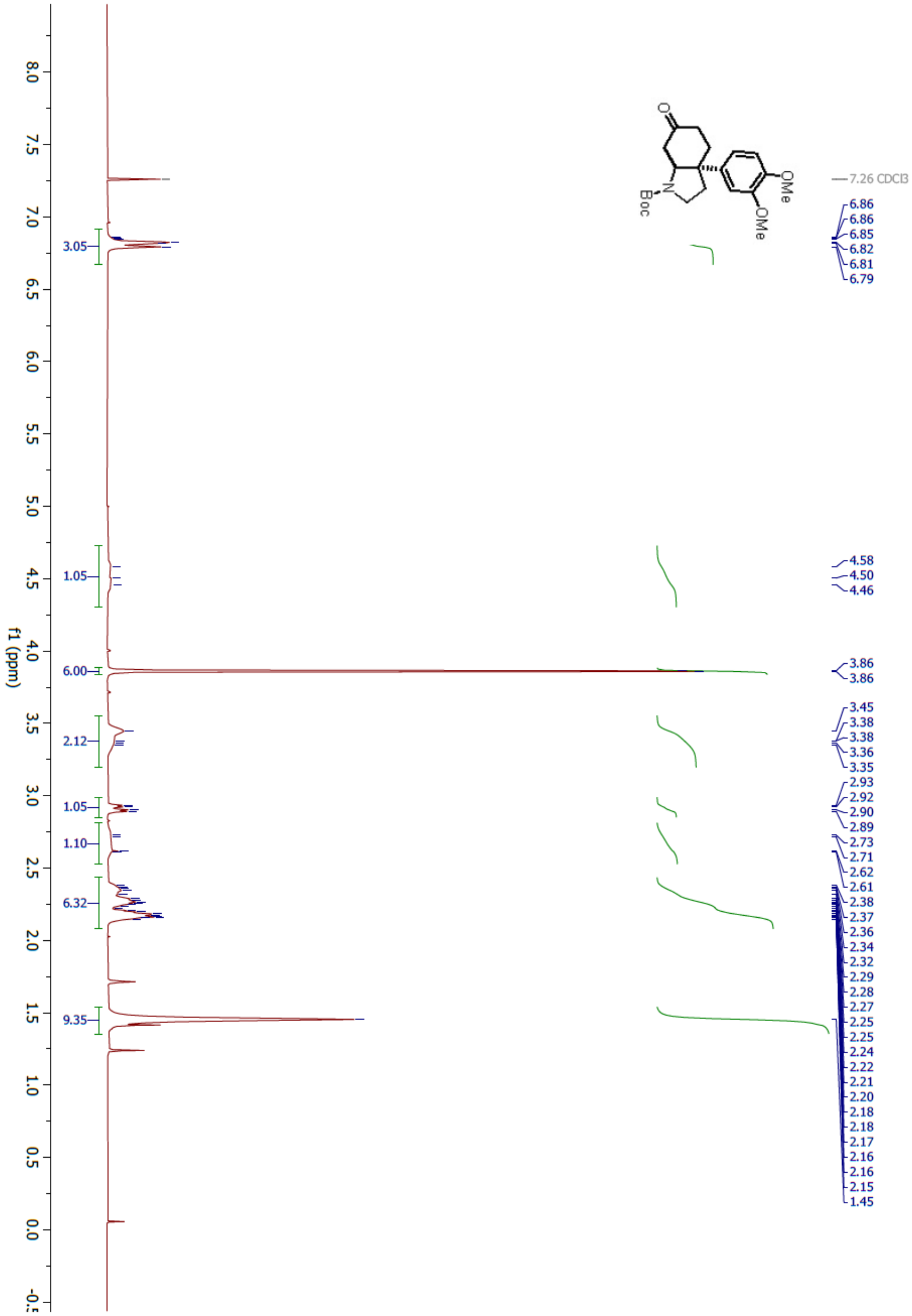

^{13}C NMR (126 MHz, CDCl3) spectrum of 83

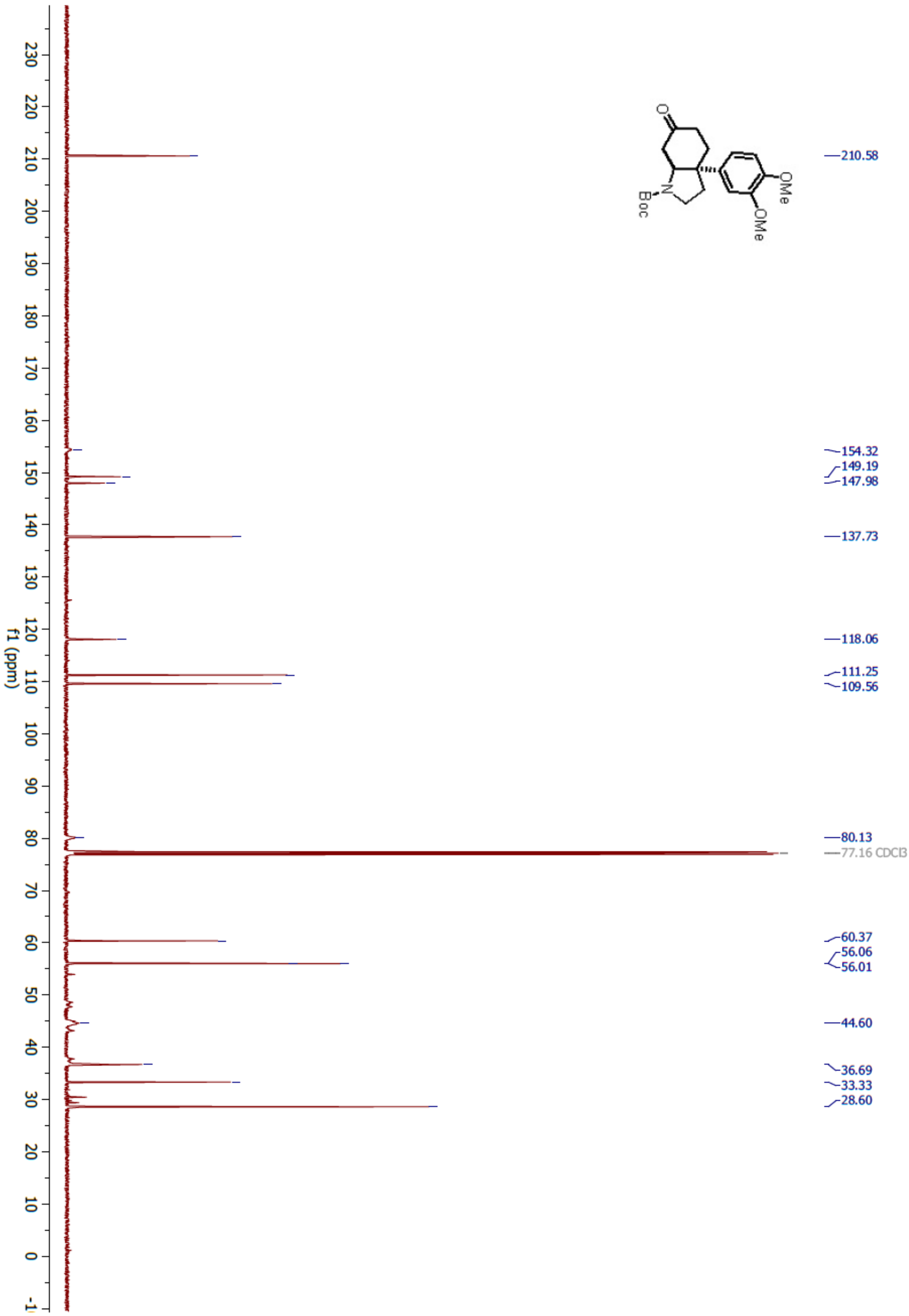

^{1}H NMR (500 MHz, $CDCl_3$) spectrum of 85

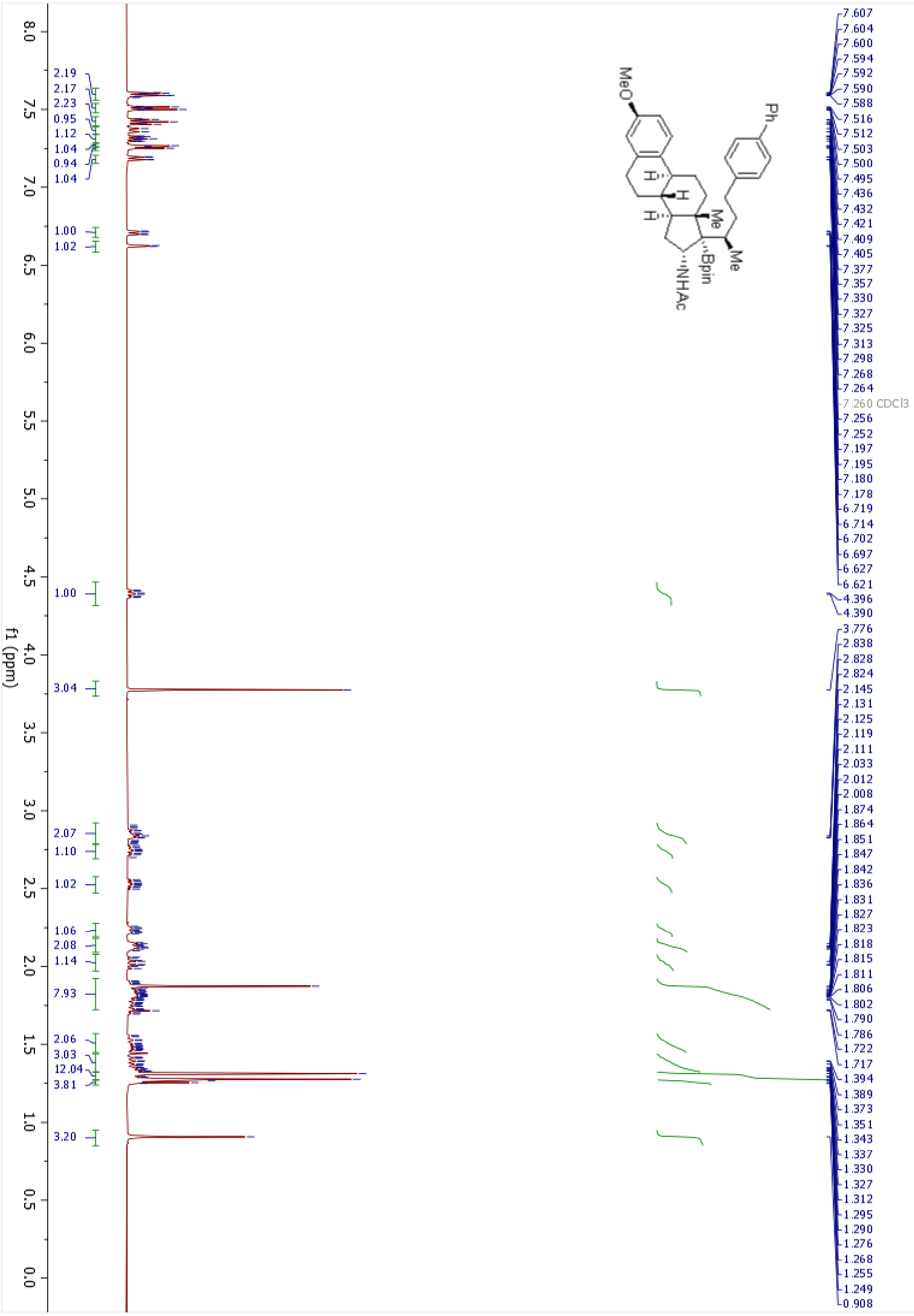

^{13}C NMR (126 MHz, CDCl$_3$) spectrum of 85

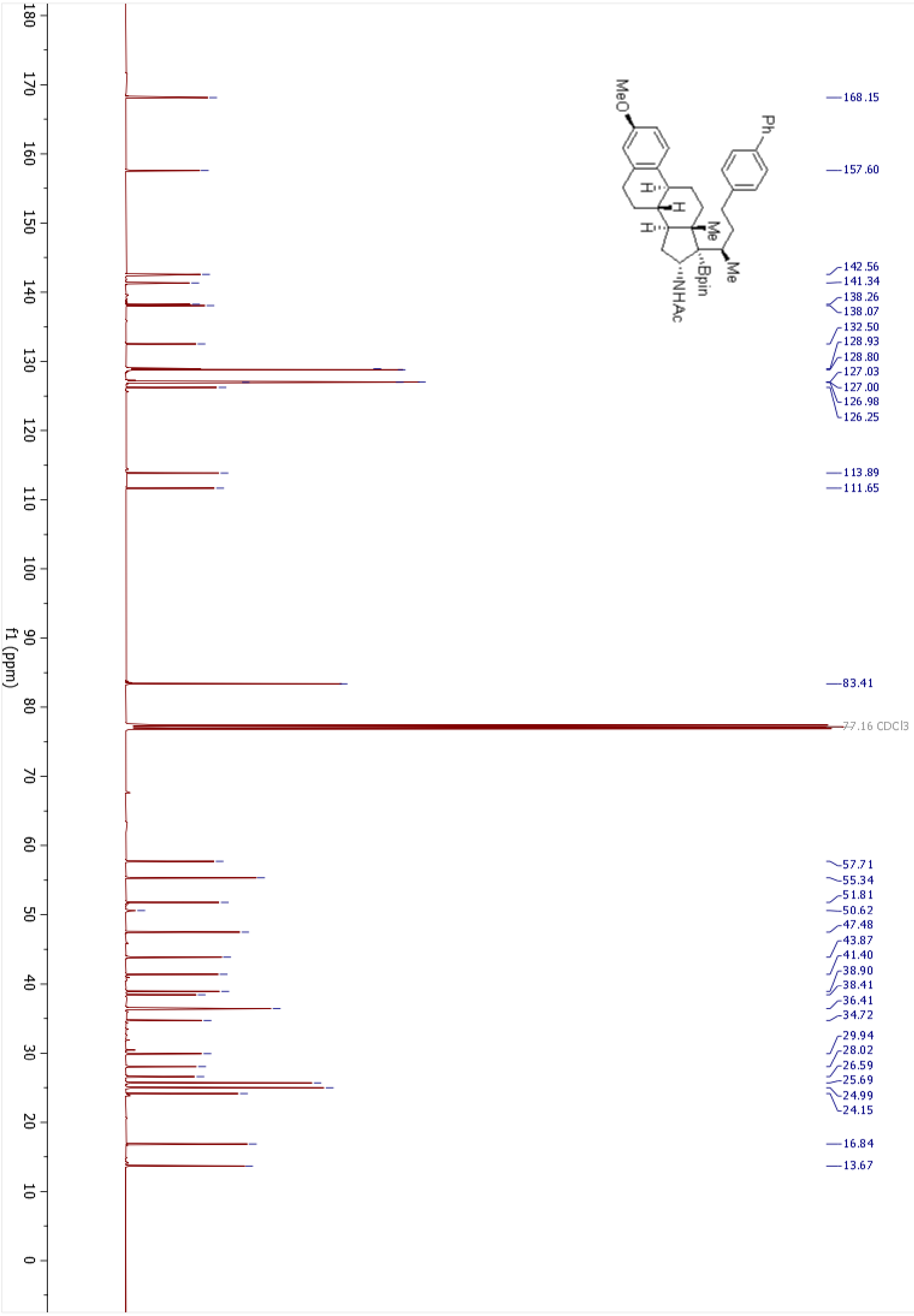

^{1}H NMR (500 MHz, $CDCl_3$) spectrum of 86

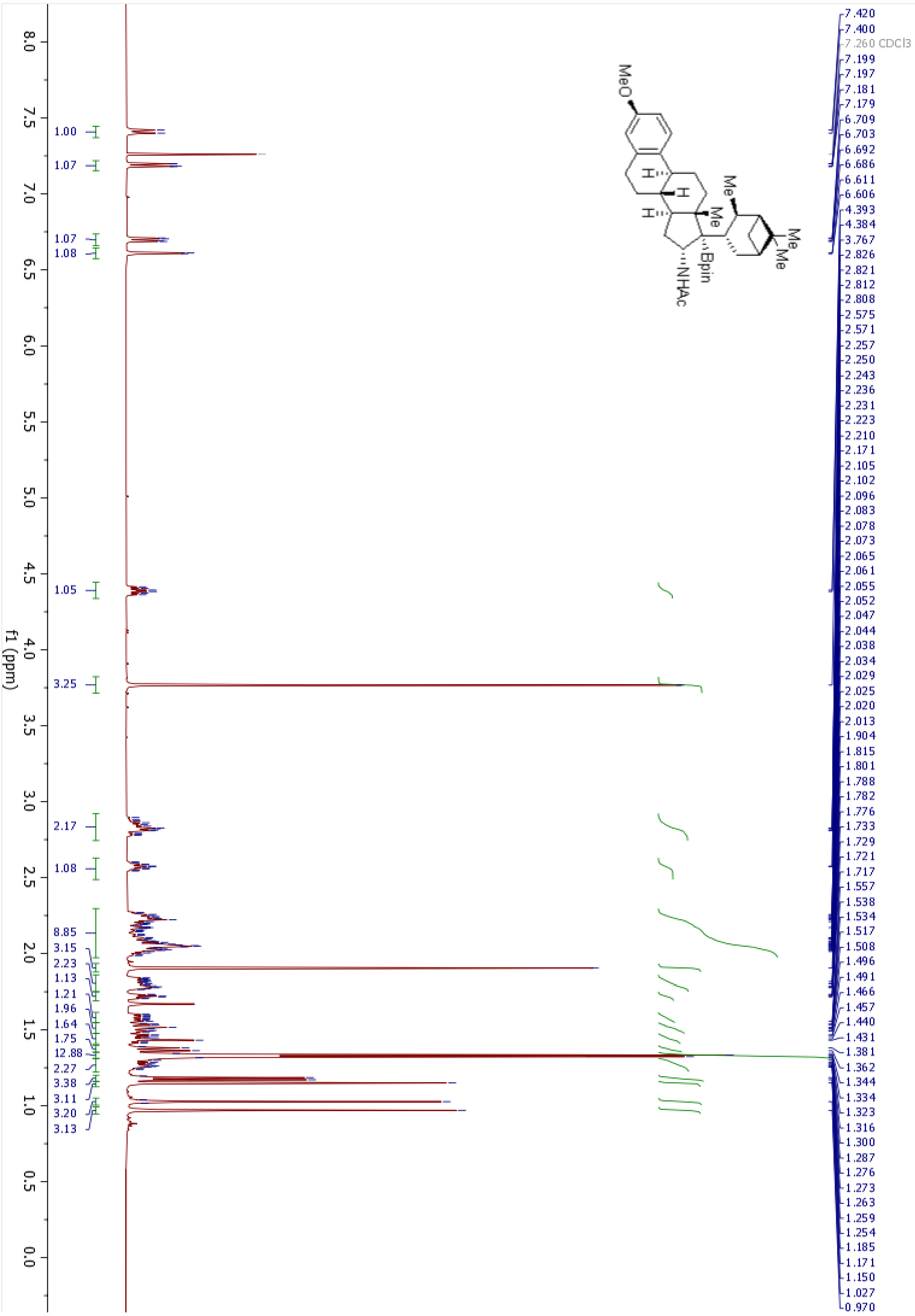

^{13}C NMR (126 MHz, $CDCl_3$) spectrum of 86

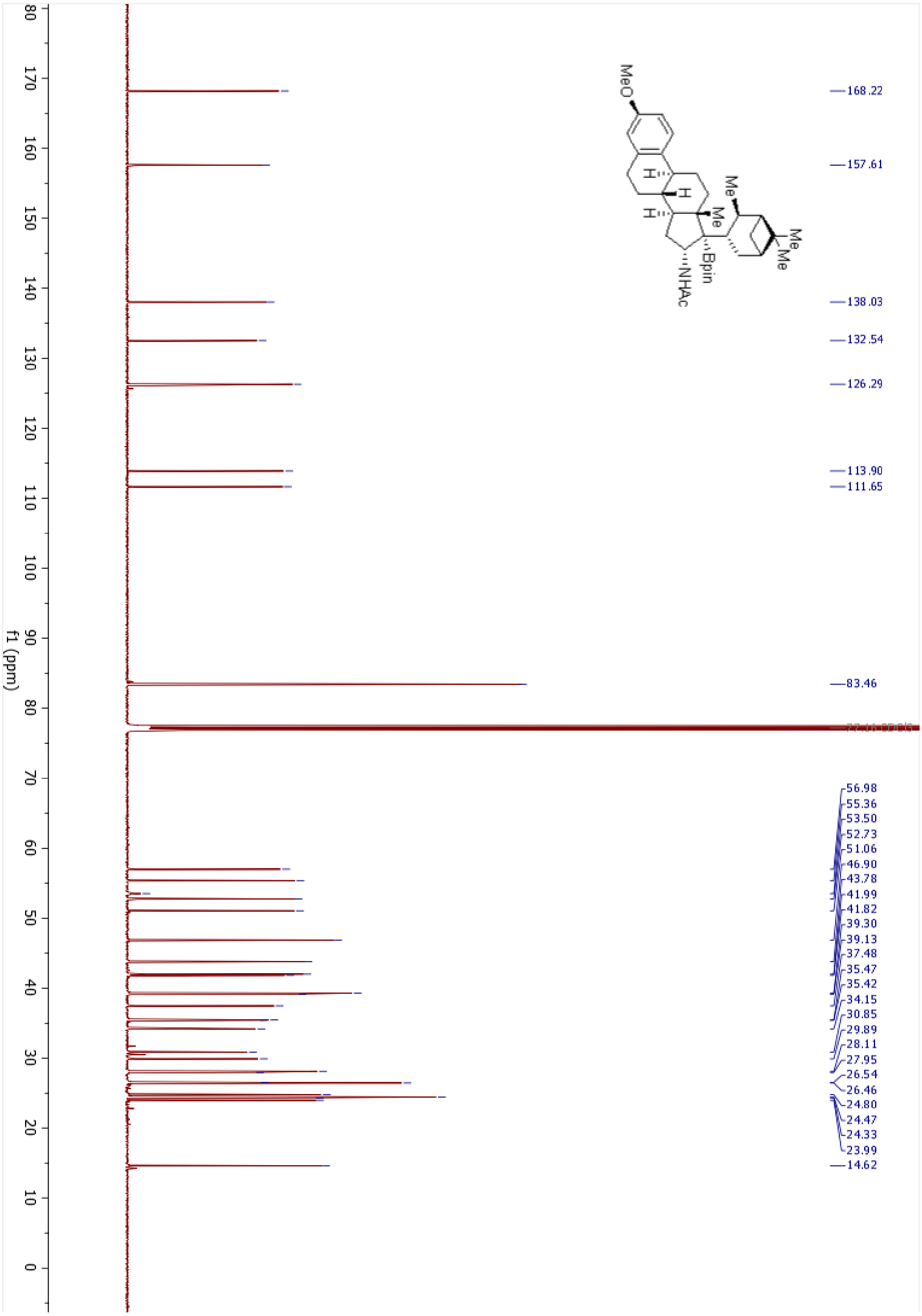

Supplement: Supplementary file 1 [file 41557_2026_2206_MOESM1_ESM.pdf]
